# Elongation factor 1A1 regulates metabolic substrate preference in mammalian cells

**DOI:** 10.1016/j.jbc.2024.105684

**Published:** 2024-01-23

**Authors:** Rachel B. Wilson, Alexandra M. Kozlov, Helia Hatam Tehrani, Jessica S. Twumasi-Ankrah, Yun Jin Chen, Matthew J. Borrelli, Cynthia G. Sawyez, Siddhant Maini, Trevor G. Shepherd, Robert C. Cumming, Dean H. Betts, Nica M. Borradaile

**Affiliations:** 1 Department of Physiology and Pharmacology, Schulich School of Medicine and Dentistry, Western University, London, Ontario, Canada; 2 Department of Biology, Western University, London, Ontario, Canada; 3 The Mary & John Knight Translational Ovarian Cancer Research Unit, London Regional Cancer Program, London, Ontario, Canada; 4 Department of Anatomy and Cell Biology, Schulich School of Medicine and Dentistry, Western University, London, Ontario, Canada; 5 Department of Oncology, Schulich School of Medicine and Dentistry, Western University, London, Ontario, Canada; 6 Department of Obstetrics and Gynaecology, Schulich School of Medicine and Dentistry, Western University, London, Ontario, Canada; 7 Genetics and Development Division, The Children’s Health Research Institute, Lawson Health Research Institute, London, Ontario, Canada

**Keywords:** lipid, lipid metabolism, glucose, glycolysis, translation elongation factor

## Abstract

Eukaryotic elongation factor 1A1 (EEF1A1) is canonically involved in protein synthesis but also has noncanonical functions in diverse cellular processes. Previously, we identified EEF1A1 as a mediator of lipotoxicity and demonstrated that chemical inhibition of EEF1A1 activity reduced mouse liver lipid accumulation. These findings suggested a link between EEF1A1 and metabolism. Therefore, we investigated its role in regulating metabolic substrate preference. EEF1A1-deficient Chinese hamster ovary (2E2) cells displayed reduced media lactate accumulation. These effects were also observed with EEF1A1 knockdown in human hepatocyte-like HepG2 cells and in WT Chinese hamster ovary and HepG2 cells treated with selective EEF1A inhibitors, didemnin B, or plitidepsin. Extracellular flux analyses revealed decreased glycolytic ATP production and increased mitochondrial-to-glycolytic ATP production ratio in 2E2 cells, suggesting a more oxidative metabolic phenotype. Correspondingly, fatty acid oxidation was increased in 2E2 cells. Both 2E2 cells and HepG2 cells treated with didemnin B exhibited increased neutral lipid content, which may be required to support elevated oxidative metabolism. RNA-seq revealed a >90-fold downregulation of a rate-limiting glycolytic enzyme, hexokinase 2, which we confirmed through immunoblotting and enzyme activity assays. Pathway enrichment analysis identified downregulations in TNFA signaling *via* NFKB and MYC targets. Correspondingly, nuclear abundances of RELB and MYC were reduced in 2E2 cells. Thus, EEF1A1 deficiency may perturb glycolysis by limiting NFKB- and MYC-mediated gene expression, leading to decreased hexokinase expression and activity. This is the first evidence of a role for a translation elongation factor, EEF1A1, in regulating metabolic substrate utilization in mammalian cells.

Protein synthesis is one of the most energetically costly ATP-consuming cellular processes (1, 2). Therefore, it is likely that protein synthesis and metabolism are coordinately regulated. This concept is supported by a growing body of evidence. Systems biology approaches have defined relationships between protein translation efficiency and energy metabolism, initially identified through early comparisons across yeast strains (3). Nitrogen limitation studies indicated that yeast have large reserves to support increased requirements for metabolic and translational capacities and that translational capacity reserves are used to translate mRNAs involved in energy metabolism (4). Further work using metabolic modeling demonstrated that strains which rely on aerobic fermentation, rather than respiration, have reduced mitochondrial ribosomal translation efficiencies and lower expression of proteins required for oxidative metabolism (5). Moreover, a network of hypoxia-responsive RNA-binding proteins enhances the translation efficiency of glycolytic mRNAs to support a shift toward anaerobic metabolism under low oxygen conditions (6). Collectively, these findings suggest a relationship between protein translation efficiency and energy metabolism.

In addition to overall translation efficiency, several studies have linked the regulation of energy metabolism to specific translation initiation factors. Eukaryotic initiation factor 6 (EIF6) controls the translation of lipogenic transcription factors, which promote lipogenesis (7, 8), and has been implicated in lactate release and ATP production in various cell types (9, 10). Similarly, EIF4E controls the translation of mRNAs involved in lipid processing, transport, and storage (11) and is implicated in promoting mitochondrial function by translating nuclear-encoded mitochondrial-related mRNAs (12). Finally, EIF5A2 regulates glucose metabolism and lipogenesis in hepatocellular carcinoma cell lines (13), while EIF5B preferentially translates mRNAs involved in central carbon metabolism under hypoxic conditions (14).

Eukaryotic elongation factor 1A (EEF1A) is a critical component of the translational apparatus and has long been recognized for this canonical function. EEF1A also has several noncanonical functions, including regulation of the actin cytoskeleton (15, 16, 17, 18, 19), nuclear tRNA (20) and protein export (21), proteolysis (22), apoptosis (23), and viral propagation (24, 25). EEF1A exists as two paralogs in mammalian systems—EEF1A1 and EEF1A2—the expression of which are mutually exclusive in most adult tissues (26). A ROSAβgeo retroviral promoter trap screen identified that mutant, EEF1A1-deficient Chinese hamster ovary (CHO-K1) cells were resistant to palmitate-induced lipotoxicity, a form of metabolic stress (27). These cells did not exhibit impaired total protein synthesis, because maintained expression of the EEF1A2 paralog appeared to compensate for this canonical function (27). Furthermore, we have shown that chemical inhibition of EEF1A1 reduced hepatic lipid accumulation in two obese mouse models (28, 29). Studies in nonmammalian organisms, which express the single homolog *EEF1A*, suggest a link between this factor and the regulation of metabolic pathways. In *Saccharomyces cerevisiae*, overexpression of EEF1A induced genes involved in glucose metabolism and altered cellular levels of glucose storage molecules trehalose and glycogen (30). In nutritionally stressed *Trypanosoma cruzi*, EEF1A preferentially interacted with mRNAs involved in metabolic processes, including glycolysis (31). Additionally, microarray analyses comparing gene expression in high *versus* low EEF1A genotypes in maize endosperm demonstrated that upregulation of genes involved in carbohydrate metabolism is associated with high EEF1A expression (32). Taken together, these findings raise the possibility that EEF1A1 may have a role in regulating metabolism; however this has not been directly investigated in mammalian cells.

Here, we interrogated the role of EEF1A1 in energy metabolism using EEF1A1-deficient CHO-K1 cells, shRNA-mediated EEF1A1 knockdown in human HepG2 cells, and selective inhibitors of EEF1A, didemnin B, or plitidepsin, in CHO-K1 and HepG2 cells. We show that EEF1A1 impairment by genetic deficiency, targeted knockdown, or chemical inhibition reduced glycolysis, increased fatty acid oxidation, and increased neutral lipid storage. Furthermore, EEF1A1 deficiency promoted a shift toward oxidative metabolism to support cell proliferation and migration. Transcriptomic, biochemical fractionation, and enzyme activity studies indicated reduced NFKB and MYC signaling and decreased hexokinase expression and activity in response to EEF1A1 deficiency, which may underlie the observed glycolytic defects. This is the first evidence demonstrating a direct role of a translation elongation factor in regulating metabolic substrate preference in mammalian cells.

## Results

### EEF1A1-deficient CHO-K1 cells exhibit altered morphology, but a similar proliferation rate compared to WT cells

**Figure 1:**
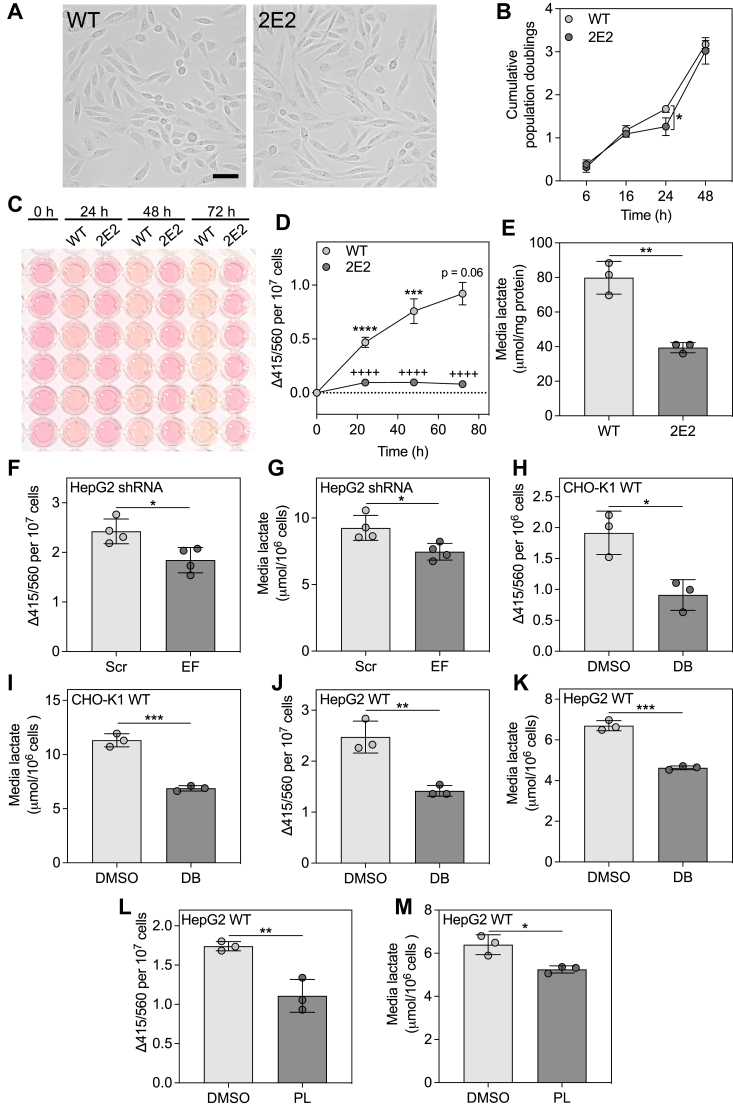
**EEF1A1 deficiency and inhibition reduce media acidification and media lactate.***A*, light micrographs of WT and EEF1A1-deficient (2E2) CHO-K1 cells at low density. The scale bar represents 50 μm. *B*, cumulative population doublings for WT and 2E2 cells at the indicated time points, n = 3. *C*, conditioned media from WT and 2E2 cells over 72 h. *D*, change in 415/560 (Δ415/560) from 0 h to 72 h for WT and 2E2 cell conditioned media, normalized to total cell number, n = 3. ∗∗∗∗*p* < 0.0001 for WT 24 h *versus* WT 0 h; ∗∗∗*p* < 0.001 for WT 48 h *versus* WT 24 h; *p* = 0.06 for WT 72 h *versus* WT 48 h; ++++ *p* < 0.0001 for 2E2 *versus* WT at the corresponding time point. *E*, lactate concentration in conditioned media from WT and 2E2 cells after 24 h, n = 3. *F*, Δ415/560 and (*G*) lactate concentration in conditioned media collected from HepG2 cells expressing scrambled (Scr) and EEF1A1 (EF) shRNA after 48 h, n = 4. *H*, Δ415/560 and (*I*) lactate concentration in conditioned media collected from CHO-K1 WT cells treated with DMSO or didemnin B (DB) (20 nM) for 24 h, n = 3. *J* and *L*, Δ415/560 and (*K* and *M*) lactate concentration in conditioned media collected from HepG2 WT cells treated with DMSO and (*J* and *K*) DB (80 nM) or (*L* and *M*) plitidepsin (PL) (80 nM) for 24 h, n = 3. Data are means ± SD. ∗*p* < 0.05, ∗∗*p* < 0.01. and ∗∗∗*p* < 0.001. CHO, Chinese hamster ovary; EEF1A, eukaryotic elongation factor 1A.

EEF1A1-deficient CHO-K1 cells, designated as 2E2 cells, were previously generated through a genetic screen for resistance to palmitate-induced cell death, and are known to express only EEF1A2 (27). Compared to WT CHO-K1 (CHO WT) cells, EEF1A1-deficient CHO-K1 (CHO 2E2) cells are more elongated with a spindle-like morphology (Fig. 1*A*). This is likely related to the known role of EEF1A1 in cytoskeletal regulation, and the previous observation of increased F-actin in CHO 2E2 cells (27). Proliferation was assessed in CHO WT and 2E2 cells by determining cumulative population doublings at the indicated time points. Compared to CHO WT cells, CHO 2E2 cells exhibited a modest, but significant decrease in proliferation rate at 24 h, but no differences at 6 h, 16 h, and 48 h (Fig. 1*B*).

### EEF1A1 deficiency and inhibition reduce media acidification and media lactate

Conditioned phenol red–containing media were collected from CHO WT and 2E2 cells over a 72 h culture period. Media collected from CHO WT cells became more yellow over time, suggesting increased acidity, while media collected from CHO 2E2 cells remained pink, resembling unconditioned (0 h) medium (Fig. 1*C*). To quantify the difference in media color, we employed a spectrophotometric phenol red–based method. Due to differences in absorbances of acidic and basic media at 415 and 560 nm, the ratio of absorbance at 415 nm *versus* 560 nm can be used as an indication of media acidity (https://www.agilent.com/cs/library/applications/phenol-red-to-assess-ph-in-tissue-culture-media-5994-3391EN-agilent.pdf). The change in the 415/560 ratio (relative to 0 h; Δ415/560) in CHO WT media increased over 72 h, while the Δ415/560 in CHO 2E2 media was unchanged and significantly lower than for CHO WT media at all time points (Fig. 1*D*). This difference cannot be explained by marked differences in proliferation rate (Fig. 1*B*) or cell number (Fig. S1*A*). Media acidification is predominantly driven by the release of acidic metabolic by-products, including lactic acid. Consistent with impaired media acidification, media lactate concentration in CHO 2E2 cells was reduced to less than 50% of that in CHO WT cells (Fig. 1*E*). To confirm the relationship of EEF1A1 to this phenotype, we used HepG2 human hepatoma cells stably expressing an shRNA targeting EEF1A1, which we previously demonstrated resulted in a modest knockdown (−24%) (33). Like CHO 2E2 cells, EEF1A1 knockdown in HepG2 cells reduced media acidification (Fig. 1*F*) and media lactate (Fig. 1*G*); this occurred in the absence of dramatic changes in cell number (Fig. S1*B*). In addition to these genetic approaches, we used a highly selective chemical inhibitor of EEF1A peptide elongation activity, didemnin B. Didemnin B is known to bind EEF1A between domains 1 and 3 and trap it in a posthydrolysis GDP-bound state, which stalls protein translation (34, 35). In CHO WT (Fig. 1, *H* and *I*) and HepG2 WT (Fig. 1, *J* and *K*) cells, incubation with didemnin B resulted in reductions in media acidification (Fig. 1, *H* and *J*) and media lactate concentration (Fig. 1, *I* and *K*) similar to those observed in CHO 2E2 cells and independent of changes in cell number (Fig. S1, *C* and *D*). Furthermore, targeting EEF1A1 with a different inhibitor, plitidepsin, in HepG2 cells elicited similar changes in media colour (Fig. 1*L*) and media lactate concentration (Fig. 1*M*), independently of dramatic changes in cell number (Fig. S1*E*). These findings suggest that EEF1A1 impairment, through genetic deficiency, targeted knockdown, or chemical inhibition causes defects in glycolysis in Chinese hamster and human cells.

### EEF1A1-deficient CHO-K1 cells exhibit reduced glycolysis and increased fatty acid oxidation

**Figure 2:**
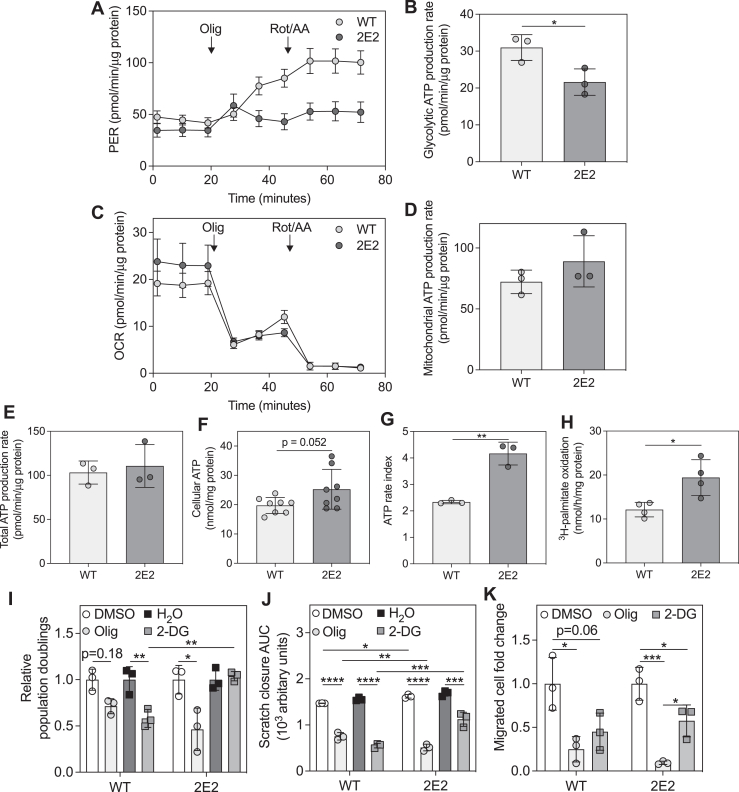
**EEF1A1-deficient CHO-K1 cells exhibit reduced glycolysis and a more oxidative metabolic phenotype.***A*, proton efflux rate (PER) and (*C*) oxygen consumption rate (OCR) for WT and EEF1A1-deficient (2E2) CHO-K1 cells under basal conditions and upon treatment with oligomycin (Olig) or rotenone/antimycin A (Rot/AA), n = 3. *B*, glycolytic, (*D*) mitochondrial, and (*E*) total ATP production rates determined from data in *A* and *C*. *F*, total cellular ATP in WT and 2E2 cell lysates, n = 8. *G*, ATP rate index calculated by dividing the mitochondrial ATP production rate in *D* by the glycolytic ATP production rate in *B*. *H*, fatty acid oxidation in WT and 2E2 cells, n = 4. *I*, WT and 2E2 cell relative population doublings after treatment with oligomycin (Olig) or 2-deoxyglucose (2-DG) for 16 h, n = 3. *J*, areas under scratch closure curves from scratch assays performed over 28 h with WT and 2E2 cells treated as in *I*, n = 3. *K*, transwell migration of WT and 2E2 cells that were treated as in I for 24 h, n = 3. Data are means ± SD. ∗*p* < 0.05, ∗∗*p* < 0.01, ∗∗∗*p* < 0.001, and ∗∗∗∗*p* < 0.0001. CHO, Chinese hamster ovary; EEF1A, eukaryotic elongation factor 1A.

We used a Seahorse ATP rate assay to evaluate the effects of EEF1A1 deficiency on glycolysis and oxidative phosphorylation. The proton efflux rate, which is predominantly driven by glycolysis followed by lactate fermentation, dramatically increased in CHO WT cells after injection of the mitochondrial ATP synthase inhibitor, oligomycin (Fig. 2*A*). This is expected upon oxidative phosphorylation inhibition, as cells are forced to switch to glycolysis to continue generating ATP. Notably, after injection of oligomycin, CHO 2E2 cells failed to substantially increase their proton efflux rate (Fig. 2*A*), suggesting they were unable to effectively switch to glycolysis. Furthermore, CHO 2E2 cells exhibited a significantly lower glycolytic ATP production rate than CHO WT cells (Fig. 2*B*). The oxygen consumption rate (Fig. 2*C*), which is predominantly driven by oxidative phosphorylation, and the mitochondrial ATP production rate (Fig. 2*D*) were variable in CHO 2E2 cells and not significantly different from CHO WT cells. Despite no changes in total ATP production rate (Fig. 2*E*) and total cellular ATP (Fig. 2*F*), CHO 2E2 cells had a significantly higher ATP rate index (mitochondrial ATP production/glycolytic ATP production) (Fig. 2*G*), suggesting that CHO 2E2 cells have a greater preference for oxidative metabolism than CHO WT cells. Consistent with a shift toward oxidative metabolism, CHO 2E2 cells also exhibited significantly higher palmitate oxidation than CHO WT cells (Fig. 2*H*).

### EEF1A1-deficient CHO-K1 cells are less dependent on glycolysis to support proliferation and migration

Given the clear differences in metabolism in CHO 2E2 cells, we explored their dependency on glycolysis compared to oxidative phosphorylation to support two energy-intensive cellular activities, proliferation, and migration. CHO WT cell proliferation was sensitive to inhibition of either oxidative phosphorylation (oligomycin, *p* = 0.18) or glycolysis (2-deoxyglucose) (Fig. 2*I*). In contrast, CHO 2E2 cell proliferation was sensitive to oxidative phosphorylation inhibition, but was unaffected by glycolysis inhibition (Fig. 2*I*), suggesting that CHO 2E2 cells do not rely on glycolysis to maintain proliferation. Correspondingly, scratch assays, which measure both cell migration and proliferation, revealed that scratch closure was drastically slowed in monolayers of CHO WT cells by treatment with either inhibitor (Figs. 2*J* and S2, *A*–*C*). CHO 2E2 cell scratch closure was also slowed by both inhibitors but was less sensitive to inhibition of glycolysis compared to CHO WT cells (Figs. 2*J* and S2, *A*–*C*). Similarly, CHO WT cell migration through transwell inserts was reduced similarly by inhibition of either oxidative phosphorylation or glycolysis (Figs. 2*K* and S3, *A* and *B*), while CHO 2E2 cell migration was more dramatically slowed by oxidative phosphorylation inhibition than glycolysis inhibition (Figs. 2*K* and S3, *A* and *B*). These data support the concept that EEF1A1 deficiency reduces cellular dependence on glycolysis to support energy-intensive cellular functions.

### EEF1A1-deficient CHO-K1 cells maintain scratch closure in response to palmitate

Based on the findings that CHO 2E2 cell proliferation (Fig. 2*I*), scratch closure (Fig. 2*J*), and migration (Fig. 2*K*) were more sensitive to inhibition of oxidative metabolism than inhibition of glycolysis and that CHO 2E2 cells displayed increased fatty acid oxidation (Fig. 2*H*), we tested whether supplementation with palmitate would alter scratch closure in CHO WT and 2E2 cells. We performed scratch assays (Fig. S4) in medium containing 0.1 mM palmitate, which is lower than concentrations typically used to induce lipotoxicity. In response to palmitate, CHO WT cells had significantly impaired scratch closure compared to basal conditions (Fig. S4*D*). In contrast, this impairment was not observed in CHO 2E2 cells (Fig. S4*E*). This difference in scratch closure with palmitate is likely related to the previously demonstrated palmitate-resistance of CHO 2E2 cells (27) and is consistent with the observed increase in palmitate oxidation in these cells (Fig. 2*H*). We also performed these experiments with the addition of the fatty acid oxidation inhibitor, etomoxir, which did not affect scratch closure in CHO WT cells (Fig. S4*D*). *p* values for scratch closure comparisons with etomoxir in CHO 2E2 cells are reported in Fig. S4*E*.

### EEF1A1 deficiency and inhibition promote increased neutral lipid storage

**Figure 3:**
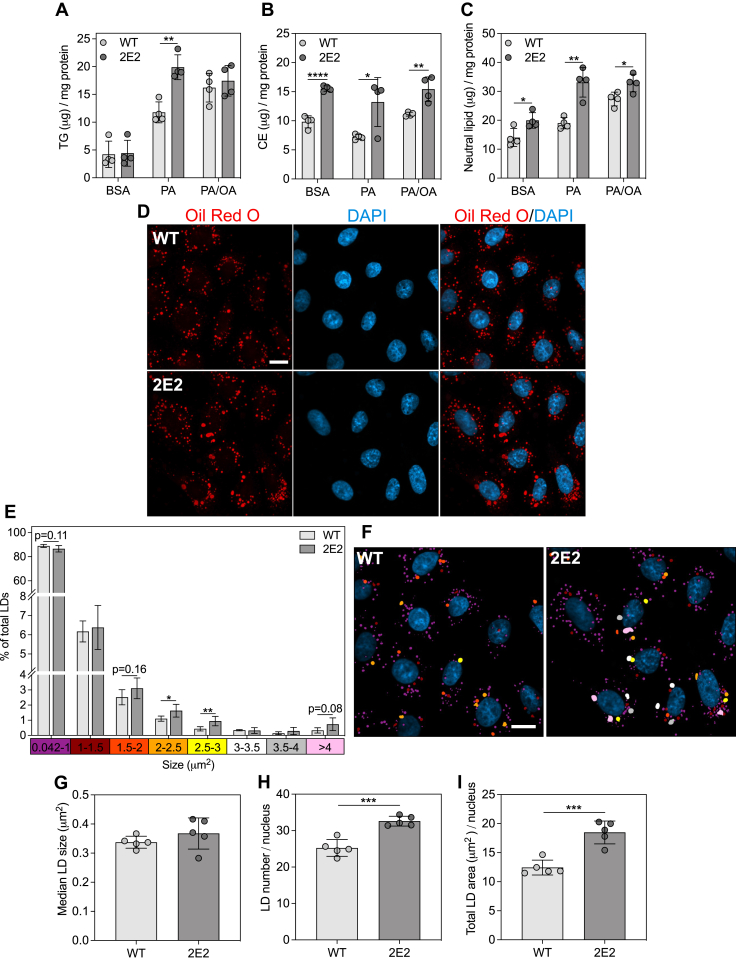
**EEF1A1-deficient CHO-K1 cells exhibit increased neutral lipid accumulation.***A*, triglyceride (TG) and (*B*) cholesteryl ester (CE) masses measured in WT and EEF1A1-deficient (2E2) CHO-K1 cells after treatment with media containing BSA alone or 0.5 mM palmitate (PA) or 0.5 mM palmitate plus oleate (2:3 ratio, PA/OA) for 6 h, n = 4. *C*, total neutral lipid mass calculated as the sum of TG and CE, n = 4. *D*, confocal micrographs of WT and 2E2 cells stained with Oil Red O (neutral lipid, *red*) and DAPI (nuclei, *blue*) after 6 h treatment with 0.5 mM PA/OA. The scale bar represents 10 μm. *E*, lipid droplet (LD) size distributions generated using particle analysis in ImageJ, n = 5. *F*, images from *D* colourized based on the LD size bins and corresponding colors from the *x*-axis in *E*. The scale bar represents 10 μm. *G*, median LD size, (*H*) LD number, and (*I*) total LD area determined from data in *E*. Data are means ± SD. ∗*p* < 0.05, ∗∗*p* < 0.01, ∗∗∗*p* < 0.001, and ∗∗∗∗*p* < 0.0001.

**Figure 4:**
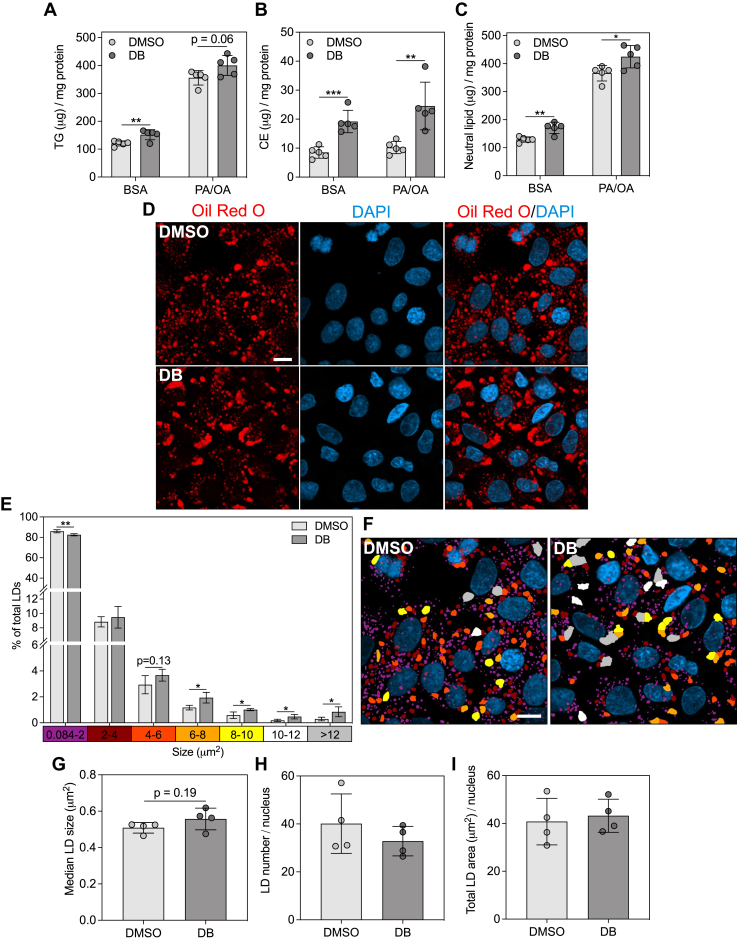
**EEF1A1 inhibition increases neutral lipid accumulation in HepG2 cells.***A*, triglyceride (TG) and (*B*) cholesteryl ester (CE) masses measured in HepG2 cells pretreated with DMSO or didemnin B (DB) (80 nM) in basal media for 24 h, followed by treatment with media containing BSA alone or 1 mM palmitate plus oleate (2:3 ratio, PA/OA) with DMSO or DB for 6 h, n = 5. *C*, total neutral lipid mass, calculated as the sum of TG and CE, n = 5. *D*, confocal micrographs of HepG2 cells stained with Oil Red O (neutral lipid, *red*) and DAPI (nuclei, *blue*) after pretreatment with DMSO or DB (80 nM) in basal media for 24 h, followed by treatment with media containing PA/OA with DMSO or DB for 6 h. The scale bar represents 10 μm. *E*, lipid droplet (LD) size distributions, generated using particle analysis in ImageJ, n = 4. *F*, images from *D* colorized based on the LD size bins and corresponding colors from the *x*-axis in *E*. The scale bar represents 10 μm. *G*, median LD size, (*H*) LD number, and (*I*) total LD area determined from data in *E*. Data are means ± SD. ∗*p* < 0.05, ∗∗*p* < 0.01, and ∗∗∗*p* < 0.001. CHO, Chinese hamster ovary; EEF1A, eukaryotic elongation factor 1A.

Given the metabolic alterations in CHO 2E2 cells which suggest increased dependence on fatty acids for energy generation, we investigated cellular neutral lipid storage and cytosolic lipid droplet size. Biochemical measurements of lipid mass revealed that CHO 2E2 cells accumulated more triglyceride in response to palmitate (Fig. 3*A*). This finding, combined with the evidence of increased palmitate oxidation in these cells (Fig. 2*H*), suggests an improved capacity for palmitate metabolism, which may partly underlie the mechanism through which EEF1A1 deficiency promotes resistance to palmitate-induced lipotoxic cell death (27). Additionally, under both basal and high fatty acid conditions, CHO 2E2 cells stored more cholesteryl ester (Fig. 3*B*) and total neutral lipid (Fig. 3*C*). Correspondingly, lipid droplet size measurements, using Oil Red O staining followed by confocal microscopy, revealed a shift toward larger lipid droplets (Fig. 3, *D*–*F*), increased lipid droplet number (Fig. 3*H*), increased total lipid droplet area (Fig. 3*I*), but no change in median lipid droplet size (Fig. 3*G*), in CHO 2E2 cells. In HepG2 WT cells, we observed increased triglyceride (Fig. 4*A*) (high fatty acid *p* = 0.06), cholesteryl ester (Fig. 4*B*), and total neutral lipid (Fig. 4*C*) under basal and high fatty acid conditions upon chemical EEF1A1 inhibition with didemnin B. Treatment with didemnin B also promoted a shift toward larger lipid droplets (Fig. 4, *D*–*F*). There were no significant changes in median lipid droplet size (Fig. 4*G*), lipid droplet number (Fig. 4*H*), and total lipid droplet area (Fig. 4*I*). The discrepancy between this latter finding and the biochemical measurements of neutral lipid (Fig. 4, *A*–*C*) is likely related to the lower sensitivity of cytological stains than biochemical measurements. Overall, these findings demonstrate that impairment of EEF1A1, either through genetic deficiency or chemical inhibition, increases neutral lipid storage. This suggests a role for EEF1A1 in regulating neutral lipid storage and/or that EEF1A1 impairment elicits an adaptive response, which may be required to maintain the more oxidative metabolic phenotype.

### Impaired glycolysis in EEF1A1-deficient CHO-K1 cells is not associated with reduced AKT activation

We next investigated the mechanism underlying the relationship between EEF1A1 and energy metabolism. Interestingly, EEF1A2 has been shown to activate AKT (36). AKT is well known to upregulate glycolysis both by HK2 phosphorylation (37) and by increasing translation of HIF1A, which can, in turn, promote transcription of its target genes encoding glycolytic enzymes (38). We hypothesized that EEF1A1 may promote AKT activation similarly to EEF1A2 and that the glycolytic defects observed in CHO 2E2 cells may be explained by reduced AKT signaling. However, in CHO 2E2 cells, we observed increased AKT phosphorylation at Ser473 (Fig. S5, *A* and *B*), which suggests increased AKT activation. Therefore, the glycolytic defects observed in CHO 2E2 cells cannot be explained by reduced AKT activation.

### Transcriptomic analyses reveal downregulation in NFKB and MYC signaling, among others, in EEF1A1-deficient CHO-K1 cells

**Figure 5:**
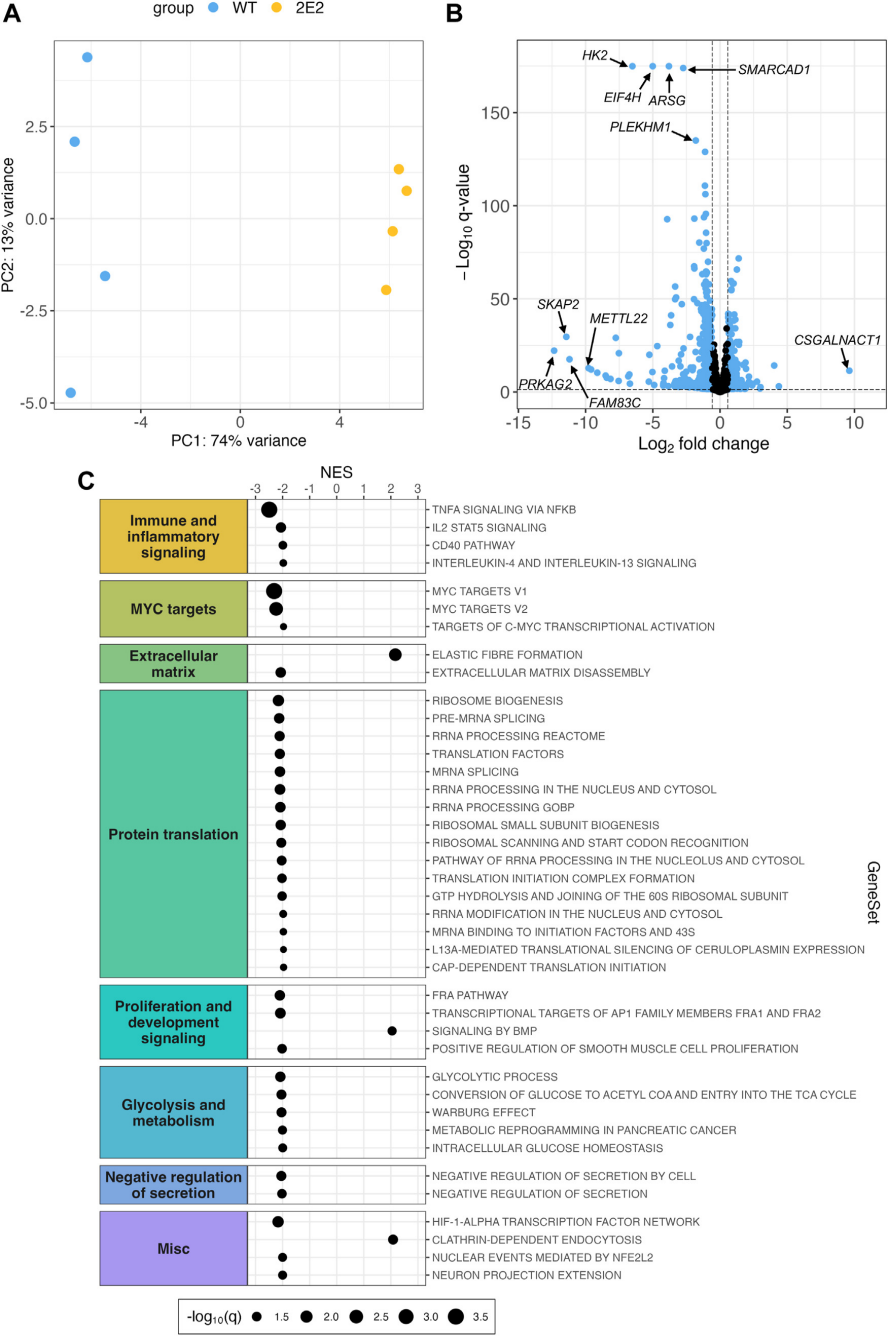
**RNA-seq and gene set enrichment analysis reveal transcriptomic alterations in NFKB and MYC signaling, among others, in EEF1A1-deficient CHO-K1 cells.***A*, principal component (PC) analysis scores plot of RNA-seq data in WT (*blue*) and EEF1A1-deficient (2E2) (*yellow*) CHO-K1 cells, n = 4. *B*, *volcano plot* with data points representing gene transcripts altered in 2E2 relative to WT cells. Genes altered at a q value <0.05 and a fold change >1.5 are indicated in *blue*. Labels correspond to the *top* five most significantly altered genes and the genes with the top five largest absolute fold changes. *C*, *bubble plot* of gene set enrichment analysis representing pathways altered in 2E2 cells relative to WT cells. Normalized enrichment scores (NES) are plotted on the *x*-axis, and gene sets are plotted on the *y*-axis. Bubble size corresponds to −log_10_(q value) (increases as q value decreases). CHO, Chinese hamster ovary; EEF1A, eukaryotic elongation factor 1A; HK2, hexokinase 2.

To gain broader insight into potential mechanisms underlying the glycolytic impairment in EEF1A1 deficiency, we performed RNA-seq in CHO WT and 2E2 cells. The entire gene list containing fold changes and q values generated using *DESeq2* is provided (Table S1). Principal component analysis showed that most of the variance in the data is explained by cell line differences, indicating the high quality of the data (Fig. 5*A*). Genes altered in CHO 2E2 cells relative to CHO WT cells were plotted based on their significance and fold change (Fig. 5*B*). Among the top five most significant gene alterations in CHO 2E2 cells was a 92-fold downregulation in *hexokinase 2 (HK2)*, which encodes a rate-limiting enzyme in glycolysis. This finding suggested a potential mechanism for the observed downregulation of glycolysis in CHO 2E2 cells, as hexokinase has been shown to control glycolytic flux (39). Gene set enrichment analysis revealed that 40 gene sets were significantly altered at a q value <0.05 in CHO 2E2 cells compared to CHO WT cells, the majority (37/40) of which were downregulated (Fig. 5*C*). The two most highly significant pathway alterations, which also had the two largest absolute normalized enrichment scores, were downregulations in “TNFA signaling *via* NFKB” and “MYC targets V1.” CHO 2E2 cells also exhibited downregulations in pathways related to protein translation and glycolysis, among others.

### EEF1A1-deficient CHO-K1 cells exhibit reduced nuclear localization of NFKB family member, RELB, and MYC, and reduced HK2 expression and hexokinase activity

**Figure 6:**
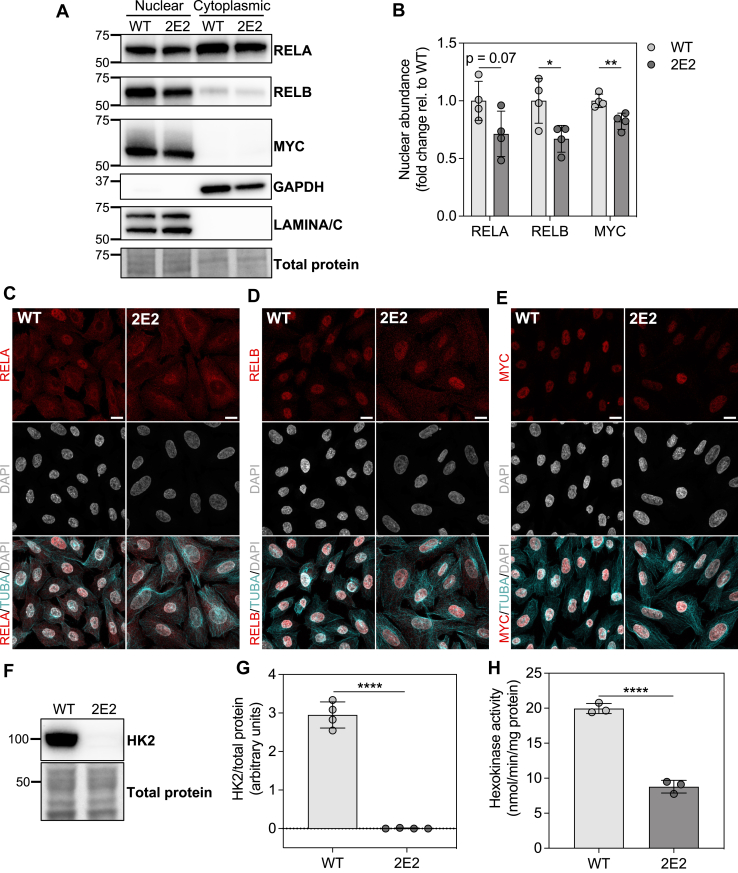
**EEF1A1-deficient CHO-K1 cells exhibit reduced nuclear abundance of RELB and MYC, reduced whole-cell HK2 abundance, and reduced hexokinase activity.***A*, representative immunoblots of nuclear and cytoplasmic fractions (probed for RELA, RELB, and MYC) prepared from WTand EEF1A1-deficient (2E2) CHO-K1 cells. *B*, densitometric analysis of RELA, RELB, and MYC in WT and 2E2 nuclear fractions, n = 4. *C*–*E*, confocal micrographs of WT and 2E2 cells stained for (*C*) RELA, (*D*) RELB, or (*E*) MYC (*red*), TUBA (*cyan*), and DAPI (*gray*). The scale bar represents 10 μm. *F*, representative immunoblots of whole-cell lysates probed for HK2 prepared from WT and 2E2 cells. *G*, densitometric analysis of HK2 in WT and 2E2 whole-cell lysates, n = 4. *H*, hexokinase activity determined by an enzymatic, colorimetric assay in WT and 2E2 cells, n = 3. Data are means ± SD. ∗*p* < 0.05, ∗∗*p* < 0.01, and ∗∗∗∗*p* < 0.0001. Molecular weight markers in kilodaltons indicated on the *left side* of each membrane image. CHO, Chinese hamster ovary; EEF1A, eukaryotic elongation factor 1A; HK2, hexokinase 2.

Based on the gene set enrichment analysis and the knowledge that NFKB (40, 41, 42, 43, 44) and MYC (45, 46) are transcription factors that regulate glycolysis, we evaluated the nuclear abundance of NFKB family members and MYC in CHO WT and 2E2 cells. CHO 2E2 cells showed reduced nuclear abundances of RELB (Fig. 6, *A* and *B*), which is involved in noncanonical NFKB signaling (47), and MYC (Fig. 6, *A* and *B*). The decrease in nuclear abundance of RELA, which is involved in canonical NFKB signaling (47), did not reach statistical significance (Fig. 6, *A* and *B*, *p* = 0.07). These differences occurred independently of alterations in whole-cell protein expression (Fig. S5, *D* and *E*) and were supported by confocal microscopy images showing decreased nuclear signal for RELA (Fig. 6*C*), RELB (Fig. 6*D*), and MYC (Fig. 6*E*) in CHO 2E2 cells. Consistent with prior evidence that NFKB (40, 48) and MYC (49) control expression of HK2, we observed decreased *HK2* mRNA (Fig. 5*B*) and protein (Fig. 6, *F* and *G*) abundances and decreased hexokinase activity (Fig. 6*H*) in CHO 2E2 cells. Combined with the RNA-seq data (Fig. 5), these data indicate that NFKB- and MYC-associated transcriptional programs, along with HK2 expression and hexokinase activity, are downregulated in CHO 2E2 cells, potentially underlying the glycolytic defects resulting from EEF1A1 deficiency.

## Discussion

**Figure 7:**
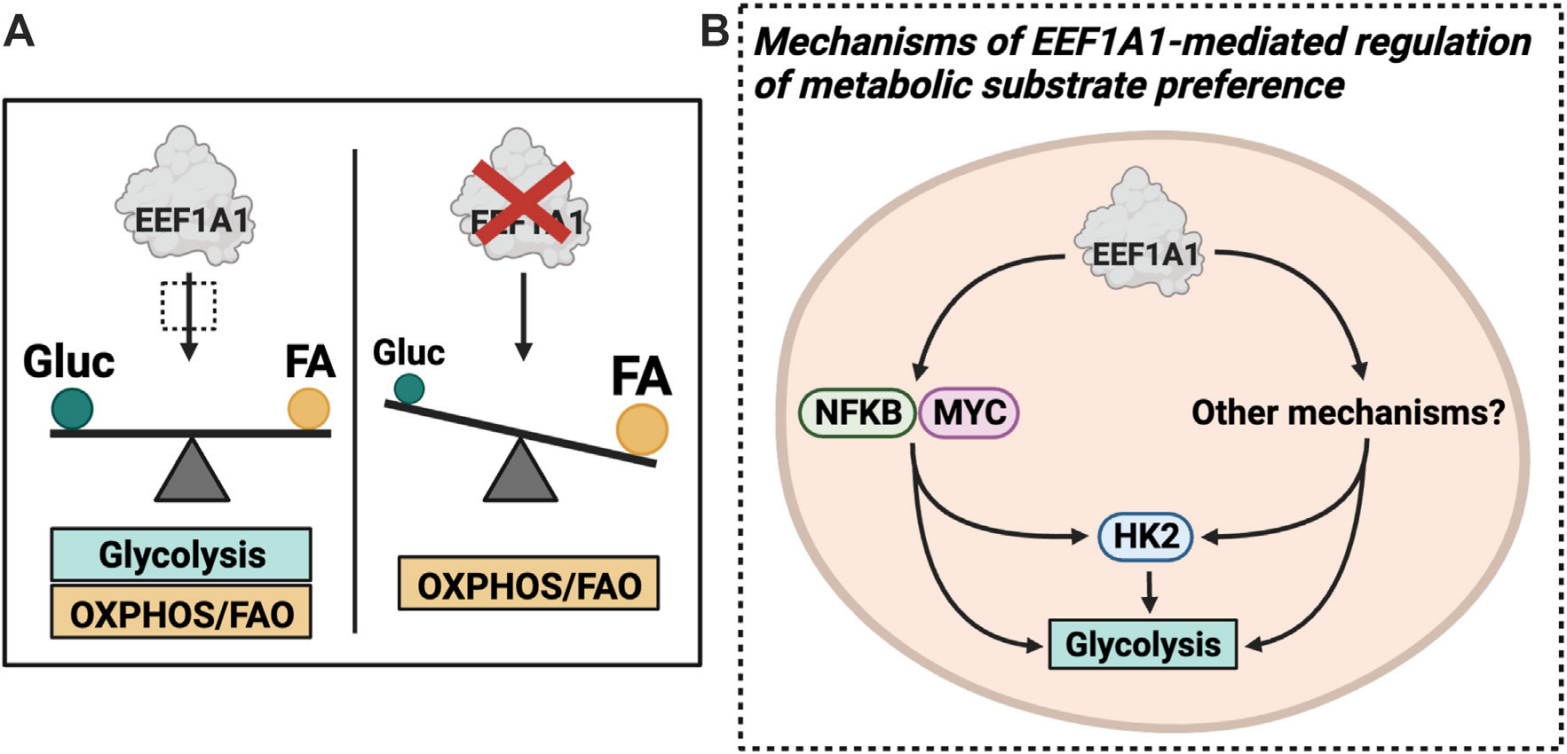
**Working model for regulation of metabolic substrate preference by EEF1A1.***A*, EEF1A1 regulates metabolic substrate preference. When EEF1A1 is intact, both glycolysis and oxidative metabolism are used for energy generation. When EEF1A1 is perturbed using genetic or chemical strategies, glycolysis is impaired and oxidative metabolism (OXPHOS) and fatty acid β oxidation (FAO) are preferred for energy generation. *B*, inset of A (*dashed outline*) summarizing potential mechanisms through which EEF1A1 regulates metabolic substrate preference. EEF1A1 deficiency impairs glycolysis, reduces hexokinase 2 (HK2) expression and hexokinase activity, and reduces NFKB and MYC signaling. EEF1A1 may promote glycolysis by promoting NFKB and MYC signaling and hexokinase expression and activity. EEF1A1 likely also regulates metabolic substrate preference through other mechanisms. Created with Biorender.com. CHO, Chinese hamster ovary; EEF1A, eukaryotic elongation factor 1A.

Our work shows that CHO-K1 cells lacking EEF1A1 exhibited impaired glycolysis, a distinct preference for oxidative metabolism to support energy-requiring activities and increased neutral lipid storage (Fig. 7*A*). We observed similar effects upon targeted EEF1A1 knockdown in human hepatocyte-like HepG2 cells and in WT CHO-K1 and HepG2 cells treated with selective inhibitors of EEF1A, didemnin B, or plitidepsin. Furthermore, transcriptomic, subcellular fractionation, and enzyme activity studies indicated reduced NFKB and MYC signaling alongside decreased HK2 expression and hexokinase activity in the setting of EEF1A1 deficiency, which may contribute to the glycolytic defects observed in these cells (Fig. 7*B*). Our findings are the first evidence of a role for a translation elongation factor in regulating metabolic substrate utilization in mammalian cells.

We explored the relationship between EEF1A1 and energy metabolism using two approaches: (1) genetic, *via* EEF1A1-deficient CHO-K1 cells generated through a promoter trap screen and HepG2 cells stably expressing an EEF1A1-targeting shRNA and (2) chemical, *via* inhibition of EEF1A with didemnin B or plitidepsin in WT CHO-K1 cells and human HepG2 cells. Didemnin B is a highly selective inhibitor of EEF1A and has been shown by cryo-EM to bind EEF1A and stall protein translation (34, 35). Its only other known target is palmitoyl protein thioesterase 1, with an IC_50_ that greatly exceeds the dose that has been used in the present work (50). Therefore, inhibition of palmitoyl protein thioesterase 1 by didemnin B is unlikely to have contributed to our findings. Furthermore, confirmation of the phenotype with a different EEF1A inhibitor, plitidepsin, further suggests the metabolic alterations upon chemical inhibition are directly related to inhibition of EEF1A. Additionally, demonstration of a phenotype similar to that observed in EEF1A1 deficiency with targeted EEF1A1 knockdown and highly selective EEF1A inhibition suggests that the metabolic phenotype is caused directly by loss of EEF1A1, as opposed to by an adaptation acquired downstream of EEF1A1 deficiency. Lastly, we observed this phenotype in both Chinese hamster and human cells, suggesting the role of EEF1A1 in regulating metabolism is reasonably well conserved at least between mammalian species.

The finding in the present study, that EEF1A1 perturbation by deficiency or chemical inhibition with didemnin B increased cellular neutral lipid storage, is discrepant with our prior finding of reduced hepatic lipid accumulation in response to didemnin B in obese mice (29). We think this discrepancy may relate to differences between the mouse and cell culture models used. In our previous work, didemnin B was used as an intervention for existing hepatic steatosis, and thus substantial hepatic lipid accumulation existed at the initiation of treatment. In the present study, EEF1A1 perturbation occurred prior to cellular lipid accumulation. Therefore, the effects of EEF1A1 perturbation on lipid accumulation may vary depending on whether lipid is present in the system at treatment initiation. Another consideration is the duration of didemnin B treatment in each model. Mice with western diet-induced hepatic steatosis were treated with didemnin B once every 3 days for a 2-week period (29). In the present study, HepG2 cells were treated with didemnin B for a total of 30 h prior to measuring lipid accumulation. Therefore, our cell culture findings could represent the acute effects of didemnin B on lipid accumulation, while our observations *in vivo* may demonstrate its chronic effects. It is possible that the acute effects of didemnin B in mouse liver, for example, 24 h after the first treatment, would recapitulate our cell culture observations. Based on our findings in the present study, didemnin B may acutely elicit a switch in metabolic substrate preference in mouse liver in which hepatocytes favour fatty acid utilization and store fatty acids in lipid droplets to support this preference. Over time, continued fatty acid oxidation would deplete accrued liver triglyceride, which would be consistent with the chronic effects we observed with didemnin B *in vivo* (29). While this explanation is plausible, additional studies are required to support this concept.

We initially tested the possibility that an AKT-dependent mechanism determines the relationship between EEF1A1 and glycolysis based on earlier work showing that EEF1A2 overexpression activates AKT in human breast cancer cells (36). We reasoned that if a similar relationship exists between EEF1A1 and AKT, this may provide a mechanism for the glycolytic defects and downregulated HK2 expression observed in CHO 2E2 cells. However, we observed increased phosphorylation of AKT at Ser473 (Fig. S5, *A* and *B*), suggesting increased activity, in EEF1A1-deficient cells. Therefore, AKT regulation does not drive the glycolytic defect observed in EEF1A1 deficiency, and EEF1A1 does not appear to have the same function as EEF1A2 in AKT activation in CHO-K1 cells.

We turned to RNA-seq to explore potential mechanisms more comprehensively. We observed highly significant downregulations in NFKB and MYC signaling in EEF1A1-deficient cells; these findings were supported by subcellular fractionation studies. There is strong evidence from previous work suggesting that these pathway alterations could explain the glycolytic defects observed in EEF1A1 deficiency. In models of hepatitis B virus–related hepatocellular carcinoma (40) and in sarcoma cell lines (48), classical NFKB signaling (RELA) controls metabolic substrate preference in favor of glycolysis through transcriptional upregulation of HK2. RELA is also required for enhanced aerobic glycolysis upon loss of p53 in mouse embryonic fibroblasts through upregulation of *Glut3* expression (41). Additionally, noncanonical NFKB signaling through RELB contributes to doxorubicin-induced glycolysis in doxorubicin-resistant diffuse large B-cell lymphoma cell lines (42). Given that MYC is a master transcriptional regulator of glycolysis (46) and specifically HK2 transcription (49, 51), it is plausible that downregulated NFKB and MYC signaling contribute to the glycolytic defects we observed (Fig. 7*B*). Interestingly, MYC has been shown to interact with EEF1A1 through coimmunoprecipitation and mass spectrometry studies (52), suggesting a potential direct relationship between the two proteins. Although a direct link has not been demonstrated between EEF1A1 and NFKB, EEF1A1 has been shown to promote IL6 expression, which is classically regulated by NFKB signaling (53).

We further observed that EEF1A1 deficiency promotes increased fatty acid oxidation and neutral lipid accumulation. These findings are consistent with studies in C2C12 rat myotubes in which EEF1A2 knockdown increased mRNA expression of genes involved in fatty acid oxidation and *de novo* lipogenesis (54). The underlying mechanism for these effects on lipid metabolism could relate to the downregulation of MYC targets and decreased MYC nuclear abundance we observed, as previous work has linked MYC to the regulation of lipid metabolism. *MYC* deficiency promotes lipid droplet accumulation in human neuroblastoma cells (55), and increases palmitate uptake and oxidation, and neutral lipid storage in human alveolar lung cancer cells (56). Moreover, mice with hepatic *Myc* deficiency exhibit increased hepatic fatty acid oxidation and increased triglyceride and lipid droplet accumulation upon fasting (57). Thus, it is plausible that decreased MYC signaling in EEF1A1-deficient CHO-K1 cells contributes to coordinated increases in lipid storage and utilization.

Given the high abundance of EEF1A (1–2% of total cellular protein) (58), there may be additional mechanisms that contribute to the regulation of metabolic substrate preference by EEF1A1 (Fig. 7*B*). Based on the growing body of evidence linking protein translation efficiency to energy metabolism, EEF1A1 could regulate glycolysis by selectively enhancing the translation efficiency of mRNAs encoding glycolytic enzymes. Alternatively, EEF1A1 deficiency could alter protein translation such that substrate availability is altered, resulting in a metabolic switch toward oxidative phosphorylation. Integrated systems biology approaches could further elucidate the relationship(s) between EEF1A1 and energy metabolism.

## Conclusions

We have identified a role for translation elongation factor EEF1A1 in regulating metabolic substrate preference and lipid storage in Chinese hamster and human cells. Moreover, we have provided insight as to potential NFKB- and MYC-mediated mechanisms, which could underlie the glycolytic defects observed in EEF1A1-deficient cells (Fig. 7). To our knowledge, this is the first translation elongation factor that has been directly linked to functional changes in energy metabolism in mammalian cells. This work has broad implications for our understanding of the relationships between translation elongation and fuel utilization and the potential mechanisms underlying these relationships.

## Experimental procedures

All assay incubations were performed at room temperature unless otherwise indicated.

### Cell culture and fatty acid media supplementation

All cell cultures were maintained at 37 °C and 5% carbon dioxide.

CHO WT and 2E2 cells (27) were a generous gift from Dr Jean Schaffer (Joslin Diabetes Centre, Harvard Medical School). Cells were maintained in a 1:1 mixture of high glucose (4.5 mg/ml) Dulbecco's modified Eagle medium (Thermo Fisher Scientific 11995-065) and Ham’s F12 Nutrient Mix (Thermo Fisher Scientific 11765-054) supplemented with 5% fetal bovine serum (FBS), 50 U/ml penicillin, 50 μg/ml streptomycin (Thermo Fisher Scientific 15140-122), 2 mM L-glutamine (Thermo Fisher Scientific 25030-081), and 500 μM sodium pyruvate (Thermo Fisher Scientific 11360-070). This resulted in final medium concentrations of 16.2 mM glucose, 4.31 mM L-glutamine, and 1.43 mM sodium pyruvate. Cells were passaged every 2 or 3 days using 0.25% trypsin-EDTA (Thermo Fisher Scientific 25200-056). CHO WT and 2E2 cells were between subcultures 14 and 31 when harvested for experiments.

HepG2 cells (WT, scrambled, and EEF1A1 shRNA) were cultured in EMEM (neutral lipid/lipid droplet analyses: Lonza 12-662F; media colour and lactate measurements: ATCC 30-2003) supplemented with 10% FBS, 20 U/ml penicillin, 20 μg/ml streptomycin (Thermo Fisher Scientific 15140-122), 0.125 μg/ml amphotericin B (Thermo Fisher Scientific 15290-018), 2 mM L-glutamine (Thermo Fisher Scientific 25030-081) (for Lonza media only), and 0.7 g/L sodium bicarbonate (Thermo Fisher Scientific 25080-094) (for ATCC media only). This resulted in final medium concentrations of 4.95 mM glucose, 1.8 mM L-glutamine, 0.9 mM sodium pyruvate, and 2 g/L sodium bicarbonate. For HepG2 media color and media lactate experiments, medium was supplemented with glucose to achieve 16 mM to match the glucose load provided to CHO WT and 2E2 cells. Cells were passaged once or twice weekly using 0.25% trypsin-EDTA (Thermo Fisher Scientific 25200-056) with media changes every 2 or 3 days. HepG2 WT cells were between subcultures 18 and 30 when harvested for experiments. HepG2 scrambled and EEF1A1 shRNA cells (target sequence: 5′-AAGTCTGTAATGAAGTGTTAT-3′) were selected by growth in 500 μg/ml hygromycin (Sigma H0654) for 4 days, followed by subculturing on day 5. This protocol elicited complete death of untransfected HepG2 WT cells. HepG2 shRNA cells were between subcultures 2 and 3 after selection when harvested for experiments.

Fatty acid–supplemented media were prepared based on a modification of a prior method (59) as previously described by our group (33, 60). Briefly, a 20 mM solution of fatty acid (palmitic acid: Sigma P5585; oleic acid: Sigma O1383) in 0.01 N NaOH was incubated at 70 °C for 30 min. Additional 1 N NaOH was added dropwise to completely solubilize the fatty acid. Aliquots of 20 mM fatty acid preparations were stored at −20 °C until supplementation into medium. Fatty acids were complexed with 30% fatty acid–free bovine serum albumin (BSA) (Sigma A9205), by dropwise addition, at a 2:1 M ratio, and supplemented into growth medium to achieve either 0.5 mM (CHO WT and 2E2) (27) or 1 mM (HepG2 WT) (29, 60) total fatty acid concentrations.

### Cell proliferation measurement using cumulative population doublings

Cumulative population doublings were determined in CHO WT and 2E2 cells as previously described (29, 60). Cells were seeded at 50,000 cells/well in 6-well plates (2 ml medium/well) and allowed to attach and grow for 24 h, after which the 0 h time point was harvested, followed by the indicated time points for up to 48 h. At each time point, cells were harvested, and live cells were counted using trypan blue exclusion with a hemocytometer. Population doublings were calculated using the following equation: (log10(number of cells harvested) – log10(number of cells at 0 h)) × (log_10_2)^−1^.

### Cell seeding densities for metabolic assays

Despite exhibiting no major differences in cell proliferation over time (Fig. 1*B*), CHO 2E2 cells exhibit slower cell attachment after seeding (Fig. S6*A*). Therefore, at a given point in time, CHO 2E2 cells have had less time attached to the culture surface compared to CHO WT cells, resulting in fewer population doublings over time after seeding in CHO 2E2 cells (24 h: WT 1.50, 2E2 1.28; 48 h: WT 3.09, 2E2 2.69; 72 h: WT 4.70, 2E2 4.08) (Fig. S6*B*). Additionally, HepG2 EEF1A1 shRNA cells appear to grow slightly faster than their control counterparts, and didemnin B and plitidepsin can modestly decrease cell proliferation. Because cell density can influence cellular metabolism, all cells were seeded such that control and experimental (CHO 2E2, EEF1A1 shRNA, or didemnin B) conditions would have comparable cell numbers at the time of harvest (Fig. S1, *A*–*E*).

### Spectrophotometric measurements of media color

#### CHO WT and 2E2 cells

CHO WT and 2E2 cells were seeded in 10 cm dishes (10 ml medium/dish) at densities that would achieve subconfluence (85–95%) at harvest (24 h WT 3.5 × 10^6^, 2E2 4.2 × 10^6^; 48 h WT 1.15 × 10^6^, 2E2 1.55 × 10^6^; 72 h: WT 4.05 × 10^5^, 2E2 5.7 × 10^5^). At the time of harvest, conditioned media were collected, and cells were harvested and counted with a hemocytometer to determine total cell number. Conditioned media and basal medium were plated in a 96-well plate and were allowed to equilibrate at 5% carbon dioxide for at least 2 h. Absorbances were measured at 415, 560, and 750 nm (https://www.agilent.com/cs/library/applications/phenol-red-to-assess-ph-in-tissue-culture-media-5994-3391EN-agilent.pdf) using a SpectraMAX M5 plate reader (Molecular Devices) with SoftMax Pro 7.1.2 software (Molecular Devices) (www.moleculardevices.com). Background (750 nm) was subtracted from optical density values measured at 415 and 560 nm, and 415/560 values were calculated for basal medium and for conditioned media by dividing the corrected 415 nm absorbance values by the corrected 560 nm absorbance values. The change in 415/560 (Δ415/560) was calculated by subtracting the 415/560 of basal medium (0 h) from the 415/560 of conditioned media harvested at 24, 48, and 72 h and was normalized to cell number.

#### HepG2 cells expressing scrambled and EEF1A1 shRNA

HepG2 scrambled and EEF1A1 shRNA cells were seeded in 6-well plates (2 ml medium/well) at densities that would achieve near confluence (95%) after 4 days (Scrambled: 6–7.5 × 10^5^, EEF1A1: 6 × 10^5^). Two days after seeding, spent medium was replaced with fresh medium (2 ml/well) and cells were incubated for an additional 48 h. At the end of the incubation period, conditioned media were collected and Δ415/560 values and cell numbers were determined as described for CHO WT and 2E2 cells.

#### CHO WT cells treated with didemnin B

CHO WT cells were seeded in 6-well plates (2 ml medium/well) at densities that would achieve subconfluence (85–95%) 48 h later (dimethyl sulfoxide (DMSO): 1.6 × 10^5^, didemnin B: 2.5–2.8 × 10^5^). 24 h after seeding, cells were treated with either didemnin B (Open Chemical Repository, Developmental Therapeutics Program, National Cancer Institute) (20 nM) or DMSO in 2 ml medium for an additional 24 h. This concentration of didemnin B was used as it was the lowest tested concentration to elicit an effect on media color of similar magnitude to EEF1A1 deficiency (Fig. S6*C*). At the end of the incubation period, conditioned media were collected and Δ415/560 values and cell numbers were determined as described for CHO WT and 2E2 cells.

#### HepG2 WT cells treated with didemnin B and plitidepsin

HepG2 WT cells were seeded in 6-well plates (2 ml medium/well) at densities that would achieve near confluence (95%) 4 days later (DMSO: 7.5 × 10^5^, didemnin B: 1 × 10^6^). Three days after seeding, cells were treated with either didemnin B (80 nM), plitidepsin (80 nM) (Cayman Chemical Company 34053), or DMSO in 2 ml media for 24 h. This concentration of inhibitors was chosen because it was previously determined by our group as the IC_50_ of didemnin B for inhibition of protein synthesis in HepG2 cells (33). At the end of the incubation period, conditioned media were collected and Δ415/560 values and cell numbers were determined as described for CHO WT and 2E2 cells.

### Media lactate measurement

#### CHO WT and 2E2 cells

CHO WT and 2E2 cells were seeded in duplicate in 24-well plates (0.5 ml medium/well) at densities that would achieve subconfluence (85–95%) 40 h later (WT: 5 × 10^4^, 2E2: 6.2 × 10^4^). 16 h after seeding, spent medium was replaced with fresh medium (0.5 ml). After 24 h, conditioned media were collected and were deproteinized using the Deproteinizing Sample Preparation Kit, trichloroacetic acid (TCA) (Abcam ab204708) following the supplier’s instructions (100 μl media deproteinized with 15 μl TCA, followed by neutralization with 10 μl neutralization solution), and stored at −80 °C until use. Cell monolayers were washed once with PBS and digested in 200 μl 0.1 N NaOH to solubilize total protein. Total cellular protein was determined by using the Pierce bicinchoninic acid (BCA) Protein Assay Kit (Thermo Fisher Scientific 23225) and used for normalization. We established that the protein-cell ratio is not substantially affected in CHO 2E2 cells (−11%) (Fig. S6*D*) and were therefore confident that total cellular protein was an appropriate surrogate for cell density. Lactate was measured in conditioned media using the L-Lactate Assay Kit (Abcam ab65330) following the supplier’s instructions. Conditioned media samples were diluted appropriately to achieve lactate concentrations in the middle of the standard curve (WT 1/55 or 1/65; 2E2 1/25), and lactate was measured using the colorimetric assay. Preliminary testing revealed minimal background in the samples, so background subtraction was not performed for the experiment.

#### HepG2 cells expressing scrambled and EEF1A1 shRNA

HepG2 scrambled and EEF1A1 shRNA cells were seeded and treated as described for media colour measurements, and conditioned media were deproteinized as described for CHO WT and 2E2 cells. Cells were washed 2X with PBS and digested in 2 ml 0.1 N NaOH to solubilize total protein, and total cellular protein was measured by BCA assay. Protein-cell ratios for HepG2 cells expressing scrambled and EEF1A1 shRNA determined in parallel with the lactate experiments (Fig. S6*E*) were used to calculate cell numbers from total protein values. These calculated cell numbers were used for normalization. Lactate was measured as described for CHO WT and 2E2 cells, with a sample dilution factor of 1/100.

#### CHO WT cells treated with didemnin B

CHO WT cells were seeded in 6-well plates (2 ml medium/well) at densities that would achieve subconfluence (85–95%) 48 h later (DMSO: 1.6 × 10^5^, didemnin B: 2.2 × 10^5^), and treated as described for media colour measurements. Conditioned media were collected and deproteinized as described for CHO WT and 2E2 cells. Cells were washed 2X with PBS and digested in 800 μl 0.1 N NaOH to solubilize total protein, and total cellular protein was measured by BCA assay. Because didemnin B lowers protein-cell ratio by ∼25% in CHO WT cells (Fig. S6*F*), protein-cell ratios for CHO WT cells treated with DMSO or didemnin B for 24 h determined in parallel with the lactate experiments (Fig. S6*F*) were used to calculate cell numbers from total protein values. These calculated cell numbers were used for normalization. Lactate was measured as described for CHO WT and 2E2 cells, with the following sample dilution factors: DMSO 1/55, didemnin B 1/35.

#### HepG2 WT cells treated with didemnin B and plitidepsin

HepG2 WT cells were seeded and treated as described for media colour measurements, and conditioned media were deproteinized as described for CHO WT and 2E2 cells. Total cellular protein was determined as described for HepG2 shRNA cells. Protein-cell ratios for HepG2 cells treated with DMSO, didemnin B, or plitidepsin for 24 h determined in parallel with the lactate experiments (Fig. S6, *G* and *H*) were used to calculate cell numbers from total protein values. These calculated cell numbers were used for normalization. Lactate was measured as described for CHO WT and 2E2 cells, with the following sample dilution factors: DMSO 1/100, didemnin B/plitidepsin 1/75.

### Seahorse XF real-time ATP rate assay

Extracellular measurements of metabolic flux were performed using the Seahorse XF Real-Time ATP Rate Assay Kit (Agilent 103592-100), Seahorse XFe24 FluxPaks mini (Agilent 102342-100), Seahorse XF Dulbecco's modified Eagle's medium assay medium pack (Agilent 103680-100), and a Seahorse XFe24 Analyzer. The day prior to the assay, CHO WT and 2E2 cells were seeded per the supplier’s instructions. Briefly, cells were seeded in triplicate at 30,000 cells/well in an XF24 culture microplate in 100 μl medium and were left on a level surface at room temperature for 1 h to settle. Cells were allowed to attach at 37 °C and 5% CO_2_ for an additional 3 h, after which 150 μl of medium was added to each well. The assay was performed as described in the Agilent Seahorse XF real-time ATP Rate Assay Kit User Guide, with the following modifications: (1) prior to incubation of cell culture microplate in non-CO_2_ incubator, washes were performed gently with a P200 micropipette rather than by aspiration to limit cell detachment and (2) the wash after the 1 h non-CO_2_ incubation was omitted. Once the run was finished, cells were washed once with PBS and were incubated with 35 μl radioimmunoprecipitation assay (RIPA) lysis buffer (150 mM NaCl, 50 mM Tris pH 8, 1% IGEPAL CA-630, 0.5% sodium deoxycholate, 0.1% SDS) on ice for 30 min. After incubation, cells were scraped, and lysates were collected and incubated on ice for an additional 30 min, vortexing every 5 min to lyse cells further. Lysates were centrifuged at 12,000*g* at 4 °C for 10 min and supernatants were collected. Total cellular protein was determined by BCA assay and used for normalization. Calculations of glycolytic and mitochondrial ATP production rates and ATP rate index were performed in Agilent Seahorse Analytics using calculations provided in the Agilent Seahorse XF real-time atp rate assay kit user guide and the improving quantification of cellular glycolytic rate using Agilent Seahorse XF technology white paper.

### Cellular ATP measurement

Total cellular ATP was measured using the ATP Determination Kit (Thermo Fisher Scientific A22066). CHO WT and 2E2 cells were seeded in 6-well plates (2 ml medium/well) in duplicate at densities that would achieve subconfluence (85–95%) 24 h later (WT: 4.5 × 10^5^, 2E2: 5.3 × 10^5^). Cell lysates were harvested using a modification of the methods described by (61, 62). Twenty four hours after seeding, cells were washed once with cold PBS, and harvested by scraping into 500 μl distilled deionized H_2_O on ice. Aliquots were taken for protein measurement and were mixed 1:1 with 0.2 N NaOH to solubilize total protein. The remaining lysate was boiled on a heat block at 100 °C for 10 min. Lysates were vortexed, returned to the ice, and centrifuged at 12,000*g* at 4 °C for 5 min. Supernatants were collected and stored at −20 °C until use. ATP was measured as per the supplier’s instructions, using ATP standards ranging from 1.56 to 100 nM (final concentration). Samples (diluted 1/50) and standards were added to the reaction solution and incubated in the dark for 10 min prior to measuring luminescence using a luminometer (Turner Biosystems, Promega). Cellular ATP values were normalized to total protein, as determined by BCA assay.

### Fatty acid oxidation

Palmitate oxidation was determined by conversion of palmitate to H_2_O, as described by our group and others (60, 63). CHO WT and 2E2 cells were seeded in triplicate in 24-well plates (0.5 ml medium/well) at densities that would achieve subconfluence (85–95%) 24 h later (WT: 1.7 × 10^5^, 2E2: 1.95 × 10^5^). Twenty three hours after seeding, cells were labeled for 1 h in 200 μl medium containing 2 μCi/ml [9,10-^3^H(N)]palmitic acid (PerkinElmer) in 100 μM cold palmitate conjugated to BSA at a molar ratio of 2:1. Media were collected, cells were washed with PBS, and PBS washes were collected into media-containing tubes. Protein was precipitated by the addition of 400 μl 10% trichloroacetic acid and incubation on ice for 30 min. Cell monolayers were lysed by incubation in 100 μl 1 N NaOH at 37 °C for 30 min and were then neutralized with 100 μl 1 N HCl. Cells were removed from the plate using a P200 pipette, and a 50 μl aliquot was taken for protein measurement. The remaining lysates (∼150 μl) were added to tubes containing TCA. Samples were centrifuged at 2200*g* for 10 min. Supernatants were collected, and unreacted fatty acids were removed from samples by four hexane extractions (5 ml hexane per sample per extraction). Radiolabeled aqueous fractions were quantified by liquid scintillation counting (Beckman-Coulter, LS 6500 Multi-Purpose Scintillation Counter). Total protein was determined by BCA assay and used for normalization. Negative controls were performed by preincubating cells with 200 μM etomoxir (Sigma E1905) for 30 min prior to metabolic labelling.

### Cell proliferation experiments with metabolic inhibitors

Cell proliferation was measured using the CyQUANT Cell Proliferation Assay (Thermo Fisher Scientific C7026). CHO WT and 2E2 cells were seeded in triplicate in a 96-well plate (100 μl medium/well) at 23,000 cells/well and 25,000 cells/well respectively and were allowed to settle on a level surface at room temperature for 1 h. Cells were allowed to attach at 37 °C and 5% CO_2_ for an additional 3 h, after which 100 μl of medium was added to each well. Eight hours after seeding (4 h after addition of 100 μl medium), spent medium was removed and cells were treated with 20 mM 2-deoxyglucose (Sigma D6134) (in H_2_O), 1 μM oligomycin A (Sigma 75351) (in DMSO), or the appropriate vehicle controls in fresh medium (200 μl/well) for 16 h at 37 °C and 5% CO_2_. At the end of the incubation, media were removed using a P200 pipette, and cells were gently washed with PBS. After removal of the PBS wash, cells were frozen at −80 °C until measurement. Cell standards for use in the CyQUANT assay were prepared by resuspending CHO WT and 2E2 pellets in 2% FBS in PBS, aliquoting cell suspensions into microfuge tubes to achieve 8.5 × 10^5^ cells per tube, pelleting by centrifugation at 1600*g* for 3 min, removal of the supernatant, and freezing of the remaining cell pellets at −80 °C. Cell numbers were determined using the CyQUANT assay following the supplier’s instructions, with the following specifications: (1) a cell standard curve was generated for both CHO WT and 2E2 cells, ranging from 2.5 × 10^4^ – 1.25 × 10^5^ cells/well, (2) CyQUANT GR dye was used at a 3X final concentration to allow for detection of up to 150,000 cells in a 200 μl volume, and (3) after addition of GR dye/lysis buffer to cells, reactions were incubated in the dark for 5 min. Fluorescence was measured using a SpectraMAX M5 plate reader (Molecular Devices). Cell numbers were calculated for CHO WT and 2E2 cells from the relative fluorescence units using the corresponding cell standard curves. Relative population doublings were calculated as previously described (29, 60).

### Scratch assays

Scratch closure was evaluated following a modification of a previously described method (64). CHO WT and 2E2 cells were seeded in 12-well plates (1 ml/well) at different densities depending on when the scratch would be made the following day (scratch at 22 h postseeding: WT 4.5 × 10^5^, 2E2 5.3 × 10^5^; scratch at 30 h postseeding: WT 3.2 × 10^5^, 2E2 3.8 × 10^5^). After scratches were made, cells were washed once with PBS, incubated with experimental treatments in fresh medium (1 ml/well) (described below), and images were captured at regular intervals over the course of 28 h at 5X magnification using a Leica DM IL LED microscope and Leica Application Suite V4.4 (Leica Microsystems). For scratch assays with inhibitors of glycolysis and oxidative metabolism, cells were treated with 20 mM 2-deoxyglucose, 1 μM oligomycin, or the appropriate vehicle controls. For scratch assays with fatty acids and the fatty acid oxidation inhibitor etomoxir, cells were treated with 0.1 mM palmitate conjugated to BSA at a molar ratio of 2:1 or BSA alone and with 4 μM etomoxir (Sigma E1905) or water. This concentration of etomoxir was chosen because concentrations in this range have been shown to inhibit CPT1 and palmitate oxidation (65, 66) without off-target effects on mitochondrial metabolism that have been shown with concentrations ≥10 μM (65, 66). Furthermore, this concentration is used by Agilent in the Seahorse XF long-chain fatty acid oxidation stress test kit (see corresponding user manual). Percent closure was calculated by expressing the scratch area at each time point as a percentage of the initial scratch area, and areas under the percent closure curves were calculated in GraphPad Prism 9.5.1 (www.graphpad.com).

### Transwell migration assays

CHO WT and 2E2 cells were seeded at 7.5 × 10^4^ cells in 150 μl serum-free medium containing 0.1% BSA and 20 mM 2-deoxyglucose, 1 μM oligomycin, or DMSO in the upper chamber of transwell cell culture inserts (polyester membrane, 8 μm pore size) (Thermo Fisher Scientific C3464). Cell migration was induced over the course of 24 h at 37 °C and 5% CO_2_ using medium containing 20% FBS (600 μl) in the lower chamber. After 24 h, a cotton swab was used to remove unmigrated cells in the upper chamber, and inserts were fixed in cold methanol for 10 min and air dried. Inserts were stained with 0.1% crystal violet (Sigma 61135) (dissolved in 20% methanol) for 10 min, followed by washing with distilled deionized water. Inserts were allowed to air dry and were then imaged at 5X magnification using a Leica DM IL LED microscope and Leica Application Suite V4.4 (Leica Microsystems). Crystal violet stain was extracted with 450 μl 10% acetic acid for 10 min with agitation. Extracts were added in duplicate to a 96-well plate and absorbances were measured at 595 nm using a microplate reader. Migrated cell number was calculated from the absorbance values using CHO WT and 2E2 cell standard curves (described below).

To quantify migrated cell number from crystal violet extract absorbance values, cell number standard curves were generated for CHO WT and 2E2 cells. Cells were seeded in two parallel 48-well plates at increasing densities (0, 3.75 × 10^3^, 7.5 × 10^3^, 1.5 × 10^4^, 3 × 10^4^) in 200 μl medium/well and were allowed to attach and grow for 24 h. Cells in the first 48-well plate were washed twice with PBS at the end of the 24 h period and 2 to 3 times between every subsequent staining step. Cells were fixed with 4% paraformaldehyde for 10 min and stained with 0.1% crystal violet solution for 10 min. Crystal violet stain was extracted and absorbances were measured as described for transwell inserts in the paragraph above. In parallel, cells were harvested from the other 48-well plate using 0.25% trypsin-EDTA and counted with a hemocytometer with the addition of trypan blue. Cell standard curves were generated for CHO WT (R^2^ = 0.981) and CHO 2E2 (R^2^ = 0.997) (Fig. S3, *C* and *D*) cells by plotting the crystal violet absorbance values against the corresponding cell numbers.

### Lipid mass measurements

#### Lipid extract preparation from CHO WT, 2E2, and HepG2 WT cells

CHO WT and 2E2 cells were seeded in 10 cm dishes (10 ml medium/dish) at densities that would achieve subconfluence (85–95%) 30 h later (WT: 2.8 × 10^6^, 2E2: 3.3 × 10^6^). Twenty four hours after seeding, cells were treated with media (8 ml/dish) containing 0.5 mM palmitate or palmitate plus oleate (2:3 ratio) conjugated to BSA at a molar ratio of 2:1 or BSA alone for 6 h. Cellular lipids were extracted using a modification of a previously published method (67). Cells were washed once with 0.2% BSA in PBS, followed by two washes in PBS. After the final PBS wash, liquid was removed completely prior to lipid extraction. Cellular lipids were extracted through two sequential incubations with 4 ml hexane:isopropanol (3:2, v:v) on a rocker for 30 min. Extracts were stored at room temperature until measurement. The remaining cell monolayers were incubated overnight with 5 ml 0.1 N NaOH to solubilize protein. Total protein was measured by BCA assay and used for normalization.

HepG2 WT cells were seeded in 6-well plates (2 ml medium/well) in duplicate at a density that would achieve near confluence (95%) at the time of harvest. Due to variations in cell seeding accuracy or cell growth, cells were seeded at 6 to 7.5 × 10^5^ cells/well and harvested 4 to 6 days after seeding. Cells were pretreated with didemnin B (80 nM) or DMSO in basal medium (2 ml/well) for 24 h, followed by treatment with didemnin B or DMSO in media (2 ml/well) containing BSA or palmitate plus oleate (2:3, 1 mM total fatty acid concentration) for 6 h. Cells were washed with PBS containing 0.2% BSA (2 × 5 min washes), followed by washing with PBS (3 × 5 min washes). Cellular lipids were extracted as described for CHO WT and 2E2 cells with the following modifications: (1) two sequential extractions were performed using 1 ml hexane:isopropanol (3:2, v:v) per well per extraction and (2) cell protein was solubilized with 2 ml 0.1 N NaOH per well.

#### Lipid mass measurement

Cellular triglyceride and cholesteryl ester were measured as previously described (60, 67). Lipid extracts were evaporated at 65 °C to dryness under N_2_ and reconstituted in a chloroform-Triton X-100 (Triton) mixture (0.5% Triton v/v) (CHO WT and 2E2: 700 μl; HepG2 WT: 2.4 ml). The solvent was evaporated at 65 °C to dryness under N_2_, and extract was resolubilized in ddH_2_O (CHO WT and 2E2: 175 μl; HepG2 WT: 600 μl) (2% final Triton concentration in sample). Samples were vortexed and incubated at 37 °C with agitation for 20 min to ensure Triton was resolubilized.

To measure triglyceride mass, samples were diluted appropriately with distilled deionized water containing 2% Triton (CHO WT and 2E2 no dilution; HepG2 WT 1:1.5). Samples were aliquoted (2 × 50 μl) into 96-well plates and triglyceride was measured using a colorimetric, enzymatic, free glycerol-blanked triglyceride assay (Roche, cat#11877771216). Reagent 1 (75 μl) was added to each well and incubated for 10 min to remove free glycerol in samples. Reagent 2 (75 μl) was added to each well and incubated for 50 min. Absorbances were measured at 505 nm and 700 nm. Absorbance values were corrected by subtracting the absorbance values at 700 nm (background) from those at 505 nm.

To measure cholesterol mass, samples were diluted appropriately with distilled deionized water containing 2% Triton (CHO WT and 2E2 1:1; HepG2 WT no dilution). Samples were then aliquoted (2 × 50 μl) into 96-well plates, and total cholesterol and free cholesterol were measured using colorimetric, enzymatic assays (FUJIFILM Wako, total cholesterol: cat# 439-17501, free cholesterol: cat# 435-35801). One hundred fifty microliters of reagent from either kit was added to each well and incubated for 60 min. Absorbances were measured at 600 and 700 nm. Absorbance values were corrected by subtracting the absorbance values at 700 nm (background) from those at 600 nm.

Triglyceride and cholesterol standards (1–20 μg/well) used for standard curves were processed identically to experimental samples. Masses of triglyceride, total cholesterol, and free cholesterol in samples were determined using standard curves. Cholesteryl ester was calculated by subtracting the free cholesterol mass from the total cholesterol mass. Triglyceride and cholesteryl ester masses were normalized to total cellular protein. Neutral lipid mass was calculated as the sum of triglyceride and cholesteryl ester masses.

### Lipid droplet size measurements

#### Lipid droplet staining and confocal microscopy

CHO WT, CHO 2E2, and HepG2 WT cells were grown in 8-well chamber slides (Nunc Lab-Tek II Chamber Slide System, Thermo Fisher Scientific 154534) in 200 μl medium per well. CHO WT and 2E2 cells were seeded at densities that would yield 70 to 80% confluence 30 h later (WT: 3.5 × 10^4^; 2E2: 4 × 10^4^). 24 h after seeding, cells were treated with medium containing 0.5 mM palmitate plus oleate (2:3) for 6 h. HepG2 WT cells were seeded at densities that would yield 70 to 80% confluence at the time of harvest (DMSO: 8 × 10^4^, DB: 9 × 10^4^). Three days after seeding, cells were pretreated with didemnin B (80 nM) or DMSO in basal medium for 24 h, followed by treatment with didemnin B or DMSO in medium containing 1 mM palmitate plus oleate (2:3) for 6 h. At the end of the incubation, CHO WT, CHO 2E2, and HepG2 WT cells were washed twice with PBS, and 2 to 3 PBS washes were also performed between each of the following staining steps. Cells were fixed using 4% paraformaldehyde in PBS for 10 min and permeabilized using 0.1% saponin (Sigma 47036) in PBS for 10 min. Cells were stained using Oil Red O solution (Sigma O1391) diluted in PBS (3:2, v:v) for 10 min. HepG2 WT cells were washed with 60% isopropanol for 30 s, once before and once after Oil Red O staining, as has been done by others for hepatocyte cultures (68). Cells were then stained with 4′,6-diamidino-2-phenylindole (DAPI) (Sigma D1306) (5 μg/ml) for 10 min and coverslips (Fisher Scientific 12-545-F) were mounted on slides using ProLong Glass Antifade Mountant (Thermo Fisher Scientific P36980). Slides were allowed to cure for 24 h and were then sealed with nail polish. Images were captured on a Zeiss LSM 800 confocal microscope with a 63X/1.40 oil objective using the appropriate channels DAPI channel: laser 2.0%, gain 850 V; Oil Red O channel: laser 3.0%; gain 1000 V) and the AiryScan module. The following acquisition parameters were used: resolution = 2696 × 2696, bits per pixel = 16, scan speed = 4, averaging number = 2, averaging mode = line, scan area = 1.3X.

#### Lipid droplet size measurements and image colorization

Lipid droplet sizes were measured from confocal micrographs using a macro developed in Fiji/ImageJ version 2.3.0 (www.fiji.sc) using the Macro Recorder tool. A color threshold of 62 (out of 255) was applied to generate a mask over the stained areas. This mask was made binary and was segmented using the watershed function. The particle analysis function was used to measure sizes of individual lipid droplets. A lower size limit (HepG2 WT: 100 pixels or 0.084 μm^2^; CHO WT and 2E2: 50 pixels or 0.042 μm^2^) was applied to limit detection of nonspecific or poorly defined signal. These lower limits are in a similar range to those used by other groups when measuring lipid droplet size (69). Lipid droplet sizes and areas were used to generate lipid droplet size frequency distributions, and calculate median lipid droplet size, lipid droplet number, and total lipid droplet area. Lipid droplet number and total lipid droplet area were normalized to total number of nuclei, which were determined by counting in Fiji/ImageJ.

For visualization of lipid droplet sizes, segmented Oil Red O channel images were artificially colorized with a Fiji/ImageJ macro that colorizes each droplet based on its corresponding size bin using a for loop.

### Preparation of whole-cell lysates for pAKT measurement

CHO WT and 2E2 cells were seeded in 6-well plates at densities that would achieve subconfluence (85–95%) 24 h later (WT: 5.5 × 10^5^, 2E2: 6.2 × 10^5^). Cell monolayers were washed twice with cold PBS on ice and scraped in 200 μl RIPA, followed by a 20 min incubation on ice. Lysates were collected into microfuge tubes and incubated for a further 10 min on ice, vortexing for 10 s up to three times throughout the incubation. Whole-cell lysates were sonicated using a Sonicator S-4000 (Misonix) (amplitude = 30, 5 × 1-s pulses) once, centrifuged at 12,000*g* for 10 min at 4 °C, and supernatants were collected and stored at −80 °C prior to protein measurement and immunoblotting.

### RNA-seq and differential expression analysis

RNA was isolated from CHO WT and 2E2 cells using the RNeasy Mini Kit (QIAGEN 74104). All samples were sequenced at the London Regional Genomics Centre (Robarts Research Institute; http://www.lrgc.ca) using an Illumina NextSeq 500 (Illumina Inc).

Total RNA samples were quantified by NanoDrop (Thermo Fisher Scientific), and quality was assessed using an Agilent 2100 Bioanalyzer (Agilent Technologies Inc) and the RNA 6000 Nano kit (Caliper Life Sciences). Samples were then processed using the Vazyme VAHTS Total RNA-seq (H/M/R) Library Prep Kit for Illumina (Vazyme), which includes rRNA reduction. Briefly, samples were rRNA depleted, fragmented, and complementary DNA (cDNA) was synthesized, indexed, cleaned, and amplified *via* PCR. Libraries were then equimolar pooled into one library and size distribution was assessed using an Agilent High Sensitivity DNA Bioanalyzer chip and quantitated using the Qubit 2.0 Fluorimeter (Thermo Fisher Scientific).

The library was sequenced using an Illumina NextSeq 500 as 76 bp single end runs, with one High Output v2 kit (75 cycles). Fastq data files were analyzed using Partek Flow. After importation, data was aligned to the *Cricetulus griseus* CHOK1GS genome using STAR 2.7.3a and annotated using NCBI *C. griseus* v104. Genes with less than ten reads across all samples were removed, and differential expression analysis was performed using the *DESeq2* package in R version 4.2.2 (70) following the corresponding vignette on Bioconductor (http://bioconductor.org/packages/devel/bioc/vignettes/DESeq2/inst/doc/DESeq2.html) (log fold change shrinkage using *apeglm* method (71); count data transformation using variance stabilizing transformation, or *vst*). Unfiltered and filtered (fold change > 1.5, q value < 0.05) gene lists were generated. Principal component analysis was performed using the plotPCA function in *DESeq2*. A volcano plot of differentially expressed genes in CHO 2E2 cells relative to CHO WT cells was generated using the *EnhancedVolcano* package in R (Blighe, *Rana*, and Lewis, 2018), following the corresponding vignette on Bioconductor (https://bioconductor.org/packages/devel/bioc/vignettes/EnhancedVolcano/inst/doc/EnhancedVolcano.html).

### Gene set enrichment analysis

Gene set enrichment analysis (GSEA) (Broad Institute, v4.3.2) (72) was performed on *DESeq2* output following a modification of a previously described method (73). The unfiltered gene list was ranked using the *DESeq2* Wald statistic (stat column), as has been done by other groups (74). GSEA was performed using the ranked gene list and the Human_GOBP_AllPathways_no_GO_iea_April_02_2023_symbol.gmt gene set file (http://download.baderlab.org/EM_Genesets/). Human gene sets were used as has been done for other GSEA in CHO cells (75), because *C. griseus* gene sets are not yet available. Gene sets containing greater than 200 genes and less than 15 genes were excluded, as inclusion of both large and small gene sets can complicate results interpretation (73). Gene set alterations were considered statistically significant at a q value <0.05. A bubble plot of significantly altered gene sets was generated using *ggplot2* in R. Similar gene sets were grouped into biological themes, and biological themes were arranged from top to bottom in the bubble plot in decreasing order based on the highest absolute normalized enrichment score within a biological theme. Two gene sets, “TNFA signaling *via* NFKB” and “MYC targets V1” had a q value of 0. To plot these gene sets on the bubble plot, the zero q values were replaced with the lowest nonzero q value multiplied by 10^−1^, which is a method used by the *EnhancedVolcano* R package to handle *p* values of 0.

### Preparation of nuclear and cytoplasmic fractions

Nuclear and cytoplasmic fractions were prepared by hypotonic lysis following a modification of a previously described method (76). CHO WT and 2E2 cells were seeded in 10 cm dishes at densities to achieve subconfluence (85–95%) 24 h later (WT: 3.7 × 10^6^, 2E2: 4.4 × 10^6^). For each cell line, two 10 cm dishes were washed twice with cold PBS, scraped, and pooled into 1 ml cold PBS. Cells were pelleted at 1650*g* for 3 min at 4 °C, and pellets were resuspended in 900 μl hypotonic lysis buffer (20 mM Hepes, 1 mM EGTA, 1 mM EDTA, 1 mM DTT, 0.3% NP-40, pH 7.4) using a 1 ml syringe and 27G needle. Cells were incubated on ice for 30 min, vortexing for 10 s, at approximately 10 min intervals during the 30 min incubation. Lysates were centrifuged at maximum speed at 4 °C for 3 min. Eight hundred twenty microliters supernatant was collected as the cytoplasmic fraction, and any remaining supernatant was discarded. Nuclear pellets were washed by vortexing for 20 s at maximum speed in 1 ml of wash buffer (10 mM Hepes, 10 mM KCl, 0.1 mM EGTA, 0.1 mM EDTA, pH 7.4), followed by centrifugation at maximum speed at 4 °C for 1 min. The supernatant wash was then discarded. This process was repeated for a total of two washes. Remaining nuclear pellets were resuspended in 90 μl RIPA buffer and were lysed by incubation on ice for 20 min, vortexing for 10 s at approximately 5 min intervals throughout the 20 min incubation. Samples were centrifuged at maximum speed at 4 °C for 10 min, and the supernatant was collected as the nuclear fraction. Cytoplasmic and nuclear fractions were clarified by a final centrifugation at maximum speed for 20 min at 4 °C, and supernatants were collected and stored at −80 °C until use. Whole-cell lysates were prepared in parallel from one 10 cm dish using 500 μl RIPA as described in “Preparation of whole-cell lysates for pAKT measurement.”

### Immunoblotting

All protein samples used for immunoblotting (subcellular fractions and whole-cell lysates) were prepared in buffer supplemented with a protease inhibitor cocktail (Roche cOmplete, Mini Protease Inhibitor Cocktail 11836153001) and phosphatase inhibitor cocktails 2 (Abcam ab201113; Sigma P5726) and 3 (Abcam ab201114; Sigma P0044) following supplier’s instructions. Protein concentrations were determined by Bradford assay (Bio-Rad 5000002) and samples (whole-cell lysates: 20 μg; nuclear and cytosolic fractions: 10 μg) were resolved by 10% SDS-PAGE (Bio-Rad 4561035 or 4568035; Thermo Fisher Scientific XP00105BOX) and electroblotted onto polyvinylidene difluoride membrane by semidry transfer (HEP-1 Semidry Electroblotter, Thermo Fisher Scientific). Membranes were blocked with 3% nonfat dry milk in Tris-buffered saline (20 mM Tris, 150 mM NaCl, pH 7.6) containing 0.1% Tween 20 (TBST) (for GAPDH), 5% milk in TBST (for RELA, RELB, LAMINA/C, HK2) or 5% BSA in TBST (for pAKT and AKT). The following primary antibodies were used: GAPDH (from ProteoExtract Cytoskeleton Enrichment and Isolation Kit, Sigma 17-10195, 1:5000), phospho-Akt (Ser473) (Cell Signaling Technology #9271, 1:1000), Akt (Cell Signaling Technology #2920, 1:2000), NF-κB p65/RelA (Cell Signaling Technology #8242, 1:1000), RelB (Cell Signaling Technology #4922, 1:2000), c-Myc (Cell Signaling Technology #5605, 1:1000), Hexokinase II (Cell Signaling Technology #2867, 1:1000). Membranes were probed overnight at 4 °C with primary antibody and subsequently with the appropriate secondary antibodies (goat anti-rabbit HRP, Abcam ab97080; goat anti-mouse HRP, Abcam ab97040; 1:10,000). All antibodies were diluted in 5% BSA in TBST except for the GAPDH antibody, which was diluted in 3% milk in TBST. Bands were detected using Immobilon Forte Western HRP substrate (Sigma WBLUF0100) and immunoblots were imaged using a ChemiDoc (Bio-Rad). Following target protein detection, membranes were stained for total protein using PageBlue Protein Staining Solution (Thermo Fisher Scientific 24620), following a modification of the supplier’s instructions. Dried polyvinylidene difluoride membranes were stained with PageBlue Protein Staining Solution for 10 min with agitation, washed with 30% ethanol (3 × 5 min) and then with distilled deionized water (3 × 5 min), and dried completely prior to imaging on a ChemiDoc using the Colorimetric setting. Target protein band and total protein lane intensities were quantified by densitometry in ImageLab version 6.1 (Bio-Rad), and target protein intensities were normalized against total protein lane intensities.

### Immunocytochemistry/immunofluorescence for RELA, RELB, and MYC

CHO WT and 2E2 cells were seeded on 22 mm square #1 glass cover slips in 6-well plates at densities of approx. 1.6 × 10^4^ and 2.1 × 10^4^ cells/cm^−2^, respectively, each in a total volume of 2 ml/well. After 48 h, cells were washed twice with PBS. All subsequent washes were performed with either PBS or wash buffer (0.1% Triton X-100 in PBS containing 0.2% BSA), as indicated, using 3 × 10 min washes with mild agitation. Cells were fixed in 4% methanol-free formaldehyde, pH 8 (Thermo Fisher Scientific 28906) for 15 min, followed by PBS washes. Cells were permeabilized with 0.1% Triton X-100 in PBS (permeabilization buffer) for 10 min. Coverslips were blocked for 1 h using 10% normal goat serum blocking solution (Thermo Fisher Scientific 50062Z). Primary antibodies (NF-κB p65/RelA and c-Myc as described in “immunoblotting” with the following dilutions: 1:400; RelB, Cell Signaling Technology #10544, 1:800; α-tubulin, Sigma-Aldrich T5168, 1:1000) were diluted in permeabilization buffer containing 1% BSA (antibody incubation buffer) and coverslips were incubated in primary antibody overnight at 4 °C in a humidified chamber. The next day, coverslips were washed with wash buffer, briefly rinsed with PBS, and incubated with secondary antibodies (goat anti-rabbit Alexa Fluor 488 Plus, Thermo Fisher Scientific #A48282, 1:1000; goat anti-mouse Alexa Fluor 647 Plus, Thermo Fisher Scientific #A48289, 1:1000) diluted in antibody incubation buffer for 2 h. Coverslips were washed with wash buffer and incubated with 200 ng/ml DAPI in wash buffer for 10 min and washed again with wash buffer. Prior to mounting coverslips on microscope slides (Thermo Fisher Scientific 12-550-15), coverslips were dipped in PBS, then dipped sequentially into two separate beakers of distilled deionized water to eliminate residual salts. Slides were mounted using ProLong Glass antifade mountant and allowed to cure for 72 h before imaging. Images were acquired using a Leica DM6 upright microscope and Leica STELLARIS 5 confocal platform (63x/1.40 oil objectives).

### Hexokinase activity assays

Hexokinase activity was measured in fresh samples using the Hexokinase Assay Kit (Abcam ab136957), following the supplier’s instructions. CHO WT and 2E2 cells were seeded in duplicate in 6-well plates at densities that would achieve subconfluence (85–95%) 24 h later (WT: 5.5 × 10^5^, 2E2: 6.2 × 10^5^). At the time of harvest, cells were washed with cold PBS on ice and harvested in an ice-cold assay buffer by scraping. Lysates were centrifuged for 5 min at 12,000*g* at 4 °C, and supernatants were collected. Total cellular protein was measured in supernatants by BCA assay and was used for normalization. Samples were diluted appropriately to achieve optimal linearity of NADH production over the incubation period (WT 1/5; 2E2 1/2). NADH production was measured at 450 nm on kinetic mode every 5 min for 60 min. NADH production was most linear between 25 and 60 min, so hexokinase activity was calculated between these two time points. Background NADH in the samples was substantial, so background subtraction was performed for these experiments.

### Statistical analyses

Unless otherwise indicated, all statistical analyses were performed in GraphPad Prism 9.5.1. Most comparisons were performed using unpaired *t* tests, except for Figure 2, I and J and Figs. S2, *B* and *C*, S3*B*, S4, *B*–*E*, which were compared by two-way ANOVA, and Figure 2*K* and Fig. S6*C*, which were compared by one-way ANOVA, followed by post hoc analysis using Tukey tests or Šídák test (in the case of Fig. S4, *D* and *E*). Statistical analyses were performed on biological replicates. The number of biological replicates is indicated by n. Each biological replicate consisted of a number of technical replicates, which varied depending on the assay performed and is indicated in the corresponding methods section for each assay.

## Data availability

Data are contained within the manuscript and supporting information. Unprocessed RNA-seq data are available by request from the corresponding author, Nica Borradaile (nica.borradaile@schulich.uwo.ca).

## Supporting information

This article contains supporting information.

## Conflict of interest

The authors declare that they have no conflicts of interest with the contents of this article.

## Supporting information

Supporting Figure S1 **Media colour differences resulting from EEF1A1 deficiency, knockdown, or inhibition cannot be explained by dramatic cell number differences.***A*, cell numbers for wild type (WT) and EEF1A1-deficient (2E2) CHO-K1 cells at the indicated time points, n = 3. *B*, cell numbers for HepG2 cells expressing scrambled (Scr) or EEF1A1 (EF) shRNA, n = 4. *C*, cell numbers for CHO-K1 WT cells treated with DMSO or didemnin B (DB) (20 nM) for 24 h, n = 3. *D* and *E*, cell numbers for HepG2 WT cells treated with DMSO and (*D*) DB (80 nM) or (*E*) plitidepsin (PL) (80 nM) for 24 h, n = 3. Data are means ± SD. ∗*p* < 0.05.

Supporting Figure S2 **EEF1A1-deficient CHO-K1 cells are less dependent on glycolysis to support scratch closure.***A*, representative scratch closure images from wild type (WT) and EEF1A1-deficient (2E2) CHO-K1 cells upon treatment with oligomycin (Olig) or 2-deoxyglucose (2-DG) at the indicated time points. Scale bar = 500 μm. *B* and C, percent closure of scratches in WT and 2E2 cell monolayers after 8, 16, 20, 24 and 28 h treatment with (*B*) Olig-containing medium or (*C*) 2-DG-containing medium, n = 3. Data are means ± SD. Values with different letters are significantly different at *p* < 0.05.

Supporting Figure S3 **EEF1A1-deficient CHO-K1 cells are less dependent on glycolysis to support cell migration.***A*, representative images of migrated wild type (WT) and EEF1A1-deficient (2E2) CHO-K1 cells stained with crystal violet after treatment with oligomycin (Olig) or 2-deoxyglucose (2-DG) for 24 h. Scale bar = 500 μm. *B*, number of migrated WT and 2E2 cells in response to metabolic inhibitors quantified from the absorbance of crystal violet extract using cell standard curves in *C* and *D*, n = 3. Data are means ± SD. ∗∗*p* < 0.01. *C*, WT and (*D*) 2E2 cell standard curves plotting crystal violet extract absorbance against corresponding cell number.

Supporting Figure S4 **EEF1A1-deficient CHO-K1 cells maintain scratch closure in response to palmitate.***A*, representative scratch closure images from wild type (WT) and EEF1A1-deficient (2E2) CHO-K1 cells upon treatment with BSA alone (BSA), 0.1 mM palmitate (PA), or PA with etomoxir (Et) at the indicated time points. Scale bar = 500 μm. *B* and *C*, percent closure of scratches in WT and 2E2 cell monolayers after 8, 16, 20, 24, 28 h treatment with (*B*) medium containing BSA with or without Et and (*C*) medium containing PA with or without Et. Values with different letters are significantly different at *p* < 0.05. *D* and *E*, Areas under scratch closure curves from scratch assays performed over 28 h with (*D*) WT cells and (*E*) 2E2 cells treated as in *B* and *C*, n = 4. Data are means ± SD. ∗∗∗*p* < 0.001.

Supporting Figure S5 **EEF1A1-deficient CHO-K1 cells exhibit increased AKT phosphorylation at Ser473, and no substantial differences in cytoplasmic RELA and RELB, and whole-cell RELA, RELB, and MYC.***A*, representative immunoblots of whole-cell lysates (probed for phospho-AKT (pAKT) (Ser473) and total AKT) prepared from wild type (WT) and EEF1A1-deficient (2E2) CHO-K1 cells. *B*, densitometric analysis of pAKT (S473) / total AKT (tAKT) in WT and 2E2 whole-cell lysates, n = 4. *C*, densitometric analysis of cytoplasmic RELA and RELB in wild type (WT) and EEF1A1-deficient CHO-K1 (2E2) cells from immunoblots in Figure 6*A*, n = 4. *D*, representative immunoblots of whole-cell lysates (probed for RELA, RELB, and MYC) prepared from WT and 2E2 cells. *E*, densitometric analysis of whole-cell RELA, RELB, and MYC in WT and 2E2 whole-cell lysates, n = 3. Data are means ± SD. ∗*p* < 0.05. Molecular weight markers in kilodaltons indicated on the *left side* of each membrane image.

Supporting Figure S6 **Effects of EEF1A1 deficiency, knockdown, and/or inhibition on cell attachment and cell proliferation after seeding, media colour, and protein-to-cell ratios.***A*, percent of wild type (WT) and EEF1A1-deficient CHO-K1 (2E2) cells attached to culture surface at 1 h and 3 h after seeding, as determined by harvesting and counting floating and attached cell populations using a haemocytometer, n = 5. *B*, population doublings in WT and 2E2 cells at 24, 48, and 72 h after seeding, as determined by harvesting and counting cells using a haemocytometer, n = 5–6. *C*, change in 415/560 (Δ415/560) in conditioned media harvested from wild type CHO-K1 cells treated with DMSO or didemnin B (DB) at various doses, normalized to total cell number, n = 3. Protein-cell ratios determined in (*D*) wild type (WT) and EEF1A1-deficient CHO-K1 (2E2) cells (n = 8), (*E*) HepG2 cells expressing scrambled (Scr) and EEF1A1 (EF) shRNA (n = 4), (*F*) CHO-K1 WT cells treated with DB (20 nM) (n = 3), and HepG2 WT cells treated with (*G*) DB (80 nM) or (*H*) plitidepsin (PL) (80 nM) (n = 3). Data are means ± SD. ∗*p* < 0.05, ∗∗*p* < 0.01, ∗∗∗*p* < 0.001.

Supporting Figure S7 **Uncropped chemiluminescent membrane images for western blots.***A*–*E*, uncropped chemiluminescent membrane images for western blots in Figure 6*A* for (*A*) RELA, (*B*) RELB, (*C*) MYC, (*D*) GAPDH, and (*E*) LAMINA/C. *F*, uncropped chemiluminescent membrane images for HK2 western blot in Figure 6*F*. *G* and *H*, uncropped chemiluminescent membrane images for western blots in Fig. S5*A* for (*G*) pAKT (Ser473) and (*H*) total AKT. *I*–*K*, uncropped chemiluminescent membrane images for western blots in Fig. S5*D* for (*I*) RELA, (*J*) RELB, and (*K*) MYC. Molecular weight markers in kilodaltons indicated on the *left side* of each membrane image.
