# Supplementary figures and images for: Elongation factor 1A1 regulates metabolic substrate preference in mammalian cells
Source: J Biol Chem. 2024 Jan 23;300(3):105684. doi: 10.1016/j.jbc.2024.105684 (PMC10891338; doi:10.1016/j.jbc.2024.105684)

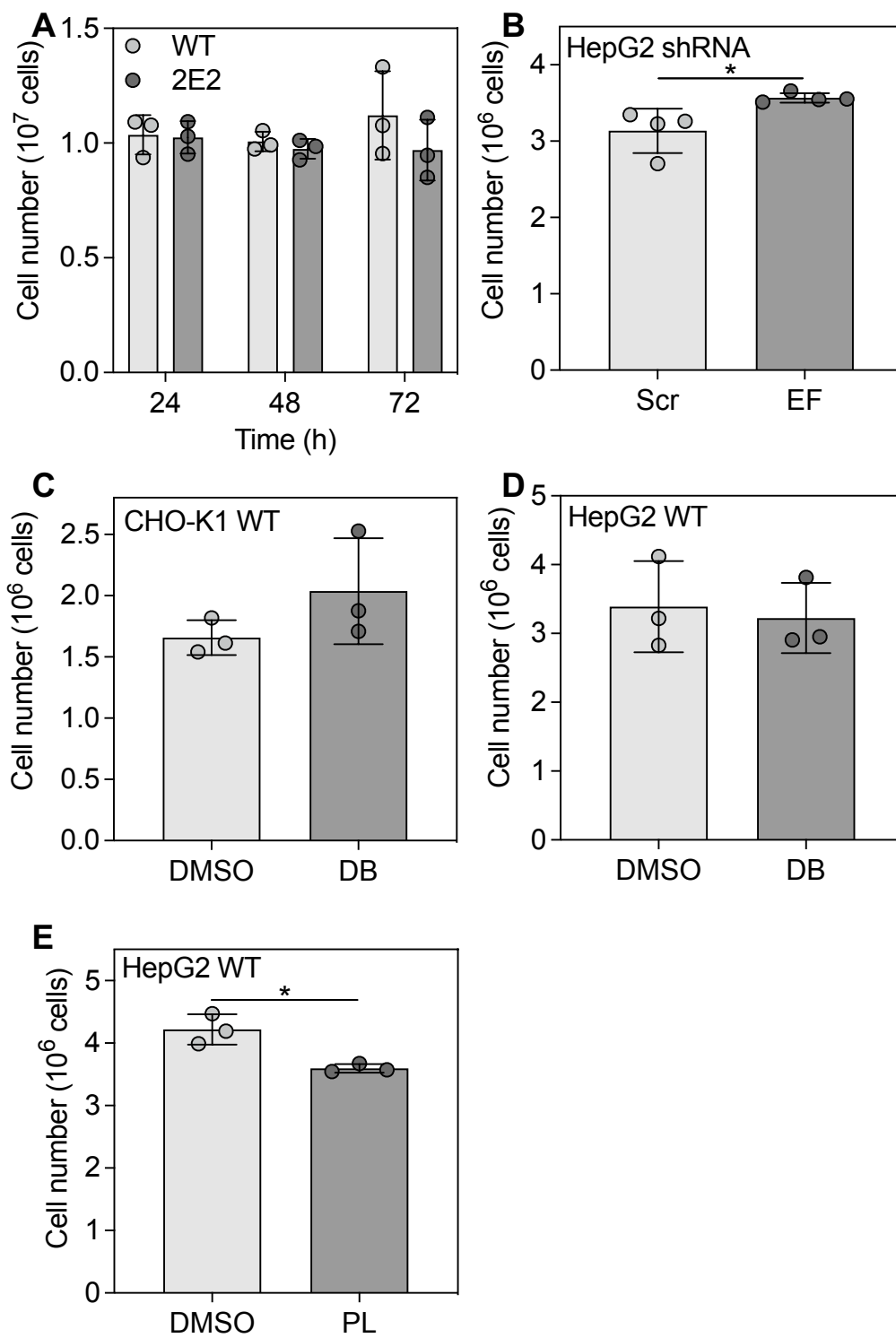

Supplement: Supporting Figure S1 — Media colour differences resulting from EEF1A1 deficiency, knockdown, or inhibition cannot be explained by dramatic cell number differences.A, cell numbers for wild type (WT) and EEF1A1-deficient (2E2) CHO-K1 cells at the indicated time points, n = 3. B, cell numbers for HepG2 cells expressing scrambled (Scr) or EEF1A1 (EF) shRNA, n = 4. C, cell numbers for CHO-K1 WT cells treated with DMSO or didemnin B (DB) (20 nM) for 24 h, n = 3. D and E, cell numbers for HepG2 WT cells treated with DMSO and (D) DB (80 nM) or (E) plitidepsin (PL) (80 nM) for 24 h, n = 3. Data are means ± SD. ∗p < 0.05. [file mmc1.pdf]

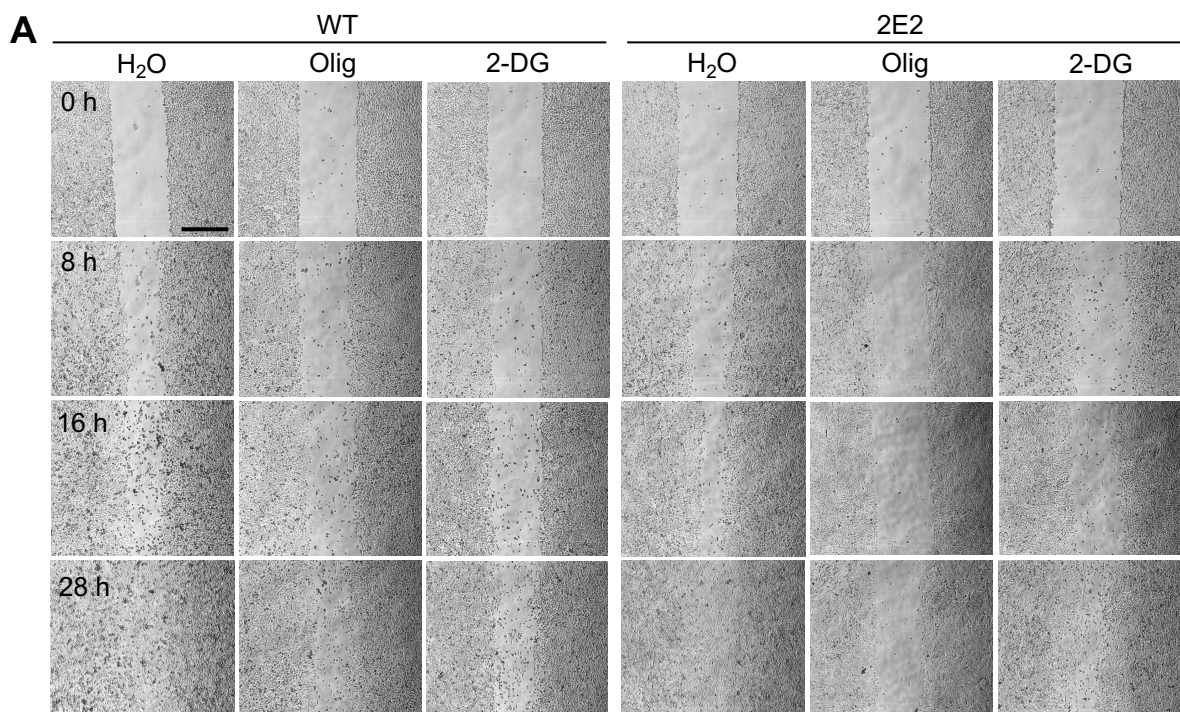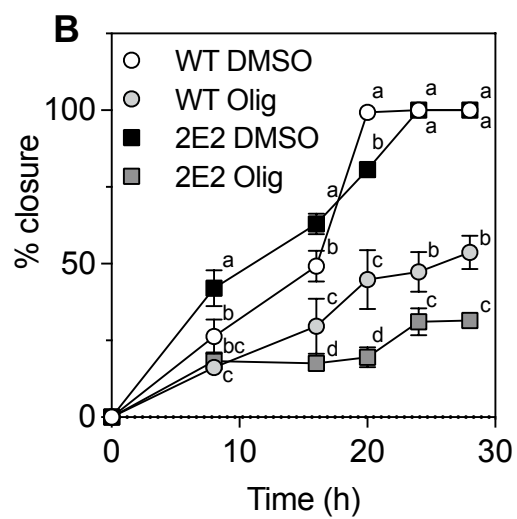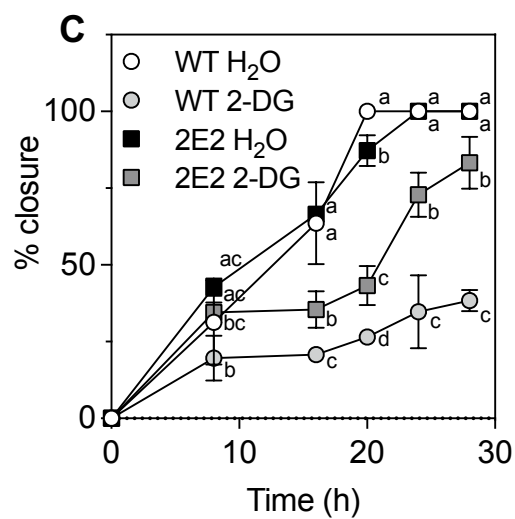

Supplement: Supporting Figure S2 — EEF1A1-deficient CHO-K1 cells are less dependent on glycolysis to support scratch closure.A, representative scratch closure images from wild type (WT) and EEF1A1-deficient (2E2) CHO-K1 cells upon treatment with oligomycin (Olig) or 2-deoxyglucose (2-DG) at the indicated time points. Scale bar = 500 μm. B and C, percent closure of scratches in WT and 2E2 cell monolayers after 8, 16, 20, 24 and 28 h treatment with (B) Olig-containing medium or (C) 2-DG-containing medium, n = 3. Data are means ± SD. Values with different letters are significantly different at p < 0.05. [file mmc2.pdf]

**A**

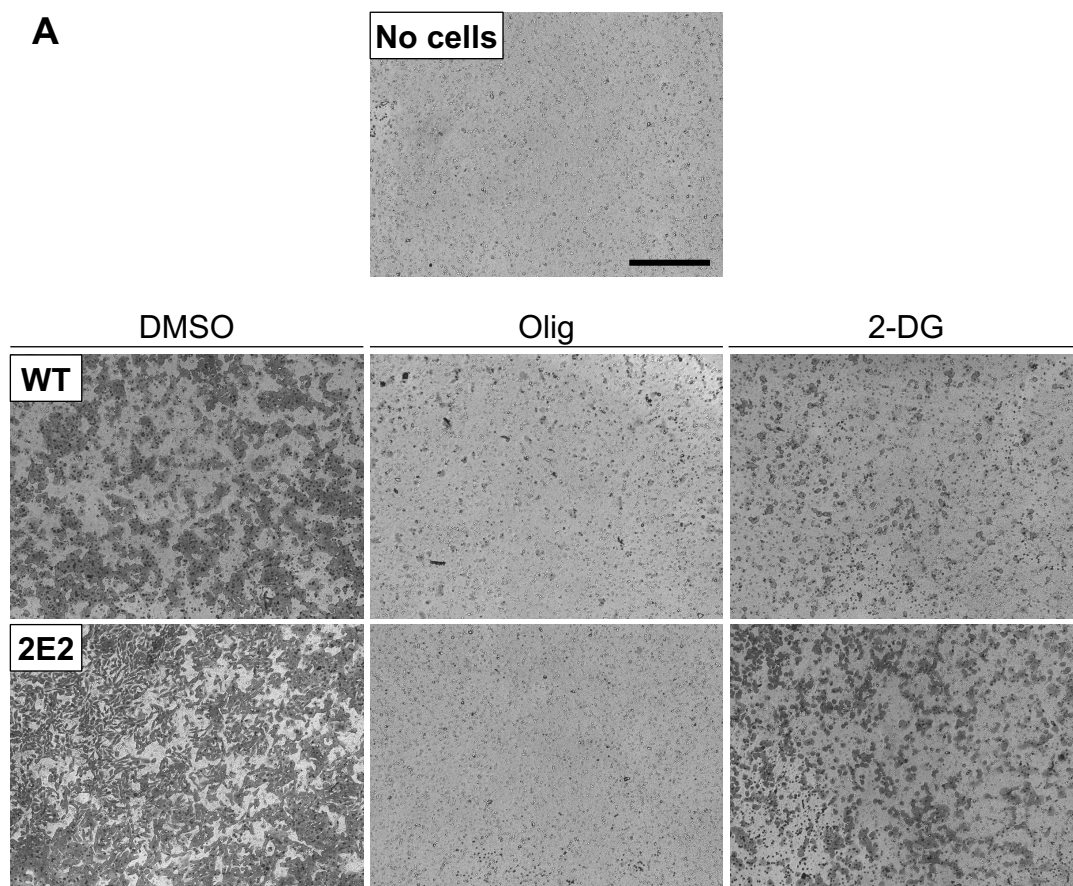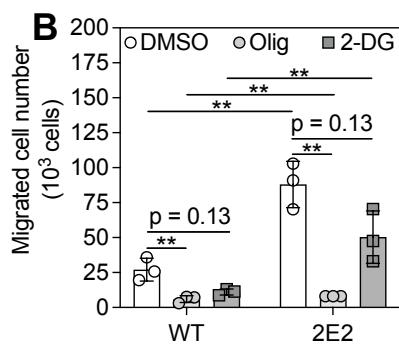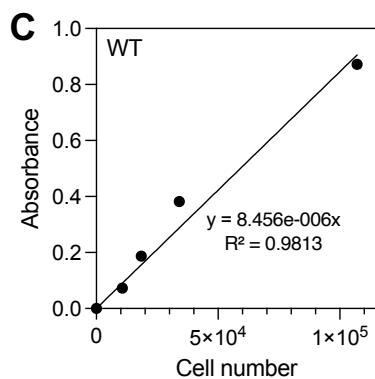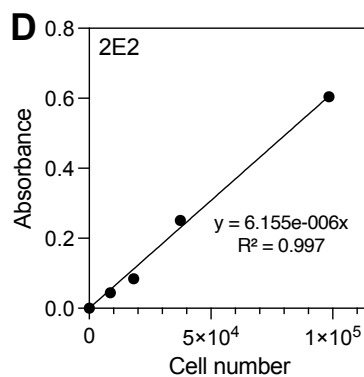

Supplement: Supporting Figure S3 — EEF1A1-deficient CHO-K1 cells are less dependent on glycolysis to support cell migration.A, representative images of migrated wild type (WT) and EEF1A1-deficient (2E2) CHO-K1 cells stained with crystal violet after treatment with oligomycin (Olig) or 2-deoxyglucose (2-DG) for 24 h. Scale bar = 500 μm. B, number of migrated WT and 2E2 cells in response to metabolic inhibitors quantified from the absorbance of crystal violet extract using cell standard curves in C and D, n = 3. Data are means ± SD. ∗∗p < 0.01. C, WT and (D) 2E2 cell standard curves plotting crystal violet extract absorbance against corresponding cell number. [file mmc3.pdf]

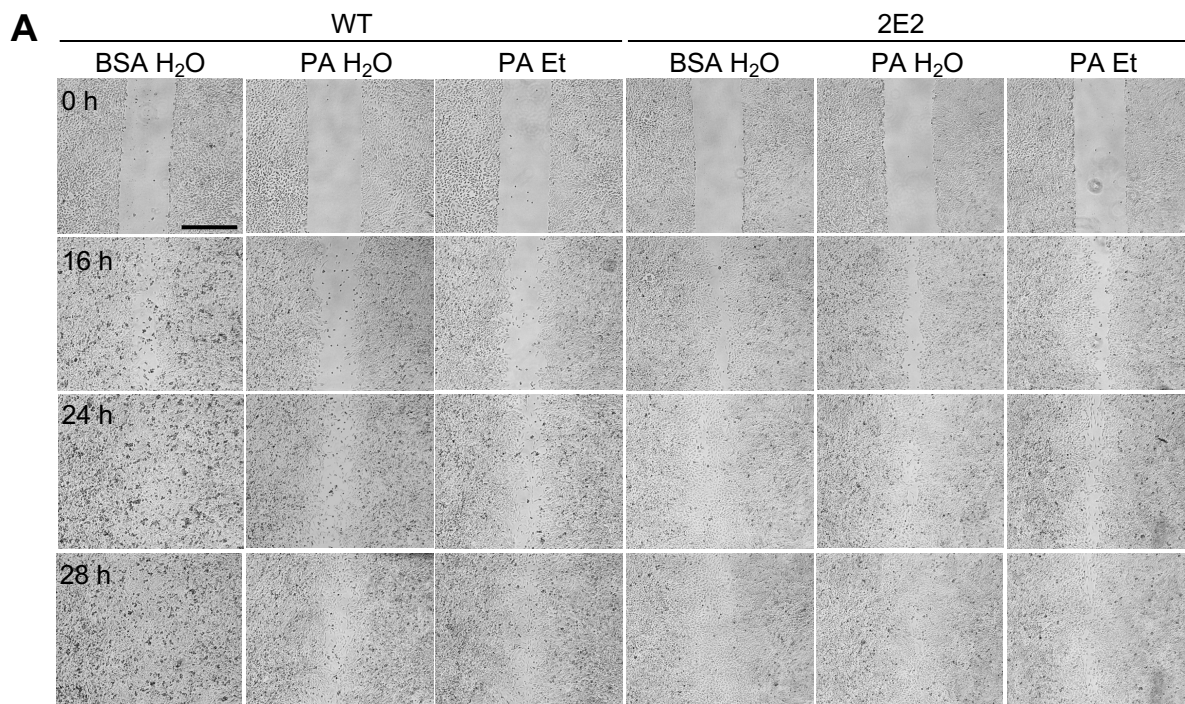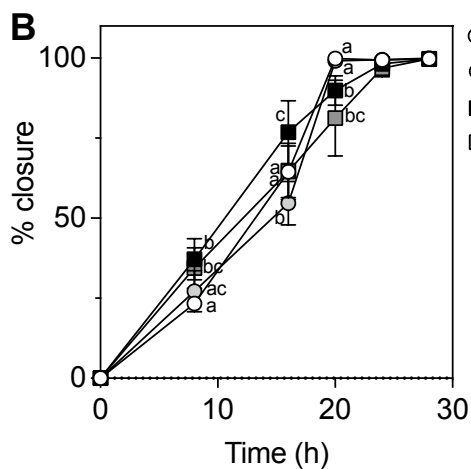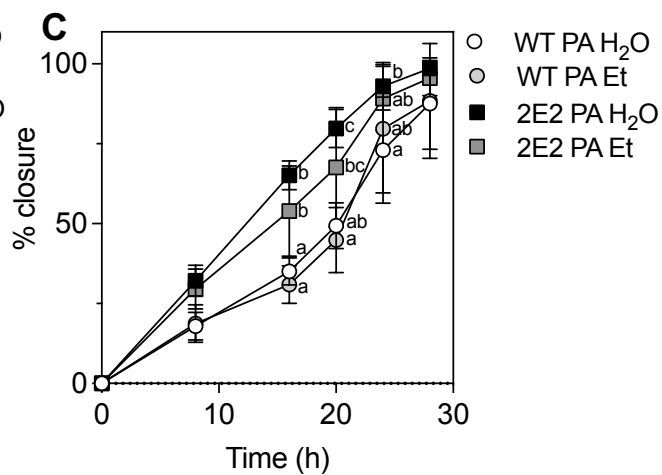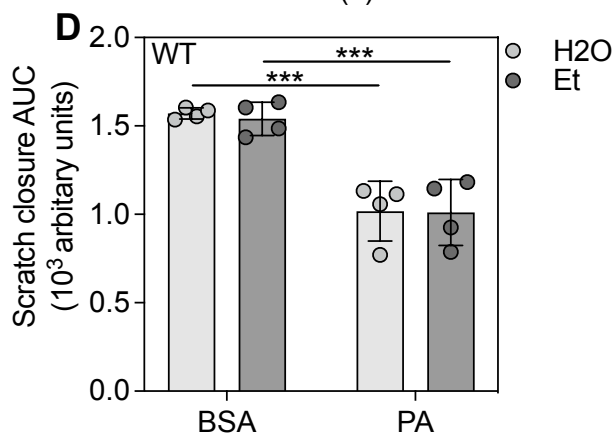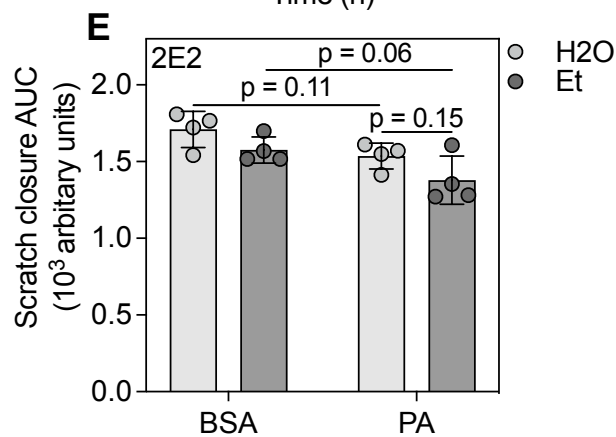

Supplement: Supporting Figure S4 — EEF1A1-deficient CHO-K1 cells maintain scratch closure in response to palmitate.A, representative scratch closure images from wild type (WT) and EEF1A1-deficient (2E2) CHO-K1 cells upon treatment with BSA alone (BSA), 0.1 mM palmitate (PA), or PA with etomoxir (Et) at the indicated time points. Scale bar = 500 μm. B and C, percent closure of scratches in WT and 2E2 cell monolayers after 8, 16, 20, 24, 28 h treatment with (B) medium containing BSA with or without Et and (C) medium containing PA with or without Et. Values with different letters are significantly different at p < 0.05. D and E, Areas under scratch closure curves from scratch assays performed over 28 h with (D) WT cells and (E) 2E2 cells treated as in B and C, n = 4. Data are means ± SD. ∗∗∗p < 0.001. [file mmc4.pdf]

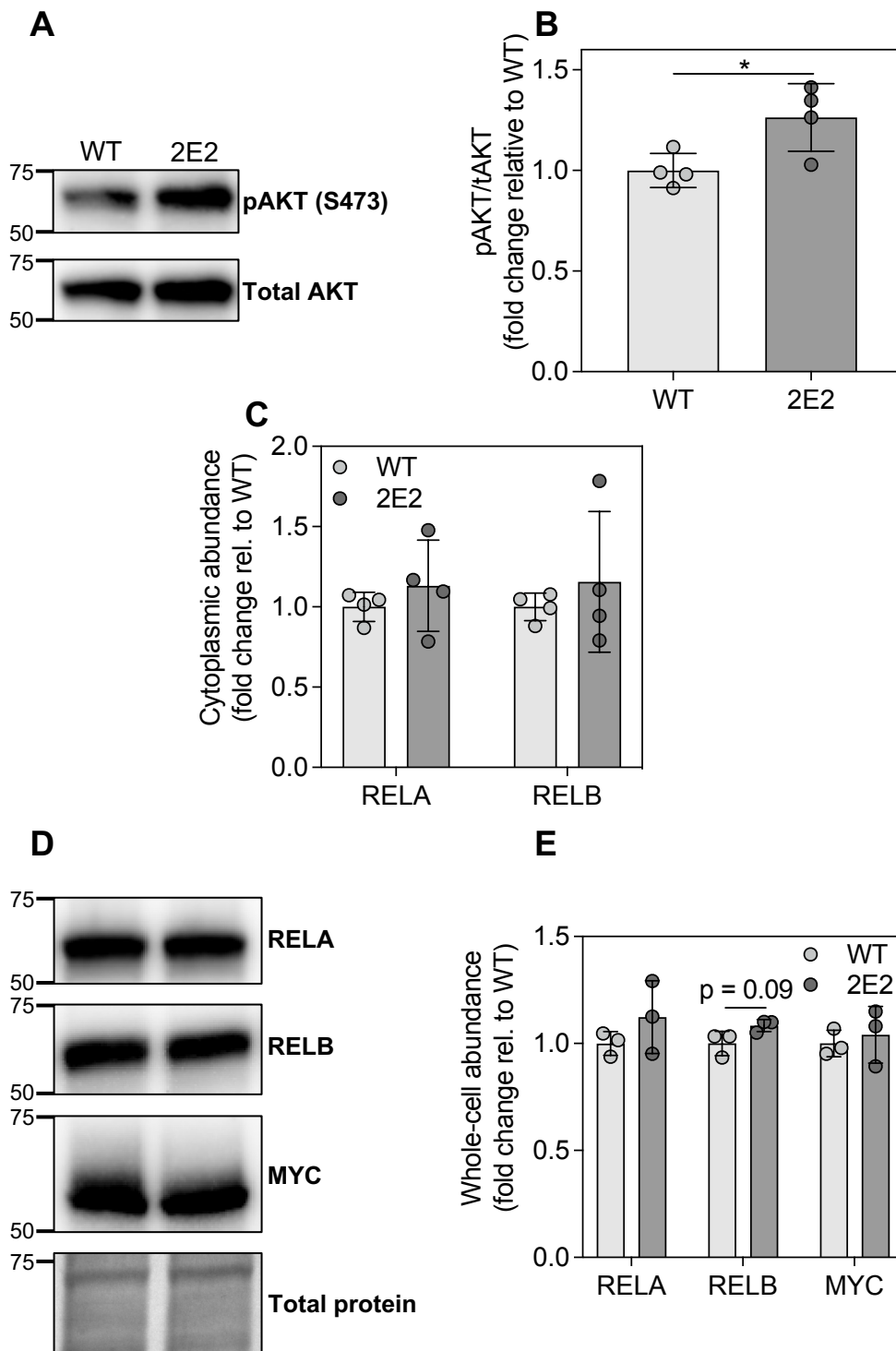

Supplement: Supporting Figure S5 — EEF1A1-deficient CHO-K1 cells exhibit increased AKT phosphorylation at Ser473, and no substantial differences in cytoplasmic RELA and RELB, and whole-cell RELA, RELB, and MYC.A, representative immunoblots of whole-cell lysates (probed for phospho-AKT (pAKT) (Ser473) and total AKT) prepared from wild type (WT) and EEF1A1-deficient (2E2) CHO-K1 cells. B, densitometric analysis of pAKT (S473) / total AKT (tAKT) in WT and 2E2 whole-cell lysates, n = 4. C, densitometric analysis of cytoplasmic RELA and RELB in wild type (WT) and EEF1A1-deficient CHO-K1 (2E2) cells from immunoblots in Figure 6A, n = 4. D, representative immunoblots of whole-cell lysates (probed for RELA, RELB, and MYC) prepared from WT and 2E2 cells. E, densitometric analysis of whole-cell RELA, RELB, and MYC in WT and 2E2 whole-cell lysates, n = 3. Data are means ± SD. ∗p < 0.05. Molecular weight markers in kilodaltons indicated on the left side of each membrane image. [file mmc5.pdf]

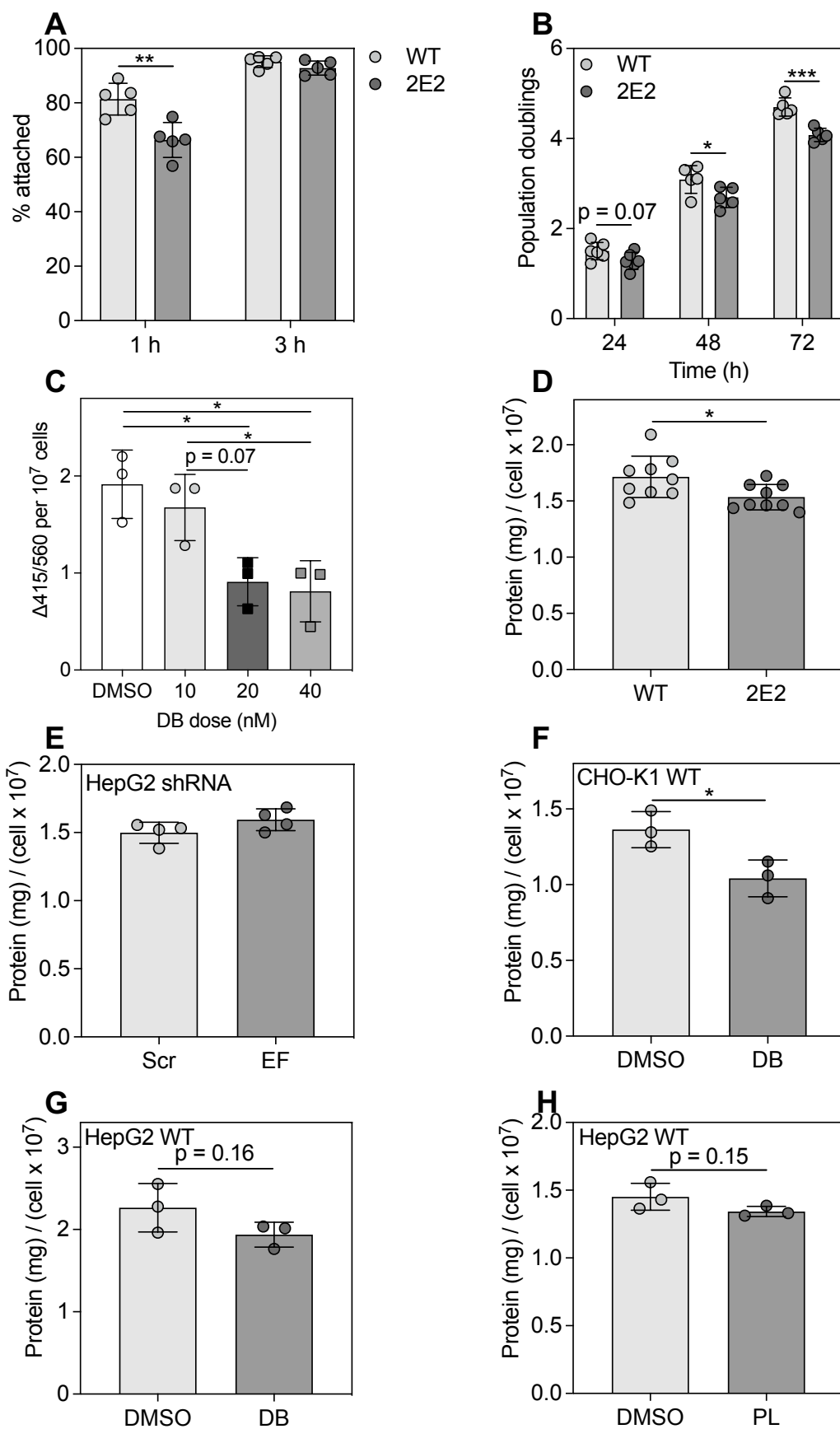

Supplement: Supporting Figure S6 — Effects of EEF1A1 deficiency, knockdown, and/or inhibition on cell attachment and cell proliferation after seeding, media colour, and protein-to-cell ratios.A, percent of wild type (WT) and EEF1A1-deficient CHO-K1 (2E2) cells attached to culture surface at 1 h and 3 h after seeding, as determined by harvesting and counting floating and attached cell populations using a haemocytometer, n = 5. B, population doublings in WT and 2E2 cells at 24, 48, and 72 h after seeding, as determined by harvesting and counting cells using a haemocytometer, n = 5–6. C, change in 415/560 (Δ415/560) in conditioned media harvested from wild type CHO-K1 cells treated with DMSO or didemnin B (DB) at various doses, normalized to total cell number, n = 3. Protein-cell ratios determined in (D) wild type (WT) and EEF1A1-deficient CHO-K1 (2E2) cells (n = 8), (E) HepG2 cells expressing scrambled (Scr) and EEF1A1 (EF) shRNA (n = 4), (F) CHO-K1 WT cells treated with DB (20 nM) (n = 3), and HepG2 WT cells treated with (G) DB (80 nM) or (H) plitidepsin (PL) (80 nM) (n = 3). Data are means ± SD. ∗p < 0.05, ∗∗p < 0.01, ∗∗∗p < 0.001. [file mmc6.pdf]

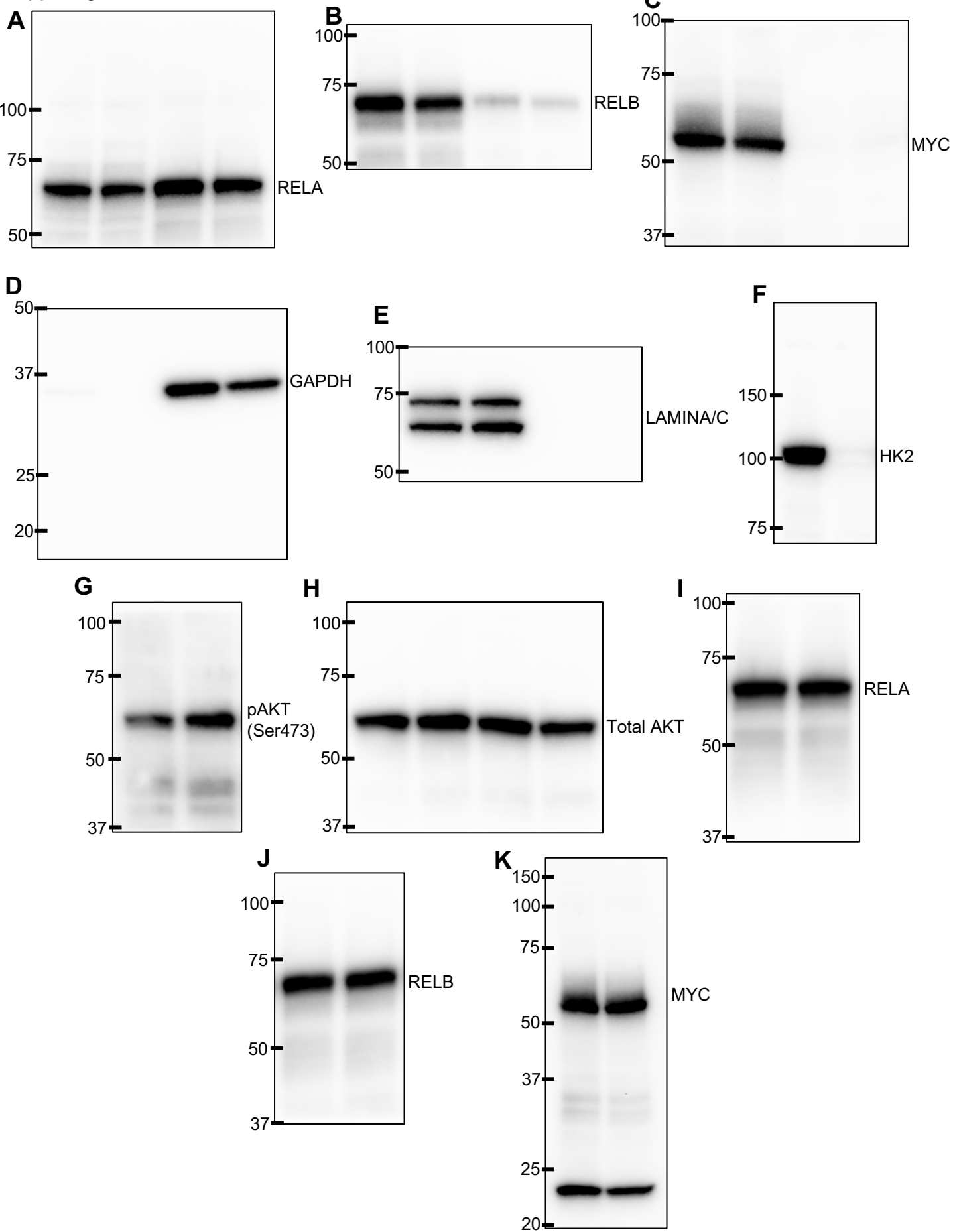

Supplement: Supporting Figure S7 — Uncropped chemiluminescent membrane images for western blots.A–E, uncropped chemiluminescent membrane images for western blots in Figure 6A for (A) RELA, (B) RELB, (C) MYC, (D) GAPDH, and (E) LAMINA/C. F, uncropped chemiluminescent membrane images for HK2 western blot in Figure 6F. G and H, uncropped chemiluminescent membrane images for western blots in Fig. S5A for (G) pAKT (Ser473) and (H) total AKT. I–K, uncropped chemiluminescent membrane images for western blots in Fig. S5D for (I) RELA, (J) RELB, and (K) MYC. Molecular weight markers in kilodaltons indicated on the left side of each membrane image. [file mmc7.pdf]
