# Supplementary material for: Elongation factor 1A1 regulates metabolic substrate preference in mammalian cells
Source: J Biol Chem. 2024 Jan 23;300(3):105684. doi: 10.1016/j.jbc.2024.105684 (PMC10891338; doi:10.1016/j.jbc.2024.105684)
Supplement: Supporting Table S1 [file mmc8.pdf]

	baseMean	FoldChange	log2FoldChar	lfcSE	pvalue	padj (q-value)
0610009B22Rik	139.537616	1.083136	0.11521441	0.16228972	0.31324112	0.51207488
0610010F05Rik	607.023293	-1.0888213	-0.1227671	0.09470302	0.14798084	0.30853427
0610010K14Rik	406.809369	1.15087173	0.20272705	0.10697892	0.03021823	0.09436397
0610012G03Rik	97.5318965	1.02796896	0.03979671	0.1769842	0.70296393	0.83109995
0610030E20Rik	17.3024827	-1.0391347	-0.0553827	0.2126878	0.41991325	0.61857046
1110004F10Rik	2051.11652	-1.0044404	-0.0063919	0.07153788	0.92381856	0.96109284
1110008P14Rik	14.7757068	1.06947708	0.09690557	0.22949578	0.20914714	0.39446038
1110012L19Rik	376.795922	-1.262403	-0.3361725	0.14340426	0.00456279	0.02059527
1110032A03Rik	37.7124728	-1.0563733	-0.0791198	0.20990202	0.3742591	0.57588697
1110038F14Rik	324.430511	-1.2165585	-0.2828057	0.11535236	0.00475376	0.02124022
1110051M20Rik	781.327526	1.12335162	0.16780958	0.08120194	0.02530195	0.08234704
1110059G10Rik	168.021521	1.06972456	0.09723938	0.15057876	0.38391302	0.58518869
1110065P20Rik	8.5086573	-1.0433958	-0.0612866	0.22210407	0.21987651	0.40720226
1500009L16Rik	277.332169	-1.2632272	-0.3371141	0.14927852	0.00569138	0.02453942
1600012H06Rik	158.3871	1.21764233	0.28409042	0.1818536	0.03220092	0.09927648
1700001P01Rik	2.11929424	-1.0030697	-0.0044219	0.21398406	0.85945383	0.92471053
1700003E16Rik	2.34660997	1.01194519	0.01713115	0.21449936	0.55429024	0.72987441
1700003F12Rik	2.66641103	-1.02756	-0.0392227	0.22016541	0.03818328	0.1134604
1700007K13Rik	41.0947534	-1.0270321	-0.0384812	0.18136626	0.70172761	0.83028433
1700008O03Rik	2.97416496	1.0105331	0.01511658	0.21401443	0.62325365	0.77883907
1700010I14Rik	5.06283947	1.01757471	0.02513472	0.2122097	0.59195503	0.75498025
1700013F07Rik	10.7875901	-1.087129	-0.1205231	0.25051544	0.09918938	0.23161064
1700014D04Rik	5.49781229	-1.0066019	-0.0094932	0.21128159	0.82268882	0.90419748
1700016H13Rik	2.03932618	-1.0167795	-0.0240068	0.21594845	0.35458026	0.55426151
1700017B05Rik	1316.48151	-1.03028	-0.0430365	0.07503516	0.53959506	0.7182266
1700025G04Rik	1916.71805	1.15898059	0.2128564	0.06436118	0.00051672	0.00334845
1700028K03Rik	6.82457804	1.04578991	0.06459306	0.2251602	0.14751244	0.30800171
1700029J07Rik	473.377706	1.32541962	0.40644918	0.12087063	0.00015227	0.00117786
1700030J22Rik	190.376629	1.28871206	0.36592996	0.17238214	0.00672112	0.02805393
1700030K09Rik	455.911046	1.10377655	0.14244815	0.10038597	0.10882394	0.24796062
1700037C18Rik	6.5534148	1.02982893	0.04240471	0.21548216	0.38938353	0.59099561
1700037H04Rik	357.532698	-1.0162529	-0.0232594	0.13438638	0.82330556	0.90419748
1700056E22Rik	15.6932977	-1.0844313	-0.1169387	0.24495421	0.12517862	0.27398566
1700066M21Rik	200.543221	1.08897029	0.12296459	0.13669606	0.25570609	0.44766913
1700067K01Rik	3.12620792	-1.0086891	-0.0124816	0.21592494	0.13125036	0.28309767
1700088E04Rik	119.577233	1.07137025	0.09945714	0.16765269	0.38219036	0.58397551
1700092M07Rik	4.67967903	1.03040472	0.04321111	0.21897564	0.22988322	0.41853384
1700094D03Rik	2.18609272	-1.0014327	-0.0020655	0.21374837	0.93741073	0.96972611
1700102P08Rik	16.4983836	-1.0486659	-0.0685551	0.21400458	0.37036276	0.571758
1700123O20Rik	102.532788	1.06040191	0.08461118	0.15800879	0.45023554	0.64578627
1810013L24Rik	1871.51544	1.01695173	0.02425121	0.07260089	0.7218474	0.84343968
1810030O07Rik	514.803551	-1.0925682	-0.1277233	0.11508187	0.19176028	0.37051699
1810037I17Rik	1384.72416	-1.0938506	-0.1294157	0.07572258	0.06712138	0.17408885
1810055G02Rik	2414.18816	-1.14979	-0.2013703	0.06586476	0.00127807	0.00710288
2010300C02Rik	3.05103619	-1.0535177	-0.0752145	0.23464377	0.00186125	0.00983134
2210016F16Rik	868.067064	1.05529505	0.07764642	0.08018968	0.29660865	0.49434775
2210016L21Rik	873.164825	-1.1491347	-0.200548	0.08110892	0.00749095	0.03074627
2210408I21Rik	375.157972	1.00888362	0.01275976	0.10560618	0.89024961	0.9416229
2300009A05Rik	74.3270838	1.05517312	0.07747972	0.18499828	0.46799017	0.66052822

2310009B15Rik	15.4734314	-1.0207503	-0.02963	0.2061573	0.65707268	0.80127899
2310011J03Rik	183.617278	-1.0560393	-0.0786635	0.13706064	0.46142576	0.65516115
2310022A10Rik	622.535449	-1.0933707	-0.1287827	0.09703483	0.13602769	0.29051596
2310022B05Rik	1074.88137	-1.0204661	-0.0292283	0.07940885	0.69105615	0.8231755
2310033P09Rik	221.131826	1.02184758	0.03118002	0.13268394	0.76700304	0.87111526
2310039H08Rik	2.05719042	1.00935074	0.01342759	0.21464127	0.58190583	0.74773936
2310057M21Rik	482.663502	-1.158571	-0.2123465	0.11228742	0.02837805	0.08955968
2310061I04Rik	1336.5377	1.19102475	0.25220339	0.08106815	0.00081345	0.00491352
2410002F23Rik	392.28837	-1.1681977	-0.2242844	0.16888446	0.06916227	0.17786965
2410004B18Rik	468.901724	-1.1300111	-0.176337	0.11340707	0.06986171	0.1789857
2410131K14Rik	225.736466	1.12719947	0.17274284	0.14231754	0.12158097	0.26801793
2510009E07Rik	1031.66348	1.20628563	0.27057156	0.08627644	0.00067811	0.00420252
2510039O18Rik	1456.92516	-1.0146469	-0.0209777	0.07786795	0.77174261	0.87309219
2610001J05Rik	204.7326	-1.0531127	-0.0746598	0.12680501	0.4667431	0.65956746
2610002M06Rik	1453.91583	-1.0666501	-0.0930869	0.07727128	0.19505423	0.37505924
2610008E11Rik	121.235974	1.09714937	0.13375995	0.15700842	0.24466846	0.43500388
2610301B20Rik	290.671173	1.02367823	0.03376231	0.10337466	0.71075814	0.83676241
2610507B11Rik	25511.5017	1.06532748	0.09129698	0.04362497	0.03262254	0.10033488
2700049A03Rik	1509.08207	-1.0888916	-0.1228603	0.08292019	0.10718247	0.24509236
2700062C07Rik	454.738348	-1.1198044	-0.1632468	0.10122673	0.06798127	0.17579687
2700081O15Rik	757.68385	-1.1026053	-0.1409164	0.08501048	0.0700686	0.17933641
2700097O09Rik	154.919853	1.02621603	0.03733447	0.1498877	0.73120821	0.84928616
2810004N23Rik	1369.78697	-1.228277	-0.296636	0.08736295	0.0002331	0.00170047
2810006K23Rik	376.715991	1.02342634	0.03340727	0.12625953	0.7462674	0.85906591
2810021J22Rik	316.353074	1.005196	0.00747684	0.1158428	0.93983657	0.97091181
2810408A11Rik	47.5248401	1.83732761	0.87760889	0.34199478	0.00058346	0.00371107
3110001I22Rik	330.800414	-1.1304759	-0.1769303	0.12088024	0.08137849	0.20047278
3110009E18Rik	48.7987014	1.0342725	0.04861634	0.19794526	0.58525782	0.74949042
3110021N24Rik	5.42924148	1.01882804	0.02691058	0.21485442	0.45262756	0.64747728
3110040N11Rik	161.101203	-1.0568776	-0.0798083	0.15084806	0.46829978	0.66067404
3110082I17Rik	1070.71857	-1.0513316	-0.0722178	0.08566065	0.35660856	0.55661699
3830417A13Rik	44.6382798	-5.7080277	-2.5129923	0.36531293	3.74E-13	1.26E-11
4430402I18Rik	46.7023344	-1.0681626	-0.0951313	0.20261564	0.35921761	0.55953035
4833420G17Rik	288.826252	1.04205717	0.05943442	0.13027772	0.56979764	0.73915016
4833439L19Rik	1957.48218	1.00597469	0.00859401	0.05678751	0.87589666	0.93330862
4921507P07Rik	474.300654	-2.1240522	-1.0868192	0.1312719	8.91E-18	4.52E-16
4921524J17Rik	770.314619	-1.0855603	-0.1184399	0.10253685	0.18594356	0.36321089
4921536K21Rik	13.3046297	-1.0511873	-0.0720197	0.21578056	0.34088706	0.54024041
4930402H24Rik	565.48218	1.39018878	0.47528081	0.12776297	2.96E-05	0.00027242
4930404N11Rik	5.29068726	-1.0035835	-0.0051606	0.21275542	0.87516827	0.93292049
4930407I10Rik	11.4851112	-1.0275195	-0.0391658	0.21068723	0.52221759	0.70431747
4930430F08Rik	798.553721	-1.0912322	-0.1259581	0.09420535	0.13584947	0.29028052
4930432K21Rik	99.8093036	-1.1036219	-0.142246	0.17758563	0.22863262	0.41744354
4930452B06Rik	1535.06098	1.14096683	0.19025685	0.06739103	0.00289072	0.01416066
4930453N24Rik	1074.01974	-1.0327694	-0.0465181	0.09467502	0.58257997	0.74815801
4930486L24Rik	904.851489	-2.4940688	-1.3185013	0.07712752	1.05E-66	5.62E-64
4930503L19Rik	457.08238	-1.1422875	-0.1919258	0.11920398	0.05687664	0.15341962
4930523C07Rik	71.4138977	-1.0915082	-0.1263229	0.19679196	0.27025969	0.46562005
4930563E22Rik	5.18181395	-1.0177396	-0.0253685	0.21445444	0.48942715	0.67718402
4930579G24Rik	1140.96076	-1.1470995	-0.1979906	0.11580207	0.04510436	0.12902788

4931406C07Rik	178.2966	1.0022814	0.00328761	0.13794882	0.97601768	0.98895979
4931406P16Rik	5.41069212	20.7352489	4.37401346	1.59711177	0.00011905	0.00095133
4931414P19Rik	69.0588665	-1.1260205	-0.1712331	0.20756028	0.16480516	0.33325242
4931428F04Rik	177.883502	1.03903111	0.05523886	0.14771128	0.6123413	0.77190436
4932438A13Rik	8159.64942	-1.0114437	-0.016416	0.04759014	0.72213268	0.84343968
4933405L10Rik	9.98933595	1.04299883	0.06073754	0.22135861	0.2440725	0.43430597
4933411K16Rik	10.7272999	1.07864148	0.10921541	0.24437805	0.109348	0.2488007
5031439G07Rik	579.160744	1.04887425	0.06884173	0.09551779	0.42127766	0.61976697
5730409E04Rik	72.8990427	1.06102878	0.08546379	0.18307313	0.4325065	0.63037983
5730455P16Rik	126.196999	1.06536544	0.09134839	0.14663637	0.40696977	0.60640721
5730480H06Rik	141.550154	1.08730288	0.12075388	0.17479828	0.29786624	0.49571879
5S_rRNA	13.7394473	1.05903051	0.08274416	0.22354022	0.25759108	0.44986254
5_8S_rRNA	369.782635	1.01039876	0.01492477	0.20204686	0.83872705	0.91371188
6030458C11Rik	458.189858	1.04333151	0.06119764	0.10436643	0.50280662	0.68781798
6030498E09Rik	206.832953	-1.3541397	-0.4373766	0.15391755	0.00071118	0.00437689
6430548M08Rik	147.023004	-1.1243521	-0.1690938	0.15581498	0.14546041	0.30503506
6430550D23Rik	1.67029039	1.00970869	0.01393912	0.21504989	0.5252353	0.70682469
6430571L13Rik	237.644315	1.30310689	0.38195543	0.13671893	0.00106545	0.00614791
7SK	221885.301	1.06266406	0.08768559	0.04874363	0.06720872	0.17420957
8030462N17Rik	223.766561	1.011995	0.01720216	0.1353879	0.87149845	0.93041135
9130008F23Rik	44.0006769	-1.4942725	-0.5794432	0.53855634	0.01124765	0.04322903
9130409I23Rik	6.06918772	1.00207883	0.002996	0.20847797	0.95656144	0.97901352
9430015G10Rik	116.73034	1.4051457	0.49071973	0.27784329	0.00729196	0.03007404
9430038I01Rik	381.122809	-1.2792378	-0.3552845	0.12093094	0.00076613	0.00466894
9530068E07Rik	4393.286	-1.0034425	-0.004958	0.04633252	0.91210209	0.95457743
9530077C05Rik	58.8080125	-1.049421	-0.0695936	0.18589955	0.50534357	0.6897657
9930012K11Rik	19.7748408	1.0657646	0.09188881	0.22514724	0.24586251	0.43597712
9930021J03Rik	2315.26488	-1.032696	-0.0464157	0.07818238	0.52299169	0.70506457
9930104L06Rik	344.080805	-1.0520372	-0.0731857	0.11780217	0.4588461	0.6530184
A430005L14Rik	383.9582	1.00807419	0.01160182	0.11691793	0.91231237	0.95462518
A530084C06Rik	3.38141846	1.00357781	0.00515248	0.21166517	0.89862773	0.94726921
A630001G21Rik	65.4673862	1.05816112	0.08155932	0.18383273	0.45086345	0.64618934
AA467197	38.87153	1.02343	0.03341243	0.19300001	0.71231854	0.83736812
AA986860	5.7801007	1.01377479	0.01973719	0.21281628	0.62850722	0.78280773
AARS	10293.2087	1.27582586	0.35143143	0.05852421	4.90E-10	1.08E-08
ABCB1	3690.90849	-1.1732831	-0.2305512	0.07339407	0.00082292	0.00495379
ADSS	5824.3666	-1.1952814	-0.2573503	0.05496158	1.28E-06	1.61E-05
ADSSL1	9.50537393	-1.0090425	-0.0129869	0.2077728	0.81734593	0.90136031
AI413582	26.4398867	1.02565814	0.03654995	0.19972793	0.65582554	0.8004869
AI429214	253.63585	-1.0595345	-0.0834306	0.11991851	0.40302395	0.60272175
AI597479	700.735428	1.03715714	0.05263449	0.08086161	0.4811058	0.67135188
AI837181	1215.36994	-1.2660017	-0.3402794	0.13196871	0.00242013	0.01226827
AIMP2	1342.1354	-1.1110503	-0.1519241	0.07159738	0.02398084	0.07925557
AK3	1150.4166	1.01377586	0.01973871	0.07069876	0.76840374	0.8713927
AKR1A1	8904.77568	1.05263065	0.07399931	0.05685294	0.17930935	0.35448155
AKR1B8	2277.58685	-1.7539795	-0.8106319	0.07569791	8.00E-28	7.32E-26
APE	4162.02744	-1.1165809	-0.1590878	0.05799026	0.00427621	0.01952183
APIP	602.417541	-1.4761821	-0.5618707	0.09400112	2.80E-10	6.46E-09
APRT	519.842276	1.24664566	0.31805145	0.1105365	0.00116606	0.00661234
ASNA1	2111.68244	1.18623269	0.24638703	0.05428022	2.65E-06	3.13E-05

ASNS	1777.11567	1.5777123	0.65783415	0.07894297	8.19E-18	4.18E-16
ATAT1	141.11548	1.15518017	0.20811788	0.17426238	0.09457141	0.22405557
ATP6	84663.546	1.21446384	0.28031954	0.21547498	0.04099652	0.11987432
ATP8	1449.07368	-1.0164627	-0.0235572	0.18263921	0.80752671	0.89434934
AU015836	2.36342829	1.00251062	0.00361751	0.21382126	0.89201789	0.94295669
AU021092	1.63787186	1.0269062	0.03830441	0.22003882	0.04101486	0.11989968
AU022252	166.265223	1.04662511	0.06574478	0.15080724	0.54956612	0.72629135
AU040320	1303.39385	1.17711903	0.23526021	0.07569454	0.00090367	0.00536673
AW209491	685.004507	-1.1607723	-0.215085	0.09893749	0.01490239	0.0542827
AW549877	595.36355	1.17697847	0.23508793	0.08859638	0.00371412	0.01742778
AW551984	480.717412	-1.0677602	-0.0945877	0.11839199	0.33733971	0.53701903
AW554918	273.252946	1.05664904	0.07949627	0.14309493	0.4662124	0.65925935
Aaas	1348.72992	1.02858832	0.04066568	0.10124884	0.65029898	0.79726959
Aacs	936.415151	1.03399686	0.04823181	0.07855259	0.51009076	0.69415393
Aadat	2.397146	-1.0130956	-0.0187704	0.21467903	0.51363611	0.69751708
Aagab	561.561326	1.01338212	0.01917828	0.10544918	0.83506659	0.91171934
Aak1	967.498323	1.16556917	0.22103462	0.07743722	0.00220753	0.01132499
Aamp	3153.75147	-1.0533706	-0.0750131	0.05573476	0.16254334	0.32987645
Aar2	793.336468	-1.0963139	-0.132661	0.10465703	0.15669743	0.32163019
Aars2	867.718646	1.0307105	0.04363917	0.09054878	0.59590009	0.75879524
Aasdh	941.892755	-1.0028557	-0.004114	0.07948512	0.95482402	0.97819064
Aatf	6223.66729	-1.0267228	-0.0380467	0.05993268	0.50793476	0.69219621
Aatk	5.07448528	1.00510961	0.00735284	0.21229959	0.84468854	0.91636689
Abca1	3434.81361	-1.0536736	-0.075428	0.05927163	0.1843365	0.36089724
Abca12	5.11343293	-1.0331134	-0.0469987	0.21940984	0.23221319	0.42139377
Abca2	1191.07017	1.05840064	0.08188584	0.08343897	0.28639652	0.48293374
Abca4	2.12649856	1.00283441	0.0040834	0.21383282	0.87974508	0.9356193
Abca5	477.565342	1.78075928	0.83249251	0.10946709	2.44E-15	1.01E-13
Abca7	240.582348	1.27614176	0.3517886	0.15432251	0.00489754	0.02173976
Abca9	90.1832591	-1.2612232	-0.3348237	0.31834513	0.0359578	0.10815202
Abcb10	546.089581	1.08930142	0.12340322	0.09706392	0.15342046	0.31632321
Abcb6	1000.00562	-1.0632307	-0.0884546	0.08093413	0.23615631	0.42583928
Abcb7	3368.43748	-1.1609335	-0.2152853	0.05745179	9.72E-05	0.00079163
Abcb8	335.064307	-1.8634626	-0.8979859	0.16013372	1.42E-09	2.94E-08
Abcc1	1650.91643	-1.1588815	-0.2127331	0.06799345	0.00093638	0.00551749
Abcc10	224.183868	1.04193267	0.05926206	0.1302945	0.56776396	0.73823146
Abcc2	13.582303	-1.0799651	-0.1109847	0.2343367	0.18721635	0.36469608
Abcc3	144.4504	1.78242628	0.83384241	0.19076825	9.26E-07	1.21E-05
Abcc4	4870.38639	-1.0445535	-0.0628863	0.05609348	0.24496968	0.43535815
Abcc5	1946.45884	1.15713767	0.21056051	0.06042048	0.00027237	0.00192982
Abcd1	292.530054	1.35770722	0.44117241	0.13911074	0.00025006	0.00180263
Abcd2	269.677641	1.0893682	0.12349166	0.12312346	0.22670926	0.41541204
Abcd3	1895.04719	1.1256551	0.17076485	0.05682262	0.00178133	0.00947626
Abcd4	287.543935	1.06864692	0.09578527	0.11031094	0.31862294	0.51720318
Abce1	5778.29405	-1.0271174	-0.0386011	0.05476012	0.465294	0.65839126
Abcf1	8115.70152	-1.2077585	-0.272332	0.04368663	1.90E-10	4.53E-09
Abcf3	1225.93318	1.04934728	0.06949222	0.07044112	0.29629159	0.49388357
Abcg1	4.31936013	-1.0216012	-0.0308322	0.21544987	0.40013373	0.60013033
Abcg4	44.465076	1.09493266	0.13084215	0.2416385	0.16500804	0.33361
Abhd1	15.2379867	1.01008462	0.01447616	0.20522368	0.82243036	0.90419748

Abhd10	550.304647	1.04944169	0.06962201	0.10170359	0.43679329	0.63431834
Abhd11	695.089895	1.13497829	0.1826647	0.08507112	0.01928196	0.06648773
Abhd12	666.252268	1.05383771	0.07565271	0.0891037	0.3501266	0.54976168
Abhd13	345.785141	1.00568532	0.00817895	0.12113888	0.93668886	0.96922329
Abhd14a	363.90563	1.10211027	0.14026857	0.11533834	0.15242081	0.31481951
Abhd14b	46.9478762	1.03518633	0.04989046	0.18436338	0.62254929	0.77850311
Abhd15	34.6909514	1.11598594	0.15831886	0.23943772	0.15926691	0.32554798
Abhd16a	408.45183	1.11147695	0.15247803	0.11240289	0.11383355	0.25582593
Abhd17a	224.32983	-1.1131512	-0.1546495	0.14849264	0.17168248	0.34357954
Abhd17b	554.549965	-1.1000549	-0.1375755	0.10852777	0.14243024	0.30053227
Abhd17c	283.54	-1.2473294	-0.3188425	0.14610673	0.00760205	0.03111253
Abhd18	142.91851	-1.385929	-0.4708533	0.2369014	0.00520661	0.02284132
Abhd2	1864.49427	-1.1253096	-0.1703219	0.06523727	0.0059067	0.02527217
Abhd4	979.538483	1.09728701	0.13394093	0.08959755	0.09930167	0.23181597
Abhd5	2192.85348	1.12241049	0.1666004	0.06557869	0.00744271	0.03061695
Abhd6	52.9571136	-1.9133459	-0.9360977	0.40611669	0.00093476	0.00551654
Abhd8	514.472186	1.09791481	0.13476611	0.11454316	0.16535419	0.33404624
Abi1	1827.20162	1.00600309	0.00863474	0.08035694	0.90893945	0.95251262
Abi2	1736.75788	1.12819689	0.17401887	0.07556555	0.01371842	0.05071657
Abitram	325.72145	-1.0751926	-0.1045952	0.1126785	0.27724626	0.47278669
Abl1	1392.59752	1.14339058	0.1933183	0.06840551	0.00281466	0.01388361
Abl2	1741.07452	-1.4437066	-0.5297776	0.06476187	4.03E-17	1.96E-15
Ablim2	3.15174397	-1.018606	-0.0265961	0.21664554	0.27071199	0.46597965
Abr	5567.4857	-1.1274452	-0.1730573	0.04367492	4.38E-05	0.0003895
Abracl	1309.5906	1.01561122	0.02234823	0.07038756	0.70822896	0.8350127
Abraxas1	537.161876	-1.0099	-0.0142125	0.10758142	0.87830674	0.93471042
Abraxas2	1542.07127	1.0132064	0.0189281	0.05908051	0.73048336	0.84898311
Abt1	613.83578	-1.2051655	-0.2692313	0.10168294	0.00304943	0.01478548
Abtb1	65.5030755	1.04482593	0.06326261	0.18169363	0.54702896	0.72383441
Abtb2	19.672324	-1.0110946	-0.0159181	0.20182722	0.82314054	0.90419748
Acaa2	1043.29358	-1.1009779	-0.1387855	0.08761348	0.08167874	0.20105778
Acaca	6682.66462	1.11779841	0.16066003	0.04883789	0.00078148	0.0047512
Acacb	80.293136	1.70998175	0.77398093	0.25538159	0.00016508	0.00126324
Acad11	287.398377	1.0909496	0.12558445	0.126229	0.22421248	0.41278555
Acad8	545.135078	1.00980236	0.01407296	0.1006843	0.87534071	0.93294904
Acad9	1906.91545	-1.1297235	-0.1759698	0.06631915	0.00511677	0.02252758
Acadl	4046.06282	1.04840758	0.06819969	0.07144505	0.31096041	0.50945171
Acadm	1032.1642	1.00526867	0.00758114	0.07506298	0.91541957	0.95578488
Acads	326.922432	1.09054053	0.12504339	0.13309211	0.24053523	0.43010648
Acadsb	2876.05739	1.24395658	0.31493612	0.05527297	3.76E-09	7.33E-08
Acadvl	923.166633	1.19040431	0.25145166	0.08290825	0.00103739	0.00601726
Acap1	1.84966712	-1.0107666	-0.0154498	0.21430745	0.5848938	0.74938527
Acap2	3425.52172	1.09149079	0.12629995	0.04727002	0.00629026	0.0266024
Acap3	724.65573	1.2297866	0.29840799	0.13156771	0.00693407	0.02882072
Acat1	5486.77491	1.23576752	0.30540736	0.07415946	1.30E-05	0.00013126
Acbd3	2404.13343	1.13401843	0.18144409	0.05286881	0.00039689	0.0026605
Acbd4	733.077096	1.18430115	0.24403598	0.09718501	0.00519153	0.02278297
Acbd5	1859.76222	1.14411515	0.19423227	0.0725774	0.00438609	0.01988877
Acbd6	882.8822	1.04171401	0.05895925	0.09483932	0.48931738	0.67718402
Ace2	1.63558865	-1.014793	-0.0211855	0.21613634	0.29794231	0.49571879

Acer3	337.834965	1.24598762	0.31728974	0.11078799	0.00123011	0.00689615
Acin1	9553.14015	-1.0954573	-0.1315332	0.05051659	0.00732537	0.0301924
Ackr3	1102.34263	1.0054639	0.00786129	0.09355855	0.92656022	0.96278138
Ackr4	44.3197443	-1.0959461	-0.1321768	0.22146443	0.22009242	0.40733182
Aco1	1713.95434	1.05123331	0.0720829	0.06336321	0.23344543	0.42285577
Acot11	17.7111828	-1.0300721	-0.0427454	0.20334483	0.58422026	0.74891039
Acot13	281.721876	-1.1074703	-0.1472681	0.1326516	0.1674043	0.33717753
Acot7	1594.72522	-1.0704195	-0.0981763	0.08467233	0.20503514	0.38899587
Acot8	327.64094	1.05050413	0.07108183	0.1318825	0.49856816	0.68452928
Acox1	1159.25947	1.12962179	0.17583983	0.07476704	0.01192231	0.04534584
Acox3	1154.36122	1.00381921	0.00549946	0.07666555	0.93947512	0.97069497
Acp2	1182.36099	-1.0443661	-0.0626276	0.0738588	0.36550682	0.56650241
Acp5	201.88684	-1.158063	-0.2117137	0.16944648	0.08477681	0.20677664
Acp6	477.406498	1.21611908	0.2822845	0.10213929	0.0020109	0.01047141
Acr	6.50342186	1.04088451	0.05781	0.22157421	0.21765814	0.40414564
Acrbp	10.1364986	1.02969232	0.04221331	0.21141527	0.49489725	0.68171472
Acsf2	500.633384	1.20304597	0.26669177	0.10914754	0.00547804	0.02381191
Acsf3	427.285036	1.40763453	0.49327281	0.10982647	1.06E-06	1.36E-05
Acsl1	2062.77161	1.04337394	0.0612563	0.05994564	0.28682031	0.48317186
Acsl3	1826.32143	1.30430768	0.38328423	0.08874147	3.67E-06	4.18E-05
Acsl6	110.582557	-4.0207334	-2.0074587	0.26361109	1.56E-15	6.61E-14
Acss2	408.633206	1.13440062	0.18193023	0.11518909	0.06406981	0.16845941
Acta1	8.69248885	-1.0378533	-0.0536025	0.22056865	0.22117418	0.40895514
Acta2	12.4846469	1.01902889	0.02719495	0.20685734	0.67646548	0.81335863
Actb	224080.668	1.0420727	0.05945593	0.05072364	0.22359948	0.41189378
Actc1	26.8601465	-1.0449912	-0.0634908	0.1968366	0.50307495	0.68784183
Actg2	4.16372373	-1.0966099	-0.1330504	0.28010376	0.00067009	0.00416087
Actl10	3.62631186	-1.0028565	-0.0041153	0.21230505	0.90711878	0.95192853
Actl6a	2159.94043	-1.1696363	-0.22606	0.0843181	0.0035072	0.01662127
Actn1	9677.64062	1.05650583	0.07930072	0.04504508	0.0716789	0.18232155
Actn3	468.598235	-1.0550978	-0.0773767	0.11344369	0.4222919	0.62078474
Actn4	5539.35017	-1.0319066	-0.0453124	0.04488133	0.30146925	0.49928806
Actr1a	5561.61358	1.01077798	0.01546615	0.05704543	0.78500516	0.87956967
Actr1b	224.728892	-1.0158431	-0.0226776	0.13761604	0.82918337	0.90792709
Actr3	12485.7152	-1.020879	-0.0298118	0.04179022	0.47230355	0.66385249
Actr5	1093.22647	1.31632552	0.3965163	0.08061564	1.93E-07	2.84E-06
Actr6	1004.0151	1.01776268	0.0254012	0.07296936	0.71207286	0.8373415
Actr8	1217.84092	-1.0374451	-0.053035	0.08602695	0.50044501	0.68583839
Actrt3	37.578274	1.00582289	0.00837629	0.18982447	0.9272505	0.9631135
Acvr1	1037.73471	-1.199872	-0.2628806	0.08877745	0.00122316	0.00686288
Acvr1b	236.346173	1.13960993	0.1885401	0.14210173	0.09301026	0.22117345
Acvr2a	549.644965	1.02486162	0.03542912	0.10626081	0.70193322	0.83028433
Acvr2b	230.071162	-1.1909073	-0.2520611	0.13510505	0.02288993	0.07639917
Acy1	546.996698	1.08710128	0.12048635	0.09469742	0.15558179	0.31975015
Acy3	79.7632901	1.48218615	0.56772665	0.23748746	0.00153659	0.0083159
Acyp1	80.1393911	1.10768307	0.14754515	0.18274142	0.21787404	0.40442426
Acyp2	69.9688238	1.00218106	0.00314318	0.16846896	0.97754598	0.98973149
Ada	807.713627	1.70960599	0.77366387	0.08990444	6.59E-19	3.69E-17
Adal	521.785867	1.07776897	0.10804796	0.09711504	0.21292372	0.39882335
Adam10	4649.22748	1.04834519	0.06811383	0.05684096	0.2138224	0.39968845

Adam12	87.5523355	-7.4590505	-2.898992	0.30389576	1.00E-22	7.02E-21
Adam15	1169.88394	-1.0913348	-0.1260937	0.08142996	0.09286645	0.2209239
Adam17	1832.86111	-1.1121051	-0.1532931	0.0563703	0.00471706	0.02112781
Adam1a	22.0956478	-1.0482308	-0.0679564	0.2045609	0.4458629	0.64231937
Adam22	842.114448	-1.0534386	-0.0751062	0.08190358	0.32049995	0.51917101
Adam23	2209.92682	1.07378171	0.10270074	0.07497865	0.14323156	0.30167647
Adam32	1.7380486	1.01788017	0.02556773	0.21618708	0.31490189	0.51409379
Adam8	3.0278599	1.01342363	0.01923738	0.21462919	0.50987823	0.69410628
Adam9	4193.29542	1.25289653	0.32526727	0.06105896	3.15E-08	5.33E-07
Adamts1	1105.72983	-2.1428338	-1.09952	0.08570704	7.66E-39	1.17E-36
Adamts15	4.2959596	-1.0277471	-0.0394853	0.21797338	0.25793635	0.45026721
Adamts3	21.7205299	-1.0934724	-0.1289168	0.24073105	0.16606415	0.33505777
Adamts4	36.5433963	1.12456516	0.16936726	0.23644199	0.1512899	0.31329189
Adamts6	1411.8475	-1.0626027	-0.0876022	0.08441356	0.2579962	0.45026721
Adamts7	817.11976	1.38542096	0.4703244	0.11060233	3.31E-06	3.81E-05
Adamtsl1	2.39821034	-1.043144	-0.0609383	0.22772411	0.00615455	0.02610588
Adamtsl4	611.220007	1.16279711	0.21759939	0.11293262	0.0257174	0.08333921
Adamtsl5	113.038955	1.02521814	0.03593091	0.16605418	0.73821549	0.85386369
Adar	1816.3861	-1.0715768	-0.0997353	0.06294673	0.09661725	0.22747051
Adarb1	839.017053	-1.3053437	-0.3844297	0.09237469	7.12E-06	7.60E-05
Adat1	459.631801	1.06844156	0.095508	0.09197566	0.25064105	0.44200329
Adat2	103.480368	-1.1066202	-0.1461602	0.18058854	0.21912843	0.40605166
Adck1	123.749773	1.30687884	0.3861254	0.21802071	0.0118697	0.04525322
Adck5	424.36936	-1.0821839	-0.1139457	0.11786676	0.24982946	0.44124022
Adcy10	2.34281739	1.00560473	0.00806334	0.21321938	0.80371788	0.89156365
Adcy3	2.46659494	1.00986508	0.01416257	0.21404035	0.6322433	0.78541342
Adcy6	1333.02538	1.09600265	0.13225128	0.07419191	0.05686277	0.1534145
Adcy7	297.716826	-2.6159192	-1.387318	0.1414382	5.24E-24	3.88E-22
Adcy9	499.390259	1.32729466	0.40848869	0.12219991	0.00016221	0.00124349
Add1	5659.97914	1.02720342	0.03872191	0.05466594	0.46421731	0.65752142
Add2	200.549442	-12.45417	-3.638557	0.26628513	3.34E-44	6.78E-42
Add3	4396.95484	-1.0137158	-0.0196533	0.04779527	0.6727983	0.81086907
Adgra2	4917.21639	1.18242589	0.24174977	0.04984711	6.06E-07	8.12E-06
Adgra3	770.182766	1.13144594	0.17816765	0.09969677	0.04390234	0.12637954
Adgrb2	10.1535547	1.08190871	0.11357877	0.24437378	0.12513374	0.27396397
Adgrd1	1039.62369	-2.6180196	-1.3884759	0.09833881	1.94E-46	4.52E-44
Adgre5	682.414045	-1.2497901	-0.3216859	0.09426324	0.00018714	0.00141764
Adgrf1	16.8921737	-4.8319246	-2.2725979	0.66575617	3.21E-05	0.0002934
Adgrf3	8.04452055	-1.0184713	-0.0264053	0.21030802	0.62314002	0.77883907
Adgrg1	2081.08881	-1.6695132	-0.7394275	0.09321397	1.91E-16	8.75E-15
Adgrg2	3.07608602	-1.0552785	-0.0776238	0.23598432	0.00158291	0.00853415
Adgrg6	11.73588	-1.0587187	-0.0823193	0.23110069	0.13465787	0.28845681
Adgrl1	11.9672228	5.2648781	2.39640013	0.70427096	3.24E-05	0.0002955
Adgrl2	4701.29578	-1.2492773	-0.3210938	0.04858632	1.25E-11	3.52E-10
Adh5	4054.39219	-1.0498509	-0.0701845	0.04685217	0.12280714	0.27030231
Adhfe1	15.4753037	-1.0304319	-0.0432492	0.20895779	0.52327806	0.70536179
Adi1	1100.94587	-1.039594	-0.0560202	0.08960157	0.49104987	0.67827785
Adipor1	6271.96537	1.17397591	0.2314028	0.05856707	3.89E-05	0.00034843
Adipor2	5239.57082	1.09494849	0.130863	0.0572714	0.01749861	0.06178534
Adk	2590.32387	-1.239784	-0.3100888	0.06384708	3.99E-07	5.50E-06

Adm	33.1663339	2.02967623	1.02124961	0.47462514	0.00124078	0.00693727
Adnp	7729.3856	1.2213741	0.28850516	0.04226427	3.72E-12	1.12E-10
Adnp2	1345.68984	-1.1282147	-0.1740416	0.07477738	0.01271512	0.04765381
Ado	170.331094	-1.0967369	-0.1332175	0.15968878	0.24563034	0.43588591
Adora2a	3.51988635	-1.0032509	-0.0046824	0.21206898	0.8981672	0.94695653
Adpgk	726.662682	1.04170255	0.05894339	0.08275392	0.43834872	0.63600036
Adprh	2858.88243	-1.2032523	-0.2669391	0.07068893	6.50E-05	0.00055519
Adprhl2	990.714515	1.0044021	0.00633695	0.07113417	0.92581056	0.96216399
Adprm	450.643297	-1.0626171	-0.0876218	0.1017928	0.3281255	0.52808662
Adsl	1663.41818	-1.0355973	-0.0504631	0.06303521	0.40138582	0.60099658
Aebp1	2.14180765	1.00864612	0.0124201	0.21351292	0.70391726	0.83178306
Aebp2	5534.2896	1.02932204	0.04169442	0.05085818	0.39687678	0.59720329
Aen	874.133082	-1.1759242	-0.233795	0.10663249	0.01256133	0.04716022
Afap1	3845.28301	1.15958641	0.21361033	0.06609688	0.00069424	0.00428422
Afdn	3753.31296	1.27089948	0.34584992	0.06397745	1.76E-08	3.08E-07
Aff1	3876.86756	-1.0162993	-0.0233253	0.05292474	0.64856532	0.79587333
Aff2	896.094284	1.30911177	0.38858827	0.08129438	4.01E-07	5.52E-06
Aff4	3465.64386	1.10927787	0.1496208	0.05172892	0.00280911	0.01387207
Afg1l	179.580133	1.0050443	0.0072591	0.12796408	0.94496416	0.97295716
Afg3l1	2778.95772	1.03912242	0.05536562	0.06341002	0.36057126	0.56088863
Afg3l2	1727.62213	1.01330448	0.01906774	0.06034782	0.74212458	0.85622047
Afmid	56.9872953	1.15730564	0.21076993	0.24483413	0.10638011	0.24404176
Afp	22.2210007	-1.0206223	-0.029449	0.20252022	0.690543	0.82279048
Aftph	1961.71015	-1.0370419	-0.0524742	0.06457156	0.39285714	0.594043
Aga	8680.16749	1.03438355	0.04877124	0.04784533	0.2923793	0.48946403
Agap1	1872.39932	-1.0294205	-0.0418324	0.06493899	0.49852418	0.68452928
Agap3	670.38536	-1.7850411	-0.8359573	0.11183968	6.09E-15	2.43E-13
Agbl3	233.560033	-1.0445848	-0.0629296	0.14040681	0.55743746	0.73141185
Agbl5	484.432977	1.69618406	0.76229273	0.1166077	5.43E-12	1.60E-10
Ager	18.2284125	-1.0153859	-0.0220281	0.20456912	0.74299419	0.85652948
Agfg1	2053.88229	-1.0737529	-0.102662	0.05669063	0.05976301	0.15952576
Agfg2	370.306637	1.15015857	0.20183278	0.10721449	0.03133909	0.09711025
Aggf1	2312.31915	-1.103992	-0.1427297	0.06601569	0.02268234	0.07584464
Agk	1463.10806	1.05668999	0.07955219	0.06874022	0.22179304	0.40961705
Agl	3495.54529	1.28725614	0.36429915	0.04909228	3.15E-14	1.15E-12
Agmo	1.59539197	1.01234855	0.01770609	0.21567004	0.3832456	0.58445306
Ago2	3117.86496	1.0685953	0.09571557	0.05799991	0.08616918	0.20937541
Ago3	490.232976	-1.0430395	-0.0607938	0.10689606	0.51185935	0.69591576
Ago4	556.746154	1.25204091	0.3242817	0.09640097	0.00022699	0.0016644
Agpat1	364.722664	1.02671794	0.03803989	0.13322945	0.71803975	0.84155927
Agpat2	436.267051	1.17591448	0.23378314	0.12345242	0.02494148	0.08144287
Agpat3	2117.26797	-1.3071918	-0.3864708	0.0867117	1.87E-06	2.29E-05
Agpat4	732.596677	-1.3831347	-0.4679417	0.09699689	2.33E-07	3.36E-06
Agpat5	1011.26181	-1.1165282	-0.1590197	0.09123604	0.053899	0.14712732
Agps	2108.07556	-1.1453661	-0.1958088	0.05958751	0.00060629	0.0038278
Agrn	1408.3824	1.03544356	0.05024891	0.09433823	0.55430324	0.72987441
Agtpbp1	1349.17509	1.36663347	0.45062637	0.07734564	1.06E-09	2.22E-08
Agtr1a	3.90404137	-1.0013977	-0.002015	0.21267259	0.95080103	0.97557351
Agtr2	1.65199885	1.01942816	0.02776011	0.21781764	0.07592816	0.19048958
Agtrap	232.72593	-1.0650757	-0.090956	0.12989066	0.38252941	0.58412228

Ahctf1	11710.1407	-1.1829915	-0.2424397	0.04996337	5.54E-07	7.44E-06
Ahcyl1	11442.6607	-1.0166628	-0.0238413	0.04368939	0.57650795	0.74378757
Ahcyl2	1794.90705	1.22389351	0.29147803	0.06192749	9.35E-07	1.22E-05
Ahdc1	693.657876	-1.1869802	-0.2472959	0.10934815	0.0097842	0.03856482
Ahi1	914.380094	-1.4255183	-0.5114866	0.08829529	1.01E-09	2.13E-08
Ahnak	74890.3946	1.00303076	0.00436585	0.05898145	0.93778342	0.96988581
Ahr	1711.43151	-1.0459	-0.0647449	0.07409467	0.35132073	0.55091425
Ahrr	688.511835	-1.1661722	-0.2217808	0.13530454	0.04411768	0.12674309
Ahsa1	6804.5753	1.03050604	0.04335296	0.05113617	0.38279176	0.58431392
Ahsa2	2033.42344	-1.0121208	-0.0173815	0.0744035	0.8026305	0.89096158
Aida	5962.37852	-1.0313789	-0.0445744	0.05419109	0.3948613	0.59576837
Aifm1	5745.56901	-1.1568087	-0.2101502	0.04879689	9.41E-06	9.85E-05
Aifm2	507.563934	1.10586197	0.14517132	0.13794807	0.18344636	0.3598695
Aifm3	3.53881549	1.0189267	0.02705027	0.21508375	0.44374686	0.64049546
Aig1	108.624691	1.32334599	0.4041903	0.21376509	0.00867072	0.03478609
Aim2	1972.98364	-1.6985881	-0.7643361	0.08235877	1.58E-21	1.04E-19
Aimp1	5163.40486	-1.1603211	-0.2145241	0.06043948	0.00020772	0.00154345
Aip	1223.67992	1.09367205	0.1291802	0.07797817	0.07460551	0.18796129
Ajuba	5953.90911	1.17955347	0.23824081	0.06193026	7.98E-05	0.000667
Ak1	9.38160065	1.0664214	0.09277764	0.23358731	0.14840687	0.30891973
Ak2	11343.3686	1.05840564	0.08189266	0.05171023	0.10253399	0.23764436
Ak5	1.94618666	-1.0097066	-0.0139361	0.2149346	0.53695354	0.71579699
Akap1	3965.1234	-1.0111103	-0.0159404	0.06285843	0.79003471	0.88271942
Akap10	632.423423	-1.1075628	-0.1473885	0.09483103	0.0828003	0.20296055
Akap11	8654.05179	1.00987912	0.01418261	0.04856656	0.76481464	0.86970932
Akap12	15999.429	1.10995011	0.15049483	0.06568459	0.01588504	0.05706321
Akap13	4586.69477	-1.0973307	-0.1339984	0.05067645	0.00638497	0.0269338
Akap17b	208.125579	1.20636977	0.27067218	0.18957108	0.04315897	0.12480055
Akap3	85.751792	-1.0535932	-0.0753179	0.166458	0.49349595	0.68023673
Akap5	3.36596908	-1.0148227	-0.0212277	0.21417661	0.53088426	0.71104678
Akap7	468.830786	1.64604469	0.71900351	0.12556673	9.94E-10	2.10E-08
Akap8	3168.4434	-1.0479	-0.0675011	0.05994547	0.24094394	0.43059318
Akap9	7824.5409	1.03508557	0.04975004	0.06029552	0.39194119	0.59326086
Akip1	799.014628	-1.1002338	-0.1378102	0.08740458	0.08373578	0.20478339
Akirin1	2473.10183	-1.0708374	-0.0987394	0.0575261	0.07491109	0.18852871
Akirin2	1320.06109	-1.1979222	-0.2605342	0.07994013	0.00046044	0.0030232
Akna	314.10526	-1.0529341	-0.0744151	0.14775511	0.49626551	0.68261154
Aknad1	2.31139761	1.00702221	0.01009551	0.21349407	0.75005454	0.86119468
Akr1b3	4762.72504	-1.1374493	-0.1858023	0.06497197	0.00259394	0.01300524
Akr1c19	1.97568489	-1.0101361	-0.0145497	0.21443943	0.5896915	0.75283517
Akr1e1	632.518672	-1.1832682	-0.2427771	0.12266718	0.01952225	0.06726196
Akr7a5	477.773068	-1.0266629	-0.0379626	0.10147316	0.67107426	0.81006985
Akt1	5049.91451	1.0134847	0.01932432	0.05169131	0.70393131	0.83178306
Akt1s1	691.514055	1.07254988	0.10104475	0.08742335	0.20526073	0.38936619
Akt2	1208.52214	1.08385676	0.11617411	0.07897167	0.11309694	0.25480406
Akt3	2142.46762	-1.0586068	-0.0821669	0.08445169	0.28900758	0.48553274
Alad	775.488684	1.02835069	0.04033234	0.08868566	0.61815434	0.77556876
Alas1	805.578329	-1.0633349	-0.0885961	0.08867966	0.25360493	0.44510871
Alcam	8721.95641	1.17621647	0.23415359	0.04795232	5.32E-07	7.18E-06
Aldh16a1	1714.59677	1.14703908	0.19791455	0.09024741	0.0157344	0.05663324

Aldh18a1	18.5183499	1.0283042	0.04026712	0.20075561	0.62298096	0.77883907
Aldh1a1	51.1563617	-1.8318209	-0.8732784	0.32828129	0.00042752	0.00283614
Aldh2	1936.96941	1.06267291	0.08769761	0.06947399	0.18161164	0.35763952
Aldh3a2	226.128524	1.04872351	0.06863437	0.14257767	0.52765473	0.70881471
Aldh3b1	3.00697898	-1.0255344	-0.0363758	0.2172596	0.2873567	0.48368923
Aldh4a1	264.65974	-1.0312124	-0.0443416	0.11801388	0.65077818	0.79746983
Aldh5a1	874.774295	1.15443011	0.20718083	0.07368304	0.00272003	0.01352626
Aldh6a1	559.990096	1.08060325	0.11183693	0.09739328	0.19836529	0.37931921
Aldh8a1	3.43598142	-1.0772112	-0.1073011	0.25623986	0.00039184	0.00263211
Aldh9a1	1074.01624	1.3219729	0.4026926	0.0779301	5.23E-08	8.52E-07
Aldoa	12310.4003	-1.51399	-0.5983556	0.06024511	3.65E-24	2.75E-22
Aldoart2	187.129143	-1.7145851	-0.7778595	0.19255145	3.88E-06	4.40E-05
Alg1	169.049288	1.0125386	0.01797691	0.1313556	0.86413465	0.92713638
Alg10b	351.317019	-1.0094162	-0.0135212	0.11145526	0.87398098	0.93213247
Alg12	833.477737	1.12494633	0.16985618	0.0969666	0.04993869	0.13888723
Alg14	284.620801	-1.037147	-0.0526204	0.12953176	0.61116745	0.77118351
Alg2	1888.82687	1.09362595	0.12911938	0.05847618	0.02112335	0.07169849
Alg3	324.720788	1.19553193	0.25765266	0.14225092	0.02488262	0.08137077
Alg5	797.819038	1.09441359	0.13015805	0.07954995	0.07677943	0.19215162
Alg6	740.040297	-1.0784476	-0.1089561	0.07933622	0.13836169	0.29403006
Alg8	718.847339	-1.1107136	-0.1514868	0.09738498	0.0807987	0.19943512
Alg9	1103.99515	-1.0648229	-0.0906135	0.07271893	0.18242201	0.35846387
Alkbh1	414.454171	-1.2319527	-0.3009468	0.11024619	0.0019932	0.01039393
Alkbh2	474.778781	1.03717643	0.05266132	0.10799405	0.57387897	0.74199183
Alkbh4	144.529098	1.00029913	0.00043148	0.15499639	0.99826796	0.99956946
Alkbh5	2474.05034	-1.3754471	-0.4599007	0.07725398	4.51E-10	9.97E-09
Alkbh6	322.726965	1.08136292	0.1128508	0.10715325	0.22560093	0.41412563
Alkbh7	157.883968	1.07313937	0.10183745	0.14210322	0.34995694	0.54965036
Alkbh8	1089.31853	1.00440155	0.00633616	0.08088353	0.93345045	0.9674353
Alms1	2088.09161	-1.8253529	-0.8681754	0.06366536	1.98E-43	3.78E-41
Aloxe3	50.878928	1.05016702	0.07061879	0.19714178	0.47072528	0.66270466
Alpk1	177.051972	1.01622619	0.02322156	0.15149197	0.83136077	0.90937848
Als2	1886.87589	1.00841278	0.01208631	0.06410919	0.84407856	0.91593785
Als2cl	1244.535	1.11027602	0.15091838	0.09336358	0.07253017	0.18387324
Amacr	772.4697	-1.0763256	-0.1061146	0.07369972	0.12430581	0.27261725
Ambra1	1043.1018	1.12498159	0.16990139	0.07874246	0.02012308	0.06897956
Amdhd2	2215.37291	1.04476796	0.06318257	0.07195878	0.35117018	0.5507456
Amer1	1000.64424	-1.2325036	-0.3015919	0.07489341	1.87E-05	0.00018046
Amfr	1888.76098	1.17405587	0.23150106	0.0763046	0.0011852	0.00669713
Amh	31.9266317	-1.1439306	-0.1939995	0.30434113	0.06742732	0.17470548
Amhr2	1.74480739	1.0100424	0.01441585	0.21474922	0.56292977	0.73507789
Amigo3	20.9585006	1.0448445	0.06328825	0.20820393	0.44220567	0.6394959
Ammecr1	1116.79348	-1.3430579	-0.4255215	0.07471915	2.45E-09	4.93E-08
Ammecr1l	1331.64752	1.02562716	0.03650637	0.07069382	0.58503149	0.74938527
Amn1	128.887623	-1.0432409	-0.0610724	0.15362904	0.57759492	0.74410617
Amot	2030.74673	1.00000097	1.39E-06	0.06118923	0.9999422	0.9999422
Amotl1	8910.12308	1.38203343	0.46679251	0.05052807	4.44E-21	2.81E-19
Amotl2	9366.63139	1.54742986	0.62987402	0.0587263	8.72E-28	7.92E-26
Ampd2	2107.63891	1.04766429	0.06717651	0.07722559	0.35111119	0.55072051
Ampd3	24.7987988	-2.3347532	-1.22327	0.85253524	0.00332512	0.01592744

Amt	79.1109088	1.2426515	0.31342175	0.28740041	0.04229786	0.12290777
Amz2	842.590656	1.16016274	0.21432719	0.08089195	0.00422674	0.01935117
Anapc1	19659.7222	1.02356723	0.03360586	0.04325147	0.35179434	0.55152191
Anapc10	617.475584	1.15904767	0.2129399	0.09860651	0.01568305	0.05652783
Anapc11	652.279553	1.15818461	0.21186524	0.12981026	0.0477617	0.13455628
Anapc13	458.223942	-1.0192532	-0.0275125	0.11401846	0.77539914	0.87498793
Anapc15	399.026306	1.0989067	0.13606891	0.11538422	0.16664643	0.33575664
Anapc16	610.972488	-1.0025532	-0.0036788	0.10375416	0.96681404	0.98394279
Anapc2	1826.36491	1.01448762	0.02075125	0.06961431	0.75346116	0.86298066
Anapc4	2652.2596	1.02411036	0.03437119	0.06379907	0.57291324	0.7414648
Anapc5	9487.33024	1.08995272	0.12426556	0.04875133	0.00877707	0.03513571
Anapc7	1252.15741	1.00787461	0.01131617	0.06825167	0.86181538	0.92618801
Angel1	690.183893	1.27715432	0.35293286	0.1232997	0.00101691	0.00592119
Angel2	1115.34001	1.10435156	0.14319951	0.0710592	0.03251172	0.10006612
Angptl4	8.35314243	-1.0171805	-0.0245757	0.21056018	0.63387659	0.7864639
Angptl6	10.7561664	1.04272845	0.0603635	0.21665624	0.3423293	0.54185573
Ank	3500.97024	-1.3278225	-0.4090623	0.06887679	6.05E-10	1.32E-08
Ank1	95.7847978	-1.8666022	-0.9004145	0.25474005	2.62E-05	0.00024376
Ank2	2394.04898	1.08747065	0.12097647	0.05594381	0.02481284	0.08125064
Ank3	1263.65272	1.07412096	0.10315646	0.09658727	0.23224356	0.42139377
Ankar	31.7040461	1.0553905	0.07777691	0.20452166	0.40657724	0.60617486
Ankfy1	1015.35044	1.19744599	0.25996058	0.08216741	0.00064422	0.00402557
Ankib1	2837.7225	-1.1084667	-0.1485655	0.05809503	0.00769479	0.03141182
Ankle1	287.402528	1.21288121	0.27843826	0.14972375	0.0196755	0.06771712
Ankle2	1616.81169	1.03681166	0.05215384	0.06520265	0.40141505	0.60099658
Ankmy1	35.3363464	1.02241696	0.03198368	0.18751466	0.73837503	0.85397112
Ankmy2	641.920582	-1.3804895	-0.4651799	0.10737948	2.41E-06	2.88E-05
Ankra2	218.847981	1.06358409	0.08893411	0.14693663	0.41939065	0.61826923
Ankrd1	1791.52454	1.30889323	0.38834742	0.07281789	2.25E-08	3.89E-07
Ankrd11	12243.3883	-1.08389	-0.1162184	0.0480267	0.01287309	0.04816131
Ankrd12	5132.92349	1.10260615	0.14091756	0.07583982	0.04667188	0.13212527
Ankrd13a	3178.04102	1.19487283	0.25685708	0.05187464	3.36E-07	4.71E-06
Ankrd13b	3.51482679	1.02988274	0.04248009	0.21841314	0.25681412	0.44899534
Ankrd13c	4102.45574	-1.1284211	-0.1743055	0.0544853	0.00091238	0.00540842
Ankrd13d	9.81027672	-1.0180188	-0.0257642	0.21124317	0.60790523	0.76830819
Ankrd16	66.0024414	1.030895	0.0438974	0.17804196	0.67431615	0.81200681
Ankrd17	11457.2006	-1.0173754	-0.0248521	0.05448965	0.63675589	0.78844056
Ankrd22	2.01882154	-1.0091127	-0.0130872	0.21464744	0.58507247	0.74938527
Ankrd24	3.9387067	-1.0244054	-0.0347868	0.21648994	0.33368086	0.53357282
Ankrd26	3010.3172	-1.0153641	-0.0219972	0.06133048	0.70758286	0.83463503
Ankrd27	556.882839	1.21913437	0.28585715	0.0984964	0.00129397	0.00718483
Ankrd28	1356.48475	-1.2246564	-0.292377	0.07363642	2.57E-05	0.00023904
Ankrd29	4.53308416	-1.0263724	-0.0375543	0.2169506	0.30920489	0.50753364
Ankrd34a	69.9095912	1.03480984	0.04936568	0.17220018	0.64567821	0.79392941
Ankrd35	15.090995	1.11654588	0.15904253	0.28813861	0.04913646	0.13737189
Ankrd37	35.8522678	-1.2292106	-0.2977322	0.36192194	0.04372626	0.12604262
Ankrd39	38.2516541	1.09626052	0.13259069	0.2312985	0.19779009	0.37850808
Ankrd42	268.066196	1.19548102	0.25759122	0.13349767	0.01979968	0.06803486
Ankrd44	567.691121	1.12654447	0.17190426	0.08987749	0.03516836	0.10635191
Ankrd46	631.251009	1.08809145	0.12179982	0.10396421	0.18105834	0.35698896

Ankrd49	1114.60291	-1.079976	-0.1109992	0.07815465	0.12634501	0.27548588
Ankrd52	2367.13827	1.12995631	0.17626699	0.06158804	0.00273396	0.01358341
Ankrd54	63.2856177	1.04275045	0.06039393	0.17787869	0.56857417	0.7386102
Ankrd61	43.3968507	1.04174421	0.05900108	0.20387061	0.493793	0.68052591
Ankrd65	3.85752983	-1.0123853	-0.0177585	0.21364542	0.60408138	0.76556019
Ankrd66	2.99165737	1.00886496	0.01273308	0.21292258	0.72432857	0.84454605
Ankrd9	54.6648578	1.06807795	0.09501694	0.21382731	0.31315792	0.51207488
Anks1	1197.381	1.16142346	0.21589408	0.08157195	0.00423853	0.01939821
Anks3	351.493497	-1.0714919	-0.0996209	0.10614587	0.28010487	0.47590659
Anks6	234.831946	1.02817149	0.04008092	0.13814167	0.7071762	0.83438584
Ankzf1	647.076737	-1.0643861	-0.0900215	0.08938992	0.26626162	0.46029843
Anln	13736.9684	-1.0091792	-0.0131824	0.04518928	0.77316785	0.87382468
Ano1	3.47817943	-1.0270444	-0.0384985	0.21771141	0.26883616	0.46368537
Ano10	2230.80077	1.1219912	0.16606137	0.05553023	0.00190441	0.01001869
Ano6	5081.43506	-1.056934	-0.0798853	0.05073084	0.10463624	0.24112075
Ano8	813.867674	1.00377477	0.00543559	0.09680478	0.94984251	0.97503667
Anp32a	4071.4758	-1.2346158	-0.3040622	0.06426365	7.73E-07	1.02E-05
Anp32b	11305.947	-1.080491	-0.1116871	0.05520485	0.03544961	0.10694994
Antxr1	17.4809631	-1.0737173	-0.1026142	0.22979294	0.2109478	0.3963521
Antxr2	4256.39209	-1.7593626	-0.8150528	0.06049292	1.89E-42	3.41E-40
Anxa1	9995.22278	-1.7477739	-0.8055186	0.06101365	6.99E-41	1.13E-38
Anxa11	1933.20856	-1.0430462	-0.0608031	0.06096701	0.30115943	0.49905648
Anxa2	30814.5759	1.08613041	0.11919734	0.04168982	0.00455778	0.0205799
Anxa3	44.1551609	1.02877048	0.04092115	0.18137806	0.68831183	0.82128116
Anxa4	1944.29698	1.28380451	0.36042553	0.07965409	1.58E-06	1.97E-05
Anxa5	3450.28952	-1.0218455	-0.0311771	0.06885727	0.56705135	0.7378158
Anxa6	3203.92869	1.16078613	0.21510218	0.05665727	8.05E-05	0.00067202
Anxa8	436.377765	-1.8022523	-0.849801	0.14694269	5.57E-10	1.22E-08
Anxa9	10.9289742	-1.0169507	-0.0242497	0.20816562	0.6846119	0.81884953
Aoc2	39.8113552	1.01891036	0.02702714	0.20011989	0.73288305	0.85069154
Aopep	204.493069	1.13933838	0.18819628	0.13648775	0.08639034	0.20968695
Aox1	99.1995072	-1.9053929	-0.9300885	0.27087927	3.39E-05	0.00030802
Ap1ar	2202.72543	-1.1360623	-0.1840419	0.05665905	0.00071171	0.00437689
Ap1b1	7856.441	1.11139168	0.15236734	0.04751058	0.00098616	0.00576571
Ap1g1	1958.98397	1.03315606	0.04705819	0.07246875	0.48870516	0.67693697
Ap1g2	281.394189	1.10364484	0.14227598	0.13065426	0.17931619	0.35448155
Ap1m1	2005.16171	1.06462489	0.0903452	0.06309767	0.13342282	0.28648112
Ap1s1	1456.36983	1.25565908	0.32844482	0.07777815	7.17E-06	7.65E-05
Ap1s2	218.178744	-1.0319935	-0.0454339	0.12775781	0.65728706	0.80140161
Ap2a1	3803.31714	1.02622074	0.03734108	0.05985351	0.51493798	0.69854448
Ap2a2	3738.10825	1.21163787	0.27695857	0.05357406	9.41E-08	1.47E-06
Ap2b1	6621.79167	1.04189607	0.05921137	0.05254816	0.241585	0.43149803
Ap2m1	5254.3018	-1.0169875	-0.024302	0.06694694	0.70165385	0.83028433
Ap2s1	833.237939	1.1728309	0.22999502	0.10028815	0.01016807	0.03980837
Ap3b1	1860.71803	1.05378862	0.0755855	0.06497867	0.22189741	0.40970105
Ap3d1	4848.35579	1.03662176	0.05188958	0.04812383	0.26740323	0.46177344
Ap3m1	2151.11666	1.1361229	0.18411891	0.05526002	0.00054888	0.00353683
Ap3s2	247.919763	1.22204733	0.28930016	0.15960578	0.02044526	0.06987826
Ap4e1	448.362705	1.02349861	0.03350915	0.09507871	0.69503033	0.82560632
Ap4m1	485.475829	-1.081117	-0.1125227	0.10633854	0.2229409	0.41127203

Ap4s1	240.545637	-1.1768811	-0.2349686	0.12845422	0.02796682	0.08858927
Ap5b1	736.549589	-1.0135671	-0.0194415	0.1243911	0.84686351	0.91769146
Ap5m1	349.742135	1.02316091	0.03303305	0.12000988	0.74242419	0.85638464
Ap5s1	266.027555	-1.0509884	-0.0717468	0.12384065	0.4792216	0.66973911
Ap5z1	236.799314	1.19421687	0.25606485	0.18039992	0.04830161	0.13561369
Apaf1	506.575111	1.00825814	0.01186506	0.09574423	0.89032332	0.9416229
Apba1	262.442747	1.40221921	0.4877119	0.1514324	0.00017502	0.00133374
Apba2	8.26994437	-1.0375378	-0.0531639	0.21825158	0.30276245	0.50059975
Apba3	298.378951	-1.050252	-0.0707356	0.13475778	0.49805431	0.68439561
Apbb1	1228.53665	1.12397206	0.16860617	0.08280257	0.02691703	0.08620179
Apbb1ip	2624.64469	-1.2628189	-0.3366477	0.06030187	7.07E-09	1.32E-07
Apbb2	1271.05692	1.04741997	0.06684002	0.08292876	0.38229653	0.58397551
Apbb3	90.8738225	-1.0150914	-0.0216096	0.15971054	0.83991182	0.9142043
Apc	5465.20145	-1.231576	-0.3005057	0.05240959	3.53E-09	6.92E-08
Apc2	3.77161877	-1.0228484	-0.0325923	0.21695327	0.28726044	0.4836759
Apeh	989.881604	1.1685065	0.22466575	0.09540559	0.00896628	0.03572446
Apex2	468.651677	-1.014853	-0.0212708	0.11232993	0.82049913	0.90334055
Aph1a	386.368779	-1.0215477	-0.0307566	0.12607382	0.76244843	0.86871514
Aplf	833.515862	-1.222515	-0.2898522	0.0847568	0.00022351	0.00164169
Apln	10.2404819	-1.0634994	-0.0888192	0.23569135	0.09553465	0.22558504
Aplp2	9567.89424	-1.2102212	-0.2752708	0.04593159	8.44E-10	1.81E-08
Apmap	2073.30132	1.03910172	0.05533689	0.05742684	0.31744818	0.51628103
Apoa2	67.6141895	1.14359514	0.19357639	0.21378562	0.12898531	0.27970309
Apobec3	1757.21566	-1.0559773	-0.0785788	0.07809481	0.27893329	0.47465532
Apom	11.1252968	1.02779896	0.03955809	0.21017627	0.53159069	0.71171371
Apoo	1126.19574	-1.007663	-0.0110132	0.06908143	0.86567822	0.92774752
Apopt1	502.740009	1.01147041	0.01645412	0.09350224	0.84593343	0.91694087
App	19413.5374	1.27414768	0.3495325	0.03910926	1.11E-19	6.53E-18
Appbp2	4326.20835	-1.0331724	-0.0470811	0.04578844	0.29189946	0.4888524
Appl1	1438.1418	1.07208086	0.10041373	0.08722779	0.20668243	0.39119212
Appl2	523.824378	1.17661188	0.23463851	0.1235276	0.02445676	0.08041352
Aptx	230.903091	1.06677758	0.09325942	0.12749354	0.36929629	0.57058081
Aqp1	11.8942865	-5.2572055	-2.3942961	0.79360604	0.00013028	0.00102637
Aqp11	123.933379	2.22007313	1.1506072	0.19327694	1.60E-10	3.89E-09
Aqp3	66.0826596	-1.0990469	-0.1362529	0.20355352	0.23840935	0.42821422
Aqr	6107.3253	-1.0254194	-0.0362142	0.05589319	0.50191252	0.68698573
Araf	914.545005	1.01698802	0.02430268	0.10099793	0.78596696	0.88018569
Arap1	1032.61523	1.05174438	0.07278411	0.08096433	0.33134878	0.53123627
Arap2	9.63000119	1.02752463	0.03917297	0.21020619	0.53208294	0.71201675
Arap3	1074.10234	-1.3719437	-0.4562213	0.07338727	9.09E-11	2.28E-09
Arcn1	4749.89871	1.06461389	0.0903303	0.05198693	0.07289225	0.18453552
Areg	97.9609669	-184.08046	-7.5241927	0.74831998	2.07E-23	1.52E-21
Arel1	4146.25471	1.13094954	0.17753456	0.05369576	0.00062242	0.00391422
Arf1	2368.03118	-1.0030539	-0.0043991	0.06659368	0.94395356	0.9727006
Arf2	8630.13437	1.05476532	0.07692204	0.04869661	0.10424683	0.24052627
Arf3	780.347033	-1.0550071	-0.0772528	0.08273748	0.31060542	0.50906543
Arf4	1701.92587	1.10749102	0.147295	0.05993561	0.01030691	0.04023813
Arf5	1171.71961	-1.0360367	-0.0510751	0.0866476	0.51848125	0.70149396
Arf6	1735.32269	1.06927192	0.09662879	0.0559078	0.07315653	0.18503211
Arfgap1	1792.40303	1.28750878	0.36458227	0.06961808	4.20E-08	6.95E-07

Arfgap2	1531.03802	1.04745339	0.06688605	0.05855154	0.23504964	0.42486043
Arfgap3	588.14009	1.38571269	0.47062817	0.11815549	1.08E-05	0.00011213
Arfgef1	3747.35216	1.02261329	0.03226069	0.06691478	0.61248981	0.77201559
Arfgef2	5380.54384	1.47060804	0.55641278	0.06105827	8.21E-21	5.08E-19
Arfip2	879.260779	1.06417204	0.08973141	0.09005657	0.27178927	0.46733162
Arglu1	1701.52577	-1.2384387	-0.3085225	0.06626016	1.09E-06	1.39E-05
Arhgap1	2459.30185	1.04426863	0.06249289	0.07502932	0.37377858	0.57528579
Arhgap10	1414.64678	-1.1112068	-0.1521273	0.06426154	0.01279939	0.04795562
Arhgap11a	7214.91923	1.0613913	0.08595663	0.05849028	0.12637897	0.27551304
Arhgap12	1956.24166	-1.0373654	-0.0529242	0.06908671	0.41787546	0.61684544
Arhgap15	3.42096127	-1.0222895	-0.0318038	0.21636598	0.33687215	0.53673286
Arhgap17	1481.49839	-1.0085354	-0.0122617	0.07415587	0.85957869	0.9247672
Arhgap18	3957.51522	-1.2693866	-0.3441316	0.05455511	8.19E-11	2.06E-09
Arhgap19	1867.31056	1.15414756	0.20682769	0.06724158	0.00117902	0.00667401
Arhgap20	186.903343	1.48877548	0.5741262	0.21156681	0.00062423	0.00391981
Arhgap21	9113.81127	1.00894877	0.01285292	0.05933401	0.82071288	0.90344006
Arhgap22	537.07597	-2.252205	-1.1713382	0.13239553	5.70E-20	3.41E-18
Arhgap23	1936.90746	1.12385396	0.16845458	0.06629627	0.0074219	0.03055096
Arhgap24	1081.73301	-1.0566459	-0.079492	0.08005138	0.28289984	0.47906941
Arhgap25	1082.55925	-1.2402689	-0.3106529	0.11190528	0.00165693	0.00889575
Arhgap27	43.639798	2.32750664	1.21878528	0.43608713	0.00022233	0.00163489
Arhgap28	199.609742	-1.6348337	-0.7091439	0.18305529	8.60E-06	9.04E-05
Arhgap29	3585.27104	1.04340539	0.06129978	0.06015953	0.28784472	0.48408702
Arhgap31	5606.55414	-1.1496614	-0.2012091	0.04860505	1.99E-05	0.00019013
Arhgap32	3370.42118	-1.0157625	-0.0225631	0.04965389	0.63880653	0.79036561
Arhgap33	125.092748	1.48224991	0.56778871	0.19589902	0.00039356	0.00264227
Arhgap35	4145.69701	1.10615393	0.14555216	0.05458548	0.00574883	0.02472056
Arhgap39	669.381357	-1.1665079	-0.2221961	0.1023344	0.0142946	0.05248318
Arhgap45	6.65323051	1.03135885	0.04454639	0.21608766	0.36876547	0.57010448
Arhgap5	5792.06776	1.02639928	0.03759206	0.05113666	0.4501297	0.64570677
Arhgap6	1043.06201	-1.1683221	-0.2244381	0.08958599	0.00599264	0.02552899
Arhgap8	33.4383192	-1.0341262	-0.0484122	0.19177086	0.61071258	0.7709133
Arhgap9	6.85758402	-1.0591178	-0.0828631	0.23663355	0.03052469	0.09509273
Arhgdia	9883.81044	-1.001033	-0.0014896	0.05601129	0.97740579	0.98968999
Arhgef1	1465.36154	1.04745895	0.0668937	0.08628315	0.39731382	0.59750998
Arhgef10	3549.85837	1.04817377	0.06787792	0.05822331	0.22560005	0.41412563
Arhgef10l	445.653536	1.31297051	0.39283452	0.11711547	0.0001645	0.00125957
Arhgef11	1384.80461	1.05526105	0.07759994	0.06304429	0.19757843	0.37832283
Arhgef12	8486.4967	1.03418594	0.0484956	0.04321201	0.25240822	0.44377565
Arhgef15	4.97893119	-1.0258507	-0.0368207	0.21714486	0.30211358	0.49988052
Arhgef16	7.98301897	-1.0412848	-0.0583648	0.22054391	0.25211819	0.44337103
Arhgef17	2037.25684	1.09465275	0.13047328	0.06583334	0.03681261	0.11027603
Arhgef18	845.698591	1.12184563	0.16587418	0.10179127	0.06477602	0.16998459
Arhgef19	20.8414583	-1.0453737	-0.0640187	0.20567541	0.454364	0.64916383
Arhgef2	1980.81783	-1.1030771	-0.1415336	0.06555609	0.02258594	0.07564076
Arhgef25	4.21943293	-1.0683059	-0.0953249	0.24380708	0.0254811	0.0827619
Arhgef28	2084.12301	1.00629993	0.00906037	0.05426612	0.86410699	0.92713638
Arhgef37	96.214742	1.13341869	0.1806809	0.21699212	0.14738852	0.30785048
Arhgef39	2.10913255	1.00584169	0.00840326	0.21380931	0.76668961	0.87106819
Arhgef4	59.6908447	1.56341778	0.64470335	0.36877314	0.00459292	0.02068754

Arhgef40	3189.74796	1.13926878	0.18810815	0.05625297	0.00051361	0.00333298
Arhgef5	3009.94444	1.00711134	0.01022318	0.05168129	0.83907607	0.91399358
Arhgef6	4039.14821	1.01657657	0.02371888	0.05267428	0.64290559	0.79201333
Arhgef7	2723.57481	1.036237	0.051354	0.054115	0.32696598	0.52673008
Arhgef9	2.70684428	-1.0390812	-0.0553084	0.225589	0.00643286	0.0270535
Arid1a	5956.8434	-1.0081502	-0.0117105	0.04739627	0.79997891	0.88925111
Arid1b	2648.24618	-1.1814009	-0.2404986	0.06576258	0.00012094	0.00096344
Arid2	4753.80728	-1.0479218	-0.0675311	0.04621404	0.13413744	0.28772615
Arid3a	283.707115	1.18846986	0.24910531	0.14215124	0.02947776	0.09239297
Arid3b	80.109574	-1.0994987	-0.1368459	0.20932868	0.23019631	0.41892523
Arid4a	1843.63545	1.01278448	0.0183272	0.07430112	0.79346872	0.88516366
Arid4b	2505.55437	1.00145398	0.00209612	0.06597393	0.97386448	0.98805671
Arid5a	493.144214	-1.2032986	-0.2669946	0.10031157	0.00295794	0.01441541
Arid5b	3536.32513	-1.4708269	-0.5566275	0.06841808	5.60E-17	2.68E-15
Arih1	4076.38302	-1.0262267	-0.0373495	0.05170102	0.46153612	0.65522729
Arih2	1411.08712	1.13368996	0.18102615	0.06641093	0.00409246	0.01883769
Arl1	1440.21666	-1.000709	-0.0010225	0.0987973	0.99050237	0.99554829
Arl11	13.7475294	1.06706958	0.09365425	0.22831119	0.21462067	0.40077126
Arl13b	1574.7698	-1.0038739	-0.0055781	0.06614975	0.92882913	0.9641278
Arl14ep	594.017587	-1.0656456	-0.0917277	0.08935703	0.25804531	0.45026721
Arl16	74.6987193	1.05729163	0.08037336	0.16641971	0.47119154	0.66285566
Arl2	964.71412	1.07365465	0.10253001	0.10525642	0.26418769	0.45767849
Arl2bp	700.94905	1.00909531	0.01306244	0.10808054	0.89021023	0.9416229
Arl3	2500.52982	1.00249567	0.003596	0.06694578	0.95572762	0.97872498
Arl4a	156.692884	-1.7011934	-0.7665472	0.18918057	3.98E-06	4.49E-05
Arl4c	189.095318	1.04278336	0.06043946	0.13269099	0.56601248	0.7369265
Arl4d	143.686802	-1.202439	-0.2659638	0.20738191	0.05254244	0.14428053
Arl5a	589.624474	-1.1606064	-0.2148788	0.15322709	0.06839923	0.17647415
Arl5b	117.169243	-1.1064685	-0.1459624	0.19557253	0.22027547	0.40752872
Arl6	912.065581	-1.0091353	-0.0131197	0.08267702	0.86275679	0.92665838
Arl6ip1	3922.95697	1.07596488	0.10563098	0.07674935	0.14461407	0.30379387
Arl6ip4	1089.78536	1.03383181	0.0480015	0.0741978	0.49100012	0.67827785
Arl6ip5	1579.42163	-1.1014902	-0.1394566	0.07464136	0.04561489	0.13002375
Arl6ip6	770.756786	-1.1491561	-0.2005748	0.08118626	0.00748183	0.03072852
Arl8a	1304.43499	1.22871001	0.29714446	0.08632263	0.00019863	0.0014871
Arl8b	526.461798	1.17495325	0.23260336	0.12993015	0.0309871	0.09625235
Arl9	117.946535	1.21702132	0.28335444	0.18443862	0.03346618	0.10243771
Armc1	2392.3871	-1.1909728	-0.2521404	0.05664993	3.91E-06	4.42E-05
Armc10	222.465398	-1.1282313	-0.1740629	0.1340747	0.10746048	0.24542141
Armc5	588.043887	1.01311763	0.01880169	0.09710177	0.82884403	0.90788335
Armc6	706.78384	1.10729643	0.1470415	0.11438074	0.13574078	0.29014507
Armc7	70.9687508	-1.0232705	-0.0331876	0.17775545	0.74371854	0.85721023
Armc8	877.302319	1.04241705	0.05993258	0.07693218	0.40432934	0.60366653
Armc9	779.069539	1.0307798	0.04373617	0.10180969	0.6150889	0.77376891
Armcx1	992.675437	1.04048577	0.05725723	0.08022863	0.44203948	0.63932773
Armcx3	354.203574	1.0389574	0.0551365	0.10910588	0.55819027	0.73209966
Armcx4	2.14928527	-1.0338641	-0.0480465	0.22256042	0.03633461	0.10908048
Armcx5	402.358123	1.01453171	0.02081396	0.10456393	0.82087252	0.90344715
Armh1	2.84156437	1.04029956	0.05699903	0.22497978	0.05866608	0.15722019
Armh3	501.797442	1.07388088	0.10283397	0.08974614	0.20640945	0.3908507

Armt1	668.121641	-1.0658736	-0.0920363	0.08583192	0.24034733	0.42994683
Arnt	1768.99991	1.09574184	0.13190794	0.06321278	0.0286255	0.09020729
Arnt2	16.0134876	-4.9090116	-2.2954326	0.70483592	4.30E-05	0.00038236
Arntl	647.586087	-1.1741345	-0.2315976	0.12586708	0.02787204	0.08832086
Arntl2	85.41311	1.02087944	0.02981251	0.15299797	0.78315813	0.87878904
Arpc1a	2210.92478	1.0755704	0.10510196	0.06856471	0.10492671	0.24161623
Arpc2	7546.3241	1.02914736	0.04144957	0.06948446	0.5282641	0.70918708
Arpc3	2179.75459	1.03915866	0.05541595	0.05634522	0.30803777	0.5062173
Arpc4	1211.37348	-1.2027652	-0.2663551	0.08626688	0.0007888	0.00478501
Arpc5l	520.496011	1.07649088	0.1063361	0.12202528	0.29494761	0.49234804
Arpin	1308.51772	1.23657235	0.30634665	0.08036225	4.53E-05	0.00040112
Arpp21	2.68567522	-1.0543384	-0.076338	0.23516763	0.00226237	0.01156522
Arrb1	510.355134	1.18521856	0.24515313	0.10287705	0.00734347	0.03025728
Arrb2	372.177018	1.11796911	0.16088032	0.11921997	0.11070034	0.25107495
Arrdc1	385.021032	1.14584629	0.19641352	0.11374138	0.04439264	0.12730462
Arrdc2	167.868972	1.13232683	0.17929043	0.1747291	0.14168278	0.29939894
Arrdc3	228.559632	1.00030493	0.00043985	0.1386694	0.99787228	0.99951082
Arsa	1371.50069	1.74841915	0.80605109	0.10329171	5.02E-16	2.21E-14
Arsg	2314.37254	-13.953336	-3.8025381	0.08649673	0	0
Arsj	571.088265	-1.0329735	-0.0468033	0.10164074	0.6001185	0.76265059
Arsk	378.266929	1.08325022	0.11536654	0.11548595	0.23936028	0.42913999
Artn	40.9614904	-1.2337735	-0.3030776	0.34362406	0.04376691	0.12607469
Arv1	583.502196	1.00419868	0.00604473	0.09596985	0.94489095	0.97295716
Arvcf	141.392792	1.32050102	0.40108542	0.28626714	0.01833445	0.06392704
Arx	5.48818699	-1.0056643	-0.0081489	0.21102883	0.84800804	0.91825723
Asah1	3999.89232	1.19095059	0.25211357	0.04967549	1.79E-07	2.65E-06
Asap1	12202.1582	-1.1111946	-0.1521115	0.04405227	0.00041427	0.00275677
Asap2	1577.9681	-1.0308453	-0.0438278	0.06951644	0.50452091	0.68893646
Asap3	147.155217	1.32562855	0.40667657	0.19275792	0.00545564	0.0237387
Asb1	1623.78767	-1.0807014	-0.111968	0.07582463	0.11330698	0.25509006
Asb11	2.67751363	1.01939312	0.02771053	0.21656141	0.29052682	0.48706303
Asb13	453.2916	1.09607725	0.13234949	0.10149508	0.13982659	0.29625845
Asb15	99.1119603	-1.1305078	-0.1769709	0.21521734	0.15164241	0.31381895
Asb3	425.223629	1.00686037	0.00986363	0.09352878	0.90756666	0.95216453
Asb5	8.5769461	-1.0592637	-0.0830618	0.23219681	0.1212101	0.26756873
Asb6	281.645184	1.02498535	0.03560329	0.11628026	0.71719236	0.8413218
Asb7	981.088388	-1.0109549	-0.0157186	0.07855843	0.82897006	0.90788335
Asb8	730.848713	-1.043726	-0.061743	0.08467729	0.42507823	0.62345455
Ascc1	1281.747	1.01377442	0.01973666	0.07824959	0.78724941	0.88067985
Ascc2	1459.28452	-1.0070199	-0.0100923	0.05861605	0.85809263	0.92418115
Ascc3	5972.0473	1.04798531	0.06761849	0.064784	0.27440237	0.46993205
Ascl2	30.2107953	-1.1783739	-0.2367974	0.33217358	0.05736492	0.15457398
Asf1a	868.196262	-1.2021544	-0.2656222	0.09919235	0.00283957	0.01397959
Asf1b	2318.05539	-1.0224397	-0.0320158	0.07007139	0.62953342	0.78353451
Ash1l	8572.94904	1.15034383	0.20206514	0.05756154	0.00025411	0.0018232
Ash2l	2810.87515	1.16754597	0.22347935	0.06424577	0.00026309	0.00187545
Asic2	497.860578	1.17137618	0.22820446	0.10404333	0.01309432	0.04876113
Asl	91.7444222	1.08910202	0.1231391	0.16644698	0.2818955	0.47790913
Aspa	1.69615594	-1.026436	-0.0376437	0.21991323	0.03859386	0.11441502
Aspg	345.929037	-1.1676346	-0.2235888	0.13943105	0.04595462	0.1306392

Asph	2294.92818	-1.0924933	-0.1276245	0.08409539	0.09801509	0.22958004
Aspm	8711.32807	-1.1508246	-0.2026679	0.0545699	0.00011801	0.00094426
Aspscr1	1322.65409	1.35796811	0.4414496	0.06767783	1.32E-11	3.68E-10
Asrgl1	1051.7183	-1.0497118	-0.0699933	0.08185392	0.35390741	0.5534799
Aste1	313.165699	1.09986222	0.1373228	0.11339958	0.15662943	0.32154215
Astl	3.01274392	-1.0006623	-0.0009552	0.21284411	0.97466267	0.98847509
Asxl1	6686.16067	-1.018416	-0.0263269	0.04759128	0.56977255	0.73915016
Asxl2	3033.84702	-1.1038391	-0.1425299	0.06523051	0.02143987	0.07257157
Asxl3	2.13455115	-1.0338927	-0.0480864	0.22295293	0.0148931	0.05427612
Atad1	3672.06573	-1.0348235	-0.0493847	0.06171048	0.4036095	0.60336535
Atad2	29000.4076	-1.0868584	-0.120164	0.05473649	0.0228597	0.07631815
Atad2b	1352.15498	1.32948491	0.41086741	0.07721906	2.23E-08	3.86E-07
Atad3a	4341.75279	-1.3934424	-0.4786534	0.06907441	7.03E-13	2.30E-11
Atad5	1141.53727	1.07235609	0.10078404	0.07691023	0.15965733	0.32567047
Ate1	3568.00098	1.07635013	0.10614745	0.05053852	0.03048129	0.09500372
Atf1	4890.48393	-1.1007388	-0.1384722	0.05694874	0.01145428	0.04390463
Atf2	3214.21153	-1.0363715	-0.0515412	0.05545779	0.3354987	0.53527692
Atf3	61.473892	-1.0970111	-0.1335781	0.20364608	0.24528991	0.43562508
Atf4	8494.23406	1.0284339	0.04044908	0.09072153	0.62255177	0.77850311
Atf5	291.537129	1.10854936	0.14867302	0.14663839	0.18601041	0.3632307
Atf6	3506.34266	1.10527415	0.14440425	0.06438277	0.01841574	0.06419202
Atf6b	2573.38934	1.23242415	0.30149886	0.09685282	0.00059467	0.00376557
Atf7ip	3106.64871	1.04106497	0.05806011	0.06536606	0.35036196	0.55001675
Atg10	129.191913	1.14126188	0.19062988	0.18270193	0.12591147	0.27486809
Atg101	1100.74727	-1.1807427	-0.2396947	0.08957697	0.00332972	0.01592744
Atg12	36.5334931	1.06037925	0.08458035	0.19851549	0.40528673	0.60467307
Atg13	1526.97874	-1.0360029	-0.051028	0.06716118	0.42280082	0.62139496
Atg14	315.938765	1.21702529	0.28335914	0.1312681	0.01010682	0.03961701
Atg16l1	845.838514	1.04055883	0.05735853	0.08296229	0.44855162	0.6442366
Atg16l2	229.9887	1.14249345	0.1921859	0.15227413	0.09982483	0.23271265
Atg2a	1091.09336	-1.0690831	-0.0963739	0.07925245	0.18926463	0.36759611
Atg2b	2637.07948	1.03065531	0.04356192	0.05675909	0.44292697	0.64003536
Atg3	2174.76859	1.06014875	0.08426671	0.06889179	0.19648471	0.37705918
Atg4b	969.461577	1.05759675	0.08078965	0.07634104	0.25716956	0.44937145
Atg4c	940.314447	1.04019955	0.05686031	0.0782382	0.43521215	0.63288315
Atg4d	166.931305	1.10728651	0.14702857	0.16000818	0.20590804	0.39027321
Atg5	1794.56725	1.08459005	0.11714984	0.06823264	0.06963899	0.17862946
Atg7	503.38794	1.09991356	0.13739015	0.10101673	0.12332984	0.27131295
Atg9a	1268.48822	1.06858247	0.09569825	0.08818449	0.23212912	0.42134853
Atg9b	110.003498	-1.7362238	-0.7959529	0.29418478	0.00040318	0.002697
Atic	9499.04543	-1.1103049	-0.1509559	0.05323277	0.00331864	0.01590413
Atl1	88.6392822	1.07620104	0.10594761	0.19338969	0.33723206	0.53697237
Atl2	1531.44685	-1.1384901	-0.1871217	0.06026325	0.00117846	0.00667401
Atl3	7125.88416	-1.0363386	-0.0514955	0.05340787	0.31769952	0.51645699
Atm	8610.92287	1.14947658	0.20097707	0.07061494	0.00256891	0.012905
Atoh8	300.442591	1.1606197	0.21489532	0.17349129	0.0841454	0.2055889
Atox1	195.308104	1.09080117	0.12538815	0.15480238	0.27047537	0.46572403
Atp10a	9536.09562	-1.1160497	-0.1584012	0.04516963	0.00032259	0.00221934
Atp11a	3619.03428	-1.0681483	-0.095112	0.05346204	0.06577704	0.17171468
Atp11b	3360.64551	-1.0012439	-0.0017934	0.06128151	0.97589317	0.98894008

Atp11c	1183.1249	-1.0265776	-0.0378427	0.07669381	0.59665164	0.75952575
Atp12a	6.30705381	-1.0268546	-0.038232	0.21706616	0.30784846	0.50610426
Atp13a1	1159.5031	-1.0206002	-0.0294178	0.06895213	0.65172169	0.79791215
Atp13a2	692.379822	1.12812409	0.17392577	0.12160842	0.08761995	0.2122965
Atp13a3	13512.4444	-1.4094363	-0.4951183	0.05729026	9.15E-19	5.01E-17
Atp1a1	8686.95136	-1.0432299	-0.0610572	0.05494941	0.24396109	0.43422836
Atp1b1	11.2742647	-1.0179503	-0.0256672	0.21010609	0.63601444	0.7880206
Atp1b3	4743.41059	-1.0928233	-0.1280601	0.05331385	0.00725585	0.02995406
Atp23	656.334547	-1.0753841	-0.104852	0.08087801	0.15935346	0.32562097
Atp2a1	11.0673363	-1.047255	-0.0666128	0.2186832	0.3112399	0.50973011
Atp2a2	13250.9441	-1.0464883	-0.0655562	0.04296731	0.12041748	0.26628282
Atp2b1	7852.57974	-1.2734408	-0.3487319	0.05809636	5.41E-10	1.19E-08
Atp2b4	2516.0644	1.35929363	0.44285714	0.08490959	3.38E-08	5.66E-07
Atp2c1	2597.80504	-1.0111165	-0.0159492	0.05725535	0.79602666	0.8865503
Atp2c2	4.28805834	-1.0266078	-0.0378851	0.217356	0.29179687	0.4887445
Atp5a1	6316.16296	-1.0728205	-0.1014088	0.04469264	0.02013585	0.06899902
Atp5b	20455.969	-1.0342291	-0.0485559	0.04411327	0.26001989	0.45216457
Atp5c1	7066.45837	-1.0285629	-0.04063	0.04697085	0.36828816	0.5699168
Atp5d	611.046097	1.03868339	0.05475596	0.11662017	0.57590541	0.74338456
Atp5e	986.103543	1.36113469	0.44480983	0.13860943	0.00021952	0.00162186
Atp5g3	3585.95572	-1.174594	-0.2321621	0.06812799	0.00032014	0.00220487
Atp5j	4177.87513	-1.0862051	-0.1192965	0.0836366	0.1177481	0.2621855
Atp5j2	1080.57796	-1.2619466	-0.3356509	0.10254315	0.00029303	0.00204782
Atp5k	1527.03565	-1.2484137	-0.3200961	0.07972455	1.82E-05	0.00017622
Atp5mpl	787.403193	1.07310356	0.1017893	0.08577793	0.19489481	0.37480896
Atp5o	4830.84304	-1.0407463	-0.0576184	0.04675775	0.20604312	0.39038787
Atp5pb	5623.89712	-1.0993311	-0.1366259	0.05439963	0.00920223	0.03649555
Atp6ap1	1516.39705	1.29052064	0.36795321	0.08021898	1.12E-06	1.43E-05
Atp6ap2	1458.13901	1.14873863	0.20005059	0.08613836	0.01120243	0.0430811
Atp6v0a1	1452.12138	1.05412404	0.07604464	0.06069136	0.1907548	0.36960782
Atp6v0a2	1629.07161	1.06210857	0.08693124	0.06067097	0.13494568	0.28897595
Atp6v0b	222.68656	-1.1066283	-0.1461707	0.16464154	0.21134782	0.39685805
Atp6v0c	698.706097	1.10533422	0.14448266	0.09851619	0.09893957	0.23111158
Atp6v0d1	1099.98564	1.2736997	0.34902517	0.06971018	1.55E-07	2.31E-06
Atp6v0e	126.04954	1.20927604	0.27414361	0.17158207	0.03256673	0.10018785
Atp6v1a	3163.46961	1.02845497	0.04047862	0.06089559	0.48862134	0.67693697
Atp6v1b2	1493.89429	1.07678169	0.10672579	0.06363699	0.07852936	0.19534763
Atp6v1c1	1330.90322	1.14509162	0.19546303	0.07726644	0.00662095	0.0277171
Atp6v1e1	1990.66066	1.0012877	0.00185657	0.06304239	0.97543623	0.98867578
Atp6v1e2	7.68207803	1.09624263	0.13256714	0.2724092	0.01909895	0.06599879
Atp6v1fnb	13.3265716	1.02202531	0.03143092	0.20474216	0.66109786	0.80385712
Atp6v1g1	1556.97115	1.00217124	0.00312904	0.0985435	0.97253453	0.98767978
Atp6v1g2	62.469119	1.07214962	0.10050625	0.20331216	0.33922575	0.53880743
Atp6v1h	814.582048	1.05882186	0.08245988	0.07595427	0.24509963	0.43540789
Atp7a	2045.9592	-1.05845	-0.0819531	0.0632912	0.17435111	0.34756268
Atp7b	550.732894	-1.1593068	-0.2132624	0.1017567	0.0179671	0.06311852
Atp8a1	572.445594	-1.0440379	-0.0621741	0.09131299	0.45086647	0.64618934
Atp8b2	3069.27565	-1.1713623	-0.2281873	0.07023563	0.00057435	0.00366591
Atp9b	570.212647	1.12277285	0.16706608	0.12077744	0.09914358	0.2315459
Atpaf1	3031.25672	1.03805298	0.05388008	0.0535029	0.29860484	0.49638308

Atpaf2	621.94443	-1.0297681	-0.0423195	0.0871812	0.59500271	0.75833099
Atpif1	339.026485	-1.3406912	-0.422977	0.1363513	0.00033243	0.002276
Atpsckmt	151.774435	1.20404121	0.26788477	0.17789648	0.03948175	0.116508
Atr	2716.18631	-1.0200647	-0.0286607	0.06216411	0.63214243	0.78541342
Atraid	219.668956	1.03228123	0.04583607	0.16412062	0.67276968	0.81086907
Atrip	302.650358	1.10710079	0.14678657	0.11915208	0.14476471	0.30405574
Atrn	2357.48556	1.19512522	0.25716178	0.05811613	4.34E-06	4.87E-05
Atrnl1	644.060343	-1.1149671	-0.1570011	0.08396965	0.04145556	0.12088759
Atrx	9553.9774	1.01466046	0.02099704	0.0675502	0.74405052	0.85736145
Atxn1	136.340104	-1.0497492	-0.0700447	0.15328304	0.52332228	0.70536179
Atxn10	3211.3724	-1.0115329	-0.0165433	0.05862546	0.76869444	0.87144584
Atxn1l	938.269338	-1.023343	-0.0332898	0.08288079	0.66249329	0.80471736
Atxn2	2509.49858	-1.0346091	-0.0490858	0.0499929	0.31238468	0.511262
Atxn2l	10139.0372	1.0552164	0.07753889	0.05233352	0.126619	0.27589547
Atxn3	1079.75259	-1.0514629	-0.072398	0.08117754	0.33367193	0.53357282
Atxn7	894.171745	1.09315401	0.12849667	0.079042	0.07988875	0.19775664
Atxn7l1	26.2239456	1.00358923	0.00516889	0.21143134	0.90176306	0.94918528
Atxn7l2	361.712553	-1.0711862	-0.0992092	0.11381663	0.30529408	0.50345227
Atxn7l3	1260.0491	1.06588547	0.09205242	0.06812885	0.15380618	0.31676317
Atxn7l3b	161.381433	-1.0374276	-0.0530106	0.14154512	0.6193345	0.77644108
Auh	152.64102	1.06789962	0.09477604	0.14871078	0.39183942	0.5931769
Aunip	749.336174	-1.0461966	-0.065154	0.09248729	0.43532401	0.63295591
Aup1	607.846603	-2.2362985	-1.1611128	0.1267923	3.46E-21	2.23E-19
Aurka	2882.55505	1.17537871	0.23312567	0.06774131	0.00028689	0.00201482
Aurkaip1	2158.57112	-1.1458618	-0.196433	0.09223497	0.01816122	0.0635122
Aurkb	594.136607	1.03934481	0.05567435	0.10332322	0.540116	0.71845252
Aurkc	746.404416	1.33616055	0.41809337	0.08370964	1.22E-07	1.86E-06
Aven	1015.29569	-1.024851	-0.0354141	0.08731039	0.656533	0.80089399
Avl9	869.615531	-2.1499619	-1.1043111	0.08118454	2.93E-43	5.51E-41
Avpi1	188.229841	-1.0223897	-0.0319452	0.15029542	0.76533539	0.87006978
Axdnd1	40.8216858	-1.0357499	-0.0506756	0.18471068	0.6154439	0.77387521
Axin1	2411.38315	-1.0266043	-0.0378802	0.05471978	0.4734421	0.66433264
Axin2	357.329293	1.51397734	0.59834361	0.1330884	7.54E-07	9.94E-06
Axl	13479.5683	-1.0576933	-0.0809213	0.04304099	0.05637586	0.15238969
Azi2	2228.87026	-1.0877784	-0.1213847	0.06395558	0.04590226	0.13055099
Azin1	7027.25222	1.19714566	0.2595987	0.05337821	5.18E-07	7.02E-06
B230118H07Rik	879.86069	1.08018648	0.1112804	0.07678028	0.12016508	0.2660267
B230219D22Rik	763.240325	1.02794709	0.039766	0.09879237	0.65754468	0.80140161
B2M	992.801903	-1.0905038	-0.1249947	0.18796038	0.27669405	0.4723077
B3GAT3	251.900665	1.13357462	0.18087936	0.13690319	0.09872982	0.23083393
B3galnt1	334.847397	1.03182784	0.04520228	0.12403606	0.65499148	0.80008018
B3galnt2	438.560424	-1.0260967	-0.0371667	0.10386537	0.68202046	0.81689408
B3galt1	37.1241541	1.01122207	0.01609985	0.19011971	0.86015716	0.92515644
B3galt4	69.5507279	1.12392242	0.16854246	0.2353983	0.15324848	0.31618941
B3galt6	136.399469	1.10646746	0.14596102	0.17189127	0.21882303	0.40578234
B3glct	1044.97544	1.22472247	0.29245486	0.09385746	0.00064128	0.0040092
B3gnt2	1364.08893	-1.2242246	-0.2918683	0.11412357	0.00345985	0.01642114
B3gnt3	4.14377198	-1.0732594	-0.1019988	0.25233439	0.00027665	0.00195579
B3gnt4	2.20167444	1.033785	0.04793618	0.22105045	0.1454691	0.30503506
B3gnt9	56.4698651	1.00563228	0.00810286	0.18842419	0.93131757	0.96584991

B3gntl1	301.050926	1.26773201	0.3422498	0.13383452	0.00255163	0.0128333
B4GALT3	468.334257	-1.0186613	-0.0266744	0.11095221	0.7810307	0.87747792
B4GALT4	544.426076	-1.0260645	-0.0371215	0.08832626	0.64473436	0.79314771
B4galt1	1069.8002	-1.1004573	-0.1381032	0.07196652	0.04088525	0.11968464
B4galt2	536.687611	1.27078128	0.34571574	0.11583195	0.00071947	0.004414
B4galt5	1577.79686	-1.1277615	-0.173462	0.07457529	0.01282121	0.0480233
B4galt6	328.918238	-1.0881444	-0.1218701	0.10976721	0.19714872	0.37797114
B4galt7	408.957679	1.07690332	0.10688873	0.10981437	0.2582975	0.45056774
B630019K06Rik	7.56809931	1.04202913	0.05939562	0.21794915	0.321584	0.52053209
B9d1	85.5522575	1.00334434	0.00481682	0.15604312	0.96559236	0.98312856
B9d2	299.003704	1.18345134	0.24300038	0.12440994	0.02073143	0.07061144
BAIAP2	4277.56634	-1.0201591	-0.0287942	0.05034917	0.60109017	0.76335532
BC003965	175.137303	-1.1504594	-0.2022101	0.16762355	0.09712342	0.22809284
BC004004	1462.52738	1.09975829	0.13718647	0.06894818	0.03532418	0.10667203
BC005537	1269.00414	-1.3276028	-0.4088236	0.08774099	6.46E-07	8.63E-06
BC005624	2236.25154	1.15408577	0.20675045	0.0598917	0.0003173	0.00218765
BC017158	578.266805	-1.6952984	-0.7615392	0.12525293	1.01E-10	2.52E-09
BC024978	45.37992	-1.0546374	-0.0767471	0.19812715	0.43618815	0.63379879
BC031181	71.2204301	1.01521476	0.02178495	0.16988649	0.83672243	0.91236077
BC034090	84.2009489	1.16251892	0.2172542	0.24797545	0.09861588	0.23067264
BC048403	946.235341	-1.0760321	-0.1057211	0.07892559	0.14767311	0.30819363
BC052040	226.385758	1.0830991	0.11516525	0.1260832	0.26554858	0.45943778
BC055324	1094.29648	1.25917116	0.33247441	0.06928761	4.85E-07	6.60E-06
BCL2	121.257012	-1.0691862	-0.0965132	0.17649091	0.38873481	0.59043074
BOP1	2232.70004	-1.0631546	-0.0883514	0.05786974	0.11215679	0.25330611
Baat	13.6936692	1.00453176	0.00652318	0.20030669	0.93141476	0.96587251
Babam1	976.382239	1.1396269	0.18856158	0.09517266	0.02773765	0.08808114
Babam2	1442.40789	-1.0222388	-0.0317322	0.06672378	0.61618626	0.77428768
Bace1	893.754978	1.51747708	0.60167472	0.08727953	6.29E-13	2.07E-11
Bach1	2074.23799	-1.2453857	-0.3165926	0.06007252	4.43E-08	7.32E-07
Bag1	853.571887	-1.0984153	-0.1354237	0.10029385	0.12683694	0.27618242
Bag2	1054.8493	-1.0348642	-0.0494415	0.07103323	0.46009552	0.65421507
Bag3	3002.74824	-1.0891022	-0.1231394	0.05570672	0.02172653	0.07331845
Bag4	693.085932	-1.0714902	-0.0996186	0.08330815	0.19166738	0.37049331
Bag5	2866.25234	-1.0359759	-0.0509904	0.05870733	0.36598081	0.56716842
Bag6	8269.99492	1.03205682	0.04552241	0.05555887	0.39639988	0.5968362
Bahcc1	3588.84793	-1.1939051	-0.2556881	0.07402756	0.00023686	0.00172158
Baiap2l1	1261.53	-1.0789293	-0.1096004	0.07386275	0.11288794	0.25453854
Baiap3	4.1364945	-1.0271851	-0.0386962	0.21801834	0.24826836	0.43923403
Bak1	1160.33357	-1.052656	-0.074034	0.08909526	0.35958733	0.55983395
Bambi	119.294106	-1.2316617	-0.300606	0.19708129	0.02983801	0.09331663
Banp	709.09391	-1.184239	-0.2439602	0.08986102	0.00284913	0.01401466
Bap1	4220.1455	1.07557382	0.10510655	0.05597529	0.05130214	0.14172535
Bard1	1691.9439	1.08930209	0.12340411	0.06546388	0.04732863	0.13352465
Basp1	1443.0746	-1.0340718	-0.0483364	0.11168144	0.61187546	0.77162085
Batf	240.369148	1.08667631	0.11992226	0.13691356	0.26785268	0.4623003
Batf2	56.1626681	-1.1327573	-0.1798388	0.24759422	0.13156381	0.28351855
Bax	666.339878	1.07570514	0.10528267	0.08824163	0.1901198	0.3688376
Baz1a	6681.01014	-1.0939845	-0.1295923	0.0521562	0.01019026	0.03987086
Baz1b	10083.8223	-1.0464359	-0.065484	0.05017721	0.17909658	0.3542114

Baz2a	5165.54069	-1.1660172	-0.2215891	0.05306542	1.58E-05	0.00015619
Baz2b	1933.58488	-1.0394163	-0.0557735	0.07307603	0.41647932	0.61542278
Bbc3	94.5605476	1.04966753	0.06993245	0.15626509	0.52809387	0.70910718
Bbof1	292.415157	1.34260675	0.42503681	0.15111663	0.00083819	0.00503622
Bbs1	341.841873	1.39763821	0.48299095	0.12325475	1.35E-05	0.00013594
Bbs10	233.709976	-1.0442351	-0.0624466	0.14528411	0.56409604	0.73589476
Bbs12	346.803344	-1.0624091	-0.0873394	0.13575877	0.41161015	0.61088622
Bbs4	422.499688	1.19612805	0.25837184	0.12114919	0.01252595	0.04708257
Bbs7	1110.06846	1.12638362	0.17169826	0.09120342	0.03763464	0.11227287
Bbs9	913.669709	1.17074449	0.22742624	0.08149768	0.002586	0.01298068
Bbx	5870.06038	-1.1309987	-0.1775973	0.05036932	0.00027853	0.00196641
Bcap29	3176.86965	1.00095205	0.00137286	0.07307431	0.98418471	0.99161602
Bcar1	2722.03893	-1.0158712	-0.0227176	0.05985931	0.69176764	0.82348698
Bcar3	1747.11713	-1.272993	-0.3482245	0.07801133	2.21E-06	2.66E-05
Bcas3	506.784815	1.2247431	0.29247916	0.10127981	0.00131188	0.00727183
Bcat1	2142.19426	1.09967703	0.13707987	0.08059329	0.06589468	0.17195168
Bccip	2118.21977	-1.0574515	-0.0805915	0.08110856	0.28202915	0.47803212
Bcdin3d	711.158069	-1.0292512	-0.0415952	0.07768709	0.56486392	0.73640411
Bckdha	484.188272	1.02439269	0.03476886	0.12561304	0.73704103	0.85316946
Bckdhb	568.344636	-1.0298276	-0.0424028	0.09371031	0.61435394	0.77329999
Bcl10	1875.88748	-1.2571663	-0.3301755	0.07693727	5.27E-06	5.77E-05
Bcl2l1	1499.85153	-1.0341051	-0.0483828	0.0620375	0.41447637	0.6132412
Bcl2l11	531.366024	-1.0855939	-0.1184845	0.09784138	0.17240962	0.34471161
Bcl2l12	279.345963	1.03028065	0.04303738	0.13507475	0.68330241	0.8177414
Bcl2l13	1522.32463	-1.1154527	-0.1576293	0.06468565	0.01030925	0.04023813
Bcl2l2	34.7373469	-1.015261	-0.0218506	0.19091889	0.80708715	0.89404933
Bcl6	302.018556	-1.3973613	-0.4827051	0.13299509	4.12E-05	0.00036847
Bcl6b	9.3334777	-1.0362624	-0.0513893	0.21890321	0.27949169	0.47521749
Bcl7a	218.770482	1.07814432	0.10855031	0.13018785	0.30206665	0.49986743
Bcl7b	376.843166	-1.01109	-0.0159115	0.10716891	0.86325471	0.92665838
Bcl7c	588.421594	-1.0198866	-0.0284088	0.10691286	0.7577982	0.86561668
Bcl9	1774.22028	1.00542034	0.00779878	0.06666634	0.90273894	0.94945197
Bcl9l	3480.28894	1.26070776	0.33423389	0.08482251	2.35E-05	0.00022004
Bclaf3	1186.46497	1.03755981	0.0531945	0.07973503	0.47299719	0.66421861
Bco1	5.10650239	1.0198401	0.02834297	0.21390673	0.49491504	0.68171472
Bco2	247.583116	1.23812768	0.3081601	0.14509184	0.00938373	0.03708996
Bcor	1192.94562	-1.0088159	-0.012663	0.07965957	0.86318403	0.92665838
Bcorl1	1496.55585	-1.0498886	-0.0702362	0.07721683	0.32834154	0.52808662
Bcr	2979.98173	1.02870992	0.04083622	0.05567915	0.44886923	0.644476
Bcs1l	476.25021	1.0713008	0.09936362	0.10531677	0.27947113	0.47521749
Bdh1	1004.59758	-1.0440798	-0.062232	0.06765335	0.33184663	0.53172095
Bdh2	216.674996	1.38271934	0.46750835	0.1788515	0.00119066	0.00671908
Bdnf	1295.47876	1.54378623	0.62647299	0.09367062	2.54E-12	7.79E-11
Bdp1	9979.08638	-1.2127123	-0.2782373	0.05447518	1.21E-07	1.84E-06
Bean1	8.46039762	-14.409396	-3.848938	1.19201038	9.13E-05	0.00074798
Becn1	3256.61079	-1.1325051	-0.1795175	0.05905749	0.0015196	0.00824835
Bend3	1754.45385	-1.334275	-0.4160561	0.06028747	1.08E-12	3.44E-11
Bend4	1059.95032	-1.0773609	-0.1075017	0.06800534	0.09307246	0.22128028
Bend6	691.952911	1.06739172	0.09408972	0.09260538	0.26044092	0.45274097
Bet1	394.771982	-1.0699341	-0.0975219	0.11347976	0.31322971	0.51207488

Bex3	953.3346	-1.125415	-0.1704571	0.10717331	0.0676665	0.17518348
Bfar	379.803014	-1.1409569	-0.1902443	0.12891775	0.0723973	0.18371819
Bfsp1	2.69507248	-1.0279856	-0.0398201	0.21955268	0.12140649	0.26782782
Bhlhb9	1311.09498	-1.0578823	-0.0811792	0.07667792	0.2560444	0.44813891
Bhlhe40	422.440849	-1.4629609	-0.5488912	0.1308017	3.31E-06	3.81E-05
Bhlhe41	1128.20824	-1.3049438	-0.3839877	0.07296274	3.41E-08	5.68E-07
Bicd1	180.33345	1.16577261	0.22128641	0.16253308	0.06908707	0.17774761
Bicd2	3765.78392	1.06298995	0.08812796	0.04520342	0.04598492	0.13064247
Bicdl1	1.81579735	1.01122297	0.01610115	0.21490018	0.52545508	0.70689766
Bicra	182.366042	1.14354398	0.19351186	0.17350879	0.11496969	0.25790472
Bicral	1661.29905	1.08131276	0.11278387	0.06159807	0.05537339	0.15021879
Bid	385.678441	-1.3537077	-0.4369163	0.12357431	6.98E-05	0.00059124
Bik	2.56775587	-1.0146426	-0.0209716	0.21543528	0.41531686	0.61434284
Bin1	846.736184	-1.0613179	-0.0858568	0.08573234	0.27343372	0.46917449
Bin3	358.423528	1.02249579	0.0320949	0.11845917	0.74710149	0.8595626
Birc2	274.326652	-1.0419325	-0.0592618	0.13110656	0.57011346	0.73941008
Birc3	909.960877	-1.9190961	-0.9404269	0.08624905	8.12E-29	8.13E-27
Birc6	11915.629	-1.0266505	-0.0379451	0.04442931	0.38229031	0.58397551
Blcap	60.2948391	-1.0264164	-0.0376161	0.18636469	0.69643446	0.8265844
Blm	3084.97594	1.10083117	0.13859322	0.05104811	0.00509058	0.02246306
Blmh	2875.30276	-1.1093536	-0.1497192	0.05889638	0.00796371	0.03235492
Blnk	335.64465	-1.4302216	-0.5162387	0.1437209	4.17E-05	0.00037235
Bloc1s2	981.527216	1.08057777	0.11180291	0.08497011	0.15159373	0.31376889
Bloc1s3	3.33777431	-1.0464043	-0.0654404	0.22697734	0.09113978	0.21814285
Bloc1s4	211.666069	-1.0425685	-0.0601422	0.11919521	0.54535133	0.72288395
Bloc1s6	499.678605	-1.026724	-0.0380484	0.10698506	0.68168749	0.81680074
Blvra	887.365412	1.1525699	0.20485425	0.10379328	0.0254183	0.08264271
Blvrb	1337.00868	-1.2363095	-0.30604	0.08670053	0.00013273	0.00104187
Blzf1	1425.19189	1.0919634	0.1269245	0.06284712	0.03417845	0.10416986
Bmerb1	2360.81856	-1.0262997	-0.0374521	0.05752051	0.49939874	0.68537582
Bmf	8.78717148	1.06341061	0.08869877	0.23754951	0.06435831	0.16904449
Bmi1	2333.10496	-1.0867495	-0.1200195	0.05695927	0.02840647	0.08956603
Bmp1	1528.80738	-1.335886	-0.4177969	0.08365702	1.17E-07	1.79E-06
Bmp2k	2834.96158	-1.0657129	-0.0918188	0.05599487	0.08866829	0.2139803
Bmp5	1.84728751	-1.0287634	-0.0409112	0.22078305	0.02819689	0.08916371
Bmp6	4.30898268	-1.0342988	-0.0486531	0.22113195	0.14619075	0.30594731
Bmper	16.4558901	1.05272211	0.07412466	0.22156166	0.27344886	0.46917449
Bmpr1a	1342.07432	1.01566643	0.02242667	0.07757187	0.75729082	0.86530153
Bmpr1b	583.660231	-1.0278331	-0.0396059	0.08605184	0.61457382	0.77342476
Bmpr2	3422.53342	1.1671671	0.22301112	0.07834901	0.00230076	0.0117407
Bms1	15867.9008	-1.0459872	-0.0648651	0.0534724	0.20336804	0.38669209
Bmt2	1201.25218	-1.0951219	-0.1310915	0.08152973	0.08089731	0.19959447
Bnc2	1557.93156	-1.2434958	-0.3144017	0.07664593	1.30E-05	0.00013098
Bnip1	845.30537	1.11380037	0.15549068	0.09292327	0.06296563	0.16606747
Bnip2	2198.35911	-1.0267553	-0.0380924	0.05632476	0.48312224	0.67255597
Bnip3	771.740024	-1.5027612	-0.5876158	0.08581392	9.12E-13	2.94E-11
Bnip3l	3605.79257	-1.241288	-0.3118379	0.05265389	1.07E-09	2.24E-08
Bnipl	6.26324774	3.59784001	1.84713104	1.43538658	0.00496033	0.02199442
Boc	609.582054	-1.0703336	-0.0980605	0.08357253	0.20110255	0.38362368
Bod1l	9391.34256	-1.0980228	-0.134908	0.07066059	0.04189411	0.12186581

Bola1	100.165598	-1.0338416	-0.0480152	0.16255077	0.65766063	0.80140161
Bola2	57.4890578	-1.063242	-0.08847	0.20151329	0.37972993	0.58189178
Bola3	112.070655	-1.3031152	-0.3819647	0.20890218	0.01110753	0.04275464
Bora	970.980548	-1.1224117	-0.1666019	0.08503144	0.03229432	0.09946869
Borcs5	202.643373	1.08603835	0.11907504	0.14120083	0.2765228	0.47217503
Borcs6	13.2636232	-1.0406187	-0.0574416	0.21155113	0.42638394	0.62465442
Borcs7	191.908218	1.00354954	0.00511184	0.13925316	0.96282443	0.98253652
Borcs8	239.702831	1.25088266	0.32294647	0.14510568	0.00670266	0.02798947
Bpgm	787.776004	-1.1136257	-0.1552644	0.08484989	0.04581408	0.13038845
Bphl	349.449633	1.23341568	0.3026591	0.13800442	0.00808525	0.03275201
Bpnt1	389.210888	-1.0566981	-0.0795633	0.10677576	0.38990879	0.59158256
Bptf	7380.97995	-1.0709051	-0.0988307	0.04401405	0.02118004	0.07185283
Braf	1775.34264	1.18378206	0.24340349	0.06506937	8.78E-05	0.00072574
Brap	1810.92007	1.09114551	0.12584351	0.06645824	0.04575209	0.13029885
Brat1	401.459273	1.06730155	0.09396784	0.13777806	0.38130891	0.5832319
Brca1	13258.7632	-1.1884642	-0.2490985	0.04146518	9.11E-10	1.94E-08
Brca2	6198.38359	1.09003669	0.12437669	0.05386919	0.01687703	0.05980491
Brcc3	277.97899	-1.326347	-0.4074582	0.16552871	0.00232323	0.01183325
Brd1	1700.27486	-1.0401145	-0.0567424	0.06982179	0.38935118	0.59099561
Brd2	11378.4839	-1.2563352	-0.3292215	0.05515199	6.87E-10	1.49E-08
Brd3	3614.45865	1.05829621	0.08174348	0.04647226	0.07107713	0.18108552
Brd4	8518.46734	-1.1188821	-0.162058	0.04550826	0.00026014	0.00185749
Brd7	5617.86068	-1.0091405	-0.013127	0.05927645	0.81720609	0.90128368
Brd8	715.863418	1.29110241	0.36860344	0.12695505	0.00083633	0.00502738
Brd9	1438.88999	1.09818849	0.1351257	0.07783028	0.06184701	0.16385939
Brdt	91.3539693	-1.0272913	-0.0388454	0.1663728	0.71726986	0.84133568
Brf1	1622.04788	1.04892293	0.06890868	0.07179504	0.30858355	0.50685412
Brf2	321.568603	1.00150196	0.00216524	0.10563542	0.98209172	0.99108517
Bri3	48.6789122	1.00314076	0.00452406	0.17818283	0.96562732	0.98312856
Bri3bp	2144.07587	-1.0813832	-0.1128778	0.07193037	0.09490604	0.22459751
Bricd5	4.99777988	-1.0102397	-0.0146976	0.21257685	0.70166617	0.83028433
Brinp3	17.4312025	-1.0943877	-0.130124	0.25177623	0.11830352	0.26303095
Brip1	1820.41142	1.01204691	0.01727617	0.06346861	0.77626228	0.875268
Brix1	2982.8879	-1.2657128	-0.3399501	0.07671649	2.67E-06	3.14E-05
Brk1	389.586492	-1.0078698	-0.0113093	0.13892717	0.91400867	0.95543627
Brms1	1066.67272	1.02606941	0.03712833	0.08124656	0.6220798	0.77824473
Brms1l	766.70524	1.02438874	0.0347633	0.09531467	0.68492519	0.81904412
Brox	3958.89157	1.01187694	0.01703384	0.06242985	0.77607025	0.87520555
Brpf1	1771.60436	-1.0724486	-0.1009086	0.06969838	0.12498545	0.27385079
Brpf3	2301.14901	1.03435933	0.04873745	0.06320545	0.41963253	0.61830252
Brwd1	2375.98524	-1.1156551	-0.1578911	0.06365243	0.00918615	0.03645434
Brwd3	3401.19564	1.01207066	0.01731002	0.06061334	0.76564113	0.87025264
Bscl2	58.4483762	1.08757248	0.12111155	0.20217126	0.27824181	0.47403611
Bsdc1	788.155461	1.06659888	0.09301772	0.08430976	0.22879369	0.41748237
Bsg	3610.38236	-1.0355061	-0.0503361	0.05429615	0.33718183	0.53697237
Bsn	2.6261226	1.02174118	0.03102978	0.2180513	0.1286639	0.27935331
Bst2	2885.46375	-1.2194348	-0.2862126	0.06875227	1.14E-05	0.00011679
Btbd1	1557.02106	1.19505792	0.25708054	0.08746192	0.00137147	0.0075531
Btbd18	5.12926992	-1.0218107	-0.0311279	0.21451579	0.45737679	0.65165128
Btbd19	80.5540172	-1.1504863	-0.2022438	0.21964707	0.1166226	0.26047865

Btbd2	339.368651	1.2360362	0.30572099	0.16215201	0.0153051	0.05539936
Btbd3	2341.35642	1.08120713	0.11264293	0.07421509	0.10487382	0.24153787
Btbd6	1129.78038	1.19559699	0.25773117	0.08824713	0.00144028	0.00786904
Btbd7	3768.64448	-1.013073	-0.0187381	0.04743039	0.6849664	0.81904412
Btbd9	499.621135	1.51036621	0.59489839	0.11584343	3.16E-08	5.34E-07
Btd	205.491931	1.0105215	0.01510001	0.12114778	0.88123398	0.93654552
Btf3	6129.36616	-1.1788173	-0.2373401	0.06649276	0.00017221	0.00131388
Btg1	3268.35561	-1.2047133	-0.2686899	0.05949213	2.60E-06	3.09E-05
Btg2	102.67255	-1.024739	-0.0352564	0.18038787	0.72543119	0.84533553
Btg3	890.210239	1.05716608	0.08020204	0.09367747	0.34153742	0.54117828
Btg4	2.5605807	1.01816192	0.02596701	0.21596436	0.35759063	0.55772996
Btrc	641.064765	1.09736943	0.13404929	0.08610932	0.08859872	0.21385898
Bub1	2853.90365	1.0405759	0.0573822	0.05582424	0.2864632	0.48296836
Bub1b	8227.81794	1.00599539	0.00862369	0.04728286	0.85700669	0.92378456
Bub3	3755.58381	-1.1186461	-0.1617537	0.08265223	0.03457144	0.10501783
Bud13	1897.77559	-1.0160685	-0.0229976	0.06539879	0.71163079	0.83709738
Bud23	1712.26093	1.12360763	0.16813832	0.05844674	0.00273577	0.01358341
Bud31	2218.62644	-1.045839	-0.0646608	0.07958558	0.38100983	0.58291365
Bzw1	6002.76075	-1.2146595	-0.2805519	0.0611215	1.72E-06	2.12E-05
C1d	2346.83997	-1.0170551	-0.0243978	0.09159723	0.76568226	0.87025264
C1galt1c1	931.881475	1.09467301	0.13049999	0.08145632	0.08272134	0.20280581
C1qbp	3117.87043	-1.1854335	-0.2454147	0.06587173	9.02E-05	0.00074144
C1ql3	6.61532205	1.01871834	0.02675523	0.21215267	0.57462443	0.74232947
C1qtnf1	108.253016	-1.2838633	-0.3604916	0.2564308	0.02330666	0.07744704
C1qtnf12	3.84360669	1.00916549	0.01316278	0.21226953	0.74343127	0.85695623
C1qtnf2	35.5494924	1.05707416	0.08007659	0.20298178	0.40704944	0.60643842
C1qtnf5	177.046085	1.20179846	0.26519498	0.22374321	0.05803937	0.15589943
C1qtnf6	1.83881061	-1.0023475	-0.0033827	0.21434305	0.8739603	0.93213247
C1rl	45.5173922	-1.1161484	-0.1585288	0.23772075	0.16066154	0.32693793
C2cd2	1117.64929	-1.2049491	-0.2689722	0.08030967	0.00031915	0.00219917
C2cd2l	485.297211	-1.0625791	-0.0875702	0.102608	0.32951295	0.52941941
C2cd3	3864.62486	-1.0426186	-0.0602115	0.05154341	0.22872193	0.41748237
C2cd5	2302.35659	-1.0837234	-0.1159966	0.0750994	0.09797935	0.22953831
C3	2.55129559	1.0361079	0.05117426	0.22170061	0.14900483	0.30990345
C330007P06Rik	677.146949	-1.1551762	-0.2081129	0.09954708	0.01866139	0.0647663
C330018D20Rik	172.771975	1.03560095	0.0504682	0.12713554	0.62442866	0.78000113
C3ar1	293.701473	-1.2782481	-0.3541679	0.14527907	0.00310167	0.01499902
C4b	46.7140567	1.03279964	0.0465604	0.18331536	0.64554759	0.79391941
C5ar1	2.27916174	-1.0194619	-0.0278078	0.2173152	0.15837208	0.32413267
C5ar2	1.78451315	-1.0287851	-0.0409416	0.21985274	0.11314392	0.25481251
C7	5.88098853	-1.0531841	-0.0747576	0.22962138	0.10715554	0.24507452
C77080	1484.77746	-1.0678429	-0.0946994	0.06165017	0.11090901	0.25145918
C87436	536.268054	-1.0110596	-0.0158681	0.0950796	0.85157972	0.92033546
C8g	3.74228515	1.04277265	0.06042464	0.22340872	0.1663751	0.33536821
C9orf72	260.622917	-1.420752	-0.5066547	0.14788603	7.95E-05	0.00066598
CASP 3	1751.95339	-1.3062472	-0.3854279	0.0600566	3.28E-11	8.75E-10
CCNB1	7839.62785	1.04326835	0.0611103	0.06724444	0.33734521	0.53701903
CDC25B	3243.02708	1.05557385	0.07802752	0.05290014	0.12766898	0.27770579
CDC6	2846.87437	-1.0576282	-0.0808325	0.06365208	0.18279794	0.35881728
CENPA	305.088207	-1.097752	-0.1345521	0.11211234	0.16177636	0.32858017

CIAO1	680.517927	-1.0489701	-0.0689736	0.09168458	0.40778033	0.60683852
CIAPIN1	1330.39595	-1.0872704	-0.1207108	0.09032527	0.13928561	0.29551304
CIRBP	3064.96642	1.04484189	0.06328464	0.05295549	0.21758692	0.40407197
CLP1	524.34256	1.05187658	0.07296543	0.10920133	0.4389091	0.63652503
CN725425	14.3128479	1.04424975	0.06246679	0.21350421	0.3931421	0.59416592
COPE	1110.69687	1.1080914	0.14807689	0.10927043	0.11735027	0.26161798
COQ5	419.928612	1.08005233	0.11110122	0.1141339	0.25190806	0.44320042
COX1	178331.155	1.06489724	0.09071422	0.16275416	0.40379068	0.60349534
COX2	69306.1413	1.04447866	0.06278302	0.15743164	0.57054349	0.73966826
COX3	109181.659	1.18681034	0.24708941	0.20414075	0.06671785	0.17342851
CTU1	369.342711	-1.0688961	-0.0961217	0.10423719	0.29041112	0.48699652
CYTB	59574.5194	1.17306689	0.23028528	0.20033317	0.08129765	0.20031216
Caap1	543.98108	-1.0398814	-0.0564189	0.10149657	0.52791222	0.7090119
Cab39l	626.922685	1.00959302	0.01377384	0.0872605	0.86361295	0.92685003
Cabin1	3957.83818	1.09500702	0.13094012	0.05773927	0.0182614	0.06374074
Cables1	201.953865	-1.2158559	-0.2819723	0.16390927	0.02478081	0.08122193
Cables2	1269.07947	2.18919313	1.13039923	0.09037981	5.19E-37	7.00E-35
Cabp4	5.65689468	1.04408764	0.06224282	0.22156898	0.24192566	0.43180545
Cabyr	27.7157481	-1.0459738	-0.0648467	0.20925521	0.42459774	0.62300818
Cacfd1	286.637184	1.26316855	0.33704715	0.14447215	0.00481197	0.02144794
Cachd1	3565.43463	-1.0241742	-0.0344611	0.06030163	0.55085898	0.72739961
Cacna1b	6.18137439	-1.065521	-0.0915591	0.24021655	0.04763554	0.13423038
Cacna1c	590.549803	1.12941504	0.17557574	0.11376842	0.07170631	0.1823259
Cacna1g	180.260084	1.76513349	0.81977729	0.2191389	1.28E-05	0.00012919
Cacna2d1	4152.79218	1.10073385	0.13846568	0.05816316	0.01322904	0.04914304
Cacna2d2	4.52384815	-1.0113429	-0.0162722	0.21293962	0.66244472	0.80471736
Cacnb3	338.680403	1.21955527	0.28635514	0.17302981	0.02703658	0.08649645
Cacnb4	4.47834952	-1.0075201	-0.0108086	0.21217921	0.7768472	0.87546517
Cacng4	9.28666777	-1.1097046	-0.1501757	0.28938657	0.01863104	0.06473111
Cactin	1705.65874	-1.0551668	-0.0774711	0.06090156	0.18737873	0.36490141
Cacul1	2083.70833	-1.2620667	-0.3357881	0.06335637	3.49E-08	5.79E-07
Cacybp	4409.76398	-1.1523573	-0.2045882	0.07243984	0.00264501	0.01321131
Cad	8562.42487	-1.0815347	-0.11308	0.05181152	0.02433007	0.08014095
Cadps	254.587861	2.11553038	1.0810194	0.15426059	1.82E-13	6.28E-12
Cage1	9.8458083	-1.1204908	-0.1641307	0.31186881	0.0074803	0.03072852
Calcoco1	813.366474	1.19373754	0.25548567	0.07951957	0.00056029	0.00359048
Calcr	28.6165144	-5.0735346	-2.3429912	0.51637323	2.72E-07	3.89E-06
Cald1	5202.95851	1.09721051	0.13384035	0.05719258	0.01501184	0.05455366
Calhm2	176.860027	1.04050192	0.05727963	0.1449481	0.59753127	0.76034325
Calhm3	41.4050759	-1.0203926	-0.0291243	0.1893251	0.75180885	0.86245729
Calhm5	2.61395182	-1.0171806	-0.0245758	0.21537161	0.41974696	0.61839662
Calhm6	42.6598634	1.11369162	0.15534981	0.2609737	0.11930657	0.26464365
Calm1	12269.2965	1.08856017	0.12242116	0.05032023	0.01217774	0.04607115
Calm3	2821.81148	1.20797462	0.27259015	0.07554842	0.00012261	0.00097134
Calr	39197.6026	1.14583086	0.1963941	0.0463895	1.40E-05	0.00014063
Calr3	54.185842	-1.0445278	-0.0628509	0.19072688	0.52891055	0.70969076
Calr4	65.8982312	-1.1537042	-0.2062733	0.22931758	0.11144907	0.25237098
Calu	8016.12312	1.24936748	0.32119788	0.09172174	0.00014957	0.00115911
Camk1	1489.32588	1.19985888	0.26286473	0.07377396	0.00015398	0.00118828
Camk2b	1.80969853	-1.0062044	-0.0089233	0.21471317	0.65741497	0.80140161

Camk2d	4608.91186	1.35883864	0.44237415	0.05415464	6.15E-17	2.91E-15
Camk2g	2989.79114	1.09303542	0.12834015	0.05912513	0.02364776	0.07836554
Camk2n1	297.463504	1.02078272	0.02967582	0.12419705	0.77142977	0.87286097
Camk2n2	30.8664286	1.05693523	0.07988697	0.21200499	0.36042717	0.56080068
Camkk1	186.548809	1.58892339	0.66804956	0.16399736	4.45E-06	4.99E-05
Camkk2	1804.56875	-1.0449467	-0.0634294	0.06471804	0.30268654	0.50059975
Camkmt	409.83471	1.23523588	0.30478656	0.11138708	0.00190956	0.01003754
Caml	895.814209	1.11465929	0.1566028	0.07839883	0.03154339	0.09767243
Camsap1	2384.56583	1.03701992	0.05244361	0.05734431	0.34303508	0.54250362
Camsap2	4872.61807	1.01416788	0.02029649	0.05308329	0.69371781	0.82486113
Camsap3	263.202424	-1.0228826	-0.0326405	0.12678303	0.74962979	0.8610022
Camta1	43.2178284	-1.0241892	-0.0344823	0.18287972	0.72761677	0.846727
Camta2	296.838643	1.27796353	0.35384666	0.16967685	0.00765651	0.03128544
Cand1	9353.25196	-1.0557566	-0.0782773	0.04757089	0.09129019	0.21838045
Cand2	411.316955	1.02118483	0.03024402	0.10343821	0.7409523	0.85589602
Cant1	1390.69663	1.28232036	0.35875673	0.09009527	1.73E-05	0.00016889
Canx	14968.0377	1.00208728	0.00300817	0.05828069	0.95584367	0.97876558
Cap1	9187.52147	1.01890986	0.02702643	0.05339222	0.60184607	0.76386091
Capg	2644.28001	-2.0396084	-1.0282922	0.08163021	1.61E-37	2.24E-35
Capn1	1457.71142	1.38525832	0.47015504	0.09677862	1.95E-07	2.87E-06
Capn10	542.666329	1.02268831	0.03236651	0.08404975	0.67671963	0.81343516
Capn15	386.04394	1.02471127	0.03521746	0.11796594	0.72193807	0.84343968
Capn2	8226.97573	1.16091156	0.21525808	0.05497178	4.96E-05	0.00043518
Capn5	2354.62986	1.13163199	0.17840486	0.05861942	0.00151861	0.00824835
Capn7	1499.57079	1.07367544	0.10255795	0.08153187	0.17356744	0.34653973
Capn8	39.2595893	-1.0360886	-0.0511474	0.19428202	0.58233718	0.74804864
Capns1	4777.71879	-1.0199932	-0.0285596	0.05959835	0.61628706	0.77428768
Caprin1	20053.0521	1.00020006	0.0002886	0.06018942	0.99665598	0.99860486
Caprin2	210.06512	1.03863544	0.05468936	0.13016358	0.60122709	0.76340352
Caps2	17.161661	-1.0269538	-0.0383713	0.20789345	0.56687278	0.7376717
Capza1	3268.7058	-1.0552975	-0.0776497	0.05900188	0.17035082	0.34160833
Capza2	10571.2798	-1.0277117	-0.0394356	0.06179481	0.50686529	0.69125353
Car14	3.8283074	-1.0284595	-0.040485	0.21788208	0.27375968	0.46933371
Car5b	142.519375	-1.0725866	-0.1010942	0.16998684	0.37074889	0.57213508
Car9	43.5468056	1.06428525	0.08988487	0.191176	0.40409103	0.60358923
Card10	1113.69232	-1.1345267	-0.1820906	0.09629815	0.03416122	0.10414212
Card19	428.459506	-1.1006474	-0.1383524	0.10854433	0.14069198	0.29779647
Card6	436.795667	-1.5043834	-0.5891723	0.11421543	2.93E-08	4.98E-07
Card9	7.28744195	-1.0166659	-0.0238456	0.2126712	0.58571049	0.74971062
Carf	118.074618	1.03348033	0.04751092	0.15596931	0.66441243	0.8056724
Carhsp1	878.470248	-1.0670138	-0.0935789	0.09278962	0.26258581	0.45548061
Carm1	3686.07209	1.03594408	0.05094612	0.05324153	0.32325513	0.5226017
Carmil1	2543.24881	1.12844445	0.1743354	0.05637535	0.00131566	0.00728648
Carmil2	4.5157213	1.03803219	0.05385119	0.22222888	0.15380694	0.31676317
Carnmt1	3636.52787	1.00928756	0.01333727	0.05500741	0.79259047	0.88449202
Carns1	23.1183756	-1.0068779	-0.0098888	0.19376431	0.9057445	0.95103172
Cars	2314.32745	1.26572772	0.33996709	0.06534943	5.82E-08	9.44E-07
Cars2	187.463956	-1.2518884	-0.324106	0.19661842	0.02082962	0.07087051
Casc1	283.69049	-1.0080646	-0.0115882	0.11567432	0.90462894	0.95022649
Casc3	6259.62341	-1.0604364	-0.0846582	0.04365879	0.04885931	0.13689582

Casc4	2516.10047	1.00504544	0.00726073	0.0601056	0.90048954	0.9485077
Casd1	658.050932	-1.0379166	-0.0536905	0.08481021	0.4892323	0.67718402
Cask	2175.7139	1.03776094	0.05347415	0.05447798	0.31024628	0.50860697
Caskin2	967.239993	1.04051918	0.05730355	0.09334369	0.49535535	0.68187813
Casp1	3.13966554	-1.0181902	-0.0260071	0.21611552	0.33281194	0.5326488
Casp12	108.565813	-1.0854663	-0.1183149	0.16258314	0.30108165	0.49901099
Casp2	821.916701	-1.0126986	-0.0182049	0.0918724	0.82545825	0.90617192
Casp7	520.571097	1.13485241	0.18250468	0.09697504	0.03521604	0.10647096
Casp8	3536.03733	-1.1816738	-0.2408319	0.06310899	6.47E-05	0.00055317
Casp8ap2	1960.59304	1.0077008	0.01106735	0.06483426	0.85866453	0.92424944
Casp9	175.278971	1.33280977	0.41447089	0.19506804	0.0051524	0.02265498
Casq1	2.31206913	1.01293246	0.01853798	0.21457294	0.53985979	0.71842966
Cast	6123.13943	-1.0265833	-0.0378507	0.04848558	0.42046017	0.61905069
Castor1	54.1680456	-1.0147602	-0.0211388	0.17946895	0.83117766	0.90933349
Castor2	49.6267548	-3.0640258	-1.6154284	0.32126034	2.60E-08	4.46E-07
Cat	2696.60799	-1.15866	-0.2124573	0.05156173	2.10E-05	0.00019982
Catsper1	10.1602273	-1.0443547	-0.0626118	0.2180479	0.31828277	0.51688671
Catsper2	33.2923459	1.03043552	0.04325423	0.19484715	0.63639005	0.78820613
Cav1	4610.89456	-1.1158375	-0.158127	0.08119866	0.03471645	0.10535899
Cav2	1339.1192	-1.0643559	-0.0899806	0.08253043	0.23554873	0.42535389
Cavin1	28065.199	1.00334721	0.00482094	0.05293915	0.92470265	0.96122057
Cavin2	1.9999912	1.01951519	0.02788327	0.21650278	0.28661493	0.48309703
Cavin3	1022.08189	1.26657038	0.34092724	0.08850759	3.22E-05	0.00029467
Cavin4	161.390214	-10.125432	-3.3399115	0.22006221	5.34E-53	1.67E-50
Cbarp	162.471078	-1.0479952	-0.0676321	0.13676833	0.52489817	0.70665744
Cbfa2t2	735.29884	1.20289839	0.26651478	0.10012941	0.00299035	0.01454858
Cbfb	2770.6862	-1.1052712	-0.1444004	0.05878322	0.010305	0.04023813
Cbl	3895.37193	1.12074996	0.16446445	0.06007024	0.00424637	0.0194202
Cblb	796.444536	-1.1818573	-0.2410559	0.07935293	0.00109062	0.00626508
Cbr3	565.157155	-1.2168836	-0.2831912	0.10342876	0.00215281	0.01108215
Cbr4	755.142256	1.01485823	0.0212782	0.08354946	0.77928056	0.87673482
Cbwd1	1111.59239	1.11029044	0.15093711	0.08593333	0.05505183	0.14947307
Cbx1	5570.04757	-1.1831692	-0.2426564	0.05887214	1.76E-05	0.00017161
Cbx2	1568.85291	1.04104511	0.05803259	0.07169462	0.39088134	0.59242664
Cbx3	1223.59783	-2.037885	-1.0270726	0.08882573	4.61E-32	5.28E-30
Cbx4	762.838624	1.03680264	0.0521413	0.09628419	0.54512614	0.72266023
Cbx5	4101.20904	-1.0817945	-0.1134265	0.08429187	0.14291651	0.30111192
Cbx6	1651.92507	-1.1848368	-0.2446884	0.07872716	0.00085171	0.00511024
Cbx7	21.0229908	-1.0063734	-0.0091657	0.20502518	0.88331717	0.93731717
Cbx8	736.233528	1.02846454	0.04049205	0.08017757	0.58339719	0.74830245
Cby1	243.393066	1.35570801	0.43904649	0.18720682	0.00281442	0.01388361
Cc2d1a	913.22729	1.12120502	0.16505011	0.08282961	0.03045551	0.09496958
Cc2d1b	1434.37764	-1.0115774	-0.0166068	0.06461706	0.78707528	0.88066764
Cc2d2a	1804.79238	1.2415435	0.31213482	0.07123972	3.95E-06	4.46E-05
Ccar1	4079.06854	-1.1565422	-0.2098179	0.06647049	0.00088115	0.00525737
Ccar2	3201.74133	1.04328832	0.06113791	0.05697627	0.26596383	0.45990775
Ccbe1	745.122313	-1.0299952	-0.0426377	0.08851102	0.59566052	0.7587919
Ccdc102a	1177.68538	-1.035804	-0.050751	0.07234645	0.45565963	0.64999448
Ccdc103	138.27894	1.35184852	0.43493351	0.19539364	0.00373452	0.01749788
Ccdc106	3.59818345	1.02387762	0.03404328	0.21488848	0.43260855	0.63045684

Ccdc107	109.478544	1.14267438	0.19241435	0.21894478	0.13157828	0.28351855
Ccdc112	291.470133	-1.0835632	-0.1157834	0.13281709	0.27674798	0.4723077
Ccdc114	257.608791	-1.1507701	-0.2025996	0.15725685	0.08776101	0.21255787
Ccdc115	829.23305	-1.0588286	-0.0824691	0.08017716	0.26669123	0.4609167
Ccdc117	559.315775	-1.0287121	-0.0408393	0.08740143	0.60929553	0.76937519
Ccdc12	510.042938	-1.1354218	-0.1832283	0.10461427	0.04613039	0.13102667
Ccdc120	420.258925	-1.1235643	-0.1680826	0.12801014	0.10929765	0.24873032
Ccdc122	249.378679	1.11792568	0.16082428	0.14953468	0.15888329	0.32507507
Ccdc124	1616.61358	1.04427088	0.06249599	0.07150374	0.35381952	0.5534776
Ccdc125	2.99002453	-1.0046327	-0.0066681	0.21185262	0.86312081	0.92665838
Ccdc126	744.829691	-1.7714964	-0.8249685	0.09646493	1.07E-18	5.81E-17
Ccdc127	427.123359	-1.0883034	-0.1220809	0.10553871	0.18401015	0.36058897
Ccdc13	3.82999214	-1.0490438	-0.0690749	0.22894119	0.06742647	0.17470548
Ccdc130	379.024167	1.13154317	0.17829163	0.11079348	0.06268191	0.16548955
Ccdc134	171.825645	1.34681552	0.42955225	0.19470537	0.00391669	0.01816867
Ccdc136	269.686683	1.27514098	0.35065676	0.15723239	0.00574591	0.02471631
Ccdc137	2379.88602	1.025405	0.03619384	0.06688195	0.56993318	0.73925111
Ccdc138	268.068908	1.69792727	0.76377467	0.14097841	5.49E-09	1.05E-07
Ccdc14	561.554162	1.11019098	0.15080788	0.10017039	0.0891072	0.21468182
Ccdc141	11.3493654	-1.0233835	-0.0333468	0.20974429	0.57682094	0.74394362
Ccdc146	636.233451	1.43585475	0.52190981	0.15790125	0.00012005	0.00095697
Ccdc148	8.05146203	-1.0908428	-0.1254433	0.2630405	0.03525568	0.10654053
Ccdc15	471.161087	1.27280638	0.34801297	0.12134035	0.00104035	0.00602752
Ccdc150	478.697683	1.13326281	0.18048247	0.13154911	0.09287009	0.2209239
Ccdc151	468.941034	1.03245473	0.04607852	0.10173754	0.6082331	0.76854074
Ccdc153	3.96613446	-1.0040495	-0.0058304	0.21214903	0.87398988	0.93213247
Ccdc154	10.0878616	-1.053127	-0.0746794	0.22139703	0.27013048	0.46554169
Ccdc157	38.7153572	1.00642282	0.00923654	0.18450611	0.92520461	0.96161226
Ccdc159	59.5290984	1.16231872	0.21700573	0.27016215	0.09180882	0.21917088
Ccdc163	213.88753	2.0969834	1.06831544	0.18250768	2.89E-10	6.65E-09
Ccdc167	84.2055612	1.73029343	0.79101672	0.26542323	0.00019676	0.00147399
Ccdc17	18.8943627	1.09153069	0.12635269	0.24349102	0.15218789	0.31449055
Ccdc170	325.728653	1.18919286	0.24998271	0.13627529	0.02528371	0.08233085
Ccdc171	58.0496453	-1.0007885	-0.0011372	0.16878671	0.9898178	0.99536884
Ccdc173	195.432008	1.03414985	0.04844525	0.12957991	0.64324375	0.79213033
Ccdc174	687.768534	-1.0358081	-0.0507568	0.08414697	0.51037233	0.69427955
Ccdc18	700.065316	1.01973512	0.02819445	0.08847614	0.72400491	0.84436479
Ccdc181	60.1943988	-1.0566662	-0.0795197	0.19638282	0.43170137	0.62965965
Ccdc186	2693.04687	-1.0317257	-0.0450594	0.07274736	0.50993563	0.69411066
Ccdc189	39.9316551	1.01758835	0.02515406	0.19661359	0.75973331	0.86676548
Ccdc191	192.163778	-1.0629742	-0.0881065	0.13366724	0.40432266	0.60366653
Ccdc22	1219.54594	-1.0713603	-0.0994437	0.08166784	0.18654871	0.36372739
Ccdc24	67.7331112	-1.0484649	-0.0682786	0.19749784	0.47951958	0.67008244
Ccdc25	819.261452	-1.0401748	-0.056826	0.07790877	0.43323957	0.63123282
Ccdc28a	354.756262	1.04774797	0.06729173	0.10360716	0.45909638	0.65322944
Ccdc28b	11.9059792	-1.0964842	-0.1328851	0.26046562	0.08213218	0.20170882
Ccdc32	578.900226	-1.0371409	-0.0526119	0.10003917	0.55157799	0.7277643
Ccdc34	882.059155	1.10695627	0.14659823	0.0758498	0.03839116	0.1138668
Ccdc43	3289.07794	1.05570174	0.0782023	0.08725006	0.3267513	0.52661551
Ccdc47	4730.25224	-1.0121841	-0.0174717	0.05652377	0.74839196	0.86047595

Ccdc50	2115.93628	1.00364451	0.00524835	0.07162772	0.93905336	0.97054583
Ccdc51	324.389698	-1.0268792	-0.0382664	0.11894412	0.69942039	0.82866955
Ccdc57	381.210241	1.06229244	0.08718098	0.10728066	0.35015656	0.54976168
Ccdc58	3579.00411	-1.0810817	-0.1124755	0.08173863	0.13564564	0.2899901
Ccdc59	1945.49163	-1.0554446	-0.0778508	0.06888008	0.23201953	0.42124475
Ccdc6	5341.83753	-1.0430816	-0.060852	0.04707889	0.18585624	0.36309569
Ccdc62	1.72525117	1.00775164	0.01114013	0.21422846	0.67550066	0.81273255
Ccdc65	130.240798	1.2046251	0.26858423	0.18168187	0.04050435	0.11882096
Ccdc66	537.684589	1.21031317	0.27538039	0.12557333	0.00979821	0.03860813
Ccdc69	4.46765181	1.03161949	0.04491093	0.2167216	0.35072967	0.55032424
Ccdc71	522.235253	1.06823031	0.09522272	0.10086053	0.28748662	0.4837691
Ccdc71l	5.91694377	-1.0195194	-0.0278892	0.2123729	0.55956782	0.73310886
Ccdc74a	10.5511476	1.13102941	0.17763645	0.32623851	0.01195551	0.04541818
Ccdc77	2742.16267	-1.2807585	-0.3569984	0.07180981	1.78E-07	2.64E-06
Ccdc8	236.33325	1.12170427	0.16569237	0.13867246	0.13307618	0.28578472
Ccdc80	15602.6011	1.66279948	0.7336142	0.05719876	1.88E-38	2.80E-36
Ccdc81	8.25412659	-1.0501012	-0.0705284	0.2269829	0.14414319	0.30324755
Ccdc82	531.472363	1.10359822	0.14221503	0.11320181	0.14221526	0.30025542
Ccdc83	17.4200379	1.14085488	0.19011529	0.32669562	0.0349558	0.10585906
Ccdc84	155.86942	-1.1218781	-0.1659159	0.15534171	0.15261314	0.31511513
Ccdc85b	510.628291	1.15978774	0.21386079	0.14539578	0.06209094	0.16430177
Ccdc85c	223.68069	-1.1458046	-0.196361	0.15202399	0.0908604	0.21775896
Ccdc86	1752.73197	-1.2323369	-0.3013967	0.05935922	1.35E-07	2.05E-06
Ccdc87	9.77529234	-1.0173987	-0.0248851	0.21017267	0.64072071	0.79070924
Ccdc88a	4411.42024	-1.0816572	-0.1132433	0.06474832	0.0659937	0.17203486
Ccdc88c	127.370111	3.27905024	1.71327801	0.21280765	5.91E-17	2.81E-15
Ccdc9	227.840957	-1.0495728	-0.0698023	0.15235687	0.52481809	0.70665744
Ccdc90b	1136.88738	1.05155615	0.07252588	0.07774387	0.31665908	0.51558939
Ccdc91	466.991857	-1.0633206	-0.0885766	0.09789827	0.30915982	0.50753364
Ccdc92	660.595243	-1.0485906	-0.0684516	0.08442284	0.37615344	0.57789945
Ccdc93	1024.8507	1.04291496	0.06062153	0.07684853	0.3983765	0.59840561
Ccdc96	33.7642892	1.09255959	0.12771196	0.23554005	0.18950931	0.36787607
Ccdc97	555.862408	1.07712133	0.10718077	0.09638767	0.21301896	0.3989434
Ccdc9b	129.952569	-1.0407282	-0.0575934	0.13590109	0.58597743	0.74971062
Ccer2	1.93440467	1.01324899	0.01898874	0.21460185	0.51785945	0.70084827
Cchcr1	695.529324	1.04580705	0.0646167	0.09454426	0.44735011	0.64330021
Ccin	24.8215956	-1.039513	-0.0559078	0.21004528	0.44826388	0.64389552
Cckar	48.8306554	1.86298836	0.89761866	0.31395356	0.00023985	0.00174079
Ccl17	6.30336881	-1.081286	-0.1127481	0.25574545	0.02459516	0.08078571
Ccl2	6715.81848	-1.6371569	-0.7111926	0.06404166	1.23E-29	1.30E-27
Ccl7	185.341885	1.29090819	0.3683864	0.17060124	0.00603646	0.0256986
Ccm2	439.716696	1.02778614	0.0395401	0.109196	0.67495518	0.81253415
Ccn1	1872.60527	1.00779604	0.01120369	0.08105324	0.8825375	0.93698481
Ccn2	1889.32708	1.13553294	0.18336956	0.211233	0.14562012	0.30521725
Ccn5	17.5177924	5.38152665	2.4280155	0.60460063	3.49E-06	3.99E-05
Ccna2	8113.85239	1.09934094	0.13663888	0.07201339	0.04357836	0.12578613
Ccnb2	3926.97845	1.04326343	0.0611035	0.06185845	0.30241892	0.50032111
Ccnc	590.826909	-1.0014512	-0.0020922	0.10307178	0.98025556	0.99079336
Ccnd1	16352.203	-1.0320302	-0.0454853	0.05907418	0.41021991	0.60933639
Ccnd2	1280.34281	1.02657715	0.03784205	0.06997243	0.56774793	0.73823146

Ccnd3	1859.73036	1.11650624	0.15899132	0.08803181	0.047183	0.13316022
Ccndbp1	812.945699	1.08721552	0.12063795	0.07546122	0.08705953	0.21101845
Ccne1	507.700067	-1.1205051	-0.1641493	0.1052472	0.07444577	0.18772644
Ccne2	1151.3662	-1.1979082	-0.2605173	0.08415653	0.00080327	0.00485829
Ccnf	1432.78192	-1.0685389	-0.0956395	0.07789195	0.18584978	0.36309569
Ccng2	266.837431	1.01597769	0.02286872	0.12339529	0.82199749	0.90384445
Ccnh	1933.99793	-1.0423232	-0.0598027	0.07376712	0.38720107	0.5885199
Ccni	1682.6856	1.05311874	0.07466811	0.07480685	0.28637748	0.48293374
Ccnj	333.317097	-1.0969065	-0.1334406	0.11341964	0.16797414	0.3380595
Ccnjl	123.447689	-1.2523122	-0.3245943	0.21053459	0.02479139	0.08122193
Ccnl1	1567.68618	-1.1614722	-0.2159547	0.07103683	0.00125445	0.00700194
Ccnl2	1456.69191	-1.0598407	-0.0838475	0.08996188	0.30280265	0.50059975
Ccnt1	1214.43539	-1.0951277	-0.1310992	0.0790685	0.07333003	0.18538734
Ccnt2	494.343824	1.06703931	0.09361333	0.11380968	0.33455851	0.53450916
Ccny	772.47769	-1.0012772	-0.0018415	0.09897637	0.98230381	0.99112412
Ccnyl1	1393.93535	-1.3867677	-0.4717262	0.07248054	1.33E-11	3.70E-10
Ccp110	2408.26437	-1.0554588	-0.0778703	0.05798167	0.1623189	0.3295776
Ccpg1	2662.36614	-1.0326381	-0.0463347	0.06171074	0.43095032	0.62901895
Ccpg1os	27.3496536	-1.0499768	-0.0703574	0.201427	0.45451927	0.64920555
Ccr10	17.1523341	1.03252703	0.04617955	0.20653734	0.54001581	0.71845252
Ccr7	335.766997	-1.1423926	-0.1920586	0.15333344	0.0988815	0.23101805
Ccr9	3.06625192	1.01149394	0.01648768	0.21471581	0.5305114	0.71086309
Ccrl2	177.755304	1.55381455	0.63581433	0.16729852	1.44E-05	0.00014387
Ccs	1507.41631	-1.0336523	-0.047751	0.08073794	0.52203733	0.70426114
Ccsap	205.548194	1.01272821	0.01824704	0.13745606	0.864644	0.92717978
Ccser2	2281.12538	1.06460209	0.0903143	0.05337522	0.08076602	0.19943512
Cct2	11889.737	-1.0918151	-0.1267286	0.04963275	0.0085151	0.03427098
Cct3	15105.3168	-1.2240383	-0.2916487	0.05280925	1.00E-08	1.83E-07
Cct4	12768.0723	-1.1217796	-0.1657892	0.04740501	0.00032642	0.00223967
Cct5	12870.8759	-1.0641767	-0.0897378	0.04789395	0.05300844	0.14534208
Cct6b	6.69373141	1.00977978	0.01404069	0.2103919	0.77290022	0.87363026
Cct7	10283.6637	-2.1289736	-1.0901581	0.0491543	4.09E-110	6.55E-107
Cct8	14421.843	-1.1848632	-0.2447205	0.04532918	2.67E-08	4.57E-07
Ccz1	2591.85302	1.00875445	0.01257504	0.0652284	0.84019988	0.91430211
Cd109	1518.96325	-2.1380125	-1.0962703	0.07875234	2.91E-45	6.65E-43
Cd151	803.35007	-1.0343759	-0.0487605	0.12346352	0.64778465	0.79544875
Cd164	8120.12171	1.08428077	0.11673838	0.05345393	0.02392882	0.07912448
Cd276	1601.90553	-1.3378515	-0.4199179	0.06851448	1.73E-10	4.16E-09
Cd28	7.60739592	-1.0819035	-0.1135719	0.25694426	0.02099619	0.07136141
Cd2ap	6490.62429	1.03961172	0.0560448	0.04690826	0.22164838	0.40954884
Cd2bp2	2088.03522	-1.0197664	-0.0282387	0.0724728	0.67818159	0.81477292
Cd320	314.16616	-1.0382625	-0.0541712	0.13168608	0.60306413	0.76487639
Cd36	126.382298	1.03042276	0.04323636	0.16599464	0.68978845	0.82250675
Cd3eap	1747.06965	-1.0258342	-0.0367976	0.07153962	0.58502927	0.74938527
Cd44	9500.5167	-1.0115242	-0.0165308	0.0437791	0.6991511	0.82849333
Cd46	39.9208038	-1.4028391	-0.4883496	0.36101095	0.0129052	0.04826733
Cd47	832.445208	1.11524318	0.15735833	0.07696802	0.02808541	0.08885505
Cd63	13295.7824	1.22469989	0.29242826	0.04540491	4.60E-11	1.21E-09
Cd81	656.063751	1.14047208	0.18963113	0.10479004	0.03901312	0.11544421
Cd82	5.88200434	-1.1359281	-0.1838715	0.348812	0.0020641	0.01069193

Cd9	11.3132689	-1.0639693	-0.0894566	0.23492808	0.11701054	0.26113327
Cd96	2.49603222	1.02741763	0.03902274	0.21939439	0.09532928	0.22521516
Cd99l2	1633.78546	1.14196678	0.19152068	0.07013125	0.00378745	0.0176844
Cda	13.8225892	-1.1158265	-0.1581127	0.30418811	0.00758672	0.03106964
Cdadc1	182.968335	-1.0219533	-0.0313293	0.14114037	0.76856381	0.87144584
Cdan1	382.460522	1.01345377	0.01928028	0.1019935	0.8280157	0.90781248
Cdc123	2421.49507	-1.0233931	-0.0333604	0.06794065	0.60392276	0.76556019
Cdc14a	1567.60899	-1.2047108	-0.2686868	0.06585092	1.86E-05	0.00018037
Cdc14b	308.053861	-1.0620422	-0.0868411	0.11542363	0.37323251	0.57484647
Cdc16	5670.13688	1.04200224	0.05935838	0.04431533	0.17107071	0.34275892
Cdc20	4541.73467	-1.0617067	-0.0863852	0.05418413	0.09873061	0.23083393
Cdc23	1097.15839	1.0242952	0.03463156	0.08186762	0.64801999	0.79553628
Cdc25a	3789.98784	-1.2453315	-0.3165298	0.0544509	2.03E-09	4.13E-08
Cdc26	990.644408	1.03131642	0.04448703	0.08542132	0.57103168	0.73980047
Cdc27	5538.56082	1.07191802	0.10019457	0.06767191	0.11789711	0.26234587
Cdc37l1	1336.69057	-1.0594507	-0.0833165	0.06514166	0.17865803	0.35350779
Cdc40	1648.5586	1.01742396	0.02492097	0.07216723	0.7145779	0.83894629
Cdc42bpa	5121.0758	1.06485648	0.090659	0.0539721	0.08211662	0.20170882
Cdc42bpb	7915.71171	1.13799981	0.18650031	0.04415927	1.65E-05	0.00016254
Cdc42ep1	1675.54371	-1.2155842	-0.2816498	0.06979169	2.06E-05	0.00019618
Cdc42ep2	59.4434054	2.39164782	1.25800496	0.34129063	1.10E-05	0.00011372
Cdc42ep3	568.131478	-1.1179136	-0.1608087	0.11074261	0.0921887	0.21979105
Cdc42se1	275.757418	-1.073288	-0.1020373	0.128523	0.32542726	0.5251604
Cdc42se2	637.430592	-1.0463555	-0.065373	0.08526309	0.40218473	0.60193789
Cdc45	1398.97216	-1.0019807	-0.0028547	0.06634474	0.96330481	0.98254201
Cdc7	2648.20769	-1.0969934	-0.1335548	0.07356962	0.05210507	0.14347934
Cdc73	3131.58204	-1.0531899	-0.0747656	0.07619549	0.29268624	0.4897074
Cdca2	3065.17668	1.19913485	0.26199391	0.06641379	3.42E-05	0.00030971
Cdca3	2171.24204	1.04084265	0.05775199	0.07788307	0.42662571	0.62469495
Cdca4	706.677395	-1.1891996	-0.2499909	0.11169797	0.01023395	0.04001263
Cdca5	771.713979	1.01632107	0.02335625	0.10476985	0.79573545	0.88630303
Cdca7	3808.55381	-1.1601572	-0.2143203	0.07303754	0.00178313	0.00948192
Cdca8	1229.52066	-1.1188312	-0.1619923	0.07283462	0.01775822	0.06252963
Cdh1	35.7801085	-2.2177001	-1.1490643	0.64077911	0.00221277	0.01134658
Cdh13	3.07646751	-1.0641515	-0.0897035	0.24260879	0.00417801	0.01916917
Cdh17	3.20376692	-1.0316018	-0.0448863	0.21947405	0.20762367	0.39245378
Cdh2	18.4136081	-1.0928807	-0.1281359	0.26215028	0.05406092	0.14747028
Cdh24	68.5519512	1.75664299	0.81282102	0.30571401	0.00044464	0.00293904
Cdh9	296.266114	1.02018675	0.02883327	0.11365239	0.76415371	0.86949805
Cdip1	153.268568	1.05903182	0.08274594	0.15815629	0.45794636	0.65210015
Cdipt	574.742125	1.03807076	0.05390479	0.10572887	0.55981366	0.73310886
Cdiptos	10.7118073	1.0544685	0.076516	0.22263912	0.26135034	0.45382919
Cdk1	6360.28637	1.03591414	0.05090443	0.05222411	0.31124972	0.50973011
Cdk10	451.318745	1.21215411	0.27757313	0.09906919	0.00185491	0.00980256
Cdk11b	3931.2167	-1.0846508	-0.1172306	0.0609246	0.04423342	0.12699016
Cdk12	11707.2502	-1.0128884	-0.0184752	0.04387505	0.65032552	0.79726959
Cdk13	3420.21902	-1.0199373	-0.0284805	0.05938679	0.61723356	0.77476042
Cdk14	4481.84026	1.26094086	0.33450062	0.05276713	7.13E-11	1.80E-09
Cdk15	18.2421222	1.01050774	0.01508038	0.20349431	0.82728504	0.9074592
Cdk16	2798.55691	1.06468117	0.09042146	0.05227538	0.07421469	0.18725432

Cdk17	2083.53856	-1.1476538	-0.1986874	0.06472492	0.00124166	0.00693727
Cdk19	2264.44826	1.11690538	0.15950697	0.06277608	0.00770351	0.03143547
Cdk2	3248.12214	1.02390153	0.03407698	0.05880478	0.54586138	0.72318588
Cdk20	209.17183	1.01203713	0.01726222	0.13728456	0.87157971	0.93041135
Cdk2ap2	299.38378	1.09719119	0.13381495	0.12439873	0.19350317	0.37301909
Cdk3	7.24422607	-1.0264988	-0.0377319	0.21400459	0.44658535	0.64292631
Cdk5	897.907612	-1.6517301	-0.723978	0.09407453	1.25E-15	5.34E-14
Cdk5r1	24.7135661	1.08201631	0.11372224	0.23457945	0.19281352	0.37186709
Cdk5rap1	962.618491	-1.0179686	-0.0256931	0.07727649	0.72078751	0.84286004
Cdk5rap2	3878.9098	1.21329395	0.27892912	0.06884806	1.98E-05	0.00018964
Cdk5rap3	1217.12769	1.13767315	0.18608614	0.07372019	0.00708261	0.02935241
Cdk6	4003.88762	1.05891322	0.08258437	0.05519554	0.12115656	0.26749666
Cdk7	1016.66806	-1.0959129	-0.1321332	0.08444136	0.08781895	0.21261714
Cdk8	2044.0478	-1.0611239	-0.0855932	0.06147349	0.14670049	0.30679235
Cdk9	2671.24989	-1.1316525	-0.178431	0.06175009	0.00248131	0.01252387
Cdkal1	1319.65762	1.16888133	0.22512847	0.07174129	0.00086409	0.00517728
Cdkl1	3.71282246	1.01048649	0.01505003	0.21264606	0.69382347	0.82486113
Cdkl2	321.415605	1.32867943	0.40999307	0.13786215	0.00054369	0.00350866
Cdkl3	34.9004619	1.05730487	0.08039144	0.20168565	0.41286002	0.61183906
Cdkl5	7.5464905	-1.166846	-0.2226142	0.44956405	0.00039074	0.0026261
Cdkn1a	875.813618	-1.1955595	-0.2576859	0.09630873	0.00301902	0.01466021
Cdkn1b	1892.88527	1.0136805	0.019603	0.08285617	0.79898262	0.88860215
Cdkn2aip	375.741954	-1.1423004	-0.1919421	0.10870516	0.04240467	0.12312175
Cdkn2aipnl	896.872653	1.04296841	0.06069546	0.08788296	0.44753723	0.64331604
Cdkn2b	343.85954	1.24084184	0.31131924	0.1455243	0.00879275	0.03517649
Cdkn2c	545.121217	1.08973636	0.12397915	0.11853855	0.2140466	0.39993246
Cdkn2d	42.5140503	-1.0021992	-0.0031693	0.18826591	0.97035701	0.98621654
Cdkn3	2735.26936	-1.0999641	-0.1374564	0.07921679	0.06091662	0.16193025
Cdnf	45.8322471	1.10392665	0.14264431	0.21714176	0.2121018	0.39786559
Cdon	1660.45495	1.138931	0.18768034	0.06047165	0.00118049	0.00667935
Cdpf1	32.5509303	-1.0437197	-0.0617343	0.20264757	0.48196506	0.67185637
Cdr2	1111.62924	-1.0325083	-0.0461534	0.08185027	0.54109506	0.71940091
Cdr2l	815.443822	1.2397498	0.310049	0.09198204	0.00024031	0.00174272
Cds1	627.852845	1.29268886	0.37037507	0.09501867	2.32E-05	0.00021807
Cds2	730.744878	-1.0917978	-0.1267057	0.09255319	0.12617484	0.2752554
Cdsn	97.7522317	1.07296103	0.10159768	0.16862448	0.37233962	0.57403666
Cdt1	1278.12253	1.0938218	0.12937772	0.0827415	0.08945685	0.21532174
Cdv3	3226.81507	-1.319779	-0.4002964	0.06635634	3.63E-10	8.19E-09
Cdyl	1524.25485	-1.098724	-0.1358291	0.06781299	0.03422825	0.10426719
Cebpa	6.78366146	1.00065216	0.00094057	0.20812615	0.98786376	0.99414995
Cebpb	58.6120271	1.02796963	0.03979765	0.19294595	0.66595577	0.80655133
Cebpd	14.4578745	-1.047039	-0.0663152	0.21151934	0.39727835	0.59750998
Cebpe	17.0587314	1.0609178	0.08531288	0.22970221	0.17620384	0.34994902
Cebpg	243.447028	1.41698993	0.5028295	0.15724433	0.00018418	0.00139603
Cebpz	10098.762	-1.0101383	-0.0145529	0.04887627	0.75939713	0.86662603
Cebpzos	484.147754	-1.0128281	-0.0183893	0.1199173	0.85291493	0.92095838
Celf1	2803.73294	1.03874872	0.0548467	0.05148574	0.272557	0.46833737
Celf4	2.48121254	1.01782232	0.02548574	0.21576391	0.38085615	0.58280281
Celsr2	6.19744444	-1.0333087	-0.0472713	0.21791257	0.29858454	0.49638308
Celsr3	6.67892895	-1.031924	-0.0453367	0.21592623	0.37134141	0.57277344

Cemip2	1712.04563	-1.0775234	-0.1077192	0.0625834	0.07070791	0.1804046
Cenpb	603.229674	1.0720552	0.1003792	0.10760303	0.28204268	0.47803212
Cenpc1	1457.66323	1.07698482	0.10699792	0.07701223	0.13611257	0.29064877
Cenpe	20222.504	1.01347287	0.01930747	0.06451634	0.75523404	0.86392958
Cenpf	16261.2126	-1.0106569	-0.0152933	0.06205276	0.80216753	0.89060202
Cenph	1065.75734	-1.1292077	-0.1753109	0.08310165	0.02181049	0.07354366
Cenpi	606.987881	-1.0758525	-0.1054803	0.09325671	0.20824597	0.39316593
Cenpj	1832.47735	1.07850463	0.10903237	0.07114918	0.10378003	0.24001123
Cenpk	810.313251	-1.1523921	-0.2046317	0.10515526	0.0267058	0.08569671
Cenpl	932.940401	1.00012993	0.00018743	0.08818471	0.99895786	0.99959215
Cenpm	576.20844	1.03317937	0.04709074	0.11690729	0.63220406	0.78541342
Cenpn	1261.97932	1.0520579	0.07321411	0.06482496	0.23597493	0.42569199
Cenpp	337.722477	-1.2240864	-0.2917054	0.15310171	0.01631099	0.05811411
Cenpq	1552.13211	1.03390392	0.04810212	0.0681215	0.45698408	0.651244
Cenps	1166.62327	1.10977314	0.15026478	0.08414501	0.05205791	0.14338031
Cenpt	1018.97206	1.07288383	0.10149387	0.08549007	0.1952067	0.37529608
Cenpu	842.044603	1.01784309	0.02551518	0.09275728	0.76106795	0.86757833
Cenpv	756.21712	1.24102606	0.31153341	0.09251152	0.00024039	0.00174272
Cenpw	716.448404	-1.1362362	-0.1842628	0.13051022	0.08531647	0.20771252
Cep104	1139.9779	1.17709446	0.2352301	0.07916881	0.00140546	0.00772037
Cep112	365.008774	-1.0712184	-0.0992527	0.1245704	0.32678116	0.52661551
Cep120	1567.81851	1.03291397	0.0467201	0.06045075	0.42101307	0.61962057
Cep126	163.903048	-1.0349918	-0.0496194	0.14295096	0.64433443	0.79303651
Cep128	1685.57132	1.14323486	0.19312181	0.0636507	0.00145791	0.00795055
Cep131	1536.63085	1.23947233	0.30972606	0.075007	1.22E-05	0.00012405
Cep135	4555.72254	-1.0527307	-0.0741364	0.06109125	0.20458691	0.38837557
Cep152	2084.45753	-1.0772582	-0.1073641	0.06109666	0.06653407	0.17312644
Cep162	1960.26537	-1.030268	-0.0430197	0.06442256	0.48330165	0.67257948
Cep164	3568.70634	1.23972921	0.31002503	0.04690859	1.42E-11	3.93E-10
Cep170	10336.9099	1.05200963	0.07314791	0.05050415	0.13595454	0.29040815
Cep170b	1932.90559	1.36579762	0.44974373	0.08802576	5.95E-08	9.62E-07
Cep19	608.953504	-1.1707041	-0.2273765	0.09734768	0.00925528	0.03667187
Cep192	1424.1835	1.06598432	0.09218622	0.06672366	0.14512003	0.30455236
Cep250	2283.24635	1.11977211	0.16320515	0.07360962	0.01801704	0.06319779
Cep290	3349.62953	1.10827669	0.14831811	0.06892113	0.02272922	0.07596172
Cep295	4708.72728	-1.0462188	-0.0651847	0.05781083	0.24110167	0.43075486
Cep295nl	169.509149	-1.0628745	-0.0879713	0.15181742	0.42770058	0.62572458
Cep350	5238.37897	1.08166258	0.11325052	0.05425506	0.03084808	0.09589109
Cep41	520.827788	1.14517034	0.19556221	0.11292586	0.04444291	0.12739744
Cep44	475.73905	-1.1182355	-0.161224	0.13245891	0.13220735	0.28425246
Cep55	2048.05637	-1.0996047	-0.1369849	0.06524108	0.02706173	0.08649645
Cep57	1065.16262	-1.090685	-0.1252345	0.08539743	0.10885836	0.24799494
Cep57l1	2112.36467	-1.1014774	-0.1394399	0.06273604	0.01974765	0.0679098
Cep63	728.560943	-1.1672167	-0.2230725	0.08758113	0.00539034	0.02351847
Cep68	698.651306	1.05994939	0.08399538	0.08365527	0.27453973	0.46993205
Cep70	1091.59218	1.11476292	0.15673692	0.07660725	0.02805267	0.08881727
Cep72	545.578111	-1.1483194	-0.199524	0.1061225	0.03170683	0.09803632
Cep78	495.948418	1.13736293	0.18569269	0.10164321	0.03912111	0.11563024
Cep83	3276.51278	-1.0422854	-0.0597504	0.05289127	0.22561927	0.41412563
Cep85	2320.06673	-1.1189029	-0.1620848	0.05922073	0.00426195	0.01946789

Cep85l	357.966021	1.01724515	0.0246674	0.11204853	0.79782451	0.88793762
Cep95	878.760853	-1.0011041	-0.0015921	0.09355411	0.98443544	0.99179064
Cep97	573.34946	-1.0056177	-0.008082	0.08684003	0.91984795	0.95871614
Cept1	1178.42224	-1.0238299	-0.0339761	0.0935022	0.68597522	0.8196191
Cercam	281.876332	1.4076456	0.49328415	0.12680852	1.47E-05	0.00014668
Cerk	390.588449	1.10018139	0.13774141	0.11925014	0.1694969	0.34052128
Cers2	986.924535	-1.1200011	-0.1635002	0.07629382	0.02133489	0.07228245
Cers5	1000.30717	-1.0442564	-0.062476	0.08192759	0.40810449	0.60710934
Cers6	517.759245	-1.2737593	-0.3490927	0.10800397	0.00029949	0.00208165
Cert1	1816.25383	-1.0117887	-0.0169081	0.07977705	0.81790053	0.90166143
Cetn3	3365.37814	1.0068663	0.00987212	0.05705743	0.85836022	0.92418976
Cetn4	122.063497	1.32669479	0.40783651	0.25062297	0.01347931	0.04994793
Cfap20	1577.87385	1.02861758	0.04070671	0.0722694	0.54970614	0.72632652
Cfap206	5.30103329	-1.0338761	-0.0480633	0.2178182	0.30450118	0.50256561
Cfap298	732.921919	1.07617348	0.10591066	0.08259909	0.16380821	0.33170773
Cfap36	3268.42212	1.05137513	0.07227751	0.06490246	0.24542348	0.43577396
Cfap410	248.114636	1.11865347	0.1617632	0.18570915	0.18396263	0.36055099
Cfap45	71.1511223	-4.524601	-2.1777906	0.28662865	1.75E-15	7.38E-14
Cfap57	2.21292344	-1.0088204	-0.0126693	0.21433042	0.6358672	0.78791438
Cfap69	84.8542593	1.31010126	0.38967832	0.24286096	0.01541706	0.05571017
Cfap70	41.839543	-1.6963542	-0.7624375	0.39569358	0.00276121	0.01368852
Cfap97	1644.79564	1.33758749	0.41963326	0.06761003	1.14E-10	2.83E-09
Cfdp1	3809.73584	1.04864785	0.06853028	0.06040325	0.23643318	0.42597877
Cfi	8.68081484	-1.0073549	-0.0105721	0.20901991	0.83555473	0.91187361
Cfl1	13892.1054	-1.0622839	-0.0871694	0.06581508	0.16331633	0.33086861
Cflar	3271.40075	-1.0975141	-0.1342395	0.06238513	0.02396196	0.07921359
Cfp	3.82058334	-1.022928	-0.0327047	0.21694041	0.28541386	0.48190417
Cgas	522.281008	-1.2139298	-0.279685	0.0937461	0.0010332	0.00599694
Cggbp1	1403.00444	1.15662516	0.20992139	0.0677896	0.00108421	0.0062393
Cgn	23.9531646	-1.1494739	-0.2009737	0.3037375	0.07096184	0.18089973
Cgrrf1	644.318804	-1.1030816	-0.1415396	0.09919016	0.10736803	0.24529775
Chac1	728.884436	1.23497856	0.304486	0.12312487	0.00431735	0.01962596
Chac2	477.588339	-1.2482607	-0.3199193	0.11360787	0.00140489	0.00772037
Chadl	4.67712886	1.02301271	0.03282407	0.21711855	0.27482803	0.47009574
Chaf1a	2424.01369	-1.1130652	-0.1545381	0.05838338	0.00578748	0.02487005
Chaf1b	1226.59733	1.02360718	0.03366217	0.07410809	0.62908331	0.78322064
Champ1	1667.17343	-1.0403911	-0.057126	0.07268913	0.40303782	0.60272175
Chchd1	398.528529	-1.0381075	-0.0539559	0.10569133	0.55725642	0.73133339
Chchd3	3302.67182	-1.0302669	-0.0430181	0.05952984	0.4514709	0.64675978
Chchd5	159.513756	1.03215865	0.04566475	0.14595413	0.67346255	0.81127095
Chchd6	1226.79386	1.04284141	0.06051978	0.07031789	0.36290438	0.56379809
Chchd7	112.615893	1.01697923	0.02429022	0.17257711	0.81772477	0.90164545
Chd1	13953.7056	-1.118185	-0.1611589	0.06199183	0.00634644	0.02681328
Chd1l	1000.47996	1.18411861	0.2438136	0.07277683	0.00037637	0.00253755
Chd2	2708.19626	-1.012414	-0.0177994	0.07371138	0.79669149	0.88705936
Chd3	3705.38247	1.10910872	0.1494008	0.06645106	0.01802588	0.06321148
Chd4	18099.568	-1.0639796	-0.0894705	0.04467388	0.0430547	0.12458341
Chd6	6140.8627	1.54810689	0.63050508	0.06186516	2.51E-25	2.08E-23
Chd7	1691.06957	-1.1128237	-0.1542251	0.07481222	0.0274822	0.08746508
Chd8	7626.50252	-1.1276603	-0.1733326	0.04803916	0.00020679	0.0015374

Chd9	4268.79722	1.04314941	0.06094581	0.05248888	0.23118475	0.42030609
Chek1	2460.33547	-1.0760975	-0.1058088	0.06919626	0.10542505	0.24237166
Chek2	453.939566	1.09265627	0.12783962	0.1025824	0.15693203	0.32200854
Cherp	1829.28469	-1.0223104	-0.0318333	0.07655017	0.65562889	0.8004869
Chfr	1479.04073	1.08988114	0.12417081	0.06169002	0.03528565	0.10658763
Chic1	69.3857748	1.10564472	0.14488788	0.18861761	0.22531768	0.41392794
Chic2	481.819568	-1.0723944	-0.1008356	0.10842702	0.28037648	0.47608996
Chid1	1448.79077	1.06497236	0.09081599	0.08085328	0.22456363	0.41301653
Chka	418.086375	1.09264997	0.12783131	0.10488494	0.16279025	0.33025443
Chkb	148.384276	1.16109287	0.21548337	0.16361269	0.07666308	0.19203246
Chl1	39.4215322	-1.0517967	-0.0728559	0.20849104	0.40118789	0.60084279
Chm	432.211433	-1.0756934	-0.105267	0.10353069	0.24564094	0.43588591
Chmp1a	1517.56416	1.07088782	0.09880736	0.06786875	0.12424772	0.27253652
Chmp2a	931.604424	1.22943431	0.29799465	0.10137632	0.00110455	0.00632793
Chmp2b	3158.69	1.0355194	0.05035458	0.05844635	0.37083672	0.57220169
Chmp3	2516.70331	-1.9425785	-0.9579729	0.06607897	9.00E-49	2.35E-46
Chmp4b	3902.24606	1.06977281	0.09730444	0.05673454	0.07478349	0.18832124
Chmp7	672.627579	1.00328261	0.00472805	0.08979923	0.95491264	0.97820319
Chordc1	3171.90242	-1.1435568	-0.193528	0.07313893	0.00475844	0.02125243
Chpf	4524.73707	1.01389949	0.01991464	0.07821056	0.78457713	0.87947319
Chpf2	743.271489	-1.3874007	-0.4723845	0.11808997	9.76E-06	0.00010173
Chpt1	579.893329	-1.2986812	-0.3770474	0.11384529	0.00020526	0.00152874
Chrac1	388.119363	-1.1821456	-0.2414077	0.11459261	0.01481138	0.05407063
Chrdl1	14.735845	-1.04757	-0.0670467	0.2231569	0.21751039	0.40398842
Chrnb1	40.7459688	1.02825153	0.04019321	0.18882384	0.67608239	0.81302522
Chst11	2872.7241	-1.1507039	-0.2025166	0.06091876	0.00050982	0.00331515
Chst12	569.987036	-1.1499866	-0.2016171	0.14671912	0.07815217	0.19483475
Chst2	123.602828	1.09670049	0.13316957	0.17632098	0.25705088	0.44927749
Chst8	3.982728	1.07190175	0.10017267	0.25034268	0.00177606	0.00946081
Chsy1	4189.73891	1.10244014	0.14070033	0.05056364	0.00412114	0.01893538
Chtf18	1372.1849	1.09242048	0.12752826	0.07485812	0.06861319	0.17691928
Chtop	2648.22173	-1.159618	-0.2136497	0.05111045	1.61E-05	0.00015845
Chuk	2823.05758	1.02418334	0.03447399	0.06113461	0.55697706	0.73128402
Ciao2b	437.395915	1.08352505	0.11573251	0.09637517	0.17941328	0.35456405
Ciao3	699.338076	1.07908963	0.1098147	0.0917694	0.18480935	0.36165716
Ciart	150.521732	1.35831264	0.44181558	0.2579276	0.00985042	0.03876617
Cib1	636.854389	1.07034516	0.0980761	0.10291257	0.27789748	0.47363847
Cib2	169.022473	1.33059091	0.41206708	0.2001887	0.00597043	0.02546633
Cib4	55.4150164	1.38937207	0.474433	0.40309334	0.01629296	0.05807876
Cic	2427.49387	1.0846078	0.11717345	0.07444277	0.09244529	0.22023884
Cideb	1.97824899	1.01475868	0.02113669	0.21516809	0.44581276	0.64231936
Cidec	2232.98963	-1.0039132	-0.0056345	0.06472218	0.9266301	0.96278138
Cilk1	2403.09181	1.01584064	0.0226741	0.06480535	0.7142525	0.83876308
Cilp2	6.03569831	1.00063807	0.00092024	0.2099405	0.98644854	0.99311583
Cinp	1135.50421	-1.1510622	-0.2029658	0.07384594	0.00334746	0.01598841
Cip2a	1787.59185	1.01858938	0.02657258	0.07066117	0.6917364	0.82348698
Cipc	1005.62322	-1.1601425	-0.2143021	0.08205532	0.00471137	0.02110969
Cir1	1199.04762	1.09768201	0.13446018	0.08791212	0.09349314	0.22211557
Cisd1	265.357453	1.031189	0.04430878	0.13429199	0.67594033	0.81295611
Cisd2	196.145309	1.00442483	0.0063696	0.12435179	0.95674464	0.97901352

Cisd3	1357.40707	1.00146508	0.00211211	0.09152258	0.98090807	0.99088655
Cish	123.798294	1.15497632	0.20786327	0.17414962	0.09499216	0.2247598
Cit	5030.48588	1.030002	0.04264714	0.05140913	0.392903	0.594043
Cited1	378.172428	1.83049404	0.87223307	0.14430506	1.05E-10	2.61E-09
Cited2	311.916796	-1.4920967	-0.577341	0.16234651	4.05E-05	0.00036189
Ciz1	545.673245	1.07030424	0.09802095	0.08965871	0.22907201	0.41766719
Ckap2	4393.66678	1.16400139	0.21909278	0.0632049	0.0002817	0.00198383
Ckap2l	2115.76087	1.15260181	0.2048942	0.07899643	0.00526337	0.02305875
Ckap4	15785.422	-1.1204793	-0.164116	0.04118454	4.97E-05	0.00043568
Ckap5	10673.5991	-1.0248571	-0.0354228	0.04320178	0.41192971	0.61109665
Cklf	518.036253	-1.4499528	-0.536006	0.11979992	9.73E-07	1.26E-05
Ckmt1	8.35366883	-1.0720998	-0.1004393	0.24401725	0.06142889	0.16308893
Cks2	378.430386	-1.0539728	-0.0758376	0.1466071	0.48739322	0.67570424
Clasp1	2730.06691	1.01382066	0.01980247	0.0609102	0.73546949	0.85207236
Clasp2	3396.78427	1.13179005	0.17860636	0.05221295	0.00040729	0.00271596
Clasrp	398.41202	1.01128544	0.01619027	0.11344443	0.8681344	0.92874575
Clba1	33.6803078	1.10183042	0.1399022	0.24284942	0.16573213	0.33465142
Clcc1	1208.07768	-1.0385123	-0.0545183	0.0662612	0.38633295	0.58747924
Clcf1	278.213356	-1.0878451	-0.1214732	0.11377698	0.21008114	0.39534882
Clcn1	4.62984267	-1.0612003	-0.085697	0.23954615	0.01307196	0.04869199
Clcn2	154.041603	-1.0981995	-0.1351401	0.15311288	0.23483642	0.42465478
Clcn3	2362.28963	-1.0257744	-0.0367134	0.05795936	0.51072656	0.69467055
Clcn4	345.726376	-1.1358577	-0.1837821	0.1094576	0.05245007	0.14420237
Clcn5	23.9785359	-2.4489925	-1.2921883	0.53035005	0.00055385	0.0035599
Clcn6	635.1048	1.32329381	0.40413341	0.09055338	1.73E-06	2.13E-05
Clcn7	467.748856	1.13999788	0.18903114	0.10740624	0.04376174	0.12607469
Cldn12	219.754864	-1.0279402	-0.0397564	0.15215317	0.71848426	0.84156113
Cldn15	13.9416198	1.0361034	0.05116799	0.21303521	0.42881864	0.62657315
Cldn6	11.1632323	-1.067009	-0.0935723	0.23743283	0.1039931	0.24032874
Cldnd1	2798.77486	-1.0790257	-0.1097292	0.07168518	0.10331051	0.23909803
Clec16a	1930.50883	-1.0154226	-0.0220803	0.06747002	0.72986764	0.84849845
Clec2l	2.80151104	-1.0156677	-0.0224285	0.21486731	0.47556435	0.66637271
Clec3b	1.88380014	-1.0178675	-0.0255498	0.21597021	0.34783528	0.54741474
Clhc1	205.997498	1.10926822	0.14960825	0.14049461	0.17485867	0.34819519
Clic4	6658.54122	-1.0970345	-0.1336089	0.04749552	0.00375404	0.01755084
Clic5	1.74941726	-1.019105	-0.0273027	0.21777131	0.06962793	0.17862946
Clint1	1737.51646	-1.0451766	-0.0637468	0.07443992	0.35966421	0.559874
Clip1	5937.49782	1.03433794	0.04870762	0.04610044	0.27836972	0.47419097
Clip2	1176.12492	-1.0683265	-0.0953527	0.06975138	0.14744057	0.30790898
Clk1	1504.57474	-1.3117413	-0.3914832	0.07365773	2.44E-08	4.19E-07
Clk2	879.177623	1.09447942	0.13024482	0.08788573	0.10411015	0.24040971
Clk3	1528.5394	-1.185918	-0.2460042	0.06450844	6.28E-05	0.0005375
Clk4	408.622975	-1.1625932	-0.2173464	0.12246894	0.03471665	0.10535899
Clmp	146.886086	-1.0141644	-0.0202916	0.13532105	0.84612907	0.91707534
Cln3	218.986081	1.00830386	0.01193046	0.12333894	0.90825345	0.95230265
Cln5	2059.03382	1.32790146	0.40914809	0.08188329	1.26E-07	1.91E-06
Cln6	473.20722	-1.0312723	-0.0444254	0.09943871	0.61332531	0.77269695
Cln8	364.032796	-1.061231	-0.0857388	0.10371162	0.34459351	0.54402797
Clns1a	2643.94819	-1.065752	-0.0918717	0.05939969	0.10451409	0.24096932
Clock	3679.87516	-1.1572977	-0.2107601	0.06389106	0.00053502	0.00345447

Clpb	1853.8193	1.05759379	0.08078561	0.06002949	0.16092029	0.3273085
Clpp	898.224667	1.02767837	0.03938882	0.09042782	0.6337797	0.7864639
Clptm1	1588.68792	-1.0321793	-0.0456936	0.07488306	0.51376506	0.69754429
Clptm1l	3368.96137	1.03169705	0.0450194	0.05295444	0.38051307	0.58257111
Clpx	1239.54361	1.20440442	0.26831991	0.06691933	2.55E-05	0.0002375
Clrn1	3.86151021	-1.0172646	-0.024695	0.21387994	0.51957708	0.702309
Clspn	4516.19606	1.01589766	0.02275508	0.0534541	0.66080099	0.80365145
Clstn1	2695.57691	1.10274414	0.14109809	0.05891529	0.01254887	0.04714822
Clstn3	4.20627219	-1.0563091	-0.0790321	0.23479567	0.03035949	0.09469322
Clta	1621.12724	-1.0179832	-0.0257138	0.07907722	0.72598374	0.84559481
Cltb	478.547815	-1.0231615	-0.0330338	0.11862573	0.73768429	0.85348047
Cltc	18769.8623	-1.0344239	-0.0488275	0.04248389	0.24025441	0.42993254
Clu	2350.99614	1.12905761	0.17511911	0.08393524	0.02328365	0.07739065
Cluh	2942.83626	-1.081366	-0.1128549	0.05926123	0.04690607	0.13263923
Clybl	293.340328	1.00305436	0.0043998	0.1180594	0.96540853	0.98312856
Cmah	31.4053412	-1.4824625	-0.5679956	0.65812142	0.01230095	0.04646295
Cmas	3608.19853	-1.0555638	-0.0780138	0.06458545	0.20411009	0.38781522
Cmc1	148.464705	1.09871706	0.13581992	0.19399909	0.24847941	0.43946171
Cmc2	610.929691	-1.0367631	-0.0520863	0.08794932	0.51621	0.6996773
Cmc4	12.7633119	-1.0154035	-0.0220531	0.20936086	0.68633308	0.8196191
Cmip	1117.72049	-1.0997079	-0.1371204	0.09193606	0.09821493	0.22996403
Cmpk1	2443.009	-1.1217698	-0.1657766	0.05731309	0.00260862	0.01305838
Cmss1	678.775097	-1.0514652	-0.0724011	0.09477233	0.39378953	0.59478601
Cmtm3	134.439308	1.01277857	0.01831879	0.14671954	0.86600269	0.92774752
Cmtm4	81.1338223	1.0977142	0.13450248	0.20858837	0.23970245	0.42939023
Cmtm6	2948.57499	-1.0677023	-0.0945094	0.06445813	0.12385029	0.27208408
Cmtm7	1567.13986	1.03775443	0.05346509	0.05701774	0.330718	0.5307564
Cmtr1	1451.71166	1.11323284	0.15475538	0.07753566	0.03201981	0.09878943
Cmtr2	814.894963	-1.2911882	-0.3686993	0.08726271	5.73E-06	6.21E-05
Cmya5	20.5592525	1.03756237	0.05319807	0.20203452	0.5358113	0.71467542
Cndp2	4142.07477	-1.0153637	-0.0219965	0.05300576	0.66787332	0.80811765
Cnep1r1	98.742862	1.04377502	0.06181078	0.1585289	0.57422177	0.74210864
Cnga4	5.26696	1.01497234	0.02144041	0.21205763	0.63504512	0.78732309
Cnih2	7.04720393	1.06192227	0.08667817	0.23763123	0.04696814	0.13267075
Cnih4	2838.01092	-1.0364896	-0.0517057	0.06622098	0.41068094	0.60980907
Cnksr1	417.261944	-1.7343327	-0.7943807	0.13409679	2.49E-10	5.77E-09
Cnksr2	626.206883	1.35140959	0.434465	0.09558363	1.02E-06	1.31E-05
Cnksr3	744.485695	-1.3531894	-0.4363638	0.0883008	1.43E-07	2.15E-06
Cnn1	6.09972503	1.03454852	0.04900131	0.21800866	0.3056595	0.50373063
Cnn2	3345.49626	1.20378853	0.26758198	0.06623192	2.23E-05	0.00021054
Cnn3	11131.4594	-1.0901799	-0.1245662	0.04575096	0.00526945	0.02307747
Cnnm2	498.727196	-1.0732608	-0.1020007	0.1019124	0.25465926	0.44629303
Cnnm3	274.016402	1.0120546	0.01728712	0.11805228	0.86155942	0.92612034
Cnnm4	2246.81614	-1.0319067	-0.0453126	0.07046615	0.49546866	0.68188156
Cnot1	16724.0286	1.00208625	0.00300669	0.03750685	0.93564367	0.96890674
Cnot10	2086.15109	1.02942457	0.04183812	0.06280321	0.48625211	0.67499887
Cnot11	1819.85646	-1.0682738	-0.0952814	0.06607954	0.12817291	0.27838166
Cnot2	3508.2169	-1.0627605	-0.0878166	0.0592765	0.12260599	0.26995234
Cnot3	1131.31137	-1.0170579	-0.0244019	0.06871267	0.70733311	0.83449412
Cnot4	1516.87654	1.01077289	0.01545887	0.05656656	0.77825122	0.87658473

Cnot6	2307.13002	-1.2750886	-0.3505974	0.07754601	1.55E-06	1.92E-05
Cnot6l	1926.53312	1.0314178	0.04462884	0.06525542	0.47327963	0.66426478
Cnot7	2799.25766	1.00646022	0.00929015	0.0610602	0.87477919	0.93258334
Cnot8	956.328455	-1.0040931	-0.0058931	0.07967663	0.93577784	0.96890674
Cnot9	4394.90476	-1.208053	-0.2726837	0.05074084	3.21E-08	5.41E-07
Cnppd1	1170.24867	1.01069685	0.01535034	0.07706857	0.83167094	0.90953575
Cnpy2	1599.17132	1.07937031	0.11018991	0.06895217	0.0952933	0.22518119
Cnpy3	1248.18381	1.28664295	0.36361176	0.10106531	7.88E-05	0.00066068
Cnpy4	742.755256	1.12466007	0.16948901	0.09913129	0.05450358	0.14839339
Cnrip1	1087.89236	-1.1606883	-0.2149806	0.08123963	0.0042182	0.01932989
Cnst	388.608711	-1.0001884	-0.0002717	0.09599022	0.99664218	0.99860486
Cntd1	16.7325074	-1.0324554	-0.0460795	0.20421971	0.55598571	0.73070338
Cntf	5.75216515	1.01045538	0.01500561	0.21068937	0.75360269	0.86298066
Cntln	1645.36554	1.13880951	0.18752645	0.09338963	0.0260404	0.08404574
Cntn1	494.801355	1.71501344	0.77821988	0.11705205	2.71E-12	8.27E-11
Cntnap1	701.568494	1.22627688	0.29428476	0.09942152	0.00103054	0.00598966
Cntrl	5688.72766	-1.0580822	-0.0814517	0.05709862	0.13823638	0.29388337
Cntrob	952.394769	1.26239098	0.3361588	0.08002748	7.59E-06	8.07E-05
Coa3	481.101748	1.26006008	0.33349253	0.10169129	0.00029335	0.00204897
Coa4	69.9586918	1.13481166	0.18245288	0.23511807	0.13857252	0.29433161
Coa5	174.679695	-1.0433897	-0.061278	0.16556916	0.57420541	0.74210864
Coa6	79.2811266	-1.2884576	-0.3656451	0.31126371	0.02731541	0.0871074
Coa7	1120.22101	-1.1314889	-0.1782224	0.08263658	0.01919136	0.06624666
Coasy	1152.19067	1.04318335	0.06099275	0.07583476	0.39017056	0.59162937
Cobl	8.06380158	1.03544976	0.05025756	0.21572291	0.37667585	0.57856327
Cobll1	5642.34328	1.22444088	0.29212312	0.06566049	3.15E-06	3.66E-05
Cog1	836.645136	1.29755745	0.37579841	0.08892496	5.63E-06	6.12E-05
Cog2	876.090954	1.08240664	0.1142426	0.0767961	0.11030596	0.25057977
Cog3	1025.63804	1.09138874	0.12616507	0.08241594	0.09694364	0.22798111
Cog4	536.413629	-1.0013302	-0.0019179	0.09394259	0.98115145	0.99097611
Cog5	978.609642	1.43598252	0.52203818	0.08603858	1.89E-10	4.52E-09
Cog6	2601.08002	1.17346017	0.23076887	0.05664565	2.34E-05	0.00021989
Cog7	1248.69587	-1.0088324	-0.0126866	0.06743695	0.8421896	0.9153694
Cog8	313.970153	-1.1727994	-0.2299563	0.13183872	0.03379189	0.1032127
Coil	755.933784	1.04115305	0.05818217	0.09224763	0.48541885	0.67442685
Col11a1	11.3924046	1.08943658	0.12358221	0.25611908	0.07450667	0.18784303
Col11a2	12.5946534	1.04368791	0.06169037	0.21788457	0.32428754	0.52374204
Col12a1	3757.51002	-1.0753314	-0.1047814	0.0703615	0.11378407	0.2557596
Col14a1	10.3706354	-1.0096768	-0.0138935	0.21140557	0.74638566	0.85912483
Col16a1	765.659308	1.83812849	0.87823762	0.09523172	2.40E-21	1.56E-19
Col17a1	30.9462621	-3.0735069	-1.6198857	0.49745012	4.59E-05	0.00040535
Col18a1	37.1801963	1.12078624	0.16451115	0.24626621	0.14665618	0.30677995
Col24a1	2.09513129	-1.0440986	-0.062258	0.22765993	0.0216757	0.07316689
Col3a1	1865.48094	-1.4825332	-0.5680644	0.07438643	2.84E-15	1.16E-13
Col4a1	13.6804043	-1.0600489	-0.0841308	0.22358155	0.25190074	0.44320042
Col4a5	1912.76807	1.10541029	0.14458195	0.05762046	0.00900427	0.03583246
Col5a1	10960.8574	1.85627794	0.89241274	0.08344665	7.59E-28	7.00E-26
Col5a2	10299.5993	1.26718599	0.34162829	0.0622019	1.14E-08	2.07E-07
Col5a3	3.05371937	1.00492859	0.00709299	0.21336401	0.81946907	0.90269144
Col6a1	187.339798	2.34303928	1.22838114	0.18900005	4.95E-12	1.47E-10

Col6a2	1.93573055	-1.0203576	-0.0290748	0.21811509	0.05469804	0.14876472
Col8a1	600.723279	-3.8276726	-1.9364674	0.12806232	4.87E-53	1.56E-50
Col8a2	1.99255881	-1.0077428	-0.0111274	0.21397659	0.69398425	0.82489916
Col9a3	2.27486455	-1.0118669	-0.0170195	0.2151328	0.46754879	0.660123
Colca2	17.3873353	1.01570992	0.02248844	0.20416701	0.74620802	0.85906591
Colgalt1	3581.86385	1.08957065	0.12375975	0.07028457	0.06155603	0.16332492
Commd10	277.068225	1.12277716	0.16707162	0.1427334	0.13517118	0.28926543
Commd2	532.402617	-1.026607	-0.037884	0.1162579	0.69765068	0.82741461
Commd3	889.626717	1.16169149	0.21622698	0.07760268	0.00281015	0.01387207
Commd4	1107.38479	1.2085335	0.27325746	0.0848182	0.000493	0.0032123
Commd6	668.261366	1.11920886	0.16247929	0.10434461	0.07579954	0.19023208
Commd8	1719.64613	1.1961818	0.25843668	0.07935113	0.00048073	0.00314188
Comt	4105.94723	1.05351666	0.07521312	0.04934487	0.1177769	0.26220406
Comtd1	210.832498	1.00912817	0.01310942	0.13241254	0.90111879	0.94874637
Cop1	2725.20089	1.04757338	0.06705131	0.06041	0.24753014	0.43833727
Copa	6511.83224	1.09232907	0.12740754	0.04745766	0.0056541	0.02442902
Copb1	4087.10746	1.02215337	0.03161169	0.06148555	0.59081217	0.75404043
Copb2	9586.76218	1.07939448	0.11022222	0.05673164	0.04398879	0.12648627
Copg1	5377.19039	1.07611754	0.10583567	0.04987925	0.02946947	0.0923896
Copg2	1677.13952	1.27161384	0.34666062	0.07287469	5.47E-07	7.36E-06
Coprs	500.964869	1.01305799	0.01871676	0.10847964	0.84260179	0.91543734
Cops2	6251.75927	-1.0487107	-0.0686168	0.06615704	0.27479496	0.47009574
Cops3	1274.64157	-1.1194316	-0.1627664	0.0984935	0.06248156	0.16499459
Cops4	3790.2556	-1.1278625	-0.1735912	0.06184611	0.00328284	0.01573846
Cops5	2302.28121	-1.0508284	-0.071527	0.0723667	0.29353398	0.49075571
Cops6	2043.64219	-1.0049074	-0.0070626	0.07251501	0.91750191	0.95706088
Cops7a	1769.23684	1.19462483	0.25655762	0.06280153	1.94E-05	0.00018665
Cops7b	1007.6201	1.0301828	0.04290036	0.07364978	0.53549134	0.71432303
Cops8	3196.04642	1.01371105	0.01964648	0.07449915	0.77909055	0.87673482
Cops9	55.4795642	-1.1375088	-0.1858777	0.24089111	0.12898601	0.27970309
Copz1	2380.20579	1.01616432	0.02313371	0.06618636	0.71382212	0.83851446
Coq10a	507.319957	1.07226671	0.1006638	0.10514903	0.27322244	0.46897755
Coq10b	1311.8422	-1.0163981	-0.0234656	0.08085125	0.75301089	0.86298066
Coq2	612.068791	1.05043356	0.07098492	0.09152409	0.39170733	0.59313526
Coq3	793.844371	1.01579739	0.02261267	0.09050738	0.78382762	0.87922895
Coq4	284.965908	1.06086548	0.08524173	0.12577117	0.40676895	0.60628124
Coq6	253.685534	1.00055503	0.00080052	0.11745077	0.99149557	0.9959271
Coq7	740.408064	1.09407734	0.12971473	0.09138866	0.11708733	0.26125915
Coq8a	60.5203845	-1.0390591	-0.0552777	0.18011771	0.59543333	0.75857792
Coq8b	113.700043	1.69028741	0.75726858	0.24041913	0.00012233	0.0009709
Coq9	463.85682	1.15034219	0.20206307	0.10901528	0.0331252	0.10156386
Corin	2.81699819	-1.0298993	-0.0425033	0.21895658	0.21602012	0.40217945
Coro1b	3262.80005	1.17942012	0.23807771	0.06690464	0.00018226	0.00138562
Coro1c	4437.55119	-1.0818387	-0.1134854	0.06263248	0.05748255	0.15479326
Coro7	895.424939	1.03851554	0.05452281	0.08939092	0.50295012	0.68781798
Cotl1	688.184559	1.1570423	0.2104416	0.11991143	0.03837265	0.11386465
Cox10	636.473559	-1.0381779	-0.0540537	0.08592094	0.49163411	0.67886526
Cox11	1023.10572	1.00101762	0.00146737	0.07111045	0.98327387	0.99140011
Cox14	183.7273	1.16504835	0.22038983	0.19222263	0.08910087	0.21468182
Cox15	1078.38598	1.21915943	0.2858868	0.09038087	0.00055526	0.00356533

Cox16	463.381579	1.1365372	0.1846449	0.10867389	0.05094034	0.14102999
Cox18	175.899578	1.02989214	0.04249325	0.14429152	0.6958317	0.82617519
Cox19	79.153071	-1.0331832	-0.047096	0.18814749	0.62878709	0.78300404
Cox20	531.321134	1.20022118	0.26330029	0.11694094	0.00922384	0.03656992
Cox4i1	5875.52594	-1.0254828	-0.0363033	0.0556588	0.49977737	0.68560164
Cox5a	4598.79551	-1.0699768	-0.0975795	0.05947703	0.09097327	0.21785424
Cox6a1	2838.24492	1.07350409	0.10232769	0.05980936	0.07453752	0.18788382
Cox6c	2436.15218	-1.1313464	-0.1780407	0.09618342	0.03853296	0.11426094
Cox7a1	18.6537133	-1.0580332	-0.0813849	0.21677328	0.31961727	0.51806873
Cox7c	1030.2601	-1.0142273	-0.020381	0.09992394	0.8151649	0.89972966
Cox8a	51.4291041	-1.0688143	-0.0960112	0.20396174	0.34700125	0.54664894
Cp	7.69650825	1.02334779	0.03329654	0.21286041	0.51260887	0.69669678
Cpd	6568.09138	1.04989978	0.07025161	0.06642035	0.26587192	0.4598109
Cpeb1	912.88133	1.13974657	0.18871307	0.07303519	0.00588778	0.02520957
Cpeb2	720.476472	1.01660688	0.0237619	0.08642653	0.76465589	0.86962346
Cpeb3	95.8782246	1.09443159	0.13018178	0.17672075	0.26649134	0.46063339
Cpeb4	603.156386	1.10417566	0.1429697	0.09814196	0.10205602	0.23671058
Cped1	1391.95527	-1.0078318	-0.011255	0.08273405	0.88203675	0.9368121
Cplane1	3368.51941	1.06368507	0.08907107	0.05401672	0.0880758	0.21307855
Cplane2	68.6950001	1.4173008	0.50314598	0.49746247	0.01492644	0.05435126
Cpm	2.52476809	-1.0457338	-0.0645156	0.22883154	0.01455616	0.05327556
Cpn1	42.6974224	-1.0982973	-0.1352686	0.22943705	0.1981342	0.37899047
Cpne1	9814.49184	1.07944559	0.11029052	0.05116436	0.02601582	0.08400875
Cpne2	1272.72639	1.0972451	0.13388583	0.07221839	0.04854607	0.13622675
Cpne3	1535.60965	1.00083998	0.00121133	0.06054372	0.98393875	0.99144618
Cpne5	2.7526709	1.03250753	0.0461523	0.22076877	0.13928123	0.29551304
Cpne8	2053.21036	1.01576084	0.02256076	0.05989022	0.69462057	0.82527263
Cpox	3902.19395	-1.2028184	-0.2664188	0.0589439	2.61E-06	3.09E-05
Cpped1	1299.76349	1.08489675	0.11755775	0.08178588	0.11973236	0.26522073
Cps1	2.25652377	-1.0044661	-0.0064289	0.2143506	0.77379239	0.87386763
Cpsf1	3261.93833	-1.0067139	-0.0096537	0.06348104	0.8722861	0.93093268
Cpsf2	9032.19332	1.00922602	0.01324931	0.04257191	0.75165409	0.86239936
Cpsf3	2905.74353	1.08939867	0.12353201	0.06336409	0.04081866	0.11957169
Cpsf4	992.711754	1.07502706	0.10437298	0.07602636	0.14121952	0.29866634
Cpsf6	5498.63276	-1.0340111	-0.0482517	0.05314853	0.35097816	0.5506467
Cpsf7	2598.60522	1.07862863	0.10919824	0.05412074	0.03680641	0.11027603
Cpt1a	1724.25694	1.13510842	0.18283011	0.06563516	0.00332797	0.01592744
Cpt1b	102.165362	1.02829082	0.04024835	0.1709555	0.70685681	0.83416267
Cpt1c	102.792089	-1.3833838	-0.4682015	0.2456119	0.00615763	0.02611032
Cpt2	1955.95922	-1.0092945	-0.0133472	0.06858368	0.83653507	0.91223413
Cptp	387.865599	1.12220923	0.16634169	0.11632705	0.09195974	0.21943286
Cr1l	3608.4208	1.12509654	0.1700488	0.0582564	0.00235127	0.01195229
Cradd	435.510804	-1.1727549	-0.2299015	0.1338324	0.03594811	0.10814827
Cramp1l	950.347983	-1.1148103	-0.1567983	0.09010712	0.05481019	0.1490381
Crat	898.845646	1.25477152	0.32742469	0.10858765	0.00071942	0.004414
Crbn	1619.58677	-1.0297304	-0.0422667	0.06097881	0.46929002	0.66135814
Crcp	252.96805	-1.0305777	-0.0434533	0.12557068	0.66991205	0.80967875
Creb1	980.524332	-1.1140793	-0.1558519	0.07465741	0.02545448	0.08271738
Creb3l1	5600.2458	2.00929924	1.00669243	0.07596509	3.32E-41	5.53E-39
Crebbp	1116.70782	-1.2591498	-0.3324499	0.081189	1.25E-05	0.00012649

Crebl2	42.6079457	1.03993197	0.05648916	0.20095958	0.52432659	0.70612107
Crebrf	307.560373	1.44349595	0.52956706	0.15160126	6.09E-05	0.00052376
Crebzf	295.321085	-1.1300127	-0.176339	0.1367931	0.10626489	0.24386479
Creg1	1611.31321	1.02161938	0.0308578	0.07335533	0.65469088	0.79986554
Creld1	1721.79844	-1.0159277	-0.0227978	0.08552839	0.7702889	0.87232885
Creld2	2607.13634	1.25912497	0.33242147	0.0607494	1.37E-08	2.44E-07
Crem	358.657743	-1.1646721	-0.2199238	0.1245931	0.0351509	0.10632421
Crhbp	1.65933793	1.01616934	0.02314084	0.21619221	0.30770966	0.50605968
Crim1	24992.7283	1.52415353	0.60800823	0.04362551	4.95E-45	1.11E-42
Crk	2693.18556	-1.0953556	-0.1313993	0.0830072	0.08471628	0.20666836
Crkl	2971.66325	-1.0186101	-0.026602	0.05776395	0.63249539	0.78556002
Crls1	260.124284	-1.1054013	-0.1445703	0.14459096	0.19391355	0.37353874
Crocc	144.88986	1.11040567	0.15108684	0.19021826	0.20983894	0.39519079
Crocc2	2.56966506	-1.0405482	-0.0573438	0.22550693	0.03943028	0.11639903
Crot	1039.24883	1.07452081	0.10369342	0.07356035	0.13266898	0.28510143
Crppa	21.9565441	1.07792	0.10825011	0.22936105	0.21845612	0.40521618
Crtap	3081.373	1.29772975	0.37598998	0.06920105	1.36E-08	2.43E-07
Crtc1	796.708708	1.10256276	0.14086077	0.07881532	0.05359209	0.1464721
Crtc2	790.918317	1.12838511	0.17425954	0.10223472	0.05614702	0.151875
Crtc3	1575.04844	1.10229165	0.14050599	0.06278877	0.01891451	0.06550282
Cry1	1357.60618	1.10549672	0.14469475	0.07383464	0.03630266	0.10901011
Cry2	617.853253	1.21926128	0.28600732	0.0912705	0.00061491	0.00387647
Cryab	10.7174605	-1.0279595	-0.0397834	0.21037117	0.52492691	0.70665744
Cryba1	290.711496	1.18525223	0.2451941	0.15363924	0.03991217	0.11753446
Crybg1	393.886781	1.5243863	0.60822855	0.10902328	2.71E-09	5.42E-08
Cryl1	146.651745	1.11486569	0.15686992	0.16489876	0.18383923	0.36041089
Cryz	529.058396	1.16334019	0.21827304	0.12661866	0.0388286	0.1149779
Cryzl1	781.382169	1.09921951	0.13647952	0.08422953	0.07721516	0.19300023
Cryzl2	233.990779	-1.0587964	-0.0824252	0.11785759	0.39632214	0.59678931
Csad	796.738128	-1.1207228	-0.1644295	0.0799249	0.02606112	0.08409142
Csdc2	2.16227083	-1.0078098	-0.0112234	0.2144552	0.64212853	0.79161344
Csde1	27988.3988	1.06331196	0.08856493	0.05024057	0.0695581	0.17853207
Cse1l	15919.9898	1.39991637	0.48534065	0.04719155	1.40E-25	1.17E-23
Csf1	1857.12059	1.10393674	0.1426575	0.07319783	0.03770412	0.11245395
Csf1r	1.88007022	-1.0208722	-0.0298022	0.21699017	0.25840797	0.45067475
Csf3	6.74492932	-1.0629748	-0.0881073	0.2390729	0.03801408	0.11311359
Csgalnact1	30.1461478	786.833284	9.61991418	2.82721254	1.07E-13	3.81E-12
Csk	1298.83167	1.01144846	0.01642281	0.07776296	0.82148399	0.90376956
Csnk1a1	5541.49497	-1.1107665	-0.1515556	0.04746541	0.00103764	0.00601726
Csnk1d	7609.78164	-1.092071	-0.1270667	0.04773616	0.00249138	0.01256479
Csnk1e	3052.02933	-1.1151891	-0.1572884	0.06227288	0.00810059	0.03279665
Csnk1g2	519.160562	-1.055531	-0.0779689	0.10018161	0.37803513	0.58022942
Csnk1g3	888.978017	-1.0556613	-0.078147	0.07267298	0.25212123	0.44337103
Csnk2a1	3913.39529	1.06602324	0.09223889	0.05262875	0.0703813	0.17988517
Csnk2a2	4476.57395	1.02730798	0.03886876	0.05269254	0.44740504	0.64330021
Csnk2b	2663.57658	-1.1284937	-0.1743983	0.05213576	0.00054479	0.003514
Cspg4	6289.52704	-1.2051993	-0.2692718	0.06143416	4.80E-06	5.32E-05
Cspg5	8.39598466	-1.0114291	-0.0163951	0.21029275	0.73797119	0.85372044
Cspp1	513.989129	-1.1876894	-0.2481575	0.1078623	0.0088194	0.03526107
Csrnp1	358.106348	-1.2829668	-0.3594839	0.12737651	0.00108703	0.00624993

Csrnp2	304.453831	1.07175839	0.09997971	0.12910625	0.33826557	0.53779382
Csrp1	4466.29399	1.06605642	0.09228379	0.05249358	0.06955922	0.17853207
Csrp2	584.216183	-1.3418362	-0.4242086	0.10772454	1.56E-05	0.00015477
Cst6	17.1688361	-1.0136935	-0.0196215	0.20474328	0.7651303	0.86999105
Cstb	2540.2767	1.07587826	0.10551484	0.06729987	0.09768654	0.22914365
Cstf1	1294.64162	1.20638963	0.27069593	0.07927007	0.00025095	0.00180628
Cstf2	2457.37355	1.05470572	0.07684052	0.05723671	0.16363454	0.33146086
Cstf2t	2256.53587	-1.2212355	-0.2883415	0.05831355	2.88E-07	4.08E-06
Cstf3	773.498091	-1.1751359	-0.2328276	0.08615833	0.00327355	0.01571396
Ctbp1	2872.85793	-1.1063483	-0.1458056	0.0608258	0.01225253	0.04631304
Ctbp2	1543.86094	-1.1012424	-0.1391321	0.07654013	0.05093589	0.14102999
Ctbs	380.331306	1.12075998	0.16447734	0.12264163	0.10847905	0.24730675
Ctc1	478.663619	1.03570049	0.05060686	0.10498311	0.58185272	0.74773936
Ctcf	5437.4508	-1.0647383	-0.0904989	0.05182321	0.07147432	0.18184429
Ctdnep1	686.289877	1.3680203	0.45208964	0.11137941	8.45E-06	8.90E-05
Ctdp1	1261.03687	-1.0842809	-0.1167385	0.08122228	0.11922581	0.26451032
Ctdsp1	1353.18439	1.14993414	0.20155123	0.07299569	0.00325636	0.01564665
Ctdsp2	3910.28005	-1.0390257	-0.0552313	0.06675951	0.38299629	0.58441721
Ctdspl	1969.54796	1.33538264	0.41725319	0.06016736	8.81E-13	2.85E-11
Ctdspl2	1431.51156	1.00234348	0.00337697	0.07079518	0.96037005	0.98128264
Ctif	221.049464	-1.0232655	-0.0331805	0.13661252	0.76784979	0.8713089
Ctnna1	5249.20489	1.00108667	0.00156688	0.05558112	0.97705436	0.98941236
Ctnna2	163.140735	-1.3842938	-0.4691501	0.20005222	0.00241423	0.01224319
Ctnnal1	843.546881	-2.2808798	-1.1895904	0.12855701	1.42E-21	9.36E-20
Ctnnb1	12941.1078	-1.0105621	-0.015158	0.04126097	0.70885446	0.83556144
Ctnnbip1	29.4560069	1.22206584	0.28932201	0.3969274	0.0393998	0.11634658
Ctnnbl1	4179.37885	1.24948146	0.32132949	0.05470679	1.39E-09	2.87E-08
Ctns	79.3190553	1.49988431	0.58485122	0.33143297	0.00543988	0.02368162
Ctps	5069.62819	1.06806537	0.09499994	0.05682411	0.08302488	0.2033554
Ctps2	923.498547	1.34810969	0.43093789	0.07438575	1.38E-09	2.86E-08
Ctr9	4066.59249	-1.1032885	-0.1418102	0.04564514	0.00143862	0.00786543
Ctsa	6510.88235	1.7683286	0.82238639	0.04953826	5.88E-63	2.90E-60
Ctsb	2703.56416	1.04510686	0.06365046	0.07376816	0.35788555	0.55790704
Ctsf	845.56074	1.68852366	0.7557624	0.09030569	5.39E-18	2.80E-16
Ctsh	1142.68874	1.02959759	0.04208058	0.09108102	0.61110672	0.77118285
Ctsk	19.7477102	-1.0388092	-0.0549307	0.20077042	0.53127859	0.71144456
Ctsl	4183.22236	-1.1023091	-0.1405288	0.06649103	0.02573583	0.08337785
Ctso	157.517719	-1.1092968	-0.1496455	0.1553898	0.19352731	0.37301909
Ctsw	4.02887785	-1.032934	-0.0467481	0.21967156	0.21353756	0.39933082
Cttn	8934.44097	-1.0064632	-0.0092944	0.04769611	0.84113588	0.91487863
Cttnbp2	150.025702	-1.0338949	-0.0480896	0.13118225	0.64393007	0.79261499
Cttnbp2nl	3217.4766	-1.1074818	-0.147283	0.04925598	0.00207245	0.01072652
Ctu2	280.613468	-1.0708819	-0.0987994	0.12516845	0.33887926	0.53850274
Cubn	1319.20085	1.2267303	0.29481811	0.07596587	3.68E-05	0.00033216
Cuedc1	113.040784	1.05268247	0.07407034	0.15081715	0.50039376	0.68583839
Cuedc2	2140.71664	-1.0959248	-0.1321488	0.06478482	0.03128655	0.09700996
Cul1	4715.15991	-1.0418853	-0.0591964	0.04798816	0.20629723	0.39069597
Cul2	2563.81622	1.06984349	0.09739975	0.07228113	0.15193814	0.31407578
Cul3	8118.81522	-1.0335215	-0.0475684	0.05749976	0.39611518	0.59664994
Cul4a	3005.00387	1.04455424	0.0628874	0.05689478	0.25173729	0.44314341

Cul5	8795.73906	-1.0173641	-0.024836	0.05003654	0.60864391	0.76890814
Cul7	4180.4547	1.14042313	0.1895692	0.0683893	0.0033629	0.01605019
Cuta	406.794216	1.02515863	0.03584717	0.11925657	0.71880978	0.84156113
Cutc	540.625163	1.27462904	0.35007743	0.12255976	0.00106103	0.00612666
Cux1	2633.21007	1.18262573	0.24199357	0.06877129	0.00020523	0.00152874
Cux2	3.97074287	-1.076746	-0.1066779	0.25365863	0.00897287	0.03572969
Cwc15	2551.06446	-1.031312	-0.0444809	0.07452966	0.52364443	0.70549906
Cwc25	1825.83484	-1.0748417	-0.1041242	0.06794724	0.10527068	0.24207229
Cwc27	1654.8535	-1.0150638	-0.0215705	0.07382352	0.75513379	0.86391568
Cwf19l1	429.993141	-1.0363141	-0.0514614	0.10242877	0.56430088	0.73589476
Cwf19l2	2129.40028	1.09474213	0.13059108	0.0774736	0.07019875	0.17955928
Cxcl1	112.924246	-1.3874254	-0.4724102	0.30792552	0.01081313	0.04193647
Cxcl10	7.35424566	-29.795182	-4.8970072	1.55078646	1.93E-05	0.00018515
Cxcl11	2.43289656	-1.013271	-0.0190201	0.2150855	0.46925302	0.66135814
Cxcl12	102.293297	4.43879285	2.15016738	0.37158438	2.59E-10	6.00E-09
Cxcl16	129.654966	-1.0463031	-0.0653009	0.16161478	0.55290612	0.72867565
Cxcl3	11.0248762	-7.1809299	-2.8441707	0.79063975	1.73E-05	0.00016889
Cxxc1	1023.95496	1.03738887	0.0529568	0.07888747	0.470486	0.66244511
Cxxc4	29.664676	-1.0994687	-0.1368066	0.25066202	0.13465822	0.28845681
Cxxc5	219.990306	1.22905221	0.29754621	0.14576111	0.01203282	0.0456211
Cyb561a3	493.082876	-1.051822	-0.0728906	0.08934687	0.36854603	0.5700404
Cyb561d1	393.93933	1.01469105	0.02104053	0.1062043	0.82051735	0.90334055
Cyb561d2	50.5959075	-1.0394323	-0.0557958	0.18330158	0.58580527	0.74971062
Cyb5a	1739.07024	1.05810974	0.08148926	0.08791309	0.3100732	0.50845337
Cyb5b	462.66453	-1.0949475	-0.1308618	0.11885564	0.18927183	0.36759611
Cyb5d1	380.018284	-1.0284793	-0.0405127	0.11670797	0.67940208	0.81556886
Cyb5d2	24.0098641	-1.0272285	-0.0387572	0.19892892	0.6444747	0.79305677
Cyb5r1	2738.82222	-1.0386821	-0.0547542	0.06026829	0.34315684	0.54257927
Cyb5r3	4199.34601	1.02603686	0.03708256	0.05373007	0.47640518	0.66709819
Cyb5r4	2215.37227	-1.0476108	-0.0671028	0.05436829	0.20118609	0.38362515
Cyb5rl	75.3257455	1.09078649	0.12536873	0.18035256	0.28304479	0.47911903
Cyba	196.779965	1.18416221	0.24386671	0.16222486	0.04689962	0.13263923
Cybc1	1330.93258	-1.0456743	-0.0644336	0.08271541	0.39730124	0.59750998
Cybrd1	1221.16314	1.29964649	0.37811926	0.07338551	6.23E-08	1.00E-06
Cyc1	2518.46373	1.05068234	0.07132656	0.07255868	0.29535698	0.49287733
Cyfip1	5827.82976	-1.008317	-0.0119493	0.04612976	0.79075558	0.88313513
Cyhr1	270.093521	1.03340124	0.04740052	0.11006425	0.61724695	0.77476042
Cyld	1926.13044	1.10465457	0.1435953	0.06985033	0.02925992	0.09181183
Cyp11b1	4.5200043	-1.038617	-0.0546637	0.22163986	0.18582561	0.36309569
Cyp20a1	2481.67401	-1.0071403	-0.0102647	0.05837159	0.85536449	0.92271319
Cyp24a1	2.09694073	1.00064297	0.00092732	0.21337335	0.97637584	0.98895979
Cyp26a1	3.48074973	-1.047556	-0.0670274	0.23034327	0.00691857	0.02877497
Cyp26b1	12.55644	-1.1548642	-0.2077232	0.39381412	0.00291097	0.01423482
Cyp27b1	11.1050095	-1.007767	-0.0111621	0.20647941	0.85024829	0.91935375
Cyp2c70	9.35533447	3.72922501	1.89887585	1.4534594	0.00285834	0.01403422
Cyp2d22	279.492378	1.10147496	0.1394367	0.11717163	0.15958127	0.32567047
Cyp2s1	7.47144038	-1.0405416	-0.0573347	0.21936578	0.28055461	0.47626617
Cyp2u1	395.497524	-1.2000986	-0.2631529	0.10615133	0.00502066	0.02222344
Cyp39a1	181.739496	1.05448671	0.07654091	0.13820543	0.47482657	0.665703
Cyp46a1	8.37948926	-1.0141838	-0.0203192	0.20877298	0.71654782	0.84081334

Cyp4b1	3.55217075	-1.0400944	-0.0567144	0.22352527	0.11965956	0.26519706
Cyp4f13	41.5982066	-1.1555527	-0.2085831	0.25554898	0.10304348	0.23860935
Cyp4f17	20.4548757	6.72262033	2.74902367	0.58570959	1.98E-07	2.90E-06
Cyp4v3	258.466848	-1.0199862	-0.0285497	0.12012254	0.77374469	0.87386763
Cyp4x1	19.3434328	-17.91913	-4.1634287	0.74749734	1.80E-09	3.68E-08
Cyp51	6578.07818	1.04792452	0.0675348	0.08236661	0.37580598	0.57757344
Cyren	68.657472	1.0638093	0.08923955	0.18713534	0.41301752	0.61200166
Cystm1	281.892388	1.081054	0.1124386	0.12589988	0.27711876	0.47275155
Cyth1	674.172303	1.07974272	0.11068758	0.1042374	0.22525386	0.41388915
Cyth2	472.787306	-1.0544507	-0.0764916	0.09657909	0.37481012	0.57659634
Cyth3	608.216325	-1.0142379	-0.0203962	0.08439259	0.79201569	0.88402889
D030056L22Rik	1804.38158	-1.0132127	-0.0189371	0.06326849	0.75367477	0.86298066
D10Wsu102e	645.029439	1.05880341	0.08243474	0.08612414	0.2961637	0.49378695
D11Wsu47e	316.446208	-1.0420214	-0.0593849	0.10976865	0.52345618	0.70546803
D16Ertd472e	3629.89249	-1.1508419	-0.2026897	0.06503881	0.00104139	0.00603086
D17H6S53E	278.590903	1.09750089	0.13422211	0.1280636	0.19937736	0.38079976
D1Ertd622e	275.679035	-1.1042617	-0.1430822	0.12145466	0.15721572	0.32238408
D1Pas1	6.04988528	-1.0087719	-0.0126	0.20808323	0.81973461	0.90288822
D230025D16Rik	304.237964	1.0045159	0.00650041	0.10836601	0.94555379	0.97298317
D2hgdh	634.29556	-1.0731648	-0.1018717	0.09368586	0.22591603	0.41443281
D3Ertd751e	764.869567	1.07743031	0.10759456	0.08760794	0.17757461	0.35179877
D430042O09Rik	1008.35202	1.15418765	0.2068778	0.08457328	0.00782136	0.0318473
D5Ertd579e	1536.70823	1.2428583	0.31366182	0.07215165	4.58E-06	5.11E-05
D630045J12Rik	2288.671	1.21199897	0.27738847	0.06036216	1.73E-06	2.13E-05
D6Wsu163e	435.875759	1.19845765	0.26117893	0.11001034	0.0068309	0.02844728
D8Ertd738e	333.882314	-1.0204115	-0.0291511	0.11692098	0.76570004	0.87025264
DHFR	3306.62217	1.17607998	0.23398617	0.06446885	0.00014308	0.00111281
DICER1	5396.43834	-1.1140677	-0.1558369	0.04855037	0.00095954	0.00564099
DIS3L2	624.516138	1.23367975	0.30296794	0.09109122	0.00029061	0.00203316
DNAJB9	686.615429	1.06299415	0.08813366	0.08181283	0.24312103	0.43303398
DNASE1L1	168.580842	1.38800344	0.47301114	0.18768655	0.00152319	0.00826085
DNPH1	229.965862	-1.0464395	-0.0654889	0.13221189	0.52973472	0.71049123
DOHH	516.412071	-1.0130025	-0.0186377	0.11882326	0.93801692	0.97004225
DPAGT1	11164.0919	-1.0421821	-0.0596074	0.0869589	0.46328701	0.65677783
DPM2	177.443365	1.10917371	0.14948533	0.14685388	0.18422267	0.36075211
Daam1	2017.7418	1.08178102	0.11340849	0.06139149	0.05337178	0.14608814
Dab2	2741.98715	1.24449448	0.31555983	0.0624265	1.43E-07	2.15E-06
Dab2ip	3140.19941	1.12711823	0.17263886	0.05692404	0.00160572	0.00865346
Dact3	14.9790942	1.16375275	0.21878458	0.37262597	0.02515548	0.08201621
Dag1	6559.98987	1.03748794	0.05309457	0.05044941	0.27893422	0.47465532
Dagla	487.584809	1.1402492	0.18934916	0.09847603	0.03101781	0.09632439
Daglb	548.57934	1.07130506	0.09936936	0.10043758	0.26421018	0.45767849
Dalrd3	366.114685	-1.0210824	-0.0300993	0.13068081	0.77089689	0.8726749
Dap3	2421.44915	-1.02176	-0.0310563	0.0683355	0.63092376	0.78474933
Dapk1	4.03512715	-1.039836	-0.0563561	0.22314185	0.1376275	0.29290717
Dapk3	520.990115	1.0256798	0.03658042	0.10384932	0.68809968	0.82110451
Dars	4757.31915	-1.0130968	-0.0187721	0.05254418	0.71450715	0.83894011
Dars2	1189.11456	1.17924652	0.23786534	0.07833499	0.0011326	0.00645687
Daxx	1566.17665	1.17791934	0.23624075	0.09789439	0.0074999	0.03077312
Dazap2	3204.8852	-1.0221782	-0.0316467	0.05903948	0.57658727	0.743815

Dbf4	3431.30575	-1.0281921	-0.0401099	0.06893403	0.53882152	0.71742061
Dbil5	88.6433196	-1.0416644	-0.0588906	0.1618383	0.58928962	0.75247209
Dbn1	4963.16387	-1.0524593	-0.0737645	0.04903562	0.12145795	0.26788505
Dbndd2	721.933555	1.25255745	0.32487677	0.1208819	0.00194052	0.01016691
Dbnl	1658.65757	1.51462129	0.59895711	0.08390508	1.17E-13	4.13E-12
Dbp	104.589458	1.33895482	0.42110728	0.26527078	0.01304066	0.04860367
Dbr1	1181.35527	-1.0957569	-0.1319278	0.09009258	0.10581726	0.24314244
Dbt	479.226577	-1.0381493	-0.0540139	0.08832826	0.50138185	0.68655281
Dcaf1	4160.05062	-1.0544977	-0.076556	0.04703481	0.09481933	0.2244866
Dcaf10	143.498001	1.35906403	0.44261342	0.27477366	0.01149102	0.04401911
Dcaf11	1968.85801	1.0124506	0.01785152	0.0659836	0.77665467	0.87544229
Dcaf12	1358.67409	1.03858469	0.05461886	0.0704028	0.4116448	0.61088622
Dcaf12l1	652.655623	-1.1469558	-0.1978098	0.10643967	0.03355911	0.10262406
Dcaf13	1279.75104	-1.0392834	-0.0555891	0.0702698	0.39721137	0.59750998
Dcaf15	369.770745	1.10405154	0.14280752	0.12484139	0.16422595	0.33234351
Dcaf17	260.183016	1.1852453	0.24518568	0.13356162	0.02600911	0.08400875
Dcaf4	556.077537	-1.0622854	-0.0871715	0.08863295	0.27903845	0.47476192
Dcaf5	1961.98721	-1.0077712	-0.0111682	0.06341333	0.85331242	0.92125381
Dcaf6	954.0431	1.02365849	0.03373449	0.09574231	0.69562621	0.82612041
Dcaf7	1539.30361	1.09221241	0.12725346	0.06646433	0.04356304	0.12578613
Dcaf8	3417.75365	1.0098897	0.01419773	0.05746806	0.78502108	0.87956967
Dcakd	1582.45803	-1.0355493	-0.0503962	0.08729773	0.52909793	0.70983045
Dcbld1	372.572719	1.12886452	0.17487235	0.15117582	0.12906002	0.27970309
Dcbld2	5230.68465	-1.2083293	-0.2730137	0.05087327	3.37E-08	5.65E-07
Dcdc2a	92.6824238	-1.3546051	-0.4378724	0.21425654	0.00539559	0.0235284
Dcdc2b	2.11810236	1.00040392	0.00058261	0.21346171	0.98534405	0.99212536
Dclk1	476.681794	-1.1482911	-0.1994885	0.11036345	0.03700704	0.11068413
Dclk2	200.497001	1.34611668	0.42880346	0.17059171	0.00184824	0.0097794
Dclre1a	1608.5097	1.07563187	0.10518441	0.05958259	0.06571382	0.1715846
Dclre1b	813.905717	1.03777804	0.05349791	0.08306726	0.48149341	0.67165597
Dclre1c	540.986706	-1.0024106	-0.0034736	0.09118464	0.965707	0.98312856
Dcn	914.081238	-1.1772068	-0.2353677	0.10069199	0.00866845	0.03478609
Dcp1a	1011.5199	-1.0258399	-0.0368056	0.07003408	0.57774239	0.74410617
Dcp1b	365.324324	1.10836224	0.14842947	0.11698598	0.13348832	0.28657373
Dcp2	265.224138	-1.1402077	-0.1892967	0.12175174	0.06345519	0.16708096
Dcps	1770.5013	1.05092341	0.07165753	0.05941381	0.20934292	0.3946729
Dctn1	3043.06479	-1.8121846	-0.8577299	0.06756096	4.75E-38	7.00E-36
Dctn2	2341.16023	1.03569968	0.05060573	0.07220774	0.45548387	0.64989401
Dctn3	1096.77276	1.07460196	0.10380237	0.08466563	0.18178127	0.35773382
Dctn4	1780.94814	1.00815927	0.01172357	0.06565212	0.8519488	0.92042373
Dctn5	1609.85749	1.10341645	0.14197739	0.06176645	0.01608191	0.05759733
Dctn6	1417.17051	1.00116205	0.00167551	0.08114489	0.9830372	0.99131753
Dctpp1	1128.67726	-1.1031762	-0.1416633	0.08150911	0.0593244	0.1587188
Dcun1d4	1030.46097	1.03712073	0.05258384	0.07620754	0.46051214	0.65437167
Dcun1d5	420.025244	1.0047698	0.00686501	0.1156624	0.94531884	0.97295716
Dcxr	425.302523	1.44078489	0.52685495	0.12167693	2.00E-06	2.43E-05
Ddah2	6.68004876	-1.0279795	-0.0398115	0.21631603	0.3529001	0.55265464
Ddb1	11504.3762	1.00232991	0.00335745	0.05792193	0.95343112	0.97755794
Ddhd1	1838.76054	1.02539835	0.03618448	0.0600967	0.53070345	0.71101319
Ddhd2	4412.90929	1.1746372	0.23221523	0.0513066	3.11E-06	3.61E-05

Ddias	420.201958	-1.0178704	-0.0255539	0.10688993	0.78177885	0.87785651
Ddit3	316.961609	-1.1311017	-0.1777286	0.1217122	0.08147098	0.20062345
Ddit4	109.748384	-2.580565	-1.367687	0.2359245	3.50E-10	7.93E-09
Ddost	6131.37563	-1.015935	-0.0228081	0.05196935	0.6497517	0.79694746
Ddr1	1280.04029	1.14449835	0.19471538	0.09121381	0.0184616	0.06433436
Ddr2	5908.07722	1.14785509	0.19894052	0.04790442	1.99E-05	0.00019011
Ddrgk1	1784.47363	1.02009129	0.02869827	0.06416892	0.63983708	0.79058554
Ddt	759.77308	1.02844984	0.04047143	0.10331944	0.65440234	0.79983482
Ddx1	6702.40654	1.02495013	0.03555372	0.04754712	0.44426804	0.64095884
Ddx10	7145.41959	-1.1246239	-0.1694426	0.05975706	0.00307958	0.01490907
Ddx11	1350.61235	1.0647256	0.09048166	0.11346129	0.34942089	0.54907773
Ddx17	5146.74432	1.02349417	0.03350289	0.0542187	0.52206587	0.70426114
Ddx19b	3685.44035	-1.1191867	-0.1624507	0.05363819	0.00175118	0.00933526
Ddx20	2097.96378	-1.3028028	-0.3816188	0.07146898	2.23E-08	3.86E-07
Ddx23	7407.78361	-1.0675215	-0.0942651	0.04342376	0.02589908	0.08382115
Ddx24	3728.26969	-1.0473968	-0.0668081	0.05161814	0.18171287	0.35773382
Ddx25	6.31231745	-1.0168145	-0.0240565	0.21390454	0.52891629	0.70969076
Ddx27	4674.86504	1.23049197	0.29923525	0.05220215	3.71E-09	7.26E-08
Ddx28	267.928594	-1.245824	-0.3171002	0.16222982	0.01231529	0.04649539
Ddx31	1088.52496	1.01595084	0.02283059	0.07315939	0.74060928	0.85573192
Ddx39	2101.25677	1.02963115	0.04212761	0.09003228	0.60712787	0.7678038
Ddx39b	48.9501322	1.0166381	0.0238062	0.18734978	0.80075463	0.88972736
Ddx3x	36029.5869	-1.0865569	-0.1197638	0.05239051	0.01865936	0.0647663
Ddx4	32.5321012	-1.0787764	-0.1093958	0.21298713	0.28313343	0.47918341
Ddx41	2083.37082	1.04514715	0.06370608	0.06790141	0.3226689	0.52195841
Ddx42	5013.81814	1.03029274	0.04305431	0.05122035	0.37956958	0.58175238
Ddx46	7452.82552	1.00260345	0.0037511	0.0526135	0.94202741	0.97189769
Ddx47	2850.13085	-1.2200462	-0.2869358	0.05770572	2.49E-07	3.59E-06
Ddx49	1103.63196	-1.0496954	-0.0699708	0.0727493	0.30539666	0.50351818
Ddx5	21231.4168	-1.0728958	-0.10151	0.04227336	0.01410139	0.05194271
Ddx50	2598.32961	1.22570942	0.293617	0.06386241	1.61E-06	2.00E-05
Ddx51	342.99768	-1.1465267	-0.1972699	0.1419303	0.07859303	0.19534763
Ddx52	1805.29507	-1.0033693	-0.0048527	0.07618774	0.94498286	0.97295716
Ddx54	3985.36292	-1.0079321	-0.0113985	0.05366948	0.82869806	0.90788335
Ddx55	1219.06005	-1.0573484	-0.0804508	0.07623977	0.25806884	0.45026721
Ddx56	1484.94078	-1.0634408	-0.0887397	0.07927691	0.22683351	0.41553786
Ddx58	309.181111	1.01136897	0.01630942	0.11713196	0.86871975	0.92913918
Ddx59	687.963415	1.02580238	0.03675283	0.08433864	0.63518317	0.78732309
Ddx6	18195.3043	-1.0898614	-0.1241446	0.03841159	0.00099692	0.00581273
Deaf1	417.54527	-1.0488263	-0.0687758	0.10454324	0.45080612	0.64618934
Decr1	829.557393	-1.0996709	-0.1370718	0.08894699	0.08970021	0.2156918
Decr2	240.355658	1.13753055	0.18590529	0.13544065	0.08846028	0.21364559
Dedd	564.812313	-1.0709217	-0.0988531	0.08771029	0.216032	0.40217945
Dedd2	82.1412776	1.06213969	0.08697352	0.17433921	0.43626389	0.63383695
Def6	1279.85063	-1.0454189	-0.0640811	0.08775047	0.42233716	0.62078474
Def8	1876.3526	1.06416633	0.08972366	0.07638536	0.20809439	0.39293766
Degs1	9304.87912	1.00517479	0.00744639	0.04862337	0.8780964	0.93464187
Dele1	471.864409	1.03408493	0.04835469	0.08918365	0.55166072	0.7277643
Denn2b	1494.92693	1.07591695	0.10556672	0.05830888	0.0596364	0.15930419
Dennd11	310.558049	-1.0396929	-0.0561575	0.11331238	0.55895683	0.73259433

Dennd1a	618.454636	1.35939699	0.44296683	0.09100861	2.09E-07	3.04E-06
Dennd1b	294.015218	-1.1432614	-0.1931553	0.12697464	0.06589003	0.17195168
Dennd2a	1305.3572	-1.1132323	-0.1547546	0.06693645	0.01459351	0.0533665
Dennd2c	546.286627	1.03502321	0.04966312	0.09240751	0.55260767	0.72866839
Dennd3	273.318326	-1.1355501	-0.1833913	0.12277223	0.07399508	0.18684743
Dennd4a	3336.19749	-1.0277036	-0.0394242	0.05965253	0.4927341	0.67961115
Dennd4b	286.405962	1.07035014	0.09808282	0.13827004	0.36181763	0.56235316
Dennd4c	3066.18691	-1.0353755	-0.0501541	0.05634039	0.35592436	0.55596456
Dennd5a	7059.11554	1.08050913	0.11171127	0.04606284	0.01286065	0.04812881
Dennd5b	580.154134	1.19631306	0.25859498	0.08641477	0.00115235	0.00655488
Dennd6a	758.485161	1.06780653	0.09465027	0.07666364	0.18560498	0.36282615
Dennd6b	59.5637872	-1.0073258	-0.0105303	0.17278417	0.91721305	0.95692723
Denr	2133.55433	-1.0265996	-0.0378735	0.05916496	0.50403942	0.68849915
Depdc1a	2338.77538	-1.0470323	-0.0663059	0.05579507	0.21795934	0.40447039
Depdc1b	952.735915	-1.016244	-0.0232468	0.08641158	0.76802815	0.8713089
Depdc5	2103.10339	1.08731997	0.12077655	0.05538881	0.02367252	0.07841866
Depdc7	501.624256	1.25410854	0.32666222	0.10446401	0.00050108	0.00326323
Deptor	2322.64239	1.50727782	0.59194536	0.0641339	3.47E-21	2.23E-19
Derl1	2603.41271	-1.1832373	-0.2427395	0.05964076	2.21E-05	0.0002089
Derl2	157.384025	-1.0542392	-0.0762023	0.15390798	0.49110911	0.67828654
Des	3.07406454	-1.0205164	-0.0292994	0.21607098	0.34177724	0.54138327
Desi1	1906.29637	-1.118882	-0.1620579	0.06610408	0.00975385	0.03845702
Desi2	931.112375	-1.1132837	-0.1548213	0.094873	0.06812497	0.17594372
Det1	706.604026	1.29842776	0.37676575	0.0975948	2.63E-05	0.00024411
Deup1	21.5563334	-1.00492	-0.0070807	0.19739892	0.92630315	0.96259782
Dexi	275.826184	1.04674204	0.06590595	0.11090442	0.4893031	0.67718402
Dffa	1102.12147	-1.0945569	-0.130347	0.08118912	0.08187845	0.20143324
Dffb	148.36245	1.03640369	0.05158606	0.14058959	0.63123068	0.78498457
Dgat1	219.818449	-1.0278593	-0.0396428	0.15064977	0.71324378	0.83814814
Dgat2	257.304339	-1.0657645	-0.0918887	0.11330245	0.34058466	0.5400284
Dgat2l6	6.03138151	1.00622046	0.00894643	0.21107333	0.8408423	0.91469652
Dgcr6	1102.40941	1.39148067	0.47662087	0.0858469	4.67E-09	8.98E-08
Dgka	318.109906	1.09163704	0.12649325	0.12506272	0.21977632	0.40707557
Dgkd	1991.72271	1.07302652	0.10168574	0.07309475	0.13834477	0.29403006
Dgke	643.850854	-1.2140394	-0.2798152	0.10024526	0.00189953	0.00999709
Dgkh	511.325473	1.10833673	0.14839626	0.08959759	0.06805103	0.17589461
Dgkq	241.264708	1.14463112	0.19488274	0.14710454	0.08859589	0.21385898
Dgkz	1738.44541	1.05753738	0.08070866	0.0771733	0.2618871	0.45457639
Dglucy	3.86496491	-1.0375149	-0.053132	0.22320624	0.08608724	0.20925571
Dguok	163.013347	-1.9687376	-0.9772708	0.17664887	2.29E-09	4.62E-08
Dhcr24	2465.10965	-1.022739	-0.032438	0.07425443	0.64072631	0.79070924
Dhcr7	1568.87515	-1.1344404	-0.1819809	0.08135176	0.01517553	0.05505481
Dhdds	1126.03985	1.01867589	0.02669511	0.07017194	0.68808013	0.82110451
Dhodh	1264.22914	-1.1684648	-0.2246143	0.07690275	0.0017432	0.00929659
Dhps	1345.11291	-1.1298294	-0.176105	0.08237243	0.02021076	0.06922027
Dhrs1	1135.88174	1.03925412	0.05554847	0.07560336	0.43245948	0.63037983
Dhrs11	426.205466	1.06819739	0.09517827	0.11734461	0.33545251	0.53526988
Dhrs13	33.9939366	-1.0143863	-0.0206072	0.18868287	0.8215621	0.90376956
Dhrs3	98.1782189	2.45620143	1.29642888	0.29749307	6.62E-07	8.80E-06
Dhrs7	1002.86655	-1.0082855	-0.0119042	0.07906427	0.87068113	0.92983954

Dhrs7b	307.093708	-1.0211996	-0.0302648	0.12078152	0.76094679	0.86754436
Dhrs9	39.1536965	-1.1454407	-0.1959028	0.27463452	0.10011186	0.23316962
Dhtkd1	513.838555	1.08289841	0.11489791	0.1005675	0.19680996	0.37747201
Dhx15	7798.32227	-1.0665707	-0.0929797	0.05322876	0.07095283	0.18089973
Dhx16	3611.36099	-1.015456	-0.0221277	0.05243533	0.66279204	0.80485032
Dhx29	2618.44399	1.00498951	0.00718045	0.06600766	0.90967873	0.95292637
Dhx30	4866.15134	1.00758806	0.01090594	0.06406866	0.86028245	0.92521351
Dhx32	1590.5653	1.20788552	0.27248372	0.07324882	7.95E-05	0.00066566
Dhx33	1679.14593	-1.2604867	-0.3339809	0.06884066	3.77E-07	5.23E-06
Dhx34	457.231051	-1.0014481	-0.0020877	0.09725112	0.98012479	0.99079336
Dhx35	1334.6205	1.13544333	0.1832557	0.07730795	0.01092429	0.04219664
Dhx36	3562.46064	-1.0890355	-0.123051	0.05511334	0.02050719	0.0700194
Dhx37	1851.25827	1.01229538	0.01763032	0.06816373	0.78577537	0.88018569
Dhx38	2759.53793	-1.0055455	-0.0079784	0.05330725	0.87671972	0.93379726
Dhx40	1203.24257	1.00790605	0.01136117	0.08854534	0.88839301	0.94028872
Dhx57	1212.10605	1.29653897	0.37466557	0.08481211	2.45E-06	2.93E-05
Dhx58	15.1796135	1.03961573	0.05605037	0.21102394	0.44230809	0.6395718
Dhx8	28783.6952	-1.0068217	-0.0098082	0.04051944	0.74884068	0.86055881
Dhx9	16416.0869	-1.2494855	-0.3213341	0.05252408	2.84E-10	6.55E-09
Diablo	1088.24783	1.13527882	0.18304666	0.07431404	0.00853054	0.03430966
Diaph1	1858.09683	-1.04004	-0.0566391	0.05746547	0.30550782	0.50361023
Diaph2	941.154253	1.0966864	0.13315104	0.07310685	0.05218582	0.14363995
Diaph3	2989.39137	1.0910924	0.12577328	0.0567488	0.02132091	0.0722542
Dido1	3968.23211	1.09741152	0.13410463	0.05204188	0.00779287	0.03175149
Dimt1	923.732663	1.00310657	0.00447488	0.08880723	0.9568252	0.97901352
Dip2a	3540.84422	1.32879789	0.41012169	0.06071846	3.09E-12	9.37E-11
Dip2b	1910.65769	1.11190861	0.15303821	0.05655236	0.00491474	0.02180733
Dip2c	761.720659	-1.0112927	-0.0162006	0.09165523	0.84399543	0.91592523
Dipk1a	212.25204	1.03397142	0.04819631	0.13083015	0.64459544	0.79310405
Dis3	4532.12918	-1.1309114	-0.1774859	0.07497741	0.01130163	0.04341047
Dis3l	954.269307	-1.0294129	-0.0418218	0.07115885	0.53252179	0.71214157
Disp1	980.206586	1.07184812	0.1001005	0.07963953	0.17522541	0.34881701
Dixdc1	773.997976	-1.0826487	-0.1145652	0.0835983	0.1358063	0.29023669
Dkk3	2342.50812	1.10448333	0.14337164	0.05914385	0.01146276	0.04392401
Dlat	4647.63487	1.18972875	0.25063269	0.04866766	1.16E-07	1.77E-06
Dlc1	4373.45544	1.40413927	0.48968604	0.05199955	7.91E-22	5.33E-20
Dld	3896.60502	-1.0328046	-0.0465674	0.06753593	0.46729739	0.660123
Dlg1	4470.52667	1.18970197	0.25060021	0.05930768	1.09E-05	0.00011282
Dlg3	2032.70289	1.12350262	0.16800349	0.07794186	0.02051353	0.0700194
Dlg4	278.919726	1.17144066	0.22828388	0.12283694	0.02776001	0.08812557
Dlg5	5494.49845	1.05450493	0.07656584	0.04725389	0.09656301	0.22742639
Dlgap1	2.39563477	-1.0015693	-0.0022623	0.21325781	0.93910348	0.97054583
Dlgap4	1036.72812	-1.0253371	-0.0360983	0.08978838	0.65707168	0.80127899
Dlgap5	4389.30788	-1.0133187	-0.0190881	0.05122704	0.70096071	0.82981668
Dlk2	2.5459613	-1.0172028	-0.0246074	0.21611455	0.32287763	0.52216418
Dlst	3112.62767	-1.0736738	-0.1025557	0.05459569	0.05155324	0.14223497
Dlx1	107.32207	1.42969067	0.51570304	0.25752098	0.00448518	0.02028783
Dlx2	987.208937	1.5249642	0.60877537	0.14070102	1.60E-06	1.99E-05
Dlx4	69.8460393	1.4267573	0.51273994	0.29407179	0.00697717	0.02898103
Dmac1	493.538804	1.00210791	0.00303787	0.09706436	0.97280585	0.98768669

Dmac2	179.755781	-1.0812836	-0.112745	0.14149144	0.30057282	0.49836109
Dmac2l	103.766099	-1.0905386	-0.1250408	0.16880651	0.28033813	0.47608796
Dmap1	925.010734	1.12552491	0.17059799	0.0966347	0.048291	0.13561369
Dmpk	22.4531487	1.07049333	0.09827581	0.22918549	0.2149341	0.40108833
Dmrt1	7.29493009	1.01280142	0.01835133	0.21038646	0.7137264	0.83848451
Dmrta2	45.9257447	-1.0758278	-0.1054471	0.20028556	0.32697561	0.52673008
Dmtf1	2175.66078	1.11814696	0.16110982	0.07894639	0.02781842	0.08822825
Dmtn	106.079243	-1.1493635	-0.2008351	0.17708183	0.10663963	0.24437455
Dmwd	537.625166	1.4393456	0.52541304	0.12618013	4.06E-06	4.57E-05
Dmxl1	1606.91756	-1.0019895	-0.0028674	0.07697123	0.96741463	0.98424124
Dna2	1082.1713	1.08148973	0.11301997	0.08495407	0.14728543	0.30768531
Dnaaf1	88.7379308	1.35675881	0.44016427	0.33033508	0.01653012	0.05878702
Dnaaf2	1111.72933	-1.169088	-0.2253836	0.08118007	0.00272134	0.0135275
Dnaaf5	879.079124	-1.0065822	-0.009465	0.0844251	0.902183	0.94933088
Dnah10	3.79288897	-1.0281076	-0.0399912	0.21848412	0.22914437	0.41766719
Dnah3	3.24784446	-1.0326976	-0.0464178	0.22160494	0.07911545	0.19637064
Dnah5	3.63497329	-1.0246236	-0.035094	0.21562369	0.39120227	0.5925625
Dnah6	2.51134054	1.03230515	0.0458695	0.22148548	0.08113881	0.20003621
Dnah8	1224.27756	1.6850994	0.75283369	0.07758206	2.81E-23	2.03E-21
Dnaic2	1.66562412	-1.0179546	-0.0256733	0.21607362	0.34221248	0.54180471
Dnaja1	3187.00769	-1.1493818	-0.2008581	0.07320296	0.00343317	0.01632475
Dnaja2	4329.93792	-1.2030892	-0.2667437	0.06012153	3.84E-06	4.36E-05
Dnaja3	3501.66553	1.00020062	0.00028941	0.05163653	0.99595855	0.99827695
Dnajb1	754.198552	1.06413954	0.08968735	0.09161681	0.27962021	0.47537291
Dnajb11	3046.20241	1.11211682	0.15330834	0.08665871	0.05313194	0.1456495
Dnajb12	2462.24186	-1.0675705	-0.0943314	0.06123048	0.10709938	0.24502127
Dnajb13	42.1138441	-1.0394699	-0.055848	0.19419318	0.55672248	0.7312227
Dnajb14	180.251717	1.46385928	0.54977688	0.1913279	0.00044001	0.00291444
Dnajb2	1459.67247	1.47566861	0.56136877	0.0797855	2.70E-13	9.14E-12
Dnajb3	37.0241502	-1.0130779	-0.0187451	0.18665001	0.84086387	0.91469652
Dnajb4	628.868979	-1.3636282	-0.4474503	0.09893911	1.08E-06	1.38E-05
Dnajb5	231.988019	1.04266664	0.06027797	0.11226533	0.53273506	0.71226071
Dnajc1	299.675	-1.1214903	-0.1654172	0.13971148	0.13416535	0.28773784
Dnajc10	9954.61985	1.04727767	0.06664401	0.04798045	0.15454133	0.31807108
Dnajc11	3438.17764	-1.0389873	-0.055178	0.06661716	0.38167966	0.5836596
Dnajc12	10.9943826	1.14271425	0.19246469	0.32860869	0.03451415	0.10489355
Dnajc13	7225.53586	1.03786174	0.05361427	0.05970268	0.3471516	0.54671896
Dnajc15	649.859353	-1.1396503	-0.1885913	0.11325191	0.05265763	0.14453487
Dnajc16	1162.75415	-1.0343269	-0.0486922	0.06998761	0.46115563	0.65499832
Dnajc17	299.951853	1.02461733	0.0350852	0.11734962	0.72295727	0.84385629
Dnajc18	1070.26692	1.08054928	0.11176488	0.06824981	0.08358309	0.20444898
Dnajc2	8533.99056	1.10792159	0.14785579	0.0545531	0.00497521	0.02204514
Dnajc21	2108.60135	-1.033123	-0.047012	0.06154191	0.42480768	0.6231291
Dnajc22	426.119754	-1.2587188	-0.3319561	0.13974211	0.0043323	0.01967971
Dnajc24	769.257916	1.20073918	0.26392281	0.11892016	0.00992761	0.039022
Dnajc25	638.445524	-1.0418209	-0.0591073	0.09950252	0.5020733	0.68713236
Dnajc27	547.01927	-1.0011893	-0.0017148	0.08809417	0.9825175	0.99114898
Dnajc28	130.117458	1.05566085	0.07814641	0.15221522	0.48050429	0.6709755
Dnajc3	2339.3777	-1.0120096	-0.017223	0.07316515	0.80197868	0.89054671
Dnajc30	20.4591384	-1.0399333	-0.056491	0.2029499	0.5070009	0.69136486

Dnajc4	132.407439	1.23358629	0.30285864	0.18939776	0.02656226	0.08534522
Dnajc5	949.017961	1.41351372	0.49928589	0.09941383	7.57E-08	1.19E-06
Dnajc5b	3.45765693	1.0069762	0.01002958	0.21223108	0.79339396	0.88515734
Dnajc6	74.4893811	-1.0358457	-0.0508091	0.16189025	0.64004634	0.79058554
Dnajc7	5724.35181	-1.1249158	-0.1698171	0.05570549	0.00154559	0.00835753
Dnajc8	4051.40599	-1.0456708	-0.0644288	0.05636555	0.23555507	0.42535389
Dnajc9	1281.56691	1.03607219	0.05112454	0.06924973	0.43564593	0.63329827
Dnal1	2111.82324	1.2630632	0.33692683	0.07625046	2.92E-06	3.41E-05
Dnal4	261.827516	1.59779045	0.67607821	0.16403134	3.56E-06	4.06E-05
Dnase1l2	26.7439561	-1.0141696	-0.020299	0.19185511	0.81717399	0.90128368
Dnase1l3	168.885807	-1.0976144	-0.1343713	0.16722968	0.2496395	0.44102634
Dnase2a	1922.1515	1.53359057	0.61691337	0.06583111	8.47E-22	5.68E-20
Dnd1	7.98099519	1.04041334	0.05715681	0.21678186	0.34701171	0.54664894
Dnhd1	40.1588295	-1.1986218	-0.2613766	0.33964383	0.05324256	0.14585911
Dnlz	112.776681	-1.0237298	-0.033835	0.15351753	0.7532382	0.86298066
Dnm1	28.7513431	-1.004369	-0.0062895	0.19116217	0.94117006	0.97139497
Dnm1l	3242.52636	1.12659723	0.17197182	0.05365955	0.00091226	0.00540842
Dnm2	2658.6903	1.0677307	0.09454782	0.06959385	0.15037987	0.31196213
Dnmbp	1531.29586	1.13226357	0.17920983	0.06713696	0.00481657	0.02146096
Dnmt1	7031.54075	-1.2514649	-0.3236179	0.05205167	1.14E-10	2.83E-09
Dnmt3a	1346.03767	1.67540441	0.74450938	0.07038613	3.64E-27	3.24E-25
Dnpep	989.068063	1.10620688	0.14562122	0.12652273	0.16174565	0.3285699
Dnttip1	1576.75759	1.32061782	0.40121302	0.08754985	1.01E-06	1.30E-05
Dnttip2	11127.6575	-1.1599881	-0.2141099	0.0770411	0.00286248	0.01404381
Doc2g	2.15892844	1.00628003	0.00903184	0.21369156	0.75360616	0.86298066
Dock1	8175.05039	1.09418971	0.1298629	0.05670002	0.01737128	0.06141314
Dock10	35.9077909	1.00776697	0.01116208	0.18615986	0.90775689	0.95220813
Dock11	2962.9221	-1.1768683	-0.2349529	0.06368446	0.00010806	0.00087167
Dock3	847.013761	1.33627532	0.41821728	0.10590388	1.55E-05	0.00015399
Dock4	856.259905	-1.0302533	-0.0429991	0.08619337	0.58511721	0.74938527
Dock5	6568.08323	1.20121589	0.26449547	0.05764404	1.91E-06	2.33E-05
Dock6	109.100233	1.0327023	0.04642442	0.1786273	0.65587752	0.8004869
Dock7	8892.5969	1.07949277	0.11035358	0.05015926	0.02345625	0.07784316
Dock8	2868.45803	-1.003175	-0.0045733	0.0570989	0.93284133	0.96688223
Dock9	2653.20669	1.27342222	0.34871085	0.0631679	9.50E-09	1.74E-07
Dok1	323.151191	-1.8596515	-0.8950323	0.14458856	4.44E-11	1.17E-09
Dok2	2.0067037	-1.0086235	-0.0123877	0.21438208	0.63510218	0.78732309
Dok3	8.09280775	-1.010796	-0.0154918	0.20787084	0.78400759	0.87922895
Dok4	150.103165	1.13776698	0.18620511	0.15177785	0.10834284	0.24704019
Dok5	621.917116	1.59633175	0.67476051	0.10393589	8.26E-12	2.37E-10
Dolk	299.254261	1.03223325	0.04576902	0.11391367	0.63564451	0.78779085
Dolpp1	638.097531	-1.1335286	-0.1808208	0.10303471	0.04614519	0.13103964
Donson	940.065473	1.13661264	0.18474067	0.07939063	0.01207638	0.0457417
Dop1a	2154.06635	1.15391355	0.20653514	0.05290411	5.40E-05	0.00046875
Dop1b	927.867346	1.22957332	0.29815776	0.09978967	0.00093213	0.00550511
Dot1l	2479.26949	-1.195806	-0.2579834	0.07666149	0.00032332	0.00222197
Doxl2	444.973038	-1.9077057	-0.9318387	0.11549007	5.41E-17	2.59E-15
Dpf1	148.396981	1.07570252	0.10527916	0.14797521	0.34358227	0.54303379
Dpf2	2334.72193	1.11545948	0.15763811	0.06940929	0.01608097	0.05759733
Dpf3	115.324327	1.3112101	0.39089887	0.25142786	0.01643991	0.05849869

Dph1	966.320148	-1.0939586	-0.1295581	0.08646207	0.10034479	0.23362719
Dph2	342.920152	-1.0755948	-0.1051347	0.1311052	0.31728313	0.51625029
Dph3	399.108867	1.12530241	0.17031276	0.13035725	0.11011869	0.2502875
Dph5	938.972791	1.02658819	0.03785757	0.09465934	0.65649733	0.80089399
Dph6	806.01247	1.11071221	0.15148506	0.09091747	0.06566568	0.17149387
Dph7	334.215203	-1.2020472	-0.2654935	0.1293684	0.01417308	0.05215659
Dpm3	12.5760747	-1.0187331	-0.0267761	0.20928495	0.64008497	0.79058554
Dpp3	2897.92919	1.09797042	0.13483918	0.05715339	0.01414925	0.05208387
Dpp7	2417.64317	1.23604556	0.30573193	0.0594886	9.55E-08	1.49E-06
Dpp8	5153.79152	1.08640616	0.11956357	0.04493517	0.00664293	0.02777284
Dpp9	3300.77007	-1.0220862	-0.0315169	0.06952929	0.63123545	0.78498457
Dpy19l1	2075.83346	1.00588022	0.00845852	0.06057068	0.88594729	0.93872913
Dpy19l3	856.637367	-1.041471	-0.0586227	0.08493442	0.45165248	0.64687704
Dpy19l4	1458.38189	1.08438443	0.1168763	0.07087053	0.07996915	0.19783792
Dpy30	501.648579	1.00642307	0.0092369	0.10046168	0.91783396	0.9572914
Dpysl2	5764.58642	1.0545585	0.07663913	0.04455842	0.0782809	0.19497926
Dpysl3	52.5466654	1.70912842	0.7732608	0.40992913	0.00287667	0.01409723
Dqx1	64.7425188	-2.8420137	-1.5069135	0.28747449	8.58E-09	1.59E-07
Dram1	89.6610637	1.03420888	0.0485276	0.16605448	0.65467865	0.79986554
Dram2	541.475309	1.21732478	0.28371413	0.09063864	0.0006372	0.00398756
Drap1	3865.24166	1.25212782	0.32438185	0.05659948	3.15E-09	6.24E-08
Drc1	2.5649204	-1.0011087	-0.0015987	0.21345047	0.95351764	0.97755794
Drc3	9.54809177	-1.0128368	-0.0184017	0.20890949	0.73325139	0.85081284
Drc7	9.84135055	-1.1916972	-0.2530177	0.4918381	0.00635662	0.0268386
Drg2	1038.13027	-1.1852483	-0.2451893	0.08144528	0.00115958	0.00658141
Drosha	8468.64282	-1.0261299	-0.0372134	0.04611376	0.40359138	0.60336535
Dscc1	3921.80994	-1.0690091	-0.0962742	0.06294537	0.10880315	0.24795738
Dse	2509.4647	-1.1136856	-0.155342	0.05450155	0.00311603	0.01505707
Dsel	477.938687	-1.1894658	-0.2503138	0.10934217	0.0089012	0.03554376
Dsn1	1357.39574	1.05802372	0.08137197	0.07565662	0.24987457	0.44125906
Dsp	3884.57779	1.12394499	0.16857142	0.0546738	0.0013947	0.00767115
Dst	26529.3253	1.13227156	0.17922001	0.05586619	0.00090996	0.00540157
Dstn	5045.64416	-1.248341	-0.3200121	0.05917001	2.04E-08	3.57E-07
Dstyk	694.855236	1.17656925	0.23458624	0.08324535	0.00229838	0.01173467
Dtd1	259.685482	1.05239227	0.07367255	0.12205175	0.46626774	0.65925935
Dtd2	820.053006	1.00825784	0.01186463	0.08031274	0.87432864	0.93221108
Dtl	1931.65817	-1.0766034	-0.1064869	0.06259329	0.07419991	0.18725432
Dtnb	649.784423	1.30365598	0.3825632	0.09755011	1.99E-05	0.00019011
Dtnbp1	722.449891	-1.0200669	-0.0286638	0.09561719	0.73674875	0.85293732
Dtwd1	1236.47704	1.08075955	0.11204559	0.06761629	0.08028091	0.19849421
Dtx2	269.187326	-1.155462	-0.2084698	0.14702461	0.06934241	0.17829713
Dtx3	276.152752	1.02065875	0.02950059	0.14106298	0.78646831	0.88053373
Dtx4	14.6551961	3.85131194	1.94534998	0.71212959	0.0002024	0.00151003
Dtymk	1438.01681	1.01539254	0.02203756	0.07558253	0.75624793	0.86464977
Duox1	11.2855001	-1.0771429	-0.1072097	0.24493616	0.09042524	0.2169596
Dus1l	903.566433	-1.0145724	-0.0208718	0.07636045	0.76900822	0.87153811
Dus2	626.229115	1.01827171	0.02612257	0.09685833	0.76368416	0.86923666
Dus3l	822.694359	-1.0363307	-0.0514845	0.08809824	0.52141	0.70372828
Dus4l	403.429627	1.15345244	0.20595852	0.11729855	0.03959375	0.11667724
Dusp1	1280.81453	1.20650457	0.27083338	0.1018936	0.00294957	0.01438843

Dusp10	1689.72772	-1.4580531	-0.5440432	0.08903084	1.35E-10	3.30E-09
Dusp11	621.282349	-2.1014706	-1.0713993	0.09617372	4.80E-30	5.21E-28
Dusp12	847.33406	-1.0015219	-0.0021939	0.0903915	0.97826854	0.9900158
Dusp13	4.21059856	-1.0195898	-0.0279889	0.21465164	0.46089492	0.65484293
Dusp14	235.29995	1.23676679	0.30657348	0.17167215	0.01861808	0.06473111
Dusp15	862.658538	-1.0792608	-0.1100435	0.08187157	0.14459716	0.30379387
Dusp16	3103.20679	-1.0483429	-0.0681107	0.0557723	0.20537067	0.38948289
Dusp18	113.194444	1.02626818	0.03740778	0.1473384	0.72961501	0.84829649
Dusp19	1288.17707	1.02373953	0.03384869	0.08337364	0.66058177	0.80353741
Dusp28	2.1940045	-1.0037627	-0.0054182	0.21356019	0.84846255	0.91851646
Dusp4	2110.77603	-1.1372366	-0.1855324	0.07172102	0.00594675	0.02539829
Dusp5	1121.225	-2.1770388	-1.1223671	0.08508673	5.92E-41	9.72E-39
Dusp6	474.785224	-1.745625	-0.8037437	0.13533865	2.26E-10	5.28E-09
Dusp7	375.018477	1.04867354	0.06856562	0.10185662	0.44538357	0.64184538
Dusp8	239.635844	-1.0106237	-0.0152458	0.13391781	0.8832432	0.93731633
Dvl1	1617.96907	-1.0652647	-0.091212	0.08944385	0.26066148	0.45300143
Dvl2	626.5099	1.03035371	0.04313969	0.07960374	0.55986258	0.73310886
Dvl3	1384.66098	1.02747599	0.03910468	0.05866701	0.4895671	0.67718402
Dxo	126.737076	-1.0454919	-0.0641818	0.14923484	0.55571993	0.73050512
Dym	1116.5987	1.12384398	0.16844177	0.07650958	0.01813454	0.06346841
Dync1h1	23584.288	-1.0170243	-0.0243542	0.04222397	0.55567967	0.73050512
Dync1i2	5032.39836	1.07600057	0.10567884	0.05886412	0.06159771	0.16336783
Dync1li1	2907.45331	1.0043628	0.0062805	0.05382471	0.90463536	0.95022649
Dync1li2	4018.03151	1.05856787	0.08211377	0.04720551	0.07461227	0.18796129
Dync2h1	1517.43841	1.03966899	0.05612428	0.07274025	0.41202574	0.61116834
Dync2li1	1225.49248	-1.2592611	-0.3325774	0.08496257	2.59E-05	0.00024118
Dynll2	510.867982	-1.0911113	-0.1257983	0.12806257	0.22758585	0.41624424
Dynlrb1	372.981852	1.02344064	0.03342743	0.11696609	0.73455551	0.85147553
Dynlt3	1215.47218	1.18598886	0.24609046	0.08348305	0.00144112	0.00786904
Dyrk1a	5018.79059	1.02495986	0.03556741	0.04736857	0.44172524	0.63907133
Dyrk1b	35.74128	1.16976218	0.22621525	0.31163275	0.06890211	0.17744994
Dyrk2	1437.91851	-1.338273	-0.4203724	0.07404348	2.80E-09	5.58E-08
Dyrk3	530.797857	1.15341076	0.20590639	0.12603801	0.04972459	0.13850229
Dysf	1.68932694	-1.0281303	-0.0400231	0.22051368	0.03371639	0.10305581
Dzank1	75.0253105	1.00799834	0.01149327	0.16692046	0.91469008	0.9555461
Dzip1	667.810344	1.63954694	0.71329721	0.10863313	5.27E-12	1.56E-10
Dzip1l	700.113787	-1.2048709	-0.2688786	0.09274259	0.00142675	0.00781053
Dzip3	2233.06396	1.04142868	0.05856404	0.06101891	0.31667895	0.51558939
E130308A19Rik	176.895709	-1.0378084	-0.0535401	0.13023556	0.60664834	0.7674467
E130309D02Rik	645.531164	-1.1145932	-0.1565173	0.10039891	0.07752495	0.1935857
E130311K13Rik	133.672565	1.19670567	0.25906836	0.20670828	0.05739838	0.1546316
E2f1	1474.21191	1.10565132	0.14489649	0.09279951	0.08321659	0.20374704
E2f2	239.180277	-1.2266994	-0.2947817	0.15737063	0.01675402	0.05945125
E2f3	1742.85141	-1.1727458	-0.2298903	0.06696087	0.00029866	0.00207699
E2f4	1772.36303	1.01428708	0.02046605	0.07814385	0.77924634	0.87673482
E2f5	75.4514367	-1.0088996	-0.0127826	0.15693993	0.9026426	0.94945197
E2f6	1386.60364	-1.0318894	-0.0452883	0.06423219	0.45916876	0.65325987
E2f7	2658.75655	-1.1627261	-0.2175113	0.06283232	0.00028509	0.0020044
E2f8	2818.26231	1.05327013	0.0748755	0.05770004	0.1771454	0.35130443
E330021D16Rik	149.670726	1.05349199	0.07517935	0.13435954	0.48116919	0.6713623

E4f1	452.31887	-1.0002669	-0.000385	0.09913514	0.99486771	0.99782769
EEF1A1	174037.32	-2.1619059	-1.1123038	0.0455312	5.68E-133	1.21E-129
EEF1E1	2492.20166	1.01522517	0.02179974	0.07389255	0.75407381	0.86317158
EIF3B	12191.3519	-1.0799623	-0.110981	0.04031987	0.0050068	0.02216977
EIF3C	19123.7212	-1.0814932	-0.1130246	0.04800179	0.01538301	0.0556028
EIF3D	7971.11941	-1.157259	-0.2107118	0.06012515	0.00025155	0.00180828
EIF3G	6741.50413	-1.2430552	-0.3138904	0.04093846	5.65E-15	2.27E-13
EIF3I	5768.04563	-1.0590176	-0.0827266	0.07785382	0.25260356	0.44390425
EIF3M	4694.68675	-1.0452175	-0.0638032	0.05907453	0.27301599	0.46881165
EIF6	3624.68941	-1.6897282	-0.7567912	0.0652779	3.99E-32	4.61E-30
ELOVL4	743.924818	1.07958003	0.1104702	0.1012889	0.21667207	0.4029572
ELOVL5	7871.40149	-1.0803733	-0.1115299	0.05097585	0.02484644	0.08131908
ELOVL6	219.858518	-1.3000876	-0.3786088	0.17516075	0.00555064	0.02407847
ELOVL7	449.970427	1.30430632	0.38328273	0.12401122	0.00041845	0.00278024
ENOPH1	869.482499	1.0018155	0.00261684	0.08861747	0.97487768	0.98861487
ENSCGRG00001000002	47245.3231	1.07095268	0.09889474	0.20675557	0.39997551	0.6001038
ENSCGRG00001000003	15.1540441	1.01955114	0.02793415	0.21186114	0.57418148	0.74210864
ENSCGRG00001000004	193048.187	1.01148092	0.01646911	0.19888288	0.84011469	0.91430211
ENSCGRG00001000009	1.93022117	-1.0012718	-0.0018337	0.21376333	0.9428575	0.97203793
ENSCGRG00001000039	5531.67205	-1.0454946	-0.0641856	0.06513511	0.28933767	0.48574196
ENSCGRG00001000040	1069.93783	1.01920816	0.02744873	0.07934798	0.71067879	0.83674589
ENSCGRG00001000042	353.584716	-1.404503	-0.4900597	0.19319871	0.00133148	0.00735819
ENSCGRG00001000043	1.94444904	-1.0005308	-0.0007655	0.21276812	0.98032631	0.99079336
ENSCGRG00001000045	25.0712661	-1.0169523	-0.024252	0.19370285	0.77812955	0.87652476
ENSCGRG00001000058	38.2601509	1.47058682	0.55639196	0.47945323	0.01187387	0.04525567
ENSCGRG00001000060	9.92009761	-1.0537166	-0.0754869	0.22573824	0.20473855	0.38854826
ENSCGRG00001000064	18.8934601	-1.0042269	-0.0060852	0.21359992	0.82675072	0.90704666
ENSCGRG00001000067	324.812788	-1.0715745	-0.0997322	0.14994525	0.36852036	0.5700404
ENSCGRG00001000070	85.9824843	-1.0783102	-0.1087722	0.18344357	0.33641955	0.5363453
ENSCGRG00001000079	1386.50717	-1.5262128	-0.6099561	0.10934351	2.83E-09	5.63E-08
ENSCGRG00001000081	125.207609	1.40894201	0.49461224	0.24782626	0.00487155	0.02165145
ENSCGRG00001000088	49.3963006	-1.0884976	-0.1223382	0.21571014	0.25324538	0.44475916
ENSCGRG00001000090	474.897356	1.07593795	0.10559488	0.1086877	0.26207825	0.45484655
ENSCGRG00001000093	416.458805	-1.0827775	-0.1147368	0.0932196	0.17108513	0.34275892
ENSCGRG00001000099	5336.25675	-1.1191089	-0.1623505	0.09493893	0.05647773	0.15256661
ENSCGRG00001000106	486.209052	1.33588389	0.41779462	0.1086383	2.32E-05	0.00021807
ENSCGRG00001000121	631.58792	-1.0249962	-0.0356185	0.09897663	0.68425933	0.81858069
ENSCGRG00001000130	779.060825	-1.0692046	-0.0965379	0.09148997	0.24249867	0.43246665
ENSCGRG00001000139	1400.64431	-1.1130282	-0.1544902	0.10260881	0.08810418	0.21310698
ENSCGRG00001000151	268.581437	1.0067015	0.00963597	0.13138189	0.92841076	0.96400599
ENSCGRG00001000174	3.01319244	-1.0012842	-0.0018516	0.21159457	0.96084348	0.98137498
ENSCGRG00001000181	67.3046472	-1.6124529	-0.689257	0.47572707	0.00606189	0.02578971
ENSCGRG00001000186	1253.04304	1.06636976	0.09270778	0.079237	0.20743501	0.3922708
ENSCGRG00001000195	74.184348	-1.0051395	-0.0073958	0.16904632	0.94223243	0.97189769
ENSCGRG00001000199	304.670287	-1.0274776	-0.0391069	0.12406545	0.69909228	0.82849333
ENSCGRG00001000206	510.356722	-1.0162171	-0.0232087	0.09775462	0.78834054	0.88132248
ENSCGRG00001000208	371.120389	-1.009057	-0.0130076	0.10394507	0.88555476	0.93868072
ENSCGRG00001000209	32.3992533	1.12348846	0.16798531	0.28356174	0.07702663	0.19267938
ENSCGRG00001000225	3677.77424	1.00013337	0.0001924	0.05140703	0.99744217	0.99915813
ENSCGRG00001000229	1014.72974	-1.0555191	-0.0779527	0.11122097	0.41246555	0.6114962

ENSCGRG00001000247	51.0425628	-1.4692566	-0.5550863	0.31563945	0.00595436	0.02541663
ENSCGRG00001000257	1155.40243	-1.0892046	-0.123275	0.08688128	0.11950759	0.26495193
ENSCGRG00001000259	131.291905	1.01605957	0.02298499	0.141744	0.83092713	0.9092919
ENSCGRG00001000272	69.6302681	1.08717826	0.12058851	0.17571936	0.29854553	0.49638308
ENSCGRG00001000281	481.443888	-1.0533718	-0.0750147	0.10335389	0.40579525	0.60529078
ENSCGRG00001000284	7777.17331	-1.2041174	-0.2679761	0.07764714	0.0002201	0.00162413
ENSCGRG00001000303	6847.55236	-1.0961452	-0.1324389	0.13141476	0.21585546	0.4021391
ENSCGRG00001000310	30.295271	-1.0183026	-0.0261663	0.19637555	0.75255477	0.8628589
ENSCGRG00001000311	3.07632648	1.0106917	0.01534299	0.2125237	0.69758664	0.82741461
ENSCGRG00001000315	558.696141	-1.0439711	-0.0620818	0.10164156	0.48695128	0.67549778
ENSCGRG00001000328	1910.96369	-1.4046279	-0.490188	0.08114477	2.45E-10	5.71E-09
ENSCGRG00001000350	1303.85748	-1.0245726	-0.0350222	0.0774362	0.62701038	0.78218883
ENSCGRG00001000351	3280.24825	1.0538848	0.07571717	0.06664045	0.23195456	0.42122738
ENSCGRG00001000354	6.38203391	1.01147166	0.0164559	0.20971024	0.74905223	0.86072471
ENSCGRG00001000377	625.642573	-1.0445427	-0.0628715	0.09402224	0.45771454	0.65198747
ENSCGRG00001000391	508.832636	-1.4855754	-0.5710218	0.17159753	9.04E-05	0.00074249
ENSCGRG00001000392	233.119878	-1.5948347	-0.6734069	0.18400654	2.23E-05	0.00021054
ENSCGRG00001000425	508.644954	1.2869355	0.36393974	0.16509507	0.00560147	0.02424967
ENSCGRG00001000436	270.27954	1.07912181	0.10985773	0.12359058	0.28331036	0.47941951
ENSCGRG00001000441	64.6780847	-1.0763186	-0.1061052	0.19105753	0.33921866	0.53880743
ENSCGRG00001000466	10484.1645	-1.4021612	-0.4876522	0.07241101	2.62E-12	8.01E-11
ENSCGRG00001000469	109.45068	-1.0284569	-0.0404814	0.17159657	0.70204017	0.83031433
ENSCGRG00001000473	177.795041	1.26609687	0.34038779	0.23465584	0.0254476	0.08271599
ENSCGRG00001000474	957.487664	1.00809545	0.01163224	0.08895621	0.88652799	0.93909067
ENSCGRG00001000484	1116.69393	1.07152553	0.09966623	0.09650101	0.25067968	0.44201055
ENSCGRG00001000492	253.240782	-1.5469144	-0.6293934	0.20549015	0.00018726	0.00141776
ENSCGRG00001000517	32.7759083	2.38896842	1.25638778	0.45590767	0.00024419	0.0017653
ENSCGRG00001000540	378.889034	-1.0347319	-0.0492571	0.11782224	0.61686908	0.77463904
ENSCGRG00001000541	604.009757	-1.1483548	-0.1995685	0.11564519	0.04367389	0.12596329
ENSCGRG00001000551	76.3209121	-1.5285053	-0.6121215	0.39433111	0.00666981	0.027867
ENSCGRG00001000559	491.802561	-1.099114	-0.136341	0.13331331	0.19976407	0.38142462
ENSCGRG00001000565	903.298182	1.46402007	0.54993533	0.08348137	6.03E-12	1.76E-10
ENSCGRG00001000566	7961.70834	-1.1752107	-0.2329194	0.04897005	9.90E-07	1.28E-05
ENSCGRG00001000568	6.59169033	-1.0350991	-0.0497689	0.2144668	0.40412254	0.60358923
ENSCGRG00001000572	7.63407862	-1.0511756	-0.0720036	0.22543089	0.19262199	0.37164661
ENSCGRG00001000578	531.857777	1.02571764	0.03663363	0.09501485	0.66806371	0.80811765
ENSCGRG00001000579	40.6186537	-1.0613632	-0.0859185	0.22236331	0.26517207	0.45891032
ENSCGRG00001000587	275.675413	1.07988278	0.11087471	0.14270801	0.31305474	0.51207488
ENSCGRG00001000591	159.951235	1.0988583	0.13600536	0.14584576	0.22363206	0.41189456
ENSCGRG00001000596	120.196352	-1.0247163	-0.0352245	0.1774029	0.73558435	0.85212837
ENSCGRG00001000597	8.25100288	-1.0924552	-0.1275741	0.26687098	0.02457337	0.08076528
ENSCGRG00001000598	31.7854369	-1.0041741	-0.0060094	0.1894724	0.94538624	0.97295716
ENSCGRG00001000599	133.71747	-1.1933443	-0.2550104	0.24697751	0.06837118	0.1764373
ENSCGRG00001000601	136.124406	-1.0305381	-0.0433978	0.16062386	0.68550914	0.81956079
ENSCGRG00001000605	115.415097	1.13059849	0.17708667	0.20266642	0.15426339	0.31765215
ENSCGRG00001000609	6041.58284	-1.1354293	-0.1832379	0.04586182	4.12E-05	0.00036832
ENSCGRG00001000615	17.1549148	1.06837435	0.09541724	0.22188489	0.270665	0.46596139
ENSCGRG00001000633	1.81578121	1.01216298	0.01744162	0.21483688	0.51486247	0.69851601
ENSCGRG00001000645	383.779978	1.07626688	0.10603586	0.11286045	0.27168817	0.4672205
ENSCGRG00001000646	1778.68625	-1.4513739	-0.5374192	0.06937519	1.34E-15	5.69E-14

ENSCGRG00001000648	1821.35306	1.2128587	0.27841149	0.11489181	0.00540164	0.02354371
ENSCGRG00001000658	10.9515102	-1.0387009	-0.0547803	0.2155717	0.37037064	0.571758
ENSCGRG00001000659	127.852749	-1.0302481	-0.0429918	0.16450025	0.6890851	0.82183486
ENSCGRG00001000667	993.913465	1.15055696	0.20233241	0.10801717	0.03176859	0.09817989
ENSCGRG00001000687	2666.99182	-1.0332675	-0.0472139	0.07014875	0.47406022	0.6647741
ENSCGRG00001000694	9.70744146	-1.1193251	-0.1626291	0.30069364	0.02478779	0.08122193
ENSCGRG00001000697	94.2324028	-1.0893753	-0.1235011	0.1715628	0.28549342	0.48190417
ENSCGRG00001000701	229.244757	-1.0759899	-0.1056646	0.17632023	0.35228353	0.55195108
ENSCGRG00001000706	7.37950382	-1.0188698	-0.0269697	0.21028779	0.61833981	0.77566023
ENSCGRG00001000714	54.6850823	-1.08281	-0.1147801	0.19662721	0.30282182	0.50059975
ENSCGRG00001000726	4478.42719	-1.218861	-0.2855336	0.06312369	2.30E-06	2.76E-05
ENSCGRG00001000733	1165.89634	1.03908062	0.05530759	0.14913247	0.61339026	0.77269695
ENSCGRG00001000738	36.2962133	-1.1874195	-0.2478297	0.36162277	0.04657852	0.13197763
ENSCGRG00001000765	482.847796	-1.0823806	-0.1142079	0.0935702	0.17291342	0.34551948
ENSCGRG00001000775	1148.35414	1.09178071	0.12668311	0.16333356	0.27316094	0.46893482
ENSCGRG00001000784	744.101775	-1.7728703	-0.826087	0.10058485	1.81E-17	8.99E-16
ENSCGRG00001000796	1953.96515	-1.1274229	-0.1730287	0.06888572	0.00769255	0.03141182
ENSCGRG00001000800	35.8995707	-1.0914332	-0.1262239	0.21218849	0.25104744	0.44235866
ENSCGRG00001000807	1219.25842	-1.0314613	-0.0446897	0.09755895	0.60676987	0.76752464
ENSCGRG00001000809	108.61402	-1.1309323	-0.1775126	0.19342067	0.15187629	0.31402144
ENSCGRG00001000811	4874.86053	1.11504208	0.15709815	0.09004018	0.05449737	0.14839339
ENSCGRG00001000813	4106.16334	1.03956422	0.05597888	0.06753177	0.38324791	0.58445306
ENSCGRG00001000829	39.6122596	-1.1378228	-0.1862759	0.25595461	0.11833787	0.26304149
ENSCGRG00001000834	74.1104351	-1.011218	-0.016094	0.1691753	0.87685042	0.93385882
ENSCGRG00001000842	255.440603	-1.0913212	-0.1260758	0.13230155	0.23443892	0.42429536
ENSCGRG00001000843	42.0741623	1.00656597	0.00944172	0.19778706	0.90784243	0.95221989
ENSCGRG00001000851	14.3617305	-1.0483113	-0.0680671	0.21689996	0.33488341	0.53462817
ENSCGRG00001000854	5.49562692	1.01288664	0.01847272	0.21315174	0.62553335	0.78084995
ENSCGRG00001000856	65.6004277	1.01614438	0.02310541	0.18790682	0.80744951	0.89434934
ENSCGRG00001000863	12.2436567	-1.0370728	-0.0525172	0.21959025	0.25375074	0.44520653
ENSCGRG00001000869	115.300104	1.38943135	0.47449455	0.22472753	0.00404806	0.01870019
ENSCGRG00001000872	482.478907	-1.0499456	-0.0703146	0.1468639	0.52031983	0.70301625
ENSCGRG00001000878	2214.1475	-1.7548718	-0.8113657	0.08165922	2.54E-24	1.94E-22
ENSCGRG00001000880	163.700926	3.72388237	1.8968075	0.22593786	2.99E-18	1.57E-16
ENSCGRG00001000882	651.700632	-1.6422459	-0.7156702	0.10868984	4.11E-12	1.23E-10
ENSCGRG00001000897	1909.97317	-1.0088334	-0.012688	0.09247629	0.88014794	0.93575978
ENSCGRG00001000902	17699.1345	-1.0722884	-0.100693	0.06002697	0.07887133	0.19584061
ENSCGRG00001000908	6.656023	-1.0889681	-0.1229616	0.26468857	0.01813879	0.06346841
ENSCGRG00001000910	884.159127	1.05346409	0.07514113	0.07687325	0.2948086	0.49218013
ENSCGRG00001000919	8.21161761	1.02861416	0.04070192	0.21598564	0.3800503	0.58210352
ENSCGRG00001000923	19007.9018	-1.1193379	-0.1626456	0.05972038	0.00439806	0.01993599
ENSCGRG00001000935	1237.82291	-1.1247501	-0.1696045	0.09349456	0.04395568	0.12648174
ENSCGRG00001000944	16.9528408	-1.1019123	-0.1400093	0.25485986	0.12157782	0.26801793
ENSCGRG00001000959	54.6331088	1.00815328	0.011715	0.18410356	0.9042274	0.95011061
ENSCGRG00001000962	107.863303	1.03243908	0.04605665	0.15571445	0.67268403	0.81086907
ENSCGRG00001000963	9.88480851	1.06430169	0.08990716	0.230705	0.18471198	0.36152185
ENSCGRG00001000965	892.301779	1.08347454	0.11566525	0.09803793	0.18573203	0.36301912
ENSCGRG00001000966	764.499131	1.96213967	0.97242774	0.14636067	2.14E-12	6.64E-11
ENSCGRG00001000971	23.2870969	-1.0880149	-0.1216983	0.22959077	0.21341082	0.39931654
ENSCGRG00001000974	1119.51319	-1.3565681	-0.4399615	0.14234681	0.00032367	0.00222316

ENSCGRG00001000975	39.2558608	-1.0443106	-0.0625509	0.2088219	0.4387838	0.63641537
ENSCGRG00001000987	48.7463218	-1.0614647	-0.0860564	0.19201975	0.41746908	0.61645866
ENSCGRG00001000989	1463.78582	1.10389442	0.14260219	0.0793916	0.05270863	0.14464386
ENSCGRG00001000991	1485.16086	-1.0427155	-0.0603456	0.08240372	0.42661494	0.62469495
ENSCGRG00001000994	71.0483656	1.09526226	0.13127637	0.19499201	0.25986076	0.45214651
ENSCGRG00001001008	1570.05822	-1.0444362	-0.0627244	0.08554787	0.42354792	0.62192216
ENSCGRG00001001018	5.02369058	1.02348508	0.03349008	0.21477216	0.43612372	0.63377706
ENSCGRG00001001028	3097.21442	-1.1762879	-0.2342412	0.10128375	0.0093205	0.03688465
ENSCGRG00001001040	2.27858866	-1.0236654	-0.0337442	0.21777991	0.20959362	0.39495356
ENSCGRG00001001047	9.03090872	-1.0074992	-0.0107787	0.20492461	0.86544285	0.92774752
ENSCGRG00001001059	72.9073516	-1.0217559	-0.0310506	0.1949549	0.71792471	0.84155927
ENSCGRG00001001076	478.818952	1.01222409	0.01752871	0.10608381	0.85252623	0.92070514
ENSCGRG00001001109	44.7906206	1.1233256	0.16777615	0.24637898	0.14335975	0.3018472
ENSCGRG00001001113	6.31702909	-1.0077303	-0.0111096	0.20999143	0.81439714	0.89926966
ENSCGRG00001001115	554.971964	-1.0339497	-0.048166	0.19905784	0.57768595	0.74410617
ENSCGRG00001001119	8797.73649	-1.0862573	-0.1193659	0.05853148	0.03353438	0.10257294
ENSCGRG00001001124	3.25648129	1.03188927	0.04528817	0.21900373	0.24901509	0.44016604
ENSCGRG00001001128	8.36930911	-1.0423152	-0.0597917	0.2214078	0.23215285	0.42134853
ENSCGRG00001001149	526.4636	-1.002681	-0.0038627	0.11528221	0.96765845	0.98425695
ENSCGRG00001001153	382.884104	-1.0480085	-0.0676505	0.12499505	0.50173148	0.68681131
ENSCGRG00001001161	139.183023	-1.0078649	-0.0113023	0.14034918	0.91465962	0.9555461
ENSCGRG00001001163	615.8649	1.26977724	0.34457542	0.11445824	0.0006714	0.00416696
ENSCGRG00001001178	1956.75923	1.01096199	0.01572876	0.0695049	0.81266121	0.89781717
ENSCGRG00001001193	9.59864791	1.02622877	0.03735237	0.20757607	0.58408256	0.74880881
ENSCGRG00001001206	301.542675	1.01941002	0.02773444	0.11595703	0.77698406	0.87554239
ENSCGRG00001001212	87.6680016	-1.212138	-0.277554	0.22605005	0.05055269	0.14016883
ENSCGRG00001001221	13.7664534	-1.0533273	-0.0749538	0.22510604	0.21089309	0.3963521
ENSCGRG00001001223	8.89818486	1.00237702	0.00342525	0.2059248	0.95677406	0.97901352
ENSCGRG00001001241	138.718457	-1.6166087	-0.6929705	0.20578905	6.21E-05	0.00053265
ENSCGRG00001001250	1180.52762	-1.0567562	-0.0796426	0.08156079	0.28935906	0.48574196
ENSCGRG00001001264	13.8346557	-1.0258295	-0.036791	0.20898816	0.56133442	0.73404389
ENSCGRG00001001277	2.45544082	-1.011123	-0.0159585	0.21441965	0.56953615	0.73906967
ENSCGRG00001001286	117.372951	-1.1457908	-0.1963437	0.1777508	0.11356805	0.25554308
ENSCGRG00001001302	832.996975	1.0447989	0.06322528	0.09548423	0.46188684	0.65536299
ENSCGRG00001001308	2504.96933	1.36177289	0.44548612	0.06222722	1.45E-13	5.06E-12
ENSCGRG00001001335	18.1210525	1.05060989	0.07122707	0.21498871	0.35866214	0.55880103
ENSCGRG00001001337	672.990896	-1.060265	-0.0844249	0.19391227	0.41741173	0.61644504
ENSCGRG00001001356	8172.43416	-1.1602573	-0.2144447	0.07424705	0.00201934	0.01050251
ENSCGRG00001001380	4.64038063	-1.0293442	-0.0417254	0.21536777	0.3934563	0.59456014
ENSCGRG00001001389	81.8004049	1.01645826	0.02355097	0.17107566	0.81502391	0.89965156
ENSCGRG00001001399	280.562238	-2.8047155	-1.4878544	0.13733552	1.30E-28	1.28E-26
ENSCGRG00001001436	252.635171	-1.2965145	-0.3746383	0.16651336	0.00467493	0.02097578
ENSCGRG00001001443	867.793955	-1.4650991	-0.5509983	0.1358995	6.03E-06	6.53E-05
ENSCGRG00001001449	1043.09756	-1.1846587	-0.2444715	0.07583109	0.00057742	0.00367995
ENSCGRG00001001528	7.44069425	1.06032526	0.08450689	0.23684387	0.04877712	0.13672536
ENSCGRG00001001537	1359.76963	-1.4705903	-0.5563953	0.28519966	0.00404705	0.01870019
ENSCGRG00001001542	1136.1733	-1.9151253	-0.9374388	0.11882919	2.56E-16	1.15E-14
ENSCGRG00001001548	12.1015168	1.01492919	0.02137907	0.20836698	0.71318721	0.83814814
ENSCGRG00001001555	39.6324493	3.39385195	1.76292363	0.47953237	1.05E-05	0.00010857
ENSCGRG00001001576	101.737674	1.03174217	0.04508249	0.16193637	0.6801967	0.8159652

ENSCGRG00001001590	10.420448	-1.0000957	-0.000138	0.20655906	0.99595998	0.99827695
ENSCGRG00001001597	5236.27351	-1.1662858	-0.2219214	0.05709714	5.42E-05	0.00047067
ENSCGRG00001001599	309.001334	1.06240373	0.08733212	0.11335585	0.36711576	0.56851461
ENSCGRG00001001612	2.08865478	-1.0120861	-0.017332	0.2151095	0.47556011	0.66637271
ENSCGRG00001001613	15.1224909	-1.0186737	-0.026692	0.19931821	0.73615617	0.85248242
ENSCGRG00001001614	8555.03021	-1.0024662	-0.0035536	0.05432288	0.98939626	0.99506682
ENSCGRG00001001615	309.553633	-1.0325936	-0.0462726	0.14079687	0.66410568	0.80545292
ENSCGRG00001001617	2337.33679	1.16907485	0.22536731	0.05682567	3.83E-05	0.00034404
ENSCGRG00001001626	3.87667048	1.00283123	0.00407882	0.21035881	0.93079595	0.96546527
ENSCGRG00001001629	12995.4514	-1.0353733	-0.0501511	0.07145578	0.44755914	0.64331604
ENSCGRG00001001662	85.9948858	1.00590036	0.0084874	0.16903446	0.93654879	0.96922329
ENSCGRG00001001669	46.8725961	-1.0278348	-0.0396084	0.18764678	0.67990475	0.81587815
ENSCGRG00001001675	11867.6508	1.05718196	0.08022372	0.04916473	0.09382208	0.22277309
ENSCGRG00001001693	5403.06618	-1.1329318	-0.180061	0.05168689	0.00032822	0.00224961
ENSCGRG00001001706	502.097516	1.30569185	0.38481445	0.1251429	0.00044766	0.00295138
ENSCGRG00001001708	171.337492	-1.9997148	-0.9997942	0.19006517	9.93E-09	1.81E-07
ENSCGRG00001001711	161.705711	-1.0755374	-0.1050577	0.14891949	0.34397588	0.54332072
ENSCGRG00001001720	101.277882	-1.0912716	-0.1260102	0.19043214	0.27410643	0.46955159
ENSCGRG00001001728	201.965664	1.10699452	0.14664809	0.17724389	0.21705247	0.40331334
ENSCGRG00001001752	607.333793	-1.7232657	-0.7851452	0.13447364	4.41E-10	9.76E-09
ENSCGRG00001001753	231.736557	1.33142757	0.41297395	0.14936667	0.00098612	0.00576571
ENSCGRG00001001766	579.804445	-1.1142263	-0.1560423	0.13612295	0.15029245	0.31183889
ENSCGRG00001001785	2610.71808	-1.0976872	-0.134467	0.09664294	0.11840438	0.26309802
ENSCGRG00001001789	10.2097809	1.01750578	0.02503698	0.21065512	0.63225474	0.78541342
ENSCGRG00001001812	164.609301	1.03192759	0.04534174	0.14515027	0.67657	0.81340795
ENSCGRG00001001829	151.838991	-1.0957669	-0.1319409	0.18129902	0.25984131	0.45214651
ENSCGRG00001001839	482.321462	-1.1412776	-0.1906498	0.1416076	0.0878243	0.21261714
ENSCGRG00001001848	200.693228	-1.0176505	-0.0252422	0.12627778	0.80477267	0.89241152
ENSCGRG00001001852	3233.04571	-1.0283286	-0.0403014	0.06571238	0.51911936	0.70206071
ENSCGRG00001001865	304.031066	-1.3068443	-0.3860873	0.39651057	0.02568176	0.08324476
ENSCGRG00001001885	376.494334	1.13815397	0.18669573	0.10683826	0.04528541	0.12940133
ENSCGRG00001001892	470.250751	-1.0181864	-0.0260017	0.11119229	0.78404971	0.87922895
ENSCGRG00001001901	1.70428776	1.00195877	0.00282315	0.21402067	0.90997397	0.95305098
ENSCGRG00001001905	350.626252	1.02606328	0.03711971	0.12944758	0.72087233	0.84286004
ENSCGRG00001001907	2.54413836	-1.005371	-0.007728	0.21193823	0.84183002	0.91522931
ENSCGRG00001001946	5.84526834	-1.0888427	-0.1227955	0.26327425	0.02436451	0.08023378
ENSCGRG00001001964	10.4354404	-1.0198603	-0.0283716	0.21417647	0.47878558	0.66920275
ENSCGRG00001001975	23.7560326	-1.0496288	-0.0698792	0.21252322	0.37807767	0.58022942
ENSCGRG00001001981	175.334852	-1.2220509	-0.2893043	0.28745165	0.0501087	0.13923914
ENSCGRG00001001985	15649.4037	-1.2518591	-0.3240722	0.08251721	3.55E-05	0.00032076
ENSCGRG00001001996	16.7507889	1.05835519	0.08182388	0.21724566	0.31841896	0.51704233
ENSCGRG00001002019	979.816199	-1.0256072	-0.0364783	0.06950041	0.57868072	0.74508996
ENSCGRG00001002036	2.63683661	-1.0434772	-0.0613991	0.22622892	0.06323186	0.16670101
ENSCGRG00001002044	4.02166198	1.00204465	0.0029468	0.21197396	0.94054163	0.97124855
ENSCGRG00001002058	2.37461685	-1.0223204	-0.0318474	0.21635403	0.33721428	0.53697237
ENSCGRG00001002066	213.330272	-1.5542887	-0.6362545	0.2008963	0.00013617	0.00106622
ENSCGRG00001002078	6.48067791	-1.0332627	-0.0472071	0.21677123	0.34323745	0.5426227
ENSCGRG00001002082	37.7972567	1.11142868	0.15241537	0.24716072	0.14961073	0.31086999
ENSCGRG00001002091	3193.57952	-1.0251209	-0.0357941	0.05683137	0.51395794	0.69765509
ENSCGRG00001002095	992.472235	-1.1488737	-0.2002202	0.07667586	0.00509931	0.02249384

ENSCGRG00001002101	11.5460809	-1.0187633	-0.0268189	0.20573	0.68579244	0.81957283
ENSCGRG00001002109	495.692694	-1.0380876	-0.0539283	0.20685095	0.4895196	0.67718402
ENSCGRG00001002141	26.2640185	1.03566488	0.05055725	0.20098951	0.55569806	0.73050512
ENSCGRG00001002179	91.2508144	1.01931721	0.02760309	0.16314486	0.79767647	0.88792454
ENSCGRG00001002201	444.921509	-1.0510527	-0.0718351	0.11000977	0.44737717	0.64330021
ENSCGRG00001002207	2972.03568	-1.1337937	-0.1811582	0.08973549	0.02611775	0.08425293
ENSCGRG00001002209	2.16247984	-1.0305831	-0.0434608	0.22155184	0.01913182	0.06607673
ENSCGRG00001002211	109.243294	-1.0069191	-0.0099478	0.16344254	0.92458662	0.96122057
ENSCGRG00001002240	928.792229	-1.2510099	-0.3230933	0.29602534	0.03824675	0.11362264
ENSCGRG00001002258	171.350485	-1.0543819	-0.0763975	0.13534345	0.47172751	0.66339108
ENSCGRG00001002259	11.4248026	-1.079765	-0.1107174	0.24762706	0.08314415	0.20360859
ENSCGRG00001002262	2.5306463	-1.0108138	-0.0155173	0.21392531	0.61765403	0.77516881
ENSCGRG00001002267	1112.57731	-1.9280805	-0.9471653	0.11811983	7.58E-17	3.56E-15
ENSCGRG00001002297	336.96158	1.39471693	0.47997234	0.15082185	0.00020583	0.0015312
ENSCGRG00001002314	4.22334667	1.00444511	0.00639873	0.21129755	0.8804883	0.9359435
ENSCGRG00001002326	15668.5698	-1.1165455	-0.1590421	0.07849049	0.03072352	0.09566559
ENSCGRG00001002350	4.76606713	-1.0110601	-0.0158687	0.21233173	0.68909666	0.82183486
ENSCGRG00001002366	125.73953	1.01404697	0.02012448	0.14649309	0.85336438	0.92125381
ENSCGRG00001002380	13.3111269	-1.0882552	-0.1220169	0.25819899	0.05005636	0.13918427
ENSCGRG00001002386	7.50301374	-1.0203914	-0.0291227	0.21182945	0.56331554	0.7353474
ENSCGRG00001002391	735.127118	-1.5875174	-0.6667724	0.10230476	7.07E-12	2.04E-10
ENSCGRG00001002393	78.0210112	-1.5562622	-0.6380851	0.27616364	0.00149602	0.00813758
ENSCGRG00001002394	41.6486355	1.02986369	0.04245339	0.20017481	0.61135954	0.77127395
ENSCGRG00001002410	9.35493952	-18.681996	-4.2235767	1.02795506	6.43E-06	6.90E-05
ENSCGRG00001002434	6406.05111	1.14967763	0.20122939	0.06377609	0.00093131	0.00550283
ENSCGRG00001002440	69.466655	1.03594519	0.05094768	0.17128393	0.63584879	0.78791438
ENSCGRG00001002452	1817.91043	-1.0274176	-0.0390227	0.0741264	0.5739595	0.74199427
ENSCGRG00001002480	1097.99362	-1.2186456	-0.2852786	0.13063284	0.00929128	0.03678037
ENSCGRG00001002486	82.9962802	1.00782416	0.01124395	0.17207882	0.91507895	0.95558501
ENSCGRG00001002491	3272.64826	-1.2047079	-0.2686834	0.10422178	0.00372591	0.0174703
ENSCGRG00001002509	826.611443	-1.330007	-0.4114338	0.09889453	6.45E-06	6.92E-05
ENSCGRG00001002516	15.3328516	-1.0351859	-0.0498898	0.2104002	0.46753925	0.660123
ENSCGRG00001002524	5.70623355	1.01259155	0.01805235	0.21090921	0.70791804	0.83479979
ENSCGRG00001002537	177.503472	1.02915956	0.04146667	0.13118805	0.69158545	0.82348698
ENSCGRG00001002555	420.94552	-1.2362342	-0.3059521	0.14747535	0.01041918	0.04056831
ENSCGRG00001002557	290.909155	1.04948901	0.06968706	0.14257633	0.5210296	0.70338173
ENSCGRG00001002572	1514.46782	-1.2092525	-0.2741155	0.10026732	0.00231142	0.01178242
ENSCGRG00001002592	6.35580184	-1.0145439	-0.0208312	0.2133913	0.58295933	0.74830245
ENSCGRG00001002599	436.150991	1.12152144	0.16545721	0.1211728	0.10399734	0.24032874
ENSCGRG00001002604	2.6535499	-1.0277741	-0.0395233	0.21835826	0.22212368	0.41005972
ENSCGRG00001002626	2.78874041	-1.010883	-0.015616	0.21315777	0.66399296	0.80543405
ENSCGRG00001002667	30.7563521	1.01438325	0.02060283	0.19517958	0.80975855	0.89615612
ENSCGRG00001002681	4306.41323	-1.0130758	-0.0187421	0.08269618	0.802254	0.89062083
ENSCGRG00001002687	1.62716016	-1.0061405	-0.0088318	0.21448355	0.6932449	0.82455591
ENSCGRG00001002727	14.92017	-1.0831874	-0.1152829	0.24406269	0.12880493	0.27951711
ENSCGRG00001002729	5.11613021	1.0202737	0.02895622	0.21338614	0.51584251	0.6994012
ENSCGRG00001002732	798.856936	-1.082499	-0.1143657	0.07973891	0.12115107	0.26749666
ENSCGRG00001002740	2.35880947	1.0093085	0.0133672	0.21463209	0.58258206	0.74815801
ENSCGRG00001002744	465.739689	1.0458249	0.06464132	0.21076862	0.41391576	0.61262402
ENSCGRG00001002761	28.6527134	1.38132408	0.46605184	0.55714712	0.01812293	0.0634476

ENSCGRG00001002780	485.949161	-1.1457808	-0.196331	0.15247175	0.09177677	0.21917026
ENSCGRG00001002806	15.463795	-1.0944675	-0.1302291	0.25906162	0.07717041	0.19292603
ENSCGRG00001002809	188.579244	-1.1502997	-0.2020098	0.17976495	0.10504596	0.24171704
ENSCGRG00001002810	1818.4357	-1.2270069	-0.2951433	0.19948632	0.03331318	0.10206694
ENSCGRG00001002821	1.86868385	1.0010243	0.00147699	0.21353933	0.95989922	0.98105115
ENSCGRG00001002837	15.3334681	-1.0729549	-0.1015894	0.24248341	0.08831954	0.21340128
ENSCGRG00001002838	28.2709213	1.1261669	0.17142066	0.25872155	0.12483494	0.27359034
ENSCGRG00001002851	35.5880569	-1.0278297	-0.0396013	0.19523802	0.65478437	0.79990347
ENSCGRG00001002858	1009.81265	-1.0005791	-0.0008353	0.08340748	0.99057444	0.99554829
ENSCGRG00001002861	115.599236	1.00554379	0.00797591	0.15371225	0.94250206	0.97194103
ENSCGRG00001002914	2401.86367	1.18497279	0.24485393	0.07439805	0.00045737	0.00300613
ENSCGRG00001002939	2.28319147	1.02454178	0.03497882	0.21761947	0.23920447	0.42898071
ENSCGRG00001002941	1.73906855	-1.0179984	-0.0257353	0.21581685	0.36825938	0.5699168
ENSCGRG00001002948	36.769265	1.06634423	0.09267324	0.20986029	0.34027387	0.53986974
ENSCGRG00001002961	75.8651322	1.00366252	0.00527424	0.17079537	0.94662715	0.97368667
ENSCGRG00001002969	3.65857368	1.00882336	0.01267359	0.2126008	0.73378848	0.8512089
ENSCGRG00001002972	9191.34518	-1.0160855	-0.0230218	0.07487804	0.74275365	0.85640632
ENSCGRG00001002978	402.613276	1.09632667	0.13267774	0.11074111	0.16415604	0.33225452
ENSCGRG00001002982	32312.2135	-1.1201236	-0.1636579	0.04339692	0.00013127	0.00103165
ENSCGRG00001002984	2.53651238	1.00504198	0.00725576	0.21339189	0.81386772	0.89891753
ENSCGRG00001002989	279.214865	-1.2079992	-0.2726195	0.13599625	0.01490338	0.0542827
ENSCGRG00001002994	4.21430252	-1.0029737	-0.0042838	0.21149252	0.91435748	0.9555461
ENSCGRG00001003022	996.422325	1.00804336	0.0115577	0.07805881	0.87435723	0.93221108
ENSCGRG00001003032	49.507493	-1.0153462	-0.0219717	0.17591604	0.82872693	0.90788335
ENSCGRG00001003056	5.29190642	1.02508886	0.03574897	0.21470709	0.44116907	0.6386457
ENSCGRG00001003060	3637.33647	-1.051114	-0.0719191	0.05767592	0.19484749	0.37480411
ENSCGRG00001003081	7223.43545	-1.0788273	-0.1094639	0.07030163	0.09877505	0.2308957
ENSCGRG00001003095	40.7382472	-1.0613206	-0.0858606	0.19634218	0.40586895	0.60533021
ENSCGRG00001003115	3.73598498	-1.0140032	-0.0200622	0.21403101	0.55990521	0.73310886
ENSCGRG00001003128	14113.7918	-1.1262966	-0.1715868	0.14911295	0.13262063	0.28504534
ENSCGRG00001003134	704.660301	-1.0695449	-0.096997	0.2062133	0.33470174	0.534538
ENSCGRG00001003137	2.81774542	-1.0246191	-0.0350878	0.21793952	0.21261094	0.39847054
ENSCGRG00001003161	38.2661152	-1.11149	-0.152495	0.25265116	0.1353024	0.2894184
ENSCGRG00001003176	14.3032483	-1.009163	-0.0131591	0.20484078	0.83748691	0.91296122
ENSCGRG00001003185	3419.45515	1.0052007	0.00748358	0.06598972	0.90590666	0.95112403
ENSCGRG00001003198	32308.9159	-1.0678969	-0.0947723	0.04500086	0.03166287	0.0979477
ENSCGRG00001003235	37.2620748	1.02134836	0.03047502	0.18873531	0.74814076	0.86041394
ENSCGRG00001003251	72.3830759	1.04609967	0.06502032	0.17923336	0.54281968	0.72049736
ENSCGRG00001003263	2479.87501	-1.2720495	-0.3471548	0.09027264	3.17E-05	0.00029067
ENSCGRG00001003291	3.04828397	-1.0297626	-0.0423118	0.21934197	0.17775013	0.35198317
ENSCGRG00001003307	225.459948	-1.2958523	-0.3739013	0.1723693	0.00566164	0.02445234
ENSCGRG00001003308	5175.54498	-1.0319113	-0.045319	0.05965837	0.42827835	0.62608502
ENSCGRG00001003314	23.3026597	-2.1487229	-1.1034794	0.66263439	0.00283121	0.01394917
ENSCGRG00001003341	29.6053974	1.07699612	0.10701306	0.21741617	0.27662827	0.47229216
ENSCGRG00001003387	7.15061699	-1.0123019	-0.0176396	0.2110938	0.70352184	0.83152932
ENSCGRG00001003393	118.871805	1.22128518	0.28840012	0.22233612	0.04395759	0.12648174
ENSCGRG00001003395	24.5132577	1.0183474	0.02622981	0.20077312	0.73570417	0.85219011
ENSCGRG00001003399	653.053104	-1.0229667	-0.0327592	0.08615274	0.67106105	0.81006985
ENSCGRG00001003405	5046.27758	-1.048111	-0.0677915	0.04762271	0.14280081	0.30105784
ENSCGRG00001003410	1180.50504	1.03539077	0.05017536	0.0996878	0.57610588	0.7435684

ENSCGRG00001003416	41.2338202	1.02776774	0.03951428	0.18470177	0.69040329	0.82279048
ENSCGRG00001003420	46.7981257	1.02591894	0.03691674	0.21271584	0.49529268	0.68187813
ENSCGRG00001003431	6.14151087	-1.0086204	-0.0123834	0.20836849	0.81774526	0.90164545
ENSCGRG00001003437	117.351225	1.18134238	0.24042715	0.21716667	0.07646074	0.19167555
ENSCGRG00001003451	22.6803436	-1.0210097	-0.0299966	0.20162493	0.69195445	0.82355631
ENSCGRG00001003454	2.5434317	-1.0275554	-0.0392161	0.219592	0.11138394	0.2523127
ENSCGRG00001003461	2.51030456	-1.0391395	-0.0553894	0.22557751	0.00740697	0.0304993
ENSCGRG00001003467	87.7232163	-1.1755798	-0.2333724	0.20337094	0.07927268	0.19668469
ENSCGRG00001003470	126.993258	-1.3159369	-0.3960903	0.22642052	0.01185935	0.04522724
ENSCGRG00001003473	1542.94582	-1.2282578	-0.2966134	0.11709953	0.00355972	0.01682659
ENSCGRG00001003477	37.1221474	-1.0679265	-0.0948123	0.20824023	0.33570063	0.5354657
ENSCGRG00001003491	150.541241	-1.2341846	-0.3035582	0.23258472	0.03786241	0.11274232
ENSCGRG00001003493	53.7538048	-1.0469314	-0.0661669	0.19777939	0.4843504	0.67336397
ENSCGRG00001003542	21688.8735	-1.0649337	-0.0907637	0.04395856	0.03221995	0.09931126
ENSCGRG00001003559	25.1542513	1.02953136	0.04198777	0.20599984	0.57028953	0.73956356
ENSCGRG00001003560	16.2087571	-1.0178237	-0.0254877	0.2043207	0.71107504	0.83690474
ENSCGRG00001003565	1834.95644	1.65127406	0.72357959	0.08690892	8.14E-18	4.17E-16
ENSCGRG00001003575	52.7584974	1.14243746	0.19211519	0.24114287	0.12511907	0.27396397
ENSCGRG00001003586	10.8276375	-1.0247422	-0.035261	0.20837149	0.58134023	0.74746245
ENSCGRG00001003587	6.38955741	-1.0443758	-0.062641	0.22218478	0.22521155	0.41388915
ENSCGRG00001003601	205.078931	-1.2111649	-0.2763953	0.20907669	0.04626274	0.13128616
ENSCGRG00001003616	1434.37937	1.08317574	0.11526733	0.07414746	0.09674223	0.22768104
ENSCGRG00001003623	170.759271	-1.0719152	-0.1001908	0.22839682	0.21888805	0.40578234
ENSCGRG00001003638	5.2883748	1.02272965	0.03242483	0.21405093	0.4684532	0.66081769
ENSCGRG00001003654	1.59672774	-1.0092981	-0.0133524	0.2151706	0.48971491	0.67723718
ENSCGRG00001003683	3.65061828	1.04577011	0.06456574	0.22955127	0.00222309	0.01138654
ENSCGRG00001003715	15.1363242	-1.0401939	-0.0568525	0.20641356	0.48052531	0.6709755
ENSCGRG00001003727	821.609356	-1.1243989	-0.169154	0.09696514	0.05050646	0.14007095
ENSCGRG00001003737	11492.9706	-1.0497117	-0.0699931	0.14224624	0.51646875	0.69986443
ENSCGRG00001003739	5.86248049	-1.0232102	-0.0331026	0.21445334	0.4462049	0.64259525
ENSCGRG00001003742	1.78518135	-1.0080327	-0.0115425	0.21372272	0.70154479	0.83028433
ENSCGRG00001003746	57.0105303	-1.0012262	-0.0017679	0.17592707	0.98455822	0.99183633
ENSCGRG00001003753	867.963051	1.10984979	0.15036443	0.08983715	0.06537166	0.1709766
ENSCGRG00001003755	5045.96526	-1.3832136	-0.468024	0.08973124	3.02E-08	5.13E-07
ENSCGRG00001003760	654.500417	1.01812001	0.02590763	0.10506524	0.76332558	0.86909614
ENSCGRG00001003795	667.943577	1.04267042	0.0602832	0.17336208	0.57690509	0.74394362
ENSCGRG00001003799	332.140382	-1.6282866	-0.7033547	0.18371018	1.05E-05	0.00010874
ENSCGRG00001003808	2.63889826	-1.0194181	-0.0277459	0.21444092	0.47288293	0.66421385
ENSCGRG00001003813	56.7923118	-1.0528831	-0.0743453	0.18157901	0.48849495	0.67686537
ENSCGRG00001003815	12.5399939	-1.0435351	-0.0614791	0.21872823	0.30464727	0.50264445
ENSCGRG00001003833	17.098395	1.00913565	0.01312011	0.20462249	0.84393706	0.91592523
ENSCGRG00001003851	966.063816	1.00817789	0.01175022	0.09331517	0.88912135	0.94090413
ENSCGRG00001003858	3.11078077	1.01615522	0.02312079	0.21463099	0.48397113	0.67307243
ENSCGRG00001003886	10.5206612	1.04246189	0.05999464	0.21702691	0.34465824	0.5440631
ENSCGRG00001003889	660.670101	1.0041223	0.00593499	0.08535399	0.94040838	0.97118925
ENSCGRG00001003901	10196.2396	-1.2160163	-0.2821625	0.1424121	0.0152914	0.05538107
ENSCGRG00001003904	1.63131441	-1.0112721	-0.0161712	0.21476506	0.53758053	0.7163492
ENSCGRG00001003912	2.05865228	1.02644434	0.0376554	0.21790268	0.25265464	0.44390425
ENSCGRG00001003914	17.9452333	-1.1060052	-0.1453581	0.26550005	0.08932836	0.2150933
ENSCGRG00001003940	264.73021	-2.4400329	-1.2869006	0.12941965	1.70E-24	1.31E-22

ENSCGRG00001003984	96.0944664	-1.0936001	-0.1290852	0.16858179	0.26568582	0.45955109
ENSCGRG00001003985	135.576271	-1.035778	-0.0507148	0.14593136	0.63906459	0.7904701
ENSCGRG00001003986	76.1864998	1.09746209	0.13417111	0.19446118	0.2519001	0.44320042
ENSCGRG00001003991	9.9588013	-1.0423728	-0.0598713	0.21829188	0.31158804	0.51021894
ENSCGRG00001004002	327.447421	-1.5248884	-0.6087036	0.20737493	0.00029849	0.00207697
ENSCGRG00001004022	13.8807948	-1.0334461	-0.0474631	0.21188574	0.45483635	0.64934566
ENSCGRG00001004023	329.006996	1.00688129	0.0098936	0.12198238	0.92255483	0.96041669
ENSCGRG00001004029	1268.59789	-1.1055053	-0.1447059	0.06691317	0.0223515	0.07505181
ENSCGRG00001004051	178.425715	1.17140665	0.22824199	0.17877914	0.07287494	0.18452815
ENSCGRG00001004057	1633.4201	-1.181508	-0.2406294	0.07152811	0.00035509	0.00241311
ENSCGRG00001004119	22.4938981	-1.0237285	-0.0338331	0.21208318	0.52060307	0.70309668
ENSCGRG00001004140	72.049075	-1.0016523	-0.0023818	0.1710981	0.98042909	0.99079336
ENSCGRG00001004154	4.98554238	-1.0297745	-0.0423285	0.21787511	0.27415824	0.46957709
ENSCGRG00001004162	117.353142	1.1124046	0.15368161	0.17102578	0.19718477	0.37797114
ENSCGRG00001004172	8.05634868	-1.030878	-0.0438736	0.21185085	0.47623035	0.66701408
ENSCGRG00001004203	14.862999	-1.0198873	-0.0284097	0.20060125	0.71362827	0.83844613
ENSCGRG00001004206	5.58377658	1.01910699	0.02730551	0.21091824	0.60600275	0.76704121
ENSCGRG00001004211	3612.18011	-1.0518007	-0.0728613	0.05066943	0.13814527	0.29381387
ENSCGRG00001004216	12.9348176	1.0242362	0.03454846	0.21145345	0.53954678	0.7182266
ENSCGRG00001004239	19.6182298	-1.0581813	-0.0815868	0.21434895	0.33660927	0.53651422
ENSCGRG00001004263	3956.59875	-1.3003198	-0.3788665	0.08761106	3.42E-06	3.92E-05
ENSCGRG00001004307	2897.86023	-1.0269026	-0.0382993	0.13253038	0.71195502	0.83733413
ENSCGRG00001004312	131.632726	-1.022132	-0.0315815	0.13572768	0.76352366	0.86923666
ENSCGRG00001004317	146.18978	-1.1984965	-0.2612257	0.17989275	0.04366157	0.12596329
ENSCGRG00001004340	1.84241148	-1.0050253	-0.0072319	0.21410519	0.77332418	0.87386763
ENSCGRG00001004377	307.564923	-1.1768434	-0.2349223	0.14374508	0.04022316	0.11812441
ENSCGRG00001004412	19.9739892	1.00701114	0.01007964	0.19595289	0.90380858	0.94993337
ENSCGRG00001004433	92.5421338	-1.078355	-0.1088322	0.18517482	0.33657484	0.53651422
ENSCGRG00001004440	15.7693206	1.09230557	0.1273765	0.25119899	0.11847279	0.26320438
ENSCGRG00001004444	3.88029005	-1.0639867	-0.0894801	0.24002741	0.03165953	0.0979477
ENSCGRG00001004454	10.8322823	-1.0231238	-0.0329807	0.20575915	0.63342721	0.78610759
ENSCGRG00001004474	69.826315	-1.0566058	-0.0794373	0.17331762	0.47077973	0.66270466
ENSCGRG00001004476	5.73495828	-1.0554885	-0.0779109	0.23163648	0.08573208	0.20845962
ENSCGRG00001004479	302.554617	1.03459955	0.04907247	0.12543325	0.63266196	0.78561455
ENSCGRG00001004486	25.7848098	-1.0208058	-0.0297084	0.19258996	0.739493	0.85480106
ENSCGRG00001004493	19.8754857	-1.1600689	-0.2142105	0.36422898	0.02510602	0.08189666
ENSCGRG00001004505	345.106661	1.2091286	0.27396769	0.11035168	0.00470724	0.0210986
ENSCGRG00001004537	2881.5629	-1.0638903	-0.0893495	0.08260737	0.2397345	0.42939023
ENSCGRG00001004551	6.88985764	1.0280497	0.03991002	0.21192064	0.49228586	0.67949091
ENSCGRG00001004565	1414.58469	-1.0280262	-0.0398771	0.17201244	0.70408771	0.83189112
ENSCGRG00001004584	276.814981	1.01555984	0.02227525	0.11572009	0.82055196	0.90334055
ENSCGRG00001004625	69.2593	1.06708495	0.09367503	0.17626505	0.40271612	0.60245165
ENSCGRG00001004643	40.0597312	1.09941254	0.13673284	0.23219486	0.19092188	0.36962885
ENSCGRG00001004644	331.459569	-1.0896238	-0.1238302	0.12901955	0.23570154	0.42542782
ENSCGRG00001004646	13.4660913	-1.0210264	-0.0300201	0.20857647	0.62143859	0.77778489
ENSCGRG00001004660	999.948631	-1.1026655	-0.1409953	0.09210302	0.08956745	0.21542603
ENSCGRG00001004664	130.895295	-1.0019539	-0.0028161	0.15143109	0.97859893	0.99027193
ENSCGRG00001004696	4.66727197	-1.0035057	-0.0050488	0.2099066	0.91404149	0.95543627
ENSCGRG00001004701	298.623096	1.25377745	0.32628128	0.14554441	0.00632874	0.02674731
ENSCGRG00001004715	5153.13722	1.20668594	0.27105024	0.05666485	7.19E-07	9.53E-06

ENSCGRG00001004717	608.294801	1.03196347	0.0453919	0.17280242	0.67086615	0.81004995
ENSCGRG00001004726	2.82010277	1.01810031	0.02587971	0.21505788	0.43934363	0.63688683
ENSCGRG00001004735	12.3815202	1.06800794	0.09492237	0.2277852	0.22299884	0.41130856
ENSCGRG00001004758	1546.18305	1.0688151	0.0960123	0.07679657	0.18001419	0.35547739
ENSCGRG00001004778	1926.09635	-1.1170111	-0.1596435	0.16720252	0.1786958	0.3535279
ENSCGRG00001004790	1970.91408	1.05573972	0.07825421	0.16369576	0.48323226	0.67255597
ENSCGRG00001004791	237.94918	1.06494002	0.09077217	0.15515344	0.4048329	0.60427423
ENSCGRG00001004794	7.92228381	-1.0059025	-0.0084905	0.20621433	0.88699996	0.93927995
ENSCGRG00001004798	1571.115	1.11251848	0.1538293	0.08223759	0.0426162	0.12359374
ENSCGRG00001004831	15.1637888	-1.06234	-0.0872456	0.21924779	0.29271581	0.4897074
ENSCGRG00001004833	594.789627	1.04827505	0.06801731	0.10794398	0.46642859	0.65941399
ENSCGRG00001004842	48.1676733	-1.0522277	-0.0734469	0.19829159	0.45215891	0.6470374
ENSCGRG00001004922	414.410812	1.1979238	0.26053615	0.12238439	0.01239025	0.04669583
ENSCGRG00001004929	1774.06877	-1.1827469	-0.2421414	0.07849883	0.00094079	0.00553841
ENSCGRG00001004942	74.2943721	-1.3481525	-0.4309837	0.38916927	0.01973394	0.06790002
ENSCGRG00001004945	2335.72883	-1.1662718	-0.221904	0.07245621	0.00112086	0.00640708
ENSCGRG00001004958	2281.60289	-1.7486022	-0.8062021	0.07654939	5.30E-27	4.68E-25
ENSCGRG00001004965	6.63316245	1.01015173	0.01457201	0.21086226	0.75150584	0.86237477
ENSCGRG00001004976	5271.15976	-1.0594336	-0.0832932	0.07963027	0.25914049	0.45146058
ENSCGRG00001004977	209.052726	-1.037921	-0.0536967	0.21986274	0.25255177	0.44390425
ENSCGRG00001004990	40362.0125	-1.1811418	-0.2401822	0.05378477	3.38E-06	3.88E-05
ENSCGRG00001005018	4.73531615	1.0236645	0.03374296	0.21490873	0.43434816	0.63198545
ENSCGRG00001005025	22.9683582	-1.8594109	-0.8948457	0.59162328	0.004071	0.01877173
ENSCGRG00001005028	942.455147	1.21787307	0.28436378	0.08408497	0.00026298	0.00187545
ENSCGRG00001005047	15.4412914	1.01788648	0.02557667	0.20090913	0.73136821	0.8493483
ENSCGRG00001005050	152.03012	-1.0166638	-0.0238427	0.13899046	0.82154333	0.90376956
ENSCGRG00001005051	7.23422161	1.03614545	0.05122653	0.22118418	0.18347851	0.35987746
ENSCGRG00001005056	1.5861848	-1.0116567	-0.0167199	0.21418376	0.58227164	0.74804864
ENSCGRG00001005061	7056.45122	-1.0885959	-0.1224686	0.05563296	0.02237424	0.07508881
ENSCGRG00001005084	3998.34984	1.03945659	0.05582951	0.05257879	0.27284286	0.46863999
ENSCGRG00001005091	1614.09675	-1.0167716	-0.0239957	0.07134244	0.7209943	0.84292571
ENSCGRG00001005107	1959.88283	1.03191687	0.04532675	0.06534002	0.46654452	0.65950511
ENSCGRG00001005154	8.94205942	-1.0051159	-0.0073618	0.20923408	0.8826771	0.93698481
ENSCGRG00001005187	2.28566704	1.03242465	0.0460365	0.2217389	0.06341726	0.16705607
ENSCGRG00001005202	2866.37608	-1.0888527	-0.1228088	0.0743017	0.07855358	0.19534763
ENSCGRG00001005205	555.863525	-1.0648689	-0.0906758	0.10930806	0.33544342	0.53526988
ENSCGRG00001005224	1.62480788	1.00280744	0.00404461	0.21436073	0.8546166	0.92229474
ENSCGRG00001005229	1885.62259	1.05933004	0.08315214	0.09081601	0.31333438	0.51209664
ENSCGRG00001005235	122.890511	-1.0682293	-0.0952214	0.17158533	0.39592019	0.59657104
ENSCGRG00001005241	1738.642	1.0522955	0.07353989	0.07512837	0.29602404	0.49369457
ENSCGRG00001005246	2.65575582	-1.0205008	-0.0292773	0.21690891	0.26330584	0.45648231
ENSCGRG00001005253	16.2899607	-1.0516738	-0.0726872	0.2235194	0.23320231	0.42258342
ENSCGRG00001005261	287.556472	-1.1325611	-0.1795889	0.12407767	0.08203103	0.20165119
ENSCGRG00001005262	548.232027	-1.1928521	-0.2544152	0.16703081	0.04209052	0.12237394
ENSCGRG00001005264	1.62295811	-1.0022593	-0.0032558	0.21346921	0.90829303	0.95230265
ENSCGRG00001005285	1513.47532	-1.1199274	-0.1634053	0.09309688	0.05113613	0.14141921
ENSCGRG00001005287	917.691208	-1.0170296	-0.0243617	0.09128909	0.76765479	0.87127745
ENSCGRG00001005302	13.5428414	-1.0945783	-0.1303752	0.25984046	0.07316063	0.18503211
ENSCGRG00001005313	1296.35024	-1.0511717	-0.0719983	0.06826319	0.26497359	0.45869182
ENSCGRG00001005314	957.15163	-1.0933994	-0.1288205	0.10715249	0.16586457	0.33486606

ENSCGRG00001005335	7428.43298	-1.1037774	-0.1424492	0.06818677	0.02691461	0.08620179
ENSCGRG00001005343	10.9031722	-1.0986555	-0.135739	0.26746956	0.05234577	0.1439565
ENSCGRG00001005357	809.773111	-1.2966409	-0.374779	0.0916022	1.00E-05	0.00010439
ENSCGRG00001005360	30.675869	-1.0353332	-0.0500951	0.19999967	0.56098028	0.73388045
ENSCGRG00001005375	1588.41534	-1.3337301	-0.4154667	0.08695299	3.51E-07	4.91E-06
ENSCGRG00001005376	1222.45162	-1.0058164	-0.008367	0.08232909	0.91160657	0.95425818
ENSCGRG00001005401	3.85737481	-1.0383333	-0.0542696	0.22078721	0.21494702	0.40108833
ENSCGRG00001005416	6.75822816	-1.0581813	-0.0815868	0.2310619	0.13049667	0.28194676
ENSCGRG00001005422	78.4457029	1.80945912	0.85555852	0.46592085	0.00269701	0.0134326
ENSCGRG00001005426	1631.70446	1.04453389	0.0628593	0.07531746	0.37258947	0.57414546
ENSCGRG00001005428	2.59352409	-1.0208295	-0.0297419	0.21727582	0.20126302	0.38365763
ENSCGRG00001005431	17.304696	-1.0184137	-0.0263238	0.21757191	0.05940267	0.15886183
ENSCGRG00001005432	15.7105331	1.02828707	0.04024308	0.20481585	0.59186935	0.75498025
ENSCGRG00001005434	509.015402	-1.0889845	-0.1229835	0.12422541	0.22987371	0.41853384
ENSCGRG00001005440	603.857842	1.07041091	0.09816473	0.22407923	0.2501357	0.44147675
ENSCGRG00001005448	5197.61542	-1.0673632	-0.0940511	0.18569959	0.39099479	0.59245839
ENSCGRG00001005467	22.9982355	1.07249241	0.10096744	0.22096342	0.26921521	0.46421414
ENSCGRG00001005474	541.072226	-1.0717131	-0.0999188	0.12483787	0.32822526	0.52808662
ENSCGRG00001005478	956.101886	-1.137322	-0.1856407	0.0853722	0.01766064	0.06225447
ENSCGRG00001005485	4.12443192	1.01331942	0.01908901	0.21189558	0.66369898	0.80543405
ENSCGRG00001005502	612.396559	1.02490415	0.03548899	0.10513772	0.69982963	0.82907773
ENSCGRG00001005504	824.184029	1.29974701	0.37823084	0.1098436	0.00013079	0.00102916
ENSCGRG00001005508	6.27670458	-1.0240857	-0.0343365	0.21392427	0.46937261	0.66138633
ENSCGRG00001005511	55.7956935	-1.1389911	-0.1877565	0.25210876	0.12207436	0.26892047
ENSCGRG00001005522	18737.5062	-1.0084383	-0.0121228	0.04569081	0.78539572	0.87991247
ENSCGRG00001005533	10.8055583	1.06054806	0.08480999	0.23333121	0.11695871	0.26106308
ENSCGRG00001005534	1.9062141	-1.0097271	-0.0139655	0.21431749	0.61158945	0.77141206
ENSCGRG00001005540	2.0621772	1.00308298	0.00444096	0.21406683	0.85635903	0.92339724
ENSCGRG00001005542	8.99892065	-1.0222857	-0.0317984	0.20886664	0.60070294	0.76301484
ENSCGRG00001005545	9285.51961	-1.1270217	-0.1725153	0.17082808	0.15230505	0.31468189
ENSCGRG00001005565	13.8873553	1.03061332	0.04350315	0.20506949	0.56811422	0.73836894
ENSCGRG00001005589	4.33653341	1.01010686	0.01450793	0.21432778	0.60397043	0.76556019
ENSCGRG00001005604	3.99316309	-1.0247846	-0.0353207	0.21540561	0.39997423	0.6001038
ENSCGRG00001005629	27.3733836	-1.0310192	-0.0440711	0.19410958	0.62823069	0.7826155
ENSCGRG00001005631	6.08842721	1.00090266	0.00130168	0.20945465	0.98036676	0.99079336
ENSCGRG00001005641	2.58182361	-1.0256182	-0.0364937	0.21744246	0.26352976	0.45674689
ENSCGRG00001005651	79.1129536	-1.0502757	-0.0707681	0.17147776	0.5179394	0.70084827
ENSCGRG00001005658	139.244389	-1.0398431	-0.0563659	0.14350064	0.59979346	0.76253989
ENSCGRG00001005673	15.6518139	-1.164523	-0.2197391	0.35398101	0.03864683	0.11454556
ENSCGRG00001005689	1.91658713	1.01614826	0.02311092	0.21522752	0.42711088	0.62529032
ENSCGRG00001005692	12.5868811	-1.0335712	-0.0476378	0.21250517	0.44348612	0.64035915
ENSCGRG00001005701	29.364395	1.02797521	0.03980547	0.19065136	0.67278911	0.81086907
ENSCGRG00001005713	203.680948	1.00948107	0.01361386	0.12952855	0.89615316	0.9455619
ENSCGRG00001005717	3.55052371	-1.0083412	-0.0119838	0.21275923	0.73434762	0.8514657
ENSCGRG00001005719	9.2780344	-1.0656089	-0.0916781	0.23123859	0.1792718	0.35448155
ENSCGRG00001005727	486.444406	1.03403801	0.04828922	0.17302952	0.65166914	0.79791215
ENSCGRG00001005732	1.74733671	-1.0029897	-0.0043068	0.21367757	0.87364234	0.93213247
ENSCGRG00001005737	1621.79642	-1.1263291	-0.1716284	0.11525009	0.08080158	0.19943512
ENSCGRG00001005738	12.8677908	-1.1811745	-0.2402221	0.42584759	0.01558668	0.05624378
ENSCGRG00001005746	63.6499879	-1.103505	-0.1420932	0.20006996	0.2273208	0.41591083

ENSCGRG00001005773	2.30663727	1.02149462	0.03068161	0.2164992	0.32339486	0.52273667
ENSCGRG00001005784	76.1632915	-1.0818434	-0.1134917	0.21011812	0.28291648	0.47906941
ENSCGRG00001005805	2831.17015	-1.1440133	-0.1941038	0.07494569	0.00548768	0.02383765
ENSCGRG00001005818	14.671205	1.00413825	0.00595791	0.21448174	0.78673568	0.88056655
ENSCGRG00001005824	2.97038521	1.02332134	0.03325925	0.21716227	0.28016444	0.47591917
ENSCGRG00001005827	4.66831755	-1.074542	-0.1037219	0.24954805	0.02403043	0.0793785
ENSCGRG00001005837	1887.38877	-1.1121558	-0.153359	0.05863289	0.00639638	0.0269709
ENSCGRG00001005842	614.387177	1.01857874	0.02655751	0.10604373	0.77370512	0.87386763
ENSCGRG00001005843	310.851417	-1.0595715	-0.0834809	0.15954157	0.45305295	0.64794108
ENSCGRG00001005844	1564.41256	-1.0516743	-0.072688	0.07422588	0.29544857	0.49292735
ENSCGRG00001005849	2.80404746	-1.0482772	-0.0680203	0.2293754	0.04338602	0.12537187
ENSCGRG00001005859	1.83523777	-1.0181699	-0.0259783	0.21585515	0.36054465	0.56088863
ENSCGRG00001005867	6376.5138	1.01479676	0.02119081	0.05007841	0.66402724	0.80543405
ENSCGRG00001005891	17.0278548	-1.0624117	-0.0873429	0.2276336	0.2076924	0.39250162
ENSCGRG00001005906	15.1918425	1.01461664	0.02093472	0.20690713	0.73492173	0.85171009
ENSCGRG00001005908	6.79702051	-1.0228292	-0.0325652	0.21047861	0.57107124	0.73980047
ENSCGRG00001005913	25.905664	-1.318461	-0.3988549	0.61818044	0.01999328	0.06857132
ENSCGRG00001005947	4.41772631	1.00778947	0.01119429	0.21218152	0.77575594	0.87508221
ENSCGRG00001005949	4512.48052	-1.0126729	-0.0181683	0.07027461	0.78417501	0.87922895
ENSCGRG00001005965	14.4820634	-1.0230784	-0.0329168	0.20513097	0.64051203	0.79068702
ENSCGRG00001005974	11.4245568	1.01903667	0.02720597	0.20733131	0.66979263	0.80967875
ENSCGRG00001005994	474.759769	1.01628936	0.02331123	0.09723185	0.78937384	0.88220895
ENSCGRG00001006003	12.7874353	-1.1898891	-0.2508271	0.51834714	0.00219152	0.01125897
ENSCGRG00001006007	28.46763	-1.0881651	-0.1218975	0.25506516	0.0720579	0.18300194
ENSCGRG00001006025	13.0314234	-1.00172	-0.0024794	0.20511098	0.96624828	0.98352328
ENSCGRG00001006054	774.879431	-1.0517561	-0.0728003	0.11178691	0.44626958	0.64261615
ENSCGRG00001006092	2.58249066	-1.0304802	-0.0433168	0.21972212	0.1695328	0.34052128
ENSCGRG00001006098	1491.7358	-1.0175068	-0.0250384	0.07507935	0.72126148	0.84308418
ENSCGRG00001006102	1011.70659	1.28079396	0.35703841	0.21643234	0.01711987	0.06063189
ENSCGRG00001006103	3.58341047	-1.0292194	-0.0415506	0.21876968	0.21675585	0.40302361
ENSCGRG00001006130	1705.01398	-1.1841535	-0.2438561	0.19992485	0.06707235	0.17406742
ENSCGRG00001006154	9.22987962	-1.0482075	-0.0679243	0.22365864	0.21286181	0.39878562
ENSCGRG00001006162	132.335951	1.01564292	0.02239327	0.16834491	0.83403545	0.91104481
ENSCGRG00001006166	25.2279009	1.07049066	0.09827221	0.22355335	0.25651272	0.44859084
ENSCGRG00001006180	2.40355635	1.00883351	0.0126881	0.21304908	0.7179847	0.84155927
ENSCGRG00001006211	34.4991936	1.0043896	0.006319	0.19378789	0.94248769	0.97194103
ENSCGRG00001006227	8.20727162	-1.0227919	-0.0325126	0.20845748	0.60297914	0.76484432
ENSCGRG00001006245	289.495382	-1.1017352	-0.1397775	0.15120587	0.21654913	0.40295557
ENSCGRG00001006248	880.100774	-1.0842425	-0.1166875	0.10015052	0.18733379	0.36486938
ENSCGRG00001006251	939.630811	-1.0383991	-0.054361	0.08897653	0.50131869	0.6865397
ENSCGRG00001006273	389.153123	1.11937255	0.16269027	0.10892975	0.08505636	0.20737951
ENSCGRG00001006277	2882.95355	1.01595793	0.02284066	0.06150208	0.69873499	0.82824051
ENSCGRG00001006281	765.700613	1.02639794	0.03759017	0.09695323	0.66595497	0.80655133
ENSCGRG00001006297	248.098455	-1.1257433	-0.1708779	0.17754662	0.15960457	0.32567047
ENSCGRG00001006312	5846.0569	-1.132539	-0.1795607	0.06443359	0.00332631	0.01592744
ENSCGRG00001006325	150.134589	1.20484569	0.26884839	0.20467082	0.0502184	0.13948347
ENSCGRG00001006338	716.598074	-2.0891479	-1.0629146	0.13340712	8.91E-17	4.16E-15
ENSCGRG00001006339	3.87160737	1.00866851	0.01245213	0.21193999	0.76259954	0.86877882
ENSCGRG00001006366	412.771377	1.27836705	0.35430213	0.15428815	0.00464389	0.02088037
ENSCGRG00001006389	11.8297742	1.03552721	0.05036547	0.20971884	0.48177014	0.67176179

ENSCGRG00001006395	21.7151172	1.00926643	0.01330708	0.20467366	0.83971407	0.9142043
ENSCGRG00001006400	18.7218031	-1.0214657	-0.0306408	0.20526767	0.65769985	0.80140161
ENSCGRG00001006451	14.9467402	1.00124505	0.00179511	0.2002949	0.98256057	0.99114898
ENSCGRG00001006454	55.0616792	-6.2267754	-2.6384852	0.46104119	3.48E-10	7.89E-09
ENSCGRG00001006463	295.520052	1.04693741	0.0661752	0.11131302	0.4879364	0.6763109
ENSCGRG00001006467	22400.63	1.01430249	0.02048796	0.04925178	0.68028949	0.8159652
ENSCGRG00001006507	1279.85273	-2.0051974	-1.0037442	0.08072198	1.12E-36	1.48E-34
ENSCGRG00001006519	1.99085635	1.00077743	0.00112116	0.21415161	0.9638734	0.9827954
ENSCGRG00001006524	628.3264	-1.4694864	-0.555312	0.31449646	0.00565434	0.02442902
ENSCGRG00001006529	1732.39487	1.14174968	0.19124639	0.07263054	0.00508575	0.02245726
ENSCGRG00001006543	2.51258528	1.01239849	0.01777726	0.21430704	0.56961036	0.73906967
ENSCGRG00001006558	8.08395121	1.02056043	0.02936161	0.21261529	0.54573244	0.72318588
ENSCGRG00001006573	4.67053122	1.01533859	0.02196091	0.21470468	0.49866378	0.68458718
ENSCGRG00001006596	683.152086	-1.0869174	-0.1202423	0.08619675	0.12565842	0.27464329
ENSCGRG00001006608	655.332504	1.0342998	0.04865443	0.12106108	0.62805568	0.78259076
ENSCGRG00001006626	57.0590849	-1.1287385	-0.1747113	0.25801975	0.12384012	0.27208408
ENSCGRG00001006646	15.6232921	-1.0692916	-0.0966554	0.22926906	0.2088856	0.39419925
ENSCGRG00001006652	636.217767	1.01730506	0.02475236	0.08449293	0.75072875	0.86180082
ENSCGRG00001006653	711.485216	-1.2158336	-0.2819458	0.10683134	0.00287263	0.0140828
ENSCGRG00001006671	60.0738049	-1.1609763	-0.2153385	0.2639308	0.09459727	0.22407378
ENSCGRG00001006673	49.4105254	1.04409575	0.06225403	0.18014466	0.55510841	0.73030078
ENSCGRG00001006690	125.478203	-1.1924032	-0.2538722	0.22143682	0.06501503	0.17042
ENSCGRG00001006725	63.9732121	-1.0641624	-0.0897183	0.19631304	0.39004426	0.59162937
ENSCGRG00001006744	6.32396539	1.00052639	0.00075922	0.21019609	0.98899265	0.99481711
ENSCGRG00001006764	61.040526	-1.1341157	-0.1815678	0.20819351	0.14688223	0.3070433
ENSCGRG00001006775	32.5747283	1.07733582	0.10746803	0.22016982	0.26502883	0.45872441
ENSCGRG00001006777	355.440457	1.0823083	0.11411151	0.14702513	0.30736448	0.50575966
ENSCGRG00001006781	6.97585019	1.0341516	0.0484477	0.21391331	0.41842666	0.61730342
ENSCGRG00001006796	57.9648174	-1.0570593	-0.0800563	0.18945214	0.44688628	0.64302826
ENSCGRG00001006808	2.90811244	-1.0087751	-0.0126046	0.21211885	0.74729463	0.8597076
ENSCGRG00001006835	1499.01987	1.032349	0.04593077	0.21634655	0.36650679	0.56770882
ENSCGRG00001006846	2059.81074	1.08104128	0.11242161	0.07098634	0.09278513	0.22088414
ENSCGRG00001006851	99.7281782	-1.0845684	-0.117121	0.21526368	0.26064325	0.45300143
ENSCGRG00001006857	34025.0247	-1.3015186	-0.3801959	0.07315781	4.67E-08	7.69E-07
ENSCGRG00001006858	310.434053	-1.2774685	-0.3532877	0.14541246	0.0034031	0.01619379
ENSCGRG00001006882	418.39592	-1.020914	-0.0298613	0.1129577	0.75559271	0.86397773
ENSCGRG00001006891	2.99699331	-1.015146	-0.0216872	0.21443971	0.51215992	0.69617663
ENSCGRG00001006908	2.57023831	-1.021278	-0.0303757	0.21710023	0.23950743	0.42922359
ENSCGRG00001006912	408.241273	-1.1811409	-0.2401811	0.12978035	0.02558227	0.08302734
ENSCGRG00001006924	4560.1046	-1.0909333	-0.1255629	0.06061873	0.02981269	0.09326021
ENSCGRG00001006928	5195.42316	1.01996447	0.0285189	0.1307268	0.76825206	0.8713089
ENSCGRG00001006936	1.62623769	1.00200694	0.0028925	0.21380049	0.91365325	0.95529506
ENSCGRG00001006948	937.87914	-1.0642804	-0.0898783	0.09200882	0.27873673	0.47450067
ENSCGRG00001006977	282.721103	1.10874535	0.14892806	0.13028768	0.15917047	0.32540277
ENSCGRG00001006979	2798.65314	-1.2312026	-0.3000682	0.06065621	2.68E-07	3.83E-06
ENSCGRG00001006997	27.0665901	-1.0650119	-0.0908696	0.22648066	0.22589973	0.41443281
ENSCGRG00001007014	10193.9917	-1.1060284	-0.1453884	0.07332521	0.03421778	0.10426492
ENSCGRG00001007015	33.516173	-1.1286506	-0.1745989	0.25523656	0.12698901	0.27646656
ENSCGRG00001007022	35.4865409	-1.0181548	-0.025957	0.20012921	0.73756474	0.85348047
ENSCGRG00001007023	1458.35835	1.02281022	0.03253848	0.08935669	0.68951339	0.82225531

ENSCGRG00001007037	361.999526	1.01504543	0.02154429	0.12421701	0.83322456	0.91063959
ENSCGRG00001007041	2.37308161	-1.0153007	-0.0219071	0.21418615	0.52657119	0.7079527
ENSCGRG00001007051	5.83991043	-1.0092264	-0.0132499	0.21241684	0.72504547	0.84502152
ENSCGRG00001007057	4.48938669	1.02442771	0.03481819	0.21422423	0.45704009	0.651244
ENSCGRG00001007062	255.865381	-1.122075	-0.166169	0.1290709	0.11531839	0.2583019
ENSCGRG00001007078	5.45117893	-1.0133859	-0.0191836	0.21255752	0.6399723	0.79058554
ENSCGRG00001007082	22.243103	-1.2483195	-0.3199872	0.4662596	0.02966954	0.09283508
ENSCGRG00001007087	104.976654	1.26183961	0.33552855	0.20157947	0.01907447	0.06596758
ENSCGRG00001007098	84.6956957	1.16115455	0.21556001	0.19818258	0.0970929	0.22809106
ENSCGRG00001007108	40.4012932	1.2819941	0.35838962	0.3742091	0.03158705	0.09776034
ENSCGRG00001007110	408.384171	-1.4950995	-0.5802415	0.11650416	7.54E-08	1.19E-06
ENSCGRG00001007115	178.341217	1.15605608	0.20921138	0.16547672	0.08628418	0.20949588
ENSCGRG00001007129	38.4490389	-1.09127	-0.1260081	0.23426399	0.19029076	0.36894577
ENSCGRG00001007146	1.73265097	1.02612032	0.0371999	0.21949635	0.08093148	0.19963802
ENSCGRG00001007149	17.2109778	-1.0435144	-0.0614505	0.21810286	0.31311307	0.51207488
ENSCGRG00001007163	2.63874484	-1.00339	-0.0048825	0.21269925	0.88469867	0.93815523
ENSCGRG00001007175	1445.56214	1.45234236	0.53838157	0.08623935	6.14E-11	1.58E-09
ENSCGRG00001007178	9744.48146	-1.0391517	-0.0554063	0.05385657	0.28681578	0.48317186
ENSCGRG00001007189	4.85678432	-1.0033383	-0.0048082	0.21175376	0.9011239	0.94874637
ENSCGRG00001007190	210.741645	-1.239685	-0.3099735	0.1823708	0.02114049	0.07173765
ENSCGRG00001007203	95.6435947	-1.0177715	-0.0254138	0.16960916	0.8076784	0.8943949
ENSCGRG00001007210	4.34642065	1.0023878	0.00344076	0.21204991	0.92937447	0.96438129
ENSCGRG00001007219	15.0472205	-1.181115	-0.2401494	0.49261467	0.00107963	0.00621857
ENSCGRG00001007228	1.72720758	-1.0123083	-0.0176488	0.2159337	0.29792942	0.49571879
ENSCGRG00001007230	8759.42773	-1.0868978	-0.1202163	0.05702781	0.02578085	0.08348147
ENSCGRG00001007240	447.361524	1.31791693	0.39825944	0.10429055	2.84E-05	0.00026232
ENSCGRG00001007247	2.69988036	1.00637927	0.00917411	0.21368466	0.75538788	0.86397488
ENSCGRG00001007248	93.6176818	1.03828024	0.05419589	0.16335449	0.62024452	0.77697362
ENSCGRG00001007310	3828.5836	1.19620533	0.25846505	0.0690727	7.90E-05	0.00066206
ENSCGRG00001007328	4.38157557	-1.0131025	-0.0187802	0.21266273	0.64106466	0.79087655
ENSCGRG00001007331	2437.61731	-1.127033	-0.1725298	0.08558848	0.02755874	0.08762161
ENSCGRG00001007353	16.8839566	-1.0409736	-0.0579335	0.20761661	0.46428654	0.65754677
ENSCGRG00001007397	2.59086091	-1.0014389	-0.0020743	0.21208043	0.95373051	0.97761765
ENSCGRG00001007408	5.61541514	-1.001698	-0.0024476	0.20950957	0.95817958	0.98010487
ENSCGRG00001007412	2.53384447	-1.0108988	-0.0156386	0.21414226	0.60260044	0.76472526
ENSCGRG00001007422	2.15819785	1.02697475	0.03840071	0.21938491	0.11271122	0.25428508
ENSCGRG00001007435	35.6172465	-1.0188227	-0.0269029	0.18045539	0.7866282	0.88053373
ENSCGRG00001007447	7.29489564	-1.0166243	-0.0237866	0.21147352	0.62064767	0.777138
ENSCGRG00001007454	5.41653716	1.07137503	0.09946358	0.2446492	0.04428507	0.12708147
ENSCGRG00001007457	3.35163698	-1.0551743	-0.0774813	0.23262715	0.05943618	0.15891829
ENSCGRG00001007458	172.487729	1.05974792	0.08372114	0.14180484	0.44176242	0.63907133
ENSCGRG00001007472	3544.722	-1.1645423	-0.219763	0.05685379	5.92E-05	0.00051052
ENSCGRG00001007481	65.7835681	-1.0443974	-0.0626708	0.18201971	0.54962332	0.72629201
ENSCGRG00001007482	979.218427	1.16645814	0.22213453	0.0972392	0.01092226	0.04219664
ENSCGRG00001007486	43.1818724	-1.0291818	-0.0414978	0.18734778	0.66761711	0.80795231
ENSCGRG00001007506	12.166297	-1.0205605	-0.0293618	0.2094391	0.61547546	0.77387521
ENSCGRG00001007508	4.99061202	-1.0097808	-0.0140421	0.20923827	0.78592276	0.88018569
ENSCGRG00001007527	19.7553123	1.07094164	0.09887986	0.22225974	0.26346073	0.45668903
ENSCGRG00001007531	41.4852174	-1.0786912	-0.109282	0.20790323	0.29996624	0.49767745
ENSCGRG00001007533	2.65617772	-1.0306105	-0.0434992	0.21892608	0.21826002	0.40491106

ENSCGRG00001007587	30.7853734	-1.0425745	-0.0601504	0.19984136	0.50728312	0.69160247
ENSCGRG00001007593	4.78494164	1.00186879	0.00269358	0.20931405	0.95823798	0.98010487
ENSCGRG00001007602	20547.9852	-1.0562937	-0.0790111	0.06272555	0.19140885	0.37010526
ENSCGRG00001007628	48.5626386	1.09426526	0.1299625	0.21094839	0.24555233	0.43588591
ENSCGRG00001007634	310.696364	-1.0326101	-0.0462956	0.12037492	0.64124095	0.79097704
ENSCGRG00001007637	261.341371	-1.0975855	-0.1343333	0.1808979	0.25250758	0.44388941
ENSCGRG00001007642	4196.61621	-1.065361	-0.0913424	0.05362475	0.07796686	0.19453748
ENSCGRG00001007647	120.992158	-1.4065972	-0.4922092	0.21873993	0.00270925	0.01347784
ENSCGRG00001007669	57.972638	1.05813594	0.08152499	0.18417003	0.45123581	0.64656129
ENSCGRG00001007673	52612.2564	-1.4967838	-0.5818659	0.04212428	3.18E-44	6.57E-42
ENSCGRG00001007675	1013.67688	-1.0763257	-0.1061147	0.09454405	0.21031355	0.39561184
ENSCGRG00001007687	18.0986795	-1.011266	-0.0161626	0.20014086	0.82966207	0.90821835
ENSCGRG00001007705	5.53321398	-1.0121597	-0.0174369	0.2105239	0.71624172	0.84070338
ENSCGRG00001007718	109.72071	1.03336774	0.04735375	0.1546646	0.66438798	0.8056724
ENSCGRG00001007720	475.555902	-1.0737925	-0.1027152	0.14046447	0.34384583	0.54324928
ENSCGRG00001007738	2.08848674	-1.0140254	-0.0200938	0.21442147	0.52155723	0.703771
ENSCGRG00001007741	36.7093976	-1.0701966	-0.0978758	0.20257596	0.34595614	0.54537265
ENSCGRG00001007744	557.376354	1.01948609	0.02784209	0.09364903	0.7419223	0.85621844
ENSCGRG00001007754	3538.62916	-1.0140626	-0.0201468	0.05259287	0.69059884	0.82279048
ENSCGRG00001007762	622.218727	-1.0228213	-0.0325541	0.17878305	0.76016581	0.86711701
ENSCGRG00001007764	32.4341108	-1.0002644	-0.0003814	0.19307987	0.99430899	0.99742843
ENSCGRG00001007777	138.925779	-1.0721714	-0.1005356	0.15580383	0.36970731	0.57107809
ENSCGRG00001007791	11.9744932	-1.0658466	-0.0919999	0.23752605	0.09140675	0.21853685
ENSCGRG00001007799	1185.23973	-1.0246339	-0.0351085	0.07437653	0.61428566	0.77329002
ENSCGRG00001007815	317.83259	-1.1804842	-0.2393787	0.13101589	0.02729659	0.08706904
ENSCGRG00001007842	569.890912	1.32465023	0.40561147	0.10067088	1.16E-05	0.00011917
ENSCGRG00001007854	496.56438	1.02048298	0.02925212	0.09978848	0.7420773	0.85622047
ENSCGRG00001007858	981.107524	-1.0094903	-0.0136271	0.19054651	0.87823556	0.93471042
ENSCGRG00001007863	819.960617	1.02198611	0.03137559	0.10400643	0.73084131	0.849168
ENSCGRG00001007868	16.7413875	-1.0662205	-0.0925058	0.22336307	0.25659072	0.44866601
ENSCGRG00001007877	56.0302724	1.00802372	0.01152959	0.17178808	0.91306892	0.95512109
ENSCGRG00001007881	2.28368751	-1.0051226	-0.0073714	0.21425468	0.75425647	0.86317158
ENSCGRG00001007884	2.45033046	1.00868743	0.01247919	0.21294392	0.72577751	0.84550835
ENSCGRG00001007895	2.06743095	-1.0067662	-0.0097287	0.21398702	0.71805174	0.84155927
ENSCGRG00001007906	2.93562229	-1.014709	-0.0210661	0.21394972	0.54644051	0.72332988
ENSCGRG00001007911	1.92855124	-1.0036955	-0.0053217	0.21432567	0.81163765	0.89730545
ENSCGRG00001007921	13.7683623	1.02780566	0.0395675	0.21237425	0.48886507	0.67701206
ENSCGRG00001007937	252.317109	-1.0671258	-0.0937302	0.22995817	0.19724437	0.37797114
ENSCGRG00001007941	730.503918	-1.1105011	-0.1512108	0.09343633	0.07267569	0.18413282
ENSCGRG00001007951	252.317109	-1.0671258	-0.0937302	0.22995817	0.19724437	0.37797114
ENSCGRG00001007978	767.985308	-1.5826697	-0.6623602	0.12074961	3.94E-09	7.65E-08
ENSCGRG00001007987	2926.08233	-1.1083818	-0.148455	0.27709901	0.05491705	0.14924667
ENSCGRG00001007988	846.834781	1.13623151	0.18425681	0.08262289	0.01545455	0.05581416
ENSCGRG00001007999	601.692066	-1.1734058	-0.230702	0.09726471	0.00822596	0.03325168
ENSCGRG00001008003	1220.69992	-1.0517644	-0.0728116	0.16589506	0.50554514	0.68982033
ENSCGRG00001008014	9142.3648	-1.0780419	-0.1084133	0.06155608	0.06599005	0.17203486
ENSCGRG00001008024	219.384255	1.58322121	0.66286285	0.20246728	8.99E-05	0.00073981
ENSCGRG00001008034	129.68598	-1.0255174	-0.036352	0.14436871	0.73408397	0.85136932
ENSCGRG00001008037	137.055049	-1.0003783	-0.0005457	0.14431648	0.99502957	0.99791191
ENSCGRG00001008041	1565.4889	-1.0479376	-0.0675528	0.07455289	0.33241522	0.53226428

ENSCGRG00001008064	7.03026657	-1.0303872	-0.0431866	0.21336099	0.44809426	0.64385407
ENSCGRG00001008076	6240.38825	-1.109386	-0.1497614	0.13942878	0.17145268	0.34337407
ENSCGRG00001008082	3.81071742	-1.0125222	-0.0179536	0.21183277	0.68230026	0.81710659
ENSCGRG00001008087	1379.6193	-1.0387435	-0.0548395	0.06363564	0.36610423	0.56724635
ENSCGRG00001008089	4.32061423	-1.0109602	-0.0157262	0.21211946	0.70041904	0.82955412
ENSCGRG00001008102	2.39802704	-1.0182779	-0.0261313	0.21532378	0.41351951	0.61232681
ENSCGRG00001008111	29.4383761	-1.03853	-0.0545429	0.19966598	0.53717273	0.71596948
ENSCGRG00001008117	2.4503815	1.02144951	0.03061789	0.21603245	0.36889391	0.57023422
ENSCGRG00001008121	840.843516	-1.6043987	-0.6820327	0.26758609	0.00075765	0.00462607
ENSCGRG00001008135	14.6130338	-1.1216698	-0.165648	0.30290841	0.02676474	0.08582137
ENSCGRG00001008177	6.64569688	1.03644265	0.05164029	0.22018948	0.23541257	0.42527641
ENSCGRG00001008191	18.5340819	-1.0434367	-0.0613431	0.20853013	0.44313223	0.64014609
ENSCGRG00001008220	64.9541642	-1.0593808	-0.0832213	0.20187973	0.39594434	0.59657104
ENSCGRG00001008233	3782.89167	1.00878117	0.01261325	0.18168106	0.89868562	0.94726921
ENSCGRG00001008245	355.241803	-1.1247229	-0.1695696	0.15860288	0.14671428	0.30679235
ENSCGRG00001008249	2.61428825	1.00949356	0.01363171	0.21406757	0.63917388	0.7904701
ENSCGRG00001008258	5.98896937	1.01574369	0.0225364	0.21196559	0.62535235	0.78077618
ENSCGRG00001008263	19.2879956	-1.0984292	-0.1354418	0.25798375	0.09823625	0.22997192
ENSCGRG00001008271	3.45696238	-1.0094238	-0.013532	0.21218238	0.73139431	0.8493483
ENSCGRG00001008296	3.78680378	1.00925816	0.01329524	0.21098037	0.77054777	0.87248798
ENSCGRG00001008303	11.0642734	-1.0026665	-0.0038419	0.20223261	0.95443587	0.97802764
ENSCGRG00001008304	4.21641825	-1.0152012	-0.0217657	0.21207474	0.62760527	0.78248298
ENSCGRG00001008315	3.21114792	1.00208119	0.0029994	0.21157318	0.94097474	0.97138258
ENSCGRG00001008325	189.005246	-1.0186939	-0.0267207	0.15344837	0.80359007	0.89156321
ENSCGRG00001008326	5.17536418	-1.0389664	-0.055149	0.22373466	0.0835331	0.20440477
ENSCGRG00001008330	5.48086576	-1.0013308	-0.0019187	0.21048651	0.96408414	0.9827954
ENSCGRG00001008338	27.3863497	-1.0342216	-0.0485454	0.19338432	0.60296492	0.76484432
ENSCGRG00001008341	25.7888996	-1.0133867	-0.0191847	0.19066059	0.82986491	0.90828516
ENSCGRG00001008347	7.35052202	-1.0088939	-0.0127745	0.21096004	0.77193157	0.87322376
ENSCGRG00001008349	3.81898977	1.01174826	0.01685037	0.21338836	0.63759864	0.7892098
ENSCGRG00001008358	3713.3097	-1.0096017	-0.0137863	0.14980739	0.89552345	0.94539263
ENSCGRG00001008360	4.91155101	-1.007296	-0.0104877	0.21297199	0.75978998	0.86676548
ENSCGRG00001008365	36.3319392	-1.0480984	-0.0677742	0.20114667	0.46119806	0.65499832
ENSCGRG00001008368	246.511532	1.00857293	0.01231541	0.1178542	0.90169966	0.94918528
ENSCGRG00001008370	2.18209027	1.01819474	0.02601351	0.21543415	0.40292219	0.60268955
ENSCGRG00001008371	3.89676128	1.00035032	0.00050531	0.21125702	0.99220882	0.99633103
ENSCGRG00001008381	141.4951	-1.0770246	-0.1070512	0.1975426	0.32641331	0.52642006
ENSCGRG00001008387	9938.09769	-1.2040863	-0.2679389	0.06324161	9.43E-06	9.86E-05
ENSCGRG00001008388	6.85648552	-1.0206906	-0.0295457	0.20960559	0.61060819	0.77085748
ENSCGRG00001008389	76.0787665	1.02547334	0.03628998	0.1826168	0.71766578	0.84149187
ENSCGRG00001008392	2.23683238	1.01590233	0.0227617	0.21525161	0.44443886	0.64099039
ENSCGRG00001008405	692.670317	1.06077495	0.08511861	0.12777175	0.41218964	0.61119913
ENSCGRG00001008416	233.83273	-1.0444952	-0.0628059	0.13824048	0.55523952	0.73032326
ENSCGRG00001008424	2.00151196	1.01331196	0.01907839	0.21527794	0.44497206	0.6413245
ENSCGRG00001008426	63.7121558	-1.0293997	-0.0418032	0.18316508	0.6756958	0.81281465
ENSCGRG00001008429	1231.13242	-1.211496	-0.2767896	0.09061491	0.00084965	0.0051003
ENSCGRG00001008438	38.6352451	-1.475961	-0.5616546	0.48570414	0.01110516	0.04275464
ENSCGRG00001008442	383.064271	-1.0243018	-0.0346409	0.12470305	0.73299777	0.85074762
ENSCGRG00001008443	175.447172	-1.0452089	-0.0637913	0.21259136	0.39292165	0.594043
ENSCGRG00001008456	15.3022517	-1.072428	-0.1008808	0.22683498	0.23248397	0.42177024

ENSCGRG00001008460	6.06170767	-1.0250953	-0.035758	0.21397035	0.45790746	0.65210015
ENSCGRG00001008470	5062.92576	-1.0600072	-0.0840741	0.12488835	0.41033915	0.60944284
ENSCGRG00001008485	3.14738549	-1.0193434	-0.0276401	0.21390853	0.50170616	0.68681131
ENSCGRG00001008493	86.4728795	-1.0830336	-0.1150779	0.20660956	0.28600314	0.48253154
ENSCGRG00001008515	53.1583047	1.01121424	0.01608869	0.1861931	0.866602	0.92795884
ENSCGRG00001008516	28.8307023	-1.0582251	-0.0816466	0.20403229	0.39398557	0.59494933
ENSCGRG00001008524	9.51502919	1.0647877	0.09056581	0.23254839	0.15475046	0.31839084
ENSCGRG00001008545	9.14490165	-1.0004725	-0.0006815	0.20668333	0.98860876	0.99466527
ENSCGRG00001008555	2.07255623	1.01279493	0.01834208	0.21426364	0.55726348	0.73133339
ENSCGRG00001008572	7265.58163	-1.0642011	-0.0897708	0.07123297	0.18031566	0.35601782
ENSCGRG00001008582	32.1958607	1.19698385	0.25940369	0.32782646	0.05766602	0.15519444
ENSCGRG00001008589	608.995125	-1.1604221	-0.2146496	0.1097731	0.02440073	0.08029113
ENSCGRG00001008593	5148.51176	2.01209426	1.00869789	0.06148303	1.21E-61	5.35E-59
ENSCGRG00001008594	129.322147	-1.089317	-0.1234239	0.1981205	0.27528987	0.47069718
ENSCGRG00001008614	6.31136485	-1.0487664	-0.0686933	0.22326671	0.22411906	0.41267287
ENSCGRG00001008617	19.6672154	1.05346336	0.07514014	0.22293982	0.25486435	0.44650059
ENSCGRG00001008644	253.960726	1.00965255	0.0138589	0.14824644	0.89913426	0.94758617
ENSCGRG00001008646	42.0865356	1.06313808	0.08832898	0.19932058	0.38921344	0.59099561
ENSCGRG00001008653	16.410243	-1.0889448	-0.1229308	0.25262915	0.09515527	0.22497951
ENSCGRG00001008676	255.81782	1.03186122	0.04524894	0.13590844	0.66988097	0.80967875
ENSCGRG00001008678	6.18772379	1.06310622	0.08828575	0.23382643	0.12418749	0.27249165
ENSCGRG00001008689	260.311644	-1.0045177	-0.006503	0.11259178	0.94535093	0.97295716
ENSCGRG00001008699	15.8618938	1.03231746	0.0458867	0.20623938	0.54378627	0.72157548
ENSCGRG00001008710	105.134922	-1.0470631	-0.0663484	0.1623873	0.54585124	0.72318588
ENSCGRG00001008713	458.549177	1.16060643	0.21487883	0.1177164	0.032364	0.09965934
ENSCGRG00001008723	94.3551397	-1.1114261	-0.1524121	0.18782484	0.20554871	0.38973933
ENSCGRG00001008734	1584.68778	1.14646901	0.19719736	0.06978017	0.00276569	0.01370033
ENSCGRG00001008738	3.37360436	-1.0363323	-0.0514867	0.22137804	0.17158586	0.3434935
ENSCGRG00001008781	3119.29675	1.09139464	0.12617286	0.06200473	0.03320385	0.1017563
ENSCGRG00001008792	1.87168553	1.00944036	0.01355567	0.21429013	0.62312648	0.77883907
ENSCGRG00001008802	5.57404357	-1.0159444	-0.0228214	0.2119625	0.61596338	0.7742156
ENSCGRG00001008805	2.23699051	1.02905442	0.04131928	0.21890477	0.21057932	0.39591194
ENSCGRG00001008806	2965.97088	-1.9927612	-0.9947688	0.07432185	6.00E-42	1.05E-39
ENSCGRG00001008812	45.7210639	-1.0194706	-0.0278202	0.1917389	0.75642473	0.86469755
ENSCGRG00001008834	1303.6627	-1.0890315	-0.1230457	0.11740206	0.21084586	0.39632215
ENSCGRG00001008839	9.58365877	-1.0319149	-0.045324	0.21192147	0.46920925	0.66135814
ENSCGRG00001008847	2.25602944	1.03607349	0.05112633	0.22395694	0.01223355	0.04626861
ENSCGRG00001008851	7.81355711	1.00668393	0.00961079	0.20768457	0.8664378	0.92793815
ENSCGRG00001008864	497.793331	-1.0649337	-0.0907637	0.18708501	0.40410022	0.60358923
ENSCGRG00001008869	3593.08246	-2.3053765	-1.2050024	0.06389517	1.32E-80	1.06E-77
ENSCGRG00001008890	952.326433	1.01128741	0.01619307	0.17399057	0.87701298	0.9339543
ENSCGRG00001008906	10.3213428	-1.0197402	-0.0282017	0.20720425	0.65917235	0.80239448
ENSCGRG00001008907	44.8193033	-1.1141493	-0.1559426	0.26195957	0.11343914	0.25534272
ENSCGRG00001008932	435.182826	-1.1244443	-0.1692121	0.13826039	0.12394031	0.27212456
ENSCGRG00001008958	3366.47108	-1.0927026	-0.1279009	0.21586481	0.24020857	0.42993254
ENSCGRG00001008965	405.057889	-1.1601286	-0.2142847	0.14185707	0.05840249	0.15657929
ENSCGRG00001008966	447.771002	1.01614541	0.02310686	0.09952141	0.79450694	0.88578189
ENSCGRG00001008971	684.283944	-1.7351335	-0.7950467	0.13529128	3.36E-10	7.64E-09
ENSCGRG00001008975	7133.0422	-1.081716	-0.1133218	0.06019721	0.04915032	0.13738067
ENSCGRG00001008979	27.4457208	-2.2581159	-1.1751195	0.72283711	0.00270528	0.01346336

ENSCGRG00001008980	48.8455204	-1.020155	-0.0287884	0.17953804	0.77349704	0.87386763
ENSCGRG00001008982	13.3042491	-1.0176998	-0.0253121	0.20645962	0.6916178	0.82348698
ENSCGRG00001008990	3.58870061	-1.0328532	-0.0466353	0.21993483	0.19113908	0.36980643
ENSCGRG00001009004	26.9893331	-1.0818042	-0.1134394	0.22186201	0.24826531	0.43923403
ENSCGRG00001009005	331.492668	-1.0205524	-0.0293502	0.13852352	0.78153673	0.87773851
ENSCGRG00001009011	970.802306	-1.0719126	-0.1001872	0.09018323	0.22008905	0.40733182
ENSCGRG00001009022	10.0222803	-1.0419878	-0.0593385	0.21877168	0.30136512	0.49928702
ENSCGRG00001009035	12734.4924	-1.055068	-0.0773359	0.1234743	0.44572462	0.64226461
ENSCGRG00001009055	30.1140373	-1.0288716	-0.0410629	0.18946758	0.66528078	0.80639448
ENSCGRG00001009061	35.1360764	1.00688708	0.0099019	0.19699771	0.90361042	0.94988097
ENSCGRG00001009071	338.25286	1.02451527	0.03494149	0.13181773	0.7420681	0.85622047
ENSCGRG00001009074	2.37764268	1.00605504	0.00870924	0.21392823	0.75355691	0.86298066
ENSCGRG00001009079	13.820024	1.06171795	0.08640056	0.22177378	0.27580495	0.47132622
ENSCGRG00001009088	1143.11378	-1.0132201	-0.0189476	0.16803708	0.85472832	0.92233761
ENSCGRG00001009101	19.9426486	-1.028371	-0.0403608	0.2081551	0.54953977	0.72629135
ENSCGRG00001009111	16.2516091	1.02649908	0.03773233	0.20186797	0.63497104	0.78732309
ENSCGRG00001009123	1401.3826	-1.1020151	-0.140144	0.09612976	0.10277482	0.23815944
ENSCGRG00001009146	10.0281739	-1.0778579	-0.1081669	0.24842133	0.05927893	0.15866342
ENSCGRG00001009149	541.834691	1.07239744	0.10083969	0.09587486	0.2395722	0.42927168
ENSCGRG00001009153	4455.70334	1.05359227	0.07531667	0.12615767	0.46539013	0.65845456
ENSCGRG00001009163	168.836026	-1.1217589	-0.1657627	0.1908619	0.17538033	0.34901469
ENSCGRG00001009195	2.73310312	-1.0302955	-0.0430582	0.21833959	0.26115474	0.45367402
ENSCGRG00001009204	9.56418551	1.04455489	0.06288831	0.22505364	0.13929022	0.29551304
ENSCGRG00001009212	2361.65529	-1.0991488	-0.1363867	0.0704074	0.03954575	0.11660248
ENSCGRG00001009217	290.266574	1.02787816	0.03966926	0.14061634	0.71224193	0.83736812
ENSCGRG00001009224	20174.7244	1.08231813	0.11412462	0.18172718	0.31786812	0.51654074
ENSCGRG00001009231	1922.43002	1.03050952	0.04335783	0.08237213	0.56929619	0.73906967
ENSCGRG00001009242	1.65914894	-1.0043486	-0.0062601	0.21428864	0.78330982	0.87888226
ENSCGRG00001009246	8.30742804	-1.0240101	-0.0342299	0.21005039	0.56371745	0.73559175
ENSCGRG00001009248	398.849572	-1.2829457	-0.3594601	0.18979671	0.01090495	0.04217765
ENSCGRG00001009256	131.555652	-1.2012274	-0.2645093	0.22874208	0.05947892	0.15899937
ENSCGRG00001009268	3.27337159	-1.0334741	-0.0475023	0.22041312	0.17005497	0.34122955
ENSCGRG00001009280	56.9947946	-1.2493407	-0.321167	0.31963777	0.03955928	0.11660248
ENSCGRG00001009285	12.7817497	-1.044787	-0.0632089	0.21862731	0.30811094	0.50627259
ENSCGRG00001009300	1.93663609	-1.0172438	-0.0246654	0.21651459	0.25805757	0.45026721
ENSCGRG00001009308	54.9870061	-2.1532428	-1.106511	0.3515943	8.34E-05	0.00069316
ENSCGRG00001009325	1201.42568	1.01309002	0.01876238	0.11219178	0.84314017	0.91561666
ENSCGRG00001009329	1784.50711	-1.0259383	-0.0369439	0.05788751	0.50664163	0.69116925
ENSCGRG00001009334	2155.97629	-1.2704931	-0.3453886	0.10250335	0.00019573	0.00146777
ENSCGRG00001009341	12.5904974	-1.0150298	-0.021522	0.20830022	0.70589551	0.83348893
ENSCGRG00001009343	71.0488347	1.00870101	0.01249861	0.17518046	0.90395363	0.94995896
ENSCGRG00001009352	12.6236838	1.01048781	0.01505192	0.20226845	0.83406988	0.91104481
ENSCGRG00001009363	1.65090922	-1.0196941	-0.0281364	0.21724171	0.19342434	0.37293285
ENSCGRG00001009365	40.0232256	1.01505747	0.02156141	0.19080415	0.81426409	0.89920026
ENSCGRG00001009373	42.4463377	-1.0306019	-0.0434872	0.19314369	0.63725098	0.78894221
ENSCGRG00001009378	5.28680928	1.00193099	0.00278314	0.21069773	0.95151644	0.9762074
ENSCGRG00001009381	59.4449299	-1.1125776	-0.153906	0.23850512	0.16292803	0.33039544
ENSCGRG00001009385	70.0366103	-1.0679374	-0.0948271	0.19152188	0.38074168	0.58280281
ENSCGRG00001009395	88.3069036	1.07690686	0.10689348	0.17400289	0.34929933	0.54895404
ENSCGRG00001009407	464.56838	-1.021476	-0.0306552	0.11665958	0.7541247	0.86317158

ENSCGRG00001009408	521.702726	1.25670223	0.32964285	0.1070278	0.00058304	0.00371024
ENSCGRG00001009423	33.4919205	-1.0635084	-0.0888315	0.21379243	0.32602707	0.52592957
ENSCGRG00001009434	124.648006	-1.1144595	-0.1563442	0.20254765	0.19191992	0.37059001
ENSCGRG00001009436	3.88488516	-1.0596394	-0.0835733	0.23968499	0.00063999	0.00400304
ENSCGRG00001009474	182.359137	1.0990907	0.13631045	0.16117777	0.23945441	0.42918861
ENSCGRG00001009486	75.101146	1.0134099	0.01921783	0.18437539	0.84363054	0.91583959
ENSCGRG00001009500	33.0331657	-1.0378224	-0.0535596	0.20539568	0.50397362	0.68848268
ENSCGRG00001009505	10.6317718	-1.1247588	-0.1696157	0.30082865	0.04004591	0.11777063
ENSCGRG00001009510	2.00315295	-1.0101868	-0.0146221	0.21449873	0.57767361	0.74410617
ENSCGRG00001009525	4279.54621	1.04451179	0.06282878	0.05962109	0.27160814	0.46714558
ENSCGRG00001009535	2114.70543	1.09130081	0.12604883	0.06994582	0.05613311	0.151875
ENSCGRG00001009537	396.310283	1.00094538	0.00136326	0.13313957	0.99091624	0.99565747
ENSCGRG00001009540	1833.0426	1.01398201	0.02003206	0.0711844	0.76656656	0.87106819
ENSCGRG00001009541	18674.5625	1.01538984	0.02203374	0.08281821	0.76766031	0.87127745
ENSCGRG00001009546	600.808585	1.03210541	0.04559033	0.20892746	0.53156011	0.71171371
ENSCGRG00001009554	79.1932546	1.06796376	0.0948627	0.17030205	0.40109963	0.60084279
ENSCGRG00001009562	6420.3657	1.03528085	0.05002219	0.07132284	0.45675367	0.65105314
ENSCGRG00001009565	2700.21869	1.13838215	0.18698495	0.15346757	0.11015664	0.25032935
ENSCGRG00001009581	7.31528375	-1.009458	-0.0135809	0.20684953	0.81888928	0.90236315
ENSCGRG00001009582	3368.97858	-1.1028219	-0.1411999	0.06663149	0.02528412	0.08233085
ENSCGRG00001009587	1831.31765	-1.0884797	-0.1223145	0.07067498	0.06594272	0.17200697
ENSCGRG00001009606	7.56241647	1.00966629	0.01387855	0.21107757	0.76291829	0.86886409
ENSCGRG00001009609	1498.56993	1.00990673	0.01422206	0.16974267	0.89447166	0.9447757
ENSCGRG00001009619	1306.00808	-1.1015283	-0.1395066	0.08349426	0.06880518	0.17730725
ENSCGRG00001009622	3220.6784	1.05581589	0.07835829	0.05951992	0.170077	0.34122955
ENSCGRG00001009640	1325.4645	1.10915454	0.1494604	0.07142775	0.02601222	0.08400875
ENSCGRG00001009646	57.0499491	-1.1052823	-0.1444149	0.21286201	0.21256734	0.39844711
ENSCGRG00001009651	4286.04334	1.02457541	0.03502618	0.06683279	0.5859366	0.74971062
ENSCGRG00001009670	147.215634	1.10993472	0.15047483	0.18142643	0.20998311	0.39528048
ENSCGRG00001009672	2290.93787	-1.0022708	-0.0032724	0.07063616	0.95951816	0.98103821
ENSCGRG00001009716	539.68981	-1.3028749	-0.3816986	0.12056873	0.00034465	0.00235214
ENSCGRG00001009721	370.691735	-1.0207013	-0.0295607	0.12152924	0.76746746	0.87125648
ENSCGRG00001009730	2645.02513	-1.1145448	-0.1564546	0.07028592	0.01811816	0.0634476
ENSCGRG00001009737	533.811751	-1.1745031	-0.2320505	0.12479924	0.02666379	0.08560481
ENSCGRG00001009755	1545.40225	1.03447252	0.04889532	0.10750351	0.60167694	0.76372192
ENSCGRG00001009760	593.26324	-1.0496627	-0.0699257	0.1247506	0.49275788	0.67961115
ENSCGRG00001009781	81.7313612	1.06703895	0.09361284	0.19430674	0.38049662	0.58257111
ENSCGRG00001009785	28.3887642	-1.0035323	-0.0050871	0.19361612	0.9504884	0.97538704
ENSCGRG00001009824	36.3762102	1.02395439	0.03415145	0.184772	0.72946748	0.84829649
ENSCGRG00001009825	1383.72059	-1.1171334	-0.1598014	0.08119264	0.03290217	0.10107358
ENSCGRG00001009833	16905.9219	-2.0530836	-1.0377923	0.05218174	3.51E-89	3.46E-86
ENSCGRG00001009840	110.983357	-1.1551141	-0.2080353	0.18548067	0.10001888	0.2330378
ENSCGRG00001009851	11.458447	1.0051958	0.00747655	0.20492473	0.90842288	0.95236084
ENSCGRG00001009873	4061.9599	1.0150891	0.02160637	0.06290475	0.73109984	0.8492373
ENSCGRG00001009881	373.575989	-1.040907	-0.0578412	0.12715282	0.57389969	0.74199183
ENSCGRG00001009907	434.232499	-1.0471793	-0.0665085	0.1073043	0.47327333	0.66426478
ENSCGRG00001009918	216.276557	1.18263722	0.24200758	0.18963321	0.06537419	0.1709766
ENSCGRG00001009921	39016.2076	1.00617963	0.00888789	0.05471246	0.82307477	0.90419748
ENSCGRG00001009937	1702.20794	-1.0265415	-0.0377919	0.16411704	0.72322264	0.84393428
ENSCGRG00001009945	91.4055894	-1.0126613	-0.0181517	0.16016738	0.86424165	0.92713638

ENSCGRG00001009957	3215.62055	-1.1410574	-0.1903714	0.07149025	0.00457486	0.02062069
ENSCGRG00001009965	4854.79121	-1.0624585	-0.0874065	0.16446867	0.43744252	0.63497322
ENSCGRG00001009968	9.13856572	-1.0132995	-0.0190606	0.20875143	0.73064976	0.84909947
ENSCGRG00001009974	116.628256	-1.0791782	-0.1099332	0.16397746	0.33295478	0.53281083
ENSCGRG00001009988	2218.99513	1.03772662	0.05342643	0.05722822	0.3326986	0.53260048
ENSCGRG00001009996	42407.7575	1.01392476	0.01995059	0.05511335	0.72155615	0.84335167
ENSCGRG00001010002	2.76297486	-1.0103129	-0.0148022	0.21349659	0.65765189	0.80140161
ENSCGRG00001010009	168.664293	-1.1597125	-0.2137672	0.18134894	0.09096718	0.21785424
ENSCGRG00001010016	404.438853	1.01038801	0.01490942	0.12156277	0.88136159	0.93656093
ENSCGRG00001010021	172.966873	-1.0026126	-0.0037642	0.12647766	0.96966981	0.98567452
ENSCGRG00001010025	2.35983025	1.02582391	0.03678311	0.21690347	0.3152031	0.51423226
ENSCGRG00001010027	59.9642831	-1.2439111	-0.3148834	0.31035279	0.04136666	0.12070773
ENSCGRG00001010033	2280.85947	-1.3040293	-0.3829763	0.06693987	2.51E-09	5.03E-08
ENSCGRG00001010070	6.27248584	-1.0006662	-0.0009608	0.20820261	0.98373713	0.99141261
ENSCGRG00001010071	151.659197	1.10093641	0.13873114	0.16325663	0.23381877	0.42333238
ENSCGRG00001010096	1496.13796	-1.0753172	-0.1047623	0.1111263	0.27114818	0.46641711
ENSCGRG00001010099	140.407249	-1.318059	-0.3984149	0.329349	0.02186633	0.07367376
ENSCGRG00001010109	60.694666	1.02709699	0.03857242	0.19194534	0.6768899	0.81348715
ENSCGRG00001010151	3.15930208	1.0011665	0.00168193	0.21168251	0.96766058	0.98425695
ENSCGRG00001010154	984.388049	-1.2595383	-0.3328949	0.13461462	0.00337794	0.01610996
ENSCGRG00001010157	17.4164964	1.02886989	0.04106054	0.2063388	0.57437988	0.74221064
ENSCGRG00001010164	325.135684	1.21222655	0.27765935	0.19283334	0.04004812	0.11777063
ENSCGRG00001010165	108.561055	-1.0410983	-0.0581064	0.16428167	0.59527858	0.75850205
ENSCGRG00001010169	229.033786	-1.1958592	-0.2580476	0.20103684	0.05614988	0.151875
ENSCGRG00001010179	544.23405	-1.2180677	-0.2845944	0.11113401	0.00354438	0.01676645
ENSCGRG00001010181	8.55240779	1.01941986	0.02774836	0.20940536	0.63030765	0.78413528
ENSCGRG00001010187	2525.05858	-1.1445648	-0.1947992	0.06570025	0.00177695	0.00946081
ENSCGRG00001010190	2159.92641	1.0327095	0.04643448	0.08292373	0.54432865	0.72190205
ENSCGRG00001010209	665.439019	-1.0457873	-0.0645894	0.11834322	0.51296766	0.69679862
ENSCGRG00001010228	6.60675595	1.02623787	0.03736517	0.21315441	0.48065367	0.67100844
ENSCGRG00001010233	28.3489418	-1.2005545	-0.2637009	0.35153734	0.04927161	0.13759958
ENSCGRG00001010239	10107.9846	-1.0196569	-0.0280838	0.04133716	0.48653201	0.67514677
ENSCGRG00001010247	504.481453	1.12620398	0.17146816	0.16449929	0.15063177	0.31236777
ENSCGRG00001010274	19553.8084	-1.0659712	-0.0921684	0.05246127	0.0694911	0.17850031
ENSCGRG00001010285	4.55994871	-1.0000376	-5.42E-05	0.21002692	0.99729751	0.99909135
ENSCGRG00001010305	8.68768653	-1.0279751	-0.0398053	0.21132592	0.50329934	0.68800176
ENSCGRG00001010314	6550.12167	-1.0358912	-0.0508725	0.07412709	0.46398489	0.65728516
ENSCGRG00001010322	63.0411817	-1.2233673	-0.2908576	0.24292971	0.04656181	0.13195945
ENSCGRG00001010332	1795.51995	-1.1548561	-0.2077131	0.05638633	0.00012907	0.00101857
ENSCGRG00001010351	604.318834	1.30119665	0.37983901	0.10503029	6.70E-05	0.00057073
ENSCGRG00001010358	824.684379	-1.0185406	-0.0265035	0.07959276	0.71930575	0.8417967
ENSCGRG00001010369	60.1900212	-1.1103512	-0.151016	0.22051227	0.19046215	0.3691663
ENSCGRG00001010370	50.2714065	-1.0107372	-0.0154079	0.18504154	0.86968819	0.92939899
ENSCGRG00001010371	306.994975	1.09882749	0.13596491	0.13923737	0.21502695	0.40117903
ENSCGRG00001010382	4064.28838	-1.0880229	-0.1217089	0.13662727	0.25872889	0.45092749
ENSCGRG00001010393	160.130447	-1.0677688	-0.0945993	0.18084092	0.39505478	0.59587014
ENSCGRG00001010395	1475.69111	-2.0493867	-1.0351922	0.1130479	3.68E-21	2.36E-19
ENSCGRG00001010398	146.555164	-1.1478399	-0.1989214	0.16643385	0.10054217	0.2340442
ENSCGRG00001010403	4.88344375	-1.0003071	-0.0004429	0.21044524	0.99021056	0.9954343
ENSCGRG00001010454	135.191292	-1.6119407	-0.6887987	0.19832118	4.22E-05	0.00037592

ENSCGRG00001010457	4.04337434	-1.0875171	-0.1210381	0.26879673	0.00015888	0.00122094
ENSCGRG00001010474	65.7193624	-1.0698142	-0.0973603	0.17744151	0.38574512	0.58691158
ENSCGRG00001010476	9214.53119	-1.0168195	-0.0240636	0.18427448	0.7496151	0.8610022
ENSCGRG00001010501	2011.62479	1.04798164	0.06761344	0.07685503	0.34585363	0.54527815
ENSCGRG00001010509	8.5967714	-1.0595167	-0.0834064	0.22650443	0.21475221	0.40095844
ENSCGRG00001010523	1831.91773	1.22436898	0.2920384	0.08442581	0.00019185	0.00144398
ENSCGRG00001010536	104.720843	1.1352811	0.18304956	0.18757152	0.1421064	0.30009611
ENSCGRG00001010545	18.0218971	-1.0222556	-0.031756	0.2041524	0.65995231	0.80307677
ENSCGRG00001010550	4869.28247	1.02451665	0.03494344	0.17098739	0.7579045	0.86561668
ENSCGRG00001010560	2686.27103	-1.028051	-0.0399118	0.1531169	0.71033492	0.83664861
ENSCGRG00001010563	103.830332	1.16784241	0.22384561	0.21429755	0.09207297	0.21963775
ENSCGRG00001010566	976.320678	1.0135667	0.01944103	0.10016522	0.82778951	0.90766699
ENSCGRG00001010572	60.7148752	-1.0894951	-0.1236598	0.18598924	0.28477464	0.48145217
ENSCGRG00001010579	146.534919	1.08752275	0.12104558	0.18767313	0.29538012	0.49287733
ENSCGRG00001010597	1907.33722	1.0586614	0.08224124	0.09966886	0.35249072	0.55220816
ENSCGRG00001010602	230.380696	1.03140125	0.0446057	0.11865421	0.65406489	0.79971091
ENSCGRG00001010645	52.5805904	-1.3118379	-0.3915894	0.35957758	0.02425997	0.07997174
ENSCGRG00001010653	9149.80966	-1.0226789	-0.0323532	0.04533162	0.46474183	0.65790063
ENSCGRG00001010672	248.437365	1.01162778	0.01667856	0.12132602	0.86882596	0.9291752
ENSCGRG00001010677	9600.85134	1.37308424	0.45742013	0.05729282	2.81E-16	1.26E-14
ENSCGRG00001010711	36351.4255	-1.1829526	-0.2423923	0.06027233	2.64E-05	0.00024481
ENSCGRG00001010718	1396.12045	-1.0225244	-0.0321353	0.07970638	0.66261973	0.80471736
ENSCGRG00001010723	981.604555	-1.2502885	-0.3222611	0.07669279	8.18E-06	8.63E-05
ENSCGRG00001010729	11.0856346	1.00802251	0.01152785	0.21064423	0.80319456	0.89146475
ENSCGRG00001010734	13.9349733	-1.042305	-0.0597776	0.21575826	0.36085283	0.5610541
ENSCGRG00001010735	11682.4763	-1.0796224	-0.1105268	0.07196732	0.09664905	0.22750355
ENSCGRG00001010756	3811.03102	1.01481146	0.02121171	0.05311278	0.6807687	0.81638251
ENSCGRG00001010765	624.381913	1.10158927	0.13958641	0.09126924	0.09119309	0.21822967
ENSCGRG00001010768	22.1121561	1.03838396	0.05434001	0.20752836	0.48724333	0.67564268
ENSCGRG00001010774	121.496803	-1.0008371	-0.0012072	0.20623143	0.98367394	0.99141261
ENSCGRG00001010780	100.681118	-1.1911742	-0.2523844	0.22538666	0.06778557	0.17542083
ENSCGRG00001010787	185.398345	-1.7608058	-0.8162358	0.19005609	1.34E-06	1.68E-05
ENSCGRG00001010803	2942.91233	-1.0591642	-0.0829262	0.05646547	0.12728715	0.27706855
ENSCGRG00001010807	170.440699	-1.2894173	-0.3667193	0.20865532	0.01323589	0.04914544
ENSCGRG00001010818	4551.37583	-1.1723265	-0.2293744	0.06116061	8.83E-05	0.0007285
ENSCGRG00001010828	223.213384	-1.0561523	-0.0788178	0.15467825	0.47597602	0.66673076
ENSCGRG00001010832	9334.47368	-1.1762491	-0.2341937	0.1549608	0.04983494	0.13871916
ENSCGRG00001010834	174.181602	-1.1133333	-0.1548855	0.16876179	0.19042043	0.3691413
ENSCGRG00001010835	114.447408	-1.1103024	-0.1509527	0.20587664	0.20405601	0.38776999
ENSCGRG00001010836	4.32714077	1.01669175	0.02388234	0.21350648	0.5550388	0.73030078
ENSCGRG00001010839	38.6866783	-1.0188863	-0.026993	0.18365997	0.78117254	0.87751158
ENSCGRG00001010840	9512.22032	-1.0134842	-0.0193236	0.14134788	0.85781467	0.92418115
ENSCGRG00001010848	1528.73293	-1.0253456	-0.0361103	0.06942429	0.5823801	0.74804864
ENSCGRG00001010858	6.94018437	1.06138989	0.08595471	0.23665655	0.05996826	0.15987376
ENSCGRG00001010867	92.0622962	-1.3747933	-0.4592147	0.22191627	0.00460374	0.02072167
ENSCGRG00001010885	31080.9933	-1.2384608	-0.3085482	0.07544398	1.81E-05	0.00017606
ENSCGRG00001010889	611.300596	-1.2198053	-0.2866509	0.13399657	0.01004842	0.0394122
ENSCGRG00001010901	12494.6604	-1.079915	-0.1109177	0.05052853	0.02416821	0.07975137
ENSCGRG00001010907	286.740124	-1.3444896	-0.4270586	0.1979629	0.00441869	0.02002245
ENSCGRG00001010922	589.489934	-1.0168814	-0.0241514	0.10633899	0.79316151	0.88497509

ENSCGRG00001010929	1374.31215	1.19230613	0.25375471	0.09621637	0.00345563	0.01641328
ENSCGRG00001010939	2.18719065	1.00154048	0.00222073	0.21335986	0.94216123	0.97189769
ENSCGRG00001010950	385.036265	-1.1997284	-0.2627078	0.1313512	0.01627344	0.05806761
ENSCGRG00001010968	6280.79936	-1.046965	-0.0662132	0.17080722	0.54416299	0.72190205
ENSCGRG00001011002	30.6778266	1.02795359	0.03977514	0.19849407	0.64171249	0.79125392
ENSCGRG00001011009	1.82114226	-1.0129439	-0.0185542	0.21447373	0.54040236	0.71870372
ENSCGRG00001011016	262.843924	-1.109388	-0.149764	0.13180442	0.15973826	0.32578366
ENSCGRG00001011021	190.446382	-1.3431523	-0.4256229	0.15591827	0.00104638	0.0060515
ENSCGRG00001011022	3.01234934	-1.029956	-0.0425828	0.22044157	0.0995859	0.23219791
ENSCGRG00001011027	4.84678546	1.00949912	0.01363965	0.21214788	0.73708247	0.85316946
ENSCGRG00001011030	15.7704572	-2.5712055	-1.3624449	0.87727281	0.00282195	0.01391425
ENSCGRG00001011038	3184.80118	1.03141206	0.04462081	0.09706719	0.60434756	0.76574602
ENSCGRG00001011042	455.211987	-1.0194077	-0.0277311	0.10689621	0.76424509	0.86952478
ENSCGRG00001011048	279.049294	1.0312972	0.04446014	0.13054059	0.6709313	0.81004995
ENSCGRG00001011053	10.3269224	1.00670482	0.00964073	0.20786313	0.8630315	0.92665838
ENSCGRG00001011066	219.351861	1.01849747	0.0264424	0.11774861	0.7912446	0.88345188
ENSCGRG00001011075	291.952662	-1.0109397	-0.0156969	0.1350778	0.86591646	0.92774752
ENSCGRG00001011078	385.761791	-1.0552202	-0.0775441	0.09971192	0.38089189	0.58280281
ENSCGRG00001011084	317.385445	-1.2186467	-0.2852799	0.11404294	0.00413309	0.01898346
ENSCGRG00001011087	150.783439	1.15434378	0.20707294	0.15830085	0.08231484	0.20204121
ENSCGRG00001011091	1.8774471	1.00500454	0.00720202	0.21312316	0.82585736	0.90637704
ENSCGRG00001011102	2386.72449	1.09722395	0.13385802	0.05511994	0.01175028	0.04487808
ENSCGRG00001011105	4.43806597	1.00854066	0.01226924	0.21249963	0.7451532	0.8582271
ENSCGRG00001011109	413.173481	-1.042357	-0.0598495	0.09917534	0.4955749	0.6819545
ENSCGRG00001011137	6.01025054	1.02940099	0.04180508	0.21346447	0.44967396	0.64526979
ENSCGRG00001011139	7612.09715	-1.0853525	-0.1181637	0.06395254	0.05258252	0.14435964
ENSCGRG00001011145	41.1470774	-1.0332812	-0.0472329	0.20160781	0.56815789	0.73836894
ENSCGRG00001011152	1.57561127	-1.0270005	-0.0384368	0.2200865	0.04038467	0.11854437
ENSCGRG00001011159	4.55801262	-1.0125078	-0.017933	0.21039086	0.71644303	0.84081334
ENSCGRG00001011162	259.949378	-1.1526999	-0.2050169	0.15888668	0.08549198	0.20800612
ENSCGRG00001011185	825.233008	-1.1071792	-0.1468888	0.08921929	0.06948785	0.17850031
ENSCGRG00001011191	170.87731	1.17556068	0.23334901	0.1739955	0.06414584	0.16862471
ENSCGRG00001011199	1501.97071	-1.1945154	-0.2564255	0.09134678	0.00205846	0.01066701
ENSCGRG00001011231	19.4932655	-1.0357998	-0.0507452	0.20938331	0.48143297	0.67165597
ENSCGRG00001011246	2051.54651	1.00395531	0.00569505	0.07665838	0.93735599	0.96972611
ENSCGRG00001011251	185.455738	-1.188077	-0.2486283	0.14529383	0.03176174	0.09817989
ENSCGRG00001011288	120.667989	-1.0673638	-0.094052	0.15391526	0.39803002	0.5982359
ENSCGRG00001011310	5210.49741	-1.0341822	-0.0484904	0.06312264	0.4205607	0.61909705
ENSCGRG00001011314	249.964853	-1.2180168	-0.2845341	0.4039453	0.03721124	0.11119103
ENSCGRG00001011322	531.608528	1.07422836	0.10330071	0.1058086	0.26327335	0.45648231
ENSCGRG00001011323	23.6756712	-1.121489	-0.1654155	0.28604228	0.0655642	0.17133361
ENSCGRG00001011331	57.5249484	1.0636386	0.08900804	0.19975534	0.38490603	0.58600502
ENSCGRG00001011334	1.57118306	-1.025387	-0.0361686	0.21907418	0.08693598	0.21083868
ENSCGRG00001011336	4.76301	1.01654061	0.02366785	0.21176489	0.61695558	0.77467173
ENSCGRG00001011343	4.42081718	1.00174395	0.0025138	0.2111176	0.95382342	0.97763466
ENSCGRG00001011367	54.1783565	-1.0856377	-0.1185427	0.22163415	0.24263046	0.43258124
ENSCGRG00001011370	100.408348	1.03594217	0.05094347	0.16755765	0.63911949	0.7904701
ENSCGRG00001011396	149.772144	-1.0610239	-0.0854572	0.16837967	0.44189257	0.63918743
ENSCGRG00001011405	1179.89642	-1.0384743	-0.0544655	0.19580261	0.55401937	0.72976742
ENSCGRG00001011413	94.1993497	1.0587651	0.08238254	0.17892949	0.45618459	0.6503867

ENSCGRG00001011428	152.018468	1.01989254	0.02841715	0.15864059	0.79373966	0.88527965
ENSCGRG00001011435	5082.39461	1.12790567	0.17364642	0.04386998	4.71E-05	0.00041399
ENSCGRG00001011436	188.405515	-1.0890036	-0.1230088	0.16978487	0.28564454	0.48209573
ENSCGRG00001011448	47.5436771	-1.1421552	-0.1917587	0.2711627	0.10223526	0.23703778
ENSCGRG00001011455	7897.93969	-1.149349	-0.2008169	0.15572835	0.08795949	0.21287759
ENSCGRG00001011461	292.737208	-1.0147616	-0.0211409	0.12840813	0.83613346	0.91195143
ENSCGRG00001011473	5.82503785	1.00657242	0.00945097	0.20975429	0.8466899	0.91760555
ENSCGRG00001011475	354.789944	1.00643226	0.00925007	0.11089676	0.92349209	0.96109284
ENSCGRG00001011481	160.046383	-1.0425573	-0.0601267	0.14551386	0.57834188	0.74472854
ENSCGRG00001011527	32.6463054	-1.0686384	-0.0957737	0.20215687	0.35277229	0.55258169
ENSCGRG00001011533	42.786345	-1.0051733	-0.0074442	0.21344378	0.7994301	0.88879531
ENSCGRG00001011538	15247.1502	-1.0264942	-0.0377255	0.04702001	0.43536101	0.63295591
ENSCGRG00001011539	4600.96729	1.07241078	0.10085762	0.05943579	0.07649494	0.19168626
ENSCGRG00001011540	6.709681	1.02485727	0.035423	0.2127995	0.50164893	0.68681131
ENSCGRG00001011559	454.828101	1.01173568	0.01683243	0.10958687	0.85926117	0.92465857
ENSCGRG00001011560	12.0479101	-1.0739276	-0.1028968	0.23555527	0.16444654	0.33263227
ENSCGRG00001011568	2873.08841	-1.0214614	-0.0306347	0.05788503	0.58176399	0.74773936
ENSCGRG00001011581	58.7492576	1.04568603	0.06444974	0.19809238	0.49766452	0.68402173
ENSCGRG00001011590	207.111645	-1.0191134	-0.0273145	0.14712149	0.79872675	0.88851089
ENSCGRG00001011604	30.7033495	1.00487406	0.00701469	0.19516224	0.93451794	0.96830662
ENSCGRG00001011608	128.058966	-1.037562	-0.0531976	0.17405372	0.61598793	0.7742156
ENSCGRG00001011609	568.460142	-1.4415709	-0.5276418	0.14850234	4.57E-05	0.00040465
ENSCGRG00001011613	515.902351	-1.0157196	-0.0225021	0.09829566	0.79610145	0.88655652
ENSCGRG00001011619	39.7953215	-1.1446278	-0.1948786	0.298898	0.0731251	0.18501531
ENSCGRG00001011667	5082.37384	1.13182904	0.17865606	0.15820897	0.12939453	0.28013248
ENSCGRG00001011703	18.6912609	-1.0349689	-0.0495874	0.21093924	0.46117801	0.65499832
ENSCGRG00001011707	15.557213	1.02600811	0.03704214	0.20620257	0.60264649	0.76472526
ENSCGRG00001011749	121.418185	-1.0576943	-0.0809228	0.1758277	0.46031824	0.65424127
ENSCGRG00001011760	80.8219693	1.267823	0.34235335	0.23698199	0.0256562	0.08320404
ENSCGRG00001011762	83.2325798	-1.0726823	-0.1012229	0.17923724	0.36791528	0.5695462
ENSCGRG00001011776	4.6961932	-1.0138339	-0.0198213	0.21273323	0.6278999	0.78258394
ENSCGRG00001011794	69.2804029	1.15711464	0.2105318	0.22763686	0.10711313	0.24502127
ENSCGRG00001011824	1315.46751	-1.1420253	-0.1915946	0.0744979	0.00597031	0.02546633
ENSCGRG00001011827	3023.37233	-1.3369398	-0.4189345	0.08129483	5.21E-08	8.49E-07
ENSCGRG00001011831	2750.45622	-1.0519797	-0.0731068	0.14245418	0.49706589	0.68356566
ENSCGRG00001011836	2.31988761	1.0133339	0.01910964	0.21488019	0.48698064	0.67549778
ENSCGRG00001011843	5.5125526	-1.0351602	-0.049854	0.21816322	0.30027733	0.49806456
ENSCGRG00001011859	696.744142	1.13500395	0.18269731	0.0906074	0.0261303	0.08427218
ENSCGRG00001011860	6.49194715	-1.0193682	-0.0276753	0.21251362	0.5543285	0.72987441
ENSCGRG00001011861	11806.0893	-1.033426	-0.047435	0.05768431	0.39660679	0.59707756
ENSCGRG00001011871	52.7802416	-1.2999632	-0.3784708	0.30649569	0.02457635	0.08076528
ENSCGRG00001011883	357.076353	-1.1829525	-0.2423921	0.1340313	0.02750004	0.08750014
ENSCGRG00001011892	146.015124	1.02228002	0.03179043	0.15871388	0.77081141	0.87265525
ENSCGRG00001011895	255.22447	-1.1074505	-0.1472422	0.12190627	0.14753306	0.30800171
ENSCGRG00001011925	84.5845807	-1.0157063	-0.0224833	0.15591753	0.8339263	0.91104481
ENSCGRG00001011948	16.5067588	-1.0197604	-0.0282302	0.19873433	0.72509575	0.84502152
ENSCGRG00001011961	92.8512353	1.12734835	0.17293337	0.21706279	0.15915253	0.32540277
ENSCGRG00001012002	71.6263869	1.04761459	0.06710806	0.18628774	0.52028594	0.70301625
ENSCGRG00001012006	165.914268	1.60584904	0.68333628	0.20632693	8.08E-05	0.00067374
ENSCGRG00001012009	137.488274	1.096162	0.13246102	0.17147428	0.26003875	0.45216457

ENSCGRG00001012021	1829.43353	-1.3139635	-0.3939252	0.08470577	7.18E-07	9.52E-06
ENSCGRG00001012028	358.05023	-1.0672713	-0.0939269	0.09960872	0.28661494	0.48309703
ENSCGRG00001012030	203.534097	-1.0543963	-0.0764172	0.13555025	0.47089606	0.66273111
ENSCGRG00001012031	224.512035	1.17030152	0.22688027	0.16156428	0.06229241	0.16466484
ENSCGRG00001012046	1973.30368	-1.984346	-0.9886636	0.06851734	2.54E-48	6.50E-46
ENSCGRG00001012052	61.2891558	-1.0193391	-0.027634	0.18209338	0.77883512	0.87673482
ENSCGRG00001012053	2525.02326	1.01570847	0.02248638	0.08416708	0.77399831	0.87400421
ENSCGRG00001012068	702.939365	-1.0031333	-0.0045133	0.10371668	0.95938969	0.98098514
ENSCGRG00001012084	42.9374856	1.10089856	0.13868154	0.21428136	0.22273758	0.41111463
ENSCGRG00001012091	459.670882	1.0317426	0.04508309	0.12170461	0.65510021	0.80013669
ENSCGRG00001012092	3151.52764	-1.1635305	-0.218509	0.14766306	0.05912273	0.1583446
ENSCGRG00001012104	11.9311162	-1.0990084	-0.1362024	0.27075092	0.04093716	0.11978187
ENSCGRG00001012111	10.1901359	-1.0505862	-0.0711946	0.2209314	0.27580125	0.47132622
ENSCGRG00001012121	2.23148443	-1.0069924	-0.0100528	0.2133656	0.74988592	0.86114196
ENSCGRG00001012123	859.443568	-1.2584102	-0.3316023	0.10327404	0.00036921	0.00249318
ENSCGRG00001012128	38.0865262	1.05108497	0.0718793	0.19195737	0.4801054	0.67068159
ENSCGRG00001012135	30628.9974	-1.2136534	-0.2793565	0.04943179	8.42E-09	1.56E-07
ENSCGRG00001012137	787.781931	-1.2382525	-0.3083056	0.10369474	0.00092079	0.00545068
ENSCGRG00001012140	3588.40715	1.02780272	0.03956338	0.05321563	0.443155	0.64014609
ENSCGRG00001012154	861.344681	1.09345319	0.12889145	0.09354283	0.12679999	0.27618242
ENSCGRG00001012172	2286.92348	1.06842975	0.09549206	0.06949437	0.14612373	0.30585702
ENSCGRG00001012173	426.954414	1.31601655	0.39617763	0.12313768	0.00026156	0.00186664
ENSCGRG00001012183	11.1588572	-1.0135894	-0.0194734	0.20995605	0.70359884	0.83154361
ENSCGRG00001012190	41.0604428	1.00240017	0.00345856	0.19201174	0.97031309	0.98621654
ENSCGRG00001012199	9.78382062	1.01806816	0.02583415	0.20975969	0.6439106	0.79261499
ENSCGRG00001012213	214.203812	1.10199606	0.14011907	0.13348477	0.19342098	0.37293285
ENSCGRG00001012241	250.176216	1.15916316	0.21308364	0.13284696	0.05064791	0.14034171
ENSCGRG00001012264	5157.79469	1.0366475	0.05192541	0.07177951	0.44292845	0.64003536
ENSCGRG00001012277	131.769968	-1.2888803	-0.3661182	0.20150401	0.01205755	0.04569739
ENSCGRG00001012279	2.89793825	1.01759745	0.02516696	0.21472696	0.47306089	0.66421861
ENSCGRG00001012283	10.0382615	-1.0067706	-0.0097349	0.20619248	0.86944628	0.92935896
ENSCGRG00001012307	1118.9337	-1.0144337	-0.0206745	0.10269568	0.81195786	0.89735937
ENSCGRG00001012330	12.2127537	-1.0258075	-0.03676	0.20569447	0.60130584	0.76340352
ENSCGRG00001012360	67.7215975	-1.1347241	-0.1823415	0.27702198	0.09620614	0.22666924
ENSCGRG00001012361	1.96984748	-1.0005594	-0.0008068	0.21289959	0.9791097	0.99034912
ENSCGRG00001012386	150.901922	-1.0905065	-0.1249984	0.1384161	0.25000984	0.44137625
ENSCGRG00001012406	5.03974429	-1.0038261	-0.0055094	0.21074707	0.89703253	0.94607169
ENSCGRG00001012409	1.65962166	1.00120099	0.00173163	0.21372798	0.94992299	0.97504115
ENSCGRG00001012410	1250.87007	-1.012151	-0.0174245	0.08520669	0.8232867	0.90419748
ENSCGRG00001012411	5.83629357	1.00504838	0.00726495	0.21076909	0.87151117	0.93041135
ENSCGRG00001012414	10.9718027	1.01392488	0.01995077	0.20548921	0.76223577	0.8685501
ENSCGRG00001012415	60.6023786	1.03189672	0.04529858	0.17636981	0.66625697	0.80661107
ENSCGRG00001012418	1227.22249	-1.028331	-0.0403047	0.08911274	0.61834834	0.77566023
ENSCGRG00001012442	5.01322463	1.01934795	0.02764659	0.21444638	0.47309373	0.66421861
ENSCGRG00001012449	1941.5911	1.02328336	0.0332057	0.07168713	0.62403541	0.77966386
ENSCGRG00001012453	102.555612	-1.0677766	-0.0946098	0.17752837	0.39686846	0.59720329
ENSCGRG00001012459	18.3474815	-1.0693057	-0.0966744	0.22062571	0.27229056	0.46806791
ENSCGRG00001012467	431.043041	-1.2312749	-0.3001529	0.12384607	0.00465791	0.02091408
ENSCGRG00001012483	29.8513499	1.02041857	0.02916106	0.19475308	0.73650269	0.8528066
ENSCGRG00001012489	4.4646392	1.00093781	0.00135234	0.2105514	0.97778137	0.98983462

ENSCGRG00001012494	600.426711	-1.0305759	-0.0434507	0.08387276	0.57315901	0.74163302
ENSCGRG00001012500	51.8300187	1.00469945	0.006764	0.17400565	0.94897652	0.97462988
ENSCGRG00001012514	4.04989945	-1.0024633	-0.0035495	0.21233291	0.91952775	0.95851172
ENSCGRG00001012534	2369.68154	-1.0401921	-0.05685	0.08914787	0.48235458	0.67195641
ENSCGRG00001012540	32.7028875	-1.1513713	-0.2033531	0.27870048	0.09196995	0.21943286
ENSCGRG00001012584	199.626839	-1.0344274	-0.0488323	0.13238166	0.6399214	0.79058554
ENSCGRG00001012586	6597.17583	-1.1342407	-0.1817268	0.06741343	0.00436537	0.01981588
ENSCGRG00001012601	5.46080749	1.00276229	0.00397965	0.20827341	0.94179117	0.97186658
ENSCGRG00001012604	30.2203007	-1.066442	-0.0928056	0.21202254	0.32623506	0.52619882
ENSCGRG00001012607	8.69470203	-1.0373435	-0.0528937	0.21441158	0.39834021	0.59840561
ENSCGRG00001012624	3.74553127	-1.0083712	-0.0120268	0.21220343	0.75902698	0.86645989
ENSCGRG00001012638	4.91360566	1.01944867	0.02778914	0.21436516	0.47261584	0.66401222
ENSCGRG00001012647	1.66355989	-1.0291792	-0.0414942	0.2200499	0.11556398	0.25871628
ENSCGRG00001012656	6793.34999	-1.1280885	-0.1738802	0.08003896	0.01863018	0.06473111
ENSCGRG00001012657	2165.68694	-1.5699959	-0.6507608	0.1147476	1.46E-09	2.99E-08
ENSCGRG00001012665	67.1835837	-1.114158	-0.1559539	0.19254157	0.19724723	0.37797114
ENSCGRG00001012666	83.7548049	1.2663457	0.3406713	0.29541987	0.0330144	0.10127263
ENSCGRG00001012669	272.277781	1.14844021	0.19967575	0.14688066	0.08174325	0.2011779
ENSCGRG00001012673	5538.64362	-1.1549091	-0.2077793	0.05439427	7.54E-05	0.00063469
ENSCGRG00001012681	23.4640456	-1.15555	-0.2085796	0.35013542	0.03188077	0.0984229
ENSCGRG00001012691	3635.33993	1.04886843	0.06883372	0.07391944	0.32131423	0.52022691
ENSCGRG00001012694	2331.57112	-1.0685121	-0.0956033	0.07876151	0.19062493	0.36942592
ENSCGRG00001012695	2.29349155	-1.0200792	-0.0286812	0.21615359	0.34702179	0.54664894
ENSCGRG00001012714	180.042264	-1.0240692	-0.0343132	0.12195076	0.73238849	0.85034864
ENSCGRG00001012720	217.841107	1.04343181	0.06133632	0.12249113	0.5443288	0.72190205
ENSCGRG00001012728	6.27977121	-1.0263161	-0.0374751	0.212873	0.48089441	0.67119823
ENSCGRG00001012736	57.3786414	1.06499633	0.09084847	0.19907497	0.38007134	0.58210352
ENSCGRG00001012741	12.1742151	-1.0244095	-0.0347925	0.20790587	0.59577805	0.75879524
ENSCGRG00001012747	287.120486	-1.023107	-0.0329571	0.14357537	0.757681	0.86561668
ENSCGRG00001012755	551.365526	-1.1680109	-0.2240538	0.1084653	0.01823177	0.06368937
ENSCGRG00001012771	171.095399	1.11622481	0.15862762	0.18952711	0.19093162	0.36962885
ENSCGRG00001012775	15.340302	-1.1044579	-0.1433385	0.26212955	0.09862561	0.23067264
ENSCGRG00001012782	485.456222	-1.1127095	-0.154077	0.21602495	0.19187227	0.37055386
ENSCGRG00001012785	1.83387584	-1.0341707	-0.0484744	0.22183004	0.08704542	0.21101845
ENSCGRG00001012803	106.348215	1.01815485	0.025957	0.14950853	0.80937369	0.89580758
ENSCGRG00001012806	76.3804504	-1.2662292	-0.3405385	0.28411549	0.03178013	0.09819185
ENSCGRG00001012817	65.5977316	-1.0521521	-0.0733433	0.19237068	0.47082554	0.66270466
ENSCGRG00001012819	3815.97352	-1.0186907	-0.0267161	0.07480832	0.70097161	0.82981668
ENSCGRG00001012838	1.88753805	-1.0167506	-0.0239658	0.21725422	0.07615552	0.19098516
ENSCGRG00001012841	686.291388	-1.0065584	-0.0094309	0.09704437	0.91219379	0.95459541
ENSCGRG00001012849	1160.39848	-1.0317482	-0.0450909	0.07861578	0.53698744	0.71579699
ENSCGRG00001012853	3.03275837	-1.0089749	-0.0128903	0.21273059	0.72228987	0.84343968
ENSCGRG00001012854	6280.70476	-1.1985615	-0.261304	0.07458258	0.00019238	0.00144625
ENSCGRG00001012855	1352.77442	-1.1769752	-0.2350839	0.0870403	0.00321001	0.01544133
ENSCGRG00001012856	85.9710481	-1.0119819	-0.0171835	0.16594034	0.86940955	0.92935896
ENSCGRG00001012857	419.622434	-1.5716615	-0.6522905	0.11021826	3.34E-10	7.63E-09
ENSCGRG00001012868	133.319821	1.12647315	0.17181293	0.22260328	0.16036428	0.32654052
ENSCGRG00001012869	6651.24339	-1.2107176	-0.2758624	0.08326732	0.00033636	0.00230047
ENSCGRG00001012870	3512.84859	-1.1213046	-0.1651782	0.08253712	0.02964145	0.09276986
ENSCGRG00001012875	223.206662	-1.0533752	-0.0750193	0.1328916	0.47654905	0.66710189

ENSCGRG00001012890	62.1327348	1.0145162	0.0207919	0.17961369	0.83601482	0.91195011
ENSCGRG00001012894	5152.57963	-1.0241833	-0.0344739	0.06669808	0.58175184	0.74773936
ENSCGRG00001012898	295.184856	1.21191547	0.27728908	0.12127	0.00772162	0.03149123
ENSCGRG00001012903	2.08052749	-1.0128683	-0.0184466	0.21481757	0.50392158	0.68848268
ENSCGRG00001012904	7.2675203	1.00138744	0.00200026	0.20781339	0.97270254	0.98767978
ENSCGRG00001012919	1371.31129	-1.059658	-0.0835987	0.06762926	0.19180383	0.37051699
ENSCGRG00001012940	1255889.22	1.07105545	0.09903317	0.2310287	0.28100564	0.47686108
ENSCGRG00001012941	267.656297	1.03236244	0.04594956	0.12405535	0.6524311	0.79855173
ENSCGRG00001012946	10408.5436	1.12125377	0.16511284	0.04534007	0.00018971	0.00143205
ENSCGRG00001012947	47.8905284	-1.341514	-0.4238621	0.32849602	0.01807011	0.06334925
ENSCGRG00001012950	53488.9621	1.00707818	0.01017569	0.21009945	0.84374979	0.91589143
ENSCGRG00001012960	53.5278296	-1.0215088	-0.0307017	0.18859787	0.74255769	0.85638464
ENSCGRG00001012966	49.1947056	-1.037041	-0.0524729	0.18105214	0.61227264	0.77189377
ENSCGRG00001012980	378.543985	-1.0478023	-0.0673665	0.10883715	0.47271617	0.66405243
ENSCGRG00001012985	40.8394934	-1.0127804	-0.0183214	0.18651824	0.84441734	0.91622786
ENSCGRG00001012986	22.6358019	-1.047143	-0.0664585	0.211746	0.39529003	0.59593975
ENSCGRG00001012991	708.679464	1.00837867	0.01203751	0.07948447	0.86892207	0.92920041
ENSCGRG00001013010	5.31257704	1.02110122	0.03012589	0.21275568	0.53789054	0.71655343
ENSCGRG00001013012	4.96737952	1.00203024	0.00292605	0.21130486	0.94526228	0.97295716
ENSCGRG00001013019	181.504864	1.15266713	0.20497595	0.15861074	0.08623165	0.20944774
ENSCGRG00001013035	8.45824886	-1.0210134	-0.0300018	0.21230349	0.5414532	0.71951894
ENSCGRG00001013045	429.592446	-1.0735581	-0.1024003	0.10496295	0.26305195	0.45616562
ENSCGRG00001013048	983.355916	1.17674834	0.23480582	0.08075633	0.00173665	0.00926553
ENSCGRG00001013066	30.8377218	1.02280039	0.03252462	0.198854	0.69321232	0.82455591
ENSCGRG00001013067	49.4519983	-1.4572256	-0.5432242	0.36240612	0.00890521	0.0355487
ENSCGRG00001013068	27.2899752	-4.5825771	-2.1961592	0.48037104	2.00E-07	2.93E-06
ENSCGRG00001013076	7044.98438	-1.3285549	-0.4098578	0.09712151	6.07E-06	6.56E-05
ENSCGRG00001013098	305.683075	1.01469885	0.02105162	0.11808438	0.83394288	0.91104481
ENSCGRG00001013100	41.7073658	1.03418358	0.0484923	0.19919231	0.58105935	0.74725131
ENSCGRG00001013113	6041.38033	-1.2939845	-0.3718203	0.05850511	5.35E-11	1.39E-09
ENSCGRG00001013122	8.66512163	1.00457309	0.00658254	0.21014295	0.89011634	0.9416229
ENSCGRG00001013123	2203.86	-1.0465808	-0.0656837	0.07327214	0.33957382	0.53915971
ENSCGRG00001013124	12.9103892	-1.0482399	-0.0679689	0.21755743	0.32579782	0.52569216
ENSCGRG00001013129	24378.028	1.11219411	0.15340861	0.06999535	0.02013953	0.06899902
ENSCGRG00001013149	293.152099	-1.2408841	-0.3113684	0.16584874	0.01506803	0.05469898
ENSCGRG00001013171	10.4727245	1.04745843	0.06689299	0.22136406	0.2613346	0.45382919
ENSCGRG00001013176	1.67715314	1.00056405	0.00081352	0.21333926	0.97920756	0.99034912
ENSCGRG00001013181	3.50924346	-1.0202657	-0.0289449	0.21450219	0.4651286	0.65833506
ENSCGRG00001013183	60.0901593	-1.1360683	-0.1840496	0.2553112	0.12269043	0.27009185
ENSCGRG00001013190	107.392956	1.0243644	0.03472902	0.160393	0.74857936	0.8604901
ENSCGRG00001013201	3177.59785	-1.0382423	-0.0541431	0.20060518	0.53277768	0.71226071
ENSCGRG00001013217	12.0671469	1.00724089	0.01040875	0.20495744	0.87371325	0.93213247
ENSCGRG00001013219	63.5079353	-1.0699544	-0.0975493	0.17523186	0.38497219	0.58603609
ENSCGRG00001013221	6.86109743	1.05283121	0.07427416	0.22923646	0.11643701	0.26026514
ENSCGRG00001013224	5.56060738	-1.0474986	-0.0669484	0.22549954	0.15658895	0.32152461
ENSCGRG00001013232	305.095235	-1.0734648	-0.1022749	0.12446637	0.31746168	0.51628103
ENSCGRG00001013233	312.763139	-1.1691772	-0.2254937	0.13389086	0.0389778	0.11536636
ENSCGRG00001013239	36.6114273	1.10710606	0.14679344	0.24524266	0.15758666	0.32304131
ENSCGRG00001013240	1623.82866	1.03559708	0.0504628	0.098216	0.56429514	0.73589476
ENSCGRG00001013253	821.724529	1.09718266	0.13380372	0.09197556	0.107407	0.24534301

ENSCGRG00001013256	23.3467278	-1.0572108	-0.0802631	0.21521586	0.33214491	0.53198003
ENSCGRG00001013260	3147.46061	-1.2082647	-0.2729366	0.08649769	0.00061327	0.00386801
ENSCGRG00001013270	2.5482445	-1.0361146	-0.0511836	0.22322372	0.05409257	0.1475252
ENSCGRG00001013276	14.2836712	-1.0406528	-0.0574888	0.21605378	0.35298162	0.55265464
ENSCGRG00001013282	4.64865412	1.03897937	0.055167	0.22367199	0.10455411	0.24101821
ENSCGRG00001013286	1484.84807	-1.1811622	-0.2402071	0.06896704	0.00023381	0.00170465
ENSCGRG00001013293	2293.46692	-1.0839521	-0.116301	0.09938457	0.18635684	0.36364036
ENSCGRG00001013307	25.8376419	1.05756509	0.08074646	0.20734915	0.38773787	0.58919598
ENSCGRG00001013308	10.4727731	1.001017	0.00146648	0.20723293	0.98147607	0.99108517
ENSCGRG00001013314	2.04296563	-1.0157106	-0.0224894	0.21456992	0.49501569	0.68171472
ENSCGRG00001013331	878.090533	1.33863302	0.42076051	0.13209465	0.00025617	0.00183429
ENSCGRG00001013344	25.5040333	1.04241775	0.05993356	0.19648717	0.52763406	0.70881471
ENSCGRG00001013359	728.558573	1.00019414	0.00028005	0.09064993	0.97545448	0.98867578
ENSCGRG00001013371	169.076433	1.09111282	0.12580028	0.16124396	0.27456372	0.46993205
ENSCGRG00001013373	1364.08249	1.06438134	0.09001513	0.08241197	0.23676191	0.42617157
ENSCGRG00001013403	5753.19924	1.01943825	0.02777439	0.17132523	0.79051525	0.88303452
ENSCGRG00001013409	4.01258937	-1.0168936	-0.0241688	0.2157161	0.37837823	0.58047715
ENSCGRG00001013414	763.002363	1.038517	0.05452484	0.09766665	0.53289491	0.71228631
ENSCGRG00001013415	66.1352148	-1.068285	-0.0952966	0.19149784	0.37818015	0.58024766
ENSCGRG00001013420	350.823538	-1.0064891	-0.0093315	0.10680626	0.91893631	0.95810587
ENSCGRG00001013425	346.788636	1.07293645	0.10156463	0.10934136	0.28214392	0.47814044
ENSCGRG00001013431	456.699056	-1.4510949	-0.5371418	0.18588073	0.00044187	0.00292373
ENSCGRG00001013438	227.657711	-1.0837154	-0.115986	0.13697375	0.2815415	0.47749856
ENSCGRG00001013440	192.246835	-1.2625568	-0.3363482	0.25472158	0.02989304	0.09346591
ENSCGRG00001013441	39.7272457	1.03101235	0.04406162	0.18815006	0.65266892	0.7986117
ENSCGRG00001013447	6965.22603	-1.0838368	-0.1161476	0.05192798	0.02089977	0.07107141
ENSCGRG00001013452	14.8024013	-1.0061994	-0.0089162	0.1995514	0.90440229	0.95016758
ENSCGRG00001013457	2.25072543	-1.0080507	-0.0115683	0.21341336	0.71849731	0.84156113
ENSCGRG00001013461	79.5332942	1.00121383	0.00175012	0.17174356	0.98684047	0.99335426
ENSCGRG00001013465	2965.23964	1.05395806	0.07581746	0.05441605	0.1505177	0.3121975
ENSCGRG00001013467	65.8296812	-1.003187	-0.0045906	0.16557593	0.96400839	0.9827954
ENSCGRG00001013468	54.0910844	1.03187784	0.04527218	0.18544285	0.65100566	0.79764539
ENSCGRG00001013474	8.83117802	1.02508407	0.03574223	0.21303035	0.49012271	0.67753194
ENSCGRG00001013480	7818.92723	1.05897685	0.08267105	0.07579137	0.24370095	0.43388591
ENSCGRG00001013481	93.8133166	-1.1307557	-0.1772873	0.18046994	0.14812782	0.30867285
ENSCGRG00001013482	7153.36197	-1.2479465	-0.3195561	0.09731144	0.00032177	0.0022149
ENSCGRG00001013487	370.146888	1.04076266	0.05764111	0.13315976	0.58540191	0.7496
ENSCGRG00001013496	153.232742	-1.0479361	-0.0675508	0.16645473	0.53739288	0.71618839
ENSCGRG00001013517	1102.69974	-1.0097806	-0.0140418	0.09586603	0.86907855	0.92921261
ENSCGRG00001013544	5.58182312	-1.0119237	-0.0171005	0.2120913	0.68287292	0.81734403
ENSCGRG00001013570	226.350707	1.17455948	0.23211977	0.18225293	0.07022043	0.17955928
ENSCGRG00001013583	384.575068	1.049751	0.07004716	0.10730701	0.45231279	0.64717155
ENSCGRG00001013584	312.513649	1.11605721	0.15841098	0.10994932	0.09513356	0.22496971
ENSCGRG00001013592	583.598206	-1.0214934	-0.0306799	0.12803995	0.76218457	0.8685501
ENSCGRG00001013602	188.526075	-1.1438896	-0.1939478	0.15374724	0.0970945	0.22809106
ENSCGRG00001013607	9.31979591	-1.0120942	-0.0173436	0.20629657	0.77669021	0.87544229
ENSCGRG00001013609	974.764789	-1.0069767	-0.0100304	0.09126408	0.89527483	0.9453075
ENSCGRG00001013619	77.6498343	1.06919374	0.09652329	0.18588846	0.38414776	0.58537304
ENSCGRG00001013632	186.739496	-1.122556	-0.1667874	0.18048235	0.17005747	0.34122955
ENSCGRG00001013634	176.250998	1.04271415	0.06034371	0.14616061	0.57995961	0.74651152

ENSCGRG00001013643	289.487743	-1.1392927	-0.1881385	0.16641954	0.11863562	0.26346768
ENSCGRG00001013652	259.565736	-1.0892207	-0.1232963	0.18002919	0.28760934	0.48381821
ENSCGRG00001013654	132.412704	1.01441122	0.0206426	0.14448626	0.84896486	0.91874967
ENSCGRG00001013659	1759.15198	-1.1080233	-0.1479882	0.09359035	0.07817683	0.19483475
ENSCGRG00001013666	993.545392	-1.1093689	-0.1497391	0.09470445	0.07770129	0.19395041
ENSCGRG00001013669	1369.25692	-1.2111733	-0.2764053	0.07932724	0.00019087	0.00143829
ENSCGRG00001013673	1735.14576	1.02921353	0.04154233	0.10143021	0.64326449	0.79213033
ENSCGRG00001013677	5.66475284	-1.0620358	-0.0868324	0.23287413	0.13041802	0.28185486
ENSCGRG00001013683	131.874992	1.24372252	0.31466465	0.20334987	0.02637262	0.08492541
ENSCGRG00001013687	352.836646	1.0147158	0.02107572	0.11070928	0.82559524	0.90624465
ENSCGRG00001013713	9.34936571	-1.0530627	-0.0745914	0.2230448	0.24249008	0.43246665
ENSCGRG00001013716	3989.56095	-1.1200337	-0.1635421	0.07886622	0.02533376	0.08242965
ENSCGRG00001013738	164.930121	1.04544039	0.06411081	0.14491683	0.55578538	0.73051619
ENSCGRG00001013739	2301.41925	-1.1501284	-0.2017949	0.07305855	0.00325554	0.01564665
ENSCGRG00001013740	650.423188	-1.0159514	-0.0228314	0.08768809	0.77420304	0.87402317
ENSCGRG00001013750	11.1173829	1.04030743	0.05700994	0.21818691	0.31677461	0.51567959
ENSCGRG00001013770	2180.51854	-1.2081536	-0.2728039	0.07617046	0.000135	0.00105837
ENSCGRG00001013778	1899.37229	1.04427245	0.06249816	0.06339559	0.30168911	0.49943622
ENSCGRG00001013783	5.95271231	-1.0181555	-0.0259579	0.21301933	0.55261619	0.72866839
ENSCGRG00001013790	148.008564	-1.0242841	-0.034616	0.14424419	0.74620997	0.85906591
ENSCGRG00001013807	445.077082	1.14595942	0.19655596	0.12258812	0.05648895	0.15256661
ENSCGRG00001013811	21.716889	1.00142958	0.00206097	0.19558626	0.98170861	0.99108517
ENSCGRG00001013819	51.2398579	-1.008585	-0.0123327	0.17596574	0.90190966	0.94921321
ENSCGRG00001013826	2.79319085	-1.0124924	-0.0179111	0.212886	0.64295301	0.79201333
ENSCGRG00001013848	667.380046	1.04756057	0.06703366	0.1133947	0.48846356	0.67686537
ENSCGRG00001013868	1244.0685	1.0668364	0.09333895	0.07035481	0.15987042	0.32585903
ENSCGRG00001013871	8.72237349	-1.0667096	-0.0931675	0.2369273	0.10754149	0.24551888
ENSCGRG00001013880	151.781438	-1.1362543	-0.1842858	0.14495373	0.10353602	0.23957666
ENSCGRG00001013885	236.446751	-1.0087678	-0.0125941	0.12404984	0.90007249	0.9483409
ENSCGRG00001013886	243.345545	1.01596815	0.02285518	0.13074055	0.82706601	0.90731486
ENSCGRG00001013889	608.160078	-1.0780071	-0.1083667	0.10957334	0.25345507	0.44500847
ENSCGRG00001013894	7.79797045	-1.0382455	-0.0541476	0.21705409	0.33829372	0.53779382
ENSCGRG00001013899	78.9928267	-1.1580227	-0.2116636	0.21398008	0.10446325	0.24089545
ENSCGRG00001013900	50.3639881	1.01861346	0.02660669	0.17828807	0.79447041	0.88578189
ENSCGRG00001013901	4667.87227	-1.0146697	-0.0210102	0.05588319	0.6965851	0.82661001
ENSCGRG00001013902	5.51059099	-1.0443929	-0.0626645	0.22328431	0.19119657	0.36980643
ENSCGRG00001013927	8.26278446	-8.3594154	-3.063402	1.09975523	0.00025409	0.0018232
ENSCGRG00001013937	11.0410085	-3.2105469	-1.6828191	0.80415352	0.00110971	0.00635183
ENSCGRG00001013940	174.421988	1.00897374	0.01288862	0.16175067	0.90571114	0.95103172
ENSCGRG00001013943	2.00294291	1.01858917	0.02657228	0.21635364	0.29694532	0.49465144
ENSCGRG00001013947	3467.58636	-1.1025329	-0.1408217	0.06834848	0.02905097	0.09132341
ENSCGRG00001013973	5722.18197	-1.1917265	-0.2530531	0.07488839	0.00031665	0.00218467
ENSCGRG00001013976	200.235221	-1.0240197	-0.0342435	0.16087126	0.74876365	0.86055761
ENSCGRG00001014007	123.820637	1.03816304	0.05403303	0.16524069	0.62168161	0.77793703
ENSCGRG00001014011	1460.44523	1.03462161	0.04910323	0.06186964	0.40736225	0.60664679
ENSCGRG00001014032	23.0768389	-1.0564233	-0.079188	0.20959107	0.37657272	0.57847422
ENSCGRG00001014044	148.554364	1.08634252	0.11947905	0.15990585	0.29797304	0.49571879
ENSCGRG00001014060	12.7609144	-1.0346918	-0.0492011	0.20961535	0.48475581	0.67365176
ENSCGRG00001014067	55.9377776	-1.0003997	-0.0005765	0.18370996	0.99288857	0.99685733
ENSCGRG00001014074	65.3044497	-1.0277503	-0.0394898	0.18762892	0.6808286	0.81638251

ENSCGRG00001014075	127.337046	1.76929266	0.8231727	0.19822066	2.49E-06	2.97E-05
ENSCGRG00001014077	111.171774	-1.0499482	-0.0703181	0.15933968	0.5251992	0.70682469
ENSCGRG00001014084	5637.95057	1.00857899	0.01232408	0.05506534	0.81684255	0.90103789
ENSCGRG00001014091	3.19614933	-1.0134633	-0.0192938	0.21282748	0.62808865	0.78259076
ENSCGRG00001014094	31.5167513	-1.0665806	-0.0929931	0.24564291	0.00142516	0.0078052
ENSCGRG00001014096	665.267585	-1.0990756	-0.1362906	0.108934	0.14896404	0.3098855
ENSCGRG00001014097	573.087186	1.15097367	0.20285483	0.09781721	0.02030436	0.06945231
ENSCGRG00001014099	4.538135	1.02136599	0.03049992	0.21450399	0.46576399	0.65883797
ENSCGRG00001014111	7829.85971	-1.144195	-0.1943329	0.0690167	0.00286792	0.01406511
ENSCGRG00001014116	343.285802	1.02002674	0.02860698	0.11097949	0.76465704	0.86962346
ENSCGRG00001014126	338.74726	-1.1298125	-0.1760833	0.12059209	0.08204659	0.20165119
ENSCGRG00001014136	18.1861937	1.06334441	0.08860895	0.22303994	0.26106048	0.45357179
ENSCGRG00001014167	17.4321147	-1.0445219	-0.0628427	0.2076938	0.44449307	0.64099039
ENSCGRG00001014173	23.9301149	-1.2807208	-0.356956	0.54456913	0.02290216	0.07640904
ENSCGRG00001014180	843.754646	1.12973535	0.17598485	0.07231478	0.00955058	0.03769037
ENSCGRG00001014204	6253.21129	-1.1650677	-0.2204137	0.0534245	1.95E-05	0.00018686
ENSCGRG00001014214	1.82283571	1.01600435	0.02290659	0.21542515	0.42469551	0.6230359
ENSCGRG00001014227	181.788694	1.02087966	0.02981282	0.12720599	0.77240885	0.87322896
ENSCGRG00001014240	12.4603196	1.03428793	0.04863787	0.21079552	0.47124734	0.66286136
ENSCGRG00001014263	151.281326	-1.0313143	-0.044484	0.16173284	0.68028577	0.8159652
ENSCGRG00001014273	197.873555	-1.1390777	-0.1878661	0.16437147	0.11812898	0.26275954
ENSCGRG00001014282	187.926003	-1.01824	-0.0260776	0.14217088	0.80182861	0.89053446
ENSCGRG00001014291	2658.73024	-1.0377756	-0.0534945	0.08245895	0.48086755	0.67119823
ENSCGRG00001014295	1452.87315	-1.0575835	-0.0807716	0.08388447	0.29423076	0.49157037
ENSCGRG00001014325	21.0883848	1.01905011	0.02722499	0.1984512	0.74002534	0.85526205
ENSCGRG00001014333	3326.76102	-1.1140617	-0.1558291	0.05493815	0.00294436	0.01437395
ENSCGRG00001014343	152.407019	1.01294315	0.01855321	0.14612305	0.86459589	0.92717978
ENSCGRG00001014361	860.199934	1.16127482	0.21570943	0.11313684	0.02726392	0.08698651
ENSCGRG00001014369	30.8231386	-1.0726949	-0.1012397	0.20794779	0.32056626	0.51921277
ENSCGRG00001014372	65.8461227	1.04457808	0.06292034	0.17598022	0.56034142	0.73334426
ENSCGRG00001014373	2419.00648	-1.0687965	-0.0959872	0.084281	0.21416027	0.40008648
ENSCGRG00001014391	28.6555298	-1.050505	-0.071083	0.19543728	0.4722892	0.66385249
ENSCGRG00001014404	313.102479	-1.0041154	-0.005925	0.13009115	0.95318481	0.97744936
ENSCGRG00001014430	198.361319	1.02419724	0.03449358	0.14145553	0.74824444	0.86041394
ENSCGRG00001014440	849.927283	-1.0277272	-0.0394574	0.08612238	0.61557513	0.77392457
ENSCGRG00001014450	4.25976628	1.0102679	0.01473791	0.21251874	0.70558687	0.83327813
ENSCGRG00001014458	8592.0888	-1.0732187	-0.1019441	0.05821759	0.06989905	0.17904555
ENSCGRG00001014459	11.3339635	1.00051317	0.00074016	0.20998662	0.99026591	0.9954343
ENSCGRG00001014475	953.551059	-1.0253144	-0.0360663	0.08632074	0.64731643	0.79510245
ENSCGRG00001014478	896.217436	1.10759808	0.14743446	0.1207096	0.14818675	0.30867285
ENSCGRG00001014489	526.850414	1.09016094	0.12454113	0.09641218	0.14927138	0.31026553
ENSCGRG00001014492	4038.07915	-1.1731545	-0.230393	0.092498	0.00629883	0.02662972
ENSCGRG00001014496	230.426912	-1.0077484	-0.0111356	0.12640647	0.91237128	0.95462518
ENSCGRG00001014500	57.0360611	-1.0028599	-0.00412	0.17815786	0.96568519	0.98312856
ENSCGRG00001014501	8.18669371	-1.0128149	-0.0183705	0.20762876	0.75255746	0.8628589
ENSCGRG00001014514	20.6195716	-5.0396242	-2.3333161	0.5412956	8.62E-07	1.13E-05
ENSCGRG00001014515	59.4501286	-1.5510412	-0.633237	0.28775216	0.00198791	0.01038121
ENSCGRG00001014517	2.92256188	1.00682995	0.00982003	0.21353743	0.74843271	0.86047595
ENSCGRG00001014521	1977.66411	1.32941587	0.41079249	0.06842579	4.25E-10	9.44E-09
ENSCGRG00001014532	3.13065304	1.02527265	0.03600762	0.21750666	0.27520033	0.47064042

ENSCGRG00001014554	92.5350941	1.02159084	0.0308175	0.16327968	0.77602723	0.87520555
ENSCGRG00001014561	4762.71859	-1.0303375	-0.043117	0.0591747	0.44786627	0.64361307
ENSCGRG00001014567	2.46893261	-1.0092682	-0.0133096	0.21359244	0.67951338	0.81556886
ENSCGRG00001014574	5338.00572	-1.1905068	-0.2515758	0.06919048	0.00011612	0.00093085
ENSCGRG00001014579	126.1906	-1.0473621	-0.0667603	0.15221785	0.54247308	0.72027106
ENSCGRG00001014592	518.35894	-1.0306047	-0.043491	0.09935639	0.62099396	0.77745467
ENSCGRG00001014608	5.05207437	1.00432562	0.0062271	0.21071462	0.88925664	0.94096956
ENSCGRG00001014612	307.22843	1.04154532	0.05872562	0.1357787	0.58050718	0.74684111
ENSCGRG00001014625	366.155405	1.02501407	0.03564372	0.1074161	0.70243406	0.83058637
ENSCGRG00001014660	43.2107769	-1.0836665	-0.1159208	0.22649316	0.22695853	0.41557157
ENSCGRG00001014662	776.238641	1.03275745	0.04650147	0.1751969	0.66119395	0.80385712
ENSCGRG00001014666	4796.22818	1.01333998	0.01911829	0.07242509	0.78003973	0.87690293
ENSCGRG00001014669	69.8034425	-1.044209	-0.0624105	0.19419028	0.5200465	0.70286935
ENSCGRG00001014690	94.8958445	1.36367733	0.44750231	0.29741194	0.0129868	0.04847344
ENSCGRG00001014695	306.124912	-1.0709429	-0.0988816	0.13266621	0.34956612	0.54921025
ENSCGRG00001014697	106.577699	-1.1611931	-0.215608	0.21339722	0.09885747	0.23101805
ENSCGRG00001014716	1323.89188	-1.1018686	-0.1399522	0.06864774	0.03090941	0.09605764
ENSCGRG00001014720	2483.67983	-1.1231802	-0.1675894	0.07160544	0.01271371	0.04765381
ENSCGRG00001014722	7.78284998	-1.0419684	-0.0593115	0.22434004	0.1214111	0.26782782
ENSCGRG00001014729	199.834668	1.0336695	0.04777498	0.12707895	0.64247656	0.79181496
ENSCGRG00001014769	180.540921	1.16034872	0.21455844	0.17245917	0.08552598	0.20804934
ENSCGRG00001014779	433.208321	-1.0456989	-0.0644676	0.11091341	0.49728539	0.68369178
ENSCGRG00001014784	240.50404	-1.1146625	-0.156607	0.12892323	0.13627256	0.29079652
ENSCGRG00001014797	194.341617	-1.8765381	-0.9080735	0.19342075	1.85E-07	2.73E-06
ENSCGRG00001014806	147.499503	-1.6772931	-0.7461348	0.18659819	5.14E-06	5.65E-05
ENSCGRG00001014808	208.953914	1.06509777	0.09098587	0.15591117	0.41697244	0.61586728
ENSCGRG00001014816	152.983795	1.02219289	0.03166746	0.14688682	0.76871419	0.87144584
ENSCGRG00001014818	573.428004	-1.0513583	-0.0722544	0.11719833	0.46330413	0.65677783
ENSCGRG00001014830	74.9509811	1.00778137	0.01118269	0.17318534	0.91898883	0.95810587
ENSCGRG00001014845	422.625736	-1.1323209	-0.1792828	0.14633376	0.11368928	0.25563625
ENSCGRG00001014849	1433.47357	1.10391725	0.14263203	0.08829912	0.07534317	0.18928143
ENSCGRG00001014852	71.8376003	-1.0470962	-0.066394	0.18277259	0.52662618	0.7079527
ENSCGRG00001014896	416.659333	-1.200688	-0.2638614	0.11060462	0.00640072	0.02697548
ENSCGRG00001014904	179.776116	-1.1652662	-0.2206596	0.15784606	0.06491907	0.17023359
ENSCGRG00001014928	127.198974	-1.0418097	-0.0590917	0.14721881	0.5863918	0.74996795
ENSCGRG00001014941	36.6989702	1.16799947	0.22403962	0.30903271	0.0705632	0.18017033
ENSCGRG00001015024	1.65604996	1.00524457	0.00754654	0.21376367	0.79090775	0.88315274
ENSCGRG00001015031	13067.2703	-1.0480901	-0.0677628	0.06959918	0.30140636	0.49928806
ENSCGRG00001015054	799.498489	-1.1426398	-0.1923707	0.11758808	0.05469062	0.14876472
ENSCGRG00001015077	82.6034874	1.00239016	0.00344415	0.16863791	0.97383343	0.98805671
ENSCGRG00001015089	953.191992	1.24777402	0.31935667	0.11596306	0.00169124	0.00903831
ENSCGRG00001015102	1130.4078	-1.0098301	-0.0141126	0.07950408	0.84753444	0.91805481
ENSCGRG00001015107	755.948268	-1.0474304	-0.0668544	0.09165064	0.42066114	0.61917376
ENSCGRG00001015116	2460.43255	-1.2135218	-0.2792	0.08662227	0.00046521	0.00305136
ENSCGRG00001015122	982.874816	1.25831557	0.33149378	0.07347437	1.92E-06	2.35E-05
ENSCGRG00001015137	41.8087857	-1.0031199	-0.0044941	0.18225513	0.96160381	0.98168193
ENSCGRG00001015140	43.6525062	-1.0326791	-0.046392	0.20125891	0.57727069	0.74410617
ENSCGRG00001015155	3.89006479	-1.0180215	-0.025768	0.21552313	0.39953331	0.59966476
ENSCGRG00001015162	1039.65563	-1.0163288	-0.0233673	0.10621477	0.79919957	0.88869327
ENSCGRG00001015164	2423.19539	1.06747053	0.09419625	0.06431088	0.12443078	0.27284404

ENSCGRG00001015177	63.1317114	1.90779205	0.93190393	0.27177049	3.70E-05	0.00033357
ENSCGRG00001015178	1470.88768	-1.1199043	-0.1633754	0.0721962	0.01589003	0.05706511
ENSCGRG00001015202	52.6141319	-1.1028309	-0.1412116	0.22979882	0.19182425	0.37051699
ENSCGRG00001015206	54.2891849	1.00098586	0.00142159	0.18524793	0.98985196	0.99536884
ENSCGRG00001015222	1724.26038	-1.1388086	-0.1875252	0.08610805	0.01734067	0.06137784
ENSCGRG00001015235	739.179639	-1.1393273	-0.1881822	0.11167439	0.05090559	0.14099474
ENSCGRG00001015256	2.40980586	-1.0072185	-0.0103767	0.21260135	0.7747385	0.87439649
ENSCGRG00001015258	2268.98132	-1.0051616	-0.0074274	0.08866923	0.92702258	0.96295486
ENSCGRG00001015264	9.10004916	1.01574673	0.02254072	0.20696218	0.71753274	0.84141289
ENSCGRG00001015291	7.30918401	1.07401526	0.1030145	0.24804114	0.03561029	0.10738414
ENSCGRG00001015306	2.64877298	-1.005756	-0.0082804	0.21325553	0.79139392	0.88354158
ENSCGRG00001015309	184.904224	1.0232958	0.03322324	0.14114896	0.75722203	0.86530011
ENSCGRG00001015311	2.60514412	-1.0311682	-0.0442796	0.22017965	0.14507194	0.30450133
ENSCGRG00001015316	231.21253	1.09673215	0.13321122	0.12857155	0.20445227	0.38823505
ENSCGRG00001015319	671.265923	-1.0683805	-0.0954256	0.10907031	0.30999988	0.50839821
ENSCGRG00001015320	36.358292	1.098026	0.13491221	0.25192995	0.12794306	0.27811822
ENSCGRG00001015323	174.137353	1.22537246	0.29322033	0.17671942	0.02594974	0.08388498
ENSCGRG00001015333	76.1214255	-1.7003474	-0.7658296	0.27368409	0.00034511	0.00235402
ENSCGRG00001015345	3084.73067	-1.1269579	-0.1724336	0.08542514	0.02742357	0.08732187
ENSCGRG00001015358	55.0551695	1.07202818	0.10034282	0.19387783	0.356337	0.55646281
ENSCGRG00001015375	2438.25783	-1.098995	-0.1361849	0.05875466	0.01564938	0.05643824
ENSCGRG00001015394	109.524562	-1.3972367	-0.4825765	0.26831188	0.00698842	0.02901838
ENSCGRG00001015397	32.9264121	1.0140995	0.02019921	0.19092918	0.82420452	0.90510587
ENSCGRG00001015414	1017.47722	-1.1928477	-0.2544099	0.09899359	0.00407234	0.01877173
ENSCGRG00001015424	52.3387283	-1.2692794	-0.3440097	0.36968596	0.03310121	0.10151461
ENSCGRG00001015427	1557.40535	-1.0194583	-0.0278028	0.0701247	0.7061234	0.83360434
ENSCGRG00001015437	2098.77075	-1.0340588	-0.0483182	0.06004535	0.40152414	0.60108967
ENSCGRG00001015440	383.643108	1.12678451	0.17221164	0.14266457	0.12399193	0.27216186
ENSCGRG00001015454	475.977096	-1.1747048	-0.2322983	0.10839983	0.01445677	0.05298746
ENSCGRG00001015466	42.5990129	1.0147193	0.02108069	0.17887923	0.83550079	0.91187361
ENSCGRG00001015476	109.713804	-1.0400102	-0.0565977	0.15012182	0.6027077	0.76472667
ENSCGRG00001015486	26.1488325	-1.0021426	-0.0030879	0.1889475	0.97124869	0.98673136
ENSCGRG00001015498	2.15657006	-1.0188792	-0.026983	0.21637092	0.30923498	0.50753364
ENSCGRG00001015506	17667.2314	1.13183428	0.17866274	0.05446699	0.00068361	0.00423251
ENSCGRG00001015511	2611.1819	-1.1437842	-0.1938149	0.05814737	0.00051704	0.00334845
ENSCGRG00001015513	4072.52137	-1.08729	-0.1207367	0.14271738	0.27030392	0.46562005
ENSCGRG00001015523	241.9939	1.07143786	0.09954818	0.12945368	0.34174421	0.54138327
ENSCGRG00001015533	500.069967	-1.1441423	-0.1942665	0.10581399	0.03593982	0.10814827
ENSCGRG00001015540	2.91843155	-1.0051289	-0.0073806	0.21334517	0.80756743	0.89434934
ENSCGRG00001015549	2776.69445	-1.2123709	-0.2778311	0.06170913	2.66E-06	3.14E-05
ENSCGRG00001015553	10.0785736	-1.0152161	-0.0217869	0.20366335	0.75421891	0.86317158
ENSCGRG00001015564	684.08329	-1.001574	-0.002269	0.10169575	0.97885286	0.99034912
ENSCGRG00001015573	6.25873225	-1.0250314	-0.0356681	0.21293776	0.48865782	0.67693697
ENSCGRG00001015588	4309.81803	1.28635404	0.36328777	0.06380897	3.26E-09	6.44E-08
ENSCGRG00001015594	250.205419	-1.0703472	-0.0980789	0.17102246	0.38288023	0.58437934
ENSCGRG00001015597	702.00663	1.08670838	0.11996484	0.10880421	0.20318184	0.3863954
ENSCGRG00001015599	1670.89237	-1.1143182	-0.1561613	0.06959761	0.01736188	0.06140409
ENSCGRG00001015607	1264.44366	-1.1148958	-0.1569089	0.10951656	0.09695871	0.22798111
ENSCGRG00001015618	15.8551133	1.00864064	0.01241226	0.20243872	0.86198387	0.92618801
ENSCGRG00001015623	1613.53364	-1.1229195	-0.1672545	0.07368805	0.01532303	0.0554486

ENSCGRG00001015633	6.85294011	-1.0301242	-0.0428183	0.2136809	0.44166383	0.63907133
ENSCGRG00001015635	1112.66875	-1.0493936	-0.0695559	0.07075505	0.2968082	0.49455164
ENSCGRG00001015643	391.753567	1.51739416	0.60159589	0.14705968	4.47E-06	5.00E-05
ENSCGRG00001015675	2856.61952	-1.1667399	-0.2224829	0.15516872	0.06087391	0.16188182
ENSCGRG00001015690	835.698667	1.07189102	0.10015823	0.08134436	0.18087333	0.356734
ENSCGRG00001015694	2604.60275	1.00441199	0.00635115	0.06497758	0.91876732	0.95803088
ENSCGRG00001015719	2.13564159	-1.026019	-0.0370574	0.21853124	0.18745414	0.36499279
ENSCGRG00001015727	3719.79771	-1.011515	-0.0165177	0.07061588	0.80347723	0.89151523
ENSCGRG00001015731	1249.07968	-1.2197517	-0.2865875	0.07336976	3.47E-05	0.00031442
ENSCGRG00001015740	3556.99764	-1.0885241	-0.1223734	0.05502561	0.0210678	0.07154785
ENSCGRG00001015745	783.722857	1.04294205	0.060659	0.09918212	0.49233226	0.67949091
ENSCGRG00001015752	13258.9401	1.02637962	0.03756443	0.0376223	0.31370307	0.5125684
ENSCGRG00001015765	2.13603007	-1.0053766	-0.007736	0.21445055	0.72559623	0.84545095
ENSCGRG00001015776	162.640842	1.07644209	0.10627071	0.14067437	0.33106846	0.53105271
ENSCGRG00001015782	3.32446714	1.0310847	0.04416284	0.22037169	0.13208453	0.28417918
ENSCGRG00001015787	5.1288313	-1.0758342	-0.1054558	0.24801773	0.04871902	0.13662612
ENSCGRG00001015809	83.0450356	-1.001508	-0.002174	0.16936917	0.98203996	0.99108517
ENSCGRG00001015812	6.60736355	-1.0108879	-0.0156231	0.2116422	0.7170021	0.8412527
ENSCGRG00001015827	14248.5224	-1.1536827	-0.2062465	0.05633878	0.00014321	0.00111317
ENSCGRG00001015828	247.07819	-1.0795468	-0.1104257	0.14024228	0.30944868	0.50781932
ENSCGRG00001015831	5988.57997	-1.0822285	-0.1140052	0.06657219	0.06974229	0.17882282
ENSCGRG00001015832	6.03130313	1.04020798	0.05687201	0.22002388	0.26747629	0.46183735
ENSCGRG00001015842	8.68690573	-1.032797	-0.0465567	0.21516418	0.39236642	0.59362393
ENSCGRG00001015845	127.833569	-1.2357069	-0.3053366	0.20140945	0.02926368	0.09181183
ENSCGRG00001015868	615.846367	-1.0345055	-0.0489413	0.11434491	0.61355924	0.77269695
ENSCGRG00001015872	2291.04635	-1.0997912	-0.1372297	0.10201935	0.12628218	0.27539577
ENSCGRG00001015874	563.607594	1.00397662	0.00572567	0.0975522	0.94818959	0.97437186
ENSCGRG00001015875	3.24681973	-1.0232279	-0.0331275	0.21712533	0.27354042	0.469209
ENSCGRG00001015878	1362.68842	-1.0307996	-0.043764	0.10076717	0.6225594	0.77850311
ENSCGRG00001015880	938.619169	-1.0260855	-0.037151	0.1156513	0.70246469	0.83058637
ENSCGRG00001015895	27.9578941	-1.0098337	-0.0141177	0.21215671	0.72203645	0.84343968
ENSCGRG00001015898	3182.22593	1.00122952	0.00177273	0.07547775	0.98089287	0.99088655
ENSCGRG00001015915	4.53597659	-1.0161613	-0.0231294	0.21326725	0.57040361	0.73959094
ENSCGRG00001015949	5.59385314	-1.0167173	-0.0239186	0.2108127	0.63960603	0.79058554
ENSCGRG00001015954	52.4178204	1.03826648	0.05417676	0.1972486	0.55604188	0.73070338
ENSCGRG00001015979	1228.54439	-1.0683043	-0.0953227	0.07200852	0.15907762	0.32536871
ENSCGRG00001015981	47.611977	1.76790578	0.82204139	0.47386461	0.00349735	0.01658071
ENSCGRG00001015986	25.5044522	-1.0585793	-0.0821294	0.21145767	0.35768661	0.55775598
ENSCGRG00001015988	44.2013712	-1.0411536	-0.0581829	0.19653232	0.52997909	0.71059578
ENSCGRG00001015991	30.7040585	-1.0195027	-0.0278656	0.18721457	0.76826183	0.8713089
ENSCGRG00001016005	5.35133441	-1.0210415	-0.0300416	0.21215666	0.54948294	0.72629135
ENSCGRG00001016024	6543.57868	-1.0644106	-0.0900548	0.04971159	0.06178689	0.16383566
ENSCGRG00001016025	1172.67249	1.02315392	0.03302319	0.0856411	0.67522184	0.81263946
ENSCGRG00001016030	102.208086	-1.0876281	-0.1211853	0.16104173	0.28987243	0.48630818
ENSCGRG00001016035	967.28621	-1.0096581	-0.0138668	0.11662326	0.89038862	0.9416229
ENSCGRG00001016036	92.1186223	1.12167982	0.16566093	0.20218046	0.17584304	0.34955763
ENSCGRG00001016037	5152.95289	-1.0609044	-0.0852947	0.0622288	0.1517479	0.31390274
ENSCGRG00001016045	4387.97272	1.05299647	0.0745006	0.06289432	0.21488051	0.40108105
ENSCGRG00001016049	443.218778	-1.1198833	-0.1633484	0.13955306	0.13843079	0.29412811
ENSCGRG00001016064	439.556724	-1.011019	-0.0158101	0.11436565	0.87033495	0.92970236

ENSCGRG00001016067	4.89923933	-1.0421135	-0.0595124	0.22096545	0.24310485	0.43303398
ENSCGRG00001016087	72.5575783	1.03689252	0.05226636	0.1722346	0.62763564	0.78248298
ENSCGRG00001016092	1.87517583	-1.0180478	-0.0258053	0.21684615	0.22500987	0.41364426
ENSCGRG00001016100	403.296873	1.14648716	0.1972202	0.11438626	0.04464004	0.12784238
ENSCGRG00001016103	4.70276217	1.02155529	0.03076729	0.2140147	0.47563754	0.66640238
ENSCGRG00001016108	28.0002935	1.95338741	0.9659781	0.61784912	0.00360531	0.01699193
ENSCGRG00001016119	790.476461	-1.0884287	-0.1222469	0.10825416	0.19639847	0.37705918
ENSCGRG00001016123	7253.62792	-1.2303337	-0.2990497	0.05256545	4.57E-09	8.80E-08
ENSCGRG00001016131	385.581822	-1.2149958	-0.2809514	0.15839982	0.02263039	0.07571043
ENSCGRG00001016135	426.094965	-1.1567798	-0.2101142	0.1030406	0.02103402	0.0714521
ENSCGRG00001016157	45.6338444	1.01843163	0.02634913	0.17975165	0.79534475	0.88617607
ENSCGRG00001016163	637.9259	-1.3771533	-0.4616892	0.11992702	1.87E-05	0.00018105
ENSCGRG00001016179	359.901624	-1.023477	-0.0334787	0.12289701	0.7394783	0.85480106
ENSCGRG00001016185	394.257185	1.02760612	0.03928739	0.09989917	0.66173054	0.80429905
ENSCGRG00001016191	76.6585576	-1.0132955	-0.0190549	0.16858907	0.85500136	0.92247683
ENSCGRG00001016192	591.952297	1.1814692	0.24058201	0.09594899	0.00543632	0.02367878
ENSCGRG00001016196	635.19058	1.00252142	0.00363307	0.09787995	0.96765861	0.98425695
ENSCGRG00001016201	517.745867	-1.1165218	-0.1590114	0.11644521	0.10734448	0.24528769
ENSCGRG00001016206	281.523627	-1.1457913	-0.1963443	0.15708937	0.09719146	0.2281933
ENSCGRG00001016214	969.866329	1.16486182	0.22015882	0.09748118	0.0118025	0.04503726
ENSCGRG00001016215	116.828863	-1.1895188	-0.2503781	0.21758601	0.06673658	0.17344198
ENSCGRG00001016217	5678.3813	-1.1444782	-0.1946899	0.04596792	1.18E-05	0.00012042
ENSCGRG00001016247	3946.5342	-1.1827522	-0.2421479	0.09248403	0.00390104	0.01811837
ENSCGRG00001016254	823.833409	-1.1793424	-0.2379826	0.10103996	0.00815711	0.0329942
ENSCGRG00001016261	8546.19904	-1.0450748	-0.0636061	0.0476833	0.17078969	0.34227407
ENSCGRG00001016264	11049.0559	-1.2555792	-0.328353	0.06334317	6.76E-08	1.08E-06
ENSCGRG00001016282	1583.87201	-1.1578298	-0.2114232	0.07151537	0.00168756	0.00903372
ENSCGRG00001016284	4.56374399	1.00044862	0.00064707	0.21000136	0.99076087	0.99564272
ENSCGRG00001016292	168.385122	-1.6932025	-0.7597546	0.20840531	1.95E-05	0.00018686
ENSCGRG00001016294	15.4809553	1.01513825	0.02167622	0.20552852	0.74267611	0.856394
ENSCGRG00001016295	24.5657651	-1.0113469	-0.0162779	0.19334529	0.84949259	0.91908785
ENSCGRG00001016300	13.3213071	-2.2110732	-1.1447468	0.88325505	0.00420546	0.01928129
ENSCGRG00001016303	13.9767912	-1.0111299	-0.0159684	0.20083126	0.82844007	0.90788335
ENSCGRG00001016310	230.224134	-1.122062	-0.1661524	0.12954857	0.11665621	0.26047865
ENSCGRG00001016313	3026.28008	-1.0670217	-0.0935895	0.0512355	0.0595102	0.1590498
ENSCGRG00001016319	33.0779443	-1.1712619	-0.2280637	0.31237028	0.06820994	0.17612767
ENSCGRG00001016333	248.321577	1.12019336	0.16374778	0.13702925	0.13384747	0.28729659
ENSCGRG00001016334	2.73616051	-1.0237274	-0.0338316	0.21711733	0.28510317	0.48187146
ENSCGRG00001016366	1063.2104	-1.1471718	-0.1980815	0.12941235	0.06236255	0.16478223
ENSCGRG00001016369	1331.29354	-1.062973	-0.0881049	0.07405712	0.2036577	0.38717987
ENSCGRG00001016378	232.784033	-1.6083937	-0.6856206	0.21063018	9.20E-05	0.00075225
ENSCGRG00001016397	1679.67527	-1.506658	-0.591352	0.10715159	4.01E-09	7.79E-08
ENSCGRG00001016398	88.6501722	1.05791185	0.08121943	0.17281565	0.46308547	0.65671703
ENSCGRG00001016407	123.324857	-1.1392081	-0.1880313	0.19134745	0.13426736	0.28790518
ENSCGRG00001016413	273.335484	-1.1452398	-0.1956497	0.1321429	0.06879679	0.17730725
ENSCGRG00001016416	934.871707	1.02534115	0.036104	0.09926694	0.68884214	0.82176083
ENSCGRG00001016425	37.4749626	-1.0633593	-0.0886292	0.20476027	0.368761	0.57010448
ENSCGRG00001016427	1473.45216	-1.2113487	-0.2766142	0.07186705	4.63E-05	0.0004085
ENSCGRG00001016432	81.5257384	1.07384725	0.10278879	0.17992065	0.36407748	0.56503908
ENSCGRG00001016441	6.90130432	1.05352225	0.07522078	0.22681426	0.18074638	0.35659343

ENSCGRG00001016452	2.39724617	-1.0186614	-0.0266745	0.21772665	0.04874938	0.13667752
ENSCGRG00001016456	461.119532	-1.2830765	-0.3596072	0.10862109	0.00022921	0.00167496
ENSCGRG00001016459	257.04482	1.03844832	0.05442942	0.12206949	0.59060971	0.75390277
ENSCGRG00001016471	22.5852154	1.06241758	0.08735092	0.20962858	0.35361558	0.55322613
ENSCGRG00001016481	2841.89766	-1.0562974	-0.0790161	0.0607678	0.17481479	0.34816192
ENSCGRG00001016483	428.598479	1.00024317	0.00035078	0.13499345	0.99815023	0.99955475
ENSCGRG00001016488	377.896832	1.13253067	0.17955013	0.12805119	0.08905349	0.21467355
ENSCGRG00001016490	620.156408	-1.0220858	-0.0315163	0.09161184	0.70334262	0.83145143
ENSCGRG00001016509	391.884914	-1.1728246	-0.2299873	0.14404225	0.04544388	0.12962083
ENSCGRG00001016513	1992.03501	-1.1896291	-0.2505118	0.08375329	0.00119975	0.00675409
ENSCGRG00001016514	4.24884977	1.00543051	0.00781338	0.21308554	0.81200736	0.89735937
ENSCGRG00001016525	1256.25306	1.00693001	0.00996341	0.08378892	0.89808298	0.94694568
ENSCGRG00001016546	739.472367	-1.5605237	-0.6420303	0.10315734	5.34E-11	1.39E-09
ENSCGRG00001016551	294.312679	-1.1736371	-0.2309864	0.13528797	0.03585767	0.10795224
ENSCGRG00001016557	256.756656	1.37627982	0.46077382	0.13797434	0.0001294	0.00102007
ENSCGRG00001016563	45.6373023	1.0176694	0.02526896	0.17688554	0.80538273	0.89269198
ENSCGRG00001016565	36.7758695	-1.0853414	-0.118149	0.21140411	0.26919586	0.46421414
ENSCGRG00001016581	213.156359	-7.1773581	-2.8434529	0.19332782	2.52E-50	7.35E-48
ENSCGRG00001016590	16275.9471	-1.0726517	-0.1011816	0.14782516	0.379188	0.58130664
ENSCGRG00001016609	738.437615	1.01211623	0.01737498	0.08307606	0.81812576	0.90183211
ENSCGRG00001016611	445.34654	1.06150093	0.08610564	0.12889894	0.41181012	0.61099
ENSCGRG00001016651	3195.3042	1.00249198	0.00359069	0.0563101	0.94946526	0.97488378
ENSCGRG00001016653	631.562055	1.07319879	0.10191734	0.08730979	0.20115537	0.38362368
ENSCGRG00001016671	5.47452239	-1.0364935	-0.0517111	0.21983826	0.23915539	0.42897266
ENSCGRG00001016682	3.59820196	1.01395073	0.01998755	0.21456243	0.52067118	0.70309668
ENSCGRG00001016705	30.7695242	-1.115264	-0.1573852	0.24198278	0.15545684	0.31957567
ENSCGRG00001016712	2.47518214	-1.0289478	-0.0411697	0.22099646	0.01642082	0.05844698
ENSCGRG00001016730	145.694286	1.00397228	0.00571944	0.14315653	0.96080055	0.98137498
ENSCGRG00001016753	488.907453	1.15700131	0.21039049	0.11674836	0.03482989	0.10562758
ENSCGRG00001016759	750.612384	-1.0997235	-0.1371408	0.10674708	0.13941388	0.29562851
ENSCGRG00001016762	5393.95401	1.09074879	0.12531887	0.04466314	0.00406437	0.01875254
ENSCGRG00001016772	1129.49406	-1.1714918	-0.2283469	0.0787405	0.00181366	0.00962027
ENSCGRG00001016776	1244.38021	-1.1099056	-0.1504369	0.08541874	0.05422708	0.14786056
ENSCGRG00001016787	3.75762123	-1.014396	-0.020621	0.21238018	0.62971211	0.78354659
ENSCGRG00001016789	43.1728379	1.08640858	0.11956678	0.20936026	0.27249934	0.46833737
ENSCGRG00001016793	869.028609	1.04829199	0.06804062	0.08535391	0.3835341	0.58481988
ENSCGRG00001016799	956.668983	1.31012524	0.38970473	0.08159848	4.04E-07	5.56E-06
ENSCGRG00001016800	16940.3192	-2.0392566	-1.0280433	0.06547513	1.25E-56	4.34E-54
ENSCGRG00001016802	181.539361	1.14433372	0.19450785	0.17148533	0.11293459	0.25453854
ENSCGRG00001016805	2598.28613	-1.0106208	-0.0152418	0.07282113	0.82434009	0.90517715
ENSCGRG00001016808	2.5782542	-1.0199034	-0.0284324	0.21666054	0.28059644	0.47627407
ENSCGRG00001016817	74.213618	-1.1324727	-0.1794763	0.21491932	0.15004738	0.31147415
ENSCGRG00001016819	344.049339	-1.0183734	-0.0262666	0.12910132	0.79875811	0.88851089
ENSCGRG00001016822	1688.23873	-1.0525447	-0.0738816	0.06666892	0.2425826	0.43255611
ENSCGRG00001016847	513.641067	1.1387067	0.1873962	0.12281462	0.06960856	0.17862294
ENSCGRG00001016873	1952.7734	-1.1434012	-0.1933317	0.06833877	0.00275399	0.01366331
ENSCGRG00001016879	2.35560118	1.00120634	0.00173934	0.21192563	0.9651798	0.98298245
ENSCGRG00001016901	5.92073664	1.04164285	0.05886071	0.22116553	0.2365668	0.42605621
ENSCGRG00001016904	142.390871	1.20418755	0.26806011	0.18281441	0.04163347	0.12123772
ENSCGRG00001016926	385.833475	-1.0856064	-0.1185011	0.1447881	0.28371335	0.47984791

ENSCGRG00001016927	18.1807059	-1.2407134	-0.3111699	0.52839378	0.02236877	0.07508881
ENSCGRG00001016928	26.7356597	1.18061302	0.23953615	0.31740524	0.06486217	0.17015858
ENSCGRG00001016941	5.32109057	-1.0522206	-0.0734372	0.22936997	0.10171781	0.23613722
ENSCGRG00001016962	111.057788	-1.053782	-0.0755764	0.1569942	0.49245235	0.67949091
ENSCGRG00001016964	262.35373	-1.1353356	-0.1831188	0.16075365	0.12229562	0.26932482
ENSCGRG00001016984	2669.99761	-1.1135762	-0.1552003	0.05322444	0.00254459	0.01280288
ENSCGRG00001016997	1452.67672	1.02156909	0.03078678	0.08802928	0.70194999	0.83028433
ENSCGRG00001016998	121.905608	-1.1660377	-0.2216145	0.22999827	0.09575303	0.22585091
ENSCGRG00001017005	3978.4671	-1.1680534	-0.2241063	0.06412319	0.00023694	0.00172158
ENSCGRG00001017009	754.634772	1.08082528	0.11213332	0.07562423	0.11242458	0.25372778
ENSCGRG00001017012	963.631524	1.15260701	0.2049007	0.07692197	0.00430451	0.01959515
ENSCGRG00001017023	190.330173	1.10827828	0.14832018	0.14575022	0.18806562	0.36584976
ENSCGRG00001017031	3.66611136	-1.0136815	-0.0196045	0.21392996	0.56427353	0.73589476
ENSCGRG00001017036	746.649551	-1.1673844	-0.2232797	0.08956142	0.00624379	0.02644065
ENSCGRG00001017051	248.633738	-1.0513157	-0.072196	0.11648227	0.46022638	0.65424127
ENSCGRG00001017068	820.396852	-1.0041864	-0.0060271	0.08694196	0.94221687	0.97189769
ENSCGRG00001017069	503.982326	1.06944784	0.09686612	0.09771661	0.26547636	0.45937486
ENSCGRG00001017076	5635.48606	-1.0403601	-0.057083	0.05577659	0.28912399	0.48566462
ENSCGRG00001017077	62.1244136	-1.0130072	-0.0186444	0.16797162	0.85816821	0.92418115
ENSCGRG00001017092	10.1824307	1.01269746	0.01820324	0.20737494	0.76083359	0.86754436
ENSCGRG00001017097	504.345788	1.03833617	0.05427361	0.09419586	0.52219919	0.70431747
ENSCGRG00001017103	6.85808603	1.06463717	0.09036184	0.23307249	0.15101745	0.31293005
ENSCGRG00001017109	3.88198733	1.03480342	0.04935673	0.21852826	0.28572961	0.48217577
ENSCGRG00001017126	3.10238259	-1.0155309	-0.0222342	0.21498473	0.47003545	0.66188349
ENSCGRG00001017143	722.875238	1.15617082	0.20935457	0.09582296	0.01497805	0.05446177
ENSCGRG00001017155	164.899993	-1.5900878	-0.6691064	0.20985877	0.00011701	0.00093685
ENSCGRG00001017157	12.7368671	-1.0474094	-0.0668255	0.21783378	0.32171265	0.52060877
ENSCGRG00001017162	36.7811054	1.12806846	0.17385462	0.25446935	0.12897465	0.27970309
ENSCGRG00001017170	2366.59891	1.08151279	0.11305073	0.0601236	0.04961177	0.13833389
ENSCGRG00001017197	973.542265	-1.0054051	-0.0077769	0.07348875	0.90970621	0.95292637
ENSCGRG00001017205	557.207267	-1.1257792	-0.1709239	0.09527556	0.04538984	0.12955522
ENSCGRG00001017212	35.8115876	-2.5105823	-1.328022	0.39227967	3.29E-05	0.00030028
ENSCGRG00001017214	12.6515966	1.03918144	0.05544756	0.21241905	0.42608726	0.62450547
ENSCGRG00001017215	276.306303	1.00908757	0.01305138	0.1355411	0.90267035	0.94945197
ENSCGRG00001017223	438.461213	1.2669089	0.34131279	0.17452413	0.01102194	0.04250182
ENSCGRG00001017227	38.6478912	1.10767199	0.14753073	0.2360269	0.17457074	0.3478381
ENSCGRG00001017233	348.072081	1.07684943	0.10681653	0.12124654	0.29015969	0.48663861
ENSCGRG00001017236	893.388594	-1.0205227	-0.0293083	0.11155488	0.75770444	0.86561668
ENSCGRG00001017238	3956.679	-1.0739606	-0.1029411	0.17678846	0.36292576	0.56379809
ENSCGRG00001017239	429.984737	-1.6161019	-0.6925182	0.36187143	0.00304524	0.01477073
ENSCGRG00001017243	9024.55735	-1.0396312	-0.0560719	0.04999628	0.25214348	0.44337103
ENSCGRG00001017247	10.2122554	-1.0689886	-0.0962465	0.23355707	0.16370959	0.33156046
ENSCGRG00001017251	2423.74035	-1.1848002	-0.2446438	0.07563147	0.00054938	0.00353822
ENSCGRG00001017262	459.591786	-1.1007708	-0.1385141	0.09846576	0.11324054	0.25498528
ENSCGRG00001017267	4921.22435	-1.0215168	-0.0307129	0.0554832	0.56640918	0.73729312
ENSCGRG00001017269	254.231675	1.07127744	0.09933216	0.12749617	0.33887589	0.53850274
ENSCGRG00001017298	1118.62344	1.09023156	0.12463459	0.07972878	0.09165301	0.21900299
ENSCGRG00001017302	145.596829	1.06639293	0.09273913	0.17906764	0.40750178	0.60672593
ENSCGRG00001017304	14.7239265	1.03069123	0.0436122	0.20840827	0.53092603	0.71104678
ENSCGRG00001017312	336.257513	-1.1548999	-0.2077679	0.13243561	0.05492184	0.14924667

ENSCGRG00001017313	128.405892	1.11565043	0.15788506	0.16371938	0.18174331	0.35773382
ENSCGRG00001017315	2261.11652	-1.1107511	-0.1515355	0.08246234	0.045728	0.1302882
ENSCGRG00001017316	2308.22424	-1.1262206	-0.1714894	0.06790696	0.0074322	0.03058353
ENSCGRG00001017322	5.39282466	-1.0044881	-0.0064604	0.2123005	0.85683304	0.9236751
ENSCGRG00001017325	7861.50978	-1.0477573	-0.0673046	0.05466795	0.20156215	0.3840564
ENSCGRG00001017361	501.261367	1.07879448	0.10942005	0.14031127	0.31510658	0.51423025
ENSCGRG00001017364	580.561024	1.41256864	0.49832098	0.1100285	8.94E-07	1.17E-05
ENSCGRG00001017367	693.757516	-1.0638082	-0.0892381	0.0875476	0.26243691	0.45528396
ENSCGRG00001017370	13.7461529	-1.0102326	-0.0146874	0.20126687	0.83873171	0.91371188
ENSCGRG00001017373	242.629385	-1.0428874	-0.0605834	0.13659243	0.56746674	0.73799482
ENSCGRG00001017374	316.174276	-1.0427492	-0.0603922	0.14294714	0.57259647	0.74125145
ENSCGRG00001017407	980.198538	1.05201895	0.07316069	0.0680849	0.25137437	0.44268706
ENSCGRG00001017419	5761.86136	1.09621728	0.13253379	0.04968626	0.00588766	0.02520957
ENSCGRG00001017420	103.947444	-1.351415	-0.4344708	0.251159	0.00991231	0.03897381
ENSCGRG00001017428	891.772113	1.12984416	0.1761238	0.08458801	0.02318728	0.07719049
ENSCGRG00001017457	1062.49743	-1.1494723	-0.2009717	0.0918517	0.01566424	0.05647593
ENSCGRG00001017462	37027.7234	-1.1384328	-0.1870491	0.07579905	0.01069461	0.04153972
ENSCGRG00001017501	429.486396	1.03861588	0.05466219	0.09584247	0.52991322	0.71058185
ENSCGRG00001017505	948.659445	-1.3479499	-0.4307669	0.08154036	2.45E-08	4.22E-07
ENSCGRG00001017507	4592.05378	-1.0174381	-0.0249411	0.06015696	0.66532266	0.80639448
ENSCGRG00001017511	27174.443	-1.0637882	-0.0892109	0.05439623	0.09098518	0.21785424
ENSCGRG00001017517	436.818356	1.0918107	0.12672275	0.11475958	0.19446444	0.37431848
ENSCGRG00001017525	388.937536	-1.1803827	-0.2392547	0.15203199	0.04280678	0.12395002
ENSCGRG00001017527	197.341609	-1.1521638	-0.2043458	0.1574316	0.08513547	0.20750296
ENSCGRG00001017546	32.7379211	1.12766466	0.17333811	0.2713151	0.10215552	0.23689577
ENSCGRG00001017575	22.1823772	1.14032525	0.18944538	0.28454079	0.09123933	0.21829956
ENSCGRG00001017591	3.01210032	-1.0098395	-0.014126	0.21372826	0.65822069	0.80173121
ENSCGRG00001017610	1502.99384	-1.1318033	-0.1786233	0.06579849	0.00421908	0.01932989
ENSCGRG00001017631	184.266517	-1.0601811	-0.0843107	0.13497178	0.42819635	0.62608502
ENSCGRG00001017654	3.87032204	-1.018143	-0.0259402	0.21285448	0.55346926	0.72919276
ENSCGRG00001017671	3989.86907	1.08441423	0.11691595	0.062248	0.049004	0.13717547
ENSCGRG00001017673	16917.5106	-1.0433302	-0.0611958	0.04355936	0.15170804	0.31390274
ENSCGRG00001017694	454.625973	-1.1438639	-0.1939154	0.23147894	0.12506444	0.27395272
ENSCGRG00001017700	1512.92098	-1.1912474	-0.2524731	0.07019914	0.00014355	0.00111514
ENSCGRG00001017707	2.31022093	1.00318423	0.00458658	0.21267527	0.89395404	0.94445784
ENSCGRG00001017709	9679.84156	-1.2167355	-0.2830155	0.050887	9.52E-09	1.74E-07
ENSCGRG00001017730	1582.83236	-1.0667641	-0.0932412	0.1131612	0.33323663	0.53306209
ENSCGRG00001017751	1.95945379	1.01613194	0.02308774	0.2148384	0.4681919	0.66059458
ENSCGRG00001017764	6389.55768	1.0829812	0.1150082	0.05200219	0.02261907	0.07571043
ENSCGRG00001017772	545.816062	1.24750085	0.31904079	0.1275862	0.00339292	0.01616337
ENSCGRG00001017811	11.4099255	-1.1095975	-0.1500364	0.27552851	0.06512703	0.17049042
ENSCGRG00001017817	1356.44869	-1.0043659	-0.0062849	0.1052224	0.94120918	0.97139497
ENSCGRG00001017818	4.99580432	-1.0023322	-0.0033607	0.21250669	0.92094877	0.95921243
ENSCGRG00001017824	98.0692894	-1.1609578	-0.2153156	0.19648429	0.09549091	0.22552333
ENSCGRG00001017831	104.04246	1.36446866	0.44833926	0.22345822	0.00558773	0.02419839
ENSCGRG00001017840	712.196571	1.12919755	0.1752979	0.11234523	0.06978782	0.17885163
ENSCGRG00001017855	9485.68052	1.08001468	0.11105092	0.14141041	0.3004098	0.49815528
ENSCGRG00001017863	296.614009	-1.450145	-0.5361971	0.15786101	7.97E-05	0.00066696
ENSCGRG00001017886	11.6188974	-1.0277628	-0.0395073	0.20854782	0.55361088	0.72930434
ENSCGRG00001017909	369.295851	1.04157858	0.05877169	0.10754831	0.52856714	0.70937089

ENSCGRG00001017927	3672.08896	-2.5659503	-1.3594932	0.09282287	8.63E-50	2.40E-47
ENSCGRG00001017938	866.060002	1.00002641	3.81E-05	0.08026254	0.99969045	0.99984655
ENSCGRG00001017943	645.796807	1.23083536	0.2996378	0.11441848	0.0027868	0.01377945
ENSCGRG00001017957	48.7582562	-1.2151018	-0.2810772	0.27072406	0.05513435	0.1496654
ENSCGRG00001017959	1095.16953	-1.2405776	-0.3110119	0.0764713	1.56E-05	0.00015479
ENSCGRG00001017961	167.495888	-1.0303319	-0.0431092	0.16874303	0.67273813	0.81086907
ENSCGRG00001017965	132.284446	-1.016647	-0.0238189	0.16534703	0.82256227	0.90419748
ENSCGRG00001017983	326.173517	1.06225342	0.08712799	0.13946595	0.4212986	0.61976697
ENSCGRG00001018019	6535.464	-1.1761622	-0.2340871	0.0573735	2.24E-05	0.0002112
ENSCGRG00001018024	13.8061622	1.05012974	0.07056759	0.21444534	0.36082762	0.5610541
ENSCGRG00001018034	9656.99434	-1.4396067	-0.5256747	0.08399214	4.83E-11	1.26E-09
ENSCGRG00001018041	1543.96103	1.02901629	0.04126582	0.08036641	0.58035938	0.74680766
ENSCGRG00001018045	63.7057537	-1.7636719	-0.8185822	0.32881606	0.00069431	0.00428422
ENSCGRG00001018048	89.7069474	-1.1797016	-0.238422	0.20095136	0.0727098	0.18418281
ENSCGRG00001018051	1357.37779	-1.0570234	-0.0800073	0.08603557	0.30889678	0.50723853
ENSCGRG00001018057	3716.36383	-1.0380895	-0.0539309	0.05986745	0.34752464	0.54717191
ENSCGRG00001018069	61.9988497	-1.0446358	-0.0630001	0.17648084	0.55613817	0.73075495
ENSCGRG00001018083	350.235846	-1.8878375	-0.9167346	0.15118989	1.01E-10	2.53E-09
ENSCGRG00001018112	387.016086	1.05122903	0.07207702	0.10569798	0.43413394	0.63181729
ENSCGRG00001018118	2737.25879	-1.1662341	-0.2218574	0.06862153	0.00062998	0.00395008
ENSCGRG00001018129	1539.08517	1.0287787	0.04093268	0.08108048	0.58635063	0.74996795
ENSCGRG00001018137	852.035651	-1.0386852	-0.0547585	0.08142842	0.46699991	0.6597848
ENSCGRG00001018151	29453.1086	1.29177102	0.36935036	0.1538115	0.0033453	0.01598408
ENSCGRG00001018154	1415.62586	-1.0058856	-0.0084662	0.08730435	0.91474331	0.9555461
ENSCGRG00001018176	22.6407181	-1.5617175	-0.6431336	0.81543125	0.01052402	0.04096404
ENSCGRG00001018185	3326.08111	1.10014232	0.13769017	0.06329239	0.02239524	0.07511994
ENSCGRG00001018191	6593.01076	-1.1501329	-0.2018005	0.06329326	0.00082734	0.00497801
ENSCGRG00001018213	403.73789	-1.1775679	-0.2358103	0.12953424	0.02828508	0.08937639
ENSCGRG00001018251	989.844193	-1.1364748	-0.1845657	0.08889632	0.0223044	0.07499195
ENSCGRG00001018287	65.6945037	1.04245253	0.05998169	0.18721419	0.55809636	0.73205144
ENSCGRG00001018293	8465.91834	-1.0127593	-0.0182914	0.06676798	0.77649619	0.87544229
ENSCGRG00001018310	489.038535	-1.0889357	-0.1229188	0.09666305	0.15350814	0.31640213
ENSCGRG00001018311	275.353089	-1.0943974	-0.1301367	0.11825388	0.19098577	0.369648
ENSCGRG00001018326	244.371715	-1.065893	-0.0920626	0.14195922	0.39667195	0.59710548
ENSCGRG00001018331	2293.33986	1.02964676	0.04214948	0.07442336	0.54630369	0.72332988
ENSCGRG00001018339	90.4172511	-1.0002969	-0.0004282	0.16427446	0.99535412	0.99813552
ENSCGRG00001018342	1079.7231	-1.164428	-0.2196214	0.09654197	0.0112157	0.04311918
ENSCGRG00001018378	248.823936	-1.0864874	-0.1196715	0.121251	0.23465957	0.42454769
ENSCGRG00001018389	212.595497	1.13481824	0.18246125	0.14904871	0.11198013	0.25317302
ENSCGRG00001018394	199.302555	1.09518139	0.13116983	0.14483536	0.23747503	0.42695511
ENSCGRG00001018404	871.996598	-1.0901156	-0.1244812	0.08595801	0.11294278	0.25453854
ENSCGRG00001018409	3150.92948	1.06643606	0.09279747	0.05267056	0.06889398	0.17744994
ENSCGRG00001018415	44.9343878	-1.0772334	-0.1073309	0.20274715	0.31482003	0.5140656
ENSCGRG00001018461	182.374385	-1.1915404	-0.2528279	0.15219201	0.03390463	0.10345839
ENSCGRG00001018475	74.8122078	1.24668248	0.31809407	0.26111048	0.03773335	0.11248876
ENSCGRG00001018494	140.890089	1.07393155	0.10290204	0.15990623	0.36305421	0.56391848
ENSCGRG00001018516	455.81951	1.22448508	0.29217519	0.10271501	0.00151951	0.00824835
ENSCGRG00001018517	175.666169	-1.1092599	-0.1495974	0.17199112	0.20620198	0.39063108
ENSCGRG00001018542	1105.22272	-1.0321638	-0.0456719	0.07635398	0.52159265	0.703771
ENSCGRG00001018547	351.959983	1.01765673	0.025251	0.1204236	0.80109884	0.89003262

ENSCGRG00001018549	3073.66209	-1.1986081	-0.2613601	0.07558032	0.0002287	0.0016731
ENSCGRG00001018554	27.6561827	1.00470205	0.00676772	0.1921882	0.94070188	0.9712702
ENSCGRG00001018578	249.634763	-1.061588	-0.086224	0.22125425	0.27521994	0.47064042
ENSCGRG00001018612	1319.44984	-1.1643658	-0.2195444	0.12119993	0.03228138	0.09945275
ENSCGRG00001018621	88.0740574	1.05128819	0.07215821	0.16192209	0.51617145	0.6996773
ENSCGRG00001018625	12.0307962	-1.051547	-0.0725134	0.22587926	0.18221398	0.35821993
ENSCGRG00001018631	665.383352	1.06557639	0.09163402	0.18160131	0.40805827	0.60710934
ENSCGRG00001018641	7784.22933	1.03395402	0.04817204	0.06103863	0.41433266	0.61317022
ENSCGRG00001018651	21886.4621	-1.0356552	-0.0505438	0.07545401	0.48884158	0.67701206
ENSCGRG00001018658	65.7090947	1.061447	0.08603234	0.18517466	0.42801559	0.6260203
ENSCGRG00001018665	133.422191	-1.2446961	-0.3157936	0.19075857	0.02185902	0.07366851
ENSCGRG00001018670	808.247071	1.08739954	0.12088212	0.09057847	0.14089448	0.29807734
ENSCGRG00001018703	1018.22364	-1.26208	-0.3358034	0.11068032	0.00061989	0.00390212
ENSCGRG00001018714	118.049927	1.25938003	0.33271369	0.20996731	0.02213375	0.07449642
ENSCGRG00001018722	75.8446629	1.26955967	0.3443282	0.28723749	0.03129158	0.09700996
ENSCGRG00001018725	12.4064817	-1.0109988	-0.0157812	0.20700579	0.78837427	0.88132248
ENSCGRG00001018729	24.7863342	-1.07597	-0.1056379	0.23726534	0.15806291	0.32365503
ENSCGRG00001018767	1211.01135	1.03721601	0.05271638	0.07155431	0.43501675	0.63267082
ENSCGRG00001018776	364.763124	1.04274117	0.06038109	0.11634294	0.53936276	0.71806661
ENSCGRG00001018796	8.12672641	-1.0264139	-0.0376126	0.21044453	0.53497096	0.71400063
ENSCGRG00001018825	8.18428498	-1.020487	-0.0292578	0.20946296	0.61186943	0.77162085
ENSCGRG00001018841	3109.80614	-1.1768146	-0.2348871	0.07951839	0.00148926	0.00810425
ENSCGRG00001018845	901.143416	-1.0896436	-0.1238564	0.07352781	0.07204422	0.18300194
ENSCGRG00001018874	292.035684	-1.1216358	-0.1656043	0.12334191	0.10650498	0.24419703
ENSCGRG00001018882	4.34177531	1.01519017	0.02175	0.21332323	0.58222268	0.74804864
ENSCGRG00001018890	2.23515732	-1.0243479	-0.0347057	0.21701245	0.29371805	0.49099937
ENSCGRG00001018894	17239.445	-1.0209424	-0.0299015	0.07457206	0.65608724	0.80065519
ENSCGRG00001018897	41.2670322	1.39909682	0.4844958	0.65148537	0.01543886	0.05577321
ENSCGRG00001018934	19183.1948	-1.0214364	-0.0305994	0.04130862	0.44744769	0.64330021
ENSCGRG00001018967	819.360102	1.19748624	0.26000908	0.09512481	0.00256604	0.01289563
ENSCGRG00001018983	6962.1933	-1.2682152	-0.3427995	0.08920144	3.30E-05	0.00030101
ENSCGRG00001019002	614.78973	1.11075596	0.15154189	0.09882901	0.08427678	0.20583136
ENSCGRG00001019027	49.5564036	1.2559376	0.32876478	0.3378071	0.03805003	0.11316948
ENSCGRG00001019088	256.747325	-1.0581557	-0.0815519	0.13597648	0.44454009	0.64099039
ENSCGRG00001019093	187.982111	-1.1305886	-0.1770741	0.14514643	0.11673828	0.26061649
ENSCGRG00001019101	8897.26467	-1.0316374	-0.0449359	0.07575138	0.52281792	0.70505291
ENSCGRG00001019129	567.830903	-2.5712047	-1.3624445	0.29149828	1.48E-07	2.22E-06
ENSCGRG00001019143	127.935466	-1.0775041	-0.1076933	0.15380008	0.3377119	0.53733566
ENSCGRG00001019147	627.138393	1.0240072	0.03422586	0.10673862	0.71155546	0.83708563
ENSCGRG00001019169	2239.91703	-1.1089308	-0.1491693	0.06830809	0.02064213	0.07037821
ENSCGRG00001019175	12793.0249	-1.331688	-0.4132561	0.04748719	6.99E-19	3.90E-17
ENSCGRG00001019185	5.98831596	-1.0201226	-0.0287425	0.21020901	0.60642408	0.76723877
ENSCGRG00001019189	13.3335955	-1.0338039	-0.0479626	0.20653407	0.52530526	0.70684458
ENSCGRG00001019203	2.02801872	1.00294923	0.00424858	0.21300242	0.89732388	0.94630105
ENSCGRG00001019220	209.294007	-1.3121284	-0.3919089	0.17547113	0.00447389	0.0202439
ENSCGRG00001019222	54.8804913	-1.0036182	-0.0052106	0.18317685	0.95531948	0.97846346
ENSCGRG00001019224	213.527136	1.08248362	0.1143452	0.13339522	0.28305799	0.47911903
ENSCGRG00001019229	1263.10296	-1.0268283	-0.038195	0.1062011	0.6861587	0.8196191
ENSCGRG00001019242	8.78279105	1.00493564	0.00710311	0.20460148	0.91390415	0.95543627
ENSCGRG00001019257	11752.7722	-1.0783545	-0.1088316	0.08038088	0.14282033	0.30105784

ENSCGRG00001019269	2.88235909	1.01600093	0.02290173	0.21480428	0.4786644	0.66910639
ENSCGRG00001019270	43.9997079	-1.1014227	-0.1393682	0.23308027	0.18719581	0.36469608
ENSCGRG00001019284	357.793402	1.10697053	0.14661682	0.1251767	0.15623877	0.32092634
ENSCGRG00001019286	94.5878447	1.01098802	0.0157659	0.16046636	0.88417786	0.93784205
ENSCGRG00001019287	276.20483	-1.014407	-0.0206366	0.12552956	0.83868826	0.91371188
ENSCGRG00001019290	3.35363928	-1.008707	-0.0125071	0.21592896	0.12372644	0.27203629
ENSCGRG00001019293	2992.58087	1.01564028	0.02238951	0.08829173	0.7794111	0.87673482
ENSCGRG00001019304	50.5342204	-1.4722764	-0.5580486	0.35376668	0.0075959	0.03109731
ENSCGRG00001019309	3469.91811	1.14372647	0.19374206	0.06864338	0.0028268	0.01393281
ENSCGRG00001019326	1472.01545	-1.0437599	-0.0617898	0.07939485	0.40108768	0.60084279
ENSCGRG00001019331	2102.22985	-1.1212206	-0.1650701	0.0600433	0.004094	0.01883769
ENSCGRG00001019337	610.462842	1.09674669	0.13323036	0.09784324	0.12676197	0.27616
ENSCGRG00001019344	5.07072549	1.02472851	0.03524173	0.2138776	0.46222161	0.65570972
ENSCGRG00001019362	3991.62853	-1.0168326	-0.0240822	0.07978973	0.73451954	0.85147553
ENSCGRG00001019367	447.214489	1.17257581	0.2296812	0.10794325	0.01519356	0.05510461
ENSCGRG00001019373	695.466599	1.06858915	0.09570727	0.10256282	0.28885699	0.48540707
ENSCGRG00001019410	2240.49739	1.5351122	0.61834411	0.05719321	3.57E-28	3.39E-26
ENSCGRG00001019416	225.728216	1.41849877	0.5043649	0.15919417	0.00020452	0.00152497
ENSCGRG00001019419	4922.61122	-1.0222815	-0.0317925	0.0646248	0.56522133	0.73652845
ENSCGRG00001019427	111.49617	3.82418028	1.93515054	0.26394073	8.80E-15	3.41E-13
ENSCGRG00001019431	604.467636	-1.2597682	-0.3331583	0.12337756	0.00180732	0.00959064
ENSCGRG00001019444	108.892093	1.07167277	0.09986446	0.17963084	0.37748334	0.57950034
ENSCGRG00001019446	10115.8053	-1.1469919	-0.1978551	0.07292965	0.00374725	0.01753415
ENSCGRG00001019452	887.242574	-1.1207679	-0.1644875	0.08288446	0.03081359	0.0958296
ENSCGRG00001019454	14045.7608	1.0085345	0.01226044	0.08897373	0.88262616	0.93698481
ENSCGRG00001019466	116.473741	-1.0419192	-0.0592434	0.16204981	0.58827109	0.75184602
ENSCGRG00001019489	108.621066	-1.1539636	-0.2065977	0.18621547	0.10295496	0.23845805
ENSCGRG00001019504	5649.99503	-1.020678	-0.0295278	0.04870961	0.53408938	0.71326991
ENSCGRG00001019512	15905.4419	-1.1352746	-0.1830413	0.0672973	0.00408568	0.01882648
ENSCGRG00001019518	870.644638	-1.0930388	-0.1283446	0.10095616	0.14991762	0.31130567
ENSCGRG00001019520	9229.78901	-1.1960971	-0.2583346	0.05280225	4.44E-07	6.08E-06
ENSCGRG00001019521	140.070839	-1.0947388	-0.1305867	0.14665338	0.24082595	0.43044237
ENSCGRG00001019532	147.21659	-1.0622502	-0.0871236	0.1593762	0.43407817	0.63180791
ENSCGRG00001019533	17211.5027	-1.0394138	-0.0557701	0.08297347	0.49640229	0.68272636
ENSCGRG00001019537	23.33297	-1.1331947	-0.1803957	0.29628613	0.06615434	0.17234841
ENSCGRG00001019541	3880.27378	1.18528964	0.24523964	0.0704892	0.00023159	0.0016904
ENSCGRG00001019557	8.16564959	-1.0037707	-0.0054298	0.20882029	0.91327719	0.95523619
ENSCGRG00001019564	202.633347	1.01608648	0.0230232	0.13468686	0.82866343	0.90788335
ENSCGRG00001019579	69.1813418	1.03216864	0.0456787	0.16544265	0.67304999	0.81100276
ENSCGRG00001019619	9.24715257	-1.0143132	-0.0205031	0.20721517	0.73495732	0.85171009
ENSCGRG00001019655	405.317365	-1.2302377	-0.2989371	0.10998924	0.0020968	0.0108263
ENSCGRG00001019662	1520.84358	-1.018162	-0.0259671	0.06664452	0.68142715	0.81656518
ENSCGRG00001019665	984.541542	-1.0951272	-0.1310984	0.11761131	0.18514537	0.36220407
ENSCGRG00001019675	4.94430158	-1.027511	-0.0391539	0.21430436	0.43720153	0.63469534
ENSCGRG00001019682	4.49942982	-1.0579413	-0.0812596	0.236242	0.02437755	0.08023548
ENSCGRG00001019698	58.2832948	-1.1033675	-0.1419134	0.20788309	0.22283131	0.41118322
ENSCGRG00001019699	1000.79247	1.01974604	0.0282099	0.09174419	0.73413158	0.85136932
ENSCGRG00001019717	27.6926849	-1.0716868	-0.0998833	0.21969484	0.2741067	0.46955159
ENSCGRG00001019724	1468.49498	-1.0626059	-0.0876066	0.17146715	0.42902219	0.62679907
ENSCGRG00001019730	101.664821	1.17634327	0.23430911	0.216946	0.08223562	0.20188545

ENSCGRG00001019733	84.6252259	1.0566801	0.07953868	0.1871235	0.45517731	0.64971571
ENSCGRG00001019737	1588.13949	-1.0394989	-0.0558882	0.06715423	0.3801272	0.58211948
ENSCGRG00001019743	658.305332	-1.2302895	-0.2989978	0.10840797	0.00186374	0.00983705
ENSCGRG00001019746	3285.53715	-1.0912514	-0.1259835	0.06264724	0.03478	0.10550126
ENSCGRG00001019774	2.42405072	-1.024368	-0.0347341	0.21788204	0.21886458	0.40578234
ENSCGRG00001019778	960.463958	1.02356297	0.03359986	0.10985898	0.7234752	0.84405439
ENSCGRG00001019779	1506.87463	1.01359232	0.0194775	0.10029441	0.82733926	0.9074592
ENSCGRG00001019781	4382.66379	-1.1315104	-0.1782499	0.08242337	0.01884065	0.06528232
ENSCGRG00001019803	2499.27069	-1.1842785	-0.2440084	0.06543822	9.00E-05	0.00074015
ENSCGRG00001019810	3.8499147	-1.0043563	-0.0062712	0.21300118	0.84511191	0.91643812
ENSCGRG00001019811	241.10672	-1.3980059	-0.4833705	0.18134168	0.00096677	0.00567828
ENSCGRG00001019814	3168.0847	1.06233932	0.08724465	0.06551483	0.16142013	0.32801267
ENSCGRG00001019837	302.785896	1.07044526	0.09821103	0.11512326	0.31512158	0.51423025
ENSCGRG00001019891	828.106496	-1.0197797	-0.0282575	0.07834389	0.69810525	0.82770938
ENSCGRG00001019909	920.520441	-1.0639254	-0.0893969	0.07378818	0.19587663	0.37635813
ENSCGRG00001019911	838.14333	-1.098692	-0.135787	0.08652204	0.08574346	0.20845962
ENSCGRG00001019917	250.085141	1.16406444	0.21917092	0.14576931	0.05701168	0.15368678
ENSCGRG00001019946	78.8615628	-1.9883223	-0.9915516	0.39697346	0.00058104	0.00370123
ENSCGRG00001019961	60.6468724	-1.0451886	-0.0637633	0.17701718	0.55146124	0.72774477
ENSCGRG00001020006	1694.39493	1.16134505	0.21579668	0.07011585	0.00111944	0.00640178
ENSCGRG00001020011	166.454143	-1.5152506	-0.5995564	0.19708345	0.00022081	0.0016275
ENSCGRG00001020018	755.273118	1.10450146	0.14339533	0.10772057	0.12570718	0.27465615
ENSCGRG00001020021	8547.85754	-1.0415979	-0.0587984	0.0647029	0.33965337	0.53919807
ENSCGRG00001020025	945.288999	1.00000963	1.39E-05	0.091247	0.99988398	0.9999422
ENSCGRG00001020028	628.911296	1.01306079	0.01872075	0.12114364	0.85294693	0.92095838
ENSCGRG00001020030	101.304603	-1.0897043	-0.1239368	0.16807183	0.28351513	0.47970266
ENSCGRG00001020054	106.03435	1.35578324	0.43912654	0.27297679	0.01202889	0.0456211
ENSCGRG00001020059	42.3785592	1.04418111	0.06237196	0.19714609	0.5117943	0.69590117
ENSCGRG00001020073	33.8538279	-1.1297175	-0.1759621	0.2847577	0.08079984	0.19943512
ENSCGRG00001020085	15.7354446	-1.0684939	-0.0955787	0.2265999	0.23258632	0.42189616
ENSCGRG00001020097	1462.11013	-1.2550029	-0.3276906	0.06963188	7.47E-07	9.87E-06
ENSCGRG00001020112	1259.81291	-1.0662937	-0.0926049	0.08370205	0.22774752	0.4163616
ENSCGRG00001020113	368.629744	-1.2790552	-0.3550785	0.12773192	0.00127085	0.00707499
ENSCGRG00001020124	238.193937	-1.1024337	-0.140692	0.15194583	0.21529556	0.40150476
ENSCGRG00001020137	752.255583	-1.2988118	-0.3771924	0.13504264	0.0010813	0.00622537
ENSCGRG00001020141	3.01583633	-1.0087425	-0.012558	0.21278381	0.72952186	0.84829649
ENSCGRG00001020148	124.78104	1.00961126	0.01379991	0.15958845	0.89892392	0.94744244
ENSCGRG00001020185	6950.9907	-1.0421881	-0.0596157	0.05683091	0.27623102	0.47184932
ENSCGRG00001020215	477.117683	-1.0296269	-0.0421217	0.10908444	0.63968052	0.79058554
ENSCGRG00001020227	5194.28127	-1.0086284	-0.0123947	0.06496593	0.83500423	0.91171934
ENSCGRG00001020233	285.058281	1.09676018	0.13324809	0.11431813	0.1717997	0.34376041
ENSCGRG00001020238	11376.2256	-1.1154523	-0.1576288	0.06852933	0.01493996	0.05436956
ENSCGRG00001020246	3376.96505	1.00350181	0.00504321	0.09508354	0.95802948	0.9800637
ENSCGRG00001020247	15.9653869	-1.0014407	-0.0020769	0.2037749	0.9726299	0.98767978
ENSCGRG00001020256	779.982813	-1.0035336	-0.005089	0.08904994	0.94958422	0.97492778
ENSCGRG00001020270	61.2675459	-1.1411492	-0.1904874	0.22017886	0.13194393	0.28397206
ENSCGRG00001020271	103.318506	1.12888163	0.17489422	0.20612244	0.15946378	0.32567047
ENSCGRG00001020272	3.47270422	-1.016461	-0.0235549	0.21274308	0.58339842	0.74830245
ENSCGRG00001020273	2.23701585	-1.0300447	-0.042707	0.21972892	0.1489676	0.3098855
ENSCGRG00001020289	608.696003	1.00028615	0.00041277	0.10772532	0.99894967	0.99959215

ENSCGRG00001020299	24754.5204	1.07399274	0.10298424	0.04404757	0.01811283	0.06344689
ENSCGRG00001020308	716.657682	-1.2459415	-0.3172363	0.09734563	0.00033739	0.00230625
ENSCGRG00001020315	3494.24765	1.04024709	0.05692626	0.05624165	0.29428363	0.49157037
ENSCGRG00001020325	45.0204914	1.03876164	0.05486464	0.19421615	0.56557588	0.73677912
ENSCGRG00001020329	11.4107525	-1.0608364	-0.0852022	0.23345922	0.11181465	0.2528859
ENSCGRG00001020346	1152.60161	1.02653634	0.03778471	0.07679663	0.59871723	0.76170104
ENSCGRG00001020377	1790.57303	-1.1939212	-0.2557076	0.07514447	0.00028592	0.00200909
ENSCGRG00001020388	2070.41455	-1.0970476	-0.1336261	0.06296102	0.02590538	0.08382115
ENSCGRG00001020399	59.3793788	-1.0306596	-0.043568	0.17921233	0.67084221	0.81004995
ENSCGRG00001020427	537.793319	1.03071912	0.04365124	0.10748866	0.64050169	0.79068702
ENSCGRG00001020434	782.025926	3.00678776	1.58822304	0.31727765	2.68E-08	4.58E-07
ENSCGRG00001020438	3523.94036	-1.1474582	-0.1984416	0.09730315	0.0222897	0.07496222
ENSCGRG00001020475	37.3702102	-1.0140252	-0.0200936	0.18641555	0.82965009	0.90821835
ENSCGRG00001020483	12.5748657	-1.0868633	-0.1201704	0.25369238	0.07838457	0.19519952
ENSCGRG00001020484	2831.29393	-1.0357034	-0.0506109	0.05056289	0.30323223	0.50095498
ENSCGRG00001020486	423.269082	-1.1979662	-0.2605872	0.10466901	0.00502709	0.02224421
ENSCGRG00001020487	10.0277874	-1.0069837	-0.0100403	0.20752268	0.85772645	0.92418115
ENSCGRG00001020496	546.826251	-1.4536032	-0.5396335	0.13321819	6.41E-06	6.89E-05
ENSCGRG00001020498	606.898453	-1.203614	-0.2673727	0.09825053	0.00251513	0.01266464
ENSCGRG00001020502	85.995058	-1.0453046	-0.0639234	0.16620686	0.5546222	0.73003601
ENSCGRG00001020504	125.719127	1.14882564	0.20015985	0.18781364	0.11364547	0.25558259
ENSCGRG00001020524	500.376546	-1.048226	-0.0679498	0.10619242	0.46157464	0.65522729
ENSCGRG00001020536	1171.15725	-1.2231984	-0.2906585	0.09662526	0.00089437	0.00532383
ENSCGRG00001020539	1174.39133	1.46212256	0.54806425	0.0992349	4.45E-09	8.59E-08
ENSCGRG00001020543	20.1593809	1.03520819	0.04992093	0.20682422	0.51595789	0.69948361
ENSCGRG00001020552	2.67591834	1.02786226	0.03964694	0.21991544	0.09170263	0.2190807
ENSCGRG00001020592	458.359868	-1.0333124	-0.0472765	0.10416894	0.60319473	0.76496629
ENSCGRG00001020617	4617.17096	-1.0122522	-0.0175688	0.09264313	0.8325545	0.91007181
ENSCGRG00001020641	102.457512	-1.4221235	-0.5080468	0.30083049	0.00753359	0.03089158
ENSCGRG00001020661	4662.26148	-1.5288234	-0.6124218	0.07239009	3.10E-18	1.63E-16
ENSCGRG00001020697	3985.59835	1.00388579	0.00559514	0.13239833	0.93575124	0.96890674
ENSCGRG00001020749	57.2190982	1.0444972	0.06280862	0.19806908	0.50548431	0.6898108
ENSCGRG00001020756	778.171703	1.11934374	0.16265314	0.08715575	0.04085442	0.11962175
ENSCGRG00001020757	222.630384	1.00287322	0.00413924	0.14396092	0.96927197	0.9853483
ENSCGRG00001020761	5.71697353	1.04614965	0.06508924	0.22365598	0.19885412	0.37997035
ENSCGRG00001020786	31.0994371	1.01226347	0.01758485	0.19485577	0.83719843	0.91272442
ENSCGRG00001020796	3.30313979	1.00190998	0.00275289	0.21240981	0.93925519	0.97057563
ENSCGRG00001020833	248.98599	-1.0596429	-0.0835781	0.11775512	0.39814513	0.59826851
ENSCGRG00001020861	232.648135	1.099869	0.1373317	0.1383465	0.20883025	0.39415287
ENSCGRG00001020862	231.517732	1.01060157	0.01521433	0.13101324	0.88496755	0.93818984
ENSCGRG00001020881	376.063044	-1.1354904	-0.1833155	0.11714656	0.06507283	0.17046015
ENSCGRG00001020899	7.99574719	-1.010392	-0.0149151	0.21038622	0.75328635	0.86298066
ENSCGRG00001020900	5089.96425	-1.0193523	-0.0276528	0.06161131	0.64077694	0.79070924
ENSCGRG00001020932	210.005869	-1.0302481	-0.0429918	0.12652575	0.67194408	0.81086907
ENSCGRG00001020944	1199.20023	1.02146465	0.03063928	0.07293567	0.65588685	0.8004869
ENSCGRG00001020948	993.555675	1.06484407	0.09064218	0.10752948	0.3315262	0.53138771
ENSCGRG00001020955	138.107559	1.05850335	0.08202584	0.16730773	0.46393577	0.65728516
ENSCGRG00001020965	12131.7202	-1.0658459	-0.0919989	0.06330572	0.1276883	0.27770579
ENSCGRG00001020969	1936.99888	1.05859102	0.08214532	0.15669278	0.46180419	0.65533529
ENSCGRG00001020987	1537.1806	-1.0769423	-0.1069409	0.06759439	0.0948523	0.22451183

ENSCGRG00001020992	248.835169	1.04809608	0.06777098	0.11661544	0.49098425	0.67827785
ENSCGRG00001021001	58.9831491	1.36608503	0.45004729	0.52089219	0.01893418	0.06555321
ENSCGRG00001021004	1.61291882	-1.0049061	-0.0070607	0.21361017	0.80505406	0.89249178
ENSCGRG00001021011	747.312225	-1.0056652	-0.00815	0.09606838	0.92391109	0.96109284
ENSCGRG00001021012	416.039003	1.03611	0.05117717	0.10798214	0.58918016	0.75240733
ENSCGRG00001021014	29.7898288	1.04075269	0.05762729	0.20650903	0.47778878	0.668247
ENSCGRG00001021021	80.8870208	1.00187928	0.00270868	0.17413125	0.98076106	0.99088655
ENSCGRG00001021035	4501.51187	-1.0818146	-0.1134533	0.07064643	0.08800037	0.21293629
ENSCGRG00001021036	504.581077	-1.2937355	-0.3715427	0.10609847	0.00010531	0.00085061
ENSCGRG00001021059	4398.9866	1.38924083	0.47429672	0.06342437	1.30E-14	4.94E-13
ENSCGRG00001021101	1579.85086	1.16849153	0.22464728	0.08377816	0.00363764	0.01712537
ENSCGRG00001021110	3.92754117	-1.0578462	-0.0811299	0.23337406	0.08100744	0.19975079
ENSCGRG00001021112	57.1213583	-1.1211617	-0.1649944	0.24155227	0.14999552	0.31141695
ENSCGRG00001021113	824.399704	-1.1669226	-0.2227089	0.09904727	0.01163425	0.04451455
ENSCGRG00001021117	800.551283	1.03316861	0.04707572	0.10521198	0.60858722	0.76890814
ENSCGRG00001021129	843.431401	-1.01932	-0.027607	0.10043993	0.75975905	0.86676548
ENSCGRG00001021131	3082.7527	-1.0246317	-0.0351055	0.0636599	0.56298589	0.73507789
ENSCGRG00001021135	109.090795	1.01372604	0.01966782	0.15189816	0.85603407	0.92312454
ENSCGRG00001021153	3.26258498	1.00541201	0.00778683	0.21093739	0.860857	0.925676
ENSCGRG00001021168	8027.06487	1.01730221	0.02474833	0.12658434	0.81553989	0.90006599
ENSCGRG00001021183	58.141432	-1.5634748	-0.644756	0.33945018	0.00358605	0.01692606
ENSCGRG00001021184	415.605268	1.35601303	0.43937104	0.13963884	0.00027523	0.00194793
ENSCGRG00001021188	2065.64333	-1.0251443	-0.035827	0.15752549	0.73894585	0.85447701
ENSCGRG00001021189	155.831154	-2.3815499	-1.2519008	0.18269362	4.63E-13	1.54E-11
ENSCGRG00001021201	66.9126091	-1.2367483	-0.3065519	0.29362758	0.0440665	0.12665287
ENSCGRG00001021203	4.9086849	-1.0167532	-0.0239696	0.21277071	0.58300042	0.74830245
ENSCGRG00001021211	2.94884604	-1.0344701	-0.048892	0.22116625	0.14270764	0.30091932
ENSCGRG00001021223	534.834799	1.18502551	0.24491812	0.11876612	0.01588384	0.05706321
ENSCGRG00001021236	6.39400036	1.01806214	0.02582563	0.21329918	0.54881906	0.72563893
ENSCGRG00001021244	240.543916	-1.3327603	-0.4144173	0.22344993	0.00871985	0.03496127
ENSCGRG00001021245	71.4710631	-1.0356008	-0.0504679	0.16514539	0.64016948	0.79058554
ENSCGRG00001021246	226.162661	1.05086677	0.07157977	0.17018938	0.51583401	0.6994012
ENSCGRG00001021258	200.183898	-1.035751	-0.0506772	0.1249215	0.61844225	0.77570207
ENSCGRG00001021277	863.081446	1.00419392	0.00603789	0.07819646	0.93467555	0.96834491
ENSCGRG00001021280	78.522785	1.01642199	0.02349949	0.18039481	0.81643796	0.90066916
ENSCGRG00001021296	31.1946253	-1.0296685	-0.0421799	0.19400433	0.64061758	0.79070924
ENSCGRG00001021303	2192.3974	-1.0468609	-0.0660698	0.05821042	0.2372828	0.4267892
ENSCGRG00001021315	941.210981	1.17228603	0.22932462	0.08496332	0.00334399	0.01598376
ENSCGRG00001021316	1312.22255	1.04298317	0.06071588	0.08199409	0.42310759	0.62148931
ENSCGRG00001021329	164.370147	-1.0021736	-0.0031325	0.14875347	0.975478	0.98867578
ENSCGRG00001021333	17850.0814	-1.0185306	-0.0264894	0.06121516	0.66190663	0.8043199
ENSCGRG00001021358	1047.57229	1.0636714	0.08905252	0.08488744	0.25361815	0.44510871
ENSCGRG00001021359	139.11594	-1.0307053	-0.043632	0.20565563	0.55795925	0.73202151
ENSCGRG00001021383	3.83292737	-1.0307662	-0.0437171	0.21765024	0.299954	0.49767745
ENSCGRG00001021393	3.83141517	-1.0068433	-0.0098392	0.21276549	0.77511837	0.87474816
ENSCGRG00001021398	2292.39058	1.34773006	0.43053156	0.08392635	5.68E-08	9.22E-07
ENSCGRG00001021401	2923.14593	1.01370362	0.01963591	0.05107554	0.69276301	0.82432399
ENSCGRG00001021429	539.154276	-1.0825283	-0.1144048	0.10533554	0.21322453	0.39915332
ENSCGRG00001021438	2.63592448	1.02625152	0.03738435	0.21781598	0.25275748	0.44402266
ENSCGRG00001021440	2356.40906	-1.0017426	-0.0025118	0.09538516	0.97545374	0.98867578

ENSCGRG00001021443	1632.9038	-1.2394232	-0.3096688	0.06704602	1.32E-06	1.66E-05
ENSCGRG00001021444	22.0676274	1.09348427	0.12893247	0.23343475	0.19620852	0.37688276
ENSCGRG00001021449	34674.788	-1.0478425	-0.0674219	0.0618501	0.25412489	0.44557074
ENSCGRG00001021457	615.449142	-1.2684214	-0.3430342	0.10286682	0.00022431	0.00164573
ENSCGRG00001021460	1648.95644	-1.0127732	-0.0183112	0.07782331	0.80018872	0.8893487
ENSCGRG00001021480	24.8615001	-1.0149499	-0.0214086	0.19360032	0.80345811	0.89151523
ENSCGRG00001021483	216.503814	-1.1985442	-0.2612831	0.19358669	0.05006895	0.13918908
ENSCGRG00001021491	5.35075503	-1.0047185	-0.0067914	0.21328864	0.82163925	0.90376956
ENSCGRG00001021493	2.21323684	1.00126875	0.00182925	0.21295684	0.95619676	0.97889239
ENSCGRG00001021494	467.702089	1.14489873	0.19521999	0.10221207	0.03094494	0.09614472
ENSCGRG00001021524	12284.7334	-1.3123845	-0.3921905	0.04136442	5.94E-22	4.07E-20
ENSCGRG00001021534	6.41609856	-1.0026328	-0.0037933	0.20662403	0.94675064	0.97373406
ENSCGRG00001021540	635.50767	-1.0623895	-0.0873128	0.11002249	0.35531266	0.55520311
ENSCGRG00001021576	9420.68758	-1.1098024	-0.1503029	0.05317799	0.00344439	0.01637203
ENSCGRG00001021584	4.36343545	1.02533265	0.03609204	0.21573559	0.39314153	0.59416592
ENSCGRG00001021589	7854.72497	-1.1450558	-0.1954179	0.05628485	0.00030531	0.00211751
ENSCGRG00001021591	33.537376	-1.0300999	-0.0427843	0.1902161	0.65145422	0.79782837
ENSCGRG00001021595	3644.36984	1.05070171	0.07135315	0.09255912	0.3926824	0.59396168
ENSCGRG00001021628	1717.0783	-1.0030139	-0.0043416	0.10316617	0.96065563	0.98137498
ENSCGRG00001021653	140.804152	1.24388467	0.31485273	0.20361022	0.02674172	0.08576903
ENSCGRG00001021658	645.377462	-1.0097141	-0.0139469	0.09302822	0.86725605	0.92827122
ENSCGRG00001021667	38.6404958	-1.0132656	-0.0190124	0.18509669	0.84029261	0.91430803
ENSCGRG00001021697	563.81576	1.08460139	0.11716493	0.10438387	0.19966027	0.38128325
ENSCGRG00001021699	501.223706	1.15340167	0.20589502	0.11292768	0.03491205	0.10580159
ENSCGRG00001021702	5269.42117	-1.0717353	-0.0999486	0.06175971	0.08991449	0.21593637
ENSCGRG00001021703	1987.35427	-1.0148752	-0.0213024	0.06154413	0.71743478	0.84141289
ENSCGRG00001021714	90.8951905	-1.5418023	-0.6246178	0.34376868	0.00415326	0.01906928
ENSCGRG00001021718	23.5766569	-1.1306414	-0.1771414	0.27533673	0.09783899	0.22933531
ENSCGRG00001021731	13.1950863	1.00859961	0.01235358	0.20655901	0.83874613	0.91371188
ENSCGRG00001021742	580.720484	1.00758095	0.01089575	0.09098863	0.89559689	0.94539263
ENSCGRG00001021748	1244.50051	-1.0575773	-0.0807632	0.07108026	0.22757594	0.41624424
ENSCGRG00001021751	39.0647082	1.00728063	0.01046568	0.18232652	0.91599729	0.95628315
ENSCGRG00001021756	3.12692892	-1.0406934	-0.0575452	0.22492021	0.06952544	0.1785169
ENSCGRG00001021777	1091.06966	1.00302768	0.00436142	0.07720328	0.95281442	0.977226
ENSCGRG00001021780	2369.97591	-1.0866819	-0.1199296	0.0546565	0.02292164	0.07644524
ENSCGRG00001021795	872.599277	1.0872861	0.12073161	0.0841588	0.11723352	0.26149424
ENSCGRG00001021813	1.81010457	-1.0064123	-0.0092215	0.21361413	0.75418887	0.86317158
ENSCGRG00001021827	3775.29903	1.25573199	0.32852858	0.05533765	9.09E-10	1.94E-08
ENSCGRG00001021853	1801.28594	-1.392144	-0.4773085	0.09603788	1.08E-07	1.67E-06
ENSCGRG00001021864	4367.99358	-1.0395649	-0.0559798	0.04933251	0.24284918	0.4328507
ENSCGRG00001021873	242.691241	-1.105433	-0.1446115	0.13757177	0.18298634	0.35913207
ENSCGRG00001021903	72.7700367	-1.1587556	-0.2125763	0.21845246	0.10418183	0.24046293
ENSCGRG00001021905	2127.49347	-1.0238619	-0.0340211	0.08916238	0.67541531	0.81273255
ENSCGRG00001021912	1244.88788	1.02170317	0.03097612	0.09990951	0.72684996	0.84629594
ENSCGRG00001021917	5919.35789	1.00200267	0.00288636	0.06087677	0.95976315	0.98105115
ENSCGRG00001021929	1382.35975	1.07820781	0.10863526	0.06223071	0.06766265	0.17518348
ENSCGRG00001021945	141.200245	1.30620254	0.38537861	0.26488109	0.01958039	0.06742603
ENSCGRG00001021963	417.82887	-1.0257564	-0.0366881	0.11497797	0.70572101	0.83335971
ENSCGRG00001021992	1184.36629	1.03574538	0.05066939	0.10631984	0.5846896	0.74932628
ENSCGRG00001022014	22.3179774	1.03734469	0.05289535	0.20427341	0.52070868	0.70309668

ENSCGRG00001022032	12.8351332	-1.0389554	-0.0551337	0.21681814	0.34088081	0.54024041
ENSCGRG00001022048	10769.5633	-1.2284984	-0.296896	0.04979984	9.54E-10	2.02E-08
ENSCGRG00001022056	1535.74744	-1.1662797	-0.2219138	0.07875187	0.00243725	0.01235014
ENSCGRG00001022058	417.696201	1.03311247	0.04699732	0.10163771	0.6004802	0.7628832
ENSCGRG00001022067	18419.7145	-1.0563274	-0.0790571	0.04483934	0.07105842	0.18108552
ENSCGRG00001022071	10.0181053	-1.0973179	-0.1339816	0.26493103	0.05973892	0.15949469
ENSCGRG00001022084	2094.46615	-1.1326837	-0.179745	0.09794701	0.03953345	0.11660248
ENSCGRG00001022085	1767.3425	1.18985492	0.25078567	0.08881795	0.00204661	0.0106185
ENSCGRG00001022089	339.019485	1.03670272	0.05200225	0.11623446	0.59660892	0.75952575
ENSCGRG00001022115	58.9142351	-1.1260278	-0.1712425	0.24982119	0.1354982	0.28972324
ENSCGRG00001022131	607.168108	1.11577802	0.15805004	0.12692347	0.13096928	0.28260226
ENSCGRG00001022135	20559.2853	1.06289687	0.08800162	0.07482547	0.19649171	0.37705918
ENSCGRG00001022149	1130.0823	1.18728211	0.24766278	0.07209221	0.00027064	0.00192023
ENSCGRG00001022152	113.726772	1.0453056	0.06392478	0.17741391	0.55144988	0.72774477
ENSCGRG00001022153	76.403653	1.0105892	0.01519667	0.20814278	0.79038629	0.88303231
ENSCGRG00001022159	1031.55877	-1.0155898	-0.0223178	0.08391451	0.77203857	0.87322376
ENSCGRG00001022160	26.7728899	1.0910211	0.125679	0.22673332	0.22058881	0.40793166
ENSCGRG00001022177	1303.74244	-1.2294624	-0.2980276	0.08650934	0.00019045	0.00143596
ENSCGRG00001022183	11209.0077	-1.0222489	-0.0317465	0.05216903	0.53008847	0.71061904
ENSCGRG00001022191	133.825268	1.01649414	0.02360189	0.14298789	0.82812776	0.90782091
ENSCGRG00001022201	840.252447	1.19656832	0.25890277	0.0845249	0.00091488	0.00541996
ENSCGRG00001022208	10681.3207	-1.017607	-0.0251806	0.05484864	0.63434204	0.78670941
ENSCGRG00001022210	1558.53495	1.57316922	0.65367386	0.07684109	1.97E-18	1.05E-16
ENSCGRG00001022215	441.498192	-1.278447	-0.3543924	0.15657341	0.00492743	0.02185609
ENSCGRG00001022223	3.135135	1.02874916	0.04089125	0.21763591	0.29002693	0.48647964
ENSCGRG00001022248	2367.32565	-1.0811241	-0.1125321	0.06052447	0.05190133	0.14301055
ENSCGRG00001022251	158.590275	1.03907594	0.0553011	0.14692568	0.61104624	0.77118285
ENSCGRG00001022264	363.892925	-1.0160357	-0.0229511	0.11421454	0.81191768	0.89735937
ENSCGRG00001022265	439.879142	-1.1836133	-0.2431978	0.14750913	0.03720766	0.11119103
ENSCGRG00001022274	52.0449747	-1.1403289	-0.18945	0.24299912	0.12589186	0.27486809
ENSCGRG00001022278	3.4160227	-1.023727	-0.0338311	0.21525559	0.40390172	0.60358923
ENSCGRG00001022279	1029.86021	-1.1909052	-0.2520585	0.08086642	0.00078694	0.00477758
ENSCGRG00001022282	36.9739988	1.01888093	0.02698546	0.18682641	0.77724016	0.87575393
ENSCGRG00001022284	2866.69358	-1.0584742	-0.0819861	0.09597052	0.34053371	0.5400284
ENSCGRG00001022286	59.4195879	1.05176914	0.07281807	0.18128735	0.49824522	0.68445268
ENSCGRG00001022287	540.264942	-1.1995103	-0.2624455	0.13888639	0.01995802	0.06846873
ENSCGRG00001022311	1.61785576	-1.0050979	-0.007336	0.21362244	0.79787361	0.88793762
ENSCGRG00001022326	16.711361	-1.0739218	-0.102889	0.23044351	0.20538305	0.38948289
ENSCGRG00001022329	1319.91936	1.12160759	0.16556802	0.08052424	0.02619273	0.08445226
ENSCGRG00001022347	43.8616487	-1.0767012	-0.1066179	0.20369877	0.31546772	0.51446741
ENSCGRG00001022350	8025.45849	-1.0725331	-0.1010222	0.0875796	0.20435029	0.38815646
ENSCGRG00001022376	2477.78439	-1.0362345	-0.0513505	0.05899478	0.36523349	0.56621578
ENSCGRG00001022379	7.87161878	-1.0692071	-0.0965413	0.24460889	0.0267235	0.08573205
ENSCGRG00001022384	1233.23139	1.00321994	0.00463793	0.08794114	0.94601325	0.97323002
ENSCGRG00001022403	19.7364689	-1.0345897	-0.0490587	0.206262	0.52145112	0.70372828
ENSCGRG00001022412	128.683777	-1.2440042	-0.3149914	0.22495997	0.03167933	0.09797495
ENSCGRG00001022419	1.64386744	-1.0052501	-0.0075544	0.21360649	0.79499571	0.88617256
ENSCGRG00001022425	3734.16802	1.03871407	0.05479858	0.05374906	0.29230351	0.48940112
ENSCGRG00001022426	5.25010353	-1.0279997	-0.0398398	0.21673907	0.33483759	0.53462165
ENSCGRG00001022428	3.75021792	-1.0235907	-0.033639	0.21674685	0.30978849	0.5081166

ENSCGRG00001022441	5.19253624	-1.04089	-0.0578177	0.22239722	0.18630689	0.36364036
ENSCGRG00001022446	35.8923674	-1.0533989	-0.0750518	0.19470571	0.45606729	0.6503867
ENSCGRG00001022470	177.549241	-1.2186833	-0.2853233	0.17633449	0.02800517	0.08868879
ENSCGRG00001022478	41.708768	1.06617744	0.09244756	0.19829865	0.37570314	0.57748467
ENSCGRG00001022491	926.402797	1.1527324	0.20505764	0.08320663	0.00747982	0.03072852
ENSCGRG00001022504	1086.28136	1.17894147	0.2374921	0.07440383	0.00067344	0.00417761
ENSCGRG00001022505	5.52145939	1.03633578	0.05149153	0.21824026	0.30563117	0.50373063
ENSCGRG00001022511	4.59427872	1.03201784	0.04546791	0.21880723	0.25108429	0.44235866
ENSCGRG00001022523	25.4051431	-1.015522	-0.0222214	0.20156393	0.76381976	0.86927249
ENSCGRG00001022529	639.33666	-1.0985436	-0.1355921	0.08747742	0.08849949	0.21369998
ENSCGRG00001022540	39.6644929	-1.0113063	-0.0162201	0.18253814	0.86613301	0.92774752
ENSCGRG00001022542	1494.04191	-2.1633226	-1.1132488	0.30469208	1.33E-05	0.00013366
ENSCGRG00001022547	160.529574	-1.4329373	-0.5189755	0.23734416	0.00285726	0.01403422
ENSCGRG00001022558	87.3701254	-1.6446925	-0.7178179	0.40232397	0.0036237	0.01706604
ENSCGRG00001022578	6593.94591	-1.020203	-0.0288562	0.05080594	0.56287785	0.73507789
ENSCGRG00001022585	3139.02603	1.14146998	0.19089292	0.05755226	0.00055634	0.0035705
ENSCGRG00001022606	2317.90649	-1.0226297	-0.0322839	0.07187931	0.63298929	0.78571636
ENSCGRG00001022607	184.951675	1.31589964	0.39604946	0.16231044	0.00266984	0.0133128
ENSCGRG00001022609	3966.27386	-1.2949841	-0.3729344	0.17876122	0.00663859	0.02776584
ENSCGRG00001022613	85.5530174	1.13768517	0.18610138	0.19734597	0.14061405	0.29768071
ENSCGRG00001022614	10.1138829	1.00996219	0.01430129	0.21053342	0.76317	0.86905384
ENSCGRG00001022619	8672.22375	1.11062561	0.15137257	0.08406942	0.04931685	0.13769591
ENSCGRG00001022627	1084.84069	-1.0823339	-0.1141457	0.07203165	0.09153947	0.21877249
ENSCGRG00001022636	2364.6123	-1.2460882	-0.3174061	0.05714795	9.15E-09	1.68E-07
ENSCGRG00001022651	11.2059887	1.03823358	0.05413106	0.21121093	0.44367145	0.6404729
ENSCGRG00001022655	4349.49783	-1.2823408	-0.3587798	0.09965852	7.98E-05	0.00066702
ENSCGRG00001022659	3.26381262	1.00663686	0.00954333	0.21098454	0.82975022	0.90823723
ENSCGRG00001022662	5.55051867	-1.0116349	-0.0166888	0.20996773	0.73802499	0.85372044
ENSCGRG00001022667	718.352153	-1.1475592	-0.1985686	0.10703517	0.03410107	0.10400825
ENSCGRG00001022668	1176.64256	-1.2833681	-0.359935	0.09711731	5.24E-05	0.00045708
ENSCGRG00001022669	5245.93476	-1.1217485	-0.1657492	0.05727182	0.00260406	0.01304067
ENSCGRG00001022684	1.79501342	-1.0180149	-0.0257587	0.21506147	0.43935745	0.63688683
ENSCGRG00001022692	659.930793	1.02390802	0.03408612	0.10426482	0.709567	0.83605162
ENSCGRG00001022693	3180.5342	-1.0869335	-0.1202637	0.0670415	0.05768695	0.15521315
ENSCGRG00001022694	20.357738	-1.055264	-0.077604	0.21358038	0.34909175	0.54880104
ENSCGRG00001022702	2.76222763	1.00298905	0.00430586	0.2132644	0.89055475	0.94172085
ENSCGRG00001022727	62.4996869	-1.0395749	-0.0559938	0.18573431	0.58036435	0.74680766
ENSCGRG00001022733	211.079028	1.03071915	0.04365128	0.1346744	0.67986683	0.81587815
ENSCGRG00001022741	316.609514	1.13655796	0.18467126	0.11508236	0.06032271	0.16068496
ENSCGRG00001022744	6.21202744	-1.002791	-0.004021	0.21014834	0.92830738	0.96397678
ENSCGRG00001022756	23.929388	-1.0242441	-0.0345596	0.19951619	0.6701182	0.80967875
ENSCGRG00001022762	411.168061	1.15144678	0.20344773	0.11878873	0.04383452	0.12621267
ENSCGRG00001022768	13.1724237	1.01433867	0.02053942	0.20191094	0.77930653	0.87673482
ENSCGRG00001022769	79.3878264	1.37521654	0.4596588	0.36571564	0.01618032	0.05786429
ENSCGRG00001022789	1991.09648	-1.6048021	-0.6823954	0.08118943	4.51E-18	2.36E-16
ENSCGRG00001022792	942.01384	-1.0874135	-0.1209006	0.0910676	0.14138679	0.29887207
ENSCGRG00001022800	239.677766	-1.4331737	-0.5192135	0.20338664	0.00113948	0.00649031
ENSCGRG00001022809	4.83251223	1.03618384	0.05127999	0.21930685	0.26561798	0.45949579
ENSCGRG00001022813	756.457902	-1.0294152	-0.041825	0.08712147	0.57745309	0.74410617
ENSCGRG00001022861	1088.6981	1.03914567	0.05539791	0.09235649	0.50674989	0.69116973

ENSCGRG00001022886	127.718222	1.00609809	0.00877097	0.15037357	0.93639675	0.96922329
ENSCGRG00001022897	808.392209	1.07172361	0.09993289	0.10092465	0.2676929	0.46208679
ENSCGRG00001022902	21.8721751	1.02326097	0.03317414	0.20081833	0.67910639	0.81532652
ENSCGRG00001022909	892.8132	-1.0554351	-0.0778378	0.07996636	0.29344125	0.49066471
ENSCGRG00001022918	11789.2198	-1.0582893	-0.0817341	0.0468542	0.07348165	0.18569738
ENSCGRG00001022919	1682.02227	1.16192724	0.21651973	0.07763545	0.00278793	0.01377945
ENSCGRG00001022935	2.09244897	-1.0138519	-0.0198469	0.21513146	0.45949793	0.65351044
ENSCGRG00001022936	3990.78408	-1.0765553	-0.1064224	0.07105505	0.11173996	0.25276159
ENSCGRG00001022943	396.199453	1.05234163	0.07360314	0.11476557	0.44963574	0.64526979
ENSCGRG00001022947	1396.87529	-2.348957	-1.2320203	0.09730625	6.43E-38	9.25E-36
ENSCGRG00001022954	1655.66818	1.10226021	0.14046484	0.06532563	0.02365037	0.07836554
ENSCGRG00001022958	13.342628	-1.0390355	-0.055245	0.21745601	0.32759239	0.52745833
ENSCGRG00001023010	1750.46985	-1.2041478	-0.2680125	0.09537221	0.00188822	0.00994169
ENSCGRG00001023034	4701.9551	1.00548029	0.00788481	0.0467836	0.86651209	0.92794014
ENSCGRG00001023047	33.3306908	1.0182244	0.02605555	0.18426109	0.72921248	0.84825849
ENSCGRG00001023054	4116.68845	-1.0383593	-0.0543057	0.05349092	0.29386113	0.49111037
ENSCGRG00001023061	3.5039112	-1.0110359	-0.0158343	0.21274787	0.67945898	0.81556886
ENSCGRG00001023065	8363.99817	1.08320945	0.11531223	0.08822985	0.14527576	0.30482923
ENSCGRG00001023078	74.8085738	1.01155014	0.01656784	0.16681409	0.87734254	0.93416734
ENSCGRG00001023084	68.2038361	1.08857554	0.12244153	0.19857376	0.28029566	0.47607895
ENSCGRG00001023090	886.233014	-1.7473776	-0.8051914	0.08128912	3.24E-24	2.46E-22
ENSCGRG00001023096	914.240711	1.0451315	0.06368447	0.09379063	0.45044643	0.64594412
ENSCGRG00001023105	89.1824966	-2.9108816	-1.5414562	0.27966508	2.00E-09	4.08E-08
ENSCGRG00001023108	13.0486392	-1.0827741	-0.1147323	0.24427388	0.12784946	0.27796191
ENSCGRG00001023123	1385.6601	-1.4125179	-0.4982692	0.07615403	9.32E-12	2.65E-10
ENSCGRG00001023141	601.400974	1.16523398	0.22061968	0.1134758	0.02421596	0.07988834
ENSCGRG00001023145	631.18782	-1.3168537	-0.3970951	0.119746	0.00018297	0.00139017
ENSCGRG00001023147	4.43413738	1.0188138	0.0268904	0.21283452	0.55275983	0.72867565
ENSCGRG00001023149	5.89136606	1.05838173	0.08186007	0.23113516	0.13033906	0.28179634
ENSCGRG00001023157	45.6620891	1.36219912	0.44593761	0.33963041	0.01631369	0.05811411
ENSCGRG00001023161	628.094905	1.04095334	0.0579054	0.11566791	0.55493555	0.73030078
ENSCGRG00001023178	5.22969671	-1.0316125	-0.0449011	0.21920835	0.22006048	0.40733182
ENSCGRG00001023192	3340.88399	-1.0330444	-0.0469023	0.05018795	0.33514841	0.53498456
ENSCGRG00001023196	30.7836848	-1.0183222	-0.0261941	0.18744024	0.77918698	0.87673482
ENSCGRG00001023203	469.447083	1.034566	0.04902568	0.09431447	0.56382407	0.73564742
ENSCGRG00001023209	57.5648115	-1.0347862	-0.0493327	0.17944668	0.63293865	0.78571636
ENSCGRG00001023211	3211.79302	-1.0978309	-0.1346559	0.07029226	0.04146046	0.12088759
ENSCGRG00001023214	10.3118488	1.01856126	0.02653275	0.21170106	0.5951645	0.75846172
ENSCGRG00001023215	458.920266	-1.0829947	-0.1150262	0.1182682	0.24722203	0.43796352
ENSCGRG00001023217	1189.68667	-1.1390803	-0.1878694	0.10468984	0.04084007	0.11960707
ENSCGRG00001023219	9514.54232	1.01806804	0.02583399	0.04927395	0.59064545	0.75390277
ENSCGRG00001023221	532.599564	1.13842287	0.18703654	0.1009196	0.03612808	0.1085622
ENSCGRG00001023222	1015.46712	1.1174917	0.16026412	0.08431428	0.0383027	0.11375791
ENSCGRG00001023231	160.973705	-1.0481522	-0.0678483	0.15083792	0.5360052	0.71485962
ENSCGRG00001023234	26.5594669	-1.0171956	-0.0245971	0.19716699	0.76372043	0.86923666
ENSCGRG00001023240	880.320112	-1.1252579	-0.1702558	0.08022002	0.02147212	0.07263213
ENSCGRG00001023247	48481.231	1.00173183	0.00249634	0.05846217	0.95359594	0.97755794
ENSCGRG00001023264	357.758581	1.06476628	0.09053679	0.1267564	0.38228811	0.58397551
ENSCGRG00001023286	6366.64005	-1.0587088	-0.0823058	0.04940574	0.08641197	0.20968695
ENSCGRG00001023314	4.75688928	-1.022482	-0.0320755	0.21662183	0.31175244	0.51035767

ENSCGRG00001023328	919.077397	-1.0107422	-0.015415	0.15498598	0.83566438	0.91187361
ENSCGRG00001023334	5598.64687	-1.1405574	-0.1897391	0.06036846	0.00102015	0.00593733
ENSCGRG00001023335	4743.11801	-1.0103405	-0.0148416	0.04954684	0.76036058	0.86717889
ENSCGRG00001023344	486.85325	-1.1313595	-0.1780575	0.09594738	0.03810163	0.11327691
ENSCGRG00001023349	1927.58083	-1.2101447	-0.2751796	0.07971081	0.0002164	0.00160156
ENSCGRG00001023359	27.8013927	1.03454526	0.04899677	0.2007447	0.56865346	0.73863829
ENSCGRG00001023368	20.2458719	-1.0877376	-0.1213305	0.23480322	0.1908742	0.36962885
ENSCGRG00001023403	102.720449	1.01558464	0.02231049	0.15695926	0.83770231	0.91311833
ENSCGRG00001023417	226.416796	-1.0062999	-0.0090603	0.12994765	0.92929437	0.96437629
ENSCGRG00001023421	60.958961	1.04874084	0.06865822	0.17622717	0.52668955	0.70796361
ENSCGRG00001023423	4.02803988	1.00011937	0.0001722	0.2109453	0.99859959	0.99959215
ENSCGRG00001023430	1231.89815	-1.1105981	-0.1513368	0.07261001	0.02621404	0.08447844
ENSCGRG00001023445	5.02573126	1.00635579	0.00914045	0.20930517	0.85790489	0.92418115
ENSCGRG00001023450	2393.07054	-1.1010086	-0.1388257	0.0623234	0.01934611	0.06667302
ENSCGRG00001023452	2675.62084	-1.1894561	-0.250302	0.08010361	0.00078193	0.0047517
ENSCGRG00001023489	40.3438131	-1.0476247	-0.067122	0.18742783	0.5103849	0.69427955
ENSCGRG00001023510	151.243151	1.07821967	0.10865114	0.16199285	0.34965408	0.54924203
ENSCGRG00001023512	4046.26802	-1.1267745	-0.1721988	0.06570853	0.00567906	0.02451103
ENSCGRG00001023522	1958.06986	1.0568447	0.07976339	0.05972916	0.16406829	0.3321294
ENSCGRG00001023523	192.799125	1.01146171	0.0164417	0.17141227	0.87610054	0.93339951
ENSCGRG00001023528	2.47835567	1.02813734	0.040033	0.22006265	0.08003921	0.19797302
ENSCGRG00001023532	5.14560246	1.02199495	0.03138806	0.21425262	0.463744	0.65728516
ENSCGRG00001023587	24.5231209	-1.0025538	-0.0036797	0.19444058	0.96336672	0.98254201
ENSCGRG00001023591	2992.86391	1.06028408	0.08445085	0.05796373	0.12928433	0.27999653
ENSCGRG00001023596	1559.61872	-1.278206	-0.3541204	0.07457403	5.41E-07	7.29E-06
ENSCGRG00001023620	2.87516156	1.03023107	0.04296795	0.21847585	0.25757898	0.44986254
ENSCGRG00001023638	752.959338	-1.008247	-0.0118491	0.1180262	0.90052698	0.9485077
ENSCGRG00001023639	258.144401	-1.028447	-0.0404675	0.15739368	0.70820543	0.8350127
ENSCGRG00001023642	4.92348119	-1.0168968	-0.0241733	0.21310726	0.56656314	0.73734368
ENSCGRG00001023643	2.21367302	-1.0351602	-0.0498541	0.22355713	0.01251166	0.04705648
ENSCGRG00001023646	1140.80332	-1.017423	-0.0249196	0.07137842	0.71055105	0.83674589
ENSCGRG00001023672	182.82693	1.09789299	0.13473745	0.14568404	0.2285552	0.41742118
ENSCGRG00001023683	1399.03334	-1.0245973	-0.035057	0.08588627	0.65577745	0.8004869
ENSCGRG00001023699	26.1890991	1.02308721	0.03292913	0.19489869	0.70911108	0.83566817
ENSCGRG00001023707	585.843784	1.03588733	0.0508671	0.10466464	0.57695247	0.74394362
ENSCGRG00001023711	912.979735	1.07038727	0.09813287	0.08090662	0.18989736	0.36857351
ENSCGRG00001023748	20.6240526	-1.0738496	-0.102792	0.22348568	0.25212632	0.44337103
ENSCGRG00001023751	617.752554	-1.1566584	-0.2099629	0.13057957	0.05049171	0.14006036
ENSCGRG00001023773	81.2367928	-1.0958313	-0.1320257	0.18836868	0.2578061	0.45017668
ENSCGRG00001023781	197.979559	1.06338426	0.08866301	0.13625825	0.40762122	0.60672593
ENSCGRG00001023784	497.526378	-1.0995905	-0.1369664	0.12433485	0.18119665	0.35715173
ENSCGRG00001023791	10594.6161	1.03089366	0.04389552	0.05024054	0.36925625	0.57058081
ENSCGRG00001023808	9.41136781	1.04403625	0.0621718	0.21936099	0.29088932	0.48747935
ENSCGRG00001023811	56.1488407	1.22204811	0.28930108	0.31621233	0.05059873	0.14023576
ENSCGRG00001023822	232.530617	-1.0051113	-0.0073552	0.20929187	0.87798622	0.93464187
ENSCGRG00001023830	580.103238	-1.2001289	-0.2631894	0.10534609	0.0047885	0.02135817
ENSCGRG00001023836	47.2195848	1.02725503	0.03879439	0.18550647	0.69421375	0.82509539
ENSCGRG00001023837	23.082354	-2.0627141	-1.0445439	0.59209324	0.00251239	0.0126558
ENSCGRG00001023850	2.62020548	-1.0187787	-0.0268407	0.21562936	0.39557931	0.59623144
ENSCGRG00001023881	56.1124148	-1.0411029	-0.0581126	0.18505936	0.56846068	0.73857312

ENSCGRG00001023882	2509.08108	-1.0040157	-0.0057819	0.06396098	0.92401226	0.96109284
ENSCGRG00001023910	6.28228025	-1.0329294	-0.0467417	0.21673659	0.3478498	0.54741474
ENSCGRG00001023913	30.9918817	1.04747675	0.06691822	0.20674894	0.43949211	0.63700995
ENSCGRG00001023918	841.296917	-1.0217818	-0.0310871	0.15797219	0.77218622	0.87322376
ENSCGRG00001023932	285.326749	1.04166371	0.05888959	0.1286231	0.5708003	0.73973875
ENSCGRG00001023948	4.03592028	1.01184892	0.01699389	0.21198434	0.6933678	0.82462553
ENSCGRG00001023968	86.1494049	-1.1335978	-0.1809089	0.18913995	0.14458162	0.30379387
ENSCGRG00001023976	441.106965	1.01073499	0.01540478	0.0991034	0.86339568	0.92669449
ENSCGRG00001023990	43.4471372	-1.321631	-0.4023194	0.42451646	0.02339942	0.0777351
ENSCGRG00001024004	874.023194	-1.0767794	-0.1067227	0.09075584	0.19396693	0.37358539
ENSCGRG00001024015	787.96106	1.03471509	0.04923357	0.12753851	0.634081	0.78661414
ENSCGRG00001024019	806.631244	-1.1871821	-0.2475412	0.13467213	0.02523932	0.0822477
ENSCGRG00001024021	134.720852	-1.0902213	-0.124621	0.15555857	0.27252416	0.46833737
ENSCGRG00001024024	3480.37876	-1.5429074	-0.6256515	0.05386065	4.00E-32	4.61E-30
ENSCGRG00001024026	76.1659749	1.28246123	0.35891521	0.24545654	0.02260702	0.07569156
ENSCGRG00001024056	226.06608	1.27204404	0.34714862	0.15198854	0.00508884	0.02246306
ENSCGRG00001024060	22.8178817	-1.0168513	-0.0241087	0.19539624	0.77436695	0.87413118
ENSCGRG00001024082	86.9551984	-1.0719265	-0.100206	0.16662833	0.37549149	0.57736718
ENSCGRG00001024088	13.7796159	-1.047231	-0.0665797	0.21116863	0.40092156	0.60080425
ENSCGRG00001024092	2.22068669	1.01463117	0.02095538	0.21493823	0.48160369	0.67167591
ENSCGRG00001024101	461.305228	-1.027599	-0.0392775	0.10133466	0.66161025	0.80425388
ENSCGRG00001024109	198.847843	1.35504978	0.43834586	0.18438747	0.00250817	0.01263954
ENSCGRG00001024110	25.9798019	1.0046192	0.00664875	0.18924727	0.94289955	0.97203793
ENSCGRG00001024111	10614.6988	1.15397768	0.20661532	0.14399869	0.06970566	0.17876466
ENSCGRG00001024129	153.791373	-1.1672311	-0.2230902	0.19827538	0.0871361	0.21116411
ENSCGRG00001024137	1.77732444	-1.0021383	-0.0030817	0.21378606	0.90525291	0.95071842
ENSCGRG00001024170	8.2439938	-1.0779601	-0.1083038	0.25304572	0.0193048	0.06654856
ENSCGRG00001024190	24208.8428	-1.0166784	-0.0238634	0.0674585	0.70909688	0.83566817
ENSCGRG00001024215	313.305071	-1.1273015	-0.1728734	0.1326356	0.10500687	0.24168912
ENSCGRG00001024219	38.6599168	-1.0298075	-0.0423747	0.18424806	0.66872425	0.80875733
ENSCGRG00001024250	3996.54729	1.10705722	0.14672979	0.05256138	0.00388368	0.01806463
ENSCGRG00001024251	188.764551	1.0265882	0.03785759	0.13477436	0.72059723	0.84276915
ENSCGRG00001024282	524.28799	-1.1223155	-0.1664783	0.10166654	0.06358135	0.16734683
ENSCGRG00001024289	8.06002854	-1.0506804	-0.0713239	0.22637783	0.16279736	0.33025443
ENSCGRG00001024293	6.80010549	-1.0226713	-0.0323425	0.21131997	0.55336072	0.72912475
ENSCGRG00001024300	2846.81685	-1.0318072	-0.0451735	0.06276276	0.4512806	0.64656129
ENSCGRG00001024303	63.3021352	1.08785158	0.12148173	0.2094427	0.26857775	0.46330205
ENSCGRG00001024314	32.2827582	-1.1011684	-0.1390351	0.22925886	0.19643977	0.37705918
ENSCGRG00001024315	102667.025	-1.1165584	-0.1590587	0.06824649	0.01363187	0.05042572
ENSCGRG00001024319	45.1895079	-1.0319606	-0.0453879	0.18759776	0.64162149	0.79121788
ENSCGRG00001024320	662.409524	-1.027492	-0.0391271	0.10805901	0.67456918	0.81214579
ENSCGRG00001024329	3686.74389	-1.0694235	-0.0968333	0.08215588	0.20092883	0.38336287
ENSCGRG00001024351	571.590433	-1.0191749	-0.0274016	0.11949534	0.78033543	0.87706145
ENSCGRG00001024354	1689.60558	-1.10198	-0.1400981	0.09163745	0.08973569	0.21570918
ENSCGRG00001024361	1199.70323	-1.0341112	-0.0483914	0.07339652	0.48238181	0.67195641
ENSCGRG00001024366	124.615593	-1.0613599	-0.085914	0.1563649	0.43632537	0.63385439
ENSCGRG00001024383	139.090511	1.03432713	0.04869254	0.14854345	0.65272947	0.7986117
ENSCGRG00001024393	10.8469742	1.02724407	0.038779	0.20964974	0.54607192	0.72332461
ENSCGRG00001024405	3.30592169	-1.0021418	-0.0030867	0.21330081	0.91693004	0.95692723
ENSCGRG00001024420	3560.78436	1.01226788	0.01759113	0.06033919	0.76042315	0.86717889

ENSCGRG00001024425	36.3603333	-1.1154788	-0.1576631	0.24516342	0.15177612	0.31390274
ENSCGRG00001024436	2582.23311	-1.0825245	-0.1143997	0.14119392	0.3131773	0.51207488
ENSCGRG00001024444	4350.75801	1.08412799	0.11653508	0.05549021	0.03022121	0.09436397
ENSCGRG00001024447	9.17219762	1.02302905	0.03284711	0.2125124	0.52414449	0.70595005
ENSCGRG00001024450	509.822326	1.01560856	0.02234446	0.13157195	0.83171754	0.90953575
ENSCGRG00001024456	1462.46237	-1.4453946	-0.5314634	0.11461209	4.62E-07	6.31E-06
ENSCGRG00001024458	438.700212	-1.0205705	-0.0293758	0.09555386	0.73093587	0.84920084
ENSCGRG00001024473	34.8691762	1.01254911	0.01799189	0.18692245	0.84992255	0.91932009
ENSCGRG00001024498	134.252851	1.04821049	0.06792845	0.15764133	0.54015739	0.71845252
ENSCGRG00001024501	94.1731666	-1.0146201	-0.0209397	0.16550768	0.84247447	0.91543734
ENSCGRG00001024508	1296.06715	1.03697689	0.05238374	0.07726614	0.46767314	0.66015349
ENSCGRG00001024517	606.854464	1.05006409	0.07047739	0.09575905	0.41117607	0.61033203
ENSCGRG00001024520	21.9662663	1.03203853	0.04549683	0.20446731	0.56249844	0.73502849
ENSCGRG00001024532	30.72032	1.02416051	0.03444184	0.19738692	0.6845145	0.81880949
ENSCGRG00001024544	15.2697143	-1.0015926	-0.0022958	0.19859628	0.97439384	0.98843721
ENSCGRG00001024546	180.344221	1.39240666	0.47758062	0.20147637	0.00222888	0.01141164
ENSCGRG00001024550	77.3648661	-1.0405678	-0.057371	0.19417791	0.54642694	0.72332988
ENSCGRG00001024555	11.1377123	1.05918368	0.0829528	0.22768414	0.20131573	0.38368917
ENSCGRG00001024575	2.11774912	-1.0012895	-0.0018591	0.2127912	0.95358239	0.97755794
ENSCGRG00001024602	85.6024563	1.01794981	0.02566643	0.16227289	0.81230141	0.89751717
ENSCGRG00001024608	164.812646	-1.2064038	-0.2707128	0.28310218	0.05930273	0.15869395
ENSCGRG00001024620	7764.18054	-1.0355271	-0.0503652	0.06725202	0.43439754	0.63198553
ENSCGRG00001024626	1049.33842	-1.2123611	-0.2778194	0.20208885	0.04273906	0.1238379
ENSCGRG00001024628	823.458086	-1.0368595	-0.0522204	0.12848742	0.61302168	0.77253396
ENSCGRG00001024630	92.7337639	1.07209197	0.10042868	0.17315341	0.37706617	0.57902394
ENSCGRG00001024654	2306.36298	-1.1685558	-0.2247266	0.07214915	0.00092976	0.00549618
ENSCGRG00001024656	225.627086	-1.3886178	-0.4736496	0.17869813	0.00105517	0.00609962
ENSCGRG00001024670	337.836148	1.04218523	0.05961172	0.13153852	0.57070087	0.73973875
ENSCGRG00001024677	5.00777067	-1.0022772	-0.0032816	0.21087815	0.93710679	0.96957742
ENSCGRG00001024688	1317.96808	1.10285879	0.14124809	0.07676244	0.04830642	0.13561369
ENSCGRG00001024695	232.987195	-1.0986239	-0.1356976	0.14555112	0.22317947	0.41142965
ENSCGRG00001024702	36.3622499	-1.3286475	-0.4099584	0.60769701	0.01904278	0.06587578
ENSCGRG00001024711	598.789187	-1.0358558	-0.0508232	0.08266799	0.50482413	0.68920357
ENSCGRG00001024724	12195.2091	-1.073609	-0.1024687	0.04631846	0.02324315	0.07731634
ENSCGRG00001024731	1592.1265	-1.1912829	-0.2525161	0.07560208	0.00034604	0.00235785
ENSCGRG00001024742	729.516685	1.03999094	0.05657096	0.07873939	0.43694333	0.6344643
ENSCGRG00001024749	1933.73034	-1.3628646	-0.4466423	0.07822025	2.09E-09	4.23E-08
ENSCGRG00001024750	4.52344161	-1.020869	-0.0297977	0.21483447	0.44109736	0.63861406
ENSCGRG00001024778	580.502305	1.36932325	0.45346306	0.14929661	0.00035955	0.00243695
ENSCGRG00001024787	136.690451	1.03505296	0.04970459	0.14101751	0.64359872	0.79243557
ENSCGRG00001024790	4114.73238	1.04114236	0.05816735	0.06427209	0.34334873	0.54273164
ENSCGRG00001024792	160.820778	1.18315503	0.24263913	0.15855347	0.04598476	0.13064247
ENSCGRG00001024809	16731.0173	-1.0336831	-0.047794	0.08270482	0.53629493	0.71517157
ENSCGRG00001024817	234.092337	-1.0168388	-0.0240909	0.12367608	0.81119974	0.89713102
ENSCGRG00001024842	618.4597	-1.1151111	-0.1571874	0.09570928	0.06659005	0.17320175
ENSCGRG00001024843	1.87027406	-1.0283096	-0.0402747	0.22026886	0.06120914	0.16260662
ENSCGRG00001024849	185.813768	1.15831463	0.21202718	0.14080311	0.05955664	0.1591407
ENSCGRG00001024850	2.15185535	1.01700767	0.02433056	0.21662512	0.20990461	0.39519079
ENSCGRG00001024866	15.1215319	1.01015263	0.0145733	0.20126151	0.84503998	0.91643771
ENSCGRG00001024881	210.776597	1.06710212	0.09369824	0.13517104	0.38001391	0.58210352

ENSCGRG00001024885	1.82120879	1.0194027	0.02772408	0.21783328	0.06809456	0.17593613
ENSCGRG00001024900	3.15429935	-1.008908	-0.0127947	0.21135984	0.76746587	0.87125648
ENSCGRG00001024926	463.64219	1.20732581	0.27181505	0.10969231	0.00484768	0.02156954
ENSCGRG00001024938	804.036924	1.34772995	0.43053145	0.10211032	4.51E-06	5.04E-05
ENSCGRG00001024947	914.329576	-1.0349026	-0.049495	0.06950451	0.44921218	0.64475161
ENSCGRG00001024983	135.82349	1.22289632	0.29030209	0.22376823	0.04361257	0.1258565
ENSCGRG00001024987	856.281671	-1.025177	-0.035873	0.08811394	0.65624242	0.80076828
ENSCGRG00001025005	43.9118123	-1.0022027	-0.0031743	0.18172878	0.97244078	0.98767978
ENSCGRG00001025010	224.772648	-1.0905692	-0.1250813	0.12463751	0.22312938	0.41142965
ENSCGRG00001025020	124.302099	-1.5547557	-0.6366879	0.26386844	0.00118275	0.00668626
ENSCGRG00001025043	753.084115	1.14900258	0.20038204	0.07985442	0.00681809	0.02840318
ENSCGRG00001025050	898.994484	1.03595015	0.05095459	0.10146662	0.56932098	0.73906967
ENSCGRG00001025059	1706.78353	1.1002653	0.13785143	0.08169577	0.06763606	0.17517547
ENSCGRG00001025070	2236.41676	-1.1707246	-0.2274018	0.08528339	0.00372772	0.01747242
EPRS	16476.0459	1.01842044	0.02633329	0.04872043	0.58190554	0.74773936
EXT1	2091.06236	1.20142226	0.2647433	0.06893985	5.10E-05	0.00044547
Eaf2	30.0867149	1.01738248	0.02486216	0.19538668	0.7722269	0.87322376
Eapp	1114.32701	1.22192518	0.28915595	0.07598259	5.22E-05	0.00045576
Ears2	214.039819	1.01545604	0.02212779	0.14076105	0.83692938	0.91250876
Ebag9	1087.82744	-1.0070338	-0.0101121	0.08454273	0.89588983	0.94546226
Ebf2	2730.0023	-1.0536493	-0.0753947	0.0639091	0.2160341	0.40217945
Ebf3	824.693755	1.07317949	0.10189139	0.08438675	0.18774545	0.36539338
Ebf4	70.8891056	1.45837408	0.54436082	0.29931372	0.00532438	0.02327827
Ebi3	2.29861192	-1.0226901	-0.0323691	0.21881742	0.03445885	0.10475938
Ebna1bp2	3664.25105	-1.0609955	-0.0854185	0.05978639	0.13461772	0.28845681
Ebp	1356.10786	1.05555493	0.07800165	0.07885217	0.28681488	0.48317186
Ebpl	639.265639	1.42431013	0.51026331	0.11460349	1.23E-06	1.56E-05
Ecd	2579.15195	1.01226368	0.01758515	0.06245345	0.76922845	0.87171058
Ece1	8.25800534	-1.0146692	-0.0210094	0.21080577	0.66744664	0.80789865
Ech1	297.506487	1.08389664	0.11622719	0.11684576	0.23908609	0.42897266
Echdc1	1085.51004	-1.0892139	-0.1232873	0.0649689	0.04586689	0.13048077
Echs1	324.7536	1.05332791	0.07495463	0.12057769	0.45544905	0.64989401
Eci1	304.068287	-1.0696712	-0.0971674	0.12095474	0.33305435	0.53290361
Ecm1	251.5356	-9.6226944	-3.2664409	0.21372159	5.12E-54	1.68E-51
Ecm2	94.2517663	1.19620681	0.25846683	0.21982111	0.06151517	0.16327284
Ecpas	11548.6587	1.00152527	0.00219882	0.04888091	0.97512439	0.98867578
Ecsit	2220.94672	-1.0784546	-0.1089655	0.07271407	0.11055414	0.2509934
Ect2	7022.15314	-1.0288652	-0.041054	0.04216441	0.32434392	0.52374204
Ect2l	1.74925548	1.02893746	0.04115529	0.22079873	0.03187339	0.0984229
Edaradd	839.179555	-1.0185817	-0.0265617	0.08094654	0.72268887	0.84375177
Edc3	959.822429	1.01441231	0.02064416	0.07909484	0.77974015	0.87680583
Edc4	3661.32066	-1.0003875	-0.000559	0.0488982	0.99082384	0.99564272
Edem1	3033.19753	1.03562623	0.05050341	0.06222856	0.39613644	0.59664994
Edem2	1316.45474	-1.0407072	-0.0575643	0.08552959	0.46192603	0.65536299
Edem3	13725.0705	1.01263123	0.01810889	0.04470186	0.66864344	0.80873595
Edf1	1780.35437	1.01092341	0.0156737	0.06006239	0.78635186	0.88052162
Edrf1	916.500793	-1.0695229	-0.0969674	0.08900502	0.2330056	0.42235768
Eea1	2856.61859	1.03768345	0.05336641	0.06018744	0.35593016	0.55596456
Eed	1422.01603	-1.0659712	-0.0921685	0.07689918	0.19812098	0.37899047
Eef1a2	38.0019417	1.16637731	0.22203456	0.32699917	0.05990751	0.15977832

Eef1akmt1	415.808777	-1.0374746	-0.0530761	0.09461712	0.53190912	0.71191681
Eef1akmt2	352.116177	1.1078735	0.14779316	0.12997987	0.16285346	0.33029652
Eef1akmt3	222.535426	-1.0033537	-0.0048303	0.13078129	0.96075912	0.98137498
Eef1aknmt	1433.6365	-1.1788682	-0.2374024	0.08534003	0.00248336	0.01252926
Eef1b2	13071.4245	-1.2398746	-0.3101942	0.05035909	2.42E-10	5.64E-09
Eef1d	11829.2535	-1.031214	-0.0443438	0.06092232	0.44471277	0.64109505
Eef1g	22469.2605	-1.1307773	-0.1773148	0.03874546	3.13E-06	3.64E-05
Eef2	46167.0988	-1.0072393	-0.0104065	0.04394083	0.79071644	0.88313513
Eef2k	1616.63029	-1.0848537	-0.1175005	0.07379278	0.0890944	0.21468182
Eef2kmt	477.122554	1.07565124	0.10521039	0.10325998	0.24579792	0.43597712
Eefsec	572.935929	1.02729021	0.0388438	0.09613976	0.65233582	0.7985114
Eepd1	503.105543	1.08634346	0.1194803	0.09572797	0.16280692	0.33025443
Efcab11	211.778914	1.15934847	0.21331427	0.18355573	0.09380304	0.22276918
Efcab14	2890.31548	1.15028065	0.2019859	0.05632444	0.00019582	0.00146777
Efcab2	18.0773395	-1.0595113	-0.0833989	0.23077318	0.15106749	0.31297339
Efcab5	3.86361562	-1.0109835	-0.0157595	0.21247867	0.68627658	0.8196191
Efcab7	927.332205	1.04813902	0.06783009	0.08988906	0.40686431	0.60632059
Efemp1	2393.81788	1.33872221	0.42085663	0.07248814	1.33E-09	2.77E-08
Efemp2	777.665618	1.88819685	0.91700918	0.09185827	1.30E-24	1.02E-22
Efhd2	1649.95634	-1.1573669	-0.2108463	0.06300288	0.0004449	0.00293925
Efl1	2672.07752	1.02395265	0.03414901	0.05266298	0.50423982	0.68862602
Efna1	160.09897	1.01130032	0.01621149	0.14167131	0.8799921	0.93572664
Efna2	110.810286	1.14320686	0.19308648	0.21305978	0.13082626	0.28246828
Efna3	563.091507	1.2852495	0.36204845	0.1077852	0.00018842	0.00142396
Efna4	142.756937	1.19609277	0.25832929	0.20153432	0.05701027	0.15368678
Efs	676.511855	1.037694	0.05338107	0.13381038	0.61282191	0.77235819
Eftud2	11913.1279	-1.0130179	-0.0186597	0.05219109	0.71209979	0.8373415
Egf	4.65707039	1.00473096	0.00680924	0.21189154	0.86282349	0.92665838
Egfl8	1.63963685	1.01387088	0.01987394	0.21571419	0.37589522	0.57764129
Egln1	1976.27046	-1.0636429	-0.0890139	0.07405656	0.1993441	0.38079302
Egln2	256.786329	1.06131533	0.08585336	0.13536041	0.42175743	0.62021727
Egr1	3852.44692	-1.2964875	-0.3746082	0.2421805	0.0173545	0.06139495
Egr2	87.3252839	-1.1132573	-0.1547871	0.21704755	0.18748754	0.36500235
Egr3	952.275403	-1.6548595	-0.7267087	0.09405295	1.01E-15	4.35E-14
Ehbp1	1985.16683	-1.0467213	-0.0658774	0.06367059	0.2776678	0.47337297
Ehd1	1789.6014	-1.1324635	-0.1794646	0.05959673	0.00166672	0.00894132
Ehd2	1508.45559	1.17312287	0.23035413	0.07442038	0.00097046	0.0056895
Ehd3	752.796173	-1.331817	-0.4133959	0.0995106	6.34E-06	6.83E-05
Ehd4	3394.17601	-1.3418478	-0.4242211	0.06619897	2.98E-11	8.01E-10
Ehhadh	11.7633307	1.05638411	0.0791345	0.22558028	0.21660828	0.40295557
Ehmt1	3247.01088	1.07129513	0.09935599	0.05161455	0.04713949	0.13309608
Ehmt2	5833.2902	1.28315415	0.35969449	0.04842696	3.03E-14	1.11E-12
Eid1	392.127134	-1.0299433	-0.0425649	0.10396625	0.63922559	0.7904701
Eid2	29.3589724	1.04363037	0.06161083	0.20329384	0.48235382	0.67195641
Eif1ad	2696.41412	-1.3774748	-0.4620259	0.09103484	6.54E-08	1.05E-06
Eif1ax	4888.08347	-1.0812303	-0.1126738	0.13299565	0.28548768	0.48190417
Eif1b	918.189266	-1.0195879	-0.0279862	0.09155242	0.73487054	0.85171009
Eif2a	2832.64977	-1.0555342	-0.0779733	0.0609234	0.18128892	0.35720546
Eif2ak1	3409.60657	1.11723289	0.15992995	0.06536771	0.00997406	0.03917988
Eif2ak2	206.695276	1.10024009	0.13781838	0.13928558	0.20758316	0.39243511

Eif2ak3	1118.80976	-1.8995815	-0.9256816	0.07669076	1.20E-34	1.51E-32
Eif2ak4	1544.07537	-1.0289742	-0.0412068	0.07135744	0.54005181	0.71845252
Eif2b1	1526.92591	-1.0304065	-0.0432135	0.06522938	0.48603937	0.67499613
Eif2b2	1826.12105	1.01312257	0.01880872	0.06154143	0.74975993	0.86107448
Eif2b3	1690.1781	-1.0709668	-0.0989138	0.06804149	0.12459385	0.2731087
Eif2b4	1087.77502	1.01419835	0.02033984	0.08087473	0.78688802	0.88058317
Eif2b5	1791.31281	1.04244801	0.05997543	0.05862185	0.28695867	0.48329484
Eif2d	1997.96556	-1.0603591	-0.0845529	0.07394389	0.22181994	0.40961705
Eif2s1	8604.70384	-1.1796415	-0.2383485	0.05128985	1.65E-06	2.04E-05
Eif3a	16746.1524	-1.0175136	-0.0250481	0.05219794	0.61775847	0.77522395
Eif3e	3790.86588	-1.0398614	-0.0563912	0.06016468	0.32816641	0.52808662
Eif3f	3762.43701	-1.0434562	-0.0613701	0.05499713	0.24765041	0.43847985
Eif3h	3094.78191	-1.1062781	-0.1457141	0.05571462	0.00660618	0.02766431
Eif3k	2403.87661	-1.0656242	-0.0916988	0.06689734	0.14781926	0.30829773
Eif3l	8892.23261	-1.0878824	-0.1215226	0.05843977	0.03023183	0.09436397
Eif4a1	20928.7679	1.1137479	0.15542271	0.05232136	0.00220383	0.01131051
Eif4a2	5480.36553	1.00943237	0.01354425	0.05514915	0.80020555	0.8893487
Eif4b	12868.82	-1.0477815	-0.0673379	0.04708669	0.13394435	0.28745638
Eif4e2	2334.50933	1.08086141	0.11218155	0.07347343	0.10355625	0.2395802
Eif4e3	221.077828	-1.0067187	-0.0096607	0.1286432	0.92426479	0.96110334
Eif4ebp1	2822.33473	1.10100579	0.13882206	0.06833824	0.03152718	0.09764584
Eif4ebp2	334.089709	-1.2967713	-0.3749241	0.15215459	0.00262966	0.01315345
Eif4ebp3	56.0515828	1.10617616	0.14558115	0.21319585	0.21243542	0.39831641
Eif4enif1	2712.42738	1.10215596	0.14032838	0.06829582	0.02997758	0.09370736
Eif4g1	48237.118	-1.1077933	-0.1476887	0.04626778	0.00098744	0.00577057
Eif4g2	52339.8494	-1.1728474	-0.2300154	0.03851634	1.20E-09	2.50E-08
Eif4g3	5753.28031	-1.0082101	-0.0117964	0.05034063	0.80676686	0.89377182
Eif4h	2249.21003	-32.160773	-5.0072302	0.09660731	0	0
Eif5a2	355.421891	-1.0769381	-0.1069354	0.11121578	0.26129263	0.45382919
Eif5b	12961.6635	-1.1493958	-0.2008757	0.08020452	0.00789378	0.03210076
Eipr1	431.033677	-1.0599262	-0.0839638	0.09453541	0.32139281	0.52028838
Elac1	61.2418691	1.0056688	0.00815525	0.18102215	0.93534869	0.96876821
Elac2	984.000774	-1.1352907	-0.1830617	0.08851424	0.02298875	0.07660922
Elavl1	2624.46955	-1.0389623	-0.0551433	0.06482333	0.37149187	0.57286756
Elavl2	929.473253	-1.0390911	-0.0553221	0.08641621	0.48393866	0.67307243
Elf1	2359.48274	-1.2324295	-0.3015051	0.07165335	8.82E-06	9.26E-05
Elf2	1870.9528	1.00814464	0.01170264	0.06314555	0.84586308	0.91694087
Elf3	3.00476396	1.06363427	0.08900216	0.24261347	0.00122342	0.00686288
Elf4	1268.8982	1.01476268	0.02114237	0.06974667	0.7504051	0.86150648
Elfn1	7.3384677	-1.094243	-0.1299332	0.26585697	0.03831329	0.11375791
Elk1	881.975781	1.00313215	0.00451167	0.07221743	0.94823619	0.97437186
Elk3	3435.7926	1.06670617	0.09316283	0.06489285	0.13286341	0.28537564
Elk4	757.729813	1.02612789	0.03721055	0.08218007	0.62105337	0.77745467
Ell	428.141106	-1.0954203	-0.1314846	0.11805494	0.18550818	0.36269227
Ell2	1283.30386	-1.4874696	-0.5728602	0.07883316	4.83E-14	1.75E-12
Ell3	24.3229234	1.11149569	0.15250236	0.2735144	0.07669625	0.19207799
Elmo1	1023.58301	1.1235657	0.16808449	0.09578029	0.05031549	0.13969255
Elmo2	1946.63117	1.58191963	0.66167631	0.07633485	4.92E-19	2.77E-17
Elmo3	46.6002157	-1.0874121	-0.1208987	0.20223594	0.27984307	0.47556245
Elmod2	922.804601	1.16031724	0.2145193	0.09264354	0.01055266	0.04106304

Elmod3	653.646297	-1.7215025	-0.7836683	0.10626891	1.37E-14	5.19E-13
Elmsan1	3187.4422	-1.1224589	-0.1666626	0.06891845	0.01034041	0.04031062
Eloa	24086.8668	-1.0757473	-0.1053392	0.04232522	0.01104541	0.0425667
Elob	1262.15947	-1.0013529	-0.0019504	0.09596118	0.98663845	0.99322896
Elovl1	775.675453	-1.0510289	-0.0718023	0.08185859	0.34202824	0.54157995
Elp1	6490.36883	-1.0378567	-0.0536073	0.04624879	0.23011375	0.4188345
Elp2	3662.62498	-1.1275635	-0.1732086	0.06057218	0.00278578	0.01377945
Elp3	4233.92867	-1.0517859	-0.072841	0.05347622	0.15900163	0.32526523
Elp4	550.994746	-1.0711536	-0.0991653	0.08623298	0.20786689	0.39263324
Elp5	1164.94246	1.1870034	0.24732407	0.07661997	0.00056283	0.0036049
Elp6	641.503972	-1.1624539	-0.2171735	0.08338761	0.00465377	0.02091012
Emc1	6477.9129	1.15802819	0.21167038	0.05012842	1.33E-05	0.00013442
Emc10	870.834155	1.13028034	0.17668064	0.11715495	0.07438406	0.18764471
Emc2	1792.56785	-1.0198377	-0.0283396	0.06081133	0.62625501	0.78159847
Emc3	1366.26844	-1.0350016	-0.049633	0.06361901	0.41326679	0.61222939
Emc6	92.0316317	-1.1164511	-0.1589201	0.22356088	0.17596174	0.34973932
Emc7	1476.72187	1.21284049	0.27838982	0.0816605	0.00025099	0.00180628
Emc9	40.9217066	-1.0658674	-0.092028	0.19339071	0.38679973	0.58804944
Emd	994.532507	1.09301831	0.12831756	0.068283	0.04704068	0.13284636
Eme1	219.948853	1.16684298	0.22261044	0.16310749	0.06772193	0.17529155
Eme2	39.8960232	-1.047775	-0.067329	0.19381084	0.49511871	0.68176617
Emg1	836.027655	-1.0628968	-0.0880015	0.08851061	0.2736604	0.46928911
Emilin1	1395.70438	1.29399518	0.37183224	0.09379127	1.77E-05	0.00017186
Emilin2	35.2837162	1.01182346	0.0169576	0.19635207	0.83789641	0.91325219
Eml1	9.33207099	-18.739166	-4.2279848	1.18158095	4.53E-05	0.00040112
Eml2	2747.76768	1.05488828	0.07709022	0.08109503	0.30452102	0.50256561
Eml3	1783.64646	1.17643522	0.23442188	0.06701266	0.0002272	0.00166502
Eml4	2317.40397	-1.0304634	-0.0432933	0.07464325	0.53532702	0.71428289
Eml5	294.124838	-1.4780612	-0.563706	0.13998473	6.49E-06	6.95E-05
Eml6	811.237721	1.0055787	0.008026	0.07805294	0.91283616	0.95503358
Emp1	3059.33716	-1.1142314	-0.1560489	0.14721009	0.16636074	0.33536821
Emp2	189.844996	1.18938529	0.25021613	0.16626814	0.04511445	0.12902794
Emp3	979.03073	-1.2807354	-0.3569725	0.100855	9.89E-05	0.00080411
Enah	1945.6007	-1.0289401	-0.041159	0.07078653	0.54251312	0.72027106
Enc1	552.381586	-1.4814539	-0.5670137	0.11018021	3.17E-08	5.35E-07
Endod1	9849.0691	1.0117627	0.01687095	0.03985893	0.6802331	0.8159652
Endog	180.112177	1.04453198	0.06285666	0.13804947	0.55281994	0.72867565
Endou	3.88340395	1.02066099	0.02950377	0.2150654	0.43043681	0.62836416
Endov	114.270174	-1.3125537	-0.3923764	0.22751231	0.0122381	0.04627217
Eng	10304.3744	-1.1383216	-0.1869082	0.05583733	0.00051172	0.00332413
Engase	402.30847	1.11340579	0.15497949	0.12261688	0.1292444	0.27999653
Enho	10.1361488	1.11176243	0.15284853	0.28922198	0.02833186	0.08950214
Enkd1	95.3217774	1.04923534	0.06933831	0.15984211	0.53028395	0.71070699
Enkur	3.71060173	-1.0012017	-0.0017327	0.2128564	0.95565399	0.97872498
Eno2	182.698766	1.09321748	0.12858043	0.17089022	0.25918405	0.45147507
Eno3	45.452204	1.01094565	0.01570544	0.18803265	0.86675795	0.92799996
Enox1	1269.79073	1.00567568	0.00816512	0.06802187	0.89947616	0.94779055
Enox2	516.340545	1.10671851	0.14628832	0.10512548	0.11167982	0.25271479
Enpep	34.5193199	2.62772826	1.39381609	0.53206265	0.00040011	0.00267923
Enpp1	532.539767	-1.3965125	-0.4818285	0.11576047	4.86E-06	5.38E-05

Ensa	391.272301	-1.0783267	-0.1087943	0.10308431	0.22832585	0.41720666
Enthd1	4.50799484	-1.0989797	-0.1361647	0.28508411	7.43E-05	0.00062587
Entpd5	389.594396	1.38835575	0.47337729	0.11531602	6.35E-06	6.84E-05
Entpd7	711.477661	1.08027424	0.1113976	0.08139266	0.13821807	0.29388337
Entr1	1284.56524	-1.1008571	-0.1386272	0.06589761	0.02653332	0.08531423
Eny2	561.279748	-1.1418302	-0.1913481	0.1047194	0.0375902	0.11216642
Eogt	656.321607	1.20869337	0.2734483	0.10757305	0.00402797	0.01862082
Ep300	6806.03227	-1.0155342	-0.0222388	0.04622281	0.60724867	0.7678038
Ep400	6360.69941	1.08378341	0.11607647	0.05194196	0.02180474	0.07354366
Epas1	712.800552	1.0469182	0.06614873	0.10265606	0.46434558	0.65755769
Epb41	2212.0587	1.24277035	0.31355973	0.05281211	9.81E-10	2.08E-08
Epb41l1	926.087209	1.39424201	0.479481	0.08321596	1.40E-09	2.89E-08
Epb41l2	9389.40471	1.24023148	0.31060942	0.05508702	5.90E-09	1.12E-07
Epb41l4a	13.7027846	-3.7091861	-1.8911026	0.82477156	0.0007184	0.00441165
Epb41l5	729.606458	1.11423786	0.15605724	0.09692225	0.07133632	0.18163749
Epc1	1219.67856	1.02125788	0.03034721	0.06446742	0.62217051	0.77824473
Epc2	2178.20115	1.0285055	0.0405495	0.06361453	0.50505028	0.68943884
Epcam	1.78234106	-1.0168994	-0.0241769	0.21624572	0.29773752	0.49564881
Epdr1	1169.36307	-1.1536658	-0.2062253	0.08414812	0.00764289	0.03124042
Epg5	911.457965	-1.0133069	-0.0190711	0.08393015	0.80416615	0.89189337
Epha2	1846.46196	-1.1799139	-0.2386816	0.07366917	0.00056373	0.00360891
Epha5	2.01659735	-1.0343707	-0.0487533	0.22275046	0.03802241	0.11311359
Ephb3	118.563005	-1.0769354	-0.1069317	0.15448018	0.34078912	0.54021887
Ephb4	62.7757679	-1.1735001	-0.230818	0.24956913	0.08619992	0.20941038
Ephx1	1242.21805	-1.207161	-0.2716181	0.0976832	0.00203617	0.01057288
Epm2a	26.9494713	-1.5275093	-0.6111812	0.57788028	0.01059267	0.04119371
Epm2aip1	557.132422	-1.2369804	-0.3068226	0.09797153	0.00055155	0.00354864
Epn1	537.658763	-1.0610299	-0.0854653	0.10450744	0.34708093	0.54667486
Epn2	316.56661	1.14447336	0.19468388	0.14185126	0.0839377	0.20515968
Eps15	5765.05127	-1.0144301	-0.0206695	0.05226009	0.6827289	0.81728411
Eps15l1	1670.69468	-1.0254708	-0.0362864	0.05718071	0.50812734	0.69238498
Eps8	9996.51139	-1.0536935	-0.0754553	0.04462338	0.08299396	0.2033554
Eps8l2	68.9301485	1.08022439	0.11133103	0.18948949	0.32392258	0.52337798
Epyc	1.67108399	1.00527747	0.00759376	0.21384662	0.78501753	0.87956967
Eral1	1491.39757	1.00549871	0.00791122	0.06635476	0.90076566	0.9486031
Erap1	2313.1255	-1.1303359	-0.1767515	0.06503077	0.0043206	0.01963351
Erbb2	1202.05191	1.01680117	0.0240376	0.08926151	0.767968	0.8713089
Erbb4	4.50375231	-1.0106192	-0.0152394	0.21182718	0.71892694	0.84156113
Erbin	4505.51247	1.0195572	0.02794272	0.05273492	0.58125605	0.74742923
Erc1	6530.29162	1.0599042	0.08393387	0.05131196	0.08976922	0.21574929
Ercc1	1090.95306	1.00059184	0.00085359	0.08804924	0.99832098	0.99956946
Ercc2	381.109899	-1.120817	-0.1645508	0.13984154	0.13532628	0.2894184
Ercc3	1134.61497	-1.1275493	-0.1731906	0.06523279	0.00516179	0.02268284
Ercc4	545.403174	1.13154948	0.17829968	0.0900636	0.02919074	0.09169936
Ercc6	909.519924	-1.0981537	-0.13508	0.07564427	0.05554169	0.15057969
Ercc6l	3044.10754	-1.1212857	-0.165154	0.06046594	0.00431086	0.01961709
Ercc8	697.009079	1.05222181	0.07343886	0.08253817	0.33424446	0.53414066
Ereg	9.42981627	-186.53484	-7.5433013	2.74954472	7.42E-08	1.18E-06
Erf	434.451149	-1.0225544	-0.0321776	0.11039181	0.73322845	0.85081284
Erg28	894.450933	1.05949841	0.08338143	0.06977754	0.20652355	0.39100896

Ergic1	5647.26875	1.16970649	0.22614656	0.04996509	3.15E-06	3.65E-05
Ergic2	7774.86516	-1.0531144	-0.0746622	0.05435782	0.15472558	0.31839084
Ergic3	2029.21364	1.27252562	0.3476947	0.07116909	2.83E-07	4.03E-06
Eri1	1505.5173	-1.1565755	-0.2098595	0.08795889	0.00896302	0.03572446
Eri2	959.169667	1.0301277	0.04282319	0.07950926	0.56284797	0.73507789
Eri3	1139.77197	1.29170182	0.36927307	0.07530601	2.38E-07	3.42E-06
Erich1	2476.277	-1.0404598	-0.0572212	0.05215536	0.25756113	0.44986254
Erlec1	2046.49585	-1.0021464	-0.0030933	0.06814962	0.96114053	0.98152185
Erlin1	3094.65049	1.11091511	0.15174857	0.06532189	0.01485608	0.05416731
Erlin2	2204.87951	1.0468513	0.06605653	0.0622504	0.26618242	0.46022362
Ermap	1.67985415	-1.0175646	-0.0251204	0.21614305	0.31888093	0.51733343
Ermp1	3418.0915	1.10383215	0.14252081	0.05693237	0.00924635	0.03664783
Ern1	776.094486	-1.0634257	-0.0887192	0.09427075	0.29377404	0.49102889
Ero1l	5458.15781	-1.0395102	-0.0559039	0.0474392	0.2250341	0.41364426
Ero1lb	366.531408	1.12896848	0.1750052	0.10563675	0.05837121	0.15655637
Erp29	2953.0822	1.05274259	0.07415273	0.06332733	0.22019334	0.4074356
Erp44	2959.96276	1.09095618	0.12559316	0.06468692	0.04117506	0.12028563
Errfi1	2123.43648	-1.6880087	-0.7553223	0.06169374	1.65E-35	2.09E-33
Esco1	1234.49636	1.00797429	0.01145884	0.07863772	0.87656181	0.93371167
Esco2	2599.21189	1.01300126	0.01863597	0.06663875	0.77377549	0.87386763
Esf1	5123.25036	-1.0829559	-0.1149745	0.06254589	0.05396049	0.14725904
Espl1	4135.76506	-1.0032981	-0.0047504	0.05538485	0.92880916	0.9641278
Esr1	367.852027	-1.1029126	-0.1413185	0.1318951	0.18269129	0.35877287
Esr2	51.3794876	-1.2579015	-0.331019	0.47800112	0.02824525	0.08927254
Esrp2	6.28337253	1.01500892	0.02149241	0.21286522	0.60478506	0.76592156
Esrra	226.905984	-1.0849965	-0.1176904	0.16145408	0.30187981	0.49968735
Ess2	1295.94178	-1.0394775	-0.0558586	0.07383252	0.41941918	0.61826923
Esyt1	3914.24086	1.34150848	0.42385617	0.05951508	2.17E-13	7.42E-12
Esyt2	6895.37913	1.27097077	0.34593085	0.0583663	9.04E-10	1.93E-08
Etaa1	2012.52511	-1.0195015	-0.0278638	0.06634195	0.65802381	0.80156761
Etf1	3020.51071	-1.1394108	-0.188288	0.06730159	0.00313229	0.01512423
Etfa	3548.20138	1.01647843	0.0235796	0.07064433	0.72412691	0.84443019
Etfbkmt	57.8256308	1.09065179	0.12519056	0.20228076	0.27101505	0.46635795
Etfdh	2306.83872	1.20041316	0.26353104	0.06233906	1.01E-05	0.00010457
Ethe1	256.315234	1.05684516	0.07976402	0.14326453	0.46333343	0.65677783
Etnk1	370.367389	-1.1500017	-0.201636	0.12156085	0.04902844	0.13718968
Ets1	2185.61775	-1.3869942	-0.4719617	0.06815659	7.44E-13	2.43E-11
Ets2	1285.05563	-1.4416285	-0.5276994	0.08069034	8.96E-12	2.56E-10
Etv1	1056.81844	1.19958617	0.26253679	0.07875785	0.00035798	0.00242887
Etv3	1458.94901	1.01510924	0.02163499	0.06718721	0.73534214	0.85200188
Etv4	6220.01941	-1.0643086	-0.0899166	0.05265059	0.07706458	0.19273669
Etv5	868.084041	-1.2239389	-0.2915316	0.09975892	0.00118177	0.00668365
Etv6	1172.04152	1.09146202	0.12626192	0.06611499	0.04424913	0.12700681
Eva1a	36.1522687	-3.3881275	-1.7604882	0.49570983	1.45E-05	0.00014466
Eva1b	226.425768	-1.0426602	-0.060269	0.13252372	0.56466999	0.73622622
Evc	1278.84867	1.12169189	0.16567644	0.07484713	0.01793158	0.06307579
Evc2	752.239681	1.21712521	0.28347759	0.08874525	0.00051684	0.00334845
Evi5	1889.68639	-1.0071331	-0.0102544	0.06455227	0.86724092	0.92827122
Evl	945.849542	1.304681	0.3836971	0.11918161	0.0002799	0.00197222
Evpl	15.9241969	-1.0262606	-0.0373971	0.2053567	0.60467588	0.76585901

Ewsr1	8908.63912	-1.1449375	-0.1952689	0.04323592	3.81E-06	4.33E-05
Exd1	145.52516	1.24125335	0.31179761	0.18372365	0.02139236	0.07245799
Exd2	1116.45614	1.00024898	0.00035916	0.08348305	0.99702474	0.99889621
Exo1	980.622351	-1.0136526	-0.0195633	0.06995913	0.76691873	0.87111526
Exo5	217.837311	1.2602791	0.33374326	0.16081249	0.00896191	0.03572446
Exoc1	1920.80359	1.03707622	0.05252193	0.06792311	0.41557614	0.61451349
Exoc2	1986.93507	1.05962073	0.08354797	0.06425018	0.17256279	0.34496401
Exoc3	1869.32968	1.12122459	0.16507529	0.062426	0.00558183	0.02418456
Exoc3l	4.23482791	1.00168119	0.00242341	0.21224734	0.94869932	0.97456602
Exoc3l4	5.92018951	-1.0520627	-0.0732207	0.22879399	0.11790204	0.26234587
Exoc4	1868.22798	1.26232473	0.33608309	0.05635656	7.42E-10	1.60E-08
Exoc5	2913.0029	1.07957398	0.1104621	0.06273585	0.06508096	0.17046015
Exoc6	998.619156	-1.0070536	-0.0101404	0.0800852	0.89067957	0.9417751
Exoc6b	1319.2847	1.28529813	0.36210304	0.09779982	5.35E-05	0.00046546
Exoc7	873.673526	1.03345793	0.04747967	0.08269492	0.53445415	0.71345954
Exoc8	632.08488	1.01742764	0.02492619	0.09867129	0.76768997	0.87127745
Exog	315.778214	-1.0525366	-0.0738704	0.11799183	0.45339071	0.64825706
Exosc1	431.242737	-1.1094978	-0.1499068	0.10446576	0.10093368	0.23474227
Exosc10	2269.21819	-1.0357332	-0.0506524	0.06413395	0.4070854	0.60643842
Exosc2	2104.61952	-1.1724018	-0.2294671	0.07180735	0.0006892	0.00426141
Exosc3	614.163664	-1.2278085	-0.2960855	0.11349092	0.00290433	0.01421648
Exosc4	343.284447	-1.0802153	-0.1113189	0.13476843	0.29688547	0.49461606
Exosc5	234.524151	1.33607136	0.41799706	0.1941339	0.00474649	0.02122252
Exosc6	18.3232936	-1.0300255	-0.0426801	0.20854434	0.53373031	0.71299738
Exosc7	714.816336	-1.0414737	-0.0586265	0.09036879	0.47392313	0.66475866
Exosc8	2034.57581	1.01638691	0.0234497	0.06563922	0.70776919	0.8347011
Exosc9	1008.67683	-1.129817	-0.1760891	0.08400379	0.02253715	0.07551685
Exph5	957.344942	1.20907001	0.27389778	0.08115932	0.00029147	0.00203806
Ext2	1882.58386	1.12006264	0.16357942	0.07840049	0.02462854	0.0808124
Extl1	252.298147	1.58316639	0.66281289	0.22296764	0.00023438	0.00170691
Extl2	1718.0725	1.0508154	0.07150925	0.06518135	0.24917614	0.44032919
Extl3	3697.50746	1.07931133	0.11011107	0.05279997	0.03114156	0.09661503
Eya1	12.566733	-1.0438316	-0.0618889	0.21425205	0.38106974	0.58293568
Eya3	1517.75042	1.08859254	0.12246405	0.07976226	0.09770356	0.22914365
Ezh1	651.391909	1.08113611	0.11254816	0.10240823	0.21019878	0.39545402
Ezh2	2705.50063	-1.078368	-0.1088496	0.05106254	0.02787519	0.08832086
Ezr	11565.253	-1.2835627	-0.3601538	0.04393744	6.81E-17	3.21E-15
F2r	7475.71616	1.11589112	0.15819627	0.05094944	0.0013603	0.00749802
F2rl1	71.282196	-1.4911471	-0.5764226	0.33705958	0.00588913	0.02520957
F2rl2	41.9502954	-1.2892547	-0.3665373	0.40629445	0.0288924	0.09089186
F3	32.0880742	-1.0578201	-0.0810944	0.20712116	0.38075606	0.58280281
F8	278.177333	1.35101329	0.43404186	0.16645502	0.00142222	0.0077924
F8a	117.77622	-1.1003878	-0.138012	0.21119644	0.22630149	0.41490226
FTH1	9195.90484	-1.1474812	-0.1984705	0.04554384	7.85E-06	8.31E-05
FTSJ3	7313.17436	-1.0771542	-0.1072248	0.04236956	0.00975047	0.03845552
Faah	1388.05993	1.15615904	0.20933987	0.07657126	0.00340275	0.01619379
Faap100	973.513955	1.08237938	0.11420626	0.1267528	0.27031906	0.46562005
Faap20	127.341459	1.03865929	0.05472249	0.16650258	0.61480613	0.77364111
Faap24	165.049287	1.05066903	0.07130828	0.14053913	0.50985783	0.69410628
Fabp4	9.05397672	1.01765114	0.02524308	0.2134632	0.54175771	0.71960974

Fadd	159.870146	-1.1176484	-0.1604664	0.15951271	0.17072317	0.34219431
Fads1	660.096731	1.09510724	0.13107215	0.1034897	0.14774428	0.30824596
Fads3	713.720059	1.08721723	0.12064022	0.12551498	0.2419041	0.43180545
Faf1	2891.86518	1.16808588	0.22414635	0.05634367	3.65E-05	0.00032966
Faf2	2431.25756	-1.1408095	-0.190058	0.0571753	0.00054568	0.00351797
Fah	300.902075	1.63264247	0.70720889	0.17859864	6.26E-06	6.75E-05
Fahd1	178.454033	-1.0290242	-0.0412769	0.12711851	0.68689406	0.8200478
Fahd2a	210.774241	1.02538308	0.036163	0.13027186	0.72937732	0.84829649
Faim	1112.53887	1.00045721	0.00065946	0.0734988	0.99305942	0.9968955
Fam102a	877.014434	1.00963875	0.01383918	0.09761075	0.87063909	0.92983954
Fam102b	2485.78325	1.37627742	0.46077131	0.06663447	8.53E-13	2.77E-11
Fam104a	223.285093	1.13445893	0.18200438	0.1840455	0.14241285	0.30053227
Fam107b	647.147271	-1.3269591	-0.4081239	0.12970901	0.00031232	0.00215795
Fam110a	183.167483	1.0894723	0.12362951	0.15475126	0.27632549	0.47190102
Fam110b	134.775634	-1.0431584	-0.0609583	0.15882456	0.57671553	0.74390554
Fam114a1	2698.37472	1.33782361	0.41988791	0.06206336	2.84E-12	8.64E-11
Fam114a2	633.586515	-1.0391526	-0.0554075	0.07967972	0.45211496	0.6470374
Fam117a	155.259169	-1.0165326	-0.0236565	0.14326184	0.82300539	0.90419748
Fam117b	1534.47034	1.00811544	0.01166085	0.06467664	0.85059218	0.91950091
Fam118a	66.0904225	1.03125871	0.0444063	0.16667012	0.67569485	0.81281465
Fam118b	217.390965	-1.0733524	-0.1021238	0.1242824	0.31758151	0.51633699
Fam120a	18863.6311	-1.0695618	-0.0970198	0.04001771	0.01237031	0.04664814
Fam120b	1209.20932	1.10403716	0.14278873	0.07138486	0.03338063	0.10220027
Fam120c	1357.94073	1.13928819	0.18813274	0.06363443	0.00191931	0.01007226
Fam122a	159.303872	1.01643707	0.0235209	0.13835314	0.82575312	0.90634028
Fam122b	297.244292	-1.1228516	-0.1671673	0.13598644	0.12508785	0.27395714
Fam126a	4583.9759	-2.1808825	-1.1249121	0.05013995	1.01E-114	1.85E-111
Fam126b	155.841131	-1.0365694	-0.0518167	0.15038072	0.63144481	0.78505074
Fam129a	2609.44164	1.12815379	0.17396376	0.07215468	0.01024394	0.04003198
Fam129b	3524.6346	-1.2207237	-0.2877367	0.07403238	3.70E-05	0.00033357
Fam129c	4.54911898	-1.0179781	-0.0257066	0.21298217	0.55249313	0.72865614
Fam131a	193.308208	1.00827582	0.01189035	0.12708491	0.90868502	0.95247976
Fam131b	7.68139594	-1.0275661	-0.0392312	0.21253049	0.48000483	0.67061423
Fam133b	862.566306	-1.1814082	-0.2405075	0.0874114	0.00265743	0.01325613
Fam135a	1541.37863	1.03939096	0.05573841	0.07573061	0.43198965	0.62991319
Fam136a	432.42146	-1.2473957	-0.3189192	0.10476651	0.00068928	0.00426141
Fam13a	174.851957	-2.4723466	-1.305881	0.19743753	2.45E-12	7.53E-11
Fam149a	2370.8914	-1.0032051	-0.0046166	0.06189643	0.93818655	0.97006778
Fam149b	962.167012	1.21375243	0.27947418	0.07985367	0.00017767	0.00135312
Fam151b	492.048888	1.35518009	0.43848459	0.12866815	0.00010987	0.00088463
Fam160a2	698.663261	-1.008803	-0.0126445	0.10026266	0.88780359	0.93988646
Fam160b1	2395.16394	1.14356997	0.19354464	0.06384514	0.00145608	0.00794393
Fam160b2	722.023259	1.03224751	0.04578894	0.09828787	0.60009705	0.76265059
Fam161a	560.516987	1.2331608	0.30236093	0.11505973	0.00264536	0.01321131
Fam161b	334.46276	1.30386007	0.38278905	0.13880794	0.00117218	0.0066441
Fam167a	11.8229809	1.02987847	0.04247411	0.20973629	0.5179493	0.70084827
Fam168a	307.737482	1.1030805	0.14153807	0.13100991	0.18235089	0.35837908
Fam168b	374.158363	1.22189226	0.28911708	0.12035868	0.00533234	0.02328936
Fam171a1	1923.71624	1.06723917	0.09388353	0.06657127	0.13790408	0.29339831
Fam171a2	341.993531	-1.0902975	-0.1247218	0.12783211	0.22383872	0.41221593

Fam172a	2162.30883	1.20026442	0.26335227	0.06380954	1.57E-05	0.00015551
Fam174a	441.752888	1.00134788	0.00194327	0.11798264	0.98537481	0.99212536
Fam174c	33.8609139	-1.0456083	-0.0643425	0.198448	0.49208293	0.67941176
Fam178b	2.2722246	1.01037839	0.01489569	0.21481812	0.53174743	0.71177477
Fam185a	85.9839314	1.0058452	0.00840829	0.17087021	0.93664327	0.96922329
Fam187a	56.5466753	1.5221157	0.60607803	0.44779554	0.00856971	0.03445636
Fam189a1	4.15186611	1.04623575	0.06520797	0.22858682	0.02868251	0.09036472
Fam189a2	27.2219564	-1.0239414	-0.0341332	0.19572665	0.69448499	0.82527263
Fam189b	23.848708	1.05593066	0.0785151	0.21354371	0.35492839	0.55469218
Fam192a	1033.27235	-1.1501155	-0.2017788	0.07845844	0.00567185	0.02448815
Fam193a	2002.08051	1.04657435	0.06567481	0.07740734	0.36323667	0.56391848
Fam193b	315.235533	-1.0323103	-0.0458766	0.14577304	0.67002305	0.80967875
Fam199x	798.184414	-1.1294731	-0.1756499	0.0880451	0.02835348	0.08952626
Fam204a	471.905117	-1.0556763	-0.0781675	0.10984642	0.40758542	0.60672593
Fam207a	813.856949	-1.0650408	-0.0909087	0.09442575	0.28291143	0.47906941
Fam20c	356.132116	-1.2237751	-0.2913384	0.12615332	0.00663525	0.02776584
Fam210a	673.186077	-1.1435113	-0.1934706	0.09117479	0.01911012	0.06601959
Fam210b	217.643962	1.51571368	0.59999726	0.18033497	8.63E-05	0.00071522
Fam214a	315.458593	1.36546199	0.44938916	0.14387277	0.00028916	0.00202618
Fam214b	155.546732	-1.0177092	-0.0253254	0.14326415	0.81143693	0.89723842
Fam216a	590.759957	1.15473867	0.20756639	0.11258214	0.03295684	0.10116872
Fam219a	15.4641654	1.02170607	0.03098022	0.20683057	0.63883259	0.79036561
Fam219b	320.296148	-1.012498	-0.017919	0.13943523	0.86448522	0.92717978
Fam222a	8.10379361	1.06037453	0.08457392	0.2316111	0.13719661	0.29232083
Fam222b	420.845997	1.08886307	0.12282254	0.12968834	0.24361793	0.4337984
Fam227a	10.4567642	1.04774963	0.06729402	0.22175578	0.25988718	0.45214651
Fam228b	120.691144	1.28043196	0.35663059	0.23015743	0.02011302	0.06896354
Fam229a	1.70539178	-1.0124437	-0.0178417	0.21530613	0.43098355	0.62901895
Fam229b	100.752925	1.0453795	0.06402678	0.16257627	0.56097954	0.73388045
Fam234a	1431.17006	1.22834545	0.29671635	0.07004226	8.02E-06	8.48E-05
Fam234b	330.07418	1.09519092	0.13118239	0.11449497	0.17830257	0.35291346
Fam241a	227.401211	-1.0836424	-0.1158888	0.13093566	0.27093688	0.4663041
Fam25c	115.436446	-2.3361565	-1.2241369	0.19700808	3.14E-11	8.42E-10
Fam32a	588.557829	-1.0492849	-0.0694064	0.08944311	0.39233257	0.59362393
Fam3a	654.39995	1.31318056	0.39306529	0.11498785	0.00013038	0.00102657
Fam3c	2998.27262	-1.0729288	-0.1015543	0.06419824	0.09660127	0.22747051
Fam43a	50.8815649	-3.3150504	-1.7290308	0.40404295	8.75E-07	1.14E-05
Fam45a	447.208547	1.15526995	0.20823	0.11384404	0.03398635	0.10368305
Fam50a	934.737448	-1.0600243	-0.0840974	0.07864417	0.24909496	0.44024647
Fam53a	488.983417	1.05380749	0.07561133	0.09298967	0.3684522	0.57003293
Fam53b	455.562805	-1.1339934	-0.1814122	0.10856795	0.0537882	0.14688272
Fam53c	198.481315	1.09563924	0.13177285	0.13923919	0.22834004	0.41720666
Fam57a	214.060305	1.00276426	0.00398248	0.13976436	0.97133532	0.98674112
Fam71e1	5.7015458	-1.0090152	-0.0129478	0.21132486	0.76392385	0.86931372
Fam71e2	2.79932616	-1.0227894	-0.0325091	0.21651337	0.32662988	0.52657281
Fam71f1	13.9145941	-1.0565341	-0.0793393	0.2255548	0.21746362	0.40398842
Fam71f2	6.44731648	1.01041906	0.01495376	0.21047048	0.75491082	0.86381488
Fam72a	74.933315	1.12501679	0.16994653	0.19682269	0.16788455	0.33793228
Fam76b	808.770256	-1.0114375	-0.0164072	0.08333127	0.83409317	0.91104481
Fam78a	231.18323	1.12203299	0.16611509	0.1277082	0.11481145	0.25779749

Fam78b	291.13555	-1.8452677	-0.8838301	0.15748139	1.46E-09	3.00E-08
Fam83c	72.3293374	-2351.3388	-11.199267	3.06095094	4.52E-20	2.72E-18
Fam83d	2018.67434	1.28322076	0.35976939	0.08180731	2.87E-06	3.37E-05
Fam83e	1.90235712	-1.0147916	-0.0211834	0.21607156	0.30517646	0.50332309
Fam83h	14.8956652	-7.0754249	-2.8228168	0.76386761	1.14E-05	0.00011675
Fam89b	132.740568	1.12190119	0.16594562	0.16409537	0.16116317	0.32766479
Fam8a1	445.530563	1.01288719	0.01847351	0.12871286	0.85917483	0.92464333
Fam91a1	10433.7062	-1.3635647	-0.4473832	0.04128983	4.51E-28	4.22E-26
Fam92a	522.040295	1.04014	0.05677772	0.09806131	0.51663193	0.69987892
Fam98b	2834.14622	-1.2175296	-0.2839569	0.05848792	4.67E-07	6.37E-06
Fam98c	145.815074	1.01066909	0.01531071	0.14771436	0.88791813	0.93988646
Fan1	1548.44244	1.04134464	0.05844761	0.07071612	0.38175994	0.58371269
Fanca	736.053279	-1.0245778	-0.0350296	0.09669546	0.68412828	0.81850035
Fancb	502.901939	-1.0711552	-0.0991675	0.08645629	0.20913311	0.39446038
Fancc	126.448753	-1.0942107	-0.1298906	0.16137159	0.25831144	0.45056774
Fancd2	3177.1784	1.13777654	0.18621724	0.05628988	0.00058991	0.00373837
Fancf	30.6956132	-1.0308679	-0.0438594	0.20390293	0.5732896	0.74165217
Fancg	201.100407	-1.0439229	-0.0620151	0.14804692	0.56784981	0.73826815
Fanci	2053.18979	1.07292557	0.10154999	0.06453367	0.09842747	0.23037748
Fancl	499.573247	-1.0674091	-0.0941133	0.10865681	0.31542781	0.51446741
Fancm	1820.56723	1.04278597	0.06044308	0.06205523	0.30733482	0.50575966
Fank1	6.54470359	1.04203527	0.05940411	0.22052366	0.25585517	0.44786892
Fap	6.15495177	1.08382621	0.11613344	0.25875568	0.02231172	0.07499588
Far1	3525.64108	-1.3092726	-0.3887655	0.05646737	1.38E-12	4.36E-11
Farp1	3101.8488	1.28894448	0.36619012	0.0554629	1.07E-11	3.03E-10
Farp2	288.616377	1.22396846	0.29156639	0.14194818	0.01207085	0.04573428
Fars2	761.405779	-1.0070075	-0.0100744	0.08026239	0.89162018	0.94261405
Farsa	2016.89628	1.03239612	0.04599662	0.06378155	0.45032328	0.64583981
Farsb	3692.88368	1.01613956	0.02309857	0.05672194	0.67439956	0.81201789
Fas	402.609992	-1.4188884	-0.5047611	0.12106669	4.33E-06	4.87E-05
Fasn	13165.5646	-1.1958966	-0.2580926	0.06670715	4.58E-05	0.00040479
Fastk	223.869932	-1.7674391	-0.8216605	0.20659487	4.79E-06	5.31E-05
Fastkd1	1054.30354	-1.0337656	-0.0479091	0.08924715	0.55431494	0.72987441
Fastkd2	1447.37434	1.02142112	0.0305778	0.08302433	0.69042346	0.82279048
Fastkd3	765.007676	1.01434496	0.02054837	0.10002463	0.8178305	0.90166143
Fastkd5	792.29858	-1.2404658	-0.3108819	0.10319473	0.00079664	0.00482506
Fat1	27280.3649	-1.0306266	-0.0435217	0.0512118	0.37072765	0.57213508
Fat4	14.3246231	-1.0404261	-0.0571745	0.21261562	0.41331563	0.61223094
Fbf1	918.026215	1.04909372	0.06914356	0.08836741	0.38929976	0.59099561
Fbh1	3124.68419	1.01612261	0.0230745	0.05251025	0.65079451	0.79746983
Fblim1	180.1018	1.15549745	0.20851407	0.15334679	0.07727446	0.19303545
Fbln2	2.98290042	1.00372406	0.00536271	0.21308445	0.86952472	0.92935896
Fbn1	22.9766095	-1.0079867	-0.0114766	0.19292238	0.89277246	0.94359862
Fbrs	1325.98174	-1.0337371	-0.0478693	0.08689285	0.54653473	0.72332988
Fbrsl1	182.476	-1.1637409	-0.2187699	0.16497246	0.07258983	0.18398807
Fbxl12	405.739272	-1.0680164	-0.0949337	0.11545947	0.33087899	0.53094825
Fbxl13	10.7090261	-1.0263014	-0.0374545	0.21057797	0.53081984	0.71104678
Fbxl14	238.717959	1.05512634	0.07741576	0.12518546	0.4500899	0.64570677
Fbxl15	156.279241	1.00584461	0.00840744	0.15650132	0.93749921	0.96972611
Fbxl16	1159.04318	1.21864491	0.28527781	0.08540439	0.00030774	0.00212881

Fbxl17	3149.20885	1.18372773	0.24333728	0.05795568	1.28E-05	0.00012916
Fbxl18	422.561043	1.0435852	0.06154839	0.12786113	0.54820304	0.72501352
Fbxl19	956.45148	-1.0480489	-0.067706	0.08306471	0.37556294	0.57740773
Fbxl2	53.4410325	1.00914798	0.01313775	0.18060509	0.89593804	0.94546226
Fbxl20	424.624898	1.15091418	0.20278026	0.11333351	0.03754439	0.11205583
Fbxl3	1172.5944	-1.0450511	-0.0635735	0.09810144	0.46520288	0.65833506
Fbxl4	325.930588	1.35435664	0.43760769	0.12584415	8.56E-05	0.00070939
Fbxl5	584.650095	1.13515211	0.18288563	0.09257384	0.02879488	0.09065185
Fbxl6	250.945043	-1.0403598	-0.0570825	0.12397163	0.57288319	0.7414648
Fbxl8	129.787574	1.01537921	0.02201863	0.15424261	0.83930109	0.91408323
Fbxo10	94.3996424	-1.0138709	-0.019874	0.1592551	0.85175603	0.92037071
Fbxo11	3473.265	-1.1716577	-0.2285512	0.06326335	0.00015307	0.00118266
Fbxo15	73.5253012	-1.0839208	-0.1162594	0.18519302	0.30830708	0.50652991
Fbxo21	2559.7664	-1.0196636	-0.0280933	0.05354913	0.58730211	0.75090728
Fbxo22	672.663417	1.0260997	0.03717092	0.09558813	0.66457305	0.80579089
Fbxo24	18.3398728	-1.0315152	-0.0447651	0.20169698	0.5831507	0.74830245
Fbxo25	390.155155	1.00157527	0.00227085	0.11310717	0.98212717	0.99108517
Fbxo27	207.181757	1.03125529	0.04440152	0.14008786	0.6785296	0.81499755
Fbxo3	2495.6673	1.11815786	0.16112388	0.06432707	0.00846812	0.03412287
Fbxo30	675.777561	-1.0431596	-0.06096	0.10176509	0.50099837	0.68632116
Fbxo31	873.815227	1.04308326	0.06085432	0.07806227	0.40260396	0.60235421
Fbxo32	278.968201	-1.9757725	-0.9824168	0.17163596	6.63E-10	1.44E-08
Fbxo33	1180.21866	-1.1342618	-0.1817536	0.08045237	0.01446515	0.05300303
Fbxo34	771.198104	-1.0309302	-0.0439466	0.09590067	0.6037541	0.7655241
Fbxo36	47.939228	1.30858571	0.38800842	0.38519599	0.02579841	0.08351723
Fbxo38	517.613677	1.0843556	0.11683794	0.09165806	0.15816872	0.32381994
Fbxo4	584.245387	-1.1170987	-0.1597566	0.09784076	0.06651594	0.17311442
Fbxo42	2508.73995	-1.0806668	-0.1119218	0.06011045	0.05008842	0.139213
Fbxo45	225.730291	-1.0921835	-0.1272152	0.12575358	0.21748078	0.40398842
Fbxo46	104.346984	-1.0193276	-0.0276178	0.14343739	0.79567966	0.88630303
Fbxo47	123.486511	1.23962249	0.30990084	0.19869171	0.026964	0.08633062
Fbxo48	41.413209	1.07060438	0.09842547	0.20689068	0.33531263	0.53518002
Fbxo5	973.743368	-1.1846779	-0.2444949	0.08606098	0.00198781	0.01038121
Fbxo6	1685.00726	1.01126644	0.01616316	0.09339897	0.85087946	0.91973385
Fbxo7	795.942868	1.10172227	0.13976059	0.09271684	0.09482412	0.2244866
Fbxo8	493.129082	-1.0019457	-0.0028044	0.10197119	0.97448188	0.98844825
Fbxo9	847.362187	-1.0675569	-0.094313	0.09219898	0.2562178	0.44831991
Fbxw10	175.947201	-1.4058016	-0.491393	0.19376065	0.00135264	0.00746548
Fbxw11	3725.35274	1.05249001	0.07380654	0.05279291	0.14905453	0.30990345
Fbxw4	270.021378	1.40766915	0.4933083	0.16171699	0.00029404	0.00205267
Fbxw5	914.735572	1.10784395	0.14775468	0.08908106	0.06670138	0.17342851
Fbxw7	1862.02334	-1.0650477	-0.090918	0.06274738	0.12908702	0.27970309
Fbxw8	2204.82715	1.27377547	0.34911099	0.06419183	1.52E-08	2.70E-07
Fbxw9	257.914287	1.31363099	0.39356007	0.21769153	0.01065159	0.04141028
Fcer2a	113.888367	-1.7165634	-0.7795232	0.26666767	0.00022844	0.0016731
Fcf1	829.418543	1.01974217	0.02820442	0.08179532	0.71021064	0.83657916
Fcgbp	42.3308021	-11.861892	-3.5682623	0.45439719	4.55E-16	2.01E-14
Fcgrt	137.478303	1.05383382	0.07564738	0.15127504	0.49256461	0.67949091
Fcho1	105.928097	1.04619399	0.06515038	0.1702122	0.55036966	0.72692841
Fcho2	1761.83363	1.17825946	0.23665727	0.06462491	0.00012142	0.00096607

Fchsd1	405.667135	1.14258236	0.19229817	0.1112138	0.04580524	0.13038845
Fchsd2	967.338474	1.28093665	0.35719913	0.08308094	4.50E-06	5.04E-05
Fcmr	2.15164072	1.00058158	0.0008388	0.21325553	0.9789715	0.99034912
Fcsk	493.24253	1.05167024	0.07268241	0.13545875	0.50219701	0.68715484
Fdft1	895.10772	-1.056675	-0.0795317	0.09802775	0.36078508	0.5610541
Fdps	2359.4977	1.14656449	0.1973175	0.07445821	0.00466981	0.02096013
Fdx1	382.448052	-1.0913715	-0.1261423	0.13419135	0.23916649	0.42897266
Fdxacb1	138.951842	1.11594886	0.15827092	0.17799316	0.18890416	0.36714645
Fech	777.971964	1.0898005	0.12406406	0.08763119	0.12050263	0.2664202
Fem1b	7613.19918	-1.0800762	-0.1111331	0.04532916	0.01197071	0.04546242
Fem1c	983.534423	-1.144438	-0.1946393	0.08932839	0.01647959	0.05862359
Fen1	855.219535	-1.1140556	-0.1558213	0.07887165	0.03273771	0.10061663
Fer	1710.43158	1.07442043	0.10355864	0.06494662	0.09391791	0.22291799
Fer1l4	46.2254914	1.04293587	0.06065045	0.18959064	0.54693712	0.72378765
Fer1l5	1.82053183	1.00815289	0.01171445	0.21441264	0.65014072	0.79726959
Fer1l6	24.4266091	-6.8902254	-2.7845512	0.54585009	2.43E-08	4.19E-07
Fermt2	10501.1911	-1.0826782	-0.1146046	0.03954937	0.00320382	0.01542311
Fermt3	3.8149718	-1.0111792	-0.0160387	0.21229626	0.6905741	0.82279048
Fes	75.0937156	1.44381814	0.52988904	0.26582092	0.00429504	0.01956597
Fez2	2236.10318	1.00506773	0.00729272	0.07035814	0.91340263	0.95523619
Fgb	4.13214803	1.00165383	0.002384	0.21268847	0.94603124	0.97323002
Fgd1	1318.9569	1.08226655	0.11405587	0.06006592	0.04756799	0.13406951
Fgd3	57.3662005	-1.1695243	-0.2259219	0.24536264	0.09102642	0.21791226
Fgd4	413.262494	-1.1861897	-0.2463347	0.11092609	0.01093133	0.04219664
Fgd5	7.5759381	-1.0388031	-0.0549222	0.21896599	0.28529909	0.48189364
Fgd6	526.757558	-1.2307204	-0.299503	0.13033456	0.00649976	0.02729006
Fgf13	139.336043	-1.0859913	-0.1190126	0.16771505	0.29910922	0.49689911
Fgf18	120.547203	-1.1316569	-0.1784366	0.18145761	0.14780041	0.30829773
Fgf7	59.8402343	-25.471391	-4.6708059	0.4333655	2.64E-27	2.37E-25
Fgf8	2.38622579	-1.0156872	-0.0224561	0.21629607	0.26416021	0.45767849
Fgfr1	2438.47668	-1.2688427	-0.3435133	0.06079285	4.52E-09	8.72E-08
Fgfr1op2	1611.53117	1.01565183	0.02240592	0.08215071	0.76874388	0.87144584
Fgfr2	2.03084863	-1.0059214	-0.0085175	0.21400991	0.75006628	0.86119468
Fgfr3	208.970194	1.32228511	0.40303328	0.15438951	0.00162806	0.00875543
Fgfrl1	712.227673	1.18611838	0.24624801	0.09111078	0.00298501	0.01452813
Fggy	253.051211	1.2217535	0.28895324	0.16043058	0.02050868	0.0700194
Fgl2	7.10454508	1.02593816	0.03694378	0.21173942	0.51681139	0.69997398
Fh1	3773.95364	-1.1574345	-0.2109305	0.05118902	2.12E-05	0.00020084
Fhad1	7.7047968	1.00268626	0.00387026	0.21005679	0.93631461	0.96922329
Fhdc1	332.704996	1.16495038	0.22026851	0.11062142	0.02219156	0.07467136
Fhl1	1420.89956	-1.1551121	-0.2080329	0.0914427	0.0119389	0.04537536
Fhl2	1262.01684	-1.3722742	-0.4565688	0.08250991	5.35E-09	1.02E-07
Fhl3	299.000945	-1.1191175	-0.1623615	0.16616966	0.17016361	0.34128634
Fhl4	5.95257133	1.01820282	0.02602497	0.21340197	0.54005591	0.71845252
Fhod1	778.256052	-1.117078	-0.15973	0.08201302	0.03444266	0.10475084
Fhod3	12.3975767	-1.0444747	-0.0627775	0.21435584	0.37994902	0.58210352
Fibp	653.441712	1.19355091	0.2552601	0.09282148	0.0024621	0.0124613
Ficd	145.08132	1.23002208	0.29868421	0.17693384	0.02359072	0.07822435
Fig4	1695.38361	1.12926786	0.17538773	0.06189202	0.00299675	0.01457417
Fign	318.959893	-1.0502775	-0.0707706	0.11601511	0.46920367	0.66135814

Fignl1	616.335221	1.23466343	0.30411782	0.09183587	0.00030734	0.00212815
Fignl2	39.3868434	-1.121655	-0.165629	0.24695015	0.14127327	0.2987307
Filip1	2.77479915	-1.0062097	-0.0089309	0.21349647	0.76879074	0.87144584
Filip1l	1471.3891	1.63507001	0.70935241	0.1197647	3.00E-10	6.88E-09
Fip1l1	4379.68293	-1.0438313	-0.0618886	0.06534772	0.31917252	0.51762959
Fis1	746.765479	1.09800896	0.13488982	0.0937386	0.10924067	0.24864481
Fitm2	2462.85016	1.41348417	0.49925573	0.05958589	8.93E-18	4.52E-16
Fiz1	288.343844	1.06803856	0.09496373	0.13785777	0.37366292	0.57517687
Fjx1	49.9097754	-1.5788253	-0.6588515	0.37754029	0.0043001	0.01958206
Fkbp10	5762.09237	1.37275197	0.45707098	0.05872365	1.31E-15	5.56E-14
Fkbp11	1038.95806	1.02628378	0.0374297	0.07618585	0.60023253	0.76271984
Fkbp14	1666.29135	-1.4846436	-0.5701166	0.07225482	3.79E-16	1.69E-14
Fkbp15	1132.00621	1.06708736	0.09367828	0.08141411	0.2134364	0.39931654
Fkbp1a	16749.9523	-1.0106865	-0.0153356	0.04627352	0.74032174	0.85552742
Fkbp1b	3.28761006	-1.0521233	-0.0733038	0.23194972	0.03187341	0.0984229
Fkbp2	801.762691	1.07885314	0.10949849	0.0938437	0.19474568	0.37469093
Fkbp4	6693.78382	-1.1009192	-0.1387085	0.05905766	0.01433474	0.05261549
Fkbp5	2285.46228	-1.100091	-0.1376228	0.05269162	0.00690565	0.02873057
Fkbp7	446.874674	1.19251662	0.25400937	0.12812095	0.01800025	0.06318077
Fkbp8	2367.16071	1.08126179	0.11271587	0.08378304	0.14334857	0.3018472
Fkbp9	6681.24992	-1.6145119	-0.6910981	0.05437529	5.52E-38	8.04E-36
Fkbpl	115.303218	-1.0372239	-0.0527273	0.17747595	0.6134111	0.77269695
Fktn	624.029478	-1.0860379	-0.1190744	0.09270451	0.1534727	0.31638
Flad1	124.587448	-1.1045824	-0.143501	0.1572976	0.21287245	0.39878562
Flcn	264.452554	1.09620591	0.13251881	0.14263518	0.23091835	0.42000057
Flii	3889.68973	1.25795958	0.33108557	0.04590693	1.75E-13	6.07E-12
Flna	41930.8137	1.0569586	0.07991887	0.04070049	0.04551292	0.12976198
Flnb	10744.8389	1.12896693	0.17500323	0.03917763	5.31E-06	5.81E-05
Flnc	117.008737	-1.0981576	-0.1350851	0.15503028	0.23653024	0.42605621
Flot1	1235.34743	1.07927262	0.11005933	0.09010364	0.17787284	0.35217173
Flot2	1091.25667	1.10985326	0.15036894	0.07971807	0.04154698	0.12106842
Flrt3	411.364473	-1.7817585	-0.8333018	0.14147259	2.99E-10	6.88E-09
Flt1	10.1007296	-4.9358277	-2.303292	0.94622001	0.00051148	0.00332413
Flt3l	48.8118941	-1.0236566	-0.0337318	0.18898922	0.71751685	0.84141289
Flvcr1	464.421212	-1.0498931	-0.0702424	0.0973399	0.41171026	0.61091259
Flvcr2	6.16551149	1.04523052	0.06382116	0.22339942	0.1984927	0.37944957
Flywch1	915.978348	-1.0821771	-0.1139366	0.07969329	0.12181629	0.26844428
Fmc1	263.630888	1.05153542	0.07249744	0.1470021	0.5083132	0.69256457
Fmn1	20.9588793	-1.0261877	-0.0372946	0.20127033	0.63978717	0.79058554
Fmnl1	3.62115088	-1.0355207	-0.0503564	0.22103961	0.16965013	0.34062981
Fmnl2	2310.15907	1.16184049	0.21641201	0.06504969	0.00047445	0.00310565
Fmnl3	2100.52049	-1.011489	-0.0164807	0.05583187	0.75936923	0.86662603
Fmo2	37.223087	1.07093992	0.09887755	0.21083472	0.31627193	0.51518863
Fmo4	353.751058	1.59255816	0.67134606	0.16418476	4.01E-06	4.52E-05
Fmo5	427.882946	1.43022885	0.51624601	0.13582387	1.88E-05	0.00018146
Fmod	1.97066826	1.00547965	0.00788389	0.21371501	0.78256464	0.87835389
Fmr1	1739.74272	-1.4694347	-0.5552613	0.07505968	1.89E-14	7.08E-13
Fn1	17932.7296	1.47948069	0.56509087	0.07522923	7.91E-15	3.09E-13
Fn3k	11.5689408	1.09026523	0.12467915	0.26297806	0.03537386	0.1067717
Fn3krp	1221.31817	1.14982194	0.20141047	0.07422909	0.00375555	0.01755149

Fnbp1	1096.76957	1.09337275	0.12878532	0.07612806	0.06977996	0.17885163
Fnbp1l	2826.1823	1.46496053	0.55086179	0.06829772	1.02E-16	4.74E-15
Fnbp4	2960.94864	-1.0727224	-0.1012768	0.06511162	0.10158506	0.23591455
Fndc3a	2261.80182	-1.0323452	-0.0459255	0.06038798	0.42730274	0.62541437
Fndc3b	4665.07642	1.12616709	0.17142089	0.05420785	0.00107029	0.00617029
Fndc4	93.2041677	1.68469715	0.75248927	0.25714602	0.00023488	0.00170958
Fnip1	697.982995	1.18235405	0.2416621	0.08027964	0.00119604	0.00674056
Fnip2	800.085874	-1.013325	-0.019097	0.09910479	0.8395459	0.9142043
Fnta	3439.53284	-1.0608048	-0.0851592	0.05687201	0.11954259	0.26498365
Fntb	2228.13571	-1.0288843	-0.0410808	0.05894226	0.4639992	0.65728516
Focad	1188.49196	1.03968006	0.05613964	0.06915337	0.39083453	0.59242578
Folh1	188.058078	1.05994131	0.08398439	0.14933673	0.44723174	0.64330021
Fopnl	574.582367	1.0812292	0.11267238	0.10485714	0.2180212	0.40452658
Fos	21.7175367	-1.0114355	-0.0164043	0.1965847	0.8383267	0.91364345
Fosb	24.3318904	-1.0319399	-0.0453589	0.20480395	0.55567616	0.73050512
Fosl1	3753.35872	-1.5731319	-0.6536397	0.07062467	2.18E-21	1.43E-19
Fosl2	3038.5957	-1.0933952	-0.128815	0.09046702	0.11571547	0.25901017
Foxa1	38.3989916	-1.3105192	-0.3901385	0.41192576	0.02550699	0.08282497
Foxc1	118.016264	1.01136655	0.01630596	0.1638544	0.87997299	0.93572664
Foxd2	6.05020497	-1.0386152	-0.0546612	0.22020286	0.2442944	0.43451976
Foxf1	777.47398	-1.3658133	-0.4497602	0.09650345	5.47E-07	7.36E-06
Foxf2	356.97597	-1.1193238	-0.1626274	0.14189021	0.14289917	0.30111192
Foxh1	14.7436198	1.02679344	0.03814599	0.20668696	0.58601502	0.74971062
Foxj1	1.65361475	1.01998476	0.02854759	0.2171876	0.21208899	0.39786559
Foxj2	2014.66314	-1.0139998	-0.0200574	0.06096389	0.73101255	0.84921291
Foxj3	1816.44235	1.19166788	0.25298221	0.06616289	5.92E-05	0.00051052
Foxk1	588.030311	-1.1066159	-0.1461546	0.09340932	0.0818409	0.20137956
Foxk2	3173.05406	1.0439197	0.06201074	0.0584818	0.26930801	0.46424917
Foxl1	7.69534204	-1.0488047	-0.0687461	0.22444591	0.19264118	0.37164661
Foxm1	3322.68383	1.19784919	0.26044628	0.06316882	1.63E-05	0.00016084
Foxn2	3793.49188	-1.0546451	-0.0767576	0.0672264	0.2270757	0.4157267
Foxn3	472.27278	-1.0162382	-0.0232386	0.09565841	0.77956201	0.87680583
Foxo1	2147.33225	-1.1310528	-0.1776663	0.05963826	0.00186977	0.00986074
Foxo3	3208.86629	1.07366782	0.10254771	0.06121519	0.08005948	0.19798494
Foxo4	1670.97885	-1.1392687	-0.1881081	0.0685571	0.0036715	0.01725943
Foxp1	2040.34462	-1.0024479	-0.0035273	0.05820283	0.94918689	0.97475422
Foxp2	413.582198	1.07247748	0.10094736	0.11099915	0.28931742	0.48574196
Foxq1	3.55161815	-1.0616509	-0.0863095	0.23965932	0.01626264	0.05806761
Foxred1	472.330716	-1.0515802	-0.0725589	0.10199405	0.41860229	0.61733754
Foxs1	236.813685	1.04245864	0.05999015	0.14036603	0.57697735	0.74394362
Fpgs	811.735417	-1.1524126	-0.2046573	0.0941411	0.01571497	0.05660949
Fpgt	793.928401	-1.0871153	-0.1205049	0.09686402	0.16237529	0.32963986
Fra10ac1	934.683862	1.16587293	0.22141056	0.08359841	0.00409279	0.01883769
Frem1	89.2823883	1.410564	0.49627213	0.27381756	0.00655266	0.02747614
Frg1	1011.44011	-1.0842879	-0.1167479	0.08342724	0.12774655	0.27778532
Frk	1.81042222	-1.0076609	-0.0110102	0.21392494	0.70029329	0.82955031
Frmd4a	2459.68224	-1.0483906	-0.0681763	0.05623184	0.20844253	0.39347905
Frmd4b	95.0090869	-2.6812453	-1.4229032	0.27673471	1.29E-08	2.33E-07
Frmd5	1.96002984	1.01137072	0.01631192	0.21531729	0.43766374	0.63515039
Frmd6	1453.35808	-1.289966	-0.367333	0.09060754	1.21E-05	0.00012385

Frrs1	138.817407	1.02715951	0.03866025	0.16126102	0.72206954	0.84343968
Frs2	1605.99891	-1.1130169	-0.1544756	0.06697612	0.01475301	0.0539036
Frs3	53.6065082	1.40387486	0.48941434	0.3036893	0.00938396	0.03708996
Fry	1680.52736	1.25343749	0.32589004	0.07597431	5.51E-06	6.01E-05
Fryl	3498.56994	-1.0447378	-0.0631409	0.05435792	0.22928575	0.41786178
Fsbp	106.945497	1.01152904	0.01653774	0.15708294	0.87918015	0.93525426
Fscn1	126.765002	-1.8045922	-0.8516729	0.19357539	7.72E-07	1.02E-05
Fscn3	2.64017846	1.02159686	0.030826	0.21676976	0.29881455	0.49666724
Fsd1	103.438443	1.11685101	0.15943675	0.18692601	0.189279	0.36759611
Fsd1l	157.929078	-1.2419495	-0.3126065	0.18952726	0.0228265	0.07622719
Fsip1	40.5102486	1.80047395	0.84837672	0.47758939	0.00297929	0.01450579
Fst	1320.54684	-1.387344	-0.4723255	0.07989398	5.63E-10	1.23E-08
Fstl1	23317.1744	1.63235105	0.70695136	0.05361541	1.07E-40	1.71E-38
Fstl3	13.4869306	1.04287626	0.06056799	0.21507482	0.37243353	0.57411233
Fto	2161.04392	1.09702208	0.13359256	0.06420833	0.0289282	0.09095981
Ftsj1	482.478209	-1.1688447	-0.2250832	0.14443387	0.04910675	0.1373488
Fubp1	2698.77095	1.04545339	0.06412874	0.07226323	0.34540363	0.54470275
Fubp3	2249.35732	-1.0984014	-0.1354053	0.06916539	0.0379894	0.11306864
Fuca1	5006.0329	-1.1919634	-0.2533399	0.05864784	7.06E-06	7.55E-05
Fundc2	2026.70448	1.13516784	0.18290562	0.06027261	0.00153389	0.00831182
Fuom	2.20849871	-1.0013629	-0.0019649	0.21374271	0.93895585	0.97054583
Furin	5489.66478	-1.0042797	-0.0061611	0.07164483	0.92673923	0.96281667
Fus	15253.6732	-1.1129057	-0.1543313	0.0506175	0.00166681	0.00894132
Fut11	722.694019	1.02410601	0.03436506	0.0967799	0.6918782	0.82354207
Fut8	2557.93634	-1.1157749	-0.158046	0.06441317	0.00982837	0.03869126
Fuz	292.006136	1.11716052	0.15983649	0.12002247	0.11310105	0.25480406
Fxn	726.64098	1.05435055	0.07635461	0.08856989	0.3419548	0.5415306
Fxr1	7634.64026	1.03510935	0.04978318	0.05676228	0.36644346	0.56767937
Fxr2	1997.70178	1.03012329	0.04281701	0.05849308	0.44677805	0.64302826
Fxyd5	1762.43197	-1.003646	-0.0052505	0.07509752	0.93974106	0.97089144
Fyb	26.3402434	-1.951177	-0.9643446	0.6750554	0.0044634	0.02020358
Fyco1	2686.69984	-1.0224838	-0.032078	0.05844362	0.56793962	0.73830998
Fyn	2661.48375	1.22423465	0.2918801	0.06422146	2.05E-06	2.47E-05
Fyttd1	3839.19522	1.01897911	0.02712448	0.0484124	0.56569377	0.73677912
Fzd1	137.660411	-1.0312683	-0.0444197	0.16079945	0.68290657	0.81734403
Fzd2	450.336503	1.61667551	0.69303014	0.15792063	9.78E-07	1.27E-05
Fzd3	285.128629	-1.0334773	-0.0475067	0.11948401	0.63151446	0.78505074
Fzd4	388.036901	-1.0771096	-0.107165	0.10705497	0.24831376	0.43923403
Fzd5	82.149372	-1.0924269	-0.1275368	0.19317445	0.26757497	0.46194547
Fzd7	435.072591	-1.1501505	-0.2018227	0.14439173	0.07525147	0.18917986
Fzr1	2103.38232	1.02025998	0.02893682	0.06006988	0.61497837	0.77376891
G2e3	2738.32338	1.10203471	0.14016967	0.07142909	0.03693112	0.11056032
G3bp2	4854.8839	-1.2249435	-0.2927153	0.05179365	5.88E-09	1.12E-07
G6PDH	919.342969	1.02115805	0.03020618	0.08920397	0.71050129	0.83674589
G6pc3	1334.89044	1.06319539	0.08840675	0.08549416	0.25900285	0.45128217
GADD45A	194.295636	-2.2779463	-1.1877338	0.20170711	2.10E-10	4.94E-09
GATB	768.471242	-1.000152	-0.0002193	0.08146537	0.99933036	0.99979674
GFM1	7983.46382	-1.0305444	-0.0434067	0.04060763	0.27625838	0.47184932
GMPR	758.872417	-1.0653873	-0.0913779	0.08921567	0.2585917	0.45087242
GOSR1	1554.9632	1.10537943	0.14454168	0.06726976	0.02341283	0.07775948

GPI	3389.78464	-1.1240989	-0.168769	0.04940839	0.00043219	0.00286412
Gaa	7692.71437	1.40488996	0.49045714	0.07162819	1.21E-12	3.86E-11
Gab1	458.103232	1.06674015	0.09320879	0.10167378	0.29778097	0.49565674
Gab3	72.5539609	-1.0569601	-0.0799209	0.17293619	0.46930097	0.66135814
Gabarap	719.618188	1.45008971	0.53614215	0.10181579	1.90E-08	3.32E-07
Gabarapl1	821.057503	1.00371716	0.00535278	0.0897227	0.94853114	0.9744714
Gabbr1	419.703714	-1.0299087	-0.0425164	0.12239759	0.6720409	0.81086907
Gabpa	4702.21412	1.00640427	0.00920994	0.05733731	0.86325963	0.92665838
Gabpb1	948.409768	1.05348058	0.07516372	0.08731254	0.3466886	0.54632561
Gabpb2	591.25769	-1.1334955	-0.1807786	0.08300408	0.0179828	0.06314685
Gabrq	1.94249396	1.00598194	0.00860441	0.21420284	0.72724504	0.84644821
Gad2	18.2622487	1.00487954	0.00702257	0.19437372	0.93505915	0.96855404
Gadd45b	218.038818	1.11421724	0.15603055	0.15569194	0.17737207	0.35150623
Gadd45gip1	583.207726	-1.0134557	-0.019283	0.09483383	0.82037859	0.90334055
Gak	4933.43034	1.0863263	0.11945751	0.04583878	0.00753814	0.03090033
Galc	572.257302	-1.0855816	-0.1184682	0.09812567	0.17456986	0.3478381
Gale	320.843541	1.11028409	0.15092886	0.13753792	0.16651539	0.33559821
Galk1	1049.60616	1.3905841	0.475691	0.09293774	5.16E-08	8.41E-07
Galk2	255.315505	1.147883	0.1989756	0.14289663	0.07808998	0.19480674
Galm	621.35594	-1.1868157	-0.2470959	0.09275916	0.00333972	0.01596933
Galns	365.500721	1.29546978	0.37347536	0.14389436	0.00196818	0.0103028
Galnt1	3603.04644	-1.1184248	-0.1614683	0.05357931	0.00184121	0.00974624
Galnt10	480.837419	1.4756176	0.5613189	0.12868408	1.51E-06	1.89E-05
Galnt11	852.049412	-1.6714442	-0.7410952	0.08863038	5.57E-18	2.87E-16
Galnt13	168.401369	1.52311649	0.60702628	0.20377998	0.00027812	0.00196512
Galnt7	3283.90219	-1.1534399	-0.2059428	0.06373804	0.0006748	0.00418403
Galt	411.123725	1.05301589	0.07452721	0.10624112	0.42044083	0.61905069
Gamt	123.227017	1.04799973	0.06763835	0.15088312	0.53828784	0.71691387
Gan	834.340416	-1.1417566	-0.1912551	0.07919505	0.00916318	0.03638574
Ganab	5182.46512	1.07501389	0.10435531	0.06684861	0.0976028	0.22899118
Gap43	3.6994717	1.03174245	0.04508288	0.21924312	0.2255458	0.41412563
Gapvd1	7416.45766	1.00172629	0.00248837	0.0452688	0.86262258	0.92665838
Gar1	1444.86513	-1.2892733	-0.3665582	0.07539294	2.99E-07	4.22E-06
Garem1	504.29726	1.0476545	0.06716301	0.08794878	0.40257589	0.60235421
Garem2	6.25417853	1.0338609	0.04804209	0.21591253	0.37347976	0.57503314
Garnl3	62.9847187	1.34614801	0.42883704	0.36470962	0.01982342	0.06807989
Gars	7864.41228	-1.6589089	-0.7302347	0.07597062	6.62E-23	4.66E-21
Gart	6899.64808	-1.0909319	-0.125561	0.04574609	0.00487284	0.02165145
Gas1	5.47758482	1.05819429	0.08160454	0.2347502	0.05226704	0.14380171
Gas2	314.579233	1.04859141	0.06845263	0.12158174	0.49731772	0.68369178
Gas2l1	2867.1422	1.0255622	0.03641499	0.05883409	0.5165666	0.69986443
Gas2l3	826.68221	-1.0757523	-0.1053459	0.0790471	0.15064856	0.31236777
Gas8	213.258118	1.13151713	0.17825842	0.14789409	0.11837338	0.26307478
Gask1b	1504.54064	-1.1165651	-0.1590674	0.09023912	0.05144977	0.14203889
Gata2	1102.19884	-1.0497829	-0.070091	0.09484984	0.40903603	0.6082126
Gata3	283.811839	-1.5896418	-0.6687018	0.13008681	2.55E-08	4.38E-07
Gata4	1003.7414	-1.0676588	-0.0944507	0.08472219	0.2233282	0.41157161
Gata6	363.341956	-1.0617258	-0.0864113	0.13799745	0.42009943	0.61877356
Gatad1	2407.89167	1.02216494	0.03162801	0.06402474	0.60513115	0.76620837
Gatad2a	6028.62835	-1.068883	-0.0961039	0.0489973	0.04375385	0.12607469

Gatad2b	6377.81504	1.04991165	0.07026793	0.04835683	0.13533304	0.2894184
Gatd1	44.295138	-1.1347984	-0.1824361	0.25669218	0.11986141	0.26541481
Gatd3a	889.603945	1.05625625	0.07895988	0.08387332	0.30279791	0.50059975
Gatm	3.28903044	1.01010275	0.01450206	0.21350772	0.66087127	0.80366062
Gba	1224.25518	1.23495061	0.30445334	0.08163041	6.33E-05	0.00054198
Gba2	194.697589	-1.0876209	-0.1211758	0.14725812	0.27617219	0.47182793
Gbe1	588.44437	1.04737396	0.06677664	0.11138557	0.48433714	0.67336397
Gbf1	4880.61821	1.17179165	0.22871608	0.04568199	2.87E-07	4.08E-06
Gbgt1	213.641848	1.39049169	0.47559512	0.16600648	0.00058594	0.0037213
Gbp2	428.302955	-1.1606846	-0.2149761	0.13414156	0.04912447	0.13736837
Gbp5	3.99587812	1.00344631	0.00496343	0.21284401	0.88271137	0.93698481
Gcc1	848.350694	1.27253673	0.3477073	0.08123722	5.14E-06	5.65E-05
Gcc2	4152.25451	1.00472816	0.00680521	0.05919513	0.90529768	0.95071842
Gcdh	411.43198	1.01303848	0.01868898	0.10530112	0.83970008	0.9142043
Gcfc2	846.025069	-2.0125876	-1.0090516	0.09995002	4.31E-25	3.52E-23
Gch1	2352.95159	-1.2211213	-0.2882065	0.07074863	1.67E-05	0.00016406
Gclc	2204.26549	-1.0973758	-0.1340576	0.05396237	0.01000707	0.03928613
Gclm	1237.70613	-1.1374919	-0.1858562	0.08742971	0.01975269	0.0679098
Gcn1	4534.92809	1.00179	0.00258012	0.05534155	0.96195253	0.98195967
Gda	3.05041043	-1.0038283	-0.0055126	0.21310302	0.86148092	0.92611368
Gdap2	1159.80904	1.01538331	0.02202445	0.07665538	0.75904863	0.86645989
Gde1	888.17517	-1.0116007	-0.01664	0.10782903	0.85713414	0.9238442
Gdf11	545.327358	1.07792401	0.10825548	0.11191727	0.25846559	0.45071389
Gdf9	15.3977397	1.1411477	0.19048553	0.3157641	0.04958641	0.13829782
Gdi1	968.541518	1.04948884	0.06968682	0.09099837	0.39740074	0.59757055
Gdpd1	9.66029465	-1.0289189	-0.0411292	0.21536185	0.39298809	0.59407333
Gdpd3	2.60863146	-1.0034349	-0.004947	0.21339774	0.86758109	0.92836933
Gdpd5	167.460901	-1.0049137	-0.0070717	0.13406986	0.94512571	0.97295716
Gdpgp1	158.043881	-1.1556482	-0.2087023	0.15299914	0.0747931	0.18832124
Gem	160.135161	-1.4918763	-0.5771279	0.23906319	0.00137957	0.00759445
Gemin2	690.983816	-1.1081774	-0.1481888	0.09489508	0.08118525	0.2001122
Gemin4	3759.99292	1.10225238	0.1404546	0.05315964	0.0062842	0.02659419
Gemin5	1790.16573	-1.0262458	-0.0373763	0.06738847	0.55858817	0.73247154
Gemin6	879.908154	1.15039202	0.20212557	0.1104341	0.03490565	0.10580159
Gemin7	33.5320767	1.03137364	0.04456708	0.19713431	0.61625208	0.77428768
Gemin8	926.809133	-1.0598591	-0.0838725	0.09158519	0.31013183	0.50848442
Get1	274.474211	1.0632968	0.08854436	0.11850896	0.37330703	0.57484647
Get4	521.426792	1.08403854	0.11641605	0.1312678	0.27043718	0.46572403
Gfer	569.491493	1.18415874	0.24386249	0.09898527	0.00600768	0.02558455
Gfm2	1524.85608	-1.0970614	-0.1336443	0.07873291	0.06694723	0.17383385
Gfod1	304.125988	1.66414163	0.73477823	0.13932762	1.23E-08	2.22E-07
Gfod2	226.872076	-1.0718034	-0.1000403	0.15823623	0.3745936	0.57633245
Gfpt2	10.4893818	-1.0385061	-0.0545097	0.21801605	0.31168957	0.51031997
Gfra1	21.7455634	-1.0665704	-0.0929792	0.21403976	0.31746649	0.51628103
Gga1	912.751894	1.12968114	0.17591562	0.10130579	0.05345687	0.14628979
Gga2	1488.69343	1.09978792	0.13722534	0.08229009	0.07022437	0.17955928
Gga3	861.751836	1.18518955	0.24511781	0.10020962	0.00625511	0.02647983
Ggct	772.541929	-2.1651019	-1.1144349	0.08899003	4.34E-37	5.92E-35
Ggcx	377.92038	-1.6281804	-0.7032606	0.11957465	3.66E-10	8.24E-09
Ggh	902.18338	1.1269106	0.17237307	0.08472887	0.02656295	0.08534522

Ggnbp1	123.672206	1.0386561	0.05471805	0.15542964	0.61718143	0.77476042
Ggnbp2	2812.80057	1.04169087	0.05892722	0.07484075	0.40121852	0.60084279
Ggps1	658.438266	-1.0242212	-0.0345273	0.09891998	0.69362239	0.82485173
Ggt1	3.76528479	-1.0635641	-0.0889069	0.24142628	0.01335885	0.04957327
Ggt7	556.308413	1.25808828	0.33123316	0.11258739	0.0008773	0.0052417
Ghdc	633.527086	-1.141541	-0.1909826	0.11492629	0.05197368	0.14317911
Ghitm	11025.2653	-1.0343083	-0.0486663	0.04502392	0.26836258	0.46299322
Ghr	1133.73701	-1.0313562	-0.0445426	0.08296816	0.55973243	0.73310886
Ghrl	4.79682466	1.00135374	0.00195172	0.21139348	0.96426249	0.98280897
Gid4	955.773171	1.40181151	0.48729238	0.09764668	9.70E-08	1.51E-06
Gid8	1888.42241	1.38084128	0.4655475	0.07771482	3.66E-10	8.24E-09
Gigyf1	1028.65526	1.0575583	0.0807372	0.08194949	0.28601577	0.48253154
Gigyf2	3604.7091	-1.0351981	-0.0499069	0.06109765	0.39463574	0.59565027
Gimap6	35.1412476	16.3285839	4.02932777	0.50127767	1.31E-16	6.10E-15
Gin1	702.447526	1.01138664	0.01633463	0.08993835	0.84240757	0.91543734
Ginm1	972.301042	1.1269934	0.17247907	0.08400641	0.02559279	0.08304044
Gins1	487.544709	1.05818621	0.08159352	0.11127507	0.39283366	0.594043
Gins3	1084.05011	1.12404714	0.16870254	0.07369095	0.01458787	0.05336111
Gins4	1298.88408	1.07935141	0.11016465	0.07725213	0.12573086	0.27466103
Gipc1	801.584698	1.01330127	0.01906318	0.09775543	0.82770139	0.90766699
Git1	2539.08906	1.06522211	0.09115428	0.07451765	0.19127934	0.36991068
Git2	2181.38996	1.10777157	0.14766042	0.05928495	0.00941024	0.03718234
Gja1	8916.97159	-1.1983964	-0.2611052	0.06090503	7.72E-06	8.19E-05
Gjb3	835.377508	-1.0805802	-0.1118061	0.09007987	0.17010866	0.34122955
Gjb4	105.519646	-1.0031574	-0.004548	0.15403425	0.96507918	0.98295812
Gjb5	856.975632	1.26989703	0.34471152	0.10130353	0.00017787	0.00135384
Gk5	48.3151133	1.09604316	0.13230461	0.21436117	0.2356869	0.42542782
Gkap1	367.934557	-1.0270275	-0.0384748	0.11607289	0.69317319	0.82455591
Gla	1057.69657	-1.0804062	-0.1115738	0.07391644	0.10700533	0.24494966
Glb1	1344.11928	1.23505612	0.3045766	0.08428884	0.00010046	0.0008155
Glb1l	114.342035	1.23924384	0.30946009	0.20234684	0.02807358	0.08885051
Glcci1	599.501912	-1.0082771	-0.0118921	0.08886957	0.88218105	0.93688768
Glce	2366.99892	1.12905927	0.17512123	0.06374299	0.00394035	0.0182553
Gldc	50.6387294	-3.1622621	-1.660957	0.37430288	4.13E-07	5.68E-06
Glg1	7499.35571	1.22891956	0.29739049	0.05279366	6.52E-09	1.23E-07
Gli1	416.027401	-1.2923931	-0.3700449	0.13386233	0.00121066	0.00680197
Gli2	723.675812	-1.2984288	-0.3767669	0.0885832	4.99E-06	5.50E-05
Gli3	5605.64245	1.10498386	0.1440253	0.04815715	0.00209384	0.01081536
Glipr1	83.3713369	-1.4506522	-0.5367017	0.32175395	0.00720951	0.02979156
Glipr2	1220.35216	-1.2620786	-0.3358017	0.07523836	2.35E-06	2.81E-05
Glis1	245.697083	1.58803658	0.66724414	0.15296695	1.16E-06	1.47E-05
Glis2	681.295248	-1.250053	-0.3219893	0.11087086	0.00104232	0.00603349
Glis3	1680.69474	1.44668448	0.53275031	0.07116029	1.06E-14	4.08E-13
Glmn	991.643171	1.0666364	0.09306847	0.0745349	0.18262334	0.35869442
Glmp	2268.05962	1.37768989	0.46225118	0.08565928	1.28E-08	2.31E-07
Glo1	8575.0555	-1.027704	-0.0394249	0.05471374	0.45090817	0.64618934
Glrx	1762.65174	-1.2206079	-0.2875998	0.0700586	1.51E-05	0.00015011
Glrx3	6337.43614	-1.0907331	-0.1252982	0.05825695	0.02557429	0.08302247
Glrx5	1276.21879	-1.1372385	-0.1855348	0.08038288	0.01248759	0.04698455
Gls	1786.43057	-1.201202	-0.2644788	0.06917663	5.48E-05	0.00047485

Gls2	61.7891992	-1.2333155	-0.302542	0.28147165	0.04530094	0.12941683
Glt8d1	752.15681	1.26383736	0.33781082	0.09492631	0.00010376	0.00083968
Glt8d2	81.6617439	1.00821743	0.0118068	0.1699928	0.91146102	0.95421834
Gltp	421.825481	1.04803845	0.06769164	0.11426938	0.48662218	0.67514677
Glud1	2958.87298	-1.0525119	-0.0738366	0.07408045	0.28684793	0.48317186
Glyatl3	6.04071844	-1.003131	-0.0045101	0.21160171	0.90738867	0.95207484
Glyctk	64.5134425	-1.1019063	-0.1400015	0.18998577	0.23576207	0.42542782
Glyr1	1502.03591	-1.1619039	-0.2164907	0.06584544	0.00053414	0.00345052
Gm10337	2.62630523	-1.0222733	-0.0317809	0.21762832	0.1974824	0.37819547
Gm1110	60.6397903	1.37926968	0.46390456	0.41159188	0.01740673	0.06151178
Gm1123	2.00181649	-1.0297918	-0.0423527	0.22121584	0.02273948	0.0759762
Gm11437	2.4116827	-1.0234272	-0.0334085	0.21688515	0.30323216	0.50095498
Gm12248	25.4373831	-1.0710348	-0.0990054	0.23234264	0.18641199	0.36364147
Gm12695	1.62198359	-1.0275881	-0.0392621	0.22031278	0.03659385	0.10978154
Gm14137	18.2929373	-1.2534721	-0.3259299	0.67734062	0.01302028	0.04854187
Gm17018	497.567907	1.10127524	0.13917509	0.10258104	0.12305586	0.2707567
Gm17190	668.433018	-1.4326777	-0.5187141	0.13569173	1.80E-05	0.00017531
Gm17657	3.04005288	1.01121632	0.01609166	0.21376274	0.62317761	0.77883907
Gm17728	6.90077737	-1.0290575	-0.0413236	0.2138576	0.43909619	0.6366522
Gm17949	11.4173001	1.00246681	0.00355447	0.20842298	0.94885679	0.97462988
Gm19410	2.00180018	-1.0131896	-0.0189041	0.21436483	0.54389064	0.72161979
Gm2000	3802.53376	1.0025385	0.00365765	0.09028952	0.96718444	0.98416337
Gm28710	77.8359148	-1.1947237	-0.2566771	0.31121146	0.06196894	0.1641485
Gm29797	5.9343455	1.00106115	0.00153011	0.21178114	0.97105665	0.98673136
Gm2a	922.204007	1.06121793	0.08572096	0.08659769	0.27914599	0.47481876
Gm32742	3.66680221	1.00329983	0.0047528	0.21201738	0.90352657	0.94988097
Gm35339	9.70572151	-1.0337362	-0.047868	0.21316573	0.43718685	0.63469534
Gm38394	2678.97555	-1.1290591	-0.175121	0.05595313	0.00115417	0.00656232
Gm3854	30.1604582	1.05106329	0.07184954	0.20608709	0.42329939	0.62162848
Gm39653	149.678559	-1.3395217	-0.4217179	0.24403907	0.01059224	0.04119371
Gm45902	348.724805	-1.100791	-0.1385405	0.10751549	0.13741171	0.29262934
Gm4952	16.7642228	1.08834717	0.12213883	0.23733464	0.17982719	0.35527241
Gm50241	2562.69798	-1.154365	-0.2070995	0.08149008	0.00593735	0.0253694
Gm527	186.51598	1.1532108	0.20565625	0.14347134	0.06997194	0.17916405
Gm5617	26.6671931	1.03262293	0.04631354	0.19575514	0.60895444	0.76921124
Gm5737	63.4730231	-2.716824	-1.4419211	0.27543235	9.37E-09	1.72E-07
Gmcl1	916.105701	-1.0115522	-0.0165708	0.07572355	0.81425131	0.89920026
Gmds	726.684687	1.48011845	0.56571263	0.09102642	6.95E-11	1.76E-09
Gmeb1	958.896453	-1.1239001	-0.1685138	0.10711781	0.07076744	0.18051192
Gmeb2	328.17351	1.27284013	0.34805122	0.14851151	0.00437662	0.01985286
Gmfb	1664.46815	1.09569084	0.13184079	0.08139885	0.07956736	0.19733936
Gmfg	139.269642	1.12618359	0.17144204	0.17844848	0.16031722	0.32651443
Gmip	308.765813	1.13321983	0.18042776	0.12785493	0.08701243	0.21098415
Gmnn	1861.66133	-1.0605044	-0.0847507	0.07380645	0.22053524	0.40789149
Gmppa	615.885538	1.3467683	0.42950167	0.10427676	7.05E-06	7.54E-05
Gmppb	388.160508	1.20347924	0.26721126	0.1050383	0.0041709	0.0191434
Gmpr	96.511714	1.10293853	0.14135239	0.170924	0.23103491	0.42009329
Gmps	4892.86029	-1.1455248	-0.1960087	0.07116374	0.00341506	0.01624467
Gna11	4373.78009	1.12212112	0.16622841	0.05871043	0.00315399	0.01521754
Gna12	3202.5235	-1.1069273	-0.1465604	0.05708573	0.00754969	0.03093781

Gna13	4397.90571	1.00077448	0.0011169	0.05342348	0.98336239	0.99141132
Gna15	2.46383659	1.00305919	0.00440674	0.21418963	0.85155234	0.92033546
Gnai1	672.488668	-1.2602585	-0.3337197	0.11239642	0.00079743	0.00482754
Gnai2	6291.24839	-1.0189663	-0.0271063	0.05321286	0.6046513	0.76585901
Gnai3	2834.49879	-1.0328162	-0.0465836	0.07623635	0.51265135	0.69669678
Gnao1	484.023183	2.10212797	1.0718505	0.13602628	2.25E-16	1.02E-14
Gnaq	1304.35312	1.09803118	0.13491903	0.0676883	0.03528791	0.10658763
Gnas	43529.7984	1.61336237	0.69007051	0.07191672	2.96E-23	2.13E-21
Gnat1	42.4558285	1.01803655	0.02578936	0.18212713	0.79524434	0.88617607
Gnat2	11.7870246	-1.0242936	-0.0346294	0.21108446	0.5418234	0.71962236
Gnb1	17180.5931	1.02505645	0.03570336	0.04189895	0.3955277	0.59622379
Gnb1l	95.8484358	1.12110363	0.16491965	0.21020203	0.17525541	0.34882253
Gnb2	3579.85759	1.00893873	0.01283857	0.08054724	0.86421045	0.92713638
Gnb3	8.37288951	-1.0445858	-0.062931	0.2216282	0.24206323	0.43199081
Gnb4	2660.35241	-1.1091741	-0.1494858	0.06637401	0.01742436	0.06155709
Gnb5	347.531772	-1.0429817	-0.0607139	0.12497542	0.55046238	0.72695083
Gne	524.597734	-1.0050834	-0.0073153	0.0913356	0.92875847	0.9641278
Gng10	16.1076028	1.0146908	0.02104018	0.20423018	0.75869093	0.86622743
Gng11	113.10333	1.04297793	0.06070863	0.17133155	0.57552788	0.74304698
Gng12	5500.3237	-2.0505644	-1.036021	0.06064128	1.29E-66	6.60E-64
Gng8	5.31257769	1.02201644	0.03141841	0.21438044	0.46753708	0.660123
Gngt2	2.39767067	-1.0023587	-0.0033989	0.21321991	0.90955827	0.95292637
Gnl1	2241.06714	1.01715007	0.02453256	0.07014572	0.71196279	0.83733413
Gnl2	4380.79397	-1.0368096	-0.052151	0.05218626	0.3024964	0.50038467
Gnl3	5850.80587	-1.1771417	-0.235288	0.06530819	0.00015045	0.00116522
Gnl3l	6256.43874	-1.1199065	-0.1633782	0.0474995	0.00040732	0.00271596
Gnpat	1424.37325	1.06034066	0.08452784	0.06934558	0.19702889	0.37783535
Gnpda1	409.479006	1.12958362	0.17579107	0.09866216	0.04543888	0.12962083
Gnpda2	427.718087	-1.2811899	-0.3574844	0.118661	0.00061976	0.00390212
Gnptab	9759.64286	-1.0019212	-0.0027691	0.05074086	0.95459173	0.97803088
Gnptg	477.198857	1.38725661	0.47223468	0.1344941	6.92E-05	0.00058735
Gns	875.015166	1.2384645	0.30855252	0.07753328	2.31E-05	0.00021698
Golga1	685.692381	1.07275351	0.10131863	0.09781629	0.2450985	0.43540789
Golga2	1067.02868	1.07506667	0.10442613	0.06651977	0.09783221	0.22933531
Golga3	5381.49844	1.03876969	0.05487582	0.04387687	0.20222574	0.38503444
Golga4	7902.39133	1.0094768	0.01360775	0.0571959	0.80999385	0.89618424
Golga5	1232.01253	-1.0509501	-0.0716941	0.07675638	0.31597579	0.51496817
Golga7b	3.99037969	-1.0242896	-0.0346236	0.21720987	0.2768791	0.47246854
Golgb1	5544.04787	-1.0842805	-0.116738	0.06005584	0.04241501	0.12312175
Golim4	7199.70148	1.13124353	0.17790954	0.05192604	0.0004061	0.00271084
Golm1	873.050946	1.05981294	0.08380965	0.07832427	0.25021881	0.44150178
Golph3	3971.70469	1.03356284	0.04762611	0.05578951	0.37678747	0.57866532
Golph3l	723.730853	1.13911718	0.18791616	0.10054252	0.03543038	0.10691711
Golt1b	3477.0129	1.00759369	0.010914	0.06274271	0.85588138	0.92303759
Gon4l	4525.38401	1.02790221	0.03970302	0.0569427	0.47584401	0.66661874
Gopc	1537.96418	-1.0474259	-0.0668482	0.05904324	0.23885255	0.42870971
Gorab	1330.26368	-1.1077768	-0.1476672	0.07498672	0.03485172	0.10566876
Gorasp1	404.265347	1.00437199	0.00629371	0.10927833	0.94758097	0.97412024
Gorasp2	3420.50753	1.10396653	0.14269643	0.05700974	0.00926789	0.03671047
Gosr2	4501.53312	1.17177804	0.22869932	0.06407324	0.00018349	0.00139327

Got1	2691.32496	-1.2074252	-0.2719338	0.05947318	1.95E-06	2.37E-05
Got2	3837.27877	-1.0399742	-0.0565477	0.05387487	0.27739514	0.47297109
Gp5	2.55475057	-1.0281831	-0.0400973	0.21982565	0.08266492	0.20270628
Gpaa1	649.927709	1.078079	0.1084629	0.10706731	0.24382962	0.43405468
Gpalpp1	2321.05968	1.00499545	0.00718897	0.07572741	0.91987355	0.95871614
Gpam	6419.26776	1.00970571	0.01393486	0.04586764	0.75635586	0.86469599
Gpank1	1395.32106	1.03088753	0.04388695	0.06752457	0.49423	0.68105489
Gpat3	465.710143	-1.2389867	-0.3091607	0.11261798	0.00182042	0.0096463
Gpat4	3271.74638	-1.2016154	-0.2649752	0.05975357	3.94E-06	4.45E-05
Gpatch1	330.502262	1.04205982	0.0594381	0.10550576	0.51891056	0.70185242
Gpatch11	2672.69125	1.14693544	0.19778419	0.05988515	0.00057004	0.00364381
Gpatch2	992.159982	-1.2750955	-0.3506053	0.08582757	1.18E-05	0.00012091
Gpatch2l	412.25859	1.1339322	0.18133439	0.10724266	0.05254243	0.14428053
Gpatch3	231.250963	-1.0948015	-0.1306693	0.12822437	0.21059672	0.39591194
Gpatch4	1662.92906	-1.2364776	-0.3062361	0.08087241	5.08E-05	0.00044413
Gpatch8	5540.48011	-1.0457524	-0.0645414	0.04242125	0.11578446	0.25911932
Gpbp1l1	1812.83846	1.06203173	0.08682687	0.0732731	0.20624417	0.39065324
Gpc1	4795.89715	-1.7190152	-0.7815823	0.0586788	1.42E-41	2.40E-39
Gpc6	482.328049	1.11501635	0.15706486	0.10326379	0.08385033	0.20498525
Gpcpd1	395.128868	-1.0647905	-0.0905696	0.11716304	0.35759258	0.55772996
Gpd1l	356.810574	1.02771684	0.03944283	0.10630103	0.67020075	0.80970212
Gpd2	4954.96172	-1.0121852	-0.0174733	0.05268429	0.72862678	0.84767134
Gphn	1408.89823	1.01831104	0.02617829	0.07321416	0.70419235	0.83189421
Gpkow	920.524699	-1.2527054	-0.3250472	0.08236858	2.41E-05	0.00022531
Gpld1	24.4238361	1.94589786	0.96043599	0.59599355	0.00370621	0.01739705
Gpn1	807.455295	-1.0106002	-0.0152124	0.0817215	0.83977432	0.9142043
Gpn2	435.435868	-1.1254931	-0.1705572	0.11956935	0.08990625	0.21593637
Gpn3	604.514392	1.12156109	0.1655082	0.13565182	0.12929375	0.27999653
Gpnmb	732.087458	1.6704851	0.74026711	0.09829037	4.88E-15	1.97E-13
Gpr107	1352.84007	-1.0262686	-0.0374084	0.07015585	0.57210568	0.74079387
Gpr108	627.35065	1.37289471	0.45722099	0.09147459	1.00E-07	1.55E-06
Gpr137	45.1962422	1.22896365	0.29744225	0.35730601	0.0439861	0.12648627
Gpr137b	191.016057	-1.3292567	-0.4106197	0.18697461	0.0043174	0.01962596
Gpr137c	5.29925943	1.02339751	0.03336663	0.21413027	0.46170524	0.65527892
Gpr146	3.70833867	-1.0053663	-0.0077213	0.21299603	0.8111659	0.89713102
Gpr15	3.74607342	1.00865	0.01242565	0.21297526	0.72455613	0.84468495
Gpr156	39.3758126	-2.4506653	-1.2931735	0.40611798	6.93E-05	0.00058777
Gpr158	1.63132164	-1.0023802	-0.0034298	0.21416043	0.88161748	0.93667744
Gpr161	197.743423	1.4238501	0.50979727	0.15761479	0.00015778	0.00121391
Gpr174	22.6066536	1.0150273	0.02151853	0.20134984	0.77471468	0.87439649
Gpr176	70.7295514	-1.4217922	-0.5077106	0.29819151	0.00746752	0.0307019
Gpr180	927.088221	1.01249689	0.01791748	0.08746339	0.82320409	0.90419748
Gpr19	90.0005571	-1.0303992	-0.0432034	0.17500758	0.67849046	0.81499755
Gpr27	18.9574739	-1.0173446	-0.0248085	0.20833132	0.67332979	0.81118731
Gpr3	9.86522748	1.00405855	0.0058434	0.20625104	0.92371003	0.96109284
Gpr39	330.218835	-1.7050954	-0.7698525	0.12307813	3.45E-11	9.18E-10
Gpr89	952.221243	-1.0124437	-0.0178417	0.09417515	0.83256296	0.91007181
Gprasp1	985.433896	-1.1028981	-0.1412995	0.09603575	0.0998649	0.23276372
Gprasp2	27.7247897	1.06641461	0.09276845	0.21249366	0.32356089	0.52293906
Gprc5a	450.220528	1.07124921	0.09929415	0.12366157	0.3282646	0.52808662

Gps1	3579.46925	1.10407131	0.14283335	0.04744007	0.00200767	0.01046309
Gps2	317.518579	1.7589379	0.81470455	0.17515848	2.72E-07	3.88E-06
Gpsm1	111.00196	-1.4236385	-0.5095828	0.25680256	0.00449215	0.0203122
Gpsm2	3621.42832	1.35138718	0.43444107	0.05214908	1.62E-17	8.09E-16
Gpsm3	363.451422	-1.3160304	-0.3961928	0.12857378	0.0004075	0.00271596
Gpt	2.37811919	-1.0211611	-0.0302105	0.21681815	0.2908496	0.48747935
Gpt2	455.121359	1.25905815	0.33234492	0.1499309	0.00640609	0.02698519
Gpx1	1242.13237	1.01899328	0.02714454	0.09906518	0.75428124	0.86317158
Gpx8	4570.67074	-1.0077571	-0.0111479	0.05019912	0.81828224	0.90192653
Gramd1a	466.256956	1.02295454	0.03274203	0.10794293	0.72661353	0.84617449
Gramd1b	16.6464608	-8.2921188	-3.0517408	0.7093968	7.69E-07	1.01E-05
Gramd1c	102.593279	1.1628653	0.21768399	0.20255962	0.09713087	0.22809284
Gramd2	5.51137518	1.01887974	0.02698378	0.21251039	0.56496433	0.73646006
Gramd3	749.217622	-1.0322985	-0.0458603	0.08530204	0.55719394	0.73133339
Gramd4	1329.56969	-1.2078271	-0.272414	0.09612122	0.00171094	0.00913214
Grap2	2.43520178	-1.0024194	-0.0034863	0.21425544	0.87337007	0.93193424
Grasp	93.2302089	1.04504846	0.06356984	0.16014058	0.56524677	0.73652845
Grb14	883.146469	1.4398543	0.52592283	0.08100677	1.24E-11	3.49E-10
Grb2	2793.93278	-1.1060203	-0.1453778	0.06205498	0.01403678	0.05175911
Grcc10	554.856723	-1.0380781	-0.053915	0.08408064	0.48992992	0.67738827
Greb1l	4.14090379	1.02967272	0.04218585	0.2177603	0.28406258	0.48037513
Grhl1	210.57607	-1.0390262	-0.0552321	0.14175417	0.60729025	0.7678038
Grhl3	3.00080608	-1.0374419	-0.0530305	0.22279273	0.11067513	0.25106223
Grhpr	115.160894	1.20129542	0.26459098	0.21434422	0.05577569	0.15111813
Grik5	617.117416	1.08257704	0.1144697	0.09433754	0.1761358	0.34986813
Grin2d	427.51813	1.28978643	0.36713219	0.11903058	0.00047217	0.00309385
Grin3b	3.97939309	1.00313633	0.00451768	0.21173579	0.90988591	0.95303667
Grina	777.586782	1.1181322	0.16109077	0.10002878	0.06902916	0.1776343
Gripap1	1290.52382	1.07671914	0.10664197	0.07285119	0.11871226	0.26355356
Grk2	3380.86689	1.15544269	0.2084457	0.05943863	0.00025677	0.00183757
Grk4	509.632074	1.20430906	0.26820568	0.11732216	0.00814483	0.03295492
Grk5	277.851133	1.01974696	0.02821121	0.13190643	0.7882467	0.88132248
Grk6	2050.69174	-1.1455521	-0.196043	0.06208106	0.00093494	0.00551654
Grn	3871.82909	1.38336404	0.46818086	0.07327551	2.92E-11	7.86E-10
Grpel1	1141.13973	-1.0456227	-0.0643624	0.08115017	0.39048521	0.5920364
Grsf1	3106.75013	1.01507036	0.02157973	0.07921253	0.7714701	0.87286097
Grwd1	1393.17727	-1.124461	-0.1692337	0.07158161	0.01188469	0.04526997
Gsap	221.591031	1.00791775	0.01137791	0.12105481	0.91053553	0.95356117
Gsdma	27.3080722	-1.0994946	-0.1368405	0.24316677	0.1609753	0.32736841
Gsdmd	142.996844	1.10026343	0.13784898	0.14757388	0.22546931	0.41408772
Gsdme	75.4619108	-1.2492069	-0.3210124	0.34183937	0.0396165	0.11671742
Gse1	440.020035	2.08941575	1.06309959	0.10613791	8.32E-25	6.62E-23
Gsk3a	1268.26113	1.13186243	0.17869862	0.06731767	0.00505057	0.02233554
Gsk3b	1510.90107	1.08942792	0.12357074	0.07124789	0.06560369	0.17140184
Gskip	196.404182	1.07370708	0.10260047	0.13120478	0.33012067	0.53012984
Gsn	165.654127	1.00629584	0.0090545	0.15128238	0.93434998	0.96821091
Gspt1	11729.0639	-1.2052282	-0.2693063	0.06537124	1.51E-05	0.00015058
Gspt2	9.17066657	1.00583449	0.00839293	0.20733826	0.88470266	0.93815523
Gsr	2186.8565	-1.0507031	-0.071355	0.06208643	0.22949282	0.41806073
Gss	3567.01649	-1.2667507	-0.3411326	0.06505694	4.49E-08	7.40E-07

Gsta4	1806.50129	-1.1405458	-0.1897244	0.07217572	0.00511253	0.02252758
Gstcd	1253.95883	1.01308967	0.01876188	0.06883788	0.77404979	0.87400421
Gstm1	2051.00988	1.70077156	0.76618938	0.07446443	7.70E-26	6.53E-24
Gstm2	140.584568	1.31037661	0.38998151	0.18436443	0.00594808	0.02539829
Gstm4	2240.51013	1.07159269	0.09975664	0.08179608	0.18641948	0.36364147
Gstm5	2765.0728	-1.0943098	-0.1300213	0.05240082	0.01028347	0.04017423
Gstm7	808.616686	1.62421675	0.69974417	0.08489904	1.83E-17	9.07E-16
Gsto1	294.921123	-1.2116821	-0.2770113	0.12311286	0.00839275	0.03386178
Gstp1	3080.25111	-1.0195731	-0.0279652	0.09175882	0.73331964	0.85081284
Gstp3	28.8748572	1.03245553	0.04607964	0.19869547	0.59673905	0.75956153
Gstt2	278.150655	1.21860527	0.28523088	0.12398696	0.00702718	0.0291604
Gstz1	102.362374	1.03261636	0.04630436	0.15626705	0.67223489	0.81086907
Gtdc1	271.34174	1.18555098	0.24555771	0.14590348	0.0342348	0.10426719
Gtf2a1	4477.58441	-1.0263496	-0.0375222	0.05540817	0.48319109	0.67255597
Gtf2b	1051.73267	-1.0704273	-0.0981868	0.09229406	0.23732177	0.42679936
Gtf2e1	1511.33602	-1.1471883	-0.1981022	0.06892222	0.00232529	0.01183903
Gtf2e2	858.950975	-1.0112092	-0.0160815	0.07658506	0.82152083	0.90376956
Gtf2f1	2296.11045	-1.1149243	-0.1569458	0.06208885	0.0080274	0.03257238
Gtf2f2	2418.88278	-1.2748815	-0.3503632	0.06855077	8.84E-08	1.38E-06
Gtf2h1	3283.31385	-1.1350892	-0.1828056	0.06915472	0.00506255	0.02237023
Gtf2h2	744.085198	-1.0805882	-0.1118169	0.0767004	0.11760158	0.26199586
Gtf2h3	1960.32129	-1.0503861	-0.0709198	0.0696983	0.2810629	0.47687627
Gtf2h4	1142.50972	-1.1290953	-0.1751672	0.07859592	0.01627192	0.05806761
Gtf2i	2009.8162	1.04210719	0.05950368	0.06108598	0.30964986	0.50795425
Gtf2ird1	847.622821	1.29666313	0.37480372	0.08146664	1.02E-06	1.32E-05
Gtf2ird2	120.530823	1.38626785	0.47120604	0.20875382	0.00301774	0.01465955
Gtf3a	1099.75627	-1.0396087	-0.0560406	0.07448233	0.42130607	0.61976697
Gtf3c1	6100.66434	1.08070224	0.11196908	0.04659296	0.01368219	0.05059723
Gtf3c3	3713.64216	1.03683334	0.05218402	0.06000239	0.3642624	0.56524338
Gtf3c4	1358.87962	1.00611296	0.0087923	0.07500455	0.90107296	0.94874637
Gtf3c5	1391.17746	1.15477506	0.20761186	0.06496164	0.00078463	0.00476581
Gtf3c6	2373.95293	-1.0350644	-0.0497205	0.08148617	0.50894121	0.69327275
Gtpbp1	1361.13293	1.06552288	0.09156157	0.08741673	0.25264602	0.44390425
Gtpbp10	754.47814	1.05497112	0.0772035	0.09356622	0.35933348	0.55964278
Gtpbp2	553.840853	-1.0769084	-0.1068956	0.11320223	0.26699874	0.46126148
Gtpbp3	635.10466	-1.0148614	-0.0212827	0.09791641	0.80557333	0.89269198
Gtpbp4	5827.61752	-1.1807761	-0.2397354	0.06166258	4.94E-05	0.00043334
Gtpbp6	84.2360663	-1.2032615	-0.2669502	0.23995755	0.05917866	0.15842813
Gtpbp8	93.0147652	-1.1141789	-0.1559809	0.19244131	0.19779337	0.37850808
Gtse1	2588.30669	-1.0498013	-0.0701164	0.0789121	0.33819464	0.53776978
Guca2a	2.84076253	-1.0201912	-0.0288396	0.21625244	0.33794891	0.53745346
Gucd1	1089.10519	-1.1006814	-0.1383969	0.07110061	0.03834208	0.11380028
Gucy2f	1.87907701	1.00195233	0.00281387	0.21372929	0.91715676	0.95692723
Gucy2g	2.13487122	-1.024709	-0.0352142	0.21909408	0.06225451	0.16459862
Guf1	1003.11828	1.04148243	0.0586385	0.07121514	0.38276816	0.58431392
Gulp1	907.051736	1.0008017	0.00115615	0.09227239	0.99005913	0.9954343
Gusb	2060.21786	1.02040115	0.02913643	0.06519641	0.63952361	0.79058554
Gxylt1	153.440817	-1.0123887	-0.0177632	0.14419544	0.86752863	0.92836933
Gyg	3359.13523	1.05216872	0.07336607	0.0515946	0.14288897	0.30111192
Gypc	32.7873941	-1.0205247	-0.0293112	0.19171907	0.74427845	0.85754694

Gys1	1678.11407	1.16056993	0.21483345	0.06969277	0.00109681	0.00629487
Gzf1	816.731822	1.01280438	0.01835555	0.08556974	0.81092164	0.89697834
Gzmm	30.5270856	1.10717194	0.14687929	0.23588051	0.17725471	0.3513655
H13	1815.84936	-1.0927825	-0.1280062	0.07577672	0.07006117	0.17933641
H1f10	278.015508	-1.2126906	-0.2782116	0.16698276	0.02787006	0.08832086
H1f2	13623.8223	-1.0501851	-0.0706437	0.0837371	0.37517456	0.57708767
H1f3	634.090939	-1.1066405	-0.1461866	0.13590138	0.17604965	0.34985975
H2-DMa	50.5988741	-1.1748957	-0.2325326	0.26470113	0.08301891	0.2033554
H2-Ke6	234.014926	1.2444575	0.31551697	0.15974873	0.01236155	0.04664254
H2-T23	439.924518	1.11959601	0.16297825	0.11784876	0.1019799	0.23665988
H2ac12	1048.8385	-1.7911181	-0.8408604	0.13339698	2.28E-11	6.25E-10
H2ac19	12732.5949	-1.0552779	-0.0776229	0.12352403	0.44401025	0.64065908
H2ac21	864.726544	1.14208591	0.19167117	0.12293167	0.066567	0.17317695
H2aj	28.7819989	1.03976994	0.05626435	0.19855965	0.5352165	0.71425392
H2az2	1232.30516	-1.1062002	-0.1456125	0.09419967	0.08522884	0.20760248
H2bc1	3.10469298	-1.0127037	-0.0182121	0.21344258	0.61222395	0.77189377
H2bc14	13.7913664	-1.0424295	-0.0599499	0.21144583	0.4181773	0.61714876
H2bc18	5384.26998	-1.0635537	-0.0888929	0.063381	0.14135101	0.29884576
H2bc3	1433.87149	-1.0660121	-0.0922238	0.0951577	0.2786009	0.47441657
H3f3b	18161.6564	-1.0271463	-0.0386417	0.06296386	0.52298429	0.70506457
H4f16	661.504949	1.0053515	0.00769999	0.17718833	0.94205475	0.97189769
H6pd	289.989359	1.02943997	0.0418597	0.11106809	0.6603748	0.80343823
HMGCR	2054.73645	-1.2317367	-0.3006939	0.08361982	0.00010892	0.0008781
HSPD1	39960.6921	-1.1293752	-0.1755249	0.06429982	0.00386304	0.01798822
Habp4	1030.1767	1.03415568	0.04845338	0.07372359	0.48449171	0.67336397
Hacd1	176.420176	-1.1458574	-0.1964276	0.16206485	0.10110205	0.23509117
Hacd2	1011.99714	-1.058192	-0.0816014	0.07693422	0.25471103	0.44629303
Hacd3	504.434362	-1.0413176	-0.0584101	0.09815889	0.5163058	0.6997331
Hace1	1906.07835	1.07795513	0.10829713	0.05711596	0.04889497	0.13696578
Hadh	1765.15215	1.14641142	0.19712488	0.07030976	0.00295364	0.01440279
Hadha	5250.62035	-1.0020485	-0.0029524	0.04590943	0.94423896	0.97287067
Hadhb	1343.40335	-1.1607446	-0.2150506	0.09585183	0.01242724	0.04679393
Hagh	629.184603	-1.169929	-0.226421	0.13244972	0.03728873	0.1113966
Hao1	4.08557811	-1.0153458	-0.0219712	0.21364068	0.56225379	0.73502103
Hapln4	81.8978156	1.19967681	0.2626458	0.27474515	0.06504576	0.17046015
Harbi1	230.083208	1.08818707	0.12192659	0.1312836	0.24753566	0.43833727
Hars	2519.15584	-1.0836012	-0.1158338	0.06110201	0.04739302	0.13360578
Hars2	587.454457	-1.2354198	-0.3050014	0.09801693	0.00058431	0.00371462
Has3	18.3961871	-1.1082849	-0.1483288	0.26450406	0.09936449	0.23189271
Haspin	440.928237	1.0390852	0.05531395	0.09398219	0.51292288	0.69679862
Hat1	3196.01355	-1.0190861	-0.0272759	0.05378334	0.59985281	0.76253989
Haus1	366.939403	1.39657408	0.4818921	0.1539652	0.00024069	0.00174393
Haus2	1712.13097	-1.0045457	-0.0065432	0.05849393	0.90689023	0.95184464
Haus3	1858.0417	1.12333865	0.16779292	0.08436057	0.03030474	0.09456851
Haus4	2150.9	1.2567469	0.32969414	0.06021045	1.36E-08	2.43E-07
Haus5	179.022199	-1.0538431	-0.0756601	0.14515236	0.48624956	0.67499887
Haus7	553.453383	1.05819317	0.08160301	0.08641257	0.3019816	0.49979125
Haus8	716.974156	1.13594691	0.18389541	0.09197773	0.02705267	0.08649645
Hbegf	525.478076	-1.4599006	-0.5458701	0.11466988	2.51E-07	3.60E-06
Hbp1	912.95617	1.07726724	0.10737619	0.07472753	0.12445181	0.27284404

Hbs1l	3476.48827	1.02142629	0.0305851	0.06275527	0.61105244	0.77118285
Hccs	1455.37089	-1.0929127	-0.1281782	0.07727437	0.07479926	0.18832124
Hcfc1	9530.80539	-1.0812148	-0.1126531	0.04917736	0.01991583	0.06837892
Hcfc1r1	706.349894	1.23452299	0.3039537	0.10169987	0.00090297	0.00536507
Hcfc2	1291.56457	1.23014551	0.29882898	0.07182649	1.11E-05	0.00011419
Hcn3	26.3223262	1.2754249	0.35097796	0.50936006	0.02574779	0.0833955
Hcrtr1	4.16661048	-1.0115861	-0.0166191	0.2131926	0.64567967	0.79392941
Hdac2	8285.55692	-1.1172294	-0.1599255	0.04479132	0.000255	0.00182689
Hdac3	592.991778	-1.1140044	-0.1557549	0.10071474	0.07991749	0.19778652
Hdac4	1839.16486	-1.0303394	-0.0431197	0.0611689	0.46120881	0.65499832
Hdac5	1179.22907	1.11127562	0.15221667	0.10123001	0.08868239	0.2139803
Hdac6	2489.05005	1.00655009	0.00941897	0.05973395	0.87017344	0.92968488
Hdac7	738.855643	1.15781761	0.21140801	0.09482728	0.01323141	0.04914304
Hdac8	173.675312	-1.2478673	-0.3194645	0.17222459	0.01504904	0.05465782
Hdac9	28.4774176	-2.1527678	-1.1061927	0.50221785	0.0011464	0.00652683
Hddc2	871.093515	-1.0011676	-0.0016835	0.08453372	0.98193378	0.99108517
Hddc3	322.635367	1.02037675	0.02910193	0.13708708	0.78715031	0.88066764
Hdgf	14031.8535	1.07109185	0.0990822	0.04383297	0.02092263	0.07113028
Hdgfl2	2605.51487	-1.0129936	-0.0186251	0.06623224	0.76696558	0.87111526
Hdgfl3	809.824322	1.04937429	0.06952935	0.10591141	0.45204151	0.6470374
Hdhd2	487.319317	-1.1791762	-0.2377793	0.11409945	0.01593649	0.05718387
Hdhd3	37.4294561	1.21154083	0.27684303	0.35738026	0.04733298	0.13352465
Hdhd5	392.857741	-1.1952681	-0.2573342	0.12795212	0.01617051	0.05785942
Hdlbp	38345.5261	1.1900385	0.25100825	0.03898684	5.69E-11	1.47E-09
Heatr1	8559.0797	-1.096418	-0.1327979	0.0536833	0.01038776	0.04048286
Heatr3	2416.70775	-1.0571802	-0.0802213	0.0650658	0.1942998	0.37411399
Heatr4	138.012184	-1.0507756	-0.0714547	0.14243041	0.50785559	0.69219621
Heatr5a	2494.7872	1.04899812	0.0690121	0.06855447	0.28677169	0.48317186
Heatr5b	1451.32867	1.22829892	0.2966617	0.08476436	0.00016008	0.00122943
Heatr6	894.283024	1.07595748	0.10562107	0.0780921	0.14580881	0.30539746
Hebp1	600.603472	-1.1483644	-0.1995805	0.0897564	0.01450339	0.05311781
Heca	454.441218	1.09837737	0.13537381	0.10479834	0.13981253	0.29625845
Hectd1	13712.8329	1.08844558	0.12226927	0.04622829	0.00663909	0.02776584
Hectd2	533.921459	1.28711385	0.36413967	0.11024165	0.00022862	0.0016731
Hectd3	2673.3107	1.0152374	0.02181712	0.07795237	0.76467125	0.86962346
Hectd4	3595.43647	1.08502086	0.11772278	0.05164418	0.01862297	0.06473111
Hecw2	10.6219757	-1.075613	-0.1051591	0.24391467	0.09240124	0.22017484
Heg1	3418.93908	-1.3568539	-0.4402654	0.05109221	1.33E-18	7.16E-17
Helb	1149.96713	-1.0132195	-0.0189468	0.06752366	0.76725518	0.87116991
Hells	6292.8061	-1.0397787	-0.0562765	0.05886017	0.31904276	0.51753042
Helq	442.209703	-1.0680685	-0.0950042	0.10198558	0.28895518	0.48550837
Helz	3497.6308	1.03461862	0.04909906	0.05681415	0.37072638	0.57213508
Helz2	2570.45447	1.52463709	0.60846587	0.06910315	1.54E-19	8.97E-18
Hemk1	149.578854	-1.1467216	-0.1975152	0.17632488	0.10994605	0.24993947
Henmt1	22.1120663	-1.0650028	-0.0908572	0.21214831	0.33122853	0.53110997
Herc1	5257.1163	1.13592061	0.18386201	0.05905165	0.00115687	0.00656894
Herc2	5885.17578	1.0874294	0.12092174	0.04682774	0.00851558	0.03427098
Herc3	360.066054	-1.1144193	-0.1562922	0.13094169	0.13758172	0.29285841
Herc4	2564.80568	-1.0975504	-0.1342873	0.0557766	0.01241754	0.04677116
Herc6	21.6682461	-9.9504732	-3.3147651	0.69805076	1.06E-07	1.64E-06

Herpud1	975.031097	1.1632455	0.2181556	0.08131007	0.00375276	0.01755084
Herpud2	1064.98122	-1.0575271	-0.0806946	0.073494	0.24165705	0.43150638
Hes1	426.246847	1.03636671	0.05153458	0.10086097	0.56372398	0.73559175
Hes6	318.677394	1.10977839	0.15027162	0.12550849	0.14706113	0.30736712
Hes7	5.08535549	1.00784335	0.01127142	0.21143796	0.79201726	0.88402889
Hexa	423.231479	1.4436893	0.52976029	0.12145705	1.80E-06	2.21E-05
Hexb	1345.73535	1.33084252	0.41233987	0.07732997	2.04E-08	3.57E-07
Hexdc	412.440368	1.27945366	0.3555279	0.11927505	0.00069743	0.00430146
Hexim1	1229.99733	-1.174782	-0.2323931	0.09336787	0.00590959	0.02527607
Hexim2	137.742015	-1.085881	-0.118866	0.15864488	0.29756565	0.4955559
Hfe	825.299609	1.07220495	0.1005807	0.08175974	0.18146896	0.35741349
Hgf	598.218765	-1.0830651	-0.1151199	0.08793481	0.15018161	0.31170226
Hgh1	243.712453	-1.0040368	-0.0058121	0.11728578	0.95163763	0.97625354
Hgs	5081.92796	1.08080718	0.11210916	0.06393812	0.06511182	0.17049042
Hgsnat	581.225934	1.13652823	0.18463352	0.12269239	0.07220913	0.18334964
Hhipl1	68.2134177	-1.0311346	-0.0442327	0.17496036	0.67200781	0.81086907
Hhipl2	22.783822	-1.0197363	-0.0281962	0.20452273	0.68406581	0.81850035
Hibch	1072.02183	1.11372961	0.15539902	0.07850628	0.03298999	0.10122198
Hic1	341.690894	-1.0483916	-0.0681777	0.12926805	0.51012998	0.69415393
Hic2	715.432385	1.0029657	0.00427226	0.09281015	0.95967964	0.98104669
Hid1	49.4417002	-1.0119119	-0.0170837	0.17807394	0.8632897	0.92665838
Hif1a	4793.43723	1.00094038	0.00135605	0.05957632	0.9814172	0.99108517
Hif1an	513.378259	1.0200923	0.02869969	0.09623835	0.73928214	0.85471157
Higd1a	1355.63019	-1.0389917	-0.0551842	0.08070297	0.45938277	0.65349176
Higd2a	206.034591	1.32795475	0.40920599	0.14935462	0.00110536	0.00632976
Hikeshi	1860.14187	-1.2828387	-0.3593398	0.05832445	1.79E-10	4.31E-09
Hilpda	45.3179612	1.16854816	0.22471719	0.29038762	0.07988684	0.19775664
Hinfp	948.483558	-1.1023123	-0.1405331	0.10074525	0.11548602	0.25858694
Hint1	1989.33592	-1.0531774	-0.0747485	0.06842855	0.24824413	0.43923403
Hint2	164.845544	-1.0706999	-0.0985541	0.16057166	0.38138066	0.583272
Hip1	4736.5512	-1.0124288	-0.0178204	0.05263286	0.72591936	0.84559481
Hip1r	1237.77902	1.02502927	0.03566511	0.07570654	0.61537831	0.77387521
Hipk1	6284.02819	-1.0881531	-0.1218816	0.04408649	0.00464932	0.02089749
Hipk2	3664.44927	1.05166067	0.07266928	0.05457342	0.16879067	0.33932964
Hipk3	5143.05074	-1.1099929	-0.1505504	0.05644461	0.00555529	0.02409046
Hira	2511.02372	1.0047645	0.00685739	0.05398656	0.89618347	0.9455619
Hirip3	2971.57401	-1.0958419	-0.1320397	0.06260972	0.02685324	0.08606204
Hivep1	2868.92382	1.18483194	0.24468244	0.05978476	2.02E-05	0.00019269
Hivep2	2978.25345	-1.0389661	-0.0551485	0.05328067	0.28484254	0.48150342
Hivep3	2136.32382	-1.3546408	-0.4379103	0.07303688	3.94E-10	8.82E-09
Hjurp	1564.68011	-1.0681841	-0.0951603	0.06361437	0.11634956	0.26015671
Hk1	8050.93711	-1.2279185	-0.2962148	0.04376792	4.82E-12	1.43E-10
Hk2	2305.19836	-91.710782	-6.5190195	0.1231366	0	0
Hk3	2.83551186	-1.0247328	-0.0352478	0.21749868	0.25500244	0.44662035
Hkdc1	1.81661267	1.00049697	0.0007168	0.21322432	0.98255451	0.99114898
Hlcs	472.127011	1.12941391	0.17557431	0.1043327	0.05565154	0.15084558
Hltf	4602.8418	1.11318082	0.15468795	0.06055869	0.00758514	0.03106964
Hlx	198.129604	-1.184447	-0.2442137	0.16960366	0.05017569	0.13939504
Hmbox1	932.94439	-1.0308808	-0.0438775	0.08784933	0.58357059	0.74837714
Hmbs	1169.2983	1.1383133	0.18689768	0.0845956	0.01608319	0.05759733

Hmces	533.465342	1.01166247	0.01672803	0.0931667	0.8426883	0.91543734
Hmg20a	1429.55888	1.00027491	0.00039656	0.05977099	0.99618805	0.99837028
Hmg20b	1296.56908	1.09667884	0.1331411	0.07827251	0.06684804	0.17366121
Hmga1	3090.59412	-1.3959594	-0.481257	0.09125556	2.14E-08	3.72E-07
Hmga2	90.2284464	-2.7842878	-1.4773083	0.27887009	6.77E-09	1.27E-07
Hmgb2	4739.92136	-1.1922481	-0.2536845	0.06587077	5.22E-05	0.0004559
Hmgcl	277.562478	1.04484931	0.06329489	0.1285258	0.54157702	0.71951894
Hmgcs1	1987.15981	1.11095909	0.1518057	0.0962605	0.07716312	0.19292603
Hmgcs2	153.776451	-1.6801105	-0.7485561	0.20946466	2.55E-05	0.00023798
Hmgn1	17242.3619	-1.1185502	-0.16163	0.05979712	0.00469419	0.02104743
Hmgn3	488.730957	-1.1566023	-0.2098929	0.10988847	0.0277654	0.08812557
Hmgxb3	1374.97513	-1.0210308	-0.0300264	0.06281205	0.61644459	0.77440965
Hmmr	2601.44313	1.04687462	0.06608867	0.06101863	0.25938799	0.45173799
Hmox1	2947.50507	-1.4606294	-0.5465902	0.07841686	4.16E-13	1.39E-11
Hmox2	1375.59303	1.06661032	0.09303319	0.08518482	0.23384074	0.42333238
Hnf4a	6.42396453	1.0096118	0.01380067	0.21132953	0.75300324	0.86298066
Hnrnpa0	997.602381	-1.1942669	-0.2561253	0.09336138	0.00250343	0.01262061
Hnrnpa2b1	17891.9553	-1.6799248	-0.7483967	0.04970772	2.80E-52	8.54E-50
Hnrnpa3	20757.2499	-1.5314831	-0.6149294	0.06302875	2.04E-23	1.50E-21
Hnrnpab	19828.9033	-1.1419307	-0.1914751	0.06404814	0.00168911	0.00903476
Hnrnpc	9076.55473	-1.1346722	-0.1822756	0.07594812	0.01007337	0.03949799
Hnrnpd	12892.6095	-1.1532946	-0.205761	0.06862426	0.00152757	0.00828108
Hnrnpdl	5884.39225	-1.0719363	-0.1002191	0.07339932	0.14443363	0.30365095
Hnrnpf	19694.0088	-1.1479595	-0.1990718	0.05282293	0.0001003	0.00081473
Hnrnph1	8245.56794	-1.1803829	-0.2392549	0.07650905	0.00077054	0.00469356
Hnrnph2	3947.0645	-1.0487494	-0.06867	0.06090889	0.23910148	0.42897266
Hnrnph3	1924.3303	-1.0868844	-0.1201985	0.08879696	0.13627126	0.29079652
Hnrnpk	27002.2797	-1.1401987	-0.1892852	0.04179416	3.43E-06	3.93E-05
Hnrnpl	10396.9365	-1.1377722	-0.1862117	0.04819342	6.89E-05	0.00058519
Hnrnpll	4013.88005	-1.0961524	-0.1324484	0.0599152	0.02081973	0.07085567
Hnrnpr	7297.68862	1.02607425	0.03713513	0.05965602	0.51436011	0.69805626
Hnrnpu	28529.6348	-1.2190109	-0.2857111	0.0573914	2.31E-07	3.34E-06
Hnrnpul1	7307.31005	1.00347065	0.00499842	0.05893169	0.93037663	0.96526482
Hoga1	488.420477	1.57864818	0.65868969	0.10037554	5.51E-12	1.62E-10
Homer1	2382.05832	-1.1151686	-0.1572618	0.07715413	0.02839856	0.08956603
Homer3	147.061639	1.12247687	0.16668571	0.17132051	0.16600646	0.33499428
Homez	69.9132359	-1.0626744	-0.0876996	0.18953366	0.4121253	0.61119913
Hook1	43.6167254	-2.4634009	-1.3006514	0.47311699	0.00025902	0.00185158
Hook2	560.32261	1.08079919	0.1120985	0.09204788	0.17763419	0.35180797
Hook3	1953.33716	-1.0473697	-0.0667708	0.07496802	0.34037973	0.53990395
Hoxa1	29.553768	-1.2961167	-0.3741956	0.55325424	0.02290482	0.07640904
Hoxa10	50.3233536	-1.0122608	-0.017581	0.17910061	0.85939368	0.92471053
Hoxa2	26.97348	-1.5718355	-0.6524503	0.51733901	0.00840372	0.0338847
Hoxa3	58.110387	-38.178477	-5.2546876	0.54291629	1.52E-22	1.05E-20
Hoxa4	62.88407	1.05097887	0.07173366	0.17311903	0.51320967	0.69701185
Hoxa5	37.999223	-2.7214514	-1.4443763	0.38655701	9.24E-06	9.68E-05
Hoxa6	30.984963	-1.9243569	-0.9443764	0.43156143	0.00126807	0.00706259
Hoxa7	128.703599	-1.0684966	-0.0955823	0.15579031	0.3917656	0.59313526
Hoxa9	67.6366886	-1.0838431	-0.1161559	0.21103448	0.27601991	0.47163066
Hoxb2	97.1863889	1.08907179	0.12309906	0.17488338	0.28938697	0.48574196

Hoxb3	48.9310799	1.05664446	0.07949002	0.18853304	0.45364257	0.64842237
Hoxb4	167.237993	1.11830708	0.1613164	0.16803194	0.17548439	0.34906133
Hoxb5	77.2407537	-1.0002445	-0.0003527	0.16625235	0.99589816	0.99827695
Hoxb6	93.2058685	1.06509773	0.09098582	0.17833203	0.41594372	0.61489808
Hoxb7	1334.71035	1.00454934	0.00654842	0.0694586	0.92092123	0.95921243
Hoxb8	25.8029902	-1.0183123	-0.0261801	0.20015896	0.73527076	0.85199624
Hoxc10	1530.69563	1.01464265	0.02097172	0.07741886	0.77225923	0.87322376
Hoxc4	472.647356	1.05975627	0.0837325	0.09533906	0.32743316	0.52733453
Hoxc5	83.5041632	1.10564981	0.14489452	0.21106354	0.21398155	0.39986924
Hoxc6	162.829594	1.00002681	3.87E-05	0.15628703	0.99886402	0.99959215
Hoxc8	505.619517	1.07306608	0.10173892	0.0985405	0.24494167	0.43535815
Hoxc9	357.31286	-1.1652745	-0.2206699	0.1196705	0.02999347	0.09373418
Hoxd3	43.3408526	1.0459152	0.06476588	0.20612393	0.45180553	0.64695158
Hoxd8	1569.19585	1.10013028	0.13767439	0.07836674	0.0587402	0.1573859
Hoxd9	622.372176	1.09965506	0.13705105	0.09360995	0.10360468	0.23964896
Hp1bp3	7414.47931	1.01335785	0.01914373	0.06455127	0.7569747	0.86517183
Hpcal1	467.384776	-1.1499095	-0.2015203	0.133334	0.06313714	0.16648553
Hpf1	1652.34556	-1.0086491	-0.0124244	0.06620333	0.84303856	0.9155849
Hprt	2965.97293	-1.0752221	-0.1046346	0.06502267	0.09017492	0.21644007
Hps1	298.693536	1.33947233	0.42166478	0.14410369	0.00058946	0.00373808
Hps3	817.903221	1.17874486	0.23725148	0.09806747	0.00697511	0.02898103
Hps4	722.412553	-1.008834	-0.0126888	0.10386229	0.8954819	0.94539263
Hps5	1465.40645	1.11036557	0.15103474	0.07413493	0.0295608	0.09256266
Hps6	346.383253	1.18613298	0.24626576	0.13234126	0.02441926	0.08031085
Hpse	394.040591	1.29850206	0.37684831	0.1170356	0.00028846	0.00202363
Hr	51.7470866	-2.297033	-1.1997716	0.36119037	4.21E-05	0.00037508
Hras	960.021878	-1.1030957	-0.1415579	0.07069752	0.03336692	0.10220027
Hrob	524.939883	-1.0470969	-0.066395	0.1082591	0.47713916	0.66741129
Hs1bp3	591.504627	1.20879056	0.2735643	0.09489284	0.00147988	0.00805663
Hs2st1	1286.29179	1.01296512	0.01858449	0.0811892	0.80558308	0.89269198
Hs6st1	3052.53177	1.02960012	0.04208413	0.06556755	0.50010969	0.68576981
Hsbp1	526.790788	1.10486673	0.14387236	0.12710774	0.16847149	0.33890072
Hscb	184.807098	1.13116082	0.17780405	0.15518583	0.1281677	0.27838166
Hsd17b1	21.1369059	1.01966793	0.02809939	0.20153354	0.71470237	0.83901552
Hsd17b10	4366.69473	-1.0055094	-0.0079265	0.05326443	0.87740026	0.93416734
Hsd17b11	442.764998	-1.055926	-0.0785088	0.09846439	0.36845188	0.57003293
Hsd17b12	2375.03139	-1.11452	-0.1564225	0.05928081	0.0058901	0.02520957
Hsd17b13	15.1873467	-1.2875928	-0.3646764	1.00398257	0.00988039	0.03887216
Hsd17b4	1142.6833	-1.0091002	-0.0130694	0.07005371	0.84298612	0.9155849
Hsd17b7	894.579663	-1.0487439	-0.0686625	0.09346074	0.41358445	0.61234591
Hsd3b7	169.398851	1.0065631	0.00943762	0.15303062	0.93067862	0.96546527
Hsdl1	234.397782	1.20189359	0.26530917	0.14909275	0.02499168	0.08154441
Hsdl2	2572.08261	1.01464786	0.02097912	0.05171364	0.67664462	0.81342132
Hsf1	2017.89615	-1.1664569	-0.222133	0.08000491	0.00274988	0.01364817
Hsf2	1360.49062	1.05184679	0.07292458	0.06808214	0.25880989	0.45100731
Hsf4	13.605794	1.01098895	0.01576722	0.20293989	0.82260184	0.90419748
Hsf5	16.2064706	-1.014443	-0.0206878	0.20232953	0.77285011	0.87363026
Hsh2d	1.89591583	1.02846612	0.04049427	0.21988765	0.10948036	0.24901339
Hsp90aa1	31430.132	-1.1217979	-0.1658128	0.0549697	0.00167377	0.00897113
Hsp90ab1	82876.9519	-1.1926469	-0.254167	0.03927574	4.55E-11	1.20E-09

Hsp90b1	73057.7262	1.03038638	0.04318543	0.05568701	0.41546943	0.61449756
Hspa12a	2.50733301	1.01855647	0.02652597	0.21610774	0.3422802	0.54184495
Hspa13	4648.08688	-1.0956987	-0.1318511	0.06061461	0.02370401	0.07850266
Hspa14	1716.53026	-1.1347774	-0.1824093	0.08221511	0.01603449	0.05747111
Hspa2	1041.43317	-1.284847	-0.3615966	0.08030175	1.71E-06	2.11E-05
Hspa4	7650.47056	-1.1262637	-0.1715447	0.07706616	0.01675123	0.05945125
Hspa4l	3.67588658	-1.0289544	-0.0411791	0.21893599	0.20464366	0.38842574
Hspa5	70129.0155	1.08281364	0.11478497	0.05098726	0.02328096	0.07739065
Hspb1	898.634965	-1.1835443	-0.2431137	0.12999325	0.0243745	0.08023548
Hspb11	693.343975	1.01928668	0.02755988	0.10128727	0.75870974	0.86622743
Hspb6	296.787292	1.1757454	0.23357569	0.12783772	0.02876334	0.09057484
Hspb8	4439.89165	-1.1012138	-0.1390946	0.05717055	0.01132445	0.04348509
Hspbap1	246.875304	-1.0311294	-0.0442254	0.11389405	0.64463696	0.79310405
Hspbp1	573.034701	1.05121962	0.0720641	0.11182721	0.4509201	0.64618934
Hspg2	12427.6616	1.16819457	0.22428058	0.07235782	0.00096913	0.00568723
Hsph1	7887.63037	-1.1939657	-0.2557614	0.05855003	5.53E-06	6.02E-05
Htatip2	828.602089	1.11823968	0.16122944	0.08756062	0.04371974	0.12604262
Htatsf1	5591.14235	1.01652757	0.02364935	0.05564696	0.66261021	0.80471736
Htr1b	368.864814	-1.6009302	-0.6789104	0.12966142	1.52E-08	2.70E-07
Htr7	54.7002089	-1.0178647	-0.0255459	0.17994782	0.79816874	0.88816379
Htra1	3933.07781	-1.0063533	-0.0091369	0.063055	0.8788129	0.93511531
Htra2	682.548048	-1.8265866	-0.8691501	0.12621995	4.13E-13	1.38E-11
Htt	4282.14744	1.16328163	0.21820042	0.05902052	0.00011522	0.00092421
Hunk	895.917116	1.53535561	0.61857284	0.07741458	1.60E-16	7.36E-15
Hus1	1285.96133	-1.1607998	-0.2151192	0.06839418	0.00088525	0.00527444
Huwe1	26005.9527	-1.1188826	-0.1620587	0.0463702	0.00040441	0.00270096
Hvcn1	17.8920248	1.05734282	0.08044322	0.21767978	0.31500494	0.5141709
Hyal1	46.5635072	1.09328899	0.1286748	0.22777862	0.21039915	0.39571474
Hyal2	1340.00555	-1.0059687	-0.0085855	0.07566905	0.90275761	0.94945197
Hyal3	45.2622743	1.02200257	0.03139882	0.18236147	0.75311512	0.86298066
Hydin	6.7681734	1.04599729	0.06487912	0.226915	0.09452252	0.224021
Hyi	559.622893	1.39844104	0.48381943	0.09022699	1.33E-08	2.38E-07
Hykk	264.364526	1.09056025	0.12506948	0.14018367	0.2569989	0.44925708
Hyls1	815.058365	-1.1920808	-0.253482	0.09134008	0.0023014	0.0117407
Hyou1	18638.7325	1.10781902	0.14772221	0.05368311	0.00456618	0.02060329
Hypk	1967.9614	-1.0690088	-0.0962737	0.07806506	0.184153	0.36075211
I830077J02Rik	2.43647217	-1.0420991	-0.0594925	0.22712717	0.00654509	0.02745338
ITPA	582.229603	-1.1315193	-0.1782612	0.11055433	0.06183871	0.16385939
Iah1	763.700869	1.2292391	0.29776557	0.08105054	8.28E-05	0.00068909
Iars	14513.1197	1.24118829	0.31172199	0.04878132	5.53E-11	1.43E-09
Iars2	8734.91315	1.12126515	0.16512748	0.05404459	0.00155381	0.00839491
Iba57	74.3861419	1.00348849	0.00502407	0.17306749	0.96314235	0.98254201
Ibtk	3535.7988	1.29761724	0.37586489	0.05391214	8.01E-13	2.61E-11
Ica1l	8.96102115	1.03786099	0.05361323	0.21733523	0.33402316	0.53398686
Icam1	2971.0275	-1.494724	-0.5798791	0.07779027	1.11E-14	4.24E-13
Icam4	74.6021014	1.07706917	0.1071109	0.18968804	0.33795376	0.53745346
Icam5	118.843194	1.56856312	0.64944359	0.23115886	0.00040106	0.00268419
Ice2	1282.19849	-1.0054168	-0.0077937	0.07068868	0.90629673	0.95137762
Icmt	1062.32659	-1.0882938	-0.1220681	0.07811376	0.09234462	0.22008086
Id1	368.742416	1.43558843	0.5216422	0.14154906	2.94E-05	0.00027109

Id2	1261.13814	1.2067419	0.27111714	0.12760604	0.01176012	0.04490229
Id3	1968.73743	1.42123768	0.50714784	0.10783159	3.65E-07	5.09E-06
Ide	3111.50277	1.11729567	0.16001102	0.06731984	0.01217712	0.04607115
Idh1	1233.17438	1.05299136	0.0744936	0.07953148	0.31283915	0.51180965
Idh2	712.783466	1.29477923	0.37270613	0.10970354	0.00015622	0.00120336
Idh3b	1494.16397	1.07976519	0.11071761	0.06694094	0.08084123	0.19949453
Idh3g	1430.23484	-1.1666188	-0.2223332	0.0646401	0.0003012	0.00209124
Idi1	2720.06542	-1.0554537	-0.0778633	0.07725863	0.27857726	0.47441657
Idnk	256.462379	1.02636272	0.03754068	0.14024201	0.72617683	0.84574282
Ids	13.3579586	-1.0103456	-0.0148489	0.20514559	0.81587973	0.90028592
Idua	493.329713	1.35637821	0.43975952	0.10532475	5.36E-06	5.86E-05
Ier2	80.3899004	-1.2354964	-0.3050908	0.24439468	0.04062529	0.11908696
Ier3	378.902148	-2.3243087	-1.2168017	0.19483544	2.36E-11	6.42E-10
Ier5	102.07281	-2.6520566	-1.4071116	0.3061227	2.05E-07	3.00E-06
Ier5l	66.6705004	-1.1193972	-0.162722	0.21957737	0.17266462	0.3451137
Iffo1	216.721866	-1.0637342	-0.0891377	0.15681707	0.42418138	0.6226382
Ifi209	6.14570462	-1.042561	-0.0601318	0.22411414	0.14111897	0.29850297
Ifi35	30.8131415	1.1647084	0.21996881	0.29725201	0.07725253	0.19303545
Ifi44	29.0567901	1.02063128	0.02946176	0.19746562	0.72374957	0.84422072
Ifih1	438.634581	1.02043655	0.02918648	0.09592445	0.73447539	0.85147553
Ifitm3	2891.12165	-1.222519	-0.2898569	0.08002185	0.00010479	0.00084689
Ifnar1	2184.40792	1.27226184	0.34739561	0.05633817	2.00E-10	4.74E-09
Ifnar2	1597.60392	1.11155158	0.15257489	0.07865464	0.03654711	0.10966701
Ifngr1	708.902404	-1.2393247	-0.3095542	0.0897614	0.0001798	0.00136775
Ifngr2	638.041548	-1.0233444	-0.0332918	0.0955943	0.69690876	0.82691749
Ifrd1	4104.01217	-1.7610149	-0.8164071	0.0499256	3.54E-61	1.51E-58
Ifrd2	4705.83836	-1.1083224	-0.1483776	0.05170185	0.00300655	0.01461073
Ift122	376.378646	1.11400039	0.15574974	0.11050329	0.10201183	0.23669109
Ift140	2168.50415	1.27389421	0.34924548	0.06313435	8.81E-09	1.62E-07
Ift172	1314.11205	-1.171761	-0.2286784	0.07904458	0.00186189	0.00983134
Ift20	697.735508	1.11296247	0.15440494	0.08489983	0.04735721	0.13356359
Ift22	274.627547	1.33581103	0.41771594	0.2266068	0.00885625	0.03537528
Ift27	1048.20504	1.12184504	0.16587341	0.07860186	0.02305045	0.07679485
Ift43	274.810231	1.0857387	0.11867694	0.12410756	0.24776804	0.4386275
Ift46	2464.43956	1.01116518	0.0160187	0.06164585	0.78598315	0.88018569
Ift52	2984.74981	1.58164787	0.66142844	0.05504697	3.21E-34	3.96E-32
Ift57	1259.73258	1.01611529	0.0230641	0.07627946	0.7470416	0.8595626
Ift74	2429.22165	-1.0535637	-0.0752776	0.06555127	0.22647997	0.41511065
Ift80	1783.75077	1.01843845	0.0263588	0.06292573	0.66122112	0.80385712
Ift81	1010.13483	1.29764967	0.37590095	0.10117983	4.32E-05	0.00038436
Ift88	506.77566	1.20651069	0.2708407	0.13095411	0.01347118	0.04993225
Igbp1	819.642408	1.14608059	0.1967085	0.09123515	0.01754215	0.061922
Igdcc4	283.622589	2.03085394	1.02208648	0.15384406	2.14E-12	6.64E-11
Igf1r	2781.63293	-1.1857287	-0.245774	0.06108344	2.65E-05	0.00024561
Igf2bp2	5209.56031	1.56846103	0.64934969	0.05872547	2.38E-29	2.40E-27
Igf2bp3	462.639408	1.11916006	0.16241639	0.09565682	0.05816228	0.15616406
Igf2r	8445.72209	-1.2297821	-0.2984027	0.04705671	7.99E-11	2.02E-09
Igfbp4	110976.307	1.23935249	0.30958657	0.06307624	3.15E-07	4.43E-06
Igfbp6	5.90814488	-1.0132161	-0.018942	0.21057921	0.69745557	0.82741461
Ighmbp2	1480.81078	1.00689165	0.00990844	0.07213375	0.88476606	0.93815523

Igip	4.2548924	1.03042979	0.04324621	0.21986531	0.16087594	0.32727025
Igsf8	1550.36665	1.26869476	0.34334501	0.07497761	1.31E-06	1.65E-05
Igsf9	10.3537534	-1.0028275	-0.0040735	0.20336739	0.95082243	0.97557351
Ikbip	968.174747	1.0658212	0.09196543	0.08455772	0.23677307	0.42617157
Ikbkb	1466.56146	-1.0568543	-0.0797765	0.06870774	0.21945741	0.40654365
Ikbke	190.402663	1.07546075	0.10495487	0.17664147	0.35791413	0.55790704
Ikbkg	454.487231	-1.0028927	-0.0041672	0.09273526	0.95960543	0.98104669
Ikzf2	90.6031528	1.12110137	0.16491673	0.1954771	0.1789945	0.35406416
Ikzf4	13.0539586	1.01501692	0.02150378	0.2056097	0.74144248	0.85589602
Ikzf5	1016.73542	-1.1025721	-0.140873	0.07278327	0.03907224	0.1155391
Il11	106.412937	-1.0287303	-0.0408648	0.17317276	0.6954794	0.82606316
Il13ra2	105.80542	-1.3837056	-0.468537	0.2249268	0.00429141	0.01955635
Il15	44.3171707	1.04071623	0.05757675	0.20246616	0.50789737	0.69219621
Il15ra	276.664772	-2.1055288	-1.0741826	0.16426985	4.48E-12	1.33E-10
Il17d	633.724826	1.19767454	0.26023592	0.10352601	0.00468915	0.0210322
Il17f	4.10571169	-1.0591378	-0.0828903	0.23783811	0.01402323	0.05173894
Il17ra	930.661032	1.0586403	0.08221248	0.07205954	0.22546803	0.41408772
Il17rc	73.8626818	1.27685158	0.35259084	0.27338019	0.02746871	0.08744389
Il18	275.843264	-1.0108009	-0.0154989	0.11485693	0.88242755	0.93698481
Il18bp	18.5898365	1.00909485	0.01306179	0.20461066	0.84357997	0.91583959
Il19	5.90671149	1.03871433	0.05479894	0.22199129	0.18327035	0.35957929
Il1f6	3.68080834	1.01745723	0.02496816	0.21434412	0.49576395	0.68214138
Il1r1	2537.77297	-1.0692623	-0.0966158	0.05605001	0.07343502	0.18561613
Il1rap	2692.21575	-1.3953458	-0.4806227	0.067069	1.27E-13	4.46E-12
Il1rapl2	8.21314225	-1.0197893	-0.0282711	0.21171737	0.57327755	0.74165217
Il1rl1	210.389165	-2.4240324	-1.277409	0.18384436	2.15E-13	7.35E-12
Il1rl2	17.0056824	-1.4418208	-0.5278919	1.09146386	0.01177744	0.04495502
Il21r	3.03707862	-1.017123	-0.0244941	0.21454947	0.48370061	0.67301517
Il22ra1	534.100673	1.18954257	0.2504069	0.1189687	0.01393322	0.05146613
Il23a	1.89463757	1.0086433	0.01241607	0.21475009	0.59642175	0.75938402
Il25	24.0831631	1.04217881	0.05960283	0.20300204	0.49500363	0.68171472
Il27ra	2.31410325	1.00899116	0.01291353	0.21407197	0.64617916	0.79431485
Il31ra	17.0024995	-1.0532769	-0.0748848	0.21705552	0.32663135	0.52657281
Il33	32.2237461	-17.296709	-4.1124257	0.58459908	1.15E-13	4.08E-12
Il4i1	27.1065498	1.50034379	0.58529312	0.60690392	0.0119407	0.04537536
Il4ra	638.181547	-1.2722624	-0.3473963	0.11200752	0.00048339	0.00315771
Il6	76.1936493	-4.1281909	-2.0455097	0.28698934	4.97E-14	1.80E-12
Il6ra	36.0999528	1.0876946	0.12127354	0.2217014	0.23934458	0.42913999
Il6st	3119.43108	-1.1176919	-0.1605225	0.05938267	0.00473381	0.02118801
Il7	39.020884	-1.0868672	-0.1201757	0.21814453	0.250199	0.44150178
Il9r	8.31943194	-1.0065352	-0.0093977	0.20668569	0.87149396	0.93041135
Ildr2	933.748285	-1.0890434	-0.1230614	0.08898697	0.12762564	0.27766379
Ilf3	7812.38613	1.00412914	0.00594483	0.04858138	0.89371902	0.94436531
Ilk	3520.46952	1.07586246	0.10549365	0.05714142	0.05498338	0.14935053
Ilkap	1422.6196	-1.0043481	-0.0062594	0.06341093	0.9172607	0.95692723
Ilrun	1233.4999	1.03007989	0.04275623	0.07451486	0.54123764	0.71944113
Ilvbl	202.199334	1.05911038	0.08285295	0.16526377	0.45805619	0.65218404
Immp1l	354.943204	1.04300232	0.06074236	0.09873039	0.48935035	0.67718402
Immp2l	78.2198987	1.19646579	0.25877914	0.24651343	0.06593172	0.17200697
Immt	3192.519	-2.0756837	-1.0535866	0.05005617	1.76E-99	2.50E-96

Imp3	132.948698	-1.1007122	-0.1384374	0.19553865	0.23634952	0.42594252
Imp4	1381.79879	-1.0620592	-0.0868642	0.08785202	0.27696702	0.47255562
Impa1	1269.92752	-1.1120999	-0.1532864	0.0688321	0.01821797	0.06365853
Impa2	137.488125	-1.0268634	-0.0382443	0.1500679	0.7222522	0.84343968
Impad1	3418.38305	-1.1390514	-0.1878329	0.05153323	0.00016838	0.00128694
Impdh1	2398.19022	-1.0015217	-0.0021937	0.06227963	0.97119162	0.98673136
Impdh2	6454.7845	-1.0185365	-0.0264977	0.04788743	0.57042611	0.73959094
Impg2	3.65654418	1.0218776	0.03122241	0.21620367	0.35222913	0.55193336
Inafm2	28.3713496	-1.0493264	-0.0694635	0.20447306	0.4400659	0.63744228
Inava	3.51459731	-1.0754854	-0.1049879	0.25441858	0.00039671	0.0026605
Incenp	3785.47321	-1.0166571	-0.0238331	0.04773758	0.60814587	0.76850632
Inf2	2604.08897	-1.1655172	-0.2209702	0.07515926	0.00167315	0.00897113
Ing2	1275.87553	-1.0154088	-0.0220606	0.07928276	0.76367114	0.86923666
Ing3	1138.13997	-1.4019851	-0.487471	0.07095728	1.10E-12	3.51E-11
Ing4	297.15655	1.51675344	0.60098658	0.1398848	1.97E-06	2.40E-05
Ing5	1911.56991	-1.2099347	-0.2749292	0.06061285	2.32E-06	2.78E-05
Inha	42.6479706	1.00023531	0.00033943	0.18851997	0.99910448	0.99965074
Inhba	5.03112564	1.02072836	0.02959899	0.21457828	0.4570079	0.651244
Inhbb	1461.61664	-1.7134199	-0.7768788	0.09044045	7.38E-19	4.09E-17
Inip	2255.99224	1.05201281	0.07315228	0.06058979	0.20787187	0.39263324
Inka1	42.867931	1.05100033	0.07176312	0.18793101	0.49249481	0.67949091
Inka2	99.9786152	-1.5492344	-0.6315555	0.22792865	0.00047587	0.00311176
Ino80	4318.11438	-1.0380222	-0.0538373	0.04966982	0.2649742	0.45869182
Ino80b	249.965492	-2.1535687	-1.1067293	0.16227265	5.80E-13	1.92E-11
Ino80c	527.795994	-1.3049844	-0.3840326	0.1074532	7.74E-05	0.00065062
Ino80d	1226.23261	1.15310956	0.20552959	0.07788156	0.00461725	0.02077385
Ino80e	499.10412	-1.0322253	-0.0457579	0.09146524	0.57952236	0.74602366
Inpp1	499.759954	-1.0658066	-0.0919457	0.09325112	0.27313584	0.46893482
Inpp4a	721.349528	1.16077819	0.21509231	0.08724506	0.00707851	0.02934827
Inpp5a	934.313928	-1.0817187	-0.1133254	0.09523372	0.18353293	0.3599291
Inpp5b	1458.11155	1.16406147	0.21916725	0.07682125	0.0022472	0.01150087
Inpp5d	12.5840595	-1.0598095	-0.0838049	0.22938566	0.17540645	0.34901469
Inpp5e	563.251981	1.06411413	0.08965289	0.09192176	0.28101673	0.47686108
Inpp5f	727.865818	1.33446278	0.41625906	0.10361807	1.14E-05	0.0001173
Inpp5j	167.443577	1.18696852	0.24728167	0.17742724	0.05367367	0.14666377
Inpp5k	362.556737	-1.1371002	-0.1853594	0.1162521	0.0612082	0.16260662
Inppl1	2889.97601	1.00106069	0.00152944	0.06514009	0.98065433	0.99086465
Insig1	717.377336	-1.7396954	-0.7988347	0.12295456	6.83E-12	1.97E-10
Insig2	79.985541	1.0557313	0.07824269	0.18225683	0.46930055	0.66135814
Insr	1508.16841	-1.13808	-0.186602	0.0844259	0.01602745	0.05746198
Ints1	3437.9075	1.05542025	0.07781757	0.06313978	0.19650641	0.37705918
Ints10	2425.9407	1.03762455	0.05328452	0.07249446	0.43314545	0.63116747
Ints11	2202.00177	-1.0653267	-0.0912959	0.0605892	0.11528521	0.2583019
Ints12	1268.42487	1.00406215	0.00584858	0.07187917	0.93189145	0.9662104
Ints13	6188.03105	1.17808784	0.23644711	0.0534019	4.99E-06	5.50E-05
Ints14	2502.30653	1.01977448	0.02825013	0.06485186	0.6482786	0.79575017
Ints2	2776.94811	-1.0220206	-0.0314242	0.06569642	0.61456615	0.77342476
Ints3	3636.00192	1.12693919	0.17240967	0.05260087	0.00070098	0.00431918
Ints4	1285.69502	1.07917552	0.10992953	0.07454197	0.11507451	0.25802634
Ints5	534.691165	1.07592474	0.10557717	0.10797747	0.25863511	0.45088675

Ints6	2367.42044	1.10308676	0.14154627	0.05537376	0.00800396	0.03248756
Ints6l	665.022325	-2.0008153	-1.000588	0.10730058	7.31E-22	4.98E-20
Ints8	1372.89154	1.05456095	0.07664249	0.0676311	0.23168617	0.42085932
Ints9	530.017178	-1.1369724	-0.1851972	0.09981707	0.03634387	0.10908271
Intu	1511.32462	-1.0695137	-0.096955	0.06528776	0.11785811	0.26233925
Invs	444.44925	1.07583512	0.10545699	0.10960485	0.26485169	0.45869182
Ip6k1	979.319812	-1.1156159	-0.1578404	0.09502543	0.0629582	0.16606747
Ip6k2	648.596971	-1.2465109	-0.3178955	0.10938962	0.00106128	0.00612666
Ip6k3	1.98624065	-1.0295221	-0.0419748	0.22044041	0.08950299	0.215383
Ipmk	1754.33591	-1.2785584	-0.3545181	0.06539402	1.60E-08	2.84E-07
Ipo11	3746.91267	1.04195219	0.05928908	0.05001847	0.22344529	0.41172816
Ipo13	1505.74425	-1.0123413	-0.0176957	0.08456658	0.81920382	0.90247686
Ipo4	3921.42752	1.17100409	0.22774611	0.06859444	0.00045055	0.00296582
Ipo7	10627.0741	-1.0555163	-0.0779488	0.05895581	0.16954291	0.34052128
Ipo8	1252.62538	-1.0694478	-0.096866	0.08185232	0.1984803	0.37944957
Ipo9	5526.9809	1.00180246	0.00259806	0.05217127	0.96360069	0.982702
Ipp	629.143389	-1.0077898	-0.0111947	0.08950661	0.88969462	0.94127751
Ippk	654.676083	-1.3336627	-0.4153938	0.09205168	1.22E-06	1.55E-05
Iqcb1	872.949684	1.10127166	0.13917039	0.09060096	0.09024156	0.21655946
Iqcc	101.012257	1.07954559	0.11042416	0.1767899	0.33633129	0.53627133
Iqcd	110.31636	1.21447418	0.28033182	0.22006269	0.04791422	0.1348392
Iqce	296.645388	1.2740621	0.3494356	0.17350732	0.00913411	0.03629279
Iqcf1	2.669513	1.01031008	0.01479815	0.21463282	0.55964785	0.73310886
Iqcg	93.1766545	1.22360756	0.29114092	0.21862328	0.04161724	0.12121803
Iqch	18.6489344	1.13608682	0.18407308	0.33952166	0.00927237	0.03671687
Iqck	88.1997591	-1.1613901	-0.2158527	0.2276205	0.10082372	0.23457835
Iqcm	3.95100794	-1.0589993	-0.0827016	0.23687723	0.02513524	0.08197107
Iqcn	2.01089787	-1.012115	-0.0173732	0.21478879	0.51031282	0.69427955
Iqgap1	22223.7673	1.01519029	0.02175018	0.0381698	0.56250936	0.73502849
Iqgap2	3210.71255	-1.0854526	-0.1182968	0.05346743	0.02206223	0.07429472
Iqgap3	2099.21455	-1.0012219	-0.0017617	0.07445262	0.97921639	0.99034912
Iqsec1	2026.45504	1.24787592	0.31947449	0.06133264	6.12E-08	9.86E-07
Iqsec2	170.232898	1.11218785	0.15340048	0.13840371	0.1611722	0.32766479
Irak1	1373.83172	1.13459607	0.18217877	0.0699469	0.00572895	0.0246682
Irak1bp1	1204.53985	1.03214037	0.04563918	0.07464926	0.51452785	0.69820993
Irak2	426.335072	-1.1140621	-0.1558296	0.11144326	0.10402989	0.24032874
Irak4	636.910626	-1.1991971	-0.2620688	0.10300555	0.00426287	0.01946789
Ireb2	5307.71574	-1.0359989	-0.0510224	0.05432486	0.32388992	0.52337798
Irf1	498.499652	-1.4229757	-0.508911	0.1103773	5.49E-07	7.38E-06
Irf2	898.974078	-1.1538058	-0.2064004	0.07983297	0.00527355	0.02308756
Irf2bp1	252.4973	-1.0216901	-0.0309577	0.12629926	0.76111205	0.86757833
Irf2bp2	822.718548	1.04845825	0.06826942	0.09144726	0.40890853	0.6080936
Irf2bpl	429.68537	-1.0706936	-0.0985457	0.10527736	0.28231713	0.47824418
Irf3	235.709376	1.03672873	0.05203845	0.12978127	0.61785388	0.77526772
Irf7	23.6575666	-1.0213693	-0.0305046	0.19591969	0.71970726	0.84218964
Irf9	373.498479	1.38740015	0.47238395	0.11313864	4.76E-06	5.29E-05
Irgq	36.3796111	1.18732736	0.24771776	0.34213593	0.0546386	0.14866621
Irs1	883.039258	-1.1641112	-0.2192289	0.07824771	0.00258422	0.01297681
Irs2	1113.3487	-1.1202356	-0.1638022	0.06589547	0.00880215	0.03520309
Irs3	10.3275691	-1.0042209	-0.0060766	0.20516349	0.92071765	0.95920569

Irx2	250.009025	-1.2561446	-0.3290026	0.14261631	0.00525874	0.02304634
Irx3	608.799867	-1.0649847	-0.0908327	0.10121638	0.30785189	0.50610426
Irx5	145.228945	-1.094163	-0.1298277	0.1707452	0.26682031	0.46101539
Isca2	46.9011788	1.04757127	0.0670484	0.1901161	0.50923355	0.69352347
Isg20l2	1463.01486	-1.1682319	-0.2243267	0.07229196	0.00096956	0.00568723
Isoc1	809.585589	1.15333211	0.20580801	0.08551494	0.00876144	0.03510871
Isoc2a	354.482264	1.05486507	0.07705847	0.110333	0.41655397	0.6154621
Isoc2b	27.3828502	1.14742064	0.19839438	0.31599714	0.05790856	0.15564596
Ist1	959.299758	-1.035317	-0.0500725	0.07067863	0.45250227	0.64737034
Isy1	2517.10198	-1.0084709	-0.0121695	0.0629124	0.83916108	0.91400846
Isyna1	1415.16176	-1.0226118	-0.0322585	0.09169016	0.69618702	0.82644386
Itch	3378.79611	-1.0375785	-0.0532205	0.06333608	0.37842035	0.58047715
Itfg1	3807.73257	1.23136293	0.30025604	0.05019648	7.96E-10	1.71E-08
Itfg2	249.018839	-1.0265695	-0.0378313	0.14942232	0.72677638	0.8462872
Itga1	297.275095	1.12568609	0.17080457	0.17666729	0.15956934	0.32567047
Itga2b	68.3322809	1.10166385	0.13968408	0.19600677	0.23504716	0.42486043
Itga3	2219.07367	-1.173114	-0.2303432	0.07332205	0.00082209	0.00495109
Itga5	4560.14706	-1.0413938	-0.0585157	0.06118326	0.31737253	0.51628103
Itga6	4.96114088	-1.1240918	-0.1687599	0.33101602	2.64E-05	0.00024481
Itga7	305.14683	1.62315236	0.69879842	0.18493843	1.38E-05	0.00013887
Itga9	12.7802361	-1.1147488	-0.1567186	0.27887765	0.06841554	0.17648067
Itgae	5.30663629	1.01332116	0.01909148	0.21241903	0.65370581	0.79952514
Itgav	6207.71661	1.16965134	0.22607854	0.06024048	9.07E-05	0.00074351
Itgb1	42124.4421	1.10965759	0.15011457	0.04563683	0.00057434	0.00366591
Itgb1bp1	911.346861	1.18819497	0.24877158	0.07850308	0.00068337	0.00423251
Itgb1bp2	2.11527677	1.0037296	0.00537067	0.21359356	0.8549417	0.92247683
Itgb3bp	204.456498	1.16668122	0.22241042	0.17934711	0.07984735	0.19775664
Itgb4	462.560229	1.10316645	0.14165048	0.12405969	0.1677182	0.33770357
Itgb5	8980.11911	1.3694574	0.45360439	0.06499479	5.36E-13	1.78E-11
Itih5	858.693504	1.05846146	0.08196874	0.09317779	0.32835363	0.52808662
Itm2b	1325.92302	1.02274228	0.03244264	0.07883857	0.65880269	0.80221126
Itpk1	896.58835	1.32000243	0.40054059	0.08051019	1.41E-07	2.12E-06
Itpka	244.474678	1.11519337	0.15729389	0.13489973	0.14565129	0.30521725
Itpkb	831.542159	1.02639874	0.03759131	0.08636482	0.63520563	0.78732309
Itpkc	109.367769	-1.0994295	-0.136755	0.18478421	0.24502806	0.4354015
Itpr1	3991.12401	1.12336844	0.16783118	0.0527667	0.001007	0.00586882
Itpr2	5099.319	1.20428568	0.26817766	0.04747202	6.96E-09	1.30E-07
Itpr3	3841.91777	1.31839826	0.39878625	0.05480469	7.75E-14	2.79E-12
Itprid2	3620.2921	-1.0010793	-0.0015563	0.05112533	0.97609323	0.98895979
Itprip	293.872157	-1.3689486	-0.4530682	0.13786522	0.00015682	0.00120725
Itpripl2	212.798145	1.06940378	0.09680668	0.15112154	0.38417105	0.58537304
Itsn1	2455.71288	1.11203927	0.15320774	0.05448756	0.0035597	0.01682659
Itsn2	4393.21999	1.24017667	0.31054565	0.06932245	2.49E-06	2.97E-05
Ivd	1379.30405	-1.0273413	-0.0389155	0.08393598	0.6133566	0.77269695
Ivns1abp	8388.87265	-1.2007786	-0.2639702	0.0500661	5.85E-08	9.47E-07
Iws1	1634.70681	-1.0199939	-0.0285605	0.08129916	0.70339103	0.83145143
Iyd	3.21635777	1.00161238	0.0023243	0.21316574	0.94182293	0.97186658
Izumo1	48.3289875	3.04053551	1.60432544	0.49272307	5.40E-05	0.00046875
Izumo4	26.0459707	1.24470036	0.31579848	0.6312235	0.01423247	0.05229955
Jade1	5005.11556	1.01099976	0.01578266	0.05384382	0.76270979	0.86877882

Jade2	467.50125	-1.3063181	-0.3855063	0.12378541	0.00038058	0.00256322
Jade3	650.42839	1.07367994	0.102564	0.11276701	0.28842875	0.48491905
Jag1	404.713251	-1.3531273	-0.4362975	0.12885846	0.0001226	0.00097134
Jak1	10731.7	1.17121604	0.22800721	0.04699573	6.07E-07	8.12E-06
Jak2	2708.26893	-1.0243369	-0.0346902	0.0582261	0.53534973	0.71428289
Jak3	203.553581	-1.033578	-0.0476472	0.16179962	0.65904156	0.80234959
Jakmip3	2.24088364	-1.0069668	-0.0100161	0.21414217	0.70188775	0.83028433
Jarid2	843.095419	-1.1836456	-0.2432372	0.08190321	0.00135157	0.00746277
Jchain	60.4036468	-1.2386082	-0.3087199	0.2704117	0.04157308	0.12111693
Jdp2	142.980081	1.63029629	0.70513418	0.18254673	9.49E-06	9.92E-05
Jkamp	754.526535	1.05476245	0.07691811	0.10866769	0.4126706	0.6116291
Jmjd1c	7528.85928	1.0578485	0.08113302	0.04181493	0.04780652	0.13462333
Jmjd4	218.319578	1.00061741	0.00089046	0.13641311	0.99418151	0.99742843
Jmjd6	1467.08858	1.02794442	0.03976226	0.06932797	0.54613853	0.72332863
Jmjd7	124.908031	1.11570295	0.15795297	0.17585962	0.1882075	0.36607016
Jmjd8	259.078744	1.03218688	0.0457042	0.11922537	0.6470031	0.79487837
Jmy	2271.6345	1.00688018	0.00989201	0.07105026	0.8835591	0.93741859
Josd1	448.714543	-1.0874806	-0.1209896	0.0981143	0.16466735	0.33302634
Josd2	386.16843	-1.109086	-0.1493712	0.12288543	0.14233016	0.30042005
Jph2	1.8982436	-1.0032737	-0.0047153	0.21356977	0.86840547	0.92895817
Jpt1	2237.87135	-1.227424	-0.2956337	0.06872456	6.07E-06	6.56E-05
Jpt2	1827.83046	-1.0222918	-0.031807	0.07974511	0.66593605	0.80655133
Jrk	123.80242	-1.0163351	-0.0233762	0.16355032	0.82523882	0.90600868
Jrkl	531.163534	1.05725403	0.08032206	0.11576058	0.41215045	0.61119913
Jtb	1329.36393	-1.1007623	-0.1385029	0.07873573	0.05766788	0.15519444
Jun	1927.38377	-1.4775856	-0.5632417	0.09744836	9.16E-10	1.95E-08
Junb	1120.39551	-1.2261814	-0.2941724	0.09922293	0.00100768	0.00587012
Jund	885.893683	1.03511664	0.04979335	0.11459766	0.60641258	0.76723877
Jup	14.2948966	1.05767696	0.08089906	0.21892756	0.30475288	0.50275395
KARS	14274.0937	-1.0876037	-0.121153	0.03968587	0.00182018	0.0096463
KIF2C	3097.22163	1.05390574	0.07574584	0.05577862	0.15983662	0.32585903
Kank1	1604.08721	-1.1001301	-0.1376741	0.08078186	0.0647803	0.16998459
Kank2	1625.58599	1.4860346	0.57146771	0.07672989	1.25E-14	4.77E-13
Kank3	43.4665273	-1.0413245	-0.0584198	0.18456999	0.56711848	0.7378158
Kansl1	20904.9345	-1.021286	-0.030387	0.04483343	0.48956725	0.67718402
Kansl1l	554.35088	1.09642462	0.13280663	0.09079312	0.10606943	0.24359077
Kansl2	2221.07246	-1.1105035	-0.151214	0.06644505	0.01628798	0.05807876
Kansl3	1453.53111	1.07412928	0.10316764	0.06694903	0.1043588	0.24069793
Kat14	1391.90596	1.04596531	0.06483501	0.06554399	0.29897057	0.4968621
Kat2a	3935.22477	1.19840209	0.26111204	0.05365632	4.97E-07	6.75E-06
Kat2b	1051.35659	1.11029799	0.15094693	0.08910451	0.06222637	0.16459215
Kat5	1161.32026	1.01826816	0.02611754	0.07061245	0.69602552	0.82632873
Kat6a	8685.13605	-1.0915063	-0.1263204	0.05038452	0.00981997	0.03867009
Kat6b	1915.5924	1.04818829	0.06789789	0.06940051	0.30150001	0.49928806
Kat8	644.206289	1.02077057	0.02965864	0.09707796	0.73331292	0.85081284
Katna1	2581.84318	1.02925278	0.04159734	0.0537485	0.42462798	0.62300818
Katnal1	533.183138	1.2052173	0.26929328	0.09401993	0.00161866	0.00871221
Katnal2	14.4881327	-1.0363172	-0.0514657	0.20969817	0.4723597	0.66385249
Katnb1	715.570547	-1.0605944	-0.084873	0.0916913	0.30449117	0.50256561
Katnbl1	1157.08634	-1.0827535	-0.1147048	0.09300011	0.17008388	0.34122955

Kazald1	2237.96955	2.19966184	1.13728175	0.0851181	7.55E-42	1.30E-39
Kbtbd11	25.5910912	1.04903385	0.06906124	0.20388268	0.44690715	0.64302826
Kbtbd2	1391.48293	-2.1559572	-1.1083285	0.07539142	4.28E-50	1.22E-47
Kbtbd3	103.125984	1.33619374	0.41812921	0.24311162	0.01090107	0.04217765
Kbtbd4	630.177139	1.08458132	0.11713823	0.0814174	0.11943423	0.26483511
Kbtbd6	48.1810713	-1.3325606	-0.4142012	0.34650532	0.02064647	0.07037821
Kbtbd7	294.489881	1.00314014	0.00452316	0.11468903	0.96447529	0.9828119
Kbtbd8	515.015522	-1.0973581	-0.1340344	0.10461305	0.14222872	0.30025542
Kcmf1	1564.44438	-1.5848828	-0.6643761	0.08340156	1.70E-16	7.82E-15
Kcnab2	3506.75556	1.05197514	0.07310061	0.05562513	0.17322564	0.34596515
Kcnab3	6.76632121	1.00098758	0.00142407	0.20990214	0.97810078	0.99000245
Kcnip3	106.877388	1.00530061	0.00762697	0.15336021	0.94440195	0.97295716
Kcnj12	3.7711454	-1.0686322	-0.0957653	0.24757635	0.00045965	0.00301953
Kcnj14	22.4052776	-1.1660702	-0.2216547	0.33885967	0.0504261	0.13990866
Kcnk5	116.135986	-2.1934994	-1.1332343	0.20116053	1.11E-09	2.31E-08
Kcnn4	223.067961	1.73068518	0.79134331	0.20039442	5.56E-06	6.05E-05
Kcnq5	696.299011	-1.077364	-0.1075058	0.0803271	0.14774631	0.30824596
Kcp	4.07956425	1.0112542	0.0161457	0.21270254	0.67684234	0.81348715
Kctd1	134.144762	1.02893564	0.04115275	0.14299841	0.70235815	0.83058637
Kctd10	1902.09832	1.00117548	0.00169486	0.05672636	0.97585139	0.98894008
Kctd11	23.0426461	1.12442276	0.16918457	0.26740467	0.11150455	0.25240736
Kctd12b	138.744633	-1.1277369	-0.1734305	0.17574058	0.15274461	0.31528496
Kctd13	714.549434	-1.0149146	-0.0213583	0.0757889	0.76277559	0.86877882
Kctd15	678.355795	1.19070338	0.25181406	0.12077167	0.01455325	0.05327556
Kctd17	403.184696	1.16315477	0.21804308	0.11603445	0.02840539	0.08956603
Kctd18	285.471721	1.16308653	0.21795843	0.12215981	0.03439009	0.10464062
Kctd2	96.0621676	1.35782445	0.44129697	0.20972598	0.00474157	0.02121149
Kctd20	849.950324	1.09270919	0.1279095	0.08869723	0.11217719	0.25330611
Kctd21	18.2608376	1.04019283	0.05685099	0.21074587	0.4444484	0.64099039
Kctd3	2280.90476	1.09952682	0.1368828	0.06934838	0.03677638	0.11025168
Kctd5	690.767007	-1.0361655	-0.0512545	0.11667153	0.60166294	0.76372192
Kctd6	265.028716	-1.1180607	-0.1609986	0.14696256	0.15334185	0.31626294
Kctd7	153.656447	1.03504136	0.04968842	0.1410152	0.64445071	0.79305677
Kctd9	1797.86924	-1.4361088	-0.522165	0.07721013	2.01E-12	6.28E-11
Kdelr2	4852.17964	1.0265193	0.03776076	0.05452282	0.47399731	0.66475866
Kdelr3	551.428327	1.25925425	0.33256959	0.1300792	0.0027572	0.01367394
Kdm1a	4162.21237	-1.1364441	-0.1845267	0.05735795	0.00081758	0.00492624
Kdm1b	2778.51455	1.18492091	0.24479077	0.05022934	5.25E-07	7.10E-06
Kdm2a	3804.68524	1.0099471	0.01427973	0.05093973	0.76806708	0.8713089
Kdm2b	1573.40615	1.12286604	0.16718582	0.07106462	0.01241058	0.04675869
Kdm3a	1029.36251	-1.5710898	-0.6517656	0.08152651	1.38E-16	6.39E-15
Kdm3b	1823.73009	1.08528468	0.11807353	0.07988445	0.11066857	0.25106223
Kdm4a	2928.676	1.0608197	0.08517948	0.05682398	0.11969607	0.26521935
Kdm4b	817.116525	-1.0398301	-0.0563479	0.09420939	0.50545245	0.6898108
Kdm4c	847.831261	1.12801798	0.17379006	0.08719479	0.02923218	0.09180243
Kdm4d	1.96720651	-1.0055061	-0.0079219	0.21377484	0.77752026	0.87599248
Kdm5a	3692.49884	1.06469442	0.09043942	0.0510317	0.06798678	0.17579687
Kdm5b	2143.31869	-1.1720278	-0.2290067	0.06666163	0.00029001	0.00203005
Kdm5c	7114.11361	-1.0469607	-0.0662073	0.0460746	0.16032598	0.32651443
Kdm6a	2289.92584	1.00234722	0.00338235	0.06007713	0.95391603	0.97765136

Kdm6b	1116.56574	-1.2112876	-0.2765415	0.10048376	0.00215203	0.01108215
Kdm7a	353.648151	-1.1583201	-0.212034	0.14347624	0.06181576	0.16384437
Kdm8	388.428477	-1.0552018	-0.0775189	0.12073853	0.44030787	0.63768727
Kdsr	1284.53349	-1.0869879	-0.1203358	0.07208229	0.07548992	0.18961292
Keap1	1492.36398	-1.0439748	-0.0620868	0.06764804	0.33244703	0.53226428
Khdc4	1062.30772	-1.0782721	-0.1087213	0.07489228	0.12054949	0.26647781
Khdrbs1	2621.17165	-1.0828063	-0.1147752	0.06228767	0.05335915	0.14608814
Khdrbs3	5.49634017	1.02312543	0.03298302	0.21472444	0.4439911	0.64065908
Khk	121.904777	-1.0722257	-0.1006087	0.17469474	0.37286604	0.57443343
Khnyn	290.003137	1.13067478	0.17718403	0.15211436	0.12539581	0.27419057
Khsrp	9454.13683	-1.1634743	-0.2184394	0.05727375	7.20E-05	0.00060819
Kidins220	3017.65913	-1.0089557	-0.0128628	0.05867801	0.8191982	0.90247686
Kif13a	1421.01278	1.06286505	0.08795843	0.07868639	0.22914637	0.41766719
Kif13b	738.047916	1.06597063	0.09216769	0.08298103	0.22732007	0.41591083
Kif14	6118.53598	-1.0503628	-0.0708878	0.05694591	0.19578824	0.37624472
Kif15	10162.2109	1.04479054	0.06321374	0.04918437	0.18670426	0.36393578
Kif16b	2194.21817	1.07355808	0.10240025	0.0610416	0.07976301	0.19775664
Kif18a	1924.10409	1.02596723	0.03698465	0.0687836	0.57088659	0.73973875
Kif18b	4972.41537	1.10213995	0.14030743	0.05394398	0.00707932	0.02934827
Kif1b	5399.3495	1.06612553	0.09237731	0.04839556	0.0498659	0.13874622
Kif1c	3768.55206	1.05938367	0.08322517	0.05466365	0.11514868	0.2581023
Kif20a	3712.62378	-1.0842705	-0.1167247	0.0492874	0.01483152	0.05411329
Kif20b	7766.61667	-1.1221263	-0.1662351	0.07297659	0.01499615	0.0545121
Kif21a	3499.87884	1.19494642	0.25694593	0.05735585	3.32E-06	3.81E-05
Kif22	3427.70783	-1.0756544	-0.1052146	0.0614558	0.07327085	0.1852743
Kif23	4706.1847	-1.0674813	-0.0942108	0.06533547	0.12903562	0.27970309
Kif24	281.67606	1.33821961	0.42031489	0.15208809	0.00098833	0.00577312
Kif26b	3.36404603	-1.0696125	-0.0970883	0.24632281	0.01477018	0.05393558
Kif27	16.4569243	-1.1037644	-0.1424322	0.27244032	0.05684783	0.15340651
Kif2a	3303.27662	-1.0597546	-0.0837302	0.07741883	0.24483012	0.4352309
Kif3a	1414.88026	1.11629751	0.15872158	0.07151769	0.01833409	0.06392704
Kif3b	782.84605	1.06279913	0.08786895	0.08830304	0.27465175	0.4699825
Kif3c	814.911412	-1.0253318	-0.0360908	0.07714978	0.61545143	0.77387521
Kif4	2942.1332	-1.0533094	-0.0749292	0.0646552	0.22327995	0.41154189
Kif5a	13.5489088	1.00250154	0.00360445	0.20545073	0.95685006	0.97901352
Kif5b	14426.5692	-1.0589546	-0.0826407	0.05135684	0.0978803	0.22939017
Kif7	866.353656	1.04966654	0.06993109	0.07941308	0.34297618	0.54247745
Kif9	58.6899967	1.17964065	0.23834744	0.25902502	0.08152435	0.20071631
Kifbp	2509.83745	-1.0081589	-0.011723	0.05294456	0.81862565	0.90215025
Kifc2	38.7003834	1.06522615	0.09115975	0.20611321	0.35694637	0.55694068
Kifc3	330.095042	1.24838239	0.32005991	0.15589543	0.01001316	0.03929797
Kin	527.678176	-1.0374203	-0.0530005	0.09448424	0.53014764	0.71061904
Kirrel	3102.68274	1.16296399	0.21780643	0.0640344	0.00035712	0.00242434
Kiss1r	6.48210609	1.01631354	0.02334555	0.20941863	0.6728046	0.81086907
Kitl	3209.87723	1.22130043	0.28841814	0.06135072	9.83E-07	1.27E-05
Kiz	1030.51576	1.16920437	0.22552713	0.09528625	0.00883572	0.03531531
Klc1	4574.96921	1.06527815	0.09123018	0.04932695	0.05743899	0.15470846
Klc2	2008.87329	1.08587419	0.11885697	0.07378552	0.08533306	0.20771252
Klc3	10.0629773	-1.0232153	-0.0331097	0.21152517	0.54301678	0.72068431
Klc4	917.480207	1.13452682	0.18209071	0.08008811	0.01402786	0.05174112

Klf1	1.65833789	1.00242652	0.00349648	0.21384118	0.89454992	0.9447757
Klf10	502.860578	1.09394851	0.12954484	0.09327719	0.12288051	0.27041734
Klf11	1003.58954	1.20581853	0.27001281	0.08213016	0.00039868	0.00267109
Klf13	661.016146	-1.2693006	-0.3440337	0.126706	0.00161246	0.00868613
Klf16	142.818934	-1.1027929	-0.1411619	0.1849991	0.23367323	0.42318832
Klf2	87.0586531	1.05777409	0.08103154	0.18066137	0.45783134	0.65208132
Klf3	1213.42392	1.09197429	0.12693888	0.09647914	0.14083167	0.29799368
Klf4	2523.37655	-1.1715192	-0.2283806	0.06404956	0.00020556	0.00153003
Klf5	2913.19522	-1.193087	-0.2546992	0.05835719	5.69E-06	6.18E-05
Klf6	1463.37629	-1.0072056	-0.0103583	0.08500674	0.89383352	0.9444084
Klf7	394.616882	-1.2130901	-0.2786867	0.11291813	0.00475981	0.02125243
Klf8	88.867414	-1.031005	-0.0440514	0.16611389	0.68189297	0.81683798
Klf9	232.675513	-1.2878688	-0.3649857	0.14575574	0.00258885	0.01298987
Klhdc1	118.607624	-1.2071305	-0.2715817	0.20817837	0.0496898	0.1384656
Klhdc10	1593.77987	1.11062597	0.15137303	0.08718403	0.0571794	0.15410648
Klhdc2	993.52395	-1.0061635	-0.0088647	0.07676435	0.9004719	0.9485077
Klhdc3	654.488014	1.0197218	0.0281756	0.08834313	0.72289324	0.84385629
Klhdc4	1684.35565	1.00178777	0.00257691	0.0870682	0.9778577	0.98983462
Klhdc8b	5.50178269	1.01030316	0.01478827	0.21111118	0.74401847	0.85736145
Klhdc9	43.4690243	1.10127805	0.13917876	0.23358013	0.18805288	0.36584976
Klhl1	2.56740684	-1.0508825	-0.0716014	0.23147121	0.02755147	0.08762026
Klhl10	38.82587	2.18817539	1.12972838	0.39228411	0.00020108	0.00150197
Klhl11	1584.31771	-1.2458116	-0.317086	0.07769232	1.34E-05	0.00013513
Klhl12	1165.13452	-1.042556	-0.0601249	0.06448506	0.32685264	0.52666444
Klhl13	4.33009881	-1.0032797	-0.0047238	0.21155451	0.90398125	0.94995896
Klhl15	265.918933	-1.0572787	-0.0803558	0.1141988	0.40679058	0.60628124
Klhl18	318.005562	1.04469625	0.06308354	0.10726228	0.4981648	0.68441561
Klhl2	1267.67292	-1.0394717	-0.0558505	0.08696458	0.48188655	0.67185096
Klhl20	1880.10275	1.06799421	0.09490383	0.06328216	0.11594522	0.25934315
Klhl21	1750.42456	-1.1117531	-0.1528364	0.08223226	0.04327529	0.12508042
Klhl22	1708.07966	1.1242602	0.16897597	0.06799323	0.00858217	0.03448484
Klhl23	871.879813	1.02373048	0.03383594	0.07901301	0.64594846	0.79410746
Klhl24	539.539764	1.12766472	0.17333818	0.09855127	0.04811802	0.13523633
Klhl25	203.857975	-1.0879593	-0.1216246	0.13064907	0.24950923	0.44085701
Klhl26	98.9407173	-1.0369317	-0.0523209	0.1597074	0.63077554	0.78464116
Klhl28	946.676263	-1.0952151	-0.1312142	0.08835575	0.10205718	0.23671058
Klhl3	17.6324304	1.00405082	0.00583229	0.19902384	0.94081536	0.97129632
Klhl33	5.02473086	-1.0080134	-0.0115148	0.21201696	0.7672545	0.87116991
Klhl36	170.072274	1.19862068	0.26137517	0.16622788	0.03699762	0.11068182
Klhl4	419.487476	-1.4599551	-0.545924	0.11873125	5.25E-07	7.10E-06
Klhl42	1093.13403	1.00413141	0.00594809	0.08483674	0.94004078	0.97104446
Klhl5	2654.59949	-1.1307713	-0.1773071	0.05730724	0.00128316	0.00712804
Klhl8	224.850046	1.07147854	0.09960295	0.13878561	0.35777721	0.55782936
Klrg2	263.033625	-1.2255744	-0.2934581	0.13289538	0.00827874	0.03344489
Kmt2a	12265.0425	-1.0466387	-0.0657635	0.05760594	0.23960129	0.42927168
Kmt2b	1516.94494	1.09535781	0.13140222	0.08395853	0.0881325	0.21311709
Kmt2c	5196.55212	-2.0029552	-1.0021301	0.05209457	1.45E-83	1.24E-80
Kmt2d	7081.41812	-1.0864158	-0.1195764	0.05245418	0.01859784	0.06468594
Kmt2e	3998.04383	1.14123981	0.19060198	0.07070396	0.00422401	0.01934556
Kmt5a	6346.79292	1.1172843	0.15999634	0.04502738	0.00026926	0.00191309

Kmt5b	1036.03463	1.04399564	0.06211569	0.07439684	0.37355818	0.57508476
Kmt5c	115.458607	1.260792	0.33433028	0.22836031	0.02566284	0.08320449
Knl1	5414.66597	1.06119476	0.08568945	0.05495792	0.10708377	0.24502127
Knop1	2615.52043	-1.1566	-0.2098901	0.06856979	0.00121146	0.00680351
Knstrn	2049.13671	-1.0513566	-0.072252	0.06633598	0.25146326	0.44278273
Kntc1	4518.2666	1.08971998	0.12395746	0.04796091	0.00780682	0.03179822
Kpna1	1323.917	1.02520403	0.03591105	0.0748595	0.60931392	0.76937519
Kpna3	5902.86061	-1.0009969	-0.0014376	0.06351832	0.9798701	0.99079336
Kpna4	1912.96942	1.04049098	0.05726446	0.07703201	0.42601314	0.62446829
Kpna6	9118.41286	-1.0077829	-0.0111848	0.04639223	0.80447228	0.89215565
Kpnb1	34083.6775	1.0144865	0.02074966	0.04534294	0.63641031	0.78820613
Kptn	147.437438	1.10765455	0.14750802	0.16399946	0.20919318	0.39448913
Krcc1	273.042247	-2.1815517	-1.1253547	0.13861587	3.27E-17	1.59E-15
Kremen1	297.113865	1.078721	0.10932178	0.12471441	0.28780152	0.48407793
Kremen2	2.15192828	1.02758914	0.03926355	0.22012734	0.056483	0.15256661
Kri1	1708.91807	-1.1274121	-0.173015	0.07709222	0.01596816	0.05726543
Krit1	4046.85702	-1.1612208	-0.2156423	0.05386402	3.39E-05	0.00030809
Krr1	1654.3922	1.00509542	0.00733247	0.07841765	0.92081698	0.95921243
Krt5	8.04712129	-1.027776	-0.0395259	0.21613025	0.36183192	0.56235316
Krt7	8.32510877	-1.0155125	-0.022208	0.20837137	0.69762418	0.82741461
Krt78	51.2412494	1.11401719	0.15577149	0.22884888	0.17709741	0.35130443
Krt80	638.969544	-1.5570186	-0.6387862	0.11406901	2.27E-09	4.60E-08
Krtcap2	482.652979	-1.0141642	-0.0202912	0.11658565	0.83351824	0.91080514
Ksr2	9.98956092	-1.0090641	-0.0130179	0.20532876	0.83529017	0.91187361
Ktn1	8617.03846	-1.0244719	-0.0348805	0.05209763	0.49237663	0.67949091
Kyat1	247.63835	-1.0973196	-0.1339838	0.13704054	0.21535744	0.40156169
Kynu	7.71585221	8.04605909	3.00828233	1.10888237	0.00036414	0.00246153
L1td1	60.7845811	-2.2216802	-1.1516512	0.28283695	2.52E-06	3.00E-05
L2hgdh	601.788484	1.06977711	0.09731024	0.09313622	0.24632495	0.43661583
L3hypdh	244.030741	-1.2095461	-0.2744658	0.15550562	0.02423691	0.07992673
L3mbtl1	24.0610983	-1.0252803	-0.0360184	0.19409201	0.68553332	0.81956079
L3mbtl2	735.581098	1.07658444	0.10646148	0.09163202	0.18518121	0.36221885
L3mbtl3	1697.32278	-1.151973	-0.2041069	0.07078779	0.00219203	0.01125897
LAMP1	8859.485	1.21183949	0.27719862	0.0621669	3.28E-06	3.79E-05
LGALS1	10551.119	-1.0567883	-0.0796863	0.06204535	0.1776148	0.35180797
LSM1	489.935762	-1.0695619	-0.09702	0.0975904	0.26416997	0.45767849
LTO1	193.695609	1.24755592	0.31910448	0.20844826	0.02664311	0.08555984
Lacc1	1196.77119	1.09580849	0.13199569	0.07551222	0.06142174	0.16308893
Lactb	193.911104	1.23954315	0.30980849	0.17274176	0.01825742	0.06374074
Lactb2	554.436901	1.06687112	0.09338591	0.09428128	0.27049077	0.46572403
Lad1	11.8174365	-1.0375977	-0.0532471	0.21444734	0.39244763	0.59367669
Lag3	6.1269937	-1.0057737	-0.0083058	0.20959814	0.86451966	0.92717978
Lage3	243.827677	1.05754563	0.08071991	0.16243277	0.46981207	0.66178718
Lama2	3248.72322	1.14509248	0.19546412	0.08838234	0.01526942	0.05533274
Lama3	151.437291	-1.796716	-0.8453624	0.22593611	1.23E-05	0.00012464
Lama5	12639.1543	1.36299815	0.4467836	0.06349617	3.82E-13	1.29E-11
Lamb1	16364.6622	1.04107533	0.05807446	0.03708411	0.11200538	0.25318314
Lamb2	2339.84069	1.07729223	0.10740966	0.07111657	0.10887852	0.24799677
Lamc1	25532.8882	1.02207826	0.03150567	0.03651877	0.38236779	0.58400032
Lamc2	140.883573	-2.9292734	-1.5505428	0.1744138	3.89E-20	2.35E-18

Lamp2	5057.02946	1.10332837	0.14186223	0.06485093	0.02146906	0.07263213
Lamtor1	379.020251	1.45498008	0.5409994	0.15552141	6.12E-05	0.00052607
Lamtor2	546.997144	1.25365252	0.32613752	0.13844396	0.00482177	0.02146918
Lamtor3	741.772031	1.04185669	0.05915685	0.0842759	0.44389137	0.64063187
Lamtor4	135.283842	1.09643474	0.13281995	0.17692922	0.26003784	0.45216457
Lancl1	2979.60974	1.20056021	0.26370776	0.05912575	3.53E-06	4.03E-05
Lancl2	196.018274	-1.9122978	-0.9353072	0.20553572	3.36E-07	4.71E-06
Lap3	4104.88157	-1.0637912	-0.089215	0.05155679	0.07420397	0.18725432
Laptm4a	1294.95298	-1.0223516	-0.0318914	0.06213691	0.59127661	0.75440771
Large1	1139.77143	1.01357905	0.01945861	0.07002607	0.76931108	0.87172711
Larp1	7715.88608	-1.0340202	-0.0482643	0.0451041	0.27480802	0.47009574
Larp4b	2939.70949	-1.1197009	-0.1631134	0.05733605	0.00304263	0.01476926
Larp6	450.467058	-1.2713758	-0.3463905	0.11211883	0.00050323	0.00327395
Lars	4362.70669	1.0464244	0.06546808	0.06561425	0.29857207	0.49638308
Lars2	1194.63948	-1.1010695	-0.1389055	0.06967567	0.03441921	0.10470435
Las1l	3294.33967	-1.0482958	-0.0680459	0.05726364	0.21680737	0.40303328
Lasp1	11374.7576	1.03591349	0.05090353	0.05446021	0.33445238	0.53440627
Lat	4.54012384	-1.0199596	-0.0285119	0.21391294	0.49498157	0.68171472
Lat2	6.08415882	-104.30925	-6.7047233	2.70512475	3.02E-06	3.52E-05
Lats1	4484.4532	-1.0244949	-0.0349128	0.05111807	0.47700966	0.6673759
Lax1	1.66556092	-1.0087317	-0.0125425	0.21396466	0.66560817	0.8065116
Layn	602.26212	1.01436363	0.02057492	0.09174771	0.80498013	0.89248705
Lbh	28.0979074	1.01631006	0.02334062	0.1956562	0.78490114	0.87956967
Lbr	4178.6656	-1.1056624	-0.1449109	0.05134812	0.0035122	0.0166388
Lca5	351.253205	1.29085379	0.3683256	0.13178233	0.00115553	0.00656612
Lca5l	26.0875215	1.51681822	0.6010482	0.66271905	0.01190508	0.0453292
Lcat	213.63448	1.11849083	0.16155343	0.14224898	0.14728419	0.30768531
Lck	31.0287098	-1.0425462	-0.0601113	0.19616922	0.52298401	0.70506457
Lclat1	2200.99594	-1.1342952	-0.1817961	0.06419604	0.00291586	0.0142511
Lcmt1	785.059312	-1.0587232	-0.0823254	0.07764451	0.25437465	0.44594763
Lcmt2	136.026629	-1.0893162	-0.1234228	0.18529843	0.28606373	0.4825489
Lcor	462.279766	-1.1373857	-0.1857215	0.11084873	0.05215683	0.143591
Lcorl	254.890625	1.00865515	0.01243302	0.11582584	0.89565066	0.94539263
Lcp1	32.2065473	-2.8758291	-1.5239779	0.52575335	0.00013668	0.00106894
Lct	2.06171178	1.00650004	0.00934722	0.21408579	0.72719444	0.84644821
Ldb1	2157.08739	-1.0605524	-0.0848159	0.07900763	0.2468714	0.43746336
Ldha	28318.1848	-1.394466	-0.4797128	0.05218522	6.72E-21	4.20E-19
Ldhc	33.8862882	1.07507664	0.10443951	0.22881075	0.22008067	0.40733182
Ldhd	8.52893745	-1.0615696	-0.086199	0.2338635	0.11059436	0.25101236
Ldlr	5282.53516	1.02274335	0.03244416	0.04508964	0.45663756	0.65096006
Ldlrad3	602.958231	-1.0669227	-0.0934556	0.09200252	0.26002149	0.45216457
Ldlrap1	182.669925	-1.0473835	-0.0667898	0.15140018	0.54239532	0.72027106
Lec35	74.2443708	1.09526161	0.1312755	0.20256472	0.25126553	0.44256048
Lemd2	1469.22342	1.37144102	0.45569258	0.08013294	2.33E-09	4.68E-08
Lemd3	960.074871	-1.0833807	-0.1155403	0.081488	0.1237436	0.27203629
Leng1	158.118853	-1.0234947	-0.0335036	0.12620056	0.7425694	0.85638464
Leng8	596.60605	1.06073239	0.08506073	0.10425217	0.35110598	0.55072051
Leo1	3243.44779	-1.1185679	-0.1616528	0.05700344	0.00318997	0.01536798
Leprot	109.63638	1.08129624	0.11276183	0.16440441	0.32657361	0.52657281
Leprotl1	247.094484	-1.0130766	-0.0187432	0.14285151	0.8598712	0.9249689

Letm1	5496.66909	1.03003335	0.04269105	0.04602045	0.34254298	0.54199302
Letm2	159.129189	1.2937176	0.37152274	0.19544531	0.01023586	0.04001263
Letmd1	848.736098	1.09509434	0.13105516	0.08058943	0.07856323	0.19534763
Lfng	425.654562	1.03245172	0.04607432	0.10745727	0.62830256	0.78262892
Lgals3	9431.93857	1.5597192	0.64128632	0.04864938	1.35E-40	2.14E-38
Lgals3bp	1789.24617	1.17198883	0.22895882	0.06586385	0.0002542	0.0018232
Lgals8	800.606136	-1.1222371	-0.1663775	0.08187929	0.02740607	0.08730956
Lgmn	6171.79371	-1.0485738	-0.0684283	0.04585913	0.12609995	0.27513887
Lgr4	1162.51039	1.28742164	0.36448463	0.09489812	3.00E-05	0.0002755
Lhfp	960.035587	1.19814684	0.26080473	0.07718765	0.00030706	0.00212734
Lhfpl2	382.55302	-1.0429513	-0.0606718	0.10050657	0.49062597	0.67791163
Lhpp	490.854435	1.14819634	0.19936937	0.09913938	0.02403024	0.0793785
Lhx6	269.212415	1.13754726	0.18592648	0.12571533	0.07482814	0.18835695
Lhx9	151.941271	-1.1946347	-0.2565695	0.18837016	0.05150789	0.14214047
Lias	750.193796	1.14321109	0.19309182	0.08310003	0.01170755	0.0447549
Lif	4437.22383	-1.2598471	-0.3332487	0.05982731	8.45E-09	1.56E-07
Lig1	3173.55922	1.11378739	0.15547386	0.05142611	0.00180464	0.00958038
Lig3	3300.21657	1.08335937	0.11551189	0.05057951	0.01856845	0.06461881
Lig4	1035.50025	-1.0410028	-0.057974	0.07442805	0.40507878	0.60450364
Lima1	3691.38971	1.01386361	0.01986359	0.05527633	0.71994314	0.84225325
Limd1	1252.5826	-1.1226327	-0.1668861	0.09029167	0.04108082	0.1200651
Limd2	260.47668	1.06841495	0.09547208	0.12529499	0.35217774	0.55192034
Lime1	63.5687457	1.04409455	0.06225236	0.18284931	0.55165852	0.7277643
Limk1	936.820659	-1.0387828	-0.0548941	0.08776172	0.49321128	0.6799904
Limk2	371.629453	1.09816666	0.13509702	0.10571481	0.14786195	0.30833658
Lims1	6863.16033	1.02436778	0.03473379	0.04933244	0.46901661	0.66135814
Lin37	118.048123	1.08894326	0.12292879	0.19127959	0.28732342	0.48368923
Lin52	172.033404	1.04455068	0.06288249	0.14329945	0.5625516	0.73502849
Lin54	3321.36285	-1.0732712	-0.1020147	0.05476469	0.05359112	0.1464721
Lin7b	1.89552682	1.02353447	0.03355969	0.21847953	0.12815731	0.27838166
Lin7c	8835.09943	-1.110078	-0.150661	0.04781951	0.00119883	0.00675331
Lin9	1593.4975	1.09442881	0.13017811	0.07375458	0.05965513	0.15930419
Lins1	306.190816	1.0544976	0.07655581	0.11335693	0.42762684	0.62570864
Lipa	1761.24456	1.05037518	0.07090473	0.06482218	0.25073657	0.44205003
Lipe	225.40619	1.02401637	0.03423878	0.12898875	0.74135098	0.85589602
Liph	12.9820951	-1.0636016	-0.0889579	0.22680618	0.22352285	0.41181183
Lipn	2.7484017	-1.0313491	-0.0445327	0.22165693	0.02715247	0.08673893
Lipt1	82.7548404	1.06815752	0.09512441	0.18667111	0.3875655	0.58900393
Lipt2	2927.7137	-1.1718382	-0.2287734	0.07155282	0.00069213	0.00427696
Litaf	401.73785	-1.1005256	-0.1381927	0.12271695	0.17432369	0.34756268
Lix1l	1388.82305	1.15181222	0.20390554	0.07304038	0.00293981	0.01436268
Llgl1	727.845102	1.07326528	0.10200671	0.11462851	0.2961832	0.49378695
Llph	933.688145	-1.3995692	-0.4849828	0.10602855	7.35E-07	9.73E-06
Lman1	5139.4736	1.1062602	0.14569076	0.04733593	0.0015682	0.00846195
Lman2	6100.64516	1.04959204	0.06982868	0.05826238	0.21361479	0.39935914
Lman2l	1230.01596	1.03473597	0.04926269	0.06922205	0.4520229	0.6470374
Lmbr1	223.23526	-1.7556579	-0.8120117	0.15431949	1.15E-08	2.09E-07
Lmbr1l	73.809845	-1.0711364	-0.0991422	0.19543694	0.3563563	0.55646281
Lmbrd1	959.20397	-1.0325215	-0.0461718	0.09679716	0.59221959	0.75523473
Lmbrd2	1074.36426	1.02708674	0.03855803	0.08407025	0.61895506	0.7761173

Lmf1	287.55891	1.13263346	0.17968105	0.14261304	0.10851221	0.24733832
Lmf2	1202.77499	1.02029304	0.02898357	0.08656514	0.71239002	0.83737531
Lmln	89.7443222	1.31177518	0.39152048	0.2869435	0.02026066	0.06933985
Lmna	9240.23368	-1.0545621	-0.0766441	0.04119924	0.05770095	0.15521822
Lmnb1	9010.20031	-1.2321888	-0.3012233	0.04617997	2.48E-11	6.71E-10
Lmnb2	1367.68015	-1.1489208	-0.2002793	0.0710936	0.00276639	0.01370033
Lmo2	54.8653397	1.8549137	0.89135207	0.31173492	0.00024243	0.00175553
Lmo4	1394.69602	1.00149613	0.00215685	0.07140958	0.97511863	0.98867578
Lmo7	121.178598	-1.5148725	-0.5991964	0.23009549	0.00081457	0.00491352
Lmtk2	5102.25928	1.00891459	0.01280405	0.04615944	0.7768145	0.87546517
Lmtk3	118.534739	1.08456681	0.11711893	0.17051809	0.31085345	0.50934161
Lmx1b	60.7111106	1.09461019	0.1304172	0.19937639	0.25726743	0.44948115
Lnpep	3366.75079	1.0692243	0.09656453	0.05685833	0.07783432	0.19424463
Lnpk	1436.16619	-1.0084407	-0.0121262	0.06391732	0.84185378	0.91522931
Lnx1	2.77667392	-1.0479193	-0.0675277	0.23036673	0.01032724	0.04028377
Lnx2	806.785727	1.0323337	0.0459094	0.08575777	0.55893498	0.73259433
Lonp1	4930.2064	-1.049073	-0.0691151	0.05307686	0.17703019	0.35130443
Lonrf1	481.414929	1.05237224	0.07364509	0.128647	0.48061131	0.67100844
Lonrf3	1166.47743	-1.1404797	-0.1896408	0.08738244	0.01730963	0.061287
Loxl1	2365.03731	1.01802624	0.02577475	0.06733244	0.68776632	0.82093614
Loxl2	31.2816996	-1.0137789	-0.0197431	0.19188037	0.82171773	0.90376956
Loxl3	459.26444	-1.2837481	-0.3603621	0.13482436	0.00168917	0.00903476
Loxl4	646.881562	-1.304717	-0.3837369	0.11676791	0.00021642	0.00160156
Lpar1	1016.46249	1.02849529	0.04053519	0.07409152	0.56080055	0.7337952
Lpar4	298.540964	-1.1996946	-0.2626672	0.15399593	0.02887701	0.09086576
Lpar6	293.739501	-1.0387723	-0.0548794	0.13386669	0.60065349	0.76301484
Lpcat1	125.195744	1.03986863	0.05640127	0.16499609	0.65326327	0.79911216
Lpcat3	1685.9905	1.00464134	0.00668054	0.07088954	0.92127954	0.95940094
Lpcat4	1611.60332	-1.063477	-0.0887888	0.08349171	0.24629039	0.43661498
Lpgat1	703.288911	1.00374393	0.00539126	0.086554	0.94709146	0.97385147
Lpin1	166.083471	1.46491789	0.5508198	0.21373907	0.00099148	0.00578506
Lpin2	1647.91457	1.18307288	0.24253895	0.06998867	0.00024977	0.00180157
Lpin3	30.9780108	-1.219325	-0.2860828	0.36776156	0.04432097	0.12715601
Lpl	5983.51	-1.6198385	-0.69585	0.0695523	1.51E-24	1.18E-22
Lpp	237.223021	1.14145294	0.19087138	0.1311685	0.07531986	0.18928143
Lpxn	48.1029849	-1.033172	-0.0470805	0.1807068	0.64668618	0.79463308
Lrba	3857.73324	1.19428546	0.25614771	0.05406202	9.68E-07	1.26E-05
Lrch1	612.328652	-1.0201565	-0.0287905	0.08594923	0.7142909	0.83876308
Lrch2	1.86119233	-1.019642	-0.0280627	0.2168435	0.25103639	0.44235866
Lrch3	2499.23009	1.072628	0.10114983	0.06125054	0.0843743	0.20595176
Lrfn3	112.369671	-1.0205709	-0.0293765	0.14729647	0.78410628	0.87922895
Lrfn4	1195.56694	1.02735222	0.03893088	0.09432726	0.64656278	0.79457992
Lrguk	21.9094063	1.03246519	0.04609314	0.20998039	0.50080644	0.6861316
Lrif1	1216.04463	-1.0841235	-0.1165291	0.09286405	0.16307166	0.33058205
Lrig1	1364.50696	-1.3029235	-0.3817524	0.07258832	3.40E-08	5.68E-07
Lrig2	489.697625	-1.0455363	-0.0642432	0.10701993	0.48567329	0.67463401
Lrig3	1234.85045	1.01811822	0.02590509	0.06492731	0.67612472	0.81302522
Lrit3	15.9178652	-1.102892	-0.1412915	0.26334816	0.09069076	0.21747447
Lrp1	3551.68016	1.11678054	0.1593457	0.05652329	0.00338903	0.01615085
Lrp10	3185.90793	1.17362267	0.23096864	0.07088504	0.00055006	0.00354087

Lrp12	175.308423	-1.0036128	-0.0052027	0.1366788	0.95935646	0.98098514
Lrp2	30.7859011	-1.4102599	-0.4959611	0.83581562	0.01423164	0.05229955
Lrp2bp	8.05955987	-1.0386794	-0.0547504	0.21711333	0.33784212	0.53741592
Lrp3	273.1357	1.27033453	0.34520847	0.1337776	0.0023473	0.01194157
Lrp4	1775.95481	1.2292401	0.29776673	0.06710909	3.26E-06	3.77E-05
Lrp5	3017.26882	1.08199082	0.11368826	0.05717266	0.03872284	0.11471775
Lrp6	5089.77922	1.04296081	0.06068495	0.04287394	0.15099834	0.31293005
Lrp8	5559.47744	-1.0730737	-0.1017491	0.06610689	0.10401865	0.24032874
Lrpap1	4765.64899	1.05630651	0.07902853	0.06885254	0.22495707	0.41362138
Lrr1	302.457099	-1.1815282	-0.240654	0.17101918	0.05547629	0.15043424
Lrrc1	612.943852	1.1146992	0.15665445	0.10839253	0.09559247	0.22564627
Lrrc14	135.510644	1.21778044	0.28425404	0.24111792	0.04963174	0.13833389
Lrrc15	2.22635573	-1.003628	-0.0052247	0.21383796	0.84285082	0.91553625
Lrrc20	575.384342	-1.0359176	-0.0509092	0.0946703	0.54796967	0.72492941
Lrrc24	2.05431623	-1.0033059	-0.0047615	0.21351778	0.86769949	0.92836933
Lrrc28	423.835964	1.13811597	0.18664757	0.10547994	0.04380384	0.1261527
Lrrc40	767.751952	1.0914508	0.1262471	0.08088733	0.09079538	0.21764387
Lrrc41	2608.18279	-1.0438053	-0.0618526	0.08251267	0.41963482	0.61830252
Lrrc42	947.253368	-1.1141893	-0.1559943	0.07284919	0.02231728	0.07499588
Lrrc45	960.220261	1.07858002	0.10913321	0.07785044	0.13162599	0.28352828
Lrrc46	71.9134744	1.12024339	0.16381222	0.22040825	0.17201774	0.34403547
Lrrc47	1296.89315	1.13003957	0.17637329	0.0631742	0.00339817	0.01618236
Lrrc49	1107.46742	1.22331673	0.29079798	0.07439352	3.43E-05	0.00031079
Lrrc4c	1.96160943	1.02177842	0.03108237	0.21848607	0.04987715	0.13874622
Lrrc51	67.7641385	-1.1381423	-0.1866809	0.23503896	0.13190754	0.2839618
Lrrc56	9.29197815	1.01827474	0.02612687	0.20921271	0.65109956	0.79768414
Lrrc57	921.550666	1.17145217	0.22829805	0.07682256	0.00147258	0.00802029
Lrrc58	1087.84269	-1.0564088	-0.0791683	0.08711182	0.31869541	0.51720318
Lrrc59	5884.19077	-1.2664371	-0.3407754	0.04443598	4.64E-15	1.88E-13
Lrrc61	6.05445167	1.02483957	0.03539808	0.21535791	0.41352375	0.61232681
Lrrc69	13.8596705	-1.0262251	-0.0373473	0.21335115	0.46760001	0.660123
Lrrc71	188.388987	1.81419465	0.85932925	0.19955379	1.18E-06	1.50E-05
Lrrc73	7.61615781	-1.0250374	-0.0356766	0.21256314	0.49542641	0.68188156
Lrrc75a	243.838188	1.53698735	0.6201053	0.1591129	9.85E-06	0.00010238
Lrrc8b	275.621124	-1.9137884	-0.9364313	0.12855966	2.28E-14	8.50E-13
Lrrc8c	2674.87504	-1.2452105	-0.3163896	0.05784194	1.47E-08	2.61E-07
Lrrc8d	1594.7572	-1.2190362	-0.285741	0.09034979	0.00055891	0.00358337
Lrrc8e	523.765743	1.0540272	0.07591209	0.09477855	0.37270387	0.57425266
Lrrcc1	493.409748	1.11834719	0.16136814	0.11182563	0.09412386	0.22328271
Lrrfip2	2366.44089	-1.0102859	-0.0147636	0.06209397	0.80372966	0.89156365
Lrriq4	8.27232494	1.01840991	0.02631836	0.21063243	0.62066864	0.777138
Lrrk1	1389.83506	1.2374067	0.30731975	0.07239031	7.39E-06	7.87E-05
Lrrk2	1895.52023	1.22287302	0.29027461	0.07844361	7.85E-05	0.00065882
Lrrn3	243.522908	1.15420889	0.20690435	0.14188126	0.06710954	0.17408885
Lrrn4	10.0833604	1.06891366	0.09614533	0.23890662	0.0968695	0.22789683
Lrrn4cl	9.62980531	-1.013977	-0.0200249	0.20990125	0.69655317	0.82661001
Lrsam1	322.15403	1.00880516	0.01264757	0.11306408	0.89692479	0.94603595
Lrwd1	363.996298	1.02076823	0.02965534	0.12312327	0.7826506	0.87837342
Lsg1	3761.77842	-1.0930961	-0.1284202	0.06517378	0.03814891	0.11338458
Lsm11	44.9429004	1.12574542	0.17088061	0.23288495	0.1532604	0.31618941

Lsm12	10365.31	1.01921521	0.02745871	0.05060656	0.57735417	0.74410617
Lsm14a	3111.73221	1.1892322	0.25003044	0.06253653	2.93E-05	0.00027035
Lsm14b	1639.93632	1.6222184	0.69796806	0.08834434	3.00E-16	1.35E-14
Lsm2	146.376131	-1.0078605	-0.011296	0.16451788	0.91441956	0.9555461
Lsm3	2877.71292	-1.0779451	-0.1082837	0.07317812	0.11433843	0.25678038
Lsm4	576.945363	-1.0841783	-0.116602	0.10298149	0.1976195	0.37834491
Lsm5	1314.72267	-2.0385025	-1.0275097	0.09320442	2.24E-29	2.28E-27
Lsm6	885.605586	-1.1411558	-0.1904958	0.11380383	0.05107222	0.14127298
Lsm8	1786.47454	-1.1369318	-0.1851457	0.06624083	0.00320534	0.01542465
Lss	673.64708	1.55294619	0.63500784	0.10562676	1.92E-10	4.56E-09
Lta4h	3459.04676	-1.2108747	-0.2760496	0.05376936	1.15E-07	1.76E-06
Ltbp1	13218.1116	1.14510257	0.19547683	0.05931775	0.00060571	0.003826
Ltbp2	200.191821	1.02436997	0.03473687	0.15947062	0.74933311	0.86089301
Ltbp3	1272.05497	2.67837301	1.4213569	0.12869422	1.27E-29	1.32E-27
Ltbr	658.481711	1.00036282	0.00052334	0.10361937	0.99880624	0.99959215
Ltn1	3046.60023	1.00296464	0.00427074	0.06163868	0.94399796	0.9727006
Ltv1	4165.07367	-1.0744922	-0.103655	0.0508433	0.03664795	0.1099181
Luc7l	1631.34844	-1.0957652	-0.1319387	0.07879924	0.0705193	0.18009415
Luc7l2	8463.5022	1.09082883	0.12542473	0.05622421	0.01423	0.05229955
Luc7l3	2740.36589	-1.0856689	-0.1185842	0.09309178	0.15714163	0.32228375
Lurap1l	130.929075	-1.5866354	-0.6659707	0.22711665	0.00025954	0.00185426
Luzp1	3041.79355	-1.0699375	-0.0975265	0.05400721	0.06159194	0.16336783
Lxn	887.968078	1.11267123	0.15402737	0.09479082	0.07043132	0.17994121
Ly6e	2791.62339	-1.0765081	-0.1063592	0.07985183	0.14972673	0.31106055
Ly6m	7.9066829	-1.1451269	-0.1955075	0.37344407	0.00126661	0.00706059
Ly75	76.1739911	-2.6705249	-1.4171233	0.26759223	6.34E-09	1.20E-07
Ly96	5.89566011	-1.0148846	-0.0213156	0.21334249	0.58104613	0.74725131
Lyar	4742.5639	-1.3035764	-0.3824751	0.08556624	1.82E-06	2.23E-05
Lypla2	632.492507	-1.1912398	-0.2524638	0.11101646	0.00909499	0.036171
Lyplal1	432.688141	-1.0199746	-0.0285332	0.09754775	0.74163625	0.85604256
Lyrm1	153.842814	1.38846876	0.47349472	0.1923182	0.00179905	0.00956194
Lyrm2	722.070866	-1.0661309	-0.0923845	0.08897523	0.25352435	0.44506605
Lyrm4	1000.04862	1.04186217	0.05916443	0.08112935	0.43137847	0.62946179
Lyrm7	309.27568	1.12367363	0.16822307	0.13638823	0.12381935	0.27208408
Lysmd1	285.313063	-1.1150865	-0.1571557	0.14433194	0.15932208	0.32560879
Lysmd2	615.815363	-1.1855395	-0.2455438	0.13406746	0.02592241	0.08383391
Lysmd3	1265.69613	-1.0275549	-0.0392154	0.07087485	0.55697248	0.73128402
Lysmd4	615.155377	1.07719128	0.10727445	0.09315383	0.20064364	0.38287577
Lyst	855.353358	-1.0153861	-0.0220285	0.08087727	0.76819416	0.8713089
Lzic	1140.55163	1.29839139	0.37672533	0.07665329	2.19E-07	3.18E-06
Lztfl1	332.585071	1.0717704	0.09999588	0.1217947	0.32278816	0.5220854
Lztr1	802.356038	1.11734777	0.16007829	0.09089061	0.05157821	0.14227324
Lzts2	1275.41918	-1.074724	-0.1039662	0.07757072	0.14903557	0.30990345
Lzts3	723.28466	1.22417155	0.29180574	0.10705143	0.00215328	0.01108215
M6pr	2482.4901	-1.0671597	-0.0937761	0.06495177	0.12879914	0.27951711
MAD1L1	1893.14113	1.20539844	0.2695101	0.06567504	1.69E-05	0.0001649
MBTPS1	5175.51978	-1.0453613	-0.0640017	0.05657638	0.2405525	0.43010648
MBTPS2	1191.48796	-1.0275148	-0.0391591	0.07042675	0.55556494	0.73050512
MED10	813.238645	-1.3061038	-0.3852696	0.08127347	4.61E-07	6.30E-06
MED11	256.887741	-1.0288415	-0.0410207	0.1296437	0.69166306	0.82348698

MED17	1253.54415	-1.0923818	-0.1274772	0.08424463	0.09857939	0.23067264
MED18	851.787615	-1.1124243	-0.1537072	0.10281767	0.0889092	0.2144064
MED19	780.805965	-1.1392114	-0.1880355	0.09833932	0.03191959	0.09850397
MED8	436.415618	1.1463578	0.1970574	0.1194289	0.05167654	0.14245244
METAP2	8713.46144	-1.2103371	-0.275409	0.05213642	6.03E-08	9.75E-07
MFT	474.547935	-1.0951354	-0.1311093	0.10022198	0.13887951	0.294837
MGAT5	1245.09273	1.06358259	0.08893207	0.0697607	0.17741385	0.35153463
MGME1	613.92654	-1.0603571	-0.0845502	0.10016206	0.34034055	0.53990395
MOCS2	1128.29226	-1.0323402	-0.0459184	0.06808217	0.47655343	0.66710189
MPPE1	152.50251	1.13767895	0.18609349	0.17387818	0.12908433	0.27970309
MRI1	186.743951	-1.0731563	-0.1018602	0.15707256	0.36533365	0.56630253
MT1	4.95568471	1.02619996	0.03731188	0.21472439	0.42836384	0.62608502
Mab21l3	19.3499485	-1.0734914	-0.1023107	0.23492736	0.16820918	0.3384262
Macf1	14512.9008	-1.0382494	-0.054153	0.04459382	0.21419418	0.4000915
Maco1	1483.01458	-1.1228584	-0.167176	0.07460924	0.01654817	0.05882622
Macrod1	327.131062	1.04290303	0.06060503	0.10914967	0.52017042	0.70296266
Macroh2a1	1541.00081	1.08750089	0.12101658	0.06748167	0.05849973	0.15680718
Mad2l1	1767.3034	-2.1058531	-1.0744048	0.06706426	6.26E-59	2.43E-56
Mad2l2	514.423295	-1.0009504	-0.0013706	0.09562412	0.98199846	0.99108517
Madd	1947.92336	1.34238268	0.42479601	0.07541997	3.51E-09	6.90E-08
Maea	3307.96193	-1.0239367	-0.0341266	0.05637093	0.52980706	0.71051387
Maf1	755.279904	1.00037369	0.00053902	0.09025394	0.99101518	0.99567878
Maff	77.7669563	-1.3334332	-0.4151455	0.25907628	0.01312898	0.04886178
Mafg	863.634051	1.12431049	0.1690405	0.10955948	0.07525844	0.18917986
Mafk	1107.44203	-1.1657166	-0.2212172	0.07105213	0.00094458	0.00555816
Maged1	5837.64551	1.22657247	0.29463247	0.04721586	1.67E-10	4.04E-09
Maged2	617.600203	1.71297471	0.77650385	0.1043368	9.14E-15	3.54E-13
Magee1	571.743708	1.30094792	0.37956321	0.11230648	0.00016251	0.00124505
Magee2	9.82954002	1.02515705	0.03584494	0.21036337	0.55017845	0.72687562
Mageh1	193.034252	1.02462932	0.03510207	0.12528698	0.73170933	0.84963711
Magi1	892.886901	-1.0895655	-0.123753	0.08334439	0.10613834	0.24370535
Magi2	260.380442	1.34124951	0.42357764	0.15160914	0.00088004	0.00525562
Magi3	1253.65456	-1.0434844	-0.061409	0.07781387	0.39622893	0.5967191
Magt1	2162.07712	-1.1789527	-0.2375058	0.06572664	0.0001212	0.0009649
Maip1	514.034777	-1.0410858	-0.0580889	0.10587465	0.52804855	0.70910718
Mak16	3841.14091	-1.0935784	-0.1290567	0.05295511	0.01173398	0.04484257
Malrd1	23.794134	-1.1983335	-0.2610295	0.47285709	0.01474634	0.05389462
Malsu1	282.444624	-1.8124919	-0.8579745	0.13532443	1.83E-11	5.04E-10
Malt1	303.003156	1.17583492	0.23368553	0.14050555	0.03886717	0.1150655
Mamdc2	2499.31977	1.36072332	0.44437375	0.07209235	1.39E-10	3.39E-09
Mamdc4	257.976876	1.04259068	0.06017287	0.16500968	0.58343755	0.74830245
Maml1	1021.34362	-1.2010479	-0.2642936	0.09003305	0.00131411	0.00728106
Maml3	474.279448	1.00879772	0.01263692	0.09834596	0.88596683	0.93872913
Mamld1	183.063991	-1.7906235	-0.840462	0.15831866	8.44E-09	1.56E-07
Man1a	2174.53757	1.01642373	0.02350196	0.05989061	0.69162734	0.82348698
Man1a2	4201.6921	1.07276655	0.10133615	0.0641246	0.09793	0.22946467
Man1b1	2391.79677	1.04552791	0.06423158	0.0526158	0.20908556	0.39446038
Man1c1	732.088016	1.6698204	0.73969294	0.08365658	9.11E-20	5.40E-18
Man2a1	5696.08151	-1.0393492	-0.0556805	0.04937681	0.24716999	0.43793189
Man2a2	819.768055	1.15036177	0.20208764	0.10201766	0.02460149	0.08078571

Man2b1	871.917321	1.2366421	0.30642803	0.09187384	0.00027951	0.00197055
Man2b2	1861.55952	1.10813823	0.14813785	0.06227526	0.0126343	0.04739251
Man2c1	3263.06236	1.19358434	0.25530051	0.06998496	0.00011442	0.00091896
Manba	9.5954988	-1.1098347	-0.1503448	0.28996443	0.01902048	0.06581641
Manbal	905.995966	1.13621791	0.18423956	0.09915595	0.03671101	0.11008148
Manea	1293.13973	1.17543355	0.23319298	0.07268913	0.00065389	0.00407833
Manf	4451.34543	1.0146365	0.02096296	0.12473114	0.83495099	0.91171934
Mansc1	2.86684966	-1.0285828	-0.0406579	0.21844095	0.23540426	0.42527641
Maoa	785.898679	1.26775177	0.34227228	0.09804723	0.00012855	0.00101522
Maob	64.4063928	-1.0032593	-0.0046945	0.17063453	0.96296037	0.98254201
Map10	171.558181	-1.0301336	-0.0428315	0.12770552	0.67821418	0.81477292
Map11	338.043226	1.06248171	0.087438	0.10620121	0.34479712	0.54414822
Map1a	6358.70582	-1.5063111	-0.5910198	0.06548956	2.28E-20	1.39E-18
Map1b	16607.8368	-1.9049978	-0.9297894	0.05884663	2.70E-57	9.61E-55
Map1lc3a	327.741592	1.4743525	0.5600815	0.15417658	3.24E-05	0.00029555
Map1s	1503.28231	-1.5217403	-0.6057222	0.09046253	2.43E-12	7.47E-11
Map2	19.5912604	1.03163671	0.04493502	0.20415111	0.56652604	0.73734368
Map2k2	1429.14353	1.03869404	0.05477075	0.08439413	0.48034459	0.67094255
Map2k3	1487.45877	-1.4307287	-0.5167501	0.07997179	1.47E-11	4.04E-10
Map2k4	414.0769	-1.03126	-0.0444081	0.10617796	0.6302388	0.78412578
Map2k5	1310.12845	1.08772617	0.12131542	0.06721234	0.05680643	0.15332708
Map3k1	1730.53755	-1.2721169	-0.3472313	0.08087629	4.78E-06	5.31E-05
Map3k10	655.08338	1.04772258	0.06725677	0.10062652	0.43571062	0.63332043
Map3k11	822.629279	-1.2049602	-0.2689855	0.0887091	0.00093958	0.00553382
Map3k12	305.836393	1.25747063	0.3305247	0.13236183	0.00324616	0.01560933
Map3k13	128.303779	-1.4217111	-0.5076283	0.25734943	0.00457248	0.02061719
Map3k14	724.804153	-1.3552608	-0.4385705	0.09277258	4.25E-07	5.83E-06
Map3k15	15.7185907	-1.0179042	-0.0256019	0.20330814	0.71862356	0.84156113
Map3k19	7.03869856	1.00370807	0.00533972	0.2105528	0.90821762	0.95230265
Map3k2	1250.9044	1.1217814	0.16579156	0.07459996	0.01755643	0.06193828
Map3k20	821.098472	1.00669116	0.00962116	0.07828431	0.89578465	0.94545616
Map3k21	1.97562539	-1.0241114	-0.0343726	0.21862782	0.12107199	0.26740209
Map3k3	1137.07501	-1.0024933	-0.0035926	0.0720831	0.95671293	0.97901352
Map3k4	3884.13991	-1.0204427	-0.0291952	0.04929052	0.54884587	0.72563893
Map3k6	720.191039	1.00356992	0.00514114	0.08437486	0.94690126	0.97373406
Map3k7	3589.56388	-1.0343143	-0.0486747	0.05887813	0.3893504	0.59099561
Map3k7cl	2.19613555	-1.0290863	-0.0413639	0.22052714	0.04507986	0.12898661
Map4	9206.00967	1.26845095	0.34306773	0.04588559	2.28E-14	8.50E-13
Map4k1	9.3460716	1.0208893	0.02982643	0.21136948	0.57208192	0.74079387
Map4k2	109.512851	-1.0035098	-0.0050548	0.14870952	0.96367701	0.982702
Map4k3	2012.20262	1.15544908	0.20845368	0.063954	0.0006257	0.0039271
Map4k4	23454.4591	1.05135191	0.07224565	0.03774615	0.05188688	0.14300148
Map6	1047.33996	-1.2249749	-0.2927522	0.08859128	0.00032614	0.00223895
Map7d1	4251.35121	-1.1017637	-0.1398149	0.06329826	0.02039441	0.0697417
Map7d2	12.6673491	-6.0630016	-2.6000322	0.88002203	0.00011689	0.00093644
Map9	144.105879	1.0850726	0.11779157	0.15518628	0.29908067	0.49689911
Mapk1	4070.71605	1.09508381	0.13104128	0.06943477	0.04545212	0.12962083
Mapk10	343.563109	-1.5070177	-0.5916963	0.13646318	1.60E-06	1.99E-05
Mapk14	2092.15906	-1.0929416	-0.1282164	0.06065123	0.02704005	0.08649645
Mapk1ip1	100.52692	1.47258529	0.55835119	0.28140394	0.00382283	0.01782038

Mapk1ip1l	783.079338	-1.0083407	-0.0119832	0.10848333	0.89927921	0.94766096
Mapk3	1756.70516	1.17266716	0.22979359	0.06166834	9.79E-05	0.00079693
Mapk7	311.443498	-1.0914438	-0.1262379	0.131287	0.23202989	0.42124475
Mapk8	713.113097	-1.0150334	-0.0215272	0.08450115	0.78123431	0.87751158
Mapk8ip1	453.369905	1.4753027	0.56101099	0.11164988	6.20E-08	9.97E-07
Mapk8ip3	597.71904	1.0070668	0.01015938	0.11299522	0.91689738	0.95692723
Mapk9	1128.18619	1.07811592	0.1085123	0.06973737	0.09942995	0.23200322
Mapkap1	2927.59404	1.15621333	0.20940761	0.06329143	0.00052494	0.00339618
Mapkapk2	3106.78931	1.02680151	0.03815732	0.06403592	0.5324162	0.71214157
Mapkapk3	641.380696	1.04872685	0.06863897	0.09651734	0.42651602	0.62469495
Mapkbp1	1335.63673	-1.147519	-0.198518	0.08069259	0.00785176	0.03196093
Mapre2	1800.12211	-1.0425614	-0.0601324	0.06500677	0.33099009	0.53103409
Mapre3	300.059661	1.02595291	0.03696451	0.11765279	0.7084827	0.83523499
Mapt	128.146743	-1.4681887	-0.5540374	0.20334106	0.00064631	0.00403471
Marchf2	1412.21703	-1.0315179	-0.0447688	0.06359309	0.46031307	0.65424127
Marchf3	190.876671	1.00511718	0.0073637	0.12236251	0.94262398	0.9719885
Marchf5	3993.06264	1.13835777	0.18695404	0.0482086	6.69E-05	0.00057042
Marchf6	1441.40035	-1.0333784	-0.0473686	0.0813655	0.52752358	0.70881471
Marchf7	2725.79841	-1.0561614	-0.0788303	0.06321252	0.18903255	0.36729838
Marchf9	78.4911457	1.21663108	0.28289176	0.24889738	0.05273864	0.14469522
Marcks	2001.53807	-1.9770797	-0.983371	0.06387132	1.36E-54	4.60E-52
Marf1	3451.59205	1.29597771	0.37404091	0.04872942	4.24E-15	1.72E-13
Mark1	2402.20988	1.03062712	0.04352246	0.05354695	0.40413621	0.60358923
Mark2	2617.30947	-1.0039253	-0.005652	0.06221596	0.92412381	0.96109284
Mark3	6465.77591	1.03625614	0.05138064	0.05569103	0.3397366	0.53919807
Mark4	776.386592	1.0834711	0.11566067	0.10667283	0.21246729	0.39831786
Mars1	2279.62502	1.39446515	0.47971188	0.08071902	4.66E-10	1.03E-08
Mars2	292.538207	-1.2777454	-0.3536004	0.1554126	0.00486769	0.02164356
Marveld1	7.90738106	1.01798838	0.02572109	0.20934833	0.65023579	0.79726959
Mast1	1.70813954	-1.0148316	-0.0212403	0.21616306	0.28988675	0.48630818
Mast2	6767.64286	1.27518825	0.35071024	0.0569884	2.07E-10	4.88E-09
Mast3	794.815591	1.07875594	0.1093685	0.09597083	0.20246138	0.38540324
Mast4	805.094017	-1.1166639	-0.159195	0.09753554	0.06719783	0.17420957
Mastl	2004.83819	1.02339845	0.03336796	0.06301028	0.57906282	0.74550701
Mat2a	4012.27875	-1.8558095	-0.8920486	0.04958551	1.82E-73	1.29E-70
Mat2b	599.055761	1.04394373	0.06204395	0.09787264	0.47680702	0.66723814
Matn4	2064.13707	1.02709558	0.03857044	0.07547746	0.58553382	0.74968416
Mau2	822.068595	1.06132152	0.08586177	0.09467311	0.3118056	0.51037947
Mavs	540.592819	-1.0141036	-0.0202051	0.10617516	0.8282709	0.90785918
Max	634.024182	-1.0377984	-0.0535262	0.10548541	0.56266115	0.73502849
Maz	2335.12794	1.10466038	0.1436029	0.08956898	0.07678966	0.19215162
Mbd1	272.458452	1.03302383	0.04687354	0.11623668	0.63298501	0.78571636
Mbd2	2452.05703	-1.0629159	-0.0880275	0.0792727	0.23101889	0.42009329
Mbd3	1304.12314	1.13786401	0.18632815	0.07309105	0.00656435	0.02751614
Mbd3l1	2.36482446	1.01122141	0.01609892	0.21453225	0.56124142	0.7339972
Mbd4	1430.19627	1.09588163	0.13209197	0.06037868	0.02232587	0.07500508
Mbd5	984.882502	-1.0048867	-0.0070328	0.08759102	0.92970748	0.96464869
Mbip	510.832632	1.05048332	0.07105325	0.09807162	0.41633232	0.61527654
Mblac1	53.0382992	1.06874508	0.09591777	0.20223923	0.35324975	0.55292391
Mblac2	1173.47009	1.06011934	0.08422668	0.07431001	0.22692152	0.41556322

Mbnl1	7647.21382	-1.5449992	-0.6276061	0.0512857	1.60E-35	2.05E-33
Mbnl2	2896.54308	-1.067728	-0.0945442	0.05821995	0.09046754	0.21702045
Mbnl3	1128.64794	1.00450146	0.00647966	0.07146239	0.92409109	0.96109284
Mboat1	776.522086	-1.1940209	-0.255828	0.10577371	0.00622183	0.02636509
Mboat7	397.526649	1.03486924	0.04944849	0.12392115	0.62696485	0.78218883
Mbp	224.928337	-1.0766897	-0.1066025	0.14210605	0.3295757	0.52945382
Mbtd1	442.84383	-1.0589045	-0.0825724	0.10601288	0.3700449	0.57146175
Mc1r	1.82473553	1.00714047	0.01026492	0.21481075	0.61363357	0.77269695
Mcat	341.471366	-1.0411573	-0.058188	0.11995312	0.55838817	0.73228424
Mcc	356.031281	-1.0003839	-0.0005538	0.1120997	0.99444209	0.99747891
Mccc1	938.019243	1.18675674	0.24702425	0.09935631	0.00548565	0.02383691
Mccc2	770.162093	1.17344879	0.23075489	0.09250605	0.00597196	0.02546633
Mcee	91.413116	1.08369451	0.11595812	0.1929485	0.30608669	0.50424004
Mcf2	2.20767529	1.01462437	0.02094571	0.21539316	0.40980945	0.60900917
Mcf2l	652.590314	1.127931	0.17367882	0.08960165	0.03270071	0.10055117
Mcfd2	1944.37689	-1.0742234	-0.103294	0.06492105	0.09387592	0.22285963
Mcl1	1408.29518	-1.1603861	-0.214605	0.11021673	0.02497773	0.08151968
Mcm10	2552.47128	1.04970239	0.06998036	0.05925866	0.21888773	0.40578234
Mcm2	11265.1162	-1.05084	-0.071543	0.0457901	0.10751228	0.24549596
Mcm3	6490.73175	-1.1925968	-0.2541064	0.04703944	3.01E-08	5.12E-07
Mcm3ap	4518.93617	-1.0194321	-0.0277657	0.0518988	0.58855615	0.75206028
Mcm4	10251.0505	-1.0755729	-0.1051053	0.04867779	0.02813595	0.08899298
Mcm5	6408.42624	1.00576959	0.00829984	0.04976693	0.86438207	0.92717978
Mcm6	7781.62981	-1.0128419	-0.018409	0.04195065	0.65372571	0.79952514
Mcm7	6843.15023	-1.0721359	-0.1004878	0.0469677	0.02759458	0.08771379
Mcm8	935.828998	1.22587555	0.29381253	0.08678803	0.00024624	0.00177912
Mcm9	738.926938	-1.0048905	-0.0070384	0.08763247	0.93215124	0.96640154
Mcmbp	2639.31095	1.07069445	0.09854683	0.05882331	0.0820089	0.201638
Mcoln1	388.77577	1.1700312	0.226547	0.11875075	0.0254186	0.08264271
Mcph1	1339.98774	-1.0199315	-0.0284722	0.06348349	0.63814655	0.78966937
Mcrip1	177.296998	1.53375539	0.61706842	0.16269413	1.49E-05	0.00014911
Mcrip2	287.17531	-1.0639426	-0.0894204	0.12703692	0.38539397	0.58657946
Mcrs1	1603.65729	-1.0448021	-0.0632297	0.08458257	0.41439528	0.61319205
Mctp1	161.428609	-1.5427919	-0.6255435	0.21483657	0.00030179	0.00209421
Mcts1	1122.7076	-1.0584	-0.0818849	0.0995272	0.35047139	0.55010693
Mcu	785.519446	-1.0245116	-0.0349364	0.08133869	0.64218945	0.79161344
Mcub	861.056936	1.0180916	0.02586737	0.07665576	0.71852872	0.84156113
Mcur1	1219.70624	-1.245969	-0.3172682	0.07801581	1.50E-05	0.00014994
Mdc1	3295.08275	1.19624764	0.25851607	0.07022574	9.95E-05	0.00080851
Mdfic	2392.10994	-1.0205978	-0.0294144	0.05533587	0.58309992	0.74830245
Mdh1	4206.13024	-1.0794273	-0.110266	0.05207686	0.02886332	0.09084501
Mdh1b	10.6283419	-1.9959642	-0.9970859	0.96945987	0.00623171	0.02639822
Mdh2	4175.06246	-1.0913593	-0.1261261	0.04913657	0.00839873	0.03387522
Mdm1	1873.63826	1.15044676	0.20219422	0.07305159	0.0031865	0.01535705
Mdm2	5953.60538	-1.1135618	-0.1551817	0.06102887	0.00770549	0.03143547
Mdm4	293.30029	1.04637589	0.06540121	0.11275356	0.49775563	0.68407355
Mdn1	5160.88044	-1.3105392	-0.3901605	0.07660751	8.14E-08	1.28E-06
Me1	3063.25528	-1.0737215	-0.1026198	0.06375429	0.0907788	0.21764387
Me2	1863.17115	-1.2021195	-0.2655804	0.07110333	7.70E-05	0.00064767
Me3	269.272381	1.63942624	0.71319099	0.15346755	2.98E-07	4.22E-06

Meaf6	1379.68086	-1.124295	-0.1690206	0.07532607	0.0162947	0.05807876
Meak7	238.518613	1.06390774	0.08937304	0.12842094	0.38975067	0.5914127
Mecp2	892.800067	-1.1570513	-0.2104528	0.09466068	0.01355027	0.05018183
Mecr	405.756621	-1.2074606	-0.2719761	0.10976392	0.00482031	0.02146918
Med1	11117.9732	1.15977774	0.21384835	0.05029773	1.18E-05	0.00012101
Med12	3190.97132	1.1449752	0.19531635	0.05722792	0.00038209	0.00257201
Med13	9284.69541	-1.0446006	-0.0629515	0.05087176	0.202897	0.38596831
Med13l	3538.90038	1.14014674	0.18921952	0.05763837	0.0006324	0.00395944
Med14	3068.18016	1.12224375	0.16638606	0.05240394	0.00097446	0.00570771
Med15	785.094767	-1.0022886	-0.003298	0.08416088	0.96905732	0.98529846
Med16	1517.96672	1.3194011	0.39988321	0.07928824	1.00E-07	1.55E-06
Med21	738.755829	1.06041706	0.08463179	0.08541799	0.27934302	0.47509082
Med22	431.984532	1.25404397	0.32658793	0.11681584	0.00143352	0.00784415
Med23	2312.42468	1.24369988	0.31463839	0.06997683	2.25E-06	2.71E-05
Med24	10195.0893	-1.0291394	-0.0414384	0.06434828	0.49839127	0.68452928
Med25	1046.48252	1.03142862	0.04464398	0.09668359	0.60392879	0.76556019
Med26	522.424282	-1.1310803	-0.1777014	0.11567299	0.07136859	0.18168354
Med27	553.885968	1.01935906	0.02766232	0.09981244	0.75528083	0.86392958
Med29	705.293256	1.00473449	0.0068143	0.11545783	0.94556343	0.97298317
Med30	377.821472	-1.1246857	-0.1695219	0.10719064	0.06945664	0.17848337
Med31	328.001872	-1.0564134	-0.0791745	0.12990488	0.44734685	0.64330021
Med4	1552.27531	-1.3371026	-0.4191102	0.06414526	1.35E-11	3.75E-10
Med6	1556.17767	1.04356429	0.06151949	0.06511914	0.32164559	0.52056602
Med7	295.066694	1.1002918	0.13788617	0.12526604	0.18210775	0.35817599
Med9	151.5912	1.01611598	0.02306509	0.16031475	0.83165518	0.90953575
Medag	2085.86953	2.38283913	1.25268156	0.07189238	3.11E-69	1.90E-66
Mef2a	4941.13584	-1.0291764	-0.0414903	0.05415652	0.42764084	0.62570864
Mef2b	3.03182998	-1.0168014	-0.0240379	0.21472113	0.47244368	0.66388806
Mef2c	521.878434	-1.1049784	-0.1440182	0.1107804	0.1314162	0.28340766
Mef2d	882.425391	-1.0299797	-0.0426159	0.08271852	0.57586696	0.74338456
Mefv	14.6120557	1.00996491	0.01430517	0.20663071	0.81599206	0.90029863
Megf11	24.3024246	-1.9161317	-0.9381967	0.66294555	0.00437436	0.01984963
Megf6	4.66363218	1.02216194	0.03162378	0.21591979	0.37599124	0.57771954
Megf8	1097.5302	1.04028663	0.05698108	0.07378203	0.41095842	0.61007965
Megf9	1436.38592	1.0810427	0.11242351	0.07541196	0.11056642	0.2509934
Meis1	619.276674	1.14398708	0.19407076	0.08387401	0.01193052	0.0453636
Meis2	816.305048	1.03477804	0.04932135	0.09492402	0.56336451	0.7353474
Meis3	141.919492	-1.044469	-0.0627697	0.14670136	0.56263316	0.73502849
Melk	1788.12883	-1.0748827	-0.1041792	0.08232775	0.16947097	0.34052128
Memo1	1498.35504	1.01833136	0.02620708	0.06419376	0.66942693	0.80937792
Men1	786.325751	1.01839666	0.02629959	0.08271215	0.72710703	0.84644139
Mep1a	10.1671452	1.14891603	0.20027336	0.35455971	0.01620256	0.05792767
Mepce	1433.272	-1.1005826	-0.1382674	0.06782896	0.03103242	0.0963464
Mertk	372.036356	1.87736877	0.90871207	0.12633763	4.33E-14	1.58E-12
Mesd	4247.32982	1.04103148	0.0580137	0.05197576	0.24999586	0.44137625
Met	10360.6699	1.41079019	0.49650345	0.05689691	4.47E-19	2.56E-17
Metap1	706.544937	-1.1241295	-0.1688082	0.08970227	0.03780029	0.11263589
Metap1d	397.277924	1.12161203	0.16557373	0.10561396	0.07297905	0.18471875
Metazoa_SRP	315632.899	-1.0728049	-0.1013878	0.14404507	0.41081628	0.60993933
Metrn	71.6487775	1.28662622	0.36359299	0.27385502	0.02468491	0.08097662

Metrnl	100.905406	1.00497195	0.00715524	0.1538332	0.94828212	0.97437186
Mettl1	508.12683	-1.0369445	-0.0523387	0.09552409	0.53981462	0.71842966
Mettl14	1512.16919	-1.0912087	-0.125927	0.06763848	0.04916695	0.13739718
Mettl15	146.550922	1.0018923	0.00272743	0.14083288	0.97820352	0.9900158
Mettl16	2128.22112	-1.1376488	-0.1860552	0.07698579	0.00944661	0.03730306
Mettl17	401.942864	-1.0920251	-0.1270061	0.12998665	0.22590437	0.41443281
Mettl18	257.704861	-1.1258996	-0.1710782	0.12661312	0.10128851	0.23528845
Mettl2	678.738622	-1.0617688	-0.0864697	0.08792966	0.2804598	0.47616834
Mettl21a	371.047308	-1.179314	-0.2379478	0.14070711	0.03580049	0.10785612
Mettl21e	3.10450321	-1.0246894	-0.0351867	0.21764369	0.24027963	0.42993254
Mettl22	31.1051567	-878.84367	-9.7794627	2.90561636	3.98E-15	1.62E-13
Mettl23	134.841322	1.04356011	0.0615137	0.1444804	0.57077517	0.73973875
Mettl26	198.967619	1.10789005	0.14781471	0.15461511	0.19738504	0.37806555
Mettl3	851.269365	-1.0454395	-0.0641096	0.07831125	0.3784713	0.5804846
Mettl4	662.76935	1.01877133	0.02683026	0.09191717	0.74753429	0.8599061
Mettl5	507.954376	1.13169871	0.17848992	0.12005939	0.07816217	0.19483475
Mettl6	1030.27476	-1.0839566	-0.1163071	0.0723218	0.08678872	0.2105214
Mettl8	387.584889	1.07881725	0.1094505	0.13579608	0.30757358	0.50590877
Mettl9	1188.41764	-1.0277053	-0.0394266	0.09685376	0.64757728	0.79527034
Mex3a	162.788084	1.01514357	0.02168379	0.14944946	0.84193604	0.91524106
Mex3b	203.100036	1.18603333	0.24614456	0.17503407	0.05317226	0.14572886
Mex3c	1756.44553	-1.1669418	-0.2227326	0.07113272	0.0008842	0.00527064
Mex3d	433.684174	1.06708244	0.09367163	0.10679309	0.31283306	0.51180965
Mfap3	156.205126	1.01228523	0.01761586	0.14834209	0.87033191	0.92970236
Mfge8	2568.8916	-1.0172712	-0.0247044	0.0624198	0.68107672	0.81646968
Mfn2	5506.01668	-1.0343773	-0.0487625	0.04767323	0.29467492	0.49208522
Mfng	1.86313293	-1.0158624	-0.022705	0.21705066	0.08829322	0.21340128
Mfsd1	3638.93423	1.09392533	0.12951426	0.0482654	0.00576238	0.02477048
Mfsd11	592.863384	-1.0679291	-0.0948159	0.11743796	0.33586259	0.53559066
Mfsd12	138.827181	1.03431064	0.04866954	0.1565888	0.65583804	0.8004869
Mfsd14a	2059.50612	-1.0629273	-0.0880429	0.05447142	0.0942261	0.2234425
Mfsd14b	643.359029	1.02626871	0.03740852	0.10301854	0.66310715	0.80515664
Mfsd2a	46.6856496	-1.1366753	-0.1848203	0.25572574	0.12034539	0.26628282
Mfsd3	71.7478296	1.0824348	0.11428013	0.2067955	0.28974207	0.48619282
Mfsd4b4	523.89625	1.41367288	0.49944833	0.10079391	1.11E-07	1.70E-06
Mfsd5	775.015103	1.11637085	0.15881636	0.1086061	0.09140261	0.21853685
Mfsd6	767.401849	1.0213746	0.03051209	0.084177	0.69032834	0.82279048
Mfsd8	222.232535	1.01845052	0.0263759	0.12446023	0.79422064	0.88567075
Mfsd9	590.509961	1.07515782	0.10454845	0.08419171	0.17364891	0.34659434
Mga	3271.94754	-1.0598312	-0.0838345	0.06258624	0.16062432	0.32691413
Mgarp	140.115823	1.04215706	0.05957272	0.13851626	0.57820953	0.74463297
Mgat1	677.23281	1.03952223	0.05592061	0.08318911	0.46517001	0.65833506
Mgat2	1252.13846	-1.0197711	-0.0282454	0.07584558	0.69003378	0.8227227
Mgat4a	3.01956819	-1.0629661	-0.0880955	0.24095973	0.01237788	0.04666291
Mgat4b	561.981037	1.13637492	0.18443889	0.12034632	0.0686095	0.17691928
Mgrn1	1221.07147	1.15210866	0.20427679	0.06710963	0.00131879	0.00729751
Mgst1	2876.66368	-1.3406174	-0.4228976	0.07663365	6.90E-09	1.29E-07
Mgst3	793.399157	-1.0549992	-0.0772419	0.08444041	0.3187184	0.51720318
Mia2	2190.91086	1.02841226	0.04041871	0.06313375	0.50281988	0.68781798
Mib1	2923.90136	1.01930307	0.02758308	0.06090301	0.63765194	0.7892098

Mib2	527.417704	1.1873145	0.24770213	0.11305738	0.01165118	0.04456603
Mical1	1979.78781	1.0510682	0.07185628	0.09206597	0.38831753	0.58993685
Mical2	1160.88188	1.43741363	0.52347527	0.08656423	2.07E-10	4.88E-09
Mical3	8401.18137	1.18129757	0.24037243	0.05037869	9.03E-07	1.18E-05
Micall1	1191.4298	1.26958535	0.34435738	0.0815106	6.68E-06	7.14E-05
Micall2	873.364125	1.23020198	0.29889521	0.07957284	6.00E-05	0.00051673
Micos10	2479.29614	1.04008496	0.05670138	0.05666765	0.29965952	0.49755521
Micos13	568.602965	1.11946776	0.16281298	0.11443691	0.09603451	0.22634813
Micu1	2898.17811	1.04716168	0.06648421	0.05968367	0.24562656	0.43588591
Micu2	1648.32042	1.13943753	0.18832183	0.06925355	0.0039801	0.01841945
Micu3	595.299405	-1.0433168	-0.0611773	0.0895524	0.45175087	0.64694563
Mid1	1246.26107	1.27766158	0.35350575	0.07121815	1.91E-07	2.81E-06
Mid1ip1	326.109228	-1.2689609	-0.3436477	0.13233504	0.00226249	0.01156522
Mid2	1079.66669	1.0509499	0.0716939	0.06928075	0.27386944	0.46939624
Midn	1027.60929	1.19706796	0.25950505	0.08907993	0.00146093	0.00796024
Mief1	666.859143	-1.1328045	-0.1798989	0.10130739	0.04451245	0.12756251
Mief2	116.841135	-1.0587489	-0.0823605	0.18053644	0.44818476	0.64385407
Mien1	684.590683	1.40958245	0.49526787	0.12957131	1.88E-05	0.00018153
Mier1	3494.26928	1.01498302	0.02145559	0.0547531	0.68576014	0.81957283
Mier2	280.905603	1.04872442	0.06863562	0.12351734	0.50043773	0.68583839
Mier3	1348.83035	1.00723196	0.01039596	0.07545559	0.88373727	0.93752998
Mif	1365.64281	-1.1829222	-0.2423552	0.09701047	0.00533237	0.02328936
Mif4gd	73.5868492	-1.0466766	-0.0658157	0.16814046	0.54607903	0.72332461
Miga2	532.330076	-1.0418871	-0.059199	0.09234355	0.47644663	0.66709819
Miip	1188.86709	-1.1477389	-0.1987945	0.08392116	0.01003135	0.03935729
Minar1	675.128544	-3.7571872	-1.909653	0.10905142	7.32E-70	4.69E-67
Mindy1	1329.98897	1.18283808	0.2422526	0.06785347	0.0001706	0.00130315
Mindy2	412.759018	1.11318546	0.15469397	0.1109326	0.10527594	0.24207229
Mindy3	673.810496	-1.1266181	-0.1719986	0.08137899	0.02204412	0.07425327
Mindy4	281.59669	-1.752545	-0.8094514	0.15154185	6.96E-09	1.30E-07
Mink1	1195.41846	1.19530964	0.25738439	0.08301886	0.00081386	0.00491352
Minpp1	2374.6045	1.01023655	0.01469314	0.07052942	0.82754006	0.90760173
Mios	1028.96683	1.0563944	0.07914856	0.07862091	0.27893874	0.47465532
Mipep	714.378809	1.19283013	0.25438861	0.10013687	0.00452005	0.02042394
Mipol1	1092.09653	1.22313889	0.29058824	0.08483064	0.00021954	0.00162186
Mis12	130.42184	1.01443458	0.02067583	0.15116019	0.84964103	0.91917082
Mis18a	532.407826	-1.0348069	-0.0493616	0.10871039	0.59937567	0.76216025
Mitd1	569.785382	-1.0180935	-0.0258701	0.09582456	0.7619828	0.86841635
Mki67	36364.1864	-1.1178148	-0.1606812	0.06047801	0.00540973	0.02357098
Mkks	1656.05864	1.00174049	0.00250881	0.07827201	0.97331774	0.98781495
Mkln1	1244.29189	1.05640171	0.07915854	0.07879402	0.27970831	0.47545959
Mknk2	1269.97913	1.14974109	0.20130902	0.08371712	0.00895708	0.03572446
Mkrn1	1099.6594	1.06866041	0.09580348	0.07900851	0.19175159	0.37051699
Mkrn3	101.643748	1.4471202	0.53318476	0.25464856	0.00327527	0.01571396
Mks1	162.870971	1.12408558	0.16875187	0.16965253	0.16011056	0.32623132
Mlc1	1.69953022	1.01256193	0.01801015	0.21573981	0.35957196	0.55983395
Mlec	1974.72385	1.02856707	0.04063587	0.06621598	0.51944873	0.70228372
Mlf2	2180.14191	1.12565887	0.17076969	0.06779173	0.00776905	0.03167459
Mlh1	750.96248	-1.0315021	-0.0447467	0.08712584	0.57344472	0.74177793
Mlh3	391.788478	-1.0018157	-0.0026171	0.1041873	0.97622785	0.98895979

Mlkl	1209.56711	-1.1156505	-0.1578851	0.07098471	0.01810791	0.06344689
Mllt1	3336.47548	-1.1206904	-0.1643878	0.04788503	0.00040845	0.00272087
Mllt10	1018.22403	1.09386749	0.12943798	0.08267963	0.08951593	0.215383
Mllt11	127.804793	-1.1158247	-0.1581104	0.20767751	0.18932026	0.36762052
Mllt3	991.931133	-1.015832	-0.0226618	0.07073906	0.7339132	0.85127021
Mllt6	1685.08862	1.0397892	0.05629108	0.07963444	0.44655142	0.64292631
Mlph	184.738685	1.45175343	0.53779645	0.31145811	0.00650217	0.02729123
Mlst8	300.047621	1.12060298	0.16427523	0.14581658	0.1457798	0.30538662
Mlx	855.221959	-1.0639152	-0.0893832	0.09061572	0.27289111	0.46866002
Mlxip	1234.14021	1.03287289	0.04666272	0.07185792	0.49118414	0.67831704
Mlxipl	2.49850702	-1.0081048	-0.0116456	0.21418823	0.66481571	0.80593254
Mlycd	192.315122	1.04121248	0.05826451	0.14158714	0.58956907	0.7527539
Mmaa	200.357989	1.57252274	0.65308088	0.20768231	0.00014817	0.00114895
Mmab	1195.28615	1.11101678	0.15188061	0.07367335	0.02781252	0.08822825
Mmachc	518.545743	1.02828388	0.0402386	0.10849224	0.6680127	0.80811765
Mmadhc	1701.77296	-1.0138586	-0.0198565	0.07983641	0.78716975	0.88066764
Mmd	551.265597	-1.3855528	-0.4704617	0.11562922	7.31E-06	7.80E-05
Mmgt1	279.257497	-1.2119515	-0.2773319	0.17350778	0.03145502	0.09744589
Mmgt2	46.7590077	1.03093779	0.04395728	0.18111634	0.66885035	0.80883347
Mmp11	167.70793	1.19581314	0.25799197	0.23331846	0.06608524	0.17223844
Mmp12	173.999267	-10.112502	-3.3380681	0.20666543	6.08E-60	2.43E-57
Mmp14	4214.45118	-1.0030843	-0.0044429	0.057466	0.93541718	0.96876821
Mmp15	2.12718751	-1.0236786	-0.0337628	0.21837997	0.13085313	0.2824787
Mmp17	58.3486737	1.83029351	0.87207502	0.34719492	0.00062374	0.00391863
Mmp19	401.304549	1.09804705	0.13493987	0.13464389	0.21272327	0.39862275
Mmp21	5.50474153	-1.0366275	-0.0518975	0.21874957	0.2852005	0.48187146
Mmp23	165.826223	1.08309976	0.11516613	0.17110673	0.31807841	0.51675136
Mmp24	8.99245736	-1.1537876	-0.2063776	0.3937029	0.00191478	0.01005258
Mmp28	286.119479	-1.0741146	-0.103148	0.11707955	0.29479537	0.49218013
Mmp3	28.0903148	-770.99564	-9.5905789	2.88633227	2.41E-14	8.96E-13
Mmp9	1534.6035	-3.7605252	-1.9109342	0.09218346	7.21E-97	8.40E-94
Mmrn2	2.12540132	1.03182162	0.04519358	0.22202046	0.01908476	0.06597355
Mms19	855.298088	1.03984457	0.0563679	0.10142942	0.52949521	0.71024436
Mms22l	1618.41163	1.01275406	0.01828386	0.08107006	0.80854212	0.89527397
Mmut	2258.72663	1.32463254	0.4055922	0.06973902	1.33E-09	2.77E-08
Mnat1	1929.16564	1.04974756	0.07004243	0.07973573	0.34412716	0.54342565
Mns1	945.19796	-1.0699594	-0.097556	0.09212662	0.23999296	0.42967293
Mnt	508.612036	-1.0897718	-0.1240261	0.10550235	0.17714091	0.35130443
Moap1	439.33541	1.13963627	0.18857344	0.11106772	0.0495233	0.13818199
Mob1a	1165.69484	-1.8740712	-0.9061757	0.07326863	2.86E-36	3.73E-34
Mob1b	618.489306	-1.118331	-0.1613473	0.09266228	0.05251771	0.14428053
Mob2	463.605977	1.00264765	0.0038147	0.09322107	0.96463096	0.98289235
Mob3a	214.394655	1.01526822	0.02186092	0.12929728	0.83350638	0.91080514
Mob3b	760.136878	-1.2326215	-0.3017298	0.08498034	0.00012913	0.00101857
Mob3c	132.41442	1.18052769	0.23943188	0.19289327	0.06799949	0.17579687
Mob4	2032.80149	-1.0662316	-0.0925209	0.06065235	0.11080481	0.2512674
Mocs1	195.404478	-1.1642359	-0.2193835	0.20184096	0.0926953	0.22072724
Mocs3	85.2130203	1.00165985	0.00239267	0.17974619	0.98218372	0.99108517
Mogat1	6.28597598	1.01666856	0.02384942	0.21101584	0.63425015	0.78670941
Mogs	649.765673	-2.357361	-1.2371727	0.08924554	6.19E-45	1.37E-42

Mok	58.9062414	1.02732466	0.03889218	0.17727807	0.70757746	0.83463503
Mon1a	438.370873	1.01933021	0.02762148	0.11989599	0.78172729	0.87785651
Mon1b	1930.75892	-1.0181562	-0.0259589	0.06981384	0.69381139	0.82486113
Mon2	2487.44558	1.05510268	0.07738341	0.05686186	0.15769634	0.32316271
Morc1	13.119377	-1.0443955	-0.0626681	0.21782012	0.32444668	0.52384194
Morc2a	3065.0389	-1.0887525	-0.122676	0.05686548	0.02491066	0.0813952
Morc2b	5.26686839	-1.1127924	-0.1541845	0.30383184	0.00256116	0.01287614
Morc3	1694.90275	-1.0753995	-0.1048727	0.07252823	0.12415191	0.27246633
Morc4	4113.56146	1.06872131	0.09588569	0.05454593	0.06900853	0.17762124
Morn2	61.0007383	1.05937646	0.08321535	0.21023152	0.36429737	0.56524338
Morn4	77.63232	1.04690453	0.06612989	0.17091665	0.54648782	0.72332988
Morn5	183.889621	1.66255618	0.73340309	0.20869365	3.42E-05	0.00030971
Mosmo	136.585189	-1.0227932	-0.0325145	0.14401512	0.76151514	0.86796057
Mospd2	650.498417	1.06111318	0.08557854	0.09516785	0.31517197	0.51423226
Mospd3	944.872355	-1.1030939	-0.1415556	0.08505929	0.06901022	0.17762124
Mov10	921.416624	1.21771945	0.28418179	0.08713246	0.00040429	0.00270096
Moxd1	10.2720518	-10.510685	-3.3937848	1.10919012	0.00010387	0.00084005
Mpdz	5578.15596	1.26749494	0.34197999	0.0471237	1.20E-13	4.24E-12
Mpg	238.853456	1.25559156	0.32836724	0.13814059	0.0045322	0.0204716
Mphosph10	4706.28473	-1.0255512	-0.0363995	0.05660046	0.50358582	0.68831993
Mphosph8	3029.41115	-1.0699344	-0.0975224	0.06155104	0.09757709	0.2289728
Mphosph9	2119.98863	1.03569555	0.05059997	0.05613184	0.35060645	0.55019828
Mpi	1006.46343	1.09806604	0.13496482	0.10429229	0.1396076	0.29594535
Mpnd	370.378446	1.2865269	0.36348162	0.12448367	0.00078969	0.00478744
Mpp1	1006.64879	1.07945409	0.11030189	0.08561319	0.15965256	0.32567047
Mpp5	1381.03237	1.09112069	0.12581069	0.07960178	0.08816778	0.21314007
Mpp6	3875.48313	-1.965122	-0.9746188	0.05657511	1.33E-67	7.43E-65
Mpp7	17.2528792	-7.6822882	-2.9415361	0.70226107	1.79E-06	2.20E-05
Mpst	689.920361	-1.1041707	-0.1429632	0.10880368	0.12803762	0.27827653
Mpv17	206.196425	1.02397232	0.03417671	0.13225897	0.74487065	0.85815209
Mpv17l	538.832179	1.1646282	0.21986945	0.11795968	0.02873137	0.09049638
Mpv17l2	372.525194	1.01327433	0.01902481	0.11727958	0.84726926	0.91784517
Mpzl1	361.20331	1.22725685	0.29543722	0.12307568	0.00517837	0.022733
Mpzl3	18.9323948	1.00264863	0.00381611	0.20200552	0.95886377	0.98060394
Mras	764.100723	1.45746976	0.54346595	0.11142337	1.12E-07	1.72E-06
Mre11a	1587.84112	1.17438628	0.23190702	0.08407012	0.00278816	0.01377945
Mrfap1	1274.37749	-1.0836019	-0.1158348	0.11716618	0.24049067	0.43010648
Mrgbp	542.927211	1.45530995	0.54132645	0.12224646	1.22E-06	1.55E-05
Mrm1	131.988377	-1.0133049	-0.0190683	0.15285226	0.85840628	0.92418976
Mrm2	423.694018	-1.0357623	-0.0506929	0.10778924	0.58684283	0.75046987
Mrm3	574.793554	-1.0847343	-0.1173417	0.0976844	0.17682714	0.35096927
Mrnip	273.974257	-1.0539805	-0.0758482	0.11368015	0.4332925	0.63123813
Mro	13.6121251	-18.475803	-4.2075651	0.99666177	1.33E-06	1.67E-05
Mroh1	356.998957	1.28701467	0.3640285	0.13612362	0.00163789	0.00880092
Mroh2b	13.2899375	-1.0743415	-0.1034527	0.23730054	0.14840009	0.30891973
Mroh6	6.28894194	-1.0460268	-0.0649199	0.22611017	0.11491361	0.25790472
Mroh8	54.8330953	1.0964224	0.13280371	0.2097204	0.24057087	0.43010648
Mrpl10	1611.5656	1.07512426	0.10450342	0.07620273	0.14148765	0.29903594
Mrpl11	817.949282	1.06742227	0.09413102	0.08186751	0.21132044	0.39685805
Mrpl12	4988.3352	1.0333336	0.04730608	0.05921189	0.40616907	0.60567396

Mrpl13	6746.14471	-1.1343158	-0.1818223	0.05111063	0.00024318	0.00175899
Mrpl14	862.819212	1.03045394	0.04328002	0.10036158	0.62741486	0.78236001
Mrpl16	963.018728	-1.03806	-0.0538899	0.07947525	0.46399232	0.65728516
Mrpl18	867.404584	-1.0005386	-0.0007768	0.08536953	0.99803531	0.99951782
Mrpl19	735.586427	-2.1578357	-1.109585	0.1060495	9.43E-27	8.27E-25
Mrpl2	923.782936	-1.0493669	-0.0695192	0.08771545	0.38489693	0.58600502
Mrpl20	1618.45794	1.11085456	0.15166994	0.07192289	0.02493112	0.08142978
Mrpl21	1792.53906	-1.0656349	-0.0917132	0.07325329	0.18183957	0.35775839
Mrpl22	603.958629	-1.0361091	-0.051176	0.09922995	0.56292	0.73507789
Mrpl23	595.539265	1.10290258	0.14130537	0.10694965	0.12882662	0.27951711
Mrpl27	279.27359	1.04565775	0.06441072	0.1358854	0.54484341	0.722411
Mrpl28	1574.66234	1.05848169	0.08199632	0.07233307	0.2277192	0.4163616
Mrpl3	2927.29241	-1.0095134	-0.0136601	0.05295163	0.78977332	0.8825784
Mrpl30	1876.36501	1.12880674	0.17479851	0.06023405	0.00244592	0.01238917
Mrpl32	971.583144	-1.0422614	-0.0597171	0.08600929	0.44814203	0.64385407
Mrpl34	260.397742	1.01667717	0.02386165	0.13840329	0.82330675	0.90419748
Mrpl35	625.233307	-1.9929573	-0.9949108	0.13620113	1.86E-14	7.00E-13
Mrpl36	38.5024488	1.01188623	0.01704709	0.19114201	0.84985007	0.91931932
Mrpl37	2176.41657	-1.0287948	-0.0409553	0.07124647	0.54152908	0.71951894
Mrpl38	574.745182	1.05528643	0.07763463	0.11293894	0.42048088	0.61905069
Mrpl39	1790.26475	-1.0285999	-0.0406819	0.07273203	0.55170329	0.7277643
Mrpl4	531.587545	-1.0656053	-0.0916732	0.1221196	0.36326186	0.56391848
Mrpl40	1322.71628	1.21703911	0.28337553	0.07281935	3.81E-05	0.00034185
Mrpl41	615.663475	1.27710883	0.35288147	0.09585115	5.97E-05	0.00051436
Mrpl43	631.015852	-1.085724	-0.1186574	0.0886468	0.14080558	0.29798768
Mrpl44	927.846046	-1.0342691	-0.0486117	0.07747009	0.5001375	0.68576981
Mrpl45	3764.81793	1.1146215	0.15655389	0.0525145	0.00204992	0.01063135
Mrpl46	1338.45678	1.00400671	0.00576891	0.07671032	0.93661348	0.96922329
Mrpl47	1079.88424	-1.103928	-0.142646	0.09705655	0.09944991	0.23200752
Mrpl48	982.469642	1.02677494	0.03811999	0.08380899	0.62201322	0.77824473
Mrpl49	363.845541	-1.0891722	-0.123232	0.12499639	0.22948918	0.41806073
Mrpl50	377.308196	-1.0949327	-0.1308422	0.12392354	0.2017016	0.38425286
Mrpl52	395.553967	1.07002321	0.0976421	0.11622729	0.31750999	0.51628623
Mrpl53	141.457541	-2.1010428	-1.0711056	0.21664919	4.80E-08	7.89E-07
Mrpl54	873.495677	1.03874911	0.05484725	0.07517766	0.43609147	0.63377706
Mrpl55	482.09563	-1.0537908	-0.0755884	0.10945199	0.42150778	0.6199925
Mrpl57	242.502648	1.15456978	0.20735537	0.13981948	0.06371464	0.16762877
Mrpl58	792.130196	1.18879299	0.24949751	0.11859696	0.01410279	0.05194271
Mrpl9	1801.60846	1.00820392	0.01178747	0.0739564	0.86592818	0.92774752
Mrps10	1701.9272	1.03701825	0.05244129	0.07072765	0.43386896	0.63171872
Mrps11	853.764864	1.06139108	0.08595633	0.09731439	0.32245764	0.52174844
Mrps12	184.321567	-1.1388637	-0.1875951	0.17351502	0.12499653	0.27385079
Mrps14	1161.35237	1.10894236	0.14918439	0.09739192	0.0863984	0.20968695
Mrps15	1104.62544	1.0074418	0.0106965	0.07335282	0.87743181	0.93416734
Mrps16	228.252226	-1.0738276	-0.1027624	0.14877656	0.35435035	0.55403734
Mrps17	1026.93942	1.03311132	0.04699572	0.07791585	0.51769507	0.70080037
Mrps18a	1285.06221	-1.0252718	-0.0360065	0.07382567	0.60276699	0.76472667
Mrps18b	1635.70884	-1.0393725	-0.0557128	0.0685167	0.38962774	0.59129622
Mrps18c	556.917915	1.00689788	0.00991738	0.1234861	0.92319023	0.96092198
Mrps2	1055.06549	1.13034078	0.17675779	0.07929934	0.01617444	0.05785942

Mrps22	1833.88969	-1.0831704	-0.1152602	0.0800848	0.11971103	0.26521935
Mrps23	1898.41221	-1.0595982	-0.0835173	0.0613392	0.15445326	0.31799201
Mrps24	617.357709	1.70217389	0.76737842	0.09814768	5.12E-16	2.25E-14
Mrps25	1391.42882	1.10538701	0.14455157	0.0837126	0.06050979	0.16104933
Mrps26	813.921805	1.01557898	0.02230243	0.08474853	0.77026294	0.87232885
Mrps27	1754.13017	1.01010364	0.01450333	0.07970557	0.84570367	0.91684699
Mrps28	255.312976	1.08553762	0.11840972	0.13905813	0.27104098	0.46635795
Mrps30	1760.35553	-1.1036567	-0.1422914	0.07583513	0.04435588	0.12722767
Mrps31	899.939377	-1.0635892	-0.088941	0.07764044	0.21790291	0.40442426
Mrps33	1937.57129	1.00616041	0.00886033	0.06191229	0.88127398	0.93654552
Mrps34	782.224853	-1.074867	-0.1041581	0.09771742	0.22882866	0.41748237
Mrps35	2802.6913	-1.1844925	-0.244269	0.07420986	0.00045515	0.00299457
Mrps36	1487.70904	1.0352465	0.04997432	0.07819168	0.49289916	0.67965966
Mrps5	2676.56875	-1.0227422	-0.0324425	0.06005174	0.57642591	0.74377612
Mrps6	302.361686	1.19058159	0.25166649	0.16081728	0.0400843	0.11777063
Mrps7	1978.92319	-1.0221774	-0.0316456	0.0748971	0.65134945	0.79782837
Mrps9	1968.21261	1.01781207	0.0254712	0.05791022	0.64890706	0.79621642
Mrrf	475.444165	-1.0241487	-0.0344252	0.11652175	0.72435811	0.84454605
Mrs2	800.700868	1.05907355	0.08280278	0.07616301	0.24450685	0.43483726
Mrtfa	1046.25432	1.08186029	0.11351421	0.07161652	0.09178754	0.21917026
Mrtfb	1211.27609	1.09924326	0.13651068	0.0893169	0.09280693	0.22089498
Mrto4	2140.41348	-1.1715977	-0.2284773	0.08275139	0.00273504	0.01358341
Mrvi1	7.49561646	1.00490995	0.00706623	0.20864861	0.89438311	0.9447757
Ms4a15	1.78965726	1.02361441	0.03367236	0.21796455	0.16535019	0.33404624
Msantd2	422.454501	-1.0579803	-0.0813128	0.10071977	0.36021582	0.56053992
Msantd3	985.549334	-1.0853012	-0.1180955	0.08118861	0.11486777	0.25787876
Msantd4	1756.22371	-1.0814592	-0.1129793	0.0693764	0.08425677	0.20582174
Msh2	2424.2606	1.07545588	0.10494835	0.06602928	0.09436381	0.22371831
Msh3	1030.3606	-1.0950823	-0.1310393	0.07846015	0.07193766	0.18276903
Msh4	5.80363567	-1.0211997	-0.030265	0.21442996	0.45570651	0.64999448
Msh5	38.608256	1.11043963	0.15113096	0.23851974	0.16631676	0.33535617
Msh6	4273.33545	1.04732485	0.066709	0.05244489	0.18508588	0.36214298
Msi2	1127.57755	1.19132809	0.25257078	0.07452748	0.00030777	0.00212881
Msl1	2551.94266	1.01104199	0.01584291	0.06918875	0.80931463	0.89580758
Msl2	3356.42998	-1.1152197	-0.157328	0.05725579	0.00424808	0.01942109
Msl3	3385.68239	-1.157634	-0.2111792	0.06473693	0.00060511	0.00382413
Msl3l2	366.10186	1.00340426	0.00490297	0.11580205	0.96094971	0.98140523
Mslnl	12.7962759	1.02269102	0.03237033	0.20765486	0.62067907	0.777138
Msmo1	3311.06557	-1.1237248	-0.1682888	0.06925655	0.00997695	0.03917988
Msn	18952.8147	-1.0206436	-0.0294791	0.04290598	0.48249548	0.67201447
Msra	15.7676421	1.10553851	0.14474928	0.2596954	0.11042461	0.25076186
Msrb1	182.029989	1.16200795	0.21661993	0.17670669	0.08519104	0.20758036
Msrb2	131.21721	1.72487606	0.78649271	0.20371693	8.53E-06	8.98E-05
Msrb3	2887.41935	1.06981334	0.0973591	0.05488338	0.06645197	0.17298309
Mss51	3.00708687	-1.0095571	-0.0137224	0.21390095	0.65255981	0.7986117
Mst1	31.4865788	1.02237366	0.03192258	0.19545333	0.71355875	0.83844135
Mst1r	330.526649	1.07722171	0.10731522	0.13165133	0.30959448	0.50795425
Msto1	564.843392	1.05895738	0.08264452	0.11220427	0.38844497	0.59006048
Msx1	265.884051	1.15290818	0.20527762	0.11939516	0.04277484	0.12391355
Mta1	5200.5725	1.09217532	0.12720446	0.05877999	0.02410528	0.0795847

Mta2	1771.17996	-1.0586286	-0.0821965	0.05653222	0.13103226	0.28267485
Mta3	2319.151	1.10840833	0.14848946	0.0701585	0.02483266	0.08129477
Mtarc1	125.289962	1.13654167	0.18465059	0.18105243	0.13514846	0.28926513
Mtarc2	908.839846	-1.0153818	-0.0220223	0.08185081	0.77031881	0.87232885
Mtbp	7225.58566	-1.3826257	-0.4674106	0.04650788	1.62E-24	1.26E-22
Mtch1	3197.69031	1.56497099	0.64613592	0.05940005	1.67E-28	1.64E-26
Mtch2	5313.2272	-1.0634465	-0.0887475	0.0516881	0.07634689	0.19142762
Mtcl1	2711.34755	-1.1510961	-0.2030082	0.05549372	0.00014714	0.00114167
Mtcp1	8.44493067	-1.0710523	-0.0990289	0.24009851	0.10004718	0.23306136
Mtdh	5485.77358	-1.0766501	-0.1065495	0.05914121	0.06467268	0.16980058
Mterf2	184.443159	1.03430339	0.04865943	0.13160222	0.64296083	0.79201333
Mterf3	1310.04037	1.10250609	0.14078662	0.07899565	0.0548775	0.14918946
Mterf4	784.66587	1.06246077	0.08740957	0.09705604	0.31491756	0.51409379
Mtf1	579.32766	-1.0816851	-0.1132805	0.10367306	0.21202531	0.39783862
Mtf2	736.187065	-1.0364686	-0.0516764	0.10549336	0.58345393	0.74830245
Mtfmt	718.141803	1.04610503	0.0650277	0.09650129	0.45090593	0.64618934
Mtfp1	125.351332	1.00488564	0.00703133	0.14161014	0.94926773	0.97475909
Mtfr1	1093.94746	-1.3223397	-0.4030928	0.0724947	5.88E-09	1.12E-07
Mtfr1l	1337.08266	1.08910261	0.12313988	0.07988797	0.09588559	0.2260638
Mtfr2	1211.27282	-1.0566285	-0.0794682	0.08165261	0.29050733	0.48706303
Mtg2	1138.44729	1.23418178	0.3035549	0.08467685	0.00011136	0.00089552
Mthfd1	8996.33416	-1.0473769	-0.0667807	0.03860761	0.07847535	0.19531169
Mthfd1l	4582.03765	-1.1022354	-0.1404323	0.05497097	0.00803455	0.03258073
Mthfd2	3935.52982	-1.690751	-0.7576642	0.06279735	1.37E-34	1.70E-32
Mthfd2l	940.649855	-1.2954693	-0.3734748	0.10646493	0.0001034	0.00083777
Mthfr	584.454449	1.02956246	0.04203135	0.09824112	0.63153379	0.78505074
Mthfsd	114.785231	1.08278706	0.11474956	0.16972492	0.31935429	0.5177988
Mtif2	1122.16614	-1.0244515	-0.0348517	0.08087167	0.64109774	0.79087655
Mtif3	235.085275	-1.11704	-0.1596808	0.16039559	0.17298082	0.34558395
Mtln	64.8655897	1.00070228	0.00101281	0.17020434	0.99350627	0.99708676
Mtm1	266.089504	1.06075567	0.08509239	0.11650358	0.38444215	0.5856468
Mtmr1	1654.20475	1.01836015	0.02624788	0.07262434	0.70042602	0.82955412
Mtmr10	1291.76881	1.0972108	0.13384073	0.08803606	0.0956429	0.22567427
Mtmr11	66.1143845	1.0072816	0.01046706	0.17653223	0.91931559	0.95836855
Mtmr12	1042.15129	1.06287855	0.08797675	0.06699655	0.16857395	0.33900037
Mtmr14	1266.14474	1.04610082	0.06502189	0.07235553	0.33969319	0.53919807
Mtmr2	3456.43974	-1.1973119	-0.259799	0.0557974	1.39E-06	1.75E-05
Mtmr3	2362.54102	1.20337194	0.26708262	0.06744856	3.14E-05	0.00028831
Mtmr4	1574.41126	1.03837321	0.05432507	0.06826915	0.40119981	0.60084279
Mtmr6	3947.86455	1.16112643	0.21552507	0.06336341	0.00036312	0.00245596
Mtmr9	3776.04852	1.27261096	0.34779145	0.05608448	1.60E-10	3.90E-09
Mto1	685.387026	-1.4423497	-0.528421	0.10851269	1.51E-07	2.26E-06
Mtor	3967.21552	1.13100303	0.1776028	0.05436113	0.00071155	0.00437689
Mtpn	11081.1965	-1.0663762	-0.0927165	0.04222672	0.02430658	0.08010477
Mtr	2353.47372	-1.0139807	-0.0200302	0.05907957	0.72362266	0.84414955
Mtres1	597.986506	1.03573602	0.05065635	0.10829463	0.58912231	0.75240733
Mtrex	5709.2015	-1.1488021	-0.2001303	0.04769225	1.35E-05	0.00013539
Mtrf1	937.895326	1.00133845	0.00192969	0.07858997	0.97968823	0.99074811
Mtrf1l	483.259161	-1.1401702	-0.1892491	0.09999315	0.03291829	0.10109886
Mtrr	1309.27208	-1.1826546	-0.2420287	0.07295056	0.00041743	0.00277495

Mtss1	2043.03984	-1.2116051	-0.2769196	0.0875036	0.00057502	0.00366764
Mtss2	415.629414	1.45966453	0.54563683	0.1448081	1.98E-05	0.00018986
Mttp	2.22616774	-1.035345	-0.0501115	0.22364064	0.0123005	0.04646295
Mturn	11.2871296	-1.1421178	-0.1917114	0.34095105	0.01823768	0.06369268
Mtus2	17.1572775	-2.692754	-1.4290824	0.76839915	0.00182082	0.0096463
Mtx1	1403.44596	1.07659045	0.10646953	0.08041516	0.15233797	0.31469915
Mtx2	2077.11163	1.03473043	0.04925497	0.07517641	0.48448029	0.67336397
Mtx3	138.258979	1.09483057	0.13070762	0.1466523	0.24320391	0.43312137
Mul1	273.568429	-1.0931416	-0.1284803	0.1203374	0.20114231	0.38362368
Mus81	116.799655	-1.0350618	-0.0497169	0.14812238	0.64629434	0.79438021
Musk	4.87747521	-1.0058983	-0.0084844	0.21186158	0.82603405	0.90649329
Mutyh	103.487682	-1.0223582	-0.0319008	0.15296357	0.76692042	0.87111526
Mvb12a	807.8652	1.19076217	0.2518853	0.08987966	0.00215945	0.01110945
Mvb12b	710.434778	1.27152135	0.34655569	0.09901034	0.00012162	0.00096703
Mvd	568.006788	1.00659282	0.00948022	0.1006412	0.91541388	0.95578488
Mvk	748.231501	1.09071167	0.12526978	0.10339946	0.1671463	0.33671081
Mvp	788.835376	-1.2228771	-0.2902794	0.08874606	0.00037256	0.00251447
Mx2	773.147848	1.31601826	0.3961795	0.09485862	6.41E-06	6.89E-05
Mxd1	59.4557128	1.0559577	0.07855204	0.17350156	0.47690515	0.66730256
Mxd3	32.5270158	-1.0206159	-0.02944	0.19200511	0.74256209	0.85638464
Mxd4	553.636763	1.51096487	0.59547011	0.10939014	6.01E-09	1.14E-07
Mxi1	396.064556	-1.1053314	-0.144479	0.12276988	0.15707239	0.32219332
Mxra7	185.875519	1.04522936	0.06381956	0.14541383	0.55877595	0.73256779
Mxra8	11.1359711	1.01247134	0.01788107	0.20296567	0.80119254	0.89005953
Myadm	474.17076	1.06063807	0.08493244	0.11065798	0.37143132	0.57284315
Mybbp1a	8388.03995	-1.172293	-0.2293332	0.04150047	1.68E-08	2.96E-07
Mybl1	769.297215	-1.0542293	-0.0761887	0.08816453	0.34270402	0.54218087
Mybl2	7695.13031	1.1157089	0.15796066	0.04844402	0.00078891	0.00478501
Mybpc1	9.42081686	-1.0090002	-0.0129265	0.20828115	0.81203667	0.89735937
Mybpc3	3.97443165	1.05156114	0.07253274	0.2297735	0.08678334	0.2105214
Myc	14334.4555	-1.208372	-0.2730646	0.04399477	2.26E-10	5.28E-09
Mycbp2	10205.1291	-1.0284229	-0.0404336	0.04849174	0.39135445	0.59272293
Mycl	3.52212785	-1.0018637	-0.0026863	0.21055241	0.95026579	0.97531249
Myd88	1640.54406	-1.0786915	-0.1092823	0.07228702	0.10724383	0.24514515
Mydgf	853.527019	1.25688343	0.32985085	0.11290772	0.00097133	0.00569199
Myg1	831.63137	1.03759514	0.05324362	0.07757238	0.46171331	0.65527892
Myh10	13802.1112	-1.0665166	-0.0929064	0.04825848	0.04640315	0.13159715
Myh11	18.4657081	-4.0237131	-2.0085275	0.56306863	1.53E-05	0.00015223
Myh14	2.61975183	1.02833621	0.04031202	0.21968203	0.12072607	0.26677608
Myh2	3.26987418	-1.0527691	-0.0741891	0.23238336	0.03619475	0.10871156
Myh3	23.205547	-1.0380207	-0.0538351	0.2031663	0.51957101	0.702309
Myh7b	20.5534735	-2.7130857	-1.4399346	0.53898635	0.00028264	0.00198936
Myh9	32193.651	1.14041206	0.1895552	0.04628532	2.67E-05	0.00024722
Myl12a	4667.92288	1.08520355	0.11796567	0.0524714	0.02027208	0.06936041
Myl12b	4430.05455	1.01973787	0.02819834	0.06461036	0.64924069	0.79654949
Myl3	1.8165055	1.0002382	0.00034361	0.21400504	0.99017243	0.9954343
Myl4	3.82513174	-1.0017823	-0.0025691	0.21181815	0.94497379	0.97295716
Myl6	6269.91798	1.06772488	0.09453995	0.06987797	0.15203208	0.31421926
Myl6b	421.223693	1.02267272	0.03234452	0.10166963	0.71878551	0.84156113
Myl9	51.9191872	2.64064641	1.40089113	0.29947063	1.63E-07	2.43E-06

Mylk2	21.6821596	-1.1328723	-0.1799853	0.28588643	0.08190276	0.20145437
Mynn	571.651396	1.05149934	0.07244794	0.11587556	0.45852371	0.6527046
Myo10	1342.42667	-1.0441382	-0.0623127	0.08230817	0.41063433	0.60980907
Myo15b	2.68798841	1.01270267	0.01821066	0.21393665	0.58592649	0.74971062
Myo18a	6467.63522	1.0865764	0.11978962	0.05251253	0.0185326	0.06454667
Myo19	1118.57471	-1.0496578	-0.069919	0.07300286	0.30746561	0.50580251
Myo1a	13.5222183	-1.0593545	-0.0831855	0.22490905	0.23792783	0.42764915
Myo1b	4667.515	-1.5821293	-0.6618675	0.0493488	5.53E-42	9.84E-40
Myo1c	12170.565	1.18201263	0.24124545	0.04935588	4.92E-07	6.69E-06
Myo1d	74.6765918	-1.0800709	-0.1111261	0.18797366	0.32083146	0.51951094
Myo1e	4173.63966	-1.4056535	-0.491241	0.05958635	2.68E-17	1.31E-15
Myo1g	27.6107098	-9.723981	-3.2815471	0.68395007	8.69E-08	1.36E-06
Myo5a	6002.66651	1.16705215	0.22286903	0.06913232	0.00065393	0.00407833
Myo6	1543.0619	-1.0351172	-0.0497941	0.08520625	0.52388265	0.70567158
Myo7a	3.10301964	1.05104811	0.0718287	0.23176855	0.02123871	0.07201372
Myo9a	5681.00547	1.08436442	0.11684968	0.06070925	0.0444449	0.12739744
Myo9b	4567.40778	1.06402499	0.08953204	0.05627605	0.09337193	0.22186875
Myof	20931.1248	1.23603586	0.30572059	0.05364972	5.12E-09	9.81E-08
Myom1	24.8560897	-1.1784871	-0.2369359	0.37539685	0.03750739	0.11197149
Myom2	25.5651106	-1.8659125	-0.8998814	0.73791991	0.00571018	0.02461219
Myom3	5.73423264	-1.0539049	-0.0757447	0.2322925	0.05582304	0.15121446
Myorg	45.6757092	-1.0720193	-0.1003308	0.21186867	0.30788997	0.50610426
Mypn	4.82852234	1.01263423	0.01811316	0.21263661	0.65573889	0.8004869
Mypop	149.14277	1.02694168	0.03835425	0.14930393	0.71873066	0.84156113
Myrf	4.09418234	1.02408011	0.03432858	0.21577479	0.38563431	0.58683483
Myrfl	2.59996832	-1.0107102	-0.0153694	0.21459584	0.56116571	0.73397312
Myrip	4.67600441	1.02292811	0.03270476	0.21642642	0.32936822	0.52925324
Mysm1	2788.55462	1.07803419	0.10840293	0.06060339	0.06208576	0.16430177
Myzap	448.916084	1.04489022	0.06335137	0.10060006	0.47530473	0.66622755
Mzt1	173.653072	-1.0524165	-0.0737057	0.1523695	0.50218347	0.68715484
Mzt2	1301.63426	1.04585225	0.06467905	0.08749548	0.4159352	0.61489808
N4bp1	2873.85207	1.03591752	0.05090914	0.06063178	0.38205593	0.58397551
N4bp2	1631.63893	-1.026381	-0.0375664	0.06505925	0.54380091	0.72157548
N4bp2l2	1851.73513	1.01089913	0.01563905	0.07043644	0.81561542	0.9000718
N4bp3	89.7524858	-1.1538067	-0.2064015	0.19700877	0.10727226	0.24516639
N6amt1	653.194638	1.10711304	0.14680253	0.09115042	0.07523158	0.18917986
NAT10	2468.11247	-1.0709118	-0.0988397	0.06087651	0.08955266	0.21542603
ND1	59942.3752	-1.0373824	-0.0529477	0.14906275	0.66364142	0.80543405
ND2	57366.1891	1.03733689	0.0528845	0.15885506	0.64238297	0.7917758
ND3	9039.8976	-1.0435868	-0.0615506	0.14742603	0.56024379	0.7332914
ND4	112101.28	1.17781832	0.23611702	0.21598554	0.08038063	0.19866407
ND4L	11665.8625	1.11028212	0.15092631	0.20062271	0.2017631	0.38426781
ND5	37802.2792	1.03075344	0.04369928	0.16041255	0.69914708	0.82849333
ND6	5260.49885	-1.0967018	-0.1331713	0.22495494	0.21127475	0.39683718
NDOR1	1375.8716	-1.1230966	-0.167482	0.07420253	0.01577868	0.05674478
NDUFA1	183.37652	-1.0005229	-0.0007542	0.13904307	0.9931654	0.9968955
NDUFB11	1784.23621	-1.0043901	-0.0063197	0.07657534	0.92893458	0.96415913
NEU2	777.887423	-3.8028083	-1.9270652	0.10923686	5.08E-71	3.42E-68
NSF	25876.8933	-1.0448823	-0.0633404	0.03808158	0.09059419	0.21728356
NTHL1	376.125268	1.10927435	0.14961622	0.12564007	0.149073	0.30990345

NUBP1	747.723232	1.01648772	0.02359278	0.08311049	0.75555398	0.86397773
Naa10	1276.21019	-1.0571021	-0.0801148	0.06774951	0.21148305	0.39705378
Naa16	338.99076	-1.1565601	-0.2098402	0.1302585	0.04987192	0.13874622
Naa25	2887.83072	1.00684273	0.00983835	0.05516801	0.85431944	0.92212943
Naa30	1889.41161	-1.0357754	-0.0507112	0.06655764	0.42227658	0.62078474
Naa38	514.285697	1.01083602	0.01554899	0.09667486	0.85194741	0.92042373
Naa50	3988.87229	-1.0578724	-0.0811656	0.07216813	0.23158518	0.42073552
Naa60	341.61554	1.0327448	0.0464838	0.10338592	0.60924039	0.76937519
Naa80	200.866903	1.1414445	0.19086071	0.17136662	0.11774155	0.2621855
Naaa	22.9340395	-1.1511041	-0.2030183	0.3148431	0.06088576	0.16188182
Naaladl2	45.5363599	-1.1113526	-0.1523166	0.24505816	0.15321191	0.31618941
Nab1	3395.17869	-1.1327708	-0.179856	0.05339002	0.00048694	0.00317925
Nab2	1471.42504	1.00633183	0.0091061	0.07504737	0.89767113	0.94658933
Nabp2	854.993727	1.07908296	0.10980578	0.08334201	0.15108723	0.31297339
Nacad	47.0725857	1.05541979	0.07781694	0.1968779	0.43874715	0.63641537
Nacc1	1824.7833	1.00002176	3.14E-05	0.06332855	0.9995626	0.99979674
Nacc2	226.307777	1.02942354	0.04183667	0.1246133	0.68245797	0.8171125
Nadk	3220.38374	1.10854141	0.14866266	0.06628496	0.01818118	0.06356466
Nadk2	1268.4308	1.01389757	0.01991192	0.08452525	0.79839214	0.88833522
Nadsyn1	531.785073	1.12164516	0.16561635	0.12139092	0.10428199	0.24056407
Nae1	1011.72921	-1.0423229	-0.0598022	0.08270062	0.43246249	0.63037983
Naf1	619.068963	-1.1782011	-0.2365858	0.10206335	0.00910709	0.03620787
Naga	2204.79947	1.26829223	0.3428872	0.06565736	5.16E-08	8.41E-07
Nagk	289.848106	1.29066695	0.36811677	0.12555006	0.00075636	0.00462042
Naglu	425.363087	1.29061714	0.36806109	0.10311044	8.46E-05	0.00070212
Nagpa	625.368138	-1.0472034	-0.0665417	0.08967679	0.41323036	0.61222939
Naif1	291.595994	-1.0959732	-0.1322125	0.12181596	0.19214408	0.37085516
Nalcn	4.80451432	-1.0441313	-0.0623032	0.22511489	0.12359259	0.27184428
Nampt	5599.60173	-1.1365943	-0.1847173	0.05165828	0.00022396	0.0016441
Nanos3	3.19140957	1.00806078	0.01158262	0.21267129	0.75194147	0.86249734
Nanp	445.43163	-1.0414774	-0.0586315	0.10569382	0.52359263	0.70549906
Nans	2455.09937	1.00090012	0.00129802	0.05863521	0.98218784	0.99108517
Nap1l4	4511.6693	-1.1316575	-0.1784373	0.05358147	0.00057244	0.00365731
Napa	593.673381	-1.0351615	-0.0498559	0.09433538	0.5550927	0.73030078
Napb	31.2528623	1.06020883	0.08434846	0.21190017	0.35217246	0.55192034
Napepld	288.278705	-1.4330759	-0.519115	0.14549074	4.63E-05	0.00040824
Napg	1309.23102	-1.0590304	-0.082744	0.06201644	0.16245014	0.32973955
Naprt	50.460055	1.12363534	0.1681739	0.23028258	0.16007069	0.32620196
Narf	892.962704	-1.1720748	-0.2290646	0.10866077	0.01585517	0.05698786
Nars2	553.71692	-1.1674311	-0.2233374	0.11096426	0.02054058	0.07007318
Nasp	8762.21665	-1.1069388	-0.1465755	0.05576544	0.00636233	0.02685385
Nat14	7.74082561	-1.0243766	-0.0347462	0.21490311	0.41953813	0.61830228
Nat8	3.29780384	-1.0687778	-0.095962	0.24752156	0.00065784	0.00409871
Nat9	491.000425	1.26711831	0.34155123	0.11966714	0.00108701	0.00624993
Natd1	6.87621449	-1.0273104	-0.0388722	0.21525742	0.40006113	0.60013033
Nav1	1037.67655	1.10905927	0.14933647	0.07834421	0.04007676	0.11777063
Nav2	5000.89104	-1.1884758	-0.2491126	0.06248999	2.96E-05	0.00027262
Nav3	2722.33531	-1.3208012	-0.4014133	0.05590594	1.55E-13	5.41E-12
Naxd	383.126346	1.07973613	0.11067879	0.10669557	0.23295782	0.42235768
Naxe	1069.20525	1.24035673	0.3107551	0.1076324	0.0011789	0.00667401

Nbas	1812.88616	1.20388694	0.26769991	0.06501177	1.58E-05	0.00015629
Nbdy	9.1643792	1.02423194	0.03454246	0.21335943	0.48718776	0.67563876
Nbea	3130.6945	1.10708892	0.14677111	0.06252679	0.01389367	0.05133484
Nbeal1	618.421518	1.10533691	0.14448618	0.089713	0.07612784	0.19095314
Nbeal2	380.745657	-1.1092252	-0.1495523	0.13375463	0.16550392	0.33424329
Nbl1	448.978928	1.44750943	0.53357275	0.12591156	2.91E-06	3.40E-05
Nbn	1083.52118	1.09682272	0.13333036	0.07174827	0.04815638	0.13529516
Nbr1	7794.1898	-1.0354701	-0.0502858	0.05104501	0.30962189	0.50795425
Ncald	30.696886	-1.8732796	-0.9055663	0.78040226	0.00583323	0.02501629
Ncam1	13288.5774	-1.277745	-0.3536	0.04210475	4.82E-18	2.51E-16
Ncapd2	6089.53488	-1.0850836	-0.1178062	0.04730183	0.01076868	0.04178939
Ncapd3	5255.25968	-1.0107596	-0.0154398	0.05091095	0.74171797	0.85605975
Ncapg	3204.48217	-1.044926	-0.0634008	0.05594233	0.23983364	0.42944771
Ncapg2	4715.2768	-1.0362099	-0.0513163	0.04944059	0.28544882	0.48190417
Ncaph	3884.48303	-1.0585975	-0.0821541	0.05340494	0.11160886	0.25259886
Ncbp1	3667.30464	-1.0037843	-0.0054492	0.05177935	0.91294357	0.95506796
Ncbp2	1127.74193	1.24016052	0.31052686	0.09121479	0.00021998	0.00162413
Ncbp3	555.876379	1.11531549	0.15745186	0.11416655	0.10621536	0.24379479
Ncdn	1344.03617	-1.0289172	-0.0411269	0.09583295	0.62905326	0.78322064
Nceh1	1458.74877	-1.1549358	-0.2078127	0.07165862	0.00204232	0.01060051
Nck1	1108.41536	1.00644617	0.00927001	0.09590399	0.91499959	0.95558004
Nck2	1329.62893	-1.0602129	-0.0843539	0.06384416	0.16534529	0.33404624
Nckap1	10253.1047	1.10228068	0.14049163	0.05551052	0.00843026	0.03398102
Nckap5l	746.361098	-1.0946283	-0.130441	0.09680282	0.13079973	0.28245862
Nckipsd	110.754617	1.41092321	0.49663947	0.23928358	0.00400571	0.01853131
Ncln	2520.78517	-1.1259371	-0.1711262	0.06425771	0.00517141	0.02271023
Ncoa1	3027.58219	1.23034829	0.29906678	0.04845612	2.47E-10	5.75E-09
Ncoa2	1951.82603	1.036148	0.05123008	0.05725804	0.35343192	0.5530739
Ncoa3	5233.52686	1.53353519	0.61686128	0.04508307	1.51E-43	2.97E-41
Ncoa5	1725.17357	1.14321446	0.19309607	0.06577632	0.00198884	0.01038187
Ncoa6	5217.38755	1.07333937	0.1021063	0.0503508	0.03681894	0.11027603
Ncoa7	1618.88549	1.05446083	0.0765055	0.06809109	0.2360564	0.42571906
Ncor1	4472.9467	1.09719299	0.13381731	0.04428456	0.00196887	0.0103028
Ncor2	4053.60607	1.04831929	0.06807819	0.0679459	0.29124465	0.48801098
Ncs1	2414.03416	1.29483676	0.37277023	0.05894691	6.63E-11	1.69E-09
Ncstn	5209.78296	1.19758768	0.26013128	0.05746557	2.60E-06	3.08E-05
Ndc1	4214.2748	-1.1265813	-0.1719515	0.0528067	0.00075425	0.00460972
Ndc80	7743.1638	1.03411224	0.04839278	0.06254068	0.41947498	0.61828035
Nde1	2838.81543	1.18214939	0.24141237	0.06065797	3.31E-05	0.00030148
Ndel1	1327.81685	1.18473207	0.24456083	0.06739552	0.00013231	0.00103916
Ndfip1	1663.64053	1.08628381	0.11940108	0.0638902	0.0497184	0.13850229
Ndfip2	1517.26585	-1.0246759	-0.0351676	0.08238538	0.64300848	0.79201333
Ndp	60.4943084	1.01627021	0.02328405	0.17785729	0.81978867	0.90288822
Ndrg3	793.444737	1.03289838	0.04669833	0.08855257	0.5625528	0.73502849
Ndst1	1161.90987	-1.0154776	-0.0221584	0.06980515	0.73657453	0.8528127
Ndst2	1262.23742	1.15899448	0.2128737	0.08112208	0.00459125	0.02068725
Ndufa10	9184.47051	1.02506897	0.03572098	0.05902925	0.52838559	0.70920152
Ndufa11	798.70405	1.04965591	0.06991647	0.11246813	0.46811749	0.66059458
Ndufa12	1914.99701	1.04527584	0.06388371	0.08757767	0.42831803	0.62608502
Ndufa13	837.96791	1.0349512	0.04956274	0.09423239	0.55869632	0.73253837

Ndufa3	155.002908	1.24859491	0.32030549	0.16833719	0.01397181	0.05159378
Ndufa4	1164.38254	1.07502105	0.10436491	0.09406275	0.21659045	0.40295557
Ndufa4l2	3.75904238	1.01756143	0.02511589	0.21464476	0.4726357	0.66401222
Ndufa5	2097.74658	-1.030872	-0.0438652	0.06024739	0.44781915	0.64361307
Ndufa6	586.581803	-1.0528526	-0.0743034	0.0889508	0.35762638	0.55772996
Ndufa7	1159.64398	1.08115871	0.11257832	0.08247705	0.13848445	0.29419334
Ndufa8	2410.20913	1.10931529	0.14966947	0.0690612	0.02185019	0.07365815
Ndufa9	2615.06207	1.0324254	0.04603755	0.05641934	0.41458577	0.61333223
Ndufab1	3186.96607	-1.0338314	-0.0480009	0.07072572	0.47167948	0.66339108
Ndufaf1	1521.17699	1.05924926	0.08304212	0.06754732	0.19524712	0.37531747
Ndufaf3	133.790031	-1.0024315	-0.0035037	0.14982847	0.97272194	0.98767978
Ndufaf4	288.58781	-1.0648366	-0.0906321	0.11028948	0.33890781	0.53850274
Ndufaf5	505.704139	1.09703939	0.13361533	0.09168988	0.10703686	0.24497806
Ndufaf6	223.558448	1.00380468	0.00547858	0.11850132	0.95682754	0.97901352
Ndufaf7	1046.72868	-1.025456	-0.0362656	0.08218174	0.63215482	0.78541342
Ndufaf8	2.29153624	-1.0189055	-0.0270202	0.2157496	0.3712211	0.57265683
Ndufb10	961.132363	1.04195171	0.05928842	0.0818421	0.43381465	0.63171146
Ndufb2	208.752249	1.03274862	0.04648913	0.16983346	0.6658769	0.80655133
Ndufb3	2908.64219	-1.0002298	-0.0003314	0.06457031	0.99541355	0.99813552
Ndufb5	2639.85233	1.12430978	0.16903959	0.05784093	0.00235506	0.01196208
Ndufb6	1178.47189	1.0097137	0.01394629	0.08444902	0.85837498	0.92418976
Ndufb7	1117.65223	1.13223227	0.17916995	0.08460089	0.02110057	0.07164016
Ndufb8	1076.60462	1.02244725	0.03202642	0.0862808	0.68621597	0.8196191
Ndufb9	5509.49467	-1.0991421	-0.1363779	0.06214726	0.02141405	0.07251227
Ndufc1	1668.66468	1.06351147	0.08883559	0.152462	0.42376142	0.62216434
Ndufc2	2073.94808	1.03696127	0.05236201	0.07432314	0.45304577	0.64794108
Ndufs2	1782.16188	1.08018996	0.11128505	0.06602492	0.07580594	0.19023208
Ndufs3	1281.08786	1.12075429	0.16447002	0.06982767	0.0125183	0.04706763
Ndufs4	1788.58676	1.03058054	0.04345725	0.06720182	0.49951076	0.6853827
Ndufs5	1928.00817	1.13034798	0.17676698	0.08016235	0.01734487	0.06137784
Ndufs7	407.64858	1.2034352	0.26715846	0.12496734	0.0116063	0.04443416
Ndufs8	1437.22815	1.08841537	0.12222924	0.06606973	0.05121619	0.14157241
Ndufv1	3966.61146	1.14840963	0.19963733	0.06348627	0.00096963	0.00568723
Ndufv2	3547.49763	1.04338431	0.06127064	0.07335719	0.37397184	0.57551408
Ndufv3	1803.94033	-1.0118675	-0.0170203	0.06429023	0.78083193	0.87733155
Neb	197.282727	1.72803769	0.78913468	0.25908856	0.00015608	0.00120301
Necab1	2.54035015	-1.0031728	-0.0045701	0.21365447	0.86957818	0.92935896
Necab3	3.96307062	-1.031322	-0.0444949	0.21952343	0.19416806	0.37391655
Necap1	2053.31815	1.05813527	0.08152407	0.07230268	0.23063058	0.41954037
Necap2	972.349435	-1.0284578	-0.0404826	0.07360431	0.55741805	0.73141185
Nectin3	1582.69212	-1.1399172	-0.1889291	0.06982395	0.00410332	0.0188738
Nedd1	1487.30437	-1.0028091	-0.004047	0.06833265	0.9496679	0.97493555
Nedd4	26597.6454	1.08035684	0.11150791	0.04303297	0.00814032	0.03294707
Nedd9	1.84760483	-1.030831	-0.0438078	0.22159402	0.02359554	0.07822435
Neil1	135.435883	1.31889631	0.39933115	0.18537659	0.00514772	0.02264502
Neil2	6.0670783	1.00484885	0.00697851	0.20887729	0.89450777	0.9447757
Neil3	2036.74132	-1.7129331	-0.7764688	0.07868029	4.80E-24	3.58E-22
Nek1	2635.60516	1.11647571	0.15895187	0.07591077	0.02488764	0.08137077
Nek2	4785.21368	1.17853063	0.23698925	0.06072038	4.67E-05	0.00041141
Nek3	199.192199	1.11903326	0.16225292	0.14653334	0.15178092	0.31390274

Nek4	965.777494	-1.0693967	-0.0967971	0.08500079	0.2139813	0.39986924
Nek5	7.4634902	-1.135593	-0.1834459	0.34242275	0.00507276	0.02240761
Nek6	2281.39635	-1.1513823	-0.203367	0.06602208	0.00115586	0.00656612
Nek7	5817.49628	1.01633835	0.02338077	0.04531649	0.598416	0.76139342
Nek8	184.314378	1.37115465	0.4553913	0.17940475	0.00156036	0.00842671
Nek9	2082.81506	-1.0106377	-0.015266	0.05748701	0.78228128	0.87827816
Nelfa	1780.02421	-1.0490372	-0.0690658	0.05975823	0.22820354	0.41707623
Nelfb	1667.70704	1.04971005	0.06999088	0.08801829	0.38314864	0.58445306
Nelfcd	1251.68351	1.41075867	0.49647121	0.07913944	5.38E-11	1.39E-09
Nelfe	1160.98513	1.10864089	0.14879213	0.09470934	0.07978427	0.19775664
Nemf	1783.4661	-1.0631492	-0.0883441	0.08211943	0.24228869	0.43227271
Nemp1	1242.85532	-1.3560736	-0.4394355	0.09355527	4.81E-07	6.55E-06
Nemp2	186.019045	1.04582114	0.06463614	0.14268908	0.55119091	0.72763936
Nenf	1056.20184	1.22726209	0.29544337	0.08340125	0.0001373	0.00107242
Neo1	5829.79564	1.06079049	0.08513974	0.04391543	0.04737304	0.13357884
Nepn	4.82672368	1.00184816	0.00266387	0.21132654	0.95033962	0.97531249
Nepro	662.522872	-1.1321311	-0.1790411	0.09678804	0.03871488	0.11471775
Net1	4219.15812	1.05169378	0.0727147	0.04992439	0.13213496	0.28419221
Neu1	926.053192	1.64307797	0.71640094	0.0927909	1.08E-15	4.63E-14
Neu3	238.764781	1.09737494	0.13405653	0.14183906	0.22449104	0.41301653
Neurl1a	102.692344	1.23075564	0.29954436	0.24646701	0.04314144	0.12477801
Neurl1b	84.7459293	1.01554452	0.02225349	0.16271165	0.83580612	0.91190498
Neurl2	71.313912	1.03433477	0.0487032	0.17944651	0.63896946	0.7904586
Neurl3	232.514018	-1.5205746	-0.6046166	0.17832765	6.69E-05	0.00057054
Neurl4	1029.85946	1.56663955	0.64767328	0.07632426	2.47E-18	1.31E-16
Nexn	43.2214314	1.01124262	0.01612917	0.18585488	0.86590573	0.92774752
Nf1	4311.64644	1.23573062	0.30536428	0.0542909	6.59E-09	1.24E-07
Nf2	3541.92849	1.00686557	0.00987108	0.056473	0.85530534	0.92271319
Nfat5	5346.99366	-1.1045814	-0.1434997	0.06536846	0.02072284	0.07060096
Nfatc1	944.059827	-1.0791378	-0.1098791	0.08513349	0.15820898	0.32385059
Nfatc2ip	932.046584	-1.0131412	-0.0188352	0.08631514	0.81086529	0.89697834
Nfatc3	3323.25548	1.01904266	0.02721444	0.05542604	0.61212583	0.77186061
Nfatc4	2.71971365	-1.0037213	-0.0053587	0.2133199	0.85853689	0.92418976
Nfe2l1	7876.11844	1.22422311	0.29186651	0.05442354	3.06E-08	5.18E-07
Nfe2l2	5161.77576	-1.4487589	-0.5348176	0.05262216	4.12E-25	3.39E-23
Nfia	1515.31402	-1.1312403	-0.1779054	0.07254306	0.00865422	0.03474165
Nfib	864.099878	1.02295812	0.03274709	0.09332557	0.69798267	0.82770938
Nfic	1076.08446	-1.0955755	-0.131689	0.08809173	0.10000345	0.2330378
Nfil3	145.266516	-1.3653141	-0.4492329	0.22115228	0.00515352	0.02265498
Nfix	1141.32634	1.25477655	0.32743048	0.098197	0.00024751	0.00178724
Nfkb1	2278.68124	1.00806171	0.01158396	0.06249034	0.84699804	0.91770658
Nfkb2	5408.18756	-1.0875688	-0.1211067	0.05335186	0.01874872	0.06499894
Nfkbib	425.067127	-1.0437513	-0.061778	0.10614027	0.50303852	0.68784183
Nfkbid	2.16370949	1.00833274	0.01197179	0.21441116	0.64215007	0.79161344
Nfkbie	111.769909	-1.2105111	-0.2756163	0.20161123	0.04455302	0.12765022
Nfkbil1	238.170249	-1.0322949	-0.0458552	0.13682953	0.66469124	0.80585792
Nfkbiz	158.691428	-1.4193304	-0.5052105	0.27738035	0.00572692	0.02466772
Nfrkb	992.161546	1.05367817	0.07543429	0.07690482	0.29301114	0.49013746
Nfs1	1689.85828	1.05445876	0.07650268	0.06451807	0.21361505	0.39935914
Nfu1	661.252613	1.17828819	0.23669244	0.11162239	0.01482615	0.0541091

Nfx1	1443.64442	1.05319067	0.07476665	0.07694182	0.29806044	0.4957998
Nfxl1	1113.34394	-1.0471688	-0.066494	0.07928702	0.36611966	0.56724635
Nfya	1575.49581	1.00357342	0.00514617	0.06951872	0.9383398	0.97014793
Nfyb	216.677739	1.06201568	0.08680506	0.1478863	0.43164703	0.62965965
Nfyc	1911.65327	1.00243549	0.0035094	0.08530384	0.96504534	0.98295812
Ngdn	2234.66453	1.00655997	0.00943313	0.06176725	0.87399243	0.93213247
Ngef	146.388211	1.15419822	0.20689102	0.18806593	0.10410308	0.24040971
Ngf	408.464435	1.05891196	0.08258265	0.13786833	0.44283962	0.64003536
Ngly1	546.900638	1.00187881	0.002708	0.09559364	0.97456143	0.98845067
Ngrn	950.202278	1.00504639	0.00726209	0.07987435	0.92279676	0.96059048
Nhlrc2	2032.25516	1.00123865	0.00178589	0.05631584	0.9743265	0.98843721
Nhlrc3	302.989488	1.25583149	0.32864289	0.13465675	0.00387432	0.01803415
Nhp2	726.666692	-1.0118292	-0.0169658	0.08586275	0.82907256	0.90788335
Nhs	777.56475	1.01782565	0.02549046	0.09546391	0.76648683	0.87106819
Nhsl1	1543.11826	-1.0903908	-0.1248453	0.06230491	0.03574104	0.10772769
Nhsl2	443.787307	-1.2059686	-0.2701924	0.11302208	0.00612847	0.02602111
Nicn1	106.188091	1.19728048	0.25976116	0.21366909	0.05985573	0.1597067
Nid1	10707.3574	1.06480857	0.09059409	0.04710729	0.05390074	0.14712732
Nifk	3909.21444	-1.1887729	-0.2494731	0.07491701	0.00037989	0.00255991
Nim1k	172.68787	1.2743662	0.34977991	0.17321575	0.0091898	0.03645752
Nin	6987.56986	-1.0261886	-0.037296	0.0503979	0.44608403	0.64256565
Ninj1	724.71156	-1.5690555	-0.6498963	0.10856903	2.21E-10	5.18E-09
Ninj2	23.0441013	-1.0750931	-0.1044617	0.23503272	0.17162842	0.34352501
Ninl	632.336749	1.0399091	0.05645743	0.09636813	0.51299826	0.69679862
Nipa1	10.5068167	-1.021491	-0.0306764	0.21031348	0.58802226	0.75160299
Nipa2	772.733387	1.08422007	0.11665762	0.09183395	0.15958008	0.32567047
Nipal3	1451.28998	1.06337128	0.08864541	0.07674803	0.21559756	0.40183394
Nipbl	9094.81515	-1.0469762	-0.0662287	0.04275556	0.11364505	0.25558259
Nipsnap1	435.372384	1.0027326	0.00393693	0.10279914	0.96795382	0.98447705
Nipsnap2	769.85576	-1.1276565	-0.1733277	0.0912531	0.03509858	0.10619103
Nipsnap3b	613.334672	-1.1619329	-0.2165267	0.09605632	0.01185405	0.04522047
Nisch	3389.93129	1.20068759	0.26386082	0.06518269	2.17E-05	0.00020524
Nit1	613.699205	1.24686665	0.31830718	0.09180352	0.00016187	0.00124162
Nit2	460.88869	1.14680748	0.19762322	0.10336936	0.03023043	0.09436397
Nkain1	3639.61571	-1.0212852	-0.0303858	0.06311831	0.61403854	0.7730549
Nkap	955.085916	-1.1030298	-0.1414718	0.08345739	0.06493063	0.17023359
Nkapd1	1095.14276	-1.0759032	-0.1055482	0.09286078	0.206819	0.39127919
Nkiras1	335.641586	-1.0019455	-0.002804	0.12119169	0.98482171	0.99194575
Nkiras2	1033.39515	1.07680336	0.10675482	0.08120714	0.15500917	0.31882907
Nkrf	1585.42058	-1.178492	-0.2369419	0.06701806	0.00019366	0.00145418
Nktr	2364.09664	1.09622609	0.13254537	0.0809028	0.07648047	0.19168626
Nle1	1384.13981	-1.0398589	-0.0563878	0.09462255	0.50481699	0.68920357
Nlgn1	113.514744	1.21487681	0.28081003	0.20568664	0.04289052	0.12413636
Nlgn2	1596.32967	1.72856586	0.78957557	0.07818088	5.18E-25	4.20E-23
Nlgn3	2.39206044	-1.0100105	-0.0143703	0.21405722	0.63138018	0.78505074
Nlk	825.271977	-1.1242326	-0.1689406	0.08835801	0.03546857	0.10698195
Nln	1912.44842	1.02314793	0.03301474	0.05957035	0.56324978	0.7353474
Nlrc4	3.53089194	-1.0243168	-0.034662	0.21456863	0.4367287	0.63429644
Nlrc5	10.9970909	-1.0808177	-0.1121232	0.24521497	0.10911331	0.24844322
Nlrp3	5.00732319	-1.0594582	-0.0833266	0.23776712	0.01591037	0.05712216

Nmd3	1419.68368	1.00860942	0.01236761	0.06879746	0.85027034	0.91935375
Nme1	4340.39687	1.09891038	0.13607374	0.06075517	0.01925535	0.06643172
Nme2	10633.0019	1.0388952	0.05505013	0.05579912	0.30723364	0.50567428
Nme3	137.398322	1.04185303	0.05915177	0.14324598	0.58558468	0.74968416
Nme6	501.207262	-1.0000722	-0.0001042	0.09660665	0.99802375	0.99951782
Nme7	639.146908	-1.0121534	-0.017428	0.10235954	0.84565909	0.91684699
Nmi	1201.87031	-1.0418603	-0.0591619	0.09223591	0.47667476	0.66719882
Nmnat1	1221.36445	-1.0033858	-0.0048764	0.08060848	0.94718568	0.97387018
Nmnat3	235.844601	1.12850413	0.1744117	0.153629	0.13721508	0.29232083
Nmt2	1123.82515	1.02970136	0.04222598	0.08945163	0.60433694	0.76574602
Nnmt	199.582569	-2.5142657	-1.3301371	0.20046506	1.81E-12	5.64E-11
Noa1	1365.75258	-1.0660466	-0.0922705	0.08279653	0.22526426	0.41388915
Nob1	1719.52465	-1.1716174	-0.2285016	0.0682231	0.00040383	0.00269993
Nobox	2.82188868	1.04488776	0.06334799	0.22850058	0.01292992	0.0482893
Noc2l	5224.30421	-1.137987	-0.1864841	0.04948819	0.00010356	0.00083855
Noc4l	1271.24093	1.00074911	0.00108033	0.07851957	0.98907647	0.99482331
Noct	1320.99268	-1.3355264	-0.4174084	0.06944231	3.84E-10	8.60E-09
Nod1	609.925735	-1.6790205	-0.7476199	0.10334605	4.43E-14	1.61E-12
Nod2	60.6285298	-1.4535613	-0.539592	0.3295748	0.00760573	0.03111768
Nol10	3048.81482	-1.1012173	-0.1390992	0.0606797	0.01569628	0.05655958
Nol11	4888.85617	-1.0023301	-0.0033578	0.05116064	0.94523993	0.97295716
Nol12	1045.66436	-1.2157641	-0.2818634	0.07930044	0.00014151	0.00110267
Nol3	124.826041	1.914509	0.93697444	0.21701401	1.10E-06	1.40E-05
Nol6	1447.30039	-1.0790305	-0.1097357	0.07245463	0.10641701	0.2440827
Nol7	2265.19094	-1.0401581	-0.0568028	0.07157738	0.39954245	0.59966476
Nol9	5240.57873	-1.1219555	-0.1660154	0.04976772	0.0005752	0.00366764
Nolc1	7338.00934	-1.1343064	-0.1818104	0.05828892	0.00112589	0.00643007
Nom1	2407.13036	-1.8897818	-0.9182197	0.08032666	2.29E-31	2.57E-29
Nomo1	6731.64204	-1.1025608	-0.1408582	0.04988151	0.00359884	0.01696766
Nono	12305.9044	-1.0642479	-0.0898343	0.04958199	0.06346729	0.16708096
Nop10	1967.88406	-1.2241102	-0.2917335	0.06596167	3.58E-06	4.08E-05
Nop14	3565.30723	-1.1254366	-0.1704848	0.07189647	0.01086381	0.04211922
Nop16	1407.05669	-1.0778363	-0.108138	0.10841299	0.24865727	0.4395942
Nop2	5740.88287	-1.0867431	-0.1200109	0.05601638	0.02163392	0.07310221
Nop53	1213.0085	-1.1049019	-0.1439183	0.07323168	0.03582759	0.10788703
Nop56	9898.59582	-1.2264863	-0.2945312	0.05789725	1.34E-07	2.03E-06
Nop58	8111.54614	-1.1411614	-0.1905029	0.08327209	0.01301625	0.04854093
Nop9	2216.23321	-1.0488556	-0.0688161	0.05730367	0.21196646	0.39782488
Nos1ap	235.161131	1.3756139	0.46007559	0.15495826	0.00044941	0.00295984
Nosip	673.711138	-1.0154459	-0.0221134	0.08633231	0.77841691	0.87661719
Notch1	161.908262	-1.3554825	-0.4388065	0.17336606	0.00169111	0.00903831
Notch2	4913.35777	-1.0177776	-0.0254224	0.04758162	0.58325473	0.74830245
Notch3	247.477622	1.41529796	0.50110581	0.17391075	0.00049157	0.00320458
Nova1	1150.27749	-1.0267224	-0.0380462	0.06490699	0.53809978	0.71675763
Nox1	2.63335685	-1.0083535	-0.0120015	0.21414243	0.66191572	0.8043199
Nox4	2349.49808	-1.0022858	-0.003294	0.07297581	0.96223601	0.98209252
Noxa1	350.168561	1.1077653	0.14765225	0.11760231	0.13742842	0.29262934
Noxo1	12.5296073	1.15508501	0.20799904	0.36985759	0.01537955	0.0556028
Noxred1	2.09783265	-1.0326913	-0.046409	0.22194187	0.03906129	0.1155334
Npas1	12.1750037	-1.0312662	-0.0444167	0.21076759	0.48961915	0.67718402

Npat	4354.70469	1.00462781	0.00666111	0.0525149	0.89655393	0.94572265
Npc1	1210.68051	-1.2942652	-0.3721332	0.07660458	2.88E-07	4.08E-06
Npc2	1195.06497	1.22340933	0.29090719	0.09441755	0.00072019	0.00441627
Npdc1	76.9316268	-1.1344118	-0.1819444	0.21137782	0.14494548	0.30428573
Npepl1	3.96057867	1.00777923	0.01117963	0.21196271	0.77998671	0.87690293
Npepps	6274.87437	-1.1039276	-0.1426456	0.05013282	0.00335588	0.01602268
Nphp1	1392.51095	1.18177049	0.24094988	0.07649367	0.00076586	0.00466894
Nphp3	694.469437	1.0207125	0.02957656	0.09174964	0.72255595	0.84367348
Npl	2.65014054	1.01417158	0.02030174	0.21365733	0.58078634	0.74705021
Nploc4	12027.7998	1.12705542	0.17255846	0.05157652	0.00055482	0.00356431
Npm2	8.2646678	-1.0579332	-0.0812486	0.2296923	0.15787183	0.32336714
Npm3	1254.87973	-1.0886494	-0.1225394	0.09222592	0.14044565	0.29742251
Nppb	115.114234	-2.7861344	-1.4782649	0.21951655	9.00E-13	2.90E-11
Npr1	35.2313592	4.43937088	2.15035524	0.46834735	2.72E-07	3.89E-06
Npr2	794.52602	1.22963307	0.29822787	0.08752988	0.00022145	0.00163031
Nprl2	283.561163	1.07255923	0.10105732	0.11883029	0.31037328	0.50875005
Nprl3	561.69745	-1.099642	-0.1370339	0.09996896	0.12038433	0.26628282
Nptn	2964.32415	-1.0575923	-0.0807836	0.05564147	0.13216141	0.28420138
Nptxr	2581.39672	1.00390354	0.00562065	0.07592584	0.93755331	0.96972611
Nqo1	194.715641	-1.6928276	-0.7594351	0.19104443	5.47E-06	5.98E-05
Nqo2	324.014484	-1.0739828	-0.1029709	0.12202609	0.30774433	0.50605968
Nr1d1	1243.95221	-1.2757512	-0.351347	0.08965836	2.30E-05	0.00021651
Nr1d2	689.997476	1.02576399	0.03669883	0.09782024	0.67284648	0.81086907
Nr1h2	1035.09684	-1.0166283	-0.0237923	0.0895154	0.76956721	0.87194021
Nr1h3	445.046289	-1.0215089	-0.0307018	0.13433428	0.76891722	0.87151208
Nr1h5	12.789236	-39.330385	-5.2975724	1.0856272	2.06E-07	3.01E-06
Nr2c1	1335.30312	-1.1395345	-0.1884446	0.06638123	0.0027668	0.01370033
Nr2c2	793.596922	1.18582873	0.24589565	0.07926078	0.00085901	0.00514925
Nr2c2ap	810.071434	-1.0131341	-0.0188251	0.09233654	0.82086097	0.90344715
Nr2f1	86.8613631	-1.0256779	-0.0365778	0.16783035	0.73078044	0.849168
Nr2f2	194.610549	-1.0328125	-0.0465783	0.13422164	0.65751652	0.80140161
Nr2f6	150.568773	-1.1445781	-0.1948159	0.20745521	0.12599082	0.27494761
Nr3c1	2336.31241	1.04922431	0.06932315	0.057464	0.21117556	0.39670903
Nr3c2	553.301923	1.41288218	0.49864116	0.09641245	3.49E-08	5.79E-07
Nr4a1	66.0082228	-1.0657337	-0.091847	0.17810674	0.4083303	0.60737472
Nr4a2	406.058765	-1.0879488	-0.1216106	0.10019526	0.16939388	0.34043546
Nr4a3	58.1424694	-1.1539097	-0.2065303	0.25209907	0.10686077	0.24474665
Nr6a1	81.0014078	1.14476209	0.1950478	0.19675244	0.12530913	0.27411373
Nradd	5.57791763	1.01663933	0.02380796	0.21229215	0.60580467	0.76690956
Nrap	600.110743	-1.0646404	-0.0903662	0.09646537	0.29342231	0.49066471
Nras	1876.1404	-1.078059	-0.1084362	0.06116313	0.06399787	0.1683048
Nrbp1	2150.21364	1.12912796	0.17520898	0.07342236	0.01087177	0.04211922
Nrcam	16.8873789	-1.065257	-0.0912015	0.23114428	0.17559565	0.34915407
Nrd1	6382.0982	1.19098162	0.25215115	0.04260988	1.45E-09	2.99E-08
Nrde2	1006.51406	1.03365009	0.04774789	0.07556545	0.50016763	0.68576981
Nrf1	2000.22205	-1.1385461	-0.1871928	0.06505873	0.00245838	0.01244739
Nrg2	295.502775	-1.5142147	-0.5985698	0.14758904	5.08E-06	5.59E-05
Nrgn	52.8150825	-1.0317697	-0.045121	0.18474331	0.65056035	0.79740485
Nrip1	870.011058	-1.0111559	-0.0160055	0.09490018	0.85003085	0.91935375
Nrm	170.933302	1.18671455	0.24697296	0.17563004	0.05319692	0.14576525

Nrp1	2594.88464	-1.4562534	-0.5422614	0.07247797	1.01E-14	3.88E-13
Nrp2	3393.5501	-2.0820235	-1.0579863	0.06395596	1.26E-62	5.97E-60
Nrsn2	18.0602926	1.08764094	0.12120236	0.23715545	0.1819691	0.35795826
Nsd1	4736.61403	1.02461851	0.03508686	0.04642562	0.44131783	0.63878886
Nsd2	10052.9714	1.0536188	0.075353	0.04641879	0.09642314	0.22713873
Nsd3	3635.08575	1.08882573	0.12277306	0.06587819	0.04975127	0.13854646
Nsdhl	2058.72262	1.0401489	0.05679007	0.06063537	0.32890591	0.52884206
Nsfl1c	4853.37002	-1.0202458	-0.0289168	0.044573	0.50658407	0.69116434
Nsl1	1523.81447	1.1002682	0.13785524	0.07284331	0.04349086	0.12561846
Nsmaf	1491.26361	1.11222238	0.15344528	0.06363294	0.01137625	0.0436578
Nsmce1	742.849054	-1.0009752	-0.0014062	0.09474157	0.98293768	0.99129521
Nsmce4a	1720.59909	-1.0958509	-0.1320515	0.0904658	0.10670202	0.24447377
Nsmf	2059.48355	-1.0917188	-0.1266013	0.07272622	0.06327635	0.16674964
Nsrp1	908.505117	1.00453074	0.00652171	0.08274246	0.9327551	0.96688223
Nsun2	9209.26677	-1.0911738	-0.1258808	0.04749585	0.00645718	0.02713793
Nsun3	204.956526	-1.1362116	-0.1842315	0.14410206	0.10178916	0.23626004
Nsun4	532.767464	1.02725231	0.03879057	0.09790051	0.65709881	0.80127899
Nsun5	892.612886	-1.1174167	-0.1601673	0.0884313	0.04628099	0.13130887
Nsun6	775.555245	1.11975895	0.16318819	0.08434698	0.03502023	0.10600404
Nt5c	67.3869754	1.06188735	0.08663073	0.17749547	0.43434565	0.63198545
Nt5c2	1177.14968	1.29260968	0.3702867	0.07672954	3.45E-07	4.82E-06
Nt5c3	850.17977	-2.1433683	-1.0998798	0.0815227	1.38E-42	2.52E-40
Nt5c3b	745.347342	1.09037274	0.1248214	0.08919903	0.12365509	0.27193505
Nt5dc1	934.197635	-1.3142209	-0.3942078	0.10315824	2.80E-05	0.00025867
Nt5dc2	2500.52549	1.18114403	0.2401849	0.0730559	0.0004749	0.00310696
Nt5el	9.57160793	-1.0246875	-0.035184	0.20786866	0.59145798	0.75456396
Nt5m	8.36023491	-1.0289546	-0.0411794	0.21389073	0.44145301	0.63891233
Ntan1	1184.61601	1.43235984	0.51839397	0.10314478	6.95E-08	1.11E-06
Ntmt1	719.866425	-1.0431497	-0.0609463	0.09735083	0.48208921	0.67187634
Ntn4	560.175719	-1.0314303	-0.0446463	0.10730358	0.6320344	0.78541342
Ntpcr	314.658041	-1.0094887	-0.0136248	0.10984771	0.88430927	0.93790377
Nts	9.91128427	1.01091354	0.01565962	0.20866877	0.77759986	0.87600512
Nuak1	1171.33465	1.03191942	0.04533032	0.07482581	0.51873604	0.70169046
Nuak2	138.851035	1.43705437	0.52311464	0.24010546	0.00289694	0.01418571
Nub1	1023.01809	-1.6597983	-0.7310079	0.07579356	5.06E-23	3.58E-21
Nubp2	169.919361	1.11063878	0.15138968	0.1368951	0.16437623	0.33259509
Nubpl	676.078256	1.24973807	0.32162575	0.11889332	0.00191097	0.01003972
Nucb1	2507.68903	1.3486558	0.43152219	0.0640516	3.39E-12	1.02E-10
Nucb2	3902.85972	-1.0876237	-0.1211795	0.06066106	0.03680819	0.11027603
Nudcd2	809.255382	-1.1865886	-0.2468198	0.08912503	0.00246377	0.0124648
Nudcd3	3868.4394	1.03222823	0.04576199	0.0507441	0.35306352	0.55269995
Nudt12	1153.57512	1.08768016	0.12125438	0.08246387	0.11093274	0.25146848
Nudt13	113.676674	-1.0030722	-0.0044255	0.16829521	0.9649209	0.98295812
Nudt15	76.2141815	1.02164052	0.03088764	0.17011694	0.77059555	0.87248798
Nudt16l1	66.4575244	1.15889803	0.21275363	0.23146751	0.10500896	0.24168912
Nudt18	267.643089	1.0157898	0.02260189	0.11935992	0.8209309	0.90344715
Nudt19	179.800735	1.11552857	0.15772747	0.14957037	0.16629927	0.33535617
Nudt2	395.839987	-1.036489	-0.0517048	0.10489336	0.57168173	0.7403946
Nudt22	250.855771	1.01228264	0.01761216	0.12025085	0.86072479	0.92561153
Nudt3	513.164769	-1.0054892	-0.0078976	0.093596	0.92472938	0.96122057

Nudt4	3376.66745	-1.3397116	-0.4219224	0.05346204	6.10E-16	2.66E-14
Nudt5	1074.12437	-1.148825	-0.2001591	0.07408477	0.00393557	0.01823975
Nudt6	71.9679058	-1.0563229	-0.0790509	0.19397897	0.4399315	0.63736213
Nudt9	1884.98568	1.04472727	0.06312636	0.06626492	0.31660078	0.51558939
Nuf2	2155.99923	-1.0214762	-0.0306555	0.0693934	0.64018297	0.79058554
Nufip1	1939.97074	-1.024244	-0.0345595	0.07105954	0.60527263	0.76631176
Nufip2	10584.2601	-1.1745869	-0.2321534	0.05221569	4.37E-06	4.90E-05
Numa1	9804.81839	-1.131141	-0.1777788	0.04983776	0.00023562	0.00171398
Numb	1092.22227	1.04803194	0.06768269	0.08054284	0.36470546	0.56573952
Numbl	356.861378	-1.2038397	-0.2676433	0.13538917	0.01610173	0.05764762
Nup107	3562.66677	-1.0419832	-0.059332	0.05935344	0.29765385	0.49563834
Nup133	4648.17885	1.09292236	0.12819092	0.05008258	0.00837628	0.03380599
Nup153	8322.22618	-1.1125396	-0.1538566	0.04602412	0.00060184	0.00380533
Nup155	4708.60121	-1.0786339	-0.1092052	0.05688024	0.04596338	0.1306392
Nup160	6055.87513	-1.1226879	-0.166957	0.05485829	0.00157515	0.00849588
Nup188	3666.27167	-1.0218408	-0.0311705	0.06012397	0.58841577	0.75195591
Nup205	5953.02844	-1.0670262	-0.0935956	0.05108833	0.05933861	0.15872365
Nup210l	7.24119962	-1.0360473	-0.0510898	0.21715339	0.3316843	0.53157462
Nup214	5065.89578	1.03141375	0.04462318	0.05435522	0.39603992	0.59664487
Nup35	915.63125	-1.1460552	-0.1966766	0.08935818	0.01547214	0.05586192
Nup37	1517.32308	-1.2512778	-0.3234021	0.06413695	1.44E-07	2.16E-06
Nup50	1612.3746	-1.2993143	-0.3777505	0.07030085	1.91E-08	3.35E-07
Nup54	3475.94775	1.02794518	0.03976332	0.06486959	0.52066194	0.70309668
Nup62	1835.65909	-1.0518512	-0.0729307	0.0556425	0.17410796	0.34729414
Nup85	2287.61411	-1.0283914	-0.0403894	0.05800529	0.46954537	0.66155699
Nup88	2490.25879	-1.0126978	-0.0182038	0.06663392	0.77320865	0.87382468
Nup93	3841.9104	-1.0268829	-0.0382717	0.05248483	0.45152012	0.64675978
Nup98	11654.7229	-1.0430486	-0.0608064	0.0424808	0.1438304	0.30268891
Nupl2	246.03057	-1.6962735	-0.7623688	0.14283539	7.61E-09	1.42E-07
Nupr1	464.100792	-2.6826949	-1.423683	0.13101472	9.56E-29	9.50E-27
Nus1	5250.33513	-1.0689216	-0.096156	0.05294122	0.06041155	0.16085469
Nusap1	5259.74015	-1.1846661	-0.2444805	0.05342328	2.28E-06	2.74E-05
Nutm1	8.69808261	1.00465347	0.00669797	0.20758307	0.90682123	0.95184464
Nvl	2230.40278	-1.1505311	-0.2023	0.06608983	0.00126335	0.00704549
Nwd1	100.460247	1.05126872	0.0721315	0.15895181	0.50672093	0.69116973
Nxf1	2576.09539	1.07613391	0.10585762	0.07246816	0.11976375	0.26524441
Nxf7	15.1791	-1.1204003	-0.1640143	0.29016347	0.05229684	0.14385281
Nxn	403.284171	1.0724701	0.10093743	0.09880379	0.25033104	0.44163898
Nxpe3	413.945229	1.20919881	0.27405147	0.11385297	0.00574327	0.02471321
Nxpe4	127.364373	1.05444588	0.07648506	0.16634507	0.49254267	0.67949091
Nyap1	224.241053	1.18613795	0.24627181	0.16088373	0.04422875	0.12699016
Oaf	2265.73399	-1.7623339	-0.8174873	0.06931644	3.50E-33	4.19E-31
Oard1	627.210818	1.08858915	0.12245956	0.10038888	0.16868528	0.33917099
Oasl1	102.418919	1.15404225	0.20669604	0.1994876	0.11063286	0.25105525
Oasl2	24.3283147	-1.080493	-0.1116898	0.23099551	0.20904426	0.39444057
Oat	311.647029	1.59882789	0.67701465	0.16272898	3.02E-06	3.53E-05
Oaz1	2323.0466	1.0335344	0.0475864	0.08786654	0.56848798	0.73857312
Oaz2	2178.13483	-1.0446447	-0.0630123	0.05855547	0.26268193	0.45558564
Obsl1	454.970118	1.44316904	0.52924029	0.15619921	8.64E-05	0.00071522
Ocel1	151.857049	1.1027589	0.14111741	0.14325424	0.20250487	0.38540324

Ociad2	951.978528	1.03316376	0.04706895	0.07195291	0.48840554	0.67686537
Ocrl	2817.58203	1.05427148	0.07624642	0.05265836	0.13501994	0.28908665
Ocstamp	15.4728383	1.22673397	0.29482242	0.54683103	0.01670357	0.05930507
Odf1	3.28673753	-1.0051875	-0.0074647	0.21344573	0.80179673	0.89053446
Odf2	2780.69535	1.1785814	0.2370514	0.06426525	0.00011049	0.00088902
Odf2l	408.731947	1.08425694	0.11670667	0.11979446	0.24544177	0.43577396
Odr4	630.923882	-1.0739998	-0.1029937	0.09516519	0.22614266	0.41467312
Ofcc1	5.62579707	1.01684513	0.02409997	0.21308841	0.56247605	0.73502849
Ofd1	2012.16167	1.1021154	0.14027529	0.07222131	0.03874425	0.11475465
Oga	968.870892	-1.2111174	-0.2763387	0.08447548	0.00040915	0.00272414
Ogdh	7909.93581	1.06533619	0.09130878	0.04322516	0.03049227	0.09501484
Ogfod1	2395.58722	-1.1500531	-0.2017004	0.05767307	0.00027322	0.00193471
Ogfod2	104.339856	1.0995364	0.13689536	0.18976302	0.24414707	0.43437833
Ogfod3	1334.0796	-1.005916	-0.0085098	0.07928751	0.90707577	0.95192853
Ogfr	2009.91666	1.12696581	0.17244375	0.05663707	0.00155138	0.00838532
Ogg1	321.628631	1.21386905	0.27961279	0.11999298	0.00676786	0.02823567
Ogt	2862.3489	-1.024297	-0.0346341	0.06157392	0.55443053	0.72989503
Oip5	105.491216	-1.0797827	-0.110741	0.17272927	0.33223517	0.53205807
Oit3	2.94515863	1.04373108	0.06175005	0.22687303	0.0479472	0.13487074
Ola1	4474.49529	-1.059298	-0.0831085	0.06826145	0.20172525	0.38425286
Olfm1	809.07515	1.47171965	0.55750288	0.13227438	3.07E-06	3.57E-05
Olfm2	33.7530429	1.49618645	0.58128997	0.50440566	0.01101593	0.04249145
Olfm5	2.17025527	-1.0010796	-0.0015567	0.21397382	0.9480198	0.97437186
Olfml2b	953.325382	1.35020074	0.43317391	0.07946401	9.48E-09	1.74E-07
Olfml3	502.395532	1.32485529	0.40583479	0.13000658	0.00034599	0.00235785
Olfr1009	6.70040792	1.00207788	0.00299463	0.20913072	0.95452917	0.97803088
Olfr1013	7.83801164	-1.0590907	-0.0828262	0.23265467	0.10687843	0.24474665
Olfr1014	3.17276699	1.04827301	0.0680145	0.22916415	0.05571264	0.15097925
Olfr1377	1.86458558	-1.0203695	-0.0290916	0.21685018	0.25641794	0.44848631
Olfr644	2.72222887	-1.0119869	-0.0171906	0.21489825	0.49983703	0.68561006
Olfr994	2.20447092	-1.0103924	-0.0149157	0.21511419	0.4850493	0.6739865
Oma1	484.232318	1.1626252	0.21738608	0.10186066	0.01626084	0.05806761
Opa1	7672.67353	1.0715473	0.09969554	0.05000536	0.04001886	0.11776743
Opa3	966.535519	-1.1027138	-0.1410584	0.08101351	0.05912132	0.1583446
Ophn1	314.967748	1.04360562	0.06157662	0.10198885	0.49353007	0.68023673
Oplah	217.889417	-1.0631064	-0.088286	0.15763067	0.42804932	0.6260203
Opn3	14.5963156	-1.0322538	-0.0457977	0.20406329	0.56019886	0.7332914
Opn4	1.90889474	-1.0272632	-0.0388058	0.21961331	0.10501496	0.24168912
Optc	2.48045318	1.03646646	0.05167344	0.22418856	0.01014036	0.0397363
Optn	2064.72533	1.32732264	0.4085191	0.06069564	3.82E-12	1.15E-10
Orai1	216.590323	-1.3321282	-0.4137329	0.14438516	0.00072942	0.00447076
Orai2	226.642397	-1.0785485	-0.1090911	0.13531093	0.30746949	0.50580251
Orai3	104.053439	1.12189991	0.16594397	0.19132001	0.1754703	0.34906133
Orc1	3258.79575	-1.0670902	-0.0936821	0.06414341	0.12518642	0.27398566
Orc2	2277.83664	1.02559264	0.03645782	0.0631149	0.54488174	0.722411
Orc3	1044.07148	1.06117499	0.08566257	0.07487642	0.22168482	0.40954884
Orc4	582.100957	-1.1199881	-0.1634834	0.10322634	0.07183182	0.18253634
Orc5	1521.82163	1.04359113	0.06155658	0.0770381	0.39214464	0.59342856
Orc6	1077.82586	-1.1389364	-0.1876872	0.08347404	0.0145683	0.05330474
Ormdl2	1358.70663	1.09694244	0.13348783	0.07795825	0.06561851	0.17140562

Ormdl3	2825.10396	1.25277667	0.32512926	0.06527852	1.98E-07	2.91E-06
Os9	2822.15774	1.1318688	0.17870674	0.07200387	0.00827898	0.03344489
Osbp	3490.09133	1.05541344	0.07780826	0.05239342	0.12548745	0.27431643
Osbpl10	301.043885	1.17822167	0.23661099	0.14958414	0.0440801	0.12666354
Osbpl11	2285.80563	1.0556564	0.07814033	0.05960586	0.17188442	0.34387365
Osbpl2	3347.47185	1.63733367	0.71134836	0.07145807	2.77E-24	2.11E-22
Osbpl3	999.532549	-1.5517817	-0.6339256	0.10680796	3.36E-10	7.64E-09
Osbpl5	1288.85927	-1.123278	-0.167715	0.08243863	0.02698892	0.08638882
Osbpl7	537.406277	1.17478401	0.23239553	0.10131022	0.0099086	0.03897118
Osbpl8	4081.91452	-1.047714	-0.067245	0.05431043	0.20000773	0.38177605
Osbpl9	1017.05105	-1.0271028	-0.0385806	0.07631806	0.58727022	0.75090728
Oscp1	381.462272	1.16200882	0.21662102	0.11883342	0.03208102	0.09895446
Oser1	1718.18538	1.3076503	0.38697678	0.06786034	2.84E-09	5.66E-08
Osgep	407.571745	-1.0106124	-0.0152297	0.1072746	0.86900453	0.92921103
Osgepl1	382.103567	-1.011053	-0.0158587	0.09952745	0.85670836	0.92361843
Osgin1	55.8048825	-2.1514497	-1.1053091	0.37616159	0.00015084	0.00116755
Osgin2	302.134966	-1.2246948	-0.2924222	0.15925708	0.01865269	0.0647663
Osm	2.63926152	-1.0107016	-0.0153572	0.2135892	0.64155747	0.79121509
Osmr	563.328461	-1.2413089	-0.3118622	0.10331276	0.00077857	0.00473801
Osr2	51.3071197	1.02340031	0.03337057	0.17330526	0.75079871	0.86180389
Ost4	100.595071	-1.1021623	-0.1403367	0.18824465	0.23575275	0.42542782
Ostc	717.324925	1.0167225	0.02392597	0.08747662	0.76528081	0.87006978
Ostf1	3042.51754	1.04832984	0.06809271	0.05918855	0.23063257	0.41954037
Ostm1	1638.90073	1.22866741	0.29709444	0.08156913	9.47E-05	0.00077192
Otc	2.47776245	-1.0053363	-0.0076782	0.21356946	0.79082303	0.88313513
Otogl	65.3699065	-1.1050947	-0.14417	0.19074858	0.22427215	0.41283607
Otub1	1382.81761	1.03062178	0.04351498	0.07538454	0.53768822	0.71635841
Otub2	201.704933	-1.0937504	-0.1292836	0.14571959	0.24427538	0.43451976
Otud3	1577.44781	1.39205329	0.47721444	0.07221645	6.52E-12	1.89E-10
Otud4	5289.07107	-1.1948204	-0.2567937	0.07397971	0.0002232	0.0016404
Otud5	1563.49343	-1.1314178	-0.1781318	0.06408426	0.00361935	0.01705182
Otud6b	1169.66668	1.10265015	0.14097512	0.07732906	0.05028238	0.13963089
Otud7a	18.303913	-1.0660756	-0.0923098	0.22385346	0.25382664	0.44520653
Otud7b	1615.76892	-1.1369984	-0.1852303	0.07459616	0.00788776	0.03209727
Otulin	1000.57139	-1.0367839	-0.0521153	0.07460957	0.45540549	0.64989401
Ovca2	405.375669	1.00515869	0.00742329	0.12232201	0.94237046	0.97194103
Ovgp1	7.92185182	-1.036897	-0.0522725	0.21819204	0.30160023	0.49935363
Oxa1l	1749.66484	-1.0704137	-0.0981685	0.06571227	0.11522847	0.25823595
Oxct1	3953.3939	-1.0359032	-0.0508892	0.05058263	0.30021488	0.49802546
Oxld1	155.248188	1.57923692	0.65922762	0.26834086	0.0010247	0.00596112
Oxnad1	468.55573	-1.2448777	-0.316004	0.13686449	0.00572268	0.02465778
Oxr1	1106.41749	-1.01815	-0.0259501	0.07004022	0.69454933	0.82527263
Oxsm	287.050107	1.02106616	0.03007634	0.13427712	0.7755791	0.87508221
P2rx3	338.053134	1.00132895	0.001916	0.11015515	0.98479411	0.99194575
P2rx4	282.696427	1.26875602	0.34341467	0.14843701	0.00479684	0.02138795
P2rx5	38.592671	1.06184416	0.08657205	0.19921334	0.39585855	0.59657104
P2rx6	7.30454863	1.02571597	0.03663129	0.21383942	0.46237564	0.65585561
P2rx7	46.3854125	-1.027918	-0.0397251	0.18094356	0.69395784	0.82489916
P2ry1	194.872023	1.30462066	0.38363038	0.16566152	0.00389587	0.01810814
P2ry10	4.01824715	-1.0077847	-0.0111874	0.21563468	0.3161822	0.51517349

P2ry10b	12.6087824	-10.219616	-3.3532691	0.87262231	8.91E-06	9.34E-05
P2ry2	176.007371	1.02347626	0.03347764	0.12435123	0.74260114	0.85638464
P3h1	2328.69911	1.25203034	0.32426953	0.06941385	9.32E-07	1.21E-05
P3h2	2752.62744	-1.0438183	-0.0618706	0.05646742	0.25533064	0.44707292
P3h3	730.104106	1.32894615	0.41028265	0.08807607	6.57E-07	8.76E-06
P3h4	1688.05645	1.62730703	0.70248647	0.07255108	3.75E-23	2.68E-21
P4ha1	4606.91343	-1.3361109	-0.4180398	0.06375095	1.16E-11	3.27E-10
P4ha2	232.589481	-1.0295573	-0.0420242	0.1267011	0.68043631	0.8160649
P4ha3	709.327233	1.21686594	0.28317024	0.09607957	0.00119498	0.00673755
P4hb	19911.2849	1.31703484	0.39729351	0.0733849	1.13E-08	2.05E-07
P4htm	69.0571939	-1.0511022	-0.0719029	0.18191441	0.49809682	0.68439561
PAFAH1B1	4913.98137	1.06488947	0.0907037	0.04886746	0.05647696	0.15256661
PANX	587.117906	-1.3652202	-0.4491336	0.10615368	3.99E-06	4.49E-05
PCNA	4084.68364	-1.0923771	-0.127471	0.08018301	0.08535509	0.20771252
PEA15	4305.85048	1.246872	0.31831337	0.06006596	3.80E-08	6.30E-07
PEX19	1357.24493	1.19441339	0.25630225	0.07534731	0.0002878	0.00202009
PEX2	128.890292	-1.0088876	-0.0127654	0.1482432	0.90354181	0.94988097
PEX5	1343.81286	-1.1937045	-0.2554457	0.07649617	0.00035916	0.00243557
PEX7	157.616666	1.08968091	0.12390573	0.14913039	0.27110131	0.46639911
PGAP3	212.071087	1.12535137	0.17037553	0.16190244	0.14937018	0.31042051
PGK1	10423.3422	-1.2247814	-0.2925242	0.08267114	0.00014185	0.0011046
PGS1	555.341497	1.01108071	0.01589817	0.13012511	0.87923868	0.93525426
POFUT1	777.130577	1.16324287	0.21815234	0.08107419	0.00367637	0.01726966
POLR2F	3087.60839	-1.0631492	-0.0883441	0.06279872	0.14022977	0.29701444
PPIA	11348.5875	-1.0778243	-0.108122	0.06648571	0.08628398	0.20949588
PRDX2	2483.67344	1.00760252	0.01092663	0.06118728	0.85058761	0.91950091
PRELID3B	1953.18715	1.35500908	0.43830252	0.06553109	4.45E-12	1.33E-10
PRKACA	5261.08149	1.08824383	0.12200184	0.04520444	0.00568776	0.02453204
PRKACB	2669.24217	-1.0940946	-0.1297375	0.05618131	0.01637661	0.05832203
PRNP	125.218236	-1.0332358	-0.0471695	0.16912864	0.64804224	0.79553628
PTDSS1	1379.83401	1.19571801	0.2578772	0.07729181	0.00036305	0.00245596
PTDSS2	277.075334	1.15801344	0.211652	0.12911671	0.04717331	0.13316022
Pa2g4	13479.0117	-1.2375044	-0.3074337	0.05099059	5.73E-10	1.25E-08
Pabpc1l	4.36098346	-1.0467472	-0.0659131	0.22432228	0.18175515	0.35773382
Pabpc4	7948.46764	1.03050928	0.0433575	0.04289042	0.30322763	0.50095498
Pabpn1	6173.54741	-1.0445668	-0.0629047	0.05322109	0.22169927	0.40954884
Pacc1	360.758838	1.08451974	0.11705632	0.1153439	0.23130594	0.4203962
Pacrg	73.2088156	1.10775378	0.14763725	0.22346231	0.19554813	0.37583969
Pacs1	2323.28182	1.34267816	0.42511353	0.0560669	7.09E-15	2.80E-13
Pacs2	1001.56035	1.22853883	0.29694345	0.0925208	0.00044569	0.00294142
Pacsin2	3821.43166	-1.0773225	-0.1074502	0.04766342	0.02051388	0.0700194
Pacsin3	703.081592	1.01167725	0.01674911	0.09197038	0.84117412	0.91487863
Paf1	2053.05533	-1.0898398	-0.124116	0.06806089	0.0537862	0.14688272
Pafah1b3	929.005185	1.1061379	0.14553125	0.07896006	0.04676873	0.1323117
Pafah2	524.92455	1.04068035	0.057527	0.09894489	0.51401005	0.69765509
Pag1	78.8147372	-1.228749	-0.2971903	0.22836138	0.04064742	0.11912456
Paip2	758.182273	-1.0395637	-0.0559782	0.12527375	0.58323201	0.74830245
Paip2b	460.129657	-1.1098259	-0.1503333	0.10170062	0.09355264	0.22221571
Pak1	1940.64358	1.06699098	0.09354798	0.06292551	0.11941143	0.2648304
Pak1ip1	2423.0981	-1.1621693	-0.2168202	0.0662916	0.00056783	0.00363153

Pak2	7624.46457	1.03037146	0.04316454	0.04816112	0.3565409	0.5565792
Pak3	621.303379	-1.1755329	-0.2333149	0.10205163	0.01015401	0.03977765
Pak4	1159.1408	-1.0148678	-0.0212918	0.08507616	0.7845917	0.87947319
Palb2	408.070764	-1.0855962	-0.1184875	0.10933507	0.20791151	0.39265022
Palld	4607.94436	1.07857854	0.10913123	0.05727375	0.04811922	0.13523633
Palm	1418.30869	-1.0222614	-0.0317642	0.08239176	0.67523297	0.81263946
Pam	8295.50653	1.02896417	0.04119274	0.05510207	0.4396245	0.63705768
Pan2	2720.09831	1.20443129	0.26835209	0.05947011	2.66E-06	3.14E-05
Pan3	2496.85752	-1.0603925	-0.0845984	0.06378268	0.16440233	0.33259536
Pank1	334.591535	1.35771481	0.44118047	0.16566317	0.00120622	0.00678001
Pank2	1294.57972	1.04801142	0.06765444	0.06358819	0.26496607	0.45869182
Pank3	505.154437	1.06428077	0.0898788	0.0908458	0.27559457	0.4710924
Pank4	1033.94212	-1.0454908	-0.0641803	0.08674876	0.41776377	0.61675163
Paox	1558.58403	-1.0053698	-0.0077262	0.06591812	0.9013805	0.94893855
Papln	1.9883062	-1.0053776	-0.0077374	0.21378993	0.78022356	0.87703263
Papolg	1191.76436	1.00533761	0.00768006	0.07203472	0.9111024	0.95407683
Papss1	2374.07188	1.0164642	0.02355941	0.06086549	0.68626109	0.8196191
Paqr3	223.646197	1.23947379	0.30972776	0.14499041	0.00891505	0.03557687
Paqr4	32.1836359	-1.0132618	-0.0190069	0.18844259	0.83560168	0.91187361
Paqr5	1.65087619	1.00701519	0.01008544	0.21500039	0.58756785	0.75109711
Paqr7	39.5951863	-1.0194507	-0.0277921	0.19258504	0.74129714	0.85589602
Paqr8	83.5064439	1.37821775	0.46280384	0.29992632	0.01132863	0.04348809
Pard3	2699.04461	-1.046618	-0.0657349	0.05578555	0.22156485	0.4095154
Pard3b	797.845045	1.34213973	0.42453488	0.089627	4.39E-07	6.01E-06
Pard6a	73.1073746	1.17696674	0.23507355	0.24412556	0.08483417	0.20687717
Pard6b	1292.59185	1.21065146	0.27578358	0.09159011	0.0009754	0.00571062
Parg	1881.95522	1.02364588	0.03371671	0.07250103	0.61980942	0.77673241
Park7	8564.06943	1.16744955	0.2233602	0.04834089	2.03E-06	2.45E-05
Parl	1267.70142	-1.0664749	-0.09285	0.06142172	0.11354589	0.25553809
Parn	2325.64258	1.21311397	0.2787151	0.06878184	1.99E-05	0.00019011
Parp1	6397.01015	-1.0188139	-0.0268906	0.04951521	0.57773182	0.74410617
Parp10	57.113159	1.03884659	0.05498263	0.18558333	0.58900455	0.75240733
Parp11	191.022592	-1.0815224	-0.1130635	0.14029118	0.29907508	0.49689911
Parp12	975.566427	-1.1500885	-0.2017449	0.09847902	0.02155454	0.07289169
Parp14	919.782155	-1.5249593	-0.6087707	0.10330258	4.21E-10	9.37E-09
Parp16	904.149041	1.15833267	0.21204965	0.0964761	0.01440648	0.0528485
Parp2	938.585936	-1.0909169	-0.1255412	0.09161215	0.12904692	0.27970309
Parp3	1607.29425	1.2579777	0.33110635	0.0737617	2.16E-06	2.61E-05
Parp4	1183.33464	1.00970568	0.01393483	0.07746806	0.84813847	0.91832083
Parp6	524.866696	1.25793891	0.33106186	0.11465748	0.0010683	0.00616161
Parp8	615.894749	-1.138118	-0.1866502	0.0934909	0.02642265	0.08504375
Parp9	142.920963	1.14196942	0.19152402	0.19552184	0.13089719	0.2825262
Parpbp	1476.79719	-1.1417769	-0.1912808	0.07827914	0.00851452	0.03427098
Pars2	346.638725	-1.0632743	-0.0885138	0.12318331	0.38186531	0.58380411
Parva	6291.97584	1.12318014	0.16758933	0.05520593	0.00163889	0.0088026
Parvb	3673.70413	-1.2016177	-0.2649779	0.05387543	3.73E-07	5.19E-06
Pask	800.440029	1.2791577	0.35519414	0.08327356	5.27E-06	5.77E-05
Patj	157.969258	1.11627019	0.15868627	0.14888131	0.16314459	0.33067757
Patl1	6493.12666	1.17515142	0.23284666	0.04926314	1.08E-06	1.38E-05
Patz1	225.307383	-1.0233873	-0.0333522	0.14659768	0.75548052	0.86397773

Pawr	2691.77023	-1.0446759	-0.0630555	0.05497136	0.23471365	0.42455265
Pax3	603.466068	1.37422073	0.45861375	0.10984744	4.89E-06	5.41E-05
Paxbp1	2105.12556	-1.0253098	-0.0360598	0.0644672	0.55699752	0.73128402
Paxip1	960.451377	-2.0985749	-1.06941	0.08007814	7.62E-42	1.30E-39
Paxx	138.369821	1.12182093	0.1658424	0.15148414	0.14847405	0.30900936
Pbdc1	1436.18438	-1.0261738	-0.0372751	0.08750211	0.64025411	0.79059718
Pbk	1375.80302	-1.0275528	-0.0392125	0.07657805	0.58295113	0.74830245
Pbld1	10.8957585	-7.4127823	-2.8900151	0.95548079	0.0001091	0.00087901
Pbld2	1.71256552	-1.0134302	-0.0192467	0.21576893	0.35453154	0.55425299
Pbrm1	7073.62547	1.10909536	0.14938341	0.0480851	0.00136006	0.00749802
Pbx1	2583.25118	1.34075834	0.42304922	0.08784908	2.96E-07	4.19E-06
Pbx2	437.131633	1.14973266	0.20129844	0.13467352	0.0653984	0.171005
Pbx3	1651.84869	1.0134624	0.01929256	0.0604204	0.73965241	0.85490818
Pbx4	98.4959694	1.10479796	0.14378256	0.17720806	0.22596078	0.41445555
Pbxip1	1389.14139	1.47999512	0.56559241	0.07501136	6.17E-15	2.46E-13
Pc	1081.72435	1.22186795	0.28908837	0.07907385	9.20E-05	0.00075225
Pcbp1	9366.55523	-1.0659311	-0.0921142	0.06654094	0.14445347	0.30365095
Pcbp3	604.167303	1.17184169	0.22877768	0.10648664	0.01444854	0.0529746
Pcbp4	495.45228	1.46385655	0.54977418	0.10824054	4.99E-08	8.18E-07
Pcca	1442.26775	1.06959032	0.09705831	0.08126576	0.19640829	0.37705918
Pccb	652.049064	1.01563952	0.02238844	0.09620554	0.79560148	0.88630303
Pcdh12	3.6828102	-1.0130407	-0.0186921	0.21418508	0.56116447	0.73397312
Pcdh15	137.784565	-215.98084	-7.7547595	0.65138611	7.57E-32	8.58E-30
Pcdh17	12.8254592	1.04898309	0.06899143	0.22259622	0.24220827	0.43218943
Pcdh18	1510.82227	-1.484956	-0.5704202	0.07435224	2.14E-15	8.94E-14
Pcdh19	914.665245	-1.4313037	-0.5173298	0.09287615	3.60E-09	7.04E-08
Pcdh7	1319.07467	1.48098284	0.56655492	0.08102327	3.49E-13	1.18E-11
Pcdhb13	22.6813641	1.04182297	0.05911015	0.21347467	0.4039828	0.60358923
Pcdhb14	11.318089	1.10752195	0.14733529	0.28755422	0.01801064	0.06319263
Pcdhb15	22.8735067	1.25213578	0.32439101	0.51755916	0.02620542	0.08447192
Pcdhb16	27.7685798	1.02664353	0.03793534	0.20246073	0.62800608	0.78259076
Pcdhb17	19.7519325	1.15218641	0.20437414	0.31778155	0.0606159	0.16129822
Pcdhb18	21.8930617	2.85411051	1.5130412	0.60411019	0.00044452	0.00293904
Pcdhb19	6.88656225	1.05066666	0.07130502	0.22705405	0.14587432	0.30548472
Pcdhb2	4.02436044	1.00994139	0.01427157	0.21370386	0.65298253	0.79884503
Pcdhb20	5.00572406	1.05623165	0.07892628	0.23579656	0.01593411	0.05718387
Pcdhb21	4.52738384	1.06129713	0.08582862	0.239427	0.01520725	0.05513865
Pcdhb22	1.8845788	1.00415486	0.00598178	0.21368843	0.83294153	0.91040793
Pcdhgb2	1.8576377	-1.0155808	-0.0223051	0.21590179	0.34799877	0.54751465
Pcdhgb7	1.65647395	-1.0022885	-0.0032979	0.21350414	0.90740685	0.95207484
Pcdhgc5	2188.04289	-1.0926735	-0.1278624	0.06675836	0.04279979	0.12395002
Pced1a	1566.33051	1.02457783	0.03502958	0.07169665	0.60493392	0.76603435
Pcf11	4132.6974	-1.0330718	-0.0469405	0.06421744	0.44243672	0.63968387
Pcgf1	138.512514	-1.7311289	-0.7917131	0.19401655	3.22E-06	3.72E-05
Pcgf2	3786.40809	1.08477538	0.11739634	0.06176818	0.04666489	0.13212527
Pcgf3	1446.28274	-1.1141581	-0.155954	0.08658748	0.04843532	0.13594575
Pcgf5	1395.89632	1.27878798	0.35477709	0.07279693	3.04E-07	4.29E-06
Pcgf6	172.45242	1.06286919	0.08796406	0.13562478	0.4093863	0.60859215
Pcif1	1147.40831	1.22644582	0.2944835	0.10591929	0.00178088	0.00947626
Pck2	1465.4909	1.37692	0.46144474	0.08653512	1.68E-08	2.96E-07

Pclaf	946.34124	-1.1554213	-0.2084189	0.11453773	0.03413834	0.10409716
Pclo	286.992264	1.08646304	0.11963909	0.12121953	0.23701567	0.42654828
Pcm1	5665.87719	1.01881014	0.02688523	0.04990327	0.58066504	0.74696919
Pcmt1	1403.43613	-1.059324	-0.083144	0.06243611	0.16297763	0.33044372
Pcmtd1	282.632934	1.03917049	0.05543237	0.12018034	0.58316306	0.74830245
Pcmtd2	1060.18058	1.3700665	0.45424592	0.07821269	1.18E-09	2.46E-08
Pcnt	5131.60337	1.15339768	0.20589003	0.05059359	2.74E-05	0.00025344
Pcnx	5340.96139	1.21030165	0.27536666	0.04781942	3.85E-09	7.50E-08
Pcnx2	1.67721173	1.00241253	0.00347636	0.21426871	0.87577428	0.93325585
Pcnx3	1770.76251	1.08545699	0.11830256	0.08008829	0.11042527	0.25076186
Pcnx4	395.46576	1.01069731	0.015351	0.10458515	0.86743723	0.92836933
Pcolce	5547.1126	1.59676047	0.67514791	0.04862534	9.92E-45	2.15E-42
Pcolce2	2205.11862	-1.030152	-0.0428572	0.05914695	0.44914115	0.64475161
Pcp4l1	32.2736339	1.46810176	0.55395197	0.48238123	0.01245581	0.04688772
Pcsk4	1.64661237	-1.0038447	-0.005536	0.21473023	0.75949604	0.8666617
Pcsk5	1.9304883	1.0218133	0.03113162	0.21731158	0.23900368	0.42892086
Pcsk7	1985.27619	-1.0107987	-0.0154957	0.06820983	0.80998432	0.89618424
Pcsk9	1.82886962	-1.0103257	-0.0148204	0.21432629	0.59920082	0.76207947
Pcyox1	1910.15949	1.04109805	0.05810594	0.06490144	0.34794328	0.54749459
Pcyt1a	2903.53427	1.01441384	0.02064633	0.05686663	0.72798559	0.84701611
Pcyt1b	129.811183	1.19789429	0.2605006	0.17009404	0.04004584	0.11777063
Pcyt2	857.548474	1.09072664	0.12528958	0.07948798	0.08900358	0.21459362
Pdap1	5187.01286	-1.0730888	-0.1017694	0.05406564	0.05100813	0.14115699
Pdcd10	1832.50953	-1.1053567	-0.144512	0.06376616	0.01777811	0.06258246
Pdcd11	7139.81875	-1.1065483	-0.1460664	0.04206644	0.0003885	0.00261243
Pdcd2	376.663177	-1.0555227	-0.0779576	0.1300274	0.45408642	0.6488396
Pdcd2l	615.936536	1.15899632	0.21287598	0.10037282	0.01707136	0.06047681
Pdcd4	1186.48937	1.18301078	0.24246322	0.08178885	0.00139323	0.00766636
Pdcd6ip	6346.57833	1.03432923	0.04869547	0.04193289	0.23861969	0.42841181
Pdcd7	652.162279	-1.0394035	-0.0557558	0.09046741	0.49948418	0.6853827
Pdcl	1649.71183	-1.0014639	-0.0021104	0.0685735	0.97339875	0.9878189
Pdcl3	1858.68316	-1.0717008	-0.0999022	0.06540561	0.10766371	0.24566657
Pde10a	392.054781	1.44771171	0.53377434	0.11411997	3.88E-07	5.37E-06
Pde12	900.67538	-1.1160993	-0.1584654	0.07934184	0.03073566	0.09568013
Pde1b	5.57005453	-1.0177159	-0.0253349	0.21064363	0.62714927	0.78218883
Pde1c	15.2403935	-364.56611	-8.5100366	2.78625968	6.36E-11	1.63E-09
Pde4a	423.701062	-1.2862165	-0.3631335	0.11197427	0.00028491	0.00200425
Pde4b	708.251751	-1.2044259	-0.2683457	0.08862394	0.00096559	0.00567396
Pde4d	9.65086491	-1.0272271	-0.0387552	0.21078511	0.5210038	0.70338173
Pde4dip	2689.33978	1.29529314	0.37327863	0.06855669	1.30E-08	2.34E-07
Pde5a	3778.86515	-1.1879005	-0.248414	0.05033682	3.78E-07	5.24E-06
Pde6b	4.79354293	-1.0155363	-0.0222418	0.21268537	0.60029666	0.76272567
Pde6d	1065.07745	1.02348461	0.03348941	0.07339204	0.62776833	0.78249609
Pde7a	595.016676	-1.8922756	-0.9201222	0.10860075	1.77E-18	9.55E-17
Pdf	12.9363849	-1.0535624	-0.0752758	0.21737334	0.31936989	0.5177988
Pdgfa	237.817588	-1.4547446	-0.5407659	0.19608112	0.00061246	0.00386481
Pdgfc	2677.42797	1.01198058	0.01718161	0.06450905	0.78072732	0.87729096
Pdgfra	1639.04164	1.00168219	0.00242484	0.0769932	0.97383502	0.98805671
Pdgfrb	298.408088	-1.0916377	-0.1264941	0.12890804	0.22614414	0.41467312
Pdha1	8574.08812	-1.1851106	-0.2450217	0.0575709	9.79E-06	0.00010187

Pdhx	510.360425	-1.1094389	-0.1498303	0.1083876	0.11096628	0.2515
Pdia3	20537.9198	1.07421405	0.1032815	0.06488592	0.09397746	0.22301803
Pdia4	4730.44935	-2.0539096	-1.0383727	0.06002549	3.51E-68	2.04E-65
Pdia5	1664.36429	1.09335686	0.12876435	0.06810387	0.04619388	0.13114885
Pdia6	15605.9518	1.09438597	0.13012164	0.05137211	0.00917002	0.0364016
Pdik1l	599.457541	-1.1436624	-0.1936612	0.09002839	0.01789082	0.06296194
Pdk2	703.2631	1.14694068	0.19779077	0.09995927	0.02602747	0.08402519
Pdk3	1061.66486	1.07631805	0.10610445	0.08808828	0.18689452	0.36417993
Pdk4	244.790306	1.1090671	0.14934665	0.1252871	0.14839698	0.30891973
Pdlim1	1649.10499	-1.1762042	-0.2341385	0.08141352	0.00191154	0.01003972
Pdlim2	114.169611	1.1041862	0.14298347	0.20112993	0.22453097	0.41301653
Pdlim4	31.1976322	-1.0831825	-0.1152763	0.21922965	0.25279136	0.44402266
Pdlim5	2708.13515	-1.139792	-0.1887706	0.06363771	0.00183446	0.0097145
Pdlim7	2992.63054	1.01223796	0.01754849	0.05794553	0.75365592	0.86298066
Pdp1	456.164831	-1.3371355	-0.4191457	0.11020062	2.71E-05	0.00025056
Pdp2	367.722454	-1.00858	-0.0123255	0.0959075	0.88492607	0.93818984
Pdpk1	1859.24918	1.08180432	0.11343956	0.07155145	0.09209544	0.21965045
Pdpn	270.477741	-1.0909701	-0.1256116	0.12105098	0.21242418	0.39831641
Pdpr	1159.87215	1.18867426	0.24935341	0.06837654	0.0001196	0.00095453
Pdrg1	2713.50393	-1.0542239	-0.0761812	0.06289007	0.20451138	0.38828973
Pds5a	11562.3525	1.10207698	0.140225	0.04845801	0.00296862	0.01445931
Pds5b	5755.73738	1.04454822	0.0628791	0.04817831	0.18091098	0.35675334
Pdss1	583.946846	-1.0949128	-0.130816	0.11876387	0.19016935	0.36887786
Pdss2	690.615344	-1.0013668	-0.0019705	0.08937018	0.98002612	0.99079336
Pdxdc1	2643.79404	1.18556336	0.24557276	0.06270377	4.22E-05	0.00037592
Pdxp	50.0520339	1.00021463	0.00030962	0.17602594	0.9989679	0.99959215
Pdzd11	859.965626	1.03632632	0.05147835	0.0915432	0.53443695	0.71345954
Pdzd2	1286.49899	2.61475823	1.38667756	0.07613872	2.70E-75	2.04E-72
Pdzd7	10.5517729	1.10448128	0.14336897	0.28327856	0.01918903	0.06624666
Pdzd8	1817.13527	-1.1456947	-0.1962226	0.07596419	0.00563392	0.02437372
Pdzd9	48.1534473	1.14261178	0.19233531	0.25179921	0.11891013	0.26390138
Peak1	2430.67996	-1.0538678	-0.0756939	0.05826482	0.17638635	0.35014864
Pecam1	1.61269563	1.00422531	0.00608299	0.21429428	0.79003732	0.88271942
Pef1	1249.73513	1.01599167	0.02288857	0.11346887	0.81328153	0.89842501
Peli1	255.206503	-1.2233883	-0.2908824	0.14157867	0.01190732	0.0453292
Peli2	279.445773	-1.0919903	-0.1269601	0.13030008	0.22733861	0.41591083
Pelo	609.33761	-1.2404974	-0.3109187	0.1095775	0.00135729	0.00748792
Pelp1	1834.76286	-1.0318115	-0.0451795	0.06330243	0.45454524	0.64920555
Pemt	113.525366	-1.0048153	-0.0069303	0.14423585	0.9473506	0.97396158
Pepd	329.766673	1.19198911	0.25337106	0.1272119	0.01764164	0.06220462
Per1	309.031903	1.13076711	0.17730182	0.13108356	0.09680809	0.22779419
Per2	530.53953	1.40259404	0.48809751	0.11295788	2.32E-06	2.78E-05
Per3	1255.94499	1.13790849	0.18638454	0.07275315	0.00635185	0.02682732
Perm1	2.64083213	-1.018405	-0.0263115	0.21543421	0.40714836	0.60646169
Pet100	510.334713	1.02066901	0.02951509	0.09750509	0.73604213	0.85242742
Pet117	55.8486307	-1.0333895	-0.0473841	0.18758783	0.62964124	0.78353451
Pex10	128.149206	1.01822	0.02604932	0.16524867	0.80882903	0.89543686
Pex11a	37.889013	-1.0399047	-0.0564514	0.19554066	0.54814141	0.72500686
Pex11b	200.815914	1.19267826	0.25420491	0.18213943	0.0503298	0.13970201
Pex11g	97.2125444	1.18664231	0.24688512	0.22397347	0.07144441	0.18180723

Pex12	508.427313	1.32871207	0.41002851	0.10477558	1.88E-05	0.00018115
Pex13	297.487525	1.14618301	0.19683742	0.14055717	0.07819407	0.19483475
Pex14	526.411714	1.00750696	0.01078981	0.10671124	0.90821174	0.95230265
Pex16	120.691006	-1.0305686	-0.0434406	0.1534303	0.68860035	0.82154889
Pex26	504.412738	1.08991133	0.12421077	0.10855871	0.18636213	0.36364036
Pex6	155.585734	1.06236149	0.08727476	0.14439056	0.4251805	0.62353316
Pfas	6529.68364	1.00760565	0.01093112	0.06307453	0.85031251	0.91935375
Pfdn1	1470.59921	1.07713798	0.10720307	0.08727248	0.17847258	0.35319538
Pfdn4	4510.94113	1.19705108	0.25948472	0.06255079	1.51E-05	0.00015011
Pfdn5	1450.8446	1.09220195	0.12723964	0.07551963	0.07144556	0.18180723
Pfdn6	1201.56134	1.18799641	0.24853047	0.09467433	0.00373604	0.0174986
Pfkfb1	335.113482	1.42091864	0.50682395	0.11871084	2.80E-06	3.29E-05
Pfkfb3	1108.23677	1.00909983	0.01306891	0.06612019	0.83606106	0.91195011
Pfkfb4	117.68379	1.19523559	0.25729501	0.2038061	0.05822277	0.15627352
Pfkl	7312.12774	-1.30629	-0.3854752	0.05977436	2.72E-11	7.36E-10
Pfkm	1963.99181	1.09315646	0.12849991	0.06649635	0.04168308	0.12135461
Pfkp	705.913654	-1.1068208	-0.1464217	0.0912889	0.07376076	0.18636594
Pfn1	3939.44135	-1.1367354	-0.1848965	0.07003875	0.00499308	0.02211667
Pfn2	629.241092	-1.2088655	-0.2736537	0.13754501	0.01560151	0.05628143
Pgam1	15398.848	-1.3757241	-0.4601911	0.04881875	7.40E-22	5.02E-20
Pgam5	1083.11983	-1.0470414	-0.0663185	0.07002007	0.31540489	0.51446741
Pgap1	330.920812	-1.2797879	-0.3559047	0.12293981	0.00088289	0.00526527
Pgap2	103.17058	1.25473624	0.32738413	0.22002165	0.02600425	0.08400875
Pgd	7823.5692	-1.1827348	-0.2421266	0.04722421	1.42E-07	2.14E-06
Pgghg	413.577844	1.14940017	0.20088117	0.11395163	0.03989798	0.11751969
Pggt1b	509.014942	1.00010631	0.00015336	0.1035666	0.99954955	0.99979674
Pgls	251.766241	1.01609263	0.02303193	0.12684178	0.82326589	0.90419748
Pglyrp2	26.7998772	-1.3635969	-0.4474172	0.51350951	0.01924619	0.06641802
Pgm1	1686.28006	1.06212321	0.08695113	0.07878684	0.23495926	0.42481696
Pgm2	2768.97006	1.03766064	0.0533347	0.05014548	0.27410294	0.46955159
Pgm2l1	279.447549	1.02019663	0.02884724	0.13361681	0.78423653	0.87922895
Pgm3	1267.31556	1.00744889	0.01070665	0.06697682	0.86678528	0.92799996
Pgp	426.148201	1.11295101	0.15439009	0.10958153	0.10295952	0.23845805
Pgpep1	613.353648	1.06234778	0.08725614	0.09953516	0.32254355	0.52182152
Pgrmc1	2137.5833	-1.2306084	-0.2993717	0.08094909	7.23E-05	0.00061065
Pgrmc2	2675.26087	-1.2152386	-0.2812396	0.06787553	1.28E-05	0.00012978
Phactr4	2906.47606	1.12071788	0.16442315	0.05685916	0.00265222	0.01323528
Phax	1008.96055	-1.0722733	-0.1006727	0.07353892	0.14413306	0.30324755
Phb	4675.10793	-1.0622844	-0.0871701	0.1325596	0.40763901	0.60672593
Phb2	3047.22098	-1.1057011	-0.1449615	0.07747145	0.04312109	0.12474732
Phc1	1328.12451	1.17477145	0.23238011	0.07474139	0.00091187	0.00540842
Phc2	1466.53266	-1.2678922	-0.3424321	0.07047773	3.34E-07	4.68E-06
Phc3	1640.68645	1.05769262	0.08092042	0.06462975	0.18876583	0.36698896
Pheta1	65.4828254	-1.1149294	-0.1569524	0.22250487	0.17962996	0.3549375
Phex	7.00152348	-1.0154387	-0.0221031	0.21034455	0.66686939	0.80727622
Phf1	121.677232	1.6473386	0.72013712	0.21562728	6.26E-05	0.00053636
Phf10	2104.09687	-1.0301444	-0.0428465	0.06393313	0.48211454	0.67187634
Phf12	2333.84747	-1.0399549	-0.056521	0.07235188	0.40577421	0.60529078
Phf13	315.81677	1.05916722	0.08293038	0.11060681	0.3832336	0.58445306
Phf14	4257.88035	1.04065196	0.05748764	0.06902844	0.37900997	0.58117281

Phf19	2515.26979	-1.0533264	-0.0749525	0.05863612	0.18317748	0.35945213
Phf2	2344.12456	-1.064651	-0.0903806	0.06304259	0.13276065	0.28523315
Phf20	3289.66913	1.05036042	0.07088445	0.04935864	0.13880862	0.29478422
Phf20l1	2488.4423	-1.3693906	-0.4535341	0.06357313	1.82E-13	6.28E-12
Phf21a	882.980335	-1.0046309	-0.0066655	0.08135606	0.92879492	0.9641278
Phf21b	261.196456	-1.3462461	-0.4289422	0.15938196	0.00112807	0.0064375
Phf23	644.014496	1.15987245	0.21396616	0.08727706	0.00736859	0.03035102
Phf3	7383.10279	-1.1765956	-0.2346185	0.04904316	8.64E-07	1.13E-05
Phf5a	2004.55729	-1.024062	-0.0343031	0.063234	0.56955657	0.73906967
Phf6	2164.36442	-1.176609	-0.2346349	0.07009499	0.00039428	0.00264571
Phf7	26.5155359	-1.0092786	-0.0133245	0.1961318	0.86945049	0.92935896
Phf8	1791.31568	-1.0817777	-0.113404	0.06390179	0.06204548	0.16426711
Phip	4106.13163	-1.0149789	-0.0214498	0.06735372	0.73762201	0.85348047
Phka1	1102.18121	1.16736764	0.22325898	0.09522827	0.00934574	0.03697312
Phka2	605.560547	1.12810043	0.17389551	0.11586027	0.07849317	0.1953181
Phkb	2232.72848	1.12750065	0.17312826	0.06344257	0.0042016	0.0192705
Phkg1	1.62112402	1.01092442	0.01567514	0.21500433	0.51577732	0.6994012
Phkg2	601.352409	1.06960268	0.09707499	0.08817542	0.22652274	0.41512966
Phlda1	418.595683	-1.4028623	-0.4883734	0.13583678	4.48E-05	0.00039735
Phlda2	3.33846261	1.07120325	0.09923225	0.24868595	0.00701178	0.02910592
Phlda3	70.8799342	1.01041066	0.01494176	0.17025586	0.88793957	0.93988646
Phldb1	9485.65581	-1.2031455	-0.2668112	0.05098616	8.17E-08	1.28E-06
Phldb2	6429.19649	1.80050193	0.84839915	0.05317301	2.12E-58	7.99E-56
Phldb3	89.8239877	1.21434797	0.28018188	0.24673892	0.0537587	0.14686477
Phlpp1	947.283566	-1.0380233	-0.0538389	0.08061456	0.47003226	0.66188349
Phlpp2	1447.08089	-1.0940585	-0.1296898	0.06947386	0.04780379	0.13462333
Phospho1	222.631562	1.12341694	0.16789346	0.14464387	0.13528958	0.2894184
Phospho2	464.130531	1.21020128	0.27524702	0.11046766	0.00461857	0.02077385
Phpt1	358.041311	1.03973635	0.05621775	0.11618854	0.56716794	0.7378158
Phrf1	4052.01461	-1.0767392	-0.1066689	0.05376132	0.0400176	0.11776743
Phtf1	1171.1716	-1.0582311	-0.0816547	0.07335111	0.23527274	0.42514372
Phtf2	413.741391	-1.1633996	-0.2183467	0.11154329	0.02376089	0.07865039
Phyh	1102.20436	1.150569	0.2023475	0.07566147	0.00424171	0.0194058
Phykpl	66.3688636	1.50899984	0.59359265	0.33148177	0.00477666	0.02131278
Pi4k2a	464.470571	-1.0486779	-0.0685716	0.09627155	0.42571385	0.62417241
Pi4ka	2396.09454	1.39231886	0.47748964	0.05965592	2.05E-16	9.33E-15
Pi4kb	2534.36687	1.02232907	0.03185966	0.06882458	0.62544419	0.78081474
Pianp	24.0836835	2.26190101	1.17753579	0.4847722	0.00060089	0.0038012
Pias1	1987.1259	1.10256355	0.14086181	0.05916523	0.01306523	0.04868109
Pias2	1908.02753	1.05860005	0.08215763	0.06458701	0.18179916	0.35773382
Pias3	943.303413	1.30159114	0.38027634	0.10115444	3.87E-05	0.0003469
Pias4	540.587761	1.11352032	0.15512788	0.10351601	0.08775273	0.21255787
Pibf1	924.203221	-1.0002268	-0.0003272	0.07570981	0.9954863	0.99813552
Picalm	9741.51303	1.03198664	0.04542429	0.05486086	0.39450549	0.59559402
Pick1	420.215237	-1.0088114	-0.0126564	0.09918308	0.87531166	0.93294904
Pidd1	343.684217	-1.019117	-0.0273196	0.12567284	0.78754216	0.8808535
Piezo1	2163.0826	-1.0254354	-0.0362366	0.06828623	0.57496909	0.74262493
Pif1	535.80935	-1.1603873	-0.2146064	0.1147438	0.02958117	0.0926038
Piga	183.797079	-1.1899388	-0.2508874	0.16777984	0.04506845	0.12898277
Pigb	1494.21179	-1.0305904	-0.0434711	0.05829292	0.43849416	0.63613932

Pigc	288.158608	1.13291172	0.18003544	0.16347664	0.13043213	0.28185486
Pigf	533.593109	-1.0266571	-0.0379545	0.09318604	0.65042394	0.79731393
Pigg	538.849817	-1.0039953	-0.0057525	0.08880116	0.94274022	0.97203013
Pigh	154.755529	1.25517534	0.32788892	0.18864237	0.01796358	0.06311852
Pigk	2002.35116	-1.0131446	-0.0188401	0.06174992	0.74945134	0.86095164
Pigl	147.670105	-1.0441271	-0.0622974	0.14626906	0.56599378	0.7369265
Pigm	216.118943	-1.0347443	-0.0492744	0.12698304	0.6285855	0.78282911
Pign	340.930722	-1.1802509	-0.2390935	0.13238361	0.0283454	0.08952283
Pigo	258.583959	1.2021729	0.2656444	0.14944493	0.02495251	0.0814581
Pigq	2570.77131	1.16980897	0.22627295	0.08529907	0.00390275	0.01811837
Pigs	2221.93395	1.08188297	0.11354444	0.0755709	0.10758785	0.24558095
Pigt	4848.88493	1.42659767	0.51257853	0.05626918	1.26E-20	7.73E-19
Pigu	450.259986	-1.0083511	-0.0119981	0.1004903	0.89130044	0.9423538
Pigv	247.355939	1.01339764	0.01920038	0.12595674	0.85202115	0.92042427
Pigw	378.160984	-1.0108499	-0.0155688	0.11040713	0.86858507	0.92907271
Pigx	952.799403	1.08571411	0.11864427	0.08205843	0.11721096	0.26148944
Pigyl	79.0533292	-1.0141881	-0.0203253	0.1743923	0.84147917	0.91505503
Pih1d1	545.727263	-1.0661149	-0.092363	0.1344193	0.38386848	0.58518869
Pih1d2	236.735323	1.0788173	0.10945056	0.13623018	0.31069585	0.5091485
Pik3ap1	8.32522053	-1.0121984	-0.0174921	0.20815724	0.75506031	0.86390876
Pik3c2a	4558.49565	1.10308588	0.14154512	0.05073951	0.00378824	0.0176844
Pik3c3	806.563643	1.05405217	0.07594627	0.10246589	0.40076783	0.60079999
Pik3ca	1757.96358	1.00695807	0.0100036	0.06910091	0.87919357	0.93525426
Pik3cb	1249.78951	-1.1299466	-0.1762546	0.07716065	0.01404829	0.05178666
Pik3cg	13.0217064	-1.0090953	-0.0130625	0.20954944	0.79371371	0.88527965
Pik3ip1	6.15094005	1.0129718	0.01859401	0.21040422	0.71067431	0.83674589
Pik3r1	2108.44715	-1.2262697	-0.2942763	0.05854762	1.83E-07	2.72E-06
Pik3r2	1063.81708	-1.1743036	-0.2318055	0.09217328	0.00550939	0.02392384
Pik3r4	1929.76491	1.03221883	0.04574885	0.06860944	0.47964169	0.67017998
Pik3r5	21.0077943	-7.8892894	-2.9798954	0.59543133	2.80E-08	4.78E-07
Pikfyve	2189.75229	1.11086487	0.15168333	0.07183114	0.02473957	0.08111437
Pim1	1332.18859	-1.0918187	-0.1267334	0.07423619	0.0678679	0.17556295
Pim2	95.3388462	1.0373712	0.05293222	0.15776699	0.62688055	0.78218883
Pim3	710.117545	-1.2157946	-0.2818996	0.09837598	0.00141355	0.00775153
Pin4	951.736207	-1.0225643	-0.0321916	0.0890389	0.69060447	0.82279048
Pink1	355.733231	1.65752156	0.72902763	0.15922801	4.15E-07	5.69E-06
Pinx1	1610.34146	-1.0353398	-0.0501043	0.07605906	0.48044934	0.6709755
Pip4k2a	1399.41526	1.25297203	0.32535421	0.08062539	1.68E-05	0.0001649
Pip4k2b	951.390988	1.03056722	0.04343862	0.07597443	0.54122753	0.71944113
Pip4k2c	728.798711	1.24715295	0.31863841	0.09511003	0.00024306	0.00175899
Pip4p1	131.876305	1.21662703	0.28288696	0.19945567	0.03955606	0.11660248
Pip4p2	1019.78438	-1.0791969	-0.1099582	0.07782763	0.12816706	0.27838166
Pip5k1a	4208.37459	1.02342224	0.03340149	0.04936622	0.48718503	0.67563876
Pip5k1c	1196.00961	1.33750577	0.41954512	0.08591191	2.06E-07	3.01E-06
Pip5kl1	29.3035158	1.09766818	0.134442	0.23463086	0.18879763	0.36699509
Pir	560.395097	-1.6182062	-0.6943955	0.12095339	8.79E-10	1.89E-08
Pirt	394.403598	1.28912551	0.36639273	0.12940748	0.00103017	0.00598966
Pisd	419.875202	-1.0342783	-0.0486244	0.11054302	0.60728105	0.7678038
Pithd1	501.891731	-1.0681928	-0.095172	0.09197406	0.25093746	0.44234332
Pitpnb	3345.33057	-1.0677581	-0.0945848	0.06817633	0.14254968	0.30068523

Pitpnm1	1740.87487	1.13485098	0.18250287	0.07979962	0.01356388	0.0502177
Pitpnm2	620.648954	1.21087468	0.27604956	0.10261792	0.00259845	0.01301765
Pitrm1	5670.41954	1.05706695	0.08006675	0.04483266	0.06701389	0.17395094
Pitx3	13.7102583	1.01359757	0.01948497	0.20375332	0.77899683	0.87673482
Pja2	5965.24156	1.23648174	0.30624093	0.0517434	1.12E-09	2.34E-08
Pjvk	4.5111601	1.04507514	0.06360667	0.22579366	0.11534593	0.25831842
Pkd1	2167.89799	1.17040146	0.22700347	0.08390293	0.00332945	0.01592744
Pkd1l3	1.65472056	-1.0195558	-0.0279407	0.2178718	0.07117203	0.18129126
Pkd2	711.995253	-1.1043577	-0.1432075	0.0847931	0.06424534	0.16885162
Pkdcc	109.584402	1.04189524	0.05921022	0.1484134	0.58601341	0.74971062
Pkdrej	2.71316824	1.02814645	0.04004578	0.21912653	0.16329301	0.33086861
Pkhd1l1	2.81616314	-1.0400693	-0.0566797	0.22613196	0.00552061	0.0239563
Pkia	832.136401	1.04131405	0.05840524	0.09797193	0.50420949	0.68862602
Pkib	92.798601	1.02194806	0.03132188	0.15286502	0.77233701	0.87322482
Pkig	674.915068	1.45691642	0.54291811	0.10891589	8.45E-08	1.32E-06
Pkmyt1	470.453795	1.26251172	0.33629677	0.10879013	0.0005338	0.00345004
Pkn1	1501.99094	1.04835701	0.06813009	0.07549649	0.33467092	0.534538
Pkn2	9452.23794	1.00857879	0.01232379	0.04492869	0.77964614	0.87680583
Pkn3	747.758623	1.05392318	0.07576972	0.08377428	0.32433225	0.52374204
Pknox1	637.883471	1.02035388	0.0290696	0.08648173	0.71394826	0.83851446
Pkp1	2.86424264	1.01032376	0.01481769	0.21385883	0.6393776	0.79050546
Pkp2	88.6336762	-1.0533853	-0.0750332	0.16980771	0.495978	0.68228932
Pkp3	427.668231	-1.2986126	-0.3769711	0.13343298	0.00097942	0.00573155
Pkp4	2998.98658	-1.0774312	-0.1075957	0.05833067	0.05461019	0.14862045
Pla1a	19.7056868	-1.1332215	-0.1804299	0.2953371	0.06711989	0.17408885
Pla2g12a	1048.6916	-1.2156725	-0.2817546	0.07443049	5.82E-05	0.00050312
Pla2g15	419.013779	-1.0414859	-0.0586434	0.11051889	0.535406	0.71428358
Pla2g4a	2433.24765	1.15050165	0.20226306	0.07352946	0.00336486	0.0160536
Pla2g4c	20.2254602	1.00262497	0.00378207	0.19369157	0.96603277	0.98338206
Pla2g4d	35.2348182	-3.3732937	-1.7541579	0.45525335	4.66E-06	5.19E-05
Pla2g4e	2.92434389	-1.0413858	-0.0585046	0.22691438	0.00431611	0.01962596
Pla2g6	687.411377	1.25313473	0.32554154	0.10976293	0.00084351	0.00506582
Pla2g7	222.631793	1.80804106	0.85442744	0.15816365	4.87E-09	9.36E-08
Plaa	4222.92898	-1.0502538	-0.070738	0.05905969	0.21198693	0.39782488
Plac1	26.1767072	1.03175119	0.04509511	0.20002352	0.59476248	0.75817568
Plac8	785.514572	-2.3744368	-1.2475853	0.12291995	2.13E-25	1.77E-23
Plag1	4.4421704	1.02962191	0.04211467	0.21903643	0.21591875	0.40214004
Plagl2	853.724761	1.03591317	0.05090308	0.07772702	0.48238182	0.67195641
Plat	1918.22717	-1.0513636	-0.0722617	0.06361975	0.23342208	0.42285577
Plau	143.500224	-1.2016734	-0.2650448	0.20458963	0.05253645	0.14428053
Plaur	1373.47451	-1.1660705	-0.221655	0.07632387	0.00186692	0.00984978
Plb1	5.3293705	-1.0348267	-0.0493891	0.21971976	0.23369509	0.42318832
Plbd2	5904.5916	1.19933648	0.26223647	0.06157989	8.81E-06	9.25E-05
Plcb1	774.427116	1.22629867	0.2943104	0.1122043	0.00285092	0.01401466
Plcb3	1107.43273	1.10302261	0.14146236	0.07470898	0.04282903	0.12398641
Plcb4	13.3221441	-1.0129624	-0.0185807	0.20822924	0.74282108	0.85640699
Plcd1	654.528988	-1.0073243	-0.0105282	0.10749508	0.9436573	0.97250604
Plcd3	229.280658	1.03586174	0.05083146	0.12246057	0.61533398	0.77387521
Plce1	143.487901	1.17481669	0.23243566	0.18394167	0.07047566	0.18001858
Plcg1	8972.30442	1.53048145	0.61398555	0.04856769	1.51E-37	2.15E-35

Plch1	45.1964343	1.00105296	0.00151831	0.18697308	0.9887331	0.99471224
Plch2	34.7430702	1.04842482	0.06822341	0.2090136	0.41602823	0.61489808
Plcl1	1.98800485	-1.0054118	-0.0077866	0.21390248	0.77226791	0.87322376
Plcxd2	381.117153	-1.029885	-0.0424833	0.10393981	0.64003074	0.79058554
Plcxd3	15.6054975	1.00983073	0.01411349	0.20285401	0.84147366	0.91505503
Pld1	632.612897	1.15827828	0.21198191	0.09491046	0.01311139	0.04881048
Pld3	1488.86484	1.23529149	0.30485151	0.0856122	0.00012173	0.00096734
Plec	28618.2587	-1.3246688	-0.4056317	0.04403349	6.84E-21	4.25E-19
Plek2	2.92634851	-1.0310897	-0.0441698	0.21906358	0.2268739	0.41553786
Plekha1	1249.26455	-1.0893249	-0.1234343	0.0693797	0.05914422	0.15836903
Plekha2	594.797921	1.04609965	0.06502028	0.09918005	0.46037228	0.65424549
Plekha3	1097.39346	-1.0953338	-0.1313706	0.0751548	0.06107237	0.1623106
Plekha5	1425.15071	1.09230443	0.127375	0.08224702	0.09287082	0.2209239
Plekha6	6.72221059	-1.0295952	-0.0420772	0.21315861	0.45561759	0.64999448
Plekha7	122.925351	-1.044393	-0.0626647	0.16480714	0.5673359	0.73789957
Plekha8	1255.47472	-1.7706351	-0.824267	0.09375422	1.26E-19	7.38E-18
Plekhb1	22.7465908	1.02619426	0.03730386	0.19958132	0.65389044	0.79957394
Plekhb2	1804.0705	-1.3365098	-0.4184705	0.07351025	2.51E-09	5.03E-08
Plekhf2	170.853065	-1.1749185	-0.2325607	0.1699209	0.06225257	0.16459862
Plekhg2	1459.07278	1.43250488	0.51854006	0.10513687	1.12E-07	1.73E-06
Plekhg3	1562.37501	-1.017016	-0.0243423	0.08121045	0.74514628	0.8582271
Plekhg4	24.0847787	1.01683565	0.02408651	0.19811968	0.76715704	0.87116991
Plekhg5	413.389225	1.15254082	0.20481785	0.12412571	0.04901266	0.13717547
Plekhh1	125.094305	1.02450095	0.03492133	0.1524584	0.74853392	0.8604901
Plekhh3	201.460813	1.11191528	0.15304687	0.14028747	0.16524296	0.33403225
Plekhj1	42.081712	2.25217043	1.171316	0.38907743	0.00011878	0.0009498
Plekhm1	1459.42262	-3.495652	-1.8055616	0.07220741	3.69E-139	9.45E-136
Plekhm2	712.046511	1.03930657	0.05562128	0.0953715	0.51671813	0.69992167
Plekhm3	501.433983	1.01338299	0.01917952	0.10513497	0.83547974	0.91187361
Plekhn1	161.165805	1.37316877	0.45750896	0.19568645	0.00259207	0.01300095
Plekho1	267.834783	1.16229434	0.21697546	0.13643713	0.04982618	0.13871916
Plekho2	831.767544	-1.2466824	-0.318094	0.12635153	0.00326663	0.01569011
Plgrkt	274.605677	1.06892478	0.09616034	0.11702519	0.3292214	0.5290837
Plin2	2636.72411	-1.3097562	-0.3892982	0.06114548	4.69E-11	1.23E-09
Plin3	355.569807	1.3424783	0.42489877	0.13796301	0.00035452	0.00241131
Plin4	692.868755	2.3489472	1.23201429	0.11148249	1.51E-29	1.55E-27
Plk1	5644.90537	1.00629269	0.00904998	0.05084527	0.85558173	0.92281737
Plk2	2101.59201	-1.4366592	-0.5227179	0.08148939	2.02E-11	5.55E-10
Plk3	408.957917	-1.2839774	-0.3606198	0.11088787	0.00027217	0.00192943
Plk4	5160.51709	-1.0949747	-0.1308975	0.04896051	0.00597775	0.02548251
Pllp	3.82603114	1.02623385	0.03735952	0.21633827	0.3565046	0.5565792
Plod1	1991.9567	1.13515637	0.18289105	0.06429548	0.00279996	0.01383245
Plod2	4359.62615	-1.1100055	-0.1505668	0.07044989	0.02324319	0.07731634
Plod3	1850.45724	1.10709572	0.14677996	0.08171061	0.05145999	0.14203889
Plp1	48.0121456	1.17878944	0.23730604	0.31245814	0.0671433	0.17411047
Plpbp	2091.49703	-1.2822163	-0.3586396	0.05747386	1.16E-10	2.86E-09
Plpp1	1245.18887	1.13770636	0.18612824	0.08201916	0.01400884	0.05170073
Plpp2	754.619244	-1.2528075	-0.3251648	0.0923884	0.0001263	0.00099932
Plpp3	880.154299	-1.2500123	-0.3219423	0.08579602	5.25E-05	0.0004572
Plpp5	88.4376224	1.46464161	0.55054769	0.24085367	0.00203337	0.01056262

Plpp6	50.4986499	-1.0255706	-0.0364268	0.1768758	0.72204004	0.84343968
Plpp7	162.091421	-1.0433868	-0.0612741	0.13866602	0.565664	0.73677912
Pls3	8749.97522	1.00046429	0.00066968	0.05893195	0.99123173	0.99581018
Plscr3	887.528354	1.3616447	0.4453503	0.09385961	3.75E-07	5.20E-06
Plscr4	184.312324	1.26685365	0.34124987	0.16661488	0.00896767	0.03572446
Pltp	371.386694	1.04655556	0.0656489	0.11605278	0.50293005	0.68781798
Plxdc1	5.40541444	-1.0572245	-0.0802818	0.23446873	0.04694662	0.13263923
Plxna1	4067.43933	-1.0534115	-0.0750691	0.05865652	0.18258237	0.35866894
Plxna2	1071.85255	-1.3195967	-0.4000971	0.09157281	2.59E-06	3.08E-05
Plxna3	470.776399	1.03196559	0.04539487	0.11098894	0.63449022	0.78681701
Plxna4	1424.31865	1.34767059	0.4304679	0.09801042	2.12E-06	2.56E-05
Plxnd1	1135.03822	1.16795048	0.22397911	0.08191631	0.00310303	0.01499992
Pm20d2	84.8098492	-1.0176169	-0.0251945	0.16245147	0.81342299	0.89850379
Pmel	1.67263209	1.00690959	0.00993415	0.21400402	0.72108674	0.84295685
Pmf1	1221.43613	1.0625282	0.08750113	0.06749411	0.17155802	0.34349145
Pml	668.481889	-1.1873269	-0.2477172	0.09964174	0.00545756	0.02373902
Pmm1	606.366542	-1.0164478	-0.0235362	0.09822229	0.7876237	0.88086778
Pmm2	1057.27678	-1.077493	-0.1076785	0.09204011	0.19178132	0.37051699
Pmp22	1418.69511	1.08104865	0.11243146	0.06991452	0.08838741	0.21350985
Pmpca	2375.81632	1.06958472	0.09705075	0.06891851	0.13649827	0.29118116
Pmpcb	4294.87655	1.11402729	0.15578457	0.09136123	0.06513506	0.17049042
Pms1	1193.38759	-1.0840652	-0.1164515	0.08775898	0.14461632	0.30379387
Pmvk	458.113168	1.03942502	0.05578569	0.10757635	0.5513234	0.72771281
Pnisr	1651.0261	1.02013698	0.02876289	0.09554641	0.74128883	0.85589602
Pnkd	567.840898	-1.0840783	-0.1164689	0.09700277	0.17716605	0.35130443
Pnkp	325.309577	1.12848509	0.17438736	0.13363578	0.10656627	0.24425013
Pnn	4294.58681	-1.0915032	-0.1263163	0.06959374	0.05442994	0.1482559
Pnpla2	394.560967	-1.0341159	-0.0483978	0.09732814	0.57642628	0.74377612
Pnpla6	1216.54133	1.13435474	0.18187188	0.11561335	0.06508357	0.17046015
Pnpla7	284.198088	1.33893111	0.42108173	0.15480895	0.00110017	0.0063085
Pnpla8	1713.00148	1.1944988	0.2564054	0.06230783	1.71E-05	0.00016721
Pnpo	852.978055	1.22716431	0.29532844	0.10024232	0.00107335	0.00618518
Pnpt1	3315.20559	-1.0179724	-0.0256984	0.07230778	0.70607057	0.83360434
Pnrc1	438.597502	-1.3102373	-0.3898281	0.1159958	0.00016134	0.00123836
Pnrc2	783.232289	-1.0935583	-0.1290302	0.09392239	0.12624345	0.27535818
Poc1a	450.115234	-1.0761541	-0.1058847	0.11607696	0.27820531	0.47403611
Poc1b	312.066686	-1.0817305	-0.1133411	0.12685967	0.27268325	0.46849148
Poc5	1928.48885	-1.0914364	-0.1262281	0.05994534	0.02785015	0.08830704
Podxl2	2.16693296	-1.00275	-0.003962	0.21337419	0.89365291	0.94436531
Pofut2	2245.31067	1.16913291	0.22543895	0.07195137	0.00087062	0.00520668
Pogk	957.606534	1.41137884	0.49710529	0.08078986	1.17E-10	2.88E-09
Poglut1	678.589135	-1.1355282	-0.1833635	0.09271333	0.02735798	0.08719974
Poglut2	1098.71381	1.0440611	0.06220614	0.06849367	0.33752795	0.53718495
Poglut3	10638.0234	1.54898449	0.6313227	0.07617991	1.34E-17	6.73E-16
Pogz	2291.36592	1.04692501	0.06615811	0.06437034	0.28117007	0.47698571
Pola1	5067.47815	1.10710379	0.14679048	0.05590355	0.00641983	0.02701644
Pola2	1018.67807	-1.084565	-0.1171166	0.07788915	0.10548783	0.24247247
Polb	392.034758	-1.0587591	-0.0823744	0.14288228	0.44727488	0.64330021
Pold1	2855.47224	1.11350952	0.15511389	0.0523425	0.00220027	0.01129676
Pold2	1987.09336	-1.01974	-0.0282014	0.06167438	0.63224763	0.78541342

Pold3	1824.17176	-1.1051377	-0.1442262	0.08512747	0.06364928	0.16749121
Poldip2	2234.90428	1.17841714	0.23685032	0.07464854	0.00071707	0.00440559
Pole	3898.5293	-1.0339281	-0.0481358	0.0522705	0.34156334	0.54117828
Pole3	1644.65153	-1.0669851	-0.09354	0.07514905	0.18274164	0.35881673
Pole4	1747.35726	-2.0132438	-1.0095219	0.06908319	1.82E-49	4.95E-47
Polg	2272.02271	1.04196709	0.05930971	0.08222602	0.43650292	0.63404041
Polg2	248.212716	1.01551716	0.02221461	0.11717205	0.82955267	0.90821835
Polh	1428.24397	1.14186621	0.19139362	0.07503864	0.00643242	0.0270535
Poli	453.5901	1.06057134	0.08484166	0.09818121	0.33150269	0.53138771
Polk	919.160996	-1.2310608	-0.299902	0.07638793	2.96E-05	0.00027242
Poll	719.154853	1.10407331	0.14283597	0.09444237	0.0918937	0.21933264
Polm	148.443488	-1.0821224	-0.1138637	0.15511828	0.31253602	0.51144435
Poln	57.8963447	-1.1019116	-0.1400085	0.20619838	0.22722621	0.41591083
Polq	1327.61454	-1.0174558	-0.0249661	0.0641761	0.68238925	0.81710659
Polr1a	3055.09893	-2.289277	-1.194892	0.05733699	1.23E-97	1.58E-94
Polr1b	1622.56993	-1.2640671	-0.338073	0.08361697	1.47E-05	0.00014724
Polr1c	1053.90919	-1.0116442	-0.0167019	0.08641814	0.83194141	0.9097029
Polr1d	3144.23086	-1.0927431	-0.1279542	0.06420719	0.0361024	0.1085105
Polr1e	854.697503	-1.1905782	-0.2516623	0.08152705	0.00088103	0.00525737
Polr2a	8798.35221	1.03273267	0.04646685	0.05042676	0.34364313	0.54306298
Polr2b	6821.51363	-1.001712	-0.0024678	0.05728683	0.96433864	0.98280897
Polr2c	2663.73041	-1.0127127	-0.018225	0.06899134	0.78038614	0.87706145
Polr2d	560.559932	-1.0289032	-0.0411072	0.09361219	0.6250549	0.78055696
Polr2e	3718.33562	-1.1587637	-0.2125864	0.05199788	2.42E-05	0.00022662
Polr2h	914.711245	-1.0533119	-0.0749327	0.0772292	0.29680186	0.49455164
Polr2i	220.658509	1.04257358	0.0601492	0.12388957	0.55501293	0.73030078
Polr2j	998.425904	1.02562188	0.03649894	0.0739705	0.60002031	0.76265059
Polr2m	6292.06585	-1.0136464	-0.0195545	0.06206915	0.74143684	0.85589602
Polr3b	4064.74501	-1.0861757	-0.1192575	0.05543876	0.02564728	0.08319616
Polr3c	2621.5373	-1.0230484	-0.0328744	0.05385647	0.52772268	0.70883166
Polr3d	2200.76311	-1.0637681	-0.0891837	0.07141782	0.18459154	0.36134133
Polr3e	1693.78598	1.2578469	0.33095634	0.08355926	2.25E-05	0.000212
Polr3f	1479.44454	1.11879755	0.161949	0.07291151	0.01795307	0.06311165
Polr3gl	170.553413	1.07976546	0.11071798	0.16059644	0.33465398	0.534538
Polr3h	1971.33996	-1.0324486	-0.0460699	0.07254397	0.49888397	0.6847427
Polr3k	369.917693	1.34621686	0.42891083	0.1229088	8.69E-05	0.00071845
Polrmt	1188.66929	-1.044141	-0.0623166	0.08941998	0.4425848	0.63975527
Pom121	4008.6786	1.01235619	0.01771698	0.06838894	0.77562753	0.87508221
Pomgnt1	2862.69448	-1.0711143	-0.0991124	0.05860132	0.07820767	0.19483475
Pomgnt2	364.172105	1.15458051	0.20736878	0.11999189	0.04135354	0.12069693
Pomk	296.744357	1.10238558	0.14062893	0.15959254	0.22455039	0.41301653
Pomp	5458.51223	-1.1357789	-0.183682	0.04963272	0.00013713	0.00107176
Pomt1	599.62022	1.049012	0.06903119	0.09000977	0.39782527	0.59799832
Pomt2	560.860209	1.15116115	0.20308981	0.10126004	0.02388525	0.07900079
Pop1	692.423483	-1.0554137	-0.0778086	0.09240249	0.35008885	0.54976168
Pop4	1235.09855	-1.0523141	-0.0735655	0.07394707	0.28880246	0.4853791
Pop5	325.098244	-1.0837978	-0.1160957	0.11325441	0.22845626	0.41735947
Pop7	233.481143	-1.0244958	-0.034914	0.11562913	0.71897306	0.84156113
Por	701.486261	-1.1399115	-0.1889218	0.08168132	0.01217132	0.04607115
Porcn	119.510236	1.05621108	0.07889818	0.1526106	0.47428323	0.66501404

Postn	77.7977534	1.07598257	0.10565471	0.20259985	0.32473246	0.52423729
Pot1a	1171.71311	1.09990459	0.13737838	0.0742906	0.04809904	0.13523633
Pot1b	1272.50849	1.28043419	0.3566331	0.07399325	3.85E-07	5.33E-06
Pou2f1	580.385382	-1.1105926	-0.1513297	0.10520123	0.10082657	0.23457835
Pou2f2	26.8124918	-1.1755399	-0.2333235	0.34480976	0.05162836	0.14235026
Pou2f3	3.57266981	-1.0122182	-0.0175203	0.21449954	0.54052306	0.71878961
Pou5f2	7.94615336	1.05637044	0.07911584	0.23063006	0.13030841	0.28177765
Pou6f1	88.4260162	1.4503376	0.53638876	0.41045304	0.0110581	0.04260278
Ppa1	4788.34865	-1.0826504	-0.1145675	0.05786475	0.03923592	0.11594282
Ppa2	1386.23897	-1.1696675	-0.2260985	0.07010529	0.00063047	0.00395123
Ppan	2086.42369	-1.1630504	-0.2179136	0.07078036	0.00109064	0.00626508
Ppara	189.446728	-1.0001876	-0.0002707	0.13212665	0.99861938	0.99959215
Ppard	1058.44988	-1.1669344	-0.2227235	0.07670378	0.00185381	0.00980079
Pparg	5859.20324	1.91169192	0.93485004	0.05692963	1.05E-61	4.82E-59
Ppargc1b	235.608135	1.05464115	0.0767522	0.11612815	0.42919858	0.62691377
Ppat	3967.64827	-1.416117	-0.5019405	0.06919133	6.09E-14	2.20E-12
Ppcdc	661.005297	-1.2508774	-0.3229404	0.09252993	0.00014079	0.0010977
Ppcs	657.160521	-1.021685	-0.0309505	0.10520535	0.73582976	0.85225852
Ppdpf	214.414641	1.27663435	0.35234537	0.18841749	0.01188379	0.04526997
Ppfia1	4230.01162	1.15030344	0.20201449	0.05323208	9.06E-05	0.0007428
Ppfia2	249.659889	-1.2294701	-0.2980366	0.12457963	0.00511724	0.02252758
Ppfia3	265.144494	1.22177291	0.28897616	0.1403039	0.01212781	0.04592291
Ppfia4	17.1858725	-1.0698172	-0.0973643	0.22421152	0.2500588	0.44140186
Ppfibp1	7630.8163	1.01146326	0.01644391	0.04744265	0.72173512	0.84343968
Ppib	8230.35851	1.18809859	0.24865456	0.05786615	7.89E-06	8.34E-05
Ppic	2358.08871	-1.0145573	-0.0208504	0.06024737	0.71816456	0.84156113
Ppie	931.111204	1.05919479	0.08296793	0.07968642	0.26231182	0.45512859
Ppig	6907.75181	-1.0388747	-0.0550216	0.05956455	0.33381937	0.53372767
Ppil1	375.109655	-1.1636077	-0.2186048	0.11224479	0.02431539	0.08011319
Ppil2	977.51627	-1.0167567	-0.0239745	0.07039023	0.7178893	0.84155927
Ppil3	626.526124	-1.0768324	-0.1067937	0.08736196	0.17998616	0.35547683
Ppil4	1844.55584	-1.1371255	-0.1853915	0.08058604	0.01291576	0.04827288
Ppip5k1	1050.36975	1.02751122	0.03915414	0.08895371	0.6296102	0.78353451
Ppip5k2	10470.3706	-1.0368579	-0.0522182	0.04839771	0.30392535	0.50190586
Ppm1a	2082.58604	1.09801654	0.13489979	0.07355289	0.05040333	0.13987579
Ppm1b	1515.95854	-1.0151966	-0.0217592	0.07660259	0.76040475	0.86717889
Ppm1d	650.106865	-1.0514595	-0.0723933	0.08152162	0.3348025	0.53462165
Ppm1e	717.80374	1.11914289	0.16239425	0.09666151	0.06031628	0.16068496
Ppm1f	3759.73265	-1.0640076	-0.0895085	0.06115354	0.12583675	0.27484549
Ppm1g	9282.12148	-1.1677067	-0.2236779	0.054932	2.44E-05	0.00022801
Ppm1h	2.76632401	1.00123933	0.00178687	0.2126692	0.95991375	0.98105115
Ppm1j	5.41394835	1.0035416	0.00510043	0.20980107	0.91753823	0.95706088
Ppm1k	229.113041	-15.252554	-3.9309789	0.18957958	1.65E-96	1.76E-93
Ppm1l	314.191344	1.13663796	0.18477281	0.12969821	0.08233266	0.20204623
Ppm1m	396.200776	1.05813371	0.08152194	0.10882726	0.38577638	0.58691158
Ppm1n	1.81839494	-1.0069432	-0.0099823	0.21455269	0.65633548	0.80080555
Ppme1	1361.14614	-1.1600084	-0.2141353	0.07036495	0.00124178	0.00693727
Ppox	382.624936	1.08020512	0.11130529	0.11415153	0.2516968	0.44313303
Ppp1ca	5358.76199	1.0075701	0.01088022	0.06280499	0.85815503	0.92418115
Ppp1cb	2992.24876	-1.0203311	-0.0290374	0.05330462	0.57456877	0.74232947

Ppp1cc	5743.46298	-1.1298707	-0.1761577	0.06753519	0.00580337	0.02492162
Ppp1r10	3968.0572	-1.1805761	-0.239491	0.06046247	3.58E-05	0.0003233
Ppp1r11	2670.06794	1.2643923	0.33844416	0.05971527	4.33E-09	8.38E-08
Ppp1r12a	10431.2268	1.13896556	0.18772412	0.06052889	0.00120082	0.00675409
Ppp1r12b	422.780918	1.14596316	0.19656067	0.12940222	0.06551423	0.17123798
Ppp1r12c	128.490342	1.06313823	0.08832919	0.17894638	0.42583563	0.62427951
Ppp1r13b	1067.08259	1.36330543	0.44710881	0.08765449	6.10E-08	9.85E-07
Ppp1r13l	447.707647	1.17659305	0.23461542	0.12006569	0.02166799	0.07316689
Ppp1r15a	1092.77744	-1.4387484	-0.5248143	0.07740491	1.69E-12	5.31E-11
Ppp1r15b	4734.7532	-1.0132675	-0.019015	0.0455794	0.66379704	0.80543405
Ppp1r16a	529.278115	1.10862692	0.14877395	0.11658586	0.13210855	0.28418312
Ppp1r18	3882.64728	-1.0991157	-0.1363432	0.05675232	0.01248887	0.04698455
Ppp1r2	3367.83985	-1.1129016	-0.1543261	0.05499057	0.00359564	0.01695882
Ppp1r21	979.455283	1.17020684	0.22676356	0.08892454	0.00521447	0.02286016
Ppp1r27	5.67747109	-1.006138	-0.0088282	0.21077714	0.83988233	0.9142043
Ppp1r35	29.3511354	-1.0152488	-0.0218334	0.19136141	0.80612709	0.89314029
Ppp1r36	2001.40129	-1.2977948	-0.3760622	0.0904981	7.72E-06	8.19E-05
Ppp1r37	134.785016	-1.0796938	-0.1106222	0.15954294	0.33011511	0.53012984
Ppp1r3b	121.036938	-1.1554028	-0.2083959	0.18029503	0.095189	0.22501773
Ppp1r3f	264.796038	-1.5550396	-0.6369513	0.15962246	6.42E-06	6.90E-05
Ppp1r8	2340.66109	-1.0732806	-0.1020273	0.06022205	0.0767392	0.192148
Ppp1r9b	6037.28567	-1.0736024	-0.1024598	0.05363061	0.04795755	0.13487074
Ppp2cb	3301.57074	1.0952395	0.13124638	0.0554401	0.01408329	0.05190074
Ppp2r1b	5421.55264	-1.0472416	-0.0665943	0.04195827	0.10554955	0.24257083
Ppp2r2a	6949.79039	-1.1537462	-0.2063259	0.05906606	0.0002765	0.00195579
Ppp2r2d	2130.70447	-1.0448254	-0.0632619	0.05959403	0.26789173	0.46230541
Ppp2r3a	2227.47345	1.04988907	0.0702369	0.07511879	0.31819221	0.51687068
Ppp2r5a	616.722492	1.0129226	0.01852394	0.09879555	0.83423251	0.91111932
Ppp2r5b	629.51774	1.3489754	0.43186404	0.11903795	5.03E-05	0.00044044
Ppp2r5e	1384.91387	1.01754262	0.02508922	0.07177541	0.71141532	0.83699764
Ppp3ca	4339.85867	1.08833727	0.12212571	0.05342729	0.0179375	0.06307579
Ppp3cb	4492.97381	1.09211541	0.12712532	0.05380014	0.01444912	0.0529746
Ppp3cc	431.086683	1.26887367	0.34354844	0.10190195	0.00019969	0.00149241
Ppp3r1	3728.57972	1.01158875	0.0166229	0.0600028	0.77354499	0.87386763
Ppp4c	2673.40333	1.08736703	0.12083898	0.06221228	0.04189842	0.12186581
Ppp4r1	6675.3019	1.00833393	0.0119735	0.06098054	0.8362175	0.91196545
Ppp4r2	6009.94162	-1.1512739	-0.2032311	0.0538319	9.37E-05	0.00076482
Ppp4r3a	4424.99269	-1.1617962	-0.2163571	0.06381713	0.00037406	0.00252328
Ppp4r3b	6034.24767	-1.0888101	-0.1227524	0.04953606	0.01071647	0.04159938
Ppp5c	1626.90802	-1.1084643	-0.1485623	0.06066446	0.01040584	0.04054098
Ppp6c	2699.55433	-1.0398072	-0.0563161	0.06145982	0.33753331	0.53718495
Ppp6r1	1749.95055	1.09421523	0.12989654	0.05954946	0.02293068	0.07645549
Ppp6r2	541.910654	1.02390016	0.03407504	0.09144277	0.68126999	0.81646968
Ppp6r3	6196.9499	-1.0097904	-0.0140559	0.05170096	0.77895245	0.87673482
Pprc1	5030.89763	-1.0149688	-0.0214354	0.06238093	0.71874519	0.84156113
Ppt1	522.959851	1.0276824	0.03939448	0.10285283	0.66393385	0.80543405
Ppt2	383.150849	1.08231551	0.11412112	0.11843569	0.25106026	0.44235866
Pptc7	201.156246	-1.0672376	-0.0938814	0.15250286	0.39890217	0.59898451
Ppwd1	1704.89401	-1.0740914	-0.1031168	0.07411742	0.13714635	0.29227163
Pqbp1	2092.2991	-1.1509299	-0.2028	0.07974385	0.00610019	0.02591822

Pqlc2	161.22177	1.19011538	0.25110145	0.26585851	0.07248095	0.18387324
Pqlc3	199.835011	1.33388727	0.41563675	0.15441458	0.0012314	0.00690034
Pradc1	217.555275	-1.9205092	-0.9414889	0.16095676	3.72E-10	8.36E-09
Praf2	126.841873	1.0035105	0.00505572	0.14214317	0.96334759	0.98254201
Prag1	1086.89958	-1.0606184	-0.0849057	0.07333722	0.2167707	0.40302361
Prcc	1919.08512	-1.0984084	-0.1354146	0.07267326	0.04694	0.13263923
Prcp	1013.58092	1.30249618	0.38127914	0.09188352	7.83E-06	8.30E-05
Prdm10	761.239368	1.01812485	0.02591448	0.07529834	0.71389785	0.83851446
Prdm15	515.064364	1.05168824	0.07270709	0.1085972	0.43958896	0.63705768
Prdm16	663.759049	1.2328636	0.3020132	0.11249564	0.00227092	0.0116037
Prdm2	2611.35879	1.01745698	0.0249678	0.06106532	0.67055256	0.80997438
Prdm5	552.587844	-2.6668205	-1.4151207	0.09882959	1.03E-47	2.59E-45
Prdm9	73.5770938	1.37405209	0.4584367	0.31063732	0.01282939	0.04803988
Prdx1	820.884781	-1.7133112	-0.7767872	0.13424159	6.08E-10	1.32E-08
Prdx3	1278.39483	-1.1126657	-0.1540203	0.0721319	0.02271252	0.07592572
Prdx4	3771.85279	1.07049135	0.09827314	0.05755992	0.07561508	0.18989006
Prdx5	798.480138	1.06821839	0.09520662	0.09715796	0.27383318	0.46939624
Preb	881.410726	1.00538446	0.0077473	0.07675676	0.91468602	0.9555461
Prelid1	2518.10766	-1.0659993	-0.0922065	0.05851214	0.10117545	0.23517647
Prelid2	59.6599608	-1.042118	-0.0595186	0.18734618	0.55632992	0.73093193
Prelid3a	18.3054658	1.04388144	0.06195786	0.20995946	0.42838683	0.62608502
Prep	3796.58805	-1.0583787	-0.0818559	0.05774853	0.14485035	0.30418572
Prepl	2836.27608	1.13745194	0.18580559	0.06420724	0.00235306	0.01195663
Prex1	3.61200721	-1.0163901	-0.0234543	0.21463533	0.4837198	0.67301517
Prex2	4132.97353	1.11103803	0.1519082	0.0727304	0.0259513	0.08388498
Prickle1	71.9160377	1.34872637	0.43159769	0.35993546	0.01873129	0.06495611
Prickle3	53.221316	-1.0620855	-0.0868999	0.1988082	0.39516359	0.5958853
Prim1	1153.07116	1.04992942	0.07029235	0.08253566	0.35674729	0.55669787
Primpol	450.76098	1.09321108	0.12857199	0.10496875	0.16153493	0.32819389
Prkaa1	2365.04525	-1.0253732	-0.0361491	0.06742285	0.57110748	0.73980047
Prkab1	1136.47777	1.00334457	0.00481714	0.0744675	0.94567417	0.97301897
Prkab2	557.624041	1.00683968	0.00983399	0.0950701	0.90914887	0.95265416
Prkag1	657.78071	1.10450184	0.14339582	0.09762162	0.09861033	0.23067264
Prkag2	145.24583	-5193.9308	-12.342611	3.17361345	7.94E-25	6.36E-23
Prkar1a	9332.19995	-1.0662612	-0.0925609	0.04466332	0.03387677	0.10340643
Prkar2a	6845.03797	1.09847774	0.13550564	0.04840339	0.00405986	0.01874797
Prkar2b	2542.8539	-1.1214795	-0.1654033	0.05951557	0.00371706	0.01743518
Prkca	1120.02268	1.03905774	0.05527583	0.08397081	0.4752333	0.66620032
Prkcd	2062.69992	1.14205664	0.1916342	0.06827437	0.00308801	0.01494054
Prkce	178.12804	1.16035363	0.21456455	0.15530949	0.07025857	0.17960731
Prkci	5830.08575	1.05709612	0.08010657	0.04867557	0.09092615	0.21783504
Prkcsh	6598.36735	-1.0069304	-0.0099639	0.05379704	0.85449769	0.92224411
Prkcz	28.4276416	-1.1289823	-0.1750229	0.25182428	0.13097655	0.28260226
Prkd1	1561.57183	1.1051572	0.1442516	0.06940522	0.02760859	0.08773654
Prkd2	287.043803	1.05648086	0.07926664	0.13080601	0.44879893	0.64444729
Prkd3	1598.99369	1.10862935	0.14877711	0.06422785	0.01493287	0.05435922
Prkdc	2720.86611	-1.0111277	-0.0159652	0.06120925	0.784933	0.87956967
Prkn	219.777486	1.35017529	0.43314672	0.17651405	0.00210493	0.01086386
Prkra	297.877108	1.31263753	0.39246858	0.13007137	0.00052142	0.00337513
Prkx	2106.25125	-1.1140695	-0.1558393	0.0605757	0.00709073	0.02937529

Prlh	7.07623914	1.08953403	0.12371126	0.26379604	0.02824249	0.08927254
Prmt1	6039.68709	-1.1090847	-0.1493695	0.05566321	0.00530538	0.02321896
Prmt2	619.303442	1.28494256	0.36170387	0.09518575	3.68E-05	0.00033216
Prmt3	2310.48417	1.02478868	0.03532645	0.06276788	0.55650109	0.73108183
Prmt5	5448.0004	-1.1789163	-0.2374613	0.05810517	2.13E-05	0.00020185
Prmt6	136.6992	-1.305112	-0.3841736	0.2279141	0.01359884	0.0503326
Prmt7	1423.53885	1.02472734	0.03524009	0.07050353	0.5970087	0.75976405
Prmt9	795.688617	1.05311478	0.07466268	0.08330935	0.32900738	0.52888748
Prom2	11.3557468	2.41174996	1.27008034	0.88842956	0.00369396	0.0173459
Prorsd1	626.663606	1.02947058	0.04190261	0.10334993	0.62940602	0.78348508
Proser1	955.018014	-1.0724748	-0.1009437	0.0760631	0.15477125	0.31839084
Proser2	518.562291	-1.5866115	-0.6659489	0.09885477	1.70E-12	5.35E-11
Proser3	150.341508	1.10422	0.14302764	0.16343644	0.22157309	0.4095154
Prox2	7.30102303	-1.0556008	-0.0780643	0.23142889	0.09437734	0.22371831
Proz	2.00757896	-1.0188069	-0.0268807	0.21710441	0.18220695	0.35821993
Prpf18	1620.13676	1.10786013	0.14777575	0.06425682	0.01571897	0.05660949
Prpf19	3589.59032	-1.1176972	-0.1605293	0.05767285	0.0037429	0.01752435
Prpf3	3075.12397	-1.0867124	-0.1199702	0.06028081	0.03582494	0.10788703
Prpf31	1619.90895	1.02120439	0.03027165	0.06908077	0.6437595	0.79255735
Prpf38a	2954.03806	-1.0714693	-0.0995905	0.07334776	0.14764987	0.30819363
Prpf39	2268.03589	-1.1217208	-0.1657137	0.08534683	0.03386523	0.10340643
Prpf4	2137.11215	-1.2264748	-0.2945176	0.07743524	5.00E-05	0.00043755
Prpf40a	7994.04753	-1.0400391	-0.0566378	0.0589981	0.31716206	0.51617914
Prpf40b	452.25591	1.30830641	0.38770047	0.1316286	0.00065514	0.00408385
Prpf4b	4156.80517	-1.029361	-0.041749	0.07251034	0.5407338	0.71899523
Prpf6	8639.73037	1.29102045	0.36851186	0.04847892	7.76E-15	3.04E-13
Prps2	5484.51195	-1.0546882	-0.0768166	0.06030705	0.18215602	0.35821593
Prpsap1	1444.00161	-1.0088037	-0.0126454	0.08125262	0.86548874	0.92774752
Prpsap2	471.353115	-1.2857206	-0.3625771	0.12169344	0.00066752	0.00415092
Prr11	6074.51218	1.04207134	0.05945405	0.05881084	0.29336262	0.49066133
Prr12	2175.18451	1.07009625	0.09774056	0.06788242	0.12828706	0.27858233
Prr13	2226.88473	-1.0308821	-0.0438793	0.06368082	0.46986628	0.66179077
Prr14	683.110275	-1.1442811	-0.1944415	0.0992884	0.02769502	0.08797522
Prr14l	2328.2615	-1.0850379	-0.1177454	0.06171038	0.04578517	0.13036408
Prr22	57.9680355	1.11244504	0.15373406	0.23289172	0.17364701	0.34659434
Prr3	426.097693	1.02426119	0.03458366	0.10486088	0.70673273	0.83415689
Prr5l	72.276799	-1.1489382	-0.2003012	0.23006859	0.11899315	0.26403988
Prr7	37.6281898	1.04036534	0.05709024	0.19111057	0.56194199	0.7346884
Prrc1	861.396516	1.36666068	0.45065509	0.09234304	1.90E-07	2.80E-06
Prrc2a	15958.0135	-1.0073208	-0.0105233	0.06291287	0.86196703	0.92618801
Prrc2b	5110.51254	1.03419758	0.04851184	0.05734184	0.37975139	0.58189178
Prrc2c	36414.2926	-1.2061498	-0.2704091	0.06912093	3.92E-05	0.00035088
Prrg1	1408.27679	1.07319314	0.10190974	0.0893372	0.20970801	0.39511098
Prrg2	9.45585765	1.05761713	0.08081745	0.23079285	0.13156725	0.28351855
Prrt1	27.7946516	1.04645357	0.0655083	0.20517577	0.44346899	0.64035915
Prrt2	17.2342369	1.01713942	0.02451745	0.2108304	0.63336925	0.78610759
Prrx1	4302.0755	1.47497613	0.56069161	0.06781019	1.92E-17	9.48E-16
Prss22	11.4824476	-18.583009	-4.2159122	1.02582431	5.64E-06	6.13E-05
Prss23	5.39975475	1.01365935	0.01957291	0.21328263	0.60454704	0.76584726
Prss27	253.304393	1.05200567	0.07314248	0.1315736	0.48575857	0.67467931

Prss35	794.272491	1.00930228	0.01335832	0.0816674	0.85560514	0.92281737
Prss36	4.74795139	1.00156081	0.00225001	0.21099524	0.960126	0.98111152
Prss56	5.24141757	-1.0052562	-0.0075633	0.2126191	0.82781214	0.90766699
Prss8	2.16310191	-1.0004321	-0.0006232	0.21322753	0.98215169	0.99108517
Prtg	401.359666	1.02647067	0.0376924	0.11087436	0.69279251	0.82432399
Prune1	613.151474	1.12655588	0.17191888	0.09250398	0.03953574	0.11660248
Prune2	6773.36361	1.05899787	0.08269969	0.05342575	0.10968116	0.24938155
Prx	12.8135379	-1.023383	-0.0333461	0.20891975	0.58753565	0.75109711
Prxl2c	248.495977	1.33423072	0.41600816	0.17596696	0.00294251	0.0143704
Psap	5252.77865	1.07191772	0.10019417	0.06894269	0.12395369	0.27212456
Psat1	3901.16777	1.11407991	0.15585272	0.06672723	0.01382079	0.05108032
Psca	2.07552704	1.00487243	0.00701236	0.21352359	0.8148318	0.89951701
Psd	55.700165	1.3025472	0.38133565	0.37068585	0.02708778	0.08655387
Psd3	3389.58714	1.26654861	0.34090245	0.06891836	2.19E-07	3.18E-06
Psen1	2624.99858	-1.0698326	-0.0973851	0.05696487	0.0751778	0.18908849
Psen2	166.954816	-1.2310607	-0.2999019	0.22245047	0.03741201	0.11173884
Psenen	62.1823452	1.07486709	0.10415828	0.18901641	0.34959097	0.54921025
Psip1	12938.8364	-1.0113952	-0.0163468	0.06231215	0.78231544	0.87827816
Pskh1	714.959368	-1.1317486	-0.1785535	0.09054189	0.02925694	0.09181183
Psma1	3084.67806	-1.0227576	-0.0324642	0.05792315	0.56009403	0.73324523
Psma2	9438.09434	-1.0204896	-0.0292615	0.07282561	0.67131672	0.81028617
Psma3	5960.69139	-1.1296569	-0.1758847	0.05934312	0.00199345	0.01039393
Psma4	4869.01665	-1.0672735	-0.09393	0.05879525	0.0960948	0.22644855
Psma5	4905.82653	-1.1305969	-0.1770847	0.05022369	0.00027861	0.00196641
Psma7	10749.8833	1.25485607	0.3275219	0.04352538	1.09E-14	4.17E-13
Psma8	17.83334	-5.7871371	-2.5328498	0.63909945	3.16E-06	3.66E-05
Psmb1	4552.62327	-1.0047524	-0.00684	0.06164199	0.90893882	0.95251262
Psmb10	147.786585	-1.0325726	-0.0462432	0.14101099	0.665572	0.8065116
Psmb2	2765.71721	-1.0881875	-0.1219272	0.05601934	0.02374494	0.07861792
Psmb4	3036.00815	-1.0338047	-0.0479636	0.08386972	0.53350361	0.71285921
Psmb5	3686.71277	1.07759453	0.10781443	0.05486694	0.04179025	0.12163896
Psmb7	4969.7237	-1.0815079	-0.1130442	0.06206242	0.0561656	0.15188544
Psmb8	334.395407	-1.169105	-0.2254045	0.13188565	0.03716493	0.11110448
Psmb9	190.978498	1.01170511	0.01678884	0.13868116	0.87555831	0.93310332
Psmc1	6423.85946	-1.0558414	-0.0783931	0.05273978	0.12394895	0.27212456
Psmc2	3663.92992	-1.0771067	-0.1071612	0.04478987	0.01427602	0.05243
Psmc3	6451.4963	-1.0353842	-0.0501662	0.03916877	0.19241086	0.37131413
Psmc3ip	492.89928	1.06484938	0.09064938	0.10327891	0.31622616	0.51517959
Psmc4	5719.48604	-1.0204363	-0.0291861	0.05053637	0.55190323	0.72795309
Psmc5	4922.48527	-1.113704	-0.1553658	0.06885978	0.01629171	0.05807876
Psmc6	5004.69705	-1.1938496	-0.2556211	0.06205045	1.67E-05	0.00016428
Psmd1	11162.7138	-1.0553789	-0.0777611	0.04559749	0.07918829	0.19651338
Psmd11	8942.69911	-1.0647506	-0.0905155	0.05321863	0.07879303	0.19568412
Psmd12	6531.21895	-1.0570864	-0.0800932	0.05488025	0.13071697	0.28232749
Psmd13	4277.98143	-1.0184874	-0.0264282	0.0483135	0.58894524	0.75240733
Psmd14	3859.52254	-1.1849755	-0.2448573	0.06000545	2.11E-05	0.00020028
Psmd2	10828.1573	1.04066167	0.05750112	0.03761106	0.12057357	0.26648507
Psmd3	11673.2761	-1.1201838	-0.1637354	0.04423847	0.00015348	0.00118509
Psmd4	3389.31075	-1.048842	-0.0687973	0.05240915	0.17561278	0.34915407
Psmd5	1862.20466	-1.0821914	-0.1139557	0.0604998	0.04872039	0.13662612

Psmd6	3665.12495	-1.0395184	-0.0559153	0.06087141	0.337189	0.53697237
Psmd7	4449.53123	-1.2048491	-0.2688525	0.05419884	2.88E-07	4.08E-06
Psmd8	2743.29415	-1.0499324	-0.0702965	0.05387472	0.17718797	0.35130443
Psmd9	1616.28383	-1.0296823	-0.0421993	0.06501603	0.49535971	0.68187813
Psme4	6358.6056	1.11957499	0.16295117	0.05244644	0.00131708	0.00729118
Psmf1	1666.21368	-1.0413159	-0.0584078	0.06631688	0.35339337	0.5530739
Psmg1	1274.08488	1.05257457	0.07392245	0.08801727	0.35674195	0.55669787
Psmg2	209.267535	-1.1214384	-0.1653503	0.14708999	0.14433307	0.30349747
Psmg3	612.077812	-1.0186278	-0.026627	0.1050862	0.7798705	0.8768666
Psmg4	468.048721	1.0668144	0.0933092	0.12039094	0.35299143	0.55265464
Psors1c2	5.54114421	-1.0329688	-0.0467967	0.21729453	0.32327056	0.5226017
Pspc1	1630.7136	1.03386501	0.04804783	0.06511672	0.4389614	0.63652881
Psph	2016.20238	1.1600431	0.2141784	0.07377349	0.00197877	0.01035037
Psrc1	583.677714	1.09976576	0.13719628	0.10286646	0.129639	0.28056691
Pstk	167.3083	1.14800421	0.19912793	0.1567537	0.09228673	0.21998381
Pstpip1	10.5582675	-1.0875641	-0.1211005	0.25531346	0.06889566	0.17744994
Ptafr	2.03369317	-1.0087079	-0.0125084	0.21362399	0.6901742	0.82279048
Ptar1	986.470283	1.0089513	0.01285654	0.07634864	0.85786431	0.92418115
Ptbp2	408.559128	1.02995493	0.04258121	0.11544723	0.6634693	0.80543405
Ptcd1	520.430911	-1.0496601	-0.0699222	0.09938711	0.42636858	0.62465442
Ptcd2	706.466229	-1.1367704	-0.1849408	0.08424883	0.01681867	0.05964762
Ptcd3	3791.98147	-2.0833583	-1.058911	0.09574763	1.32E-29	1.36E-27
Ptch1	1529.81661	-1.1102427	-0.1508751	0.07154458	0.02487007	0.08135485
Ptchd3	22.7874349	-1.1429415	-0.1927516	0.31626711	0.04883202	0.13684931
Pter	378.142972	1.09836203	0.13535365	0.10758996	0.14713755	0.30747668
Ptger1	3.91257007	1.00686986	0.00987722	0.2130734	0.7711305	0.87278509
Ptger4	158.784772	-1.3586551	-0.4421793	0.22809539	0.00641451	0.02700292
Ptges	3.1026942	-1.0017269	-0.0024892	0.2132518	0.93276814	0.96688223
Ptges2	407.644379	1.00049026	0.00070712	0.10076029	0.9943139	0.99742843
Ptges3	17669.6256	-1.119615	-0.1630027	0.06284135	0.00644292	0.02708691
Ptges3l	55.2831101	1.44330534	0.52937654	0.30749707	0.00686592	0.02857453
Ptgfrn	11063.3208	-1.163331	-0.2182617	0.05519503	4.15E-05	0.00037023
Ptgr1	3797.93314	-1.4352451	-0.5212972	0.05461153	2.02E-22	1.39E-20
Ptgr2	560.661194	1.00551455	0.00793396	0.09947253	0.92919252	0.96434871
Ptgs2	798.574905	-1.8316495	-0.8731435	0.11101601	3.09E-16	1.38E-14
Pthlh	63.4873615	-8.4281089	-3.075209	0.35339949	2.83E-19	1.63E-17
Ptk2	4175.55654	-1.0499897	-0.0703752	0.0486156	0.13673885	0.29159724
Ptk2b	18.7389701	-7.9922241	-2.998597	0.65894146	2.22E-07	3.22E-06
Ptk7	7.59684048	1.05934629	0.08317427	0.23045915	0.15547194	0.31957567
Ptms	1548.37716	1.02893337	0.04114956	0.10480449	0.65443006	0.79983482
Ptov1	596.368872	1.10672975	0.14630297	0.13025807	0.16656975	0.33565495
Ptp4a2	17235.1685	1.00908179	0.01304311	0.04870007	0.7824286	0.87827816
Ptpa	1207.61469	1.09426138	0.12995739	0.09618733	0.13038987	0.28185486
Ptpdc1	1025.60708	-1.2463731	-0.317736	0.086333	7.14E-05	0.00060338
Ptpmt1	993.666915	-1.1091021	-0.1493922	0.08153292	0.04692107	0.13263923
Ptpn1	1276.12272	1.19009943	0.25108212	0.0812044	0.0008727	0.00521663
Ptpn11	13296.8746	1.08893673	0.12292013	0.04355251	0.00390372	0.01811837
Ptpn12	2414.92428	1.12434728	0.16908772	0.06440484	0.00580869	0.02493611
Ptpn13	2585.91334	-1.0739861	-0.1029753	0.05344206	0.04633674	0.13143791
Ptpn14	3035.73412	-1.1194567	-0.1627987	0.06199626	0.0059279	0.02533747

Ptpn18	25.0651999	-1.0547726	-0.0769321	0.22016202	0.28956047	0.48595174
Ptpn2	683.676089	-1.0886724	-0.1225699	0.08511806	0.11498	0.25790472
Ptpn21	2022.41671	-1.0239821	-0.0341905	0.07063742	0.60717469	0.7678038
Ptpn23	1986.86162	1.08642325	0.11958625	0.06973324	0.0695207	0.1785169
Ptpn4	591.587858	-1.1795022	-0.238178	0.09626927	0.00599076	0.02552899
Ptpn6	2.97895447	1.01389092	0.01990245	0.21439575	0.54170006	0.71960777
Ptpn9	1526.80906	1.10890629	0.14913745	0.0681926	0.02070261	0.0705508
Ptpra	2388.15952	1.21858638	0.28520852	0.06822736	1.11E-05	0.00011419
Ptprd	1.83402877	-1.0309916	-0.0440326	0.22166679	0.02318391	0.07719049
Ptpre	5.42216103	1.01592341	0.02279164	0.20966592	0.6751982	0.81263946
Ptprg	1860.3627	1.00698164	0.01003738	0.06918798	0.88024241	0.93575978
Ptprj	710.162217	1.00952149	0.01367163	0.09367022	0.87221881	0.93093268
Ptprk	577.997466	1.03835309	0.05429711	0.089169	0.50396622	0.68848268
Ptprm	3372.68321	1.79107079	0.84082236	0.06691868	3.05E-37	4.20E-35
Ptprq	101.565735	-2.6159349	-1.3873266	0.76992034	0.00184936	0.00978132
Ptprr	42.2472785	-6.6984226	-2.7438214	0.42924121	1.02E-11	2.90E-10
Ptprs	5450.45917	1.23604196	0.30572772	0.06964018	3.86E-06	4.37E-05
Ptpru	89.7074928	1.0578153	0.08108775	0.17233505	0.46563985	0.65873512
Ptprz1	1763.23199	-1.0647862	-0.0905638	0.06299312	0.13177242	0.28374595
Ptrh1	100.227154	1.41587518	0.50169408	0.23808198	0.00358754	0.01692684
Ptrh2	1099.95978	-1.1079585	-0.1479038	0.09576182	0.08403922	0.20536863
Ptrhd1	280.77971	-1.0442813	-0.0625104	0.13565798	0.55539268	0.73044972
Pts	606.462292	1.38106085	0.46577689	0.14437132	0.00018914	0.00142862
Pttg1	2436.50137	-1.0253799	-0.0361586	0.08303858	0.63623973	0.78819209
Pttg1ip	302.23076	1.07357239	0.10241948	0.15222174	0.3631891	0.56391848
Ptx3	507.992768	1.32970782	0.41110927	0.12624668	0.00021326	0.00158183
Ptx4	2.28652061	1.00380514	0.00547924	0.21337152	0.85914325	0.92464333
Puf60	2135.16394	1.03545141	0.05025985	0.05812768	0.36925164	0.57058081
Pum1	5019.15778	-1.0320151	-0.045464	0.04880792	0.33784624	0.53741592
Pum2	8539.39943	1.00302107	0.00435192	0.05446431	0.93470607	0.96834491
Pum3	4183.15168	-1.0526537	-0.074031	0.08263214	0.33056368	0.5306417
Pura	297.037669	-1.1079419	-0.1478822	0.12030591	0.14179622	0.29954673
Purb	357.153513	-1.2118156	-0.2771702	0.13920165	0.01496114	0.05441575
Purg	214.56631	1.00451025	0.00649228	0.13602516	0.9523901	0.97694725
Pus1	1194.37626	1.01198342	0.01718566	0.06620902	0.78892725	0.8818637
Pus10	567.596978	-1.2955268	-0.3735388	0.10162713	5.57E-05	0.00048284
Pus3	989.508335	1.02406164	0.03430256	0.08905911	0.67090873	0.81004995
Pus7	4113.93403	-1.0818322	-0.1134767	0.05693613	0.03831892	0.11375791
Pus7l	1032.61205	-1.0094944	-0.0136329	0.07936072	0.8524805	0.92070514
Pusl1	246.664951	1.14743165	0.19840822	0.13199417	0.06620738	0.17245152
Pwp1	2434.69141	-1.080224	-0.1113306	0.07479376	0.11146963	0.25237292
Pwp2	1489.48082	-1.2414585	-0.312036	0.10064174	0.00059008	0.00373837
Pwwp2a	326.174555	-1.0111845	-0.0160462	0.12237256	0.87322372	0.93185571
Pwwp2b	355.105991	1.33958638	0.42178761	0.1148098	4.45E-05	0.00039523
Pwwp3a	1852.59828	1.10933335	0.14969295	0.06414118	0.01423642	0.05229955
Pwwp3b	3374.95485	1.35910397	0.44265582	0.07964892	5.23E-09	9.99E-08
Pxdc1	30.9869462	-1.036	-0.051024	0.20225242	0.54150194	0.71951894
Pxdn	5652.23817	1.0341789	0.04848578	0.06013921	0.39916537	0.59923924
Pxk	3565.52266	-1.0281754	-0.0400864	0.06069412	0.49032349	0.67756676
Pxmp2	47.5233272	1.03949619	0.05588447	0.20040497	0.53016285	0.71061904

Pxmp4	406.919567	1.08640759	0.11956546	0.11074552	0.20937903	0.3946729
Pxn	1650.60053	-1.2956854	-0.3737154	0.0687031	1.32E-08	2.37E-07
Pxylp1	826.738315	1.24953816	0.32139496	0.10678285	0.0007604	0.00463844
Pycard	170.931949	1.32150323	0.40217995	0.19072205	0.00558265	0.02418456
Pycr1	320.402716	2.19146012	1.13189242	0.12878177	9.05E-20	5.39E-18
Pycr2	610.218956	-1.0020069	-0.0028925	0.09113239	0.9711476	0.98673136
Pycrl	246.585101	-1.0119096	-0.0170804	0.12384098	0.86526768	0.92769325
Pygb	3605.45796	-1.0770447	-0.1070781	0.0564772	0.04894901	0.13705724
Pygl	611.042995	-1.0069718	-0.0100232	0.09975394	0.90888572	0.95251262
Pygm	35.8307717	-1.0186197	-0.0266155	0.19060152	0.76980999	0.87206102
Pygo1	101.551133	1.35007979	0.43304467	0.23865497	0.00876758	0.03510871
Pygo2	372.853766	-1.0598543	-0.0838659	0.11845141	0.39674684	0.59714804
Pyroxd1	949.374419	1.06629579	0.09260769	0.07438143	0.18386301	0.36041089
Pyroxd2	3.76959041	-1.0427911	-0.0604502	0.22535502	0.0826015	0.20262833
Pyurf	556.968899	-1.6358741	-0.7100617	0.11613856	9.05E-11	2.27E-09
QRSL1	864.336681	-1.0533085	-0.074928	0.07470766	0.28365726	0.47981638
Qars	3074.32575	1.02452827	0.03495979	0.05635879	0.5205463	0.70309668
Qdpr	921.391085	-1.0453912	-0.064043	0.08603301	0.4155374	0.61451349
Qk	8055.10533	-1.1263879	-0.1717037	0.05065529	0.00044683	0.00294744
Qpctl	322.470924	1.17634006	0.23430518	0.12584822	0.02669578	0.08568602
Qrich1	3940.24661	-1.0337511	-0.0478888	0.0553227	0.36967279	0.57107809
Qser1	10752.4005	-1.2235306	-0.2910502	0.04618139	1.11E-10	2.76E-09
Qsox1	1189.14973	1.18660965	0.24684542	0.09674738	0.00460149	0.02071883
Qsox2	2474.38794	1.15963277	0.21366801	0.06647201	0.00071636	0.00440335
Qtrt1	576.074505	-1.0054197	-0.0077979	0.09106975	0.92389408	0.96109284
Qtrt2	704.432023	-1.1494843	-0.2009868	0.09859559	0.02238611	0.07510897
R3hcc1	784.503335	-1.0051112	-0.0073552	0.08411758	0.92691761	0.9629239
R3hcc1l	850.344715	-1.0783235	-0.10879	0.08676333	0.16964677	0.34062981
R3hdm1	3626.32513	1.10926359	0.14960223	0.07222984	0.02741348	0.08731146
R3hdm2	4519.64456	1.33952908	0.4217259	0.06150165	1.60E-12	5.04E-11
R3hdm4	1199.1281	1.04953287	0.06974735	0.07678124	0.33030493	0.53035926
RAD51	1055.76847	1.0671967	0.09382611	0.07312242	0.17190998	0.34387365
RAD51C	519.569179	1.06124959	0.085764	0.10070287	0.33585295	0.53559066
RARS	3157.87491	-1.0622964	-0.0871864	0.05594278	0.10584676	0.2431666
RCAN1	2468.01353	-1.3063673	-0.3855606	0.07660408	1.13E-07	1.73E-06
RNF168	1458.08129	1.04102928	0.05801065	0.08949245	0.47677501	0.66723814
RNaseP_nuc	52360.2787	-1.0998818	-0.1373485	0.11706665	0.14991231	0.31130567
RNase_MRP	82698.8473	1.11878207	0.16192904	0.16978718	0.17630241	0.35009051
RPS14	5170.40304	1.02358524	0.03363125	0.08117192	0.65829237	0.80174227
Rab10	8262.54424	-1.0107348	-0.0154045	0.04757253	0.74773121	0.86005538
Rab11a	2064.20499	1.06616159	0.09242612	0.05868235	0.1012076	0.23517933
Rab11b	1217.01487	1.14484439	0.19515151	0.09198568	0.01895623	0.06561179
Rab11fip2	587.132933	1.24364877	0.3145791	0.09395417	0.00025054	0.0018051
Rab11fip3	1584.35943	1.00056726	0.00081816	0.07427738	0.99130144	0.99581018
Rab11fip5	567.939959	-1.4111075	-0.4968279	0.13319064	2.47E-05	0.00023094
Rab12	2952.7241	1.07261537	0.10113283	0.05761761	0.06807993	0.1759338
Rab13	1212.66163	-1.1506331	-0.2024278	0.08697536	0.01095038	0.04225132
Rab14	3793.4985	1.00603574	0.00868156	0.05318205	0.8670702	0.92822742
Rab18	4725.95514	1.00672292	0.00966667	0.05204937	0.84923423	0.91888593
Rab1b	1727.5588	1.09211588	0.12712595	0.06241504	0.03277286	0.1007005

Rab20	46.5405755	-1.0462031	-0.065163	0.18930038	0.51930919	0.70216917
Rab21	1007.62844	-1.0674099	-0.0941143	0.07541992	0.18130756	0.35720546
Rab22a	799.204391	1.36283111	0.44660679	0.11358366	1.54E-05	0.00015272
Rab23	3590.86628	-1.0544758	-0.076526	0.05677352	0.16126685	0.32780519
Rab24	980.991586	1.1128501	0.15425928	0.08154267	0.0403485	0.11846533
Rab27a	17.4656582	-1.8369177	-0.877287	0.77920396	0.00648111	0.02722064
Rab27b	31.839708	-107.33009	-6.7459108	1.02144545	1.42E-11	3.93E-10
Rab29	297.255779	1.11177109	0.15285978	0.11871067	0.1265584	0.27581033
Rab2a	1819.44529	1.12968125	0.17591576	0.08873104	0.02950649	0.09246039
Rab30	111.179058	1.01518597	0.02174404	0.15200132	0.84160018	0.91510893
Rab31	740.180858	-1.2874114	-0.3644731	0.09160557	1.68E-05	0.0001649
Rab32	1072.92713	-1.2024753	-0.2660073	0.08203054	0.00047427	0.00310565
Rab33b	1192.18255	1.08453715	0.11707947	0.06864935	0.07121545	0.18136579
Rab34	2105.36175	1.13418751	0.18165917	0.10039477	0.0412724	0.12054252
Rab35	469.037062	-1.0228512	-0.0325962	0.10810715	0.72467506	0.84468495
Rab39b	3.36918789	-1.0032059	-0.0046178	0.21290561	0.88667975	0.93909612
Rab3a	69.5474821	1.14592683	0.19651493	0.23504334	0.12133935	0.26776177
Rab3d	657.8477	1.12601311	0.17122363	0.09246073	0.04051728	0.11882471
Rab3gap1	2540.93991	1.11085928	0.15167608	0.05416914	0.00374774	0.01753415
Rab3gap2	2261.45308	1.16294852	0.21778724	0.06216854	0.00024786	0.00178876
Rab3il1	49.9027024	2.03619413	1.02587511	0.31312555	5.45E-05	0.0004725
Rab3ip	1989.83949	-1.1352133	-0.1829634	0.05558831	0.00062991	0.00395008
Rab40c	131.085711	1.2175965	0.28403612	0.18652365	0.03427539	0.104363
Rab42	10.471723	1.05710629	0.08012045	0.22629737	0.20938306	0.3946729
Rab43	877.080501	1.21137575	0.27664644	0.09133147	0.00091517	0.00541996
Rab4a	676.189196	1.38648091	0.47142775	0.0955337	1.32E-07	2.01E-06
Rab4b	272.8387	1.07828932	0.10874433	0.13230167	0.3045808	0.50259952
Rab5b	644.584657	1.15588457	0.20899733	0.09924286	0.0180979	0.0634293
Rab5c	6758.02033	1.05597482	0.07857543	0.05717444	0.15363063	0.31655274
Rab5if	3292.27723	-1.1113211	-0.1522757	0.04723752	0.00092294	0.0054609
Rab6a	2517.45532	1.07704356	0.1070766	0.06921332	0.10171757	0.23613722
Rab7	10212.7972	1.01657321	0.02371412	0.0453633	0.59104989	0.75419355
Rab7b	672.455035	1.07009207	0.09773492	0.09071378	0.23467776	0.42454769
Rab8a	1498.73349	-1.0274188	-0.0390244	0.07369964	0.5725684	0.74125145
Rab8b	1976.80006	-1.0479158	-0.0675228	0.05821524	0.22731138	0.41591083
Rab9	361.720375	-1.1125141	-0.1538237	0.12058763	0.12745167	0.27737952
Rabac1	224.62185	1.12106629	0.16487159	0.15954844	0.15949789	0.32567047
Rabep1	2117.49761	1.01673425	0.02394264	0.0787458	0.74520375	0.8582271
Rabep2	330.775108	1.06013555	0.08424875	0.09949388	0.33984482	0.53925581
Rabgap1	3658.44842	1.08231599	0.11412177	0.05676272	0.03682936	0.11028148
Rabgap1l	1016.56565	1.37631135	0.46080687	0.09651557	3.13E-07	4.40E-06
Rabggta	698.713478	-1.0140405	-0.0201153	0.09969121	0.81901603	0.90242519
Rabggtb	3727.60578	1.07541411	0.1048923	0.07184019	0.12040438	0.26628282
Rabif	117.005515	-1.0400973	-0.0567186	0.16120295	0.60407027	0.76556019
Rabl2	406.880815	-1.0428214	-0.0604921	0.10750377	0.51545797	0.69910181
Rabl3	752.4896	-1.1349242	-0.182596	0.10465141	0.04644013	0.13167286
Rabl6	1469.9234	-1.0172771	-0.0247127	0.0761577	0.72799745	0.84701611
Rac1	8407.69543	-1.1622886	-0.2169683	0.07314835	0.00153614	0.0083159
Rac3	19.6388053	1.10860307	0.1487429	0.26960439	0.08525571	0.20762844
Racgap1	3955.83085	1.14837294	0.19959124	0.05219384	7.79E-05	0.00065408

Rack1	13541.7283	-1.1295973	-0.1758085	0.05443125	0.0008147	0.00491352
Rad1	451.084004	1.03515174	0.04984226	0.11084548	0.60130691	0.76340352
Rad17	1549.99178	-1.0117583	-0.0168646	0.0673529	0.79052614	0.88303452
Rad21	8188.27604	1.04698891	0.06624617	0.04306989	0.11599786	0.2594156
Rad23a	768.960855	1.01917198	0.02739751	0.09419429	0.74698974	0.8595626
Rad23b	13336.5956	-1.1215253	-0.1654622	0.04661421	0.00026946	0.00191342
Rad50	1954.57985	1.00846921	0.01216703	0.06869552	0.85256865	0.92070514
Rad51ap1	656.403976	1.00202744	0.00292201	0.08523657	0.97108884	0.98673136
Rad51b	248.099298	1.29374482	0.37155309	0.1440238	0.00209158	0.01080805
Rad51d	1605.47395	1.33512623	0.41697615	0.06992236	5.17E-10	1.14E-08
Rad52	428.956761	-1.2689409	-0.3436248	0.10236499	0.00021015	0.00155968
Rad54b	569.151578	-1.0265032	-0.0377381	0.0959531	0.65966791	0.80280695
Rad54l	1878.46374	1.04421634	0.06242065	0.06856357	0.33673068	0.5365742
Rad54l2	1710.15171	-1.0229944	-0.0327982	0.06558615	0.59885837	0.76173923
Rad9a	1354.31848	-1.0408859	-0.057812	0.07892258	0.42977693	0.62761543
Rad9b	2.07689242	-1.0061591	-0.0088585	0.21421609	0.71988251	0.84225325
Radx	22.753454	1.04398769	0.0621047	0.21705225	0.34248688	0.54197121
Rae1	2641.40508	1.31457854	0.39460034	0.05928938	6.52E-12	1.89E-10
Raf1	1853.81645	1.02447621	0.03488648	0.06632487	0.58042288	0.74680766
Rai1	489.663254	1.08955212	0.12373521	0.10268082	0.17038085	0.34161507
Rai14	5163.52437	1.11564215	0.15787435	0.06534983	0.01104069	0.04256133
Ralb	1934.30304	-1.1475705	-0.1985828	0.06807531	0.0020309	0.01055411
Ralbp1	7324.07806	-1.0080697	-0.0115954	0.0630159	0.84764018	0.91809172
Ralgapa1	3176.67401	1.20886281	0.27365052	0.08315293	0.00038846	0.00261243
Ralgapa2	1971.22264	-1.2419414	-0.3125971	0.08334536	5.62E-05	0.00048582
Ralgapb	5047.51219	1.52330306	0.607203	0.04465865	5.37E-43	9.97E-41
Ralgds	420.481208	1.20596322	0.27018591	0.12314935	0.00994121	0.03906346
Raly	3936.97662	-1.0192084	-0.027449	0.0481717	0.56045891	0.73342309
Ran	17751.7115	-1.0950806	-0.1310371	0.06087559	0.02525168	0.08226705
Ranbp1	7349.88682	-1.1725045	-0.2295935	0.06018969	6.88E-05	0.00058441
Ranbp10	1453.70487	-1.0500656	-0.0704795	0.06451413	0.25126795	0.44256048
Ranbp17	462.006652	1.12006876	0.1635873	0.10136055	0.06760294	0.1751251
Ranbp2	16894.951	-1.0479193	-0.0675276	0.05513459	0.20305535	0.38621219
Ranbp3	1765.77736	-1.0546408	-0.0767517	0.06190361	0.19373495	0.37330697
Ranbp3l	3.06286661	-1.0170344	-0.0243684	0.21435184	0.5007738	0.6861316
Ranbp6	1493.65122	-1.158907	-0.2127648	0.06901758	0.00110262	0.00631973
Ranbp9	2396.62521	1.0799153	0.11091817	0.05864808	0.04890866	0.13697417
Rangap1	5381.79237	-1.1954869	-0.2575983	0.05656976	2.28E-06	2.74E-05
Rap1a	1884.98721	-1.047841	-0.0674199	0.0834812	0.37851584	0.5804846
Rap1b	8005.10879	-1.0731381	-0.1018358	0.0432616	0.01575069	0.05667592
Rap1gap2	449.477721	1.06972042	0.09723379	0.10069382	0.27553844	0.47105932
Rap1gds1	4887.96485	1.39600666	0.48130582	0.05117864	9.00E-22	5.97E-20
Rap2a	244.259773	-1.2017983	-0.2651948	0.14907842	0.02490301	0.0813952
Rap2b	279.727421	-1.5040736	-0.5888752	0.15207412	1.18E-05	0.00012042
Rap2c	1503.27311	-1.1371174	-0.1853812	0.07441906	0.00763355	0.03122151
Rapgef1	4138.38388	1.07903956	0.10974776	0.0519779	0.02931482	0.09194976
Rapgef2	5338.30375	-1.0594715	-0.0833447	0.05842287	0.13753928	0.29281671
Rapgef3	224.759526	1.09028633	0.12470706	0.12676011	0.22982051	0.41853384
Rapgef4	96.6813117	-1.1336023	-0.1809146	0.20279806	0.1482613	0.30876723
Rapgef6	1657.82317	1.00998669	0.01433628	0.05832984	0.79875492	0.88851089

Rapgefl1	42.3813276	1.09091391	0.12553725	0.21543655	0.24519591	0.43551852
Raph1	3027.01477	-1.0975348	-0.1342667	0.05752199	0.01506891	0.05469898
Rapsn	2.04433086	1.0230041	0.03281193	0.21813684	0.12882078	0.27951711
Rara	1372.24325	-1.0461269	-0.0650579	0.08177936	0.38782361	0.58925637
Rarg	603.1834	1.23264468	0.30175699	0.10248804	0.00104622	0.0060515
Rarres2	1213.01807	-1.0050401	-0.007253	0.06659567	0.90812055	0.95230265
Rars2	405.815795	-1.0652468	-0.0911877	0.10140294	0.30689624	0.50524879
Rasa1	5231.72586	1.11949776	0.16285164	0.06244892	0.00629029	0.0266024
Rasa2	1639.69228	-1.1130365	-0.1545008	0.07831176	0.03349321	0.10249596
Rasa3	1313.3558	1.01329321	0.0190517	0.07481817	0.78663763	0.88053373
Rasa4	61.0715112	1.06573302	0.09184607	0.18450375	0.40463012	0.60404519
Rasal2	2622.18822	1.13975537	0.1887242	0.07290654	0.0058284	0.02500397
Rasd1	7.29075311	1.05123416	0.07208406	0.22401984	0.22286066	0.41118322
Rasgrf1	3.08490903	1.02526574	0.03599789	0.21745514	0.27979682	0.47554693
Rasip1	22.9991637	1.10570402	0.14496525	0.24855337	0.14545484	0.30503506
Rasl10b	2.84209848	-1.039025	-0.0552303	0.2246548	0.04099683	0.11987432
Rasl11a	293.133255	-1.138303	-0.1868846	0.11493255	0.05677749	0.15328128
Rassf1	161.05477	-1.018551	-0.0265182	0.14623957	0.8038169	0.89158321
Rassf3	2746.56063	-1.2116508	-0.2769739	0.05708269	4.93E-07	6.70E-06
Rassf5	284.031645	-1.2333608	-0.3025949	0.11995924	0.00352632	0.01669952
Rassf8	2434.98625	-1.075038	-0.1043876	0.07223123	0.12392555	0.27212456
Raver2	7.84761001	-1.0461962	-0.0651534	0.2228116	0.22010545	0.40733182
Rb1	1514.22626	-1.0695972	-0.0970676	0.06605336	0.12151203	0.26795819
Rb1cc1	2249.24705	-1.098227	-0.1351763	0.08370674	0.07859654	0.19534763
Rbak	343.775793	1.13301875	0.18017173	0.1453036	0.11198114	0.25317302
Rbbp5	1467.82853	-1.0387024	-0.0547823	0.0840721	0.4781663	0.66855602
Rbbp6	5844.27774	-1.100099	-0.1376334	0.06770449	0.0306513	0.09546394
Rbbp8	2979.86887	1.12431832	0.16905055	0.07046471	0.0108734	0.04211922
Rbbp9	129.506941	-1.0703106	-0.0980295	0.15722489	0.38308907	0.58445306
Rbck1	2787.3331	1.10991832	0.15045351	0.06763806	0.01865098	0.0647663
Rbfa	494.251501	-1.0824082	-0.1142447	0.09372321	0.17469535	0.34798268
Rbfox2	1124.16323	-1.0180591	-0.0258213	0.07163817	0.70161236	0.83028433
Rbks	131.246464	-1.1742418	-0.2317295	0.20805366	0.07979462	0.19775664
Rbl1	2705.3838	1.34646211	0.42917363	0.07124645	3.48E-10	7.89E-09
Rbl2	2996.45522	1.40547188	0.49105459	0.06268927	6.30E-16	2.74E-14
Rbm10	3407.55947	-1.061621	-0.0862689	0.05509955	0.1046205	0.24112075
Rbm12	2499.33942	-1.1254494	-0.1705013	0.05738498	0.00198764	0.01038121
Rbm14	1718.56993	-1.1969546	-0.2593684	0.09562379	0.00264859	0.0132223
Rbm15	1759.4813	1.00225931	0.00325582	0.07355126	0.96301612	0.98254201
Rbm15b	1242.34945	-1.1371828	-0.1854642	0.06448836	0.00247462	0.01250487
Rbm17	6300.77501	-1.0403706	-0.0570975	0.06349859	0.34518227	0.54455478
Rbm18	940.951494	1.0464334	0.06548049	0.08628883	0.40792598	0.60698476
Rbm19	4612.15242	1.05363084	0.07536947	0.04247238	0.07012973	0.17945703
Rbm22	1887.28369	-1.0817469	-0.113363	0.07911306	0.12178453	0.26842048
Rbm25	4785.36444	-1.1434509	-0.1933944	0.07946354	0.0085738	0.03446199
Rbm26	4298.74612	1.02538216	0.0361617	0.05256141	0.47864312	0.66910639
Rbm27	2505.79081	-1.0167138	-0.0239136	0.05929347	0.67432697	0.81200681
Rbm28	3274.74082	-1.1292981	-0.1754264	0.05139635	0.00042508	0.0028214
Rbm3	1615.2015	1.12991504	0.1762143	0.06872542	0.00665048	0.02779533
Rbm33	1989.93431	-1.5321401	-0.6155482	0.08039611	2.23E-15	9.32E-14

Rbm34	769.410386	-1.0590583	-0.0827821	0.09969546	0.35148377	0.55110246
Rbm39	5010.22207	-1.0709462	-0.098886	0.05401644	0.05798153	0.1557901
Rbm41	384.637147	-1.0071306	-0.0102508	0.11271794	0.91412746	0.95544817
Rbm42	1277.2756	1.11015538	0.15076162	0.07955321	0.04069275	0.11923014
Rbm43	244.417989	-1.214745	-0.2806535	0.14026799	0.01450477	0.05311781
Rbm44	7.60537178	-1.0489094	-0.06889	0.22401951	0.20681618	0.39127919
Rbm45	746.333417	-1.0059215	-0.0085177	0.08382139	0.91365665	0.95529506
Rbm4b	94.4822745	1.05890041	0.08256691	0.19025612	0.43753075	0.63502934
Rbm5	2658.35957	-1.0530846	-0.0746213	0.05664718	0.17142771	0.34337407
Rbm6	3566.63316	1.05607725	0.07871537	0.07226042	0.2467878	0.43737572
Rbm7	2501.57311	-1.088077	-0.1217807	0.06433334	0.04583795	0.13042738
Rbms1	6970.74817	-1.1514427	-0.2034426	0.04858246	1.73E-05	0.00016889
Rbms2	730.865621	1.19560412	0.25773977	0.10608318	0.00608999	0.02588394
Rbms3	477.614776	-1.0156048	-0.0223391	0.09926214	0.7992776	0.88870289
Rbmx	1494.48097	-1.2880571	-0.3651965	0.08378645	3.29E-06	3.80E-05
Rbmx2	992.509392	-1.2134596	-0.2791261	0.10621377	0.00304433	0.01477073
Rbpj	3129.29417	-1.1532669	-0.2057264	0.05772278	0.00020884	0.00155086
Rbpms	758.966233	1.17204702	0.22903045	0.08654368	0.0038829	0.01806463
Rbsn	988.181185	-1.0831021	-0.1151692	0.07654195	0.10589688	0.24323812
Rbx1	2387.88576	-1.0641355	-0.0896819	0.0624328	0.13231478	0.28443569
Rc3h1	2749.89833	-1.1279776	-0.1737384	0.05535599	0.0011287	0.0064375
Rc3h2	3942.65358	-1.0364935	-0.0517111	0.05006178	0.28758816	0.48381821
Rcan3	249.719462	1.09026594	0.12468009	0.13047444	0.23712123	0.42667831
Rcbtb2	1552.66088	1.08440217	0.1168999	0.06473559	0.05756756	0.15498958
Rcc1l	522.957249	-1.2764702	-0.3521598	0.10359286	0.00017093	0.00130491
Rcc2	2812.86172	-1.062502	-0.0874655	0.078595	0.23007773	0.41882843
Rccd1	276.544104	-1.0013089	-0.0018871	0.12143966	0.98374857	0.99141261
Rce1	67.4883906	1.06526198	0.09120828	0.18372786	0.4085114	0.60757356
Rchy1	635.384778	-1.0579176	-0.0812273	0.11010073	0.38996295	0.59159468
Rcl1	2441.64564	-1.0784404	-0.1089464	0.05348899	0.03498226	0.10591416
Rcn1	4446.19593	1.28663452	0.3636023	0.06025166	4.28E-10	9.50E-09
Rcn2	1663.58966	1.1154066	0.15756971	0.06974154	0.01659638	0.05894085
Rcn3	4788.25007	-1.3041111	-0.3830668	0.06798845	4.16E-09	8.05E-08
Rcor1	1361.53594	-1.2219946	-0.289238	0.0938715	0.00070713	0.00435495
Rcor3	555.693508	1.00184257	0.00265582	0.09395001	0.9763149	0.98895979
Rdh10	235.782212	-1.6314257	-0.7061333	0.14225798	6.52E-08	1.04E-06
Rdh11	1006.20348	-1.0247854	-0.0353219	0.08916752	0.66261307	0.80471736
Rdh13	135.342665	1.10150067	0.13947037	0.15093206	0.21893002	0.40580141
Rdh14	326.490009	1.04274689	0.06038901	0.12411617	0.55445812	0.72989503
Rdh5	409.62181	1.01069503	0.01534774	0.11235697	0.87417513	0.93221108
Rdh8	2.77647844	1.00217032	0.00312771	0.21307801	0.92351316	0.96109284
Rdm1	2.48457089	1.00859626	0.01234878	0.2141042	0.65744763	0.80140161
Rdx	14443.1159	1.23717954	0.30705488	0.0481471	6.38E-11	1.63E-09
Reck	292.17787	1.17037371	0.22696927	0.13079483	0.03577791	0.10781346
Recql	1498.87038	1.05147983	0.07242118	0.06442127	0.23813813	0.42784705
Recql4	1230.82767	1.11258953	0.15392143	0.06705155	0.01529661	0.05538429
Recql5	297.771841	-1.0223393	-0.0318741	0.12573471	0.7537102	0.86298066
Reep3	2806.89728	-1.0784484	-0.1089572	0.06564522	0.08036246	0.19865749
Reep4	819.643503	-1.1477151	-0.1987646	0.10807231	0.03460858	0.10510572
Reep5	2205.44519	1.00580933	0.00835684	0.06031696	0.88578761	0.93872913

Rel	406.256679	-1.0722936	-0.1006999	0.10703788	0.27861326	0.47441657
Rela	5575.22435	1.09993454	0.13741767	0.04731851	0.00285107	0.01401466
Relb	373.130933	-1.0653881	-0.0913791	0.11214324	0.3395311	0.53915872
Relch	2499.27182	1.07418918	0.1032481	0.0556777	0.05798651	0.1557901
Rell2	21.3293679	2.46794614	1.30331091	0.71383671	0.00208508	0.01077881
Reln	2.8908882	-1.0371361	-0.0526053	0.22464321	0.00647571	0.0272069
Relt	196.224193	1.02350411	0.03351689	0.12875974	0.74817873	0.86041394
Rep15	5.49851599	-1.0444269	-0.0627115	0.22242065	0.21329009	0.39921771
Repin1	229.027824	-2.2517733	-1.1710616	0.17857006	3.08E-12	9.35E-11
Reps1	3520.26265	1.10097775	0.13878531	0.04739091	0.00264408	0.01321131
Reps2	1.70804118	-1.0288829	-0.0410788	0.22081342	0.03046492	0.09497582
Rer1	2772.00339	1.07800935	0.10836969	0.05289761	0.03428255	0.104363
Rere	3271.42404	1.13521589	0.18296669	0.0753018	0.00935823	0.03701109
Resf1	2498.40706	1.08664826	0.11988502	0.05386075	0.02120479	0.07191775
Retreg1	733.324845	-1.1649686	-0.2202911	0.09712563	0.01090472	0.04217765
Retreg2	1786.32859	1.12981448	0.1760859	0.08781323	0.02778356	0.08816135
Retreg3	711.864086	1.01554507	0.02225426	0.09369444	0.79235095	0.88430177
Retsat	185.683754	-1.5767554	-0.6569588	0.19027957	4.91E-05	0.00043104
Rev1	1175.49363	-1.0124819	-0.0178961	0.07015082	0.78687271	0.88058317
Rev3l	6205.90182	-1.0151785	-0.0217334	0.05098633	0.65855493	0.80198581
Rex1bd	582.107173	1.15544356	0.20844679	0.11009063	0.02919204	0.09169936
Rexo1	1598.26937	-1.0358511	-0.0508167	0.0706645	0.4433398	0.64034083
Rexo4	1007.02636	-1.0422976	-0.0597672	0.0882769	0.45617818	0.6503867
Rfc1	8619.9991	1.05600642	0.07861861	0.05801655	0.15913785	0.32540277
Rfc2	979.066302	1.11102142	0.15188663	0.08355563	0.04791476	0.1348392
Rfc3	949.478599	-1.1762693	-0.2342183	0.07815691	0.00130221	0.00722135
Rfc4	1401.74623	1.02338828	0.03335361	0.06777092	0.60453501	0.76584726
Rfc5	2546.35769	1.27332792	0.348604	0.07021619	1.91E-07	2.82E-06
Rfesd	939.889021	1.04108259	0.05808452	0.07934688	0.43138525	0.62946179
Rffl	244.79476	1.0593455	0.0831732	0.11637921	0.39726342	0.59750998
Rfk	1463.56672	-1.0916375	-0.1264938	0.06579629	0.04241032	0.12312175
Rfng	652.80445	1.21257666	0.27807596	0.11142583	0.0044321	0.0200761
Rft1	257.070052	1.06288648	0.08798752	0.11189822	0.35846606	0.55863142
Rftn2	1447.10685	1.01370661	0.01964016	0.06691422	0.75806062	0.8657178
Rfwd3	1531.31621	-1.0134639	-0.0192947	0.06429791	0.75259348	0.8628589
Rfx1	227.739675	1.0106326	0.01525862	0.1361512	0.88611695	0.93881053
Rfx2	328.308633	1.02982134	0.04239407	0.11500811	0.66399014	0.80543405
Rfx3	361.10713	1.12211925	0.166226	0.12581795	0.11028834	0.25057977
Rfx5	1122.65555	-1.0788341	-0.1094731	0.08649779	0.16539933	0.33408473
Rfx7	3583.92978	-1.1319554	-0.1788171	0.0492777	0.00018809	0.00142231
Rfx8	2.1871939	-1.0213513	-0.0304792	0.21730859	0.21430008	0.40023094
Rfxank	271.702368	1.04671076	0.06586283	0.11299207	0.49502813	0.68171472
Rfxap	206.520774	-1.001065	-0.0015357	0.14369027	0.98730734	0.99374614
Rgl1	823.903555	1.02194563	0.03131845	0.08248392	0.68190987	0.81683798
Rgl2	739.802628	1.05967549	0.08362253	0.10553189	0.36326739	0.56391848
Rgl3	3.40986545	-1.0329149	-0.0467213	0.21901404	0.24831638	0.43923403
Rgma	147.73161	1.15394775	0.2065779	0.17249805	0.09569751	0.22576154
Rgmb	639.475721	-1.0712055	-0.0992352	0.11748881	0.31564182	0.5146858
Rgp1	190.266741	1.28670003	0.36367576	0.15341795	0.00378951	0.0176844
Rgs10	338.372834	1.02465297	0.03513538	0.11605461	0.71895084	0.84156113

Rgs12	503.047182	1.29415017	0.37200503	0.13474098	0.00126741	0.00706201
Rgs16	281.4846	-2.8806737	-1.5264063	0.13385157	2.64E-31	2.95E-29
Rgs17	236.505225	1.76599718	0.82048304	0.17059059	1.23E-07	1.87E-06
Rgs19	639.906014	1.29335287	0.37111594	0.12011555	0.00045556	0.00299575
Rgs2	64.5664641	-1.0256469	-0.0365342	0.16882988	0.73047369	0.84898311
Rgs3	28.4826404	1.04491214	0.06338164	0.19557458	0.51290418	0.69679862
Rhbdd1	1367.08925	1.11300035	0.15445404	0.06522216	0.01265115	0.0474418
Rhbdd2	538.098551	-1.0523411	-0.0736024	0.09051516	0.36864109	0.57004978
Rhbdd3	211.912608	1.13014325	0.17650565	0.19156211	0.1545084	0.31805442
Rhbdf1	1200.25923	-1.2237281	-0.291283	0.08439661	0.00019471	0.00146118
Rhbdf2	915.028228	-1.4475786	-0.5336417	0.10687635	7.67E-08	1.21E-06
Rhbdl1	7.50176279	1.04385626	0.06192306	0.22499522	0.11865301	0.26346768
Rheb	1652.29268	-1.8584223	-0.8940784	0.10235157	1.92E-19	1.11E-17
Rhebl1	177.883946	1.00079833	0.00115129	0.12244978	0.99187666	0.99615366
Rhno1	425.014298	-1.0662777	-0.0925832	0.11327111	0.3367262	0.5365742
Rhoa	7146.44292	1.09017909	0.12456515	0.06244569	0.05355371	0.14642969
Rhob	134.142609	1.66887358	0.73887467	0.17804985	2.77E-06	3.25E-05
Rhobtb1	1145.04929	1.18391292	0.24356297	0.07194067	0.0003316	0.00227154
Rhobtb2	498.190691	-1.036203	-0.0513067	0.10140026	0.56442534	0.73598214
Rhobtb3	3072.99336	-1.0659508	-0.0921408	0.06181723	0.11858602	0.26341025
Rhoc	1142.01838	-1.0919013	-0.1268424	0.08251333	0.09457209	0.22405557
Rhod	559.229945	1.10756289	0.14738862	0.11000899	0.12094341	0.26716418
Rhog	625.840271	-1.0858897	-0.1188776	0.10105836	0.18233149	0.35837908
Rhoj	9.4856912	-1.1127178	-0.1540877	0.29084421	0.02650851	0.08529155
Rhoq	337.589314	1.449757	0.5358111	0.13966228	1.55E-05	0.00015399
Rhot1	1046.99278	1.04161686	0.05882471	0.08512437	0.45216853	0.6470374
Rhot2	612.636349	-1.0488011	-0.0687412	0.08634242	0.38426615	0.58544831
Rhou	76.0050965	1.36689294	0.45090025	0.24047234	0.0071983	0.02975682
Rhpn1	105.732645	1.1937383	0.2554866	0.18571544	0.05142117	0.14199293
Ribc1	140.188413	1.10057214	0.13825372	0.15293856	0.226433	0.41508396
Ric1	1310.29204	-1.014081	-0.0201728	0.07383127	0.77029398	0.87232885
Ric8a	721.829095	1.04243867	0.05996251	0.10967575	0.51930598	0.70216917
Ric8b	729.635693	1.01933142	0.02762319	0.08549455	0.7230419	0.84385629
Rictor	3797.87993	1.16946641	0.22585043	0.05276	9.78E-06	0.00010187
Rida	113.801875	-1.2130746	-0.2786682	0.2102707	0.0454533	0.12962083
Rif1	9152.2847	-1.0187259	-0.0267659	0.05185382	0.59430845	0.7576723
Rilp	263.063141	1.06521846	0.09114934	0.12714214	0.37910694	0.58125193
Rilpl1	1013.18493	1.0249118	0.03549976	0.06845893	0.58477867	0.74932628
Rilpl2	369.876394	1.1706266	0.22728097	0.1379337	0.04187196	0.12184912
Rimbp3	9.13369757	-1.0407966	-0.0576881	0.21667603	0.34439543	0.54378226
Rims2	294.88602	1.0818226	0.11346394	0.13043594	0.28168838	0.47768443
Rims3	76.5670537	1.7018351	0.76709125	0.29839289	0.00064478	0.00402711
Rin1	309.46955	-1.3805852	-0.46528	0.12679085	3.78E-05	0.00033946
Rin3	2.79509376	-1.015355	-0.0219843	0.21562994	0.3827153	0.58431392
Ring1	1165.84779	1.05680812	0.07971346	0.09475301	0.34911629	0.54880104
Rint1	525.359262	-1.0762599	-0.1060265	0.09428108	0.20986768	0.39519079
Riok1	1605.97783	-1.1334701	-0.1807464	0.07850103	0.01317463	0.04898898
Riok2	2187.17758	-1.2001528	-0.2632181	0.06910078	5.85E-05	0.00050499
Riok3	2365.20854	-1.0866231	-0.1198517	0.05739117	0.04130632	0.12061407
Ripk1	1780.24215	-1.0387294	-0.0548198	0.06254872	0.35840961	0.5586114

Ripk2	1033.46445	-1.1739312	-0.2313479	0.07388487	0.00085836	0.00514777
Ripk4	2.00018758	1.01683736	0.02408895	0.21554644	0.40619399	0.60567396
Ripor1	2400.51292	1.05686659	0.07979328	0.06166855	0.1760855	0.34986813
Ripor3	2.20130612	-1.0135532	-0.0194218	0.21490009	0.48615227	0.67499887
Rit1	318.087561	-1.0843665	-0.1168524	0.11931214	0.24249786	0.43246665
Rita1	131.771915	1.00724265	0.01041128	0.14584986	0.92400644	0.96109284
Rlf	2175.74815	-1.0417702	-0.0590371	0.06925876	0.36690439	0.56825599
Rlim	5038.97728	-1.1451277	-0.1955085	0.06354853	0.00124083	0.00693727
Rmc1	636.389579	1.06010422	0.0842061	0.09531673	0.32395504	0.52337798
Rmdn1	30.4066255	-1.0627585	-0.0878138	0.20129359	0.38473679	0.58596609
Rmdn2	277.011243	1.0785248	0.10905935	0.14196031	0.32071226	0.51938358
Rmdn3	1428.04313	1.10475864	0.14373121	0.07085225	0.03132957	0.09710422
Rmi1	681.693953	-1.015147	-0.0216887	0.08642786	0.78278209	0.87844403
Rmi2	530.596035	1.04612257	0.0650519	0.0936815	0.43993411	0.63736213
Rmnd1	590.749484	1.05345857	0.07513358	0.11242895	0.43467997	0.6323246
Rmnd5a	1563.84096	-1.7893727	-0.8394539	0.07495542	3.20E-30	3.50E-28
Rmnd5b	141.449656	1.03449337	0.0489244	0.17026074	0.64956706	0.79679729
Rnaseh1	1020.30793	-1.044154	-0.0623346	0.08478025	0.4228925	0.6214584
Rnaseh2a	1935.24058	-1.0459434	-0.0648048	0.06890221	0.31971496	0.51816151
Rnaseh2b	723.989573	-1.0366503	-0.0519293	0.09020531	0.52494553	0.70665744
Rnaseh2c	676.988095	1.41895035	0.50482411	0.11708497	2.41E-06	2.88E-05
Rnasel	1076.78573	-1.1677279	-0.2237041	0.08638866	0.00475277	0.02124022
Rnd1	48.4209674	-1.0530064	-0.0745142	0.20778402	0.40095828	0.60080425
Rnd2	125.571365	1.33393767	0.41569125	0.20867896	0.00681168	0.0283857
Rnd3	2505.07668	-1.0237996	-0.0339333	0.06278863	0.57164174	0.7403946
Rnf10	3222.12509	1.06222157	0.08708473	0.05549953	0.10394191	0.24029884
Rnf103	449.200294	-2.2579777	-1.1750312	0.11290911	1.54E-26	1.34E-24
Rnf11	837.333363	-1.1995106	-0.2624459	0.10199706	0.00392368	0.01819123
Rnf111	2984.611	1.00168321	0.00242632	0.05601027	0.96489833	0.98295812
Rnf113a1	308.26417	-1.454119	-0.5401453	0.15589306	6.27E-05	0.00053732
Rnf114	2357.31799	1.35848892	0.4420028	0.07840174	3.25E-09	6.42E-08
Rnf115	3062.16215	1.13801588	0.18652069	0.05114444	0.00016823	0.00128659
Rnf121	414.32735	-1.2205129	-0.2874876	0.10940231	0.00291141	0.01423482
Rnf122	82.3626847	1.41963418	0.50551922	0.26941765	0.00568232	0.02451685
Rnf123	1015.0593	1.09173004	0.12661616	0.10556338	0.16926231	0.34022442
Rnf125	85.0715463	-1.4135717	-0.4993451	0.31528603	0.009154	0.03636055
Rnf126	1726.9177	1.0045146	0.00649853	0.08412614	0.93405093	0.96797933
Rnf13	2684.15664	-1.0053046	-0.0076328	0.06227508	0.89867782	0.94726921
Rnf130	491.435488	1.19510154	0.2571332	0.09850444	0.00367307	0.01726045
Rnf138	634.237344	1.01199132	0.01719692	0.08565318	0.82816512	0.90782091
Rnf139	2822.02652	-1.2483993	-0.3200794	0.07505243	6.23E-06	6.73E-05
Rnf14	738.283817	1.02116016	0.03020916	0.10002791	0.73433833	0.8514657
Rnf141	980.292545	1.05046689	0.07103069	0.0707539	0.28738163	0.48368923
Rnf145	822.278813	-1.0417601	-0.059023	0.11385667	0.5413208	0.71947701
Rnf146	485.439779	-1.0610631	-0.0855105	0.11238234	0.37331365	0.57484647
Rnf149	3386.66336	-1.1755401	-0.2333237	0.08746205	0.00353861	0.01674531
Rnf151	2.87630574	1.02591009	0.0369043	0.21720852	0.29428987	0.49157037
Rnf157	32.3042488	1.04821216	0.06793076	0.20764943	0.42787075	0.62590205
Rnf166	108.188636	1.63413347	0.70852583	0.2428522	0.00026673	0.00189823
Rnf169	456.831337	1.08199883	0.11369894	0.11064568	0.23294429	0.42235768

Rnf185	1230.76232	1.06081636	0.08517493	0.06882435	0.19100764	0.369648
Rnf19a	494.654053	-1.0909133	-0.1255365	0.11230125	0.1907765	0.36960782
Rnf19b	1421.20698	1.03390459	0.04810306	0.0636849	0.42908511	0.6268195
Rnf2	3252.76257	-1.1766178	-0.2346457	0.05535868	1.12E-05	0.00011527
Rnf20	4966.4285	1.01338122	0.019177	0.05762626	0.73042545	0.84898311
Rnf212	171.406885	1.2578261	0.33093247	0.17573764	0.01345855	0.04989985
Rnf213	12736.8001	1.13409265	0.1815385	0.05134793	0.00026324	0.00187545
Rnf214	3361.99402	1.28720441	0.36424117	0.06674588	1.29E-08	2.32E-07
Rnf215	510.231785	-1.0473621	-0.0667602	0.10092622	0.4534257	0.64825706
Rnf216	3847.02551	1.08234804	0.11416448	0.05088328	0.02078442	0.07075429
Rnf217	881.425596	-1.0359727	-0.050986	0.07959341	0.4896236	0.67718402
Rnf219	930.079155	1.15840063	0.21213429	0.1004436	0.01768327	0.06231712
Rnf220	1662.42476	1.03729975	0.05283286	0.06364304	0.38475153	0.58596609
Rnf223	73.621412	-1.0647689	-0.0905403	0.18797876	0.40175817	0.60136973
Rnf24	54.5370066	1.0337443	0.04787938	0.17897742	0.64700989	0.79487837
Rnf25	765.149688	-1.0302655	-0.0430161	0.07688022	0.54806107	0.72497545
Rnf31	805.632143	1.17037477	0.22697057	0.10230394	0.01236681	0.04664814
Rnf32	31.2366118	1.07365897	0.10253581	0.21828863	0.28011988	0.47590659
Rnf34	379.136753	-1.1036763	-0.1423172	0.10566738	0.12229996	0.26932482
Rnf38	700.192081	1.06092251	0.08531928	0.08218256	0.25950729	0.4518538
Rnf4	1051.02533	-1.0612311	-0.0857388	0.09181466	0.30072965	0.4985566
Rnf40	4019.44938	-1.0077302	-0.0111094	0.04653271	0.80983591	0.89616431
Rnf41	885.062907	-1.0333948	-0.0473915	0.08748453	0.55268923	0.72867565
Rnf43	6.36165336	1.04557294	0.06429371	0.22331168	0.20392314	0.38757499
Rnf44	1556.39622	-1.0120764	-0.0173183	0.0709394	0.79531045	0.88617607
Rnf5	929.256142	1.05008584	0.07050727	0.08273089	0.35547335	0.55538646
Rnf6	7265.30082	-1.0328237	-0.046594	0.05252962	0.35988206	0.56008859
Rnft1	1195.98235	1.01519468	0.02175642	0.07299733	0.75183923	0.86245729
Rnft2	436.99588	1.10152416	0.13950114	0.09883755	0.11268119	0.25426211
Rnh1	888.861021	1.1428843	0.19267936	0.09615449	0.02529695	0.08234704
Rnmt	2276.86419	-1.1790546	-0.2376305	0.07442443	0.00066524	0.00413876
Rnpc3	891.492763	1.12982154	0.17609491	0.11674196	0.07443623	0.18772644
Rnpep	1416.82916	1.06601612	0.09222925	0.07930511	0.20989109	0.39519079
Rnpepl1	1425.17992	-1.1593196	-0.2132784	0.1039655	0.01977128	0.0679555
Rnps1	4215.60472	-1.0234848	-0.0334896	0.0504778	0.492271	0.67949091
Ro60	643.840496	1.12550613	0.17057392	0.09820559	0.05122468	0.14157241
Robo1	2221.48558	-1.1405838	-0.1897725	0.05979545	0.00093609	0.00551749
Robo2	812.4227	1.12035873	0.16396074	0.08613717	0.03750511	0.11197149
Rock1	4042.76432	-1.0716468	-0.0998294	0.06506797	0.10647568	0.24417355
Rock2	18513.1931	-1.0180255	-0.0257738	0.06328858	0.67363289	0.81139984
Rogdi	436.054425	1.36570375	0.44964457	0.11629971	1.83E-05	0.00017753
Rom1	164.87153	1.00222617	0.00320812	0.15060722	0.97760129	0.98973149
Ropn1l	2.14485461	-1.0238049	-0.0339409	0.21768694	0.22318693	0.41142965
Ror1	225.202007	-1.1060652	-0.1454364	0.13307044	0.17292158	0.34551948
Rorb	142.591244	1.22543273	0.29329129	0.18588959	0.02923918	0.09180243
Rp2	703.241294	-1.3094733	-0.3889866	0.10021194	1.91E-05	0.00018396
Rp9	220.441473	1.05008028	0.07049963	0.14050199	0.51461261	0.69825098
Rpa1	8703.97838	-1.0541848	-0.0761278	0.04448892	0.07985001	0.19775664
Rpa2	2774.33712	-1.066814	-0.0933086	0.0731516	0.17373485	0.34660387
Rpa3	1139.57839	-1.0443331	-0.062582	0.09660016	0.46741208	0.660123

Rpain	239.715949	-1.012331	-0.0176811	0.14594383	0.86771007	0.92836933
Rpap1	991.890206	-1.066463	-0.092834	0.08701896	0.2418241	0.43174449
Rpap2	755.990283	1.09571652	0.13187459	0.0910402	0.10919401	0.24858277
Rpap3	2650.27625	1.03280923	0.0465738	0.05426038	0.37530219	0.57721468
Rpe	2191.65218	-1.0084278	-0.0121078	0.05973249	0.83241153	0.91007181
Rpe65	1.65504527	-1.0203345	-0.0290422	0.21784266	0.11202514	0.25318314
Rpf1	3956.19782	-1.0080096	-0.0115094	0.0721834	0.86588371	0.92774752
Rpgr	1734.75497	1.13082992	0.17738196	0.07130914	0.00820514	0.03317798
Rpgrip1	2.0477431	-1.0289391	-0.0411576	0.21966449	0.1404991	0.29748653
Rpgrip1l	1184.3784	-1.0618852	-0.0866278	0.09743913	0.31576527	0.51482158
Rph3al	344.959903	-1.2280037	-0.2963149	0.13843622	0.0096436	0.03804574
Rpl13	22348.5468	-1.0277976	-0.0395563	0.06248776	0.51371839	0.69754429
Rpl14	19909.0084	1.02021762	0.02887692	0.05288502	0.57370891	0.74199183
Rpl15	14079.3573	-1.0516642	-0.0726741	0.05649903	0.18118813	0.35715173
Rpl18a	25332.3476	-1.061226	-0.0857319	0.04703964	0.0598454	0.1597067
Rpl22	15760.9683	-1.0511457	-0.0719627	0.06869365	0.26797526	0.46238726
Rpl22l1	3246.10083	-1.1115508	-0.1525739	0.22386365	0.18423421	0.36075211
Rpl23	26763.5546	-1.0648794	-0.09069	0.07092314	0.17415781	0.3473395
Rpl23a	30000.7767	-1.1372437	-0.1855415	0.18559049	0.13665667	0.29147051
Rpl24	8436.60398	-1.0808489	-0.1121648	0.05702279	0.03698707	0.11067609
Rpl27	12758.3278	-1.1993365	-0.2622365	0.06419196	1.81E-05	0.00017605
Rpl28	3894.94811	-1.0450651	-0.0635928	0.07777428	0.38542071	0.58657946
Rpl3	33378.0083	1.0363608	0.05152635	0.04010278	0.19467072	0.37465924
Rpl34	14797.4901	-1.0968452	-0.13336	0.07032265	0.04397207	0.12648627
Rpl35	13426.0158	-1.1138924	-0.1556099	0.06579173	0.01261177	0.04732185
Rpl35a	11910.3349	1.07790526	0.10823038	0.06232079	0.06915085	0.17786965
Rpl36	5080.82116	-1.1045407	-0.1434466	0.13686477	0.1868501	0.36414876
Rpl7a	8973.96525	1.00485585	0.00698856	0.08596051	0.92475272	0.96122057
Rpl7l1	2222.48128	-1.2194307	-0.2862078	0.06083193	9.59E-07	1.24E-05
Rpn1	11249.757	-1.0437659	-0.0617981	0.05847893	0.27372646	0.46933371
Rpn2	11520.6599	1.07960238	0.11050006	0.05634061	0.04146605	0.12088759
Rpp14	55.9344077	-1.0100053	-0.0143628	0.2130157	0.68491581	0.81904412
Rpp21	300.048259	1.04062159	0.05744554	0.13912901	0.5919611	0.75498025
Rpp38	771.445028	-1.0004665	-0.0006729	0.08209309	0.99601699	0.99827695
Rpp40	1388.19745	-1.1239743	-0.168609	0.07191163	0.01255077	0.04714822
Rprd1a	166.214235	1.10660374	0.1461387	0.16652399	0.21352044	0.39933082
Rprd1b	1042.76608	1.28122921	0.3575286	0.075624	5.94E-07	7.97E-06
Rprd2	3830.15433	-1.0291289	-0.0414237	0.05328495	0.42166342	0.62015022
Rprm	24.2250561	-1.0051973	-0.0074787	0.19652452	0.92417968	0.96109284
Rps10	13967.0553	-1.0793587	-0.1101744	0.05214355	0.02933896	0.09200295
Rps11	26600.6016	-1.5612081	-0.6426629	0.04548361	1.53E-46	3.63E-44
Rps13	3148.31294	-1.1838684	-0.2435087	0.0787473	0.0009027	0.00536507
Rps18	16066.2044	1.09029148	0.12471387	0.06054012	0.03155977	0.09769953
Rps19	283.991639	1.10722898	0.14695361	0.12602225	0.15659579	0.32152461
Rps19bp1	647.654525	-1.2279269	-0.2962247	0.10854061	0.0020527	0.01064148
Rps2	22662.588	-1.0477739	-0.0673274	0.06999594	0.30883907	0.50720878
Rps20	9365.99123	-1.2123963	-0.2778614	0.0610966	2.00E-06	2.43E-05
Rps23	20844.8787	-1.0709349	-0.0988708	0.05356634	0.0560564	0.15175032
Rps24	37733.3345	1.00083883	0.00120967	0.06303589	0.98358374	0.99141261
Rps27a	14108.083	-1.0786354	-0.1092073	0.1498316	0.32527771	0.52498519

Rps27l	1542.20783	-1.0176778	-0.0252809	0.14175443	0.81157947	0.89730545
Rps3	14919.3187	-1.0155224	-0.022222	0.05129897	0.6450974	0.79348514
Rps4x	29904.1546	-1.0735945	-0.1024492	0.04710877	0.02680375	0.08592493
Rps5	5361.81649	-1.1118584	-0.152973	0.08596007	0.05116748	0.14147538
Rps6ka1	915.477179	1.15823695	0.21193042	0.0747922	0.00247699	0.01251084
Rps6ka2	1287.99974	1.34392766	0.42645548	0.08341885	6.29E-08	1.01E-06
Rps6ka3	5429.53751	-1.1792822	-0.237909	0.06121692	4.91E-05	0.0004313
Rps6ka4	2339.68597	1.05490057	0.07710703	0.06359257	0.20368502	0.38717987
Rps6ka5	240.242004	-1.0036536	-0.0052614	0.11893632	0.95697091	0.97905897
Rps6kb2	559.029229	1.14923846	0.20067818	0.10837527	0.03352331	0.10256355
Rps6kc1	1041.57786	1.07648723	0.1063312	0.07287253	0.12017928	0.2660267
Rps7	23720.2527	-1.1328321	-0.179934	0.04820015	0.00012259	0.00097134
Rpsa	38173.9079	1.09525762	0.13127025	0.04634058	0.0038127	0.01777966
Rptor	3778.84626	1.06183114	0.08655436	0.05425242	0.09887945	0.23101805
Rpusd1	205.726588	1.23260578	0.30171146	0.14591901	0.01092577	0.04219664
Rpusd2	380.952884	-1.1598625	-0.2139538	0.11154296	0.02652305	0.0853026
Rpusd3	273.051513	1.04441959	0.06270142	0.12793704	0.5458435	0.72318588
Rpusd4	534.742417	1.00252503	0.00363826	0.08658384	0.96439569	0.98280897
Rrad	114.070959	-1.0441457	-0.062323	0.15805934	0.56945851	0.73906967
Rragb	604.5352	1.21634624	0.28255396	0.10162982	0.00192682	0.01010751
Rras	388.303108	1.07127079	0.0993232	0.12262952	0.32806861	0.52808662
Rras2	2894.48024	1.10995226	0.15049763	0.06719295	0.01800233	0.06318077
Rrbp1	6070.61081	-1.0247357	-0.0352519	0.054217	0.50123205	0.68649781
Rreb1	2231.75735	-1.0094708	-0.0135992	0.05592537	0.80057524	0.88960521
Rrh	9.61111052	-1.1015479	-0.1395323	0.27019287	0.05532501	0.15011934
Rrm1	6909.96633	-1.0316465	-0.0449488	0.05014631	0.35906427	0.55935951
Rrm2	5076.97169	-1.1396492	-0.1885898	0.05690627	0.00056718	0.00362919
Rrn3	3014.56101	1.35934347	0.44291003	0.06927679	3.16E-11	8.46E-10
Rrnad1	187.61625	1.13102508	0.17763092	0.16734426	0.13940576	0.29562851
Rrp1	2039.91204	-1.1826276	-0.2419959	0.07833952	0.00089868	0.00534455
Rrp12	5441.27542	-1.0270392	-0.0384913	0.05748138	0.48660175	0.67514677
Rrp15	2528.62889	-1.0295118	-0.0419603	0.06243343	0.48199532	0.67185637
Rrp36	253.253841	1.00768386	0.0110431	0.12267375	0.91368267	0.95529506
Rrp8	462.080598	-1.1832812	-0.2427929	0.10082801	0.00692816	0.02880549
Rrp9	2295.24091	-1.0805528	-0.1117695	0.06292987	0.06277866	0.16567669
Rsad1	289.387859	-1.145404	-0.1958565	0.1209861	0.05430861	0.14798835
Rsbn1	303.382629	-1.0320729	-0.0455448	0.1136379	0.63431711	0.78670941
Rsbn1l	1064.37537	-1.0816022	-0.1131699	0.06711223	0.07509226	0.18894752
Rsf1	3838.60843	-1.0367813	-0.0521116	0.06999336	0.43034616	0.62830343
Rskr	29.2245698	-1.1534744	-0.205986	0.30930377	0.06683125	0.17365281
Rsl1d1	5509.28037	-1.3057759	-0.3849073	0.06017847	3.82E-11	1.01E-09
Rsph10b	10.4573645	-1.0125699	-0.0180215	0.20524036	0.779352	0.87673482
Rsph6a	1.65016256	1.01480236	0.02119878	0.2156593	0.39377013	0.59478601
Rsph9	1.64012996	-1.0033623	-0.0048426	0.21444691	0.8160318	0.90029863
Rspry1	1245.58266	-1.0467649	-0.0659374	0.06485172	0.28521073	0.48187146
Rsrc2	2964.2684	1.07330209	0.10205619	0.06051326	0.07845883	0.1953085
Rsrp1	513.764726	1.05265308	0.07403006	0.09651939	0.39109516	0.59250195
Rsu1	5194.08575	-1.058655	-0.0822325	0.0485208	0.08144538	0.20059899
Rtca	1346.85952	-1.0284044	-0.0404077	0.09647587	0.63934638	0.79050546
Rtcb	6116.95969	-1.0666127	-0.0930364	0.04781264	0.04531775	0.12943598

Rtel1	3097.93564	1.48483948	0.57030698	0.0716164	2.24E-16	1.02E-14
Rtf1	4391.98824	-1.0698576	-0.0974188	0.06088047	0.09419765	0.22341638
Rtf2	3538.41654	1.31465817	0.39468772	0.05130768	3.37E-15	1.38E-13
Rtkn	276.076313	-2.1094703	-1.0768808	0.12531488	5.51E-19	3.10E-17
Rtkn2	450.895043	-1.1006671	-0.1383781	0.09986633	0.11740394	0.26164657
Rtl6	80.1968764	1.1520803	0.20424127	0.23375394	0.11312027	0.25480406
Rtl9	19.907498	-5.9081562	-2.562708	0.60749191	1.06E-06	1.36E-05
Rtn2	252.212668	1.05568146	0.07817459	0.14408476	0.47333403	0.66426478
Rtn3	774.589183	-1.0642005	-0.08977	0.09923124	0.30641806	0.50465613
Rtn4	5128.93187	1.01143441	0.01640276	0.04284874	0.69572107	0.82612041
Rtn4ip1	840.881409	1.12599915	0.17120573	0.08905452	0.03446183	0.10475938
Rtn4rl1	413.36881	1.44834335	0.53440365	0.11210026	2.51E-07	3.60E-06
Rtraf	4264.92884	1.08214931	0.11389957	0.05796615	0.04097084	0.11985304
Rttn	621.120341	-1.0110151	-0.0158045	0.08918249	0.84498184	0.91643771
Rubcn	753.073971	1.08703742	0.12040161	0.0863026	0.12655676	0.27581033
Rufy1	201.741598	-1.0725679	-0.101069	0.14825956	0.36186394	0.56235316
Rufy2	237.304939	1.3630085	0.44679456	0.14350314	0.00030006	0.00208447
Rufy3	1150.83249	1.2668317	0.34122487	0.08589532	1.98E-05	0.00018963
Rundc1	1111.57673	1.01309709	0.01877245	0.07588868	0.79203743	0.88402889
Rundc3a	15.1671297	1.05143213	0.07235573	0.21847002	0.31114608	0.5096907
Rundc3b	93.7840633	-1.0227715	-0.0324839	0.16434133	0.76090175	0.86754436
Runx1	2631.55491	-1.3038071	-0.3827305	0.07048904	1.33E-08	2.39E-07
Runx2	2199.77577	1.24732591	0.31883848	0.06880312	1.15E-06	1.46E-05
Rusc1	136.563761	-1.103776	-0.1424475	0.1839334	0.22883118	0.41748237
Rusc2	2155.27384	1.23543724	0.30502173	0.06676318	1.63E-06	2.02E-05
Ruvbl1	7382.74222	-1.0268442	-0.0382173	0.04630858	0.39007892	0.59162937
Rwdd1	2568.26252	1.01951496	0.02788294	0.08944513	0.73277699	0.85067979
Rwdd2a	267.641294	1.19319978	0.25483562	0.15279293	0.03297693	0.10120616
Rwdd2b	1221.21191	-1.0413842	-0.0585024	0.07918377	0.42567777	0.62417241
Rwdd3	124.869716	1.04450006	0.06281258	0.16067581	0.56950413	0.73906967
Rwdd4a	909.328379	-1.1471182	-0.1980141	0.08657974	0.01230307	0.04646295
Rxfp1	1.85236456	-1.0190129	-0.0271723	0.21674693	0.25614198	0.44824846
Rxra	869.620338	1.40540961	0.49099067	0.10283483	2.48E-07	3.56E-06
Rxrb	1112.09892	1.10847817	0.14858036	0.08207427	0.0495591	0.13825176
Rxylt1	1189.75536	1.07854315	0.1090839	0.07404091	0.11593037	0.25934315
Rybp	2346.68676	-1.2335428	-0.3028077	0.05814638	6.64E-08	1.06E-06
Ryr1	7.95204247	-1.0617175	-0.0864	0.2320244	0.14494218	0.30428573
Ryr3	279.401227	-1.6288984	-0.7038966	0.16443215	1.64E-06	2.03E-05
S100a1	258.529384	1.19563051	0.25777162	0.1384277	0.02262684	0.07571043
S100a10	4875.9474	-1.1039153	-0.1426295	0.09104425	0.08284325	0.20302698
S100a11	7113.13363	-1.0519398	-0.0730522	0.08011568	0.32588553	0.52576747
S100a13	852.756342	-1.126626	-0.1720087	0.08535304	0.02769743	0.08797522
S100a4	299.239714	-1.5926334	-0.6714142	0.13469538	6.17E-08	9.94E-07
S100a5	74.3800077	-1.4017709	-0.4872506	0.29539193	0.00884419	0.03533813
S100a6	17294.121	1.21834168	0.28491879	0.06864794	1.26E-05	0.00012749
S100a7a	4.34398408	-1.0574591	-0.0806018	0.23698388	0.00808701	0.03275201
S100pbp	2009.97022	1.16426756	0.21942264	0.06313862	0.00027072	0.00192023
S1pr1	8.80120783	1.04253235	0.06009215	0.22117246	0.23834886	0.4281656
S1pr2	788.782862	-1.1132362	-0.1547597	0.11638404	0.11643868	0.26026514
SAR1B	778.940946	-1.1032061	-0.1417023	0.08374551	0.0654692	0.17115519

SARS	4829.68526	1.01039019	0.01491254	0.05197619	0.76794221	0.8713089
SAT1	1410.36939	-1.2575211	-0.3305826	0.0805855	1.19E-05	0.00012189
SDC1	8514.68369	-1.0837224	-0.1159953	0.06583727	0.0632504	0.16671556
SDHC	1567.4474	1.08591736	0.11891432	0.07578681	0.0929079	0.22097108
SIRT4	87.01246	1.01912289	0.02732803	0.15576232	0.80164442	0.89048431
SLC19A1	756.551733	-1.1379313	-0.1864134	0.1074934	0.04648939	0.13178338
SLC35A1	953.372799	-1.0784585	-0.1089707	0.08252536	0.15159347	0.31376889
SLX1	249.645558	-1.0250498	-0.0356941	0.1238464	0.72303375	0.84385629
SNORA62	32.359871	-1.0111546	-0.0160036	0.19291012	0.85170264	0.92037071
SNORA63	197.096263	-1.0914511	-0.1262475	0.16034688	0.27032134	0.46562005
SNORA66	130.959738	-1.2484783	-0.3201707	0.31194688	0.03939064	0.11634636
SNORA70	11.2084797	-1.0126038	-0.0180698	0.21016469	0.71995887	0.84225325
SNORA71	51.9530789	-1.0391524	-0.0554073	0.18889524	0.57374916	0.74199183
SNORA72	3.71033209	-1.0225966	-0.0322371	0.21600154	0.36773346	0.56933353
SNORA73	3787.86083	-1.2702019	-0.3450578	0.19941536	0.01617108	0.05785942
SNORA74	407.140658	-1.2149748	-0.2809264	0.18229191	0.03338012	0.10220027
SNORA75	1.74741555	-1.0118885	-0.0170504	0.21478842	0.51778396	0.70084663
SNORD14	38.2983797	-1.0816079	-0.1131776	0.24255645	0.13277481	0.28523315
SNORD15	1653.22872	-1.0861042	-0.1191625	0.20538933	0.27348368	0.46917449
SNORD16	5.51836239	1.00390857	0.00562788	0.2111613	0.89627477	0.9455619
SNORD21	2.94252613	1.00700669	0.01007327	0.21354176	0.74496713	0.85818605
SNORD22	352.980778	-1.4836503	-0.5691511	0.2173374	0.00082987	0.00499092
SNORD24	183.718326	-1.2544946	-0.3271062	0.18335751	0.01626442	0.05806761
SNORD33	61.799584	-1.0597326	-0.0837002	0.23367242	0.09708204	0.22809106
SNORD62	2.11823962	1.00295879	0.00426234	0.21333894	0.88749791	0.93972956
SNORD79	12.5629403	-1.0086643	-0.0124461	0.20892006	0.81014451	0.89627352
SNORD81	1.98502265	-1.014074	-0.020163	0.21513069	0.45322464	0.64811427
SNRPD1	4025.11847	-1.1005937	-0.138282	0.06461292	0.02424007	0.07992673
SPTLC1	1506.07319	-1.0376586	-0.0533318	0.06943096	0.4182629	0.61720398
SPTLC2	2108.84465	-1.0038098	-0.0054859	0.06805394	0.93157461	0.96596008
SREBF1	174.01993	1.00100453	0.0014485	0.14143996	0.9903056	0.9954343
SUCLA2	2710.97012	1.0218153	0.03113445	0.0575914	0.57525394	0.74291793
SUMO2	3568.35877	1.00896712	0.01287916	0.14892988	0.90774605	0.95220813
Sac3d1	291.340314	-1.3128475	-0.3926994	0.18984654	0.00638548	0.0269338
Sacm1l	2192.34289	1.17985494	0.2386095	0.06364077	8.49E-05	0.00070434
Sae1	4523.90836	1.03727343	0.05279625	0.05003383	0.27873596	0.47450067
Safb	4453.77232	1.01032865	0.01482466	0.05642548	0.78594446	0.88018569
Safb2	2253.96224	-1.1469995	-0.1978648	0.06282123	0.00094977	0.0055861
Samd1	993.785651	-1.0898345	-0.124109	0.08227325	0.10088032	0.23466078
Samd10	3.69335683	1.02242535	0.03199551	0.21630073	0.33851334	0.53807618
Samd11	42.3961852	2.29814132	1.20046752	0.48593591	0.00055213	0.00355057
Samd12	188.170226	1.1223463	0.16651789	0.16523032	0.16136712	0.32795696
Samd4	852.860752	1.00463458	0.00667084	0.08877723	0.93498094	0.96855134
Samd4b	4433.44524	1.08770783	0.12129108	0.05905917	0.0322655	0.09942771
Samd5	26.4915678	-1.0495699	-0.0697983	0.20948412	0.40375623	0.60349534
Samd8	3305.49571	1.07364996	0.10252371	0.06618161	0.1031482	0.23880868
Samhd1	5460.26103	1.04565444	0.06440616	0.05361287	0.2148028	0.40099445
Samm50	3423.54306	1.05457629	0.07666346	0.06831063	0.23637981	0.42594252
Samsn1	3.63392474	1.01478241	0.02117042	0.21482492	0.48902507	0.67716043
Sap130	1929.89865	-1.0553005	-0.0776539	0.06612494	0.21607996	0.40218896

Sap25	75.6935925	-1.2397261	-0.3100215	0.29443175	0.04267879	0.12374723
Sap30	861.979119	-1.429009	-0.515015	0.10429305	1.12E-07	1.73E-06
Sap30bp	986.975884	1.02693221	0.03834095	0.09285713	0.64842679	0.79585582
Sap30l	460.616533	1.06456837	0.09026861	0.0956533	0.29259676	0.48963613
Sapcd1	2.11404099	-1.0070343	-0.0101129	0.2143301	0.6788069	0.81510278
Sapcd2	385.923305	-1.0456249	-0.0643654	0.11207863	0.50031993	0.68583839
Sar1a	2387.552	1.03623208	0.05134716	0.07078613	0.44248546	0.63968387
Saraf	929.641355	-1.1633267	-0.2182563	0.09229632	0.00898703	0.03577495
Sardh	4.73662803	-1.0377271	-0.0534271	0.21987541	0.25345681	0.44500847
Sars2	404.873039	-1.0018662	-0.0026898	0.11638022	0.97703649	0.98941236
Sart1	4215.79311	1.04405286	0.06219475	0.06177182	0.2925776	0.48963613
Sart3	3182.79169	-1.0687349	-0.0959041	0.05277349	0.06026779	0.16060546
Sash1	4745.52411	1.09454495	0.13033121	0.04786616	0.00535137	0.02335642
Sass6	972.122504	1.01839685	0.02629986	0.10039968	0.74138618	0.85589602
Satb2	622.13868	1.0542246	0.07618226	0.08790228	0.3419119	0.5415296
Sav1	3466.79683	1.03564047	0.05052325	0.05584796	0.34895046	0.54867502
Saxo2	15.5897546	1.03804662	0.05387124	0.21169542	0.44368123	0.6404729
Sbds	423.676435	-1.1750379	-0.2327073	0.134862	0.03476014	0.105466
Sbf1	3054.51389	1.06009423	0.08419251	0.0691682	0.19806443	0.37897017
Sbf2	3530.8322	1.26464706	0.33873481	0.05473741	1.83E-10	4.40E-09
Sbk1	407.987428	-1.0172075	-0.024614	0.10552772	0.7880006	0.88115282
Sbno1	5024.4518	-1.0017917	-0.0025826	0.04821452	0.95309669	0.97743725
Sbno2	3099.62818	-1.0413935	-0.0585153	0.08094966	0.43366013	0.63155823
Sc5d	3825.68433	-1.0451132	-0.0636592	0.05903012	0.25508987	0.4467124
Scaf1	3275.38088	1.01968541	0.02812413	0.06568767	0.65424628	0.79983482
Scaf11	11189.5832	1.0488739	0.06884124	0.04841617	0.14544483	0.30503506
Scaf4	3418.64068	-1.123216	-0.1676354	0.05317072	0.00109499	0.00628722
Scaf8	3594.77091	-1.0627178	-0.0877586	0.05295417	0.08614399	0.20935391
Scai	191.116128	1.20833239	0.27301737	0.18846932	0.04110263	0.12010144
Scamp1	489.8745	1.12850208	0.17440908	0.10272873	0.05441065	0.14823488
Scamp2	1900.73134	1.06270997	0.08774792	0.08018699	0.23736051	0.42680911
Scamp3	867.439536	1.18359942	0.24318089	0.10141788	0.00709272	0.02937529
Scamp4	143.579107	-1.1102012	-0.1508212	0.16524695	0.19879309	0.3799671
Scamp5	1150.06156	1.11560083	0.15782092	0.07626133	0.02636654	0.08492541
Scand1	4.45792049	-1.0187552	-0.0268074	0.21415974	0.49324518	0.6799904
Scap	3056.88589	1.11954869	0.16291727	0.07120361	0.01495008	0.05439095
Scaper	2177.14641	1.24248648	0.31323015	0.06724943	8.00E-07	1.05E-05
Scara3	522.164924	-1.2107927	-0.2759519	0.10500358	0.00308841	0.01494054
Scarb1	1623.2497	1.02910569	0.04139116	0.07207475	0.54266303	0.72036408
Scarb2	2387.97268	1.12303575	0.16740385	0.06124509	0.00425186	0.01943145
Scarf1	36.0585286	-1.0246223	-0.0350922	0.20012377	0.66057438	0.80353741
Scarf2	630.142742	1.71411523	0.7774641	0.09970035	5.55E-16	2.43E-14
Scd1	11640.2925	-1.0476992	-0.0672246	0.04723872	0.14666045	0.30677995
Scd2	11351.4311	1.05386192	0.07568585	0.07846962	0.29971846	0.49758858
Scd3	13.3605729	1.03441932	0.04882112	0.21453793	0.40514905	0.60453807
Scfd1	1275.47054	1.08452707	0.11706606	0.07120912	0.08094613	0.19963802
Scfd2	675.708247	1.18969497	0.25059172	0.08859125	0.00201284	0.01047723
Sclt1	647.971294	1.16387721	0.21893887	0.08720844	0.00613433	0.02603737
Scly	460.986777	-1.1471721	-0.1980818	0.10277312	0.02891437	0.09093865
Scmh1	328.488954	1.04471298	0.06310664	0.10236768	0.48393322	0.67307243

Scn1a	228.460872	1.08632952	0.11946179	0.13245288	0.25983502	0.45214651
Scn2a	576.244603	-1.3307051	-0.4121909	0.10353981	1.37E-05	0.00013753
Scn3a	904.683423	-1.9005614	-0.9264256	0.11945718	6.75E-16	2.92E-14
Scn4a	4.16837023	-1.0759897	-0.1056642	0.25255741	0.01142389	0.04381437
Scn5a	2.22946725	-1.0207262	-0.0295959	0.21705125	0.23298079	0.42235768
Scn7a	8.02902317	1.02325495	0.03316565	0.2131833	0.49762547	0.68402173
Scn8a	358.417997	1.14682774	0.19764871	0.11829363	0.04938768	0.13786363
Scn9a	2.28076219	1.0217133	0.03099042	0.21616952	0.34282487	0.54230509
Scnm1	1263.6332	1.08895137	0.12293953	0.07797886	0.08971163	0.2156918
Scnn1a	37.8371699	-1.1536029	-0.2061467	0.2837487	0.08943984	0.21532124
Sco1	584.784653	-1.1070098	-0.146668	0.08769946	0.0663415	0.17276574
Sco2	131.092888	-1.0386687	-0.0547355	0.1581416	0.61581568	0.77415101
Scoc	231.750593	1.04820713	0.06792382	0.15297817	0.53874935	0.71739908
Scp2	2473.67473	1.04039558	0.05713217	0.06803216	0.37733146	0.57936186
Scpep1	2402.73911	1.1283079	0.17416082	0.06781427	0.00670349	0.02798947
Scrib	3390.34777	1.1308736	0.17743769	0.05801465	0.00144518	0.00788781
Scrn3	962.401681	-1.0657506	-0.0918698	0.1024249	0.30678069	0.50518841
Scyl1	1865.20203	1.36538346	0.44930618	0.06891787	1.30E-11	3.63E-10
Scyl3	284.131359	1.07689467	0.10687715	0.11820671	0.28362007	0.47981638
Sdad1	2835.95131	-1.0578477	-0.081132	0.06476801	0.1879594	0.3657542
Sdc2	100.534595	1.84989171	0.88744082	0.29370347	0.00014076	0.0010977
Sdc4	6985.20541	-1.5524365	-0.6345343	0.04643611	1.86E-43	3.61E-41
Sdcbp	2480.09799	-1.11886	-0.1620296	0.06353986	0.00727757	0.03003404
Sdcbp2	19.7514376	-1.0121712	-0.0174533	0.20166356	0.8093535	0.89580758
Sdccag8	737.55166	1.10704958	0.14671984	0.08525447	0.0604627	0.16095743
Sde2	1002.16929	-1.1804499	-0.2393369	0.07501077	0.00066428	0.004135
Sdf2	531.737098	1.27311047	0.34835761	0.09973181	0.00012643	0.00099977
Sdf2l1	713.271409	1.06531897	0.09128545	0.09825279	0.29619507	0.49378695
Sdha	10124.365	1.08887998	0.12284494	0.04427133	0.00465672	0.02091408
Sdhaf2	2687.89155	-1.0449292	-0.0634052	0.05545478	0.23555226	0.42535389
Sdhaf3	101.404384	1.00553411	0.00796203	0.16878116	0.94121424	0.97139497
Sdhaf4	97.815418	1.06538926	0.09138064	0.16097242	0.41679821	0.61568093
Sdhb	3393.60041	-1.088399	-0.1222075	0.06969232	0.06282895	0.16577525
Sdhd	1264.80272	1.11265039	0.15400035	0.09643283	0.07383709	0.186522
Sdk2	390.395023	1.88885633	0.91751297	0.12937009	9.62E-14	3.44E-12
Sdsl	83.206215	1.02969787	0.04222109	0.16476914	0.6963482	0.8265586
Sec11a	1647.52761	-1.0329573	-0.0467807	0.0678193	0.46666263	0.65954153
Sec11c	287.094758	1.03349552	0.04753213	0.11522099	0.62707921	0.78218883
Sec13	2901.50188	1.06981251	0.09735798	0.07662002	0.1730781	0.34572439
Sec14l1	2754.54423	1.193692	0.25543063	0.0737028	0.00023023	0.00168146
Sec16a	3607.38466	-1.0536884	-0.0754483	0.05145699	0.12998479	0.28122027
Sec16b	274.830677	1.20373094	0.26751296	0.1337296	0.01583825	0.05694303
Sec22a	427.761066	1.11368522	0.15534152	0.10719654	0.09561343	0.22564627
Sec22b	2774.82226	1.03339612	0.04739337	0.05463656	0.37017597	0.57159525
Sec22c	135.899497	1.62719484	0.70238701	0.21550483	8.65E-05	0.00071611
Sec23a	1960.61708	1.22145512	0.28860085	0.06043204	6.77E-07	8.99E-06
Sec23b	3713.74207	-1.0965764	-0.1330064	0.05748396	0.01596447	0.05726543
Sec23ip	4168.29893	1.1371773	0.18545721	0.04883691	9.20E-05	0.00075225
Sec24a	1212.15496	1.13560929	0.18346655	0.07474963	0.00870944	0.03493046
Sec24b	2171.92144	-1.0177175	-0.0253372	0.05349647	0.57087873	0.73973875

Sec24d	3961.87771	1.30516896	0.38423658	0.05186451	3.15E-14	1.15E-12
Sec31a	5412.18936	1.19038986	0.25143414	0.05462127	1.91E-06	2.33E-05
Sec31b	22.2946472	1.02068068	0.02953159	0.19357213	0.7406323	0.85573192
Sec61a1	3253.56281	-1.0910772	-0.1257532	0.06571677	0.0433957	0.12537187
Sec61a2	406.492386	1.28437372	0.36106505	0.15008003	0.00347638	0.01648737
Sec62	4859.01805	1.12158412	0.16553783	0.05619767	0.00222142	0.01138254
Sec63	5643.78517	-1.0962694	-0.1326023	0.06078735	0.02256029	0.07557463
Secisbp2	658.710532	1.26396579	0.33795742	0.09042686	5.25E-05	0.00045749
Secisbp2l	3162.06774	1.08165695	0.11324302	0.05150219	0.0232394	0.07731634
Sel1l	4292.45322	1.63145711	0.70616106	0.06328038	6.57E-30	7.07E-28
Sel1l3	1651.50586	-1.0513973	-0.0723079	0.06104516	0.2155458	0.40179594
Selenof	886.988524	1.03714657	0.05261979	0.09884466	0.5654766	0.73675297
Selenoh	165.150596	1.23476406	0.3042354	0.19305824	0.02716682	0.08674152
Selenoi	436.419403	-1.2581127	-0.3312611	0.13801794	0.0041102	0.01889865
Selenom	341.486576	1.21517107	0.28115943	0.13262203	0.01112415	0.04280574
Selenon	1348.56623	1.26638888	0.34072049	0.0721501	6.61E-07	8.80E-06
Selenoo	522.336443	1.05126023	0.07211984	0.10311327	0.42637938	0.62465442
Selenos	263.17752	-1.0346747	-0.0491773	0.14852021	0.64937582	0.79663899
Selenot	668.76021	-1.0783022	-0.1087615	0.0953952	0.20274289	0.38578701
Sem1	2623.57167	-1.1225788	-0.1668167	0.07821347	0.02164568	0.07312267
Sema3a	148.56872	-1.760099	-0.8156565	0.20115352	3.56E-06	4.06E-05
Sema3b	3164.64754	-1.0351444	-0.049832	0.06465024	0.41806553	0.61705489
Sema3c	12620.5888	1.79701887	0.84560556	0.05320333	6.23E-58	2.28E-55
Sema3e	3657.40867	-1.0320243	-0.045477	0.06839388	0.48315488	0.67255597
Sema3f	4.86004708	-1.0332928	-0.0472491	0.21832224	0.2822195	0.47814202
Sema4b	833.863086	-1.1274175	-0.1730219	0.08799658	0.03074381	0.09568228
Sema4c	766.178476	1.19321365	0.25485239	0.09958441	0.0042809	0.01953506
Sema4g	1.68802391	1.02395964	0.03415885	0.21833042	0.15683241	0.32185568
Sema6b	155.206711	-1.0133616	-0.0191491	0.15345791	0.85760934	0.92418115
Sema6c	148.736918	1.32404413	0.40495121	0.21471523	0.00876405	0.03510871
Sema7a	11.9071975	-1.0240394	-0.0342713	0.20588828	0.62216571	0.77824473
Senp1	6257.57984	1.04320867	0.06102777	0.05342447	0.23800145	0.42772147
Senp2	3432.56098	-1.0075481	-0.0108488	0.05261217	0.83099768	0.9092919
Senp3	3133.81731	1.38435693	0.46921596	0.06183855	5.80E-15	2.32E-13
Senp5	1836.00192	-1.0579827	-0.081316	0.06357915	0.17941211	0.35456405
Senp6	5124.68182	1.05616722	0.07883827	0.0634594	0.19212587	0.37085516
Senp7	1291.5341	1.09177635	0.12667735	0.07851858	0.08215886	0.20173568
Senp8	168.696253	1.27435715	0.34976966	0.17880147	0.01041304	0.0405567
Sephs1	951.271933	-1.1024506	-0.140714	0.07380094	0.04152973	0.1210457
Sephs2	3458.4402	-1.2972397	-0.3754451	0.05663089	8.83E-12	2.52E-10
Sepsecs	440.760934	1.03303691	0.0468918	0.09031512	0.56804376	0.73836894
Septin1	55.0148598	1.14742867	0.19840447	0.24448349	0.11755921	0.26194703
Septin10	1574.68948	-1.020371	-0.0290938	0.07475348	0.67738934	0.81401101
Septin11	7279.50644	-1.0254149	-0.0362078	0.05086753	0.46397113	0.65728516
Septin14	4.65217714	-1.0723315	-0.100751	0.24951727	0.00901629	0.03586917
Septin3	340.022898	2.64221025	1.40174527	0.1307988	4.93E-28	4.58E-26
Septin4	81.9120472	1.14898618	0.20036145	0.21851167	0.12031173	0.2662739
Septin6	3803.24373	1.29414843	0.37200309	0.05423744	1.79E-12	5.60E-11
Septin8	2439.5156	1.10825216	0.14828618	0.07325393	0.03084833	0.09589109
Septin9	12283.0894	-1.0135356	-0.0193968	0.04388549	0.65080013	0.79746983

Serac1	405.019399	1.14564325	0.19615787	0.10963987	0.03936872	0.11630843
Serf1	95.9719147	1.11666726	0.15919936	0.20280709	0.19087989	0.36962885
Serf2	3085.86561	1.08250614	0.11437521	0.07835457	0.1158552	0.25923233
Serhl	118.518577	1.00460306	0.00662557	0.14883827	0.95248191	0.97696319
Serinc1	3943.59454	-1.087907	-0.1215553	0.07060515	0.06691581	0.17380202
Serinc2	855.372131	1.00032165	0.00046397	0.09079874	0.99633307	0.9984375
Serinc3	5021.23795	1.21113108	0.27635502	0.05981753	1.54E-06	1.92E-05
Serinc5	1356.97752	-1.3703397	-0.4545336	0.07075329	2.40E-11	6.52E-10
Serp1	60.5153244	-1.1847279	-0.2445557	0.24531452	0.07577204	0.19023208
Serp2	8.60896904	-1.0155896	-0.0223176	0.20811942	0.70061477	0.82970095
Serpinb8	1526.39644	-1.0309149	-0.0439253	0.06561805	0.48110931	0.67135188
Serpine1	6820.14553	2.08581732	1.06061281	0.07668717	1.22E-44	2.61E-42
Serpinf1	138.409575	2.25249169	1.17152178	0.22324547	8.82E-09	1.62E-07
Serpinf2	5.11648837	-1.0420093	-0.0593681	0.22353121	0.15082093	0.31262397
Serping1	3.35626597	1.01060978	0.01522604	0.21342835	0.65943824	0.80260369
Serpinh1	8341.52209	-1.053107	-0.074652	0.0545351	0.15537865	0.31948644
Serpini1	173.926397	1.07653827	0.1063996	0.15809092	0.34775492	0.54739991
Sertad1	215.163535	-1.0671576	-0.0937732	0.14756657	0.39510702	0.59587014
Sertad2	1274.57667	1.15130273	0.20326724	0.06945812	0.00194714	0.01019742
Sertad3	93.5007018	1.08094104	0.11228783	0.17940654	0.33329317	0.53308597
Sesn1	849.033899	1.15599628	0.20913676	0.08693148	0.00852455	0.03429631
Sesn2	665.098763	1.04026918	0.05695689	0.08335266	0.45827029	0.65241637
Sestd1	1664.13167	-1.1498869	-0.2014919	0.07512868	0.00411743	0.01892513
Setd1b	867.378215	-1.0399739	-0.0565473	0.1020434	0.53650002	0.7153706
Setd2	9032.43762	1.16305561	0.21792008	0.05923973	0.00013418	0.0010526
Setd3	2291.85187	-1.0437468	-0.0617718	0.07815085	0.39507554	0.59587014
Setd4	293.620014	1.15725711	0.21070942	0.11683743	0.03494327	0.10584613
Setd5	6568.48975	-1.1819163	-0.2411278	0.04467492	3.35E-08	5.63E-07
Setd6	314.061653	1.02580936	0.03676265	0.1035258	0.68598238	0.8196191
Setd7	1017.55183	-1.1746418	-0.2322209	0.07670223	0.00118665	0.00670237
Setdb1	2385.78312	1.02926386	0.04161288	0.0541378	0.42665543	0.62469495
Setdb2	523.554852	-1.068292	-0.095306	0.10598841	0.30585474	0.50398755
Setmar	69.8165789	-1.0334193	-0.0474257	0.17846172	0.64328898	0.79213033
Setx	6236.52674	-1.0225382	-0.0321548	0.04725794	0.48657832	0.67514677
Sez6	3.23445933	1.00763501	0.01097315	0.21373994	0.71098124	0.83687123
Sf1	7274.18904	-1.0982192	-0.135166	0.06722977	0.03364612	0.1028656
Sf3a1	7074.27084	-1.0818398	-0.1134868	0.04551067	0.01066394	0.04144572
Sf3a2	1624.93085	-1.0868131	-0.1201039	0.08323639	0.11664804	0.26047865
Sf3a3	5736.27424	-1.1670559	-0.2228736	0.06267004	0.00019283	0.00144874
Sf3b1	9998.26885	-1.0733255	-0.1020876	0.04585444	0.02266289	0.07579938
Sf3b2	13759.9864	1.05699902	0.07997404	0.05047851	0.10289251	0.23838906
Sf3b3	10383.01	1.00763908	0.01097898	0.04768026	0.81476731	0.89951701
Sf3b4	2952.22697	-1.1297806	-0.1760426	0.05305387	0.00059527	0.00376746
Sf3b5	667.347792	-1.1329355	-0.1800657	0.10283143	0.0469415	0.13263923
Sf3b6	1673.631	1.03421736	0.04853943	0.06749432	0.44920716	0.64475161
Sfi1	422.215565	1.27658872	0.3522938	0.1105524	0.00036575	0.00247116
Sfmbt1	1100.13341	1.25202079	0.32425852	0.07943253	1.37E-05	0.00013714
Sfmbt2	2076.71991	1.06599945	0.0922067	0.07609635	0.19470996	0.37467848
Sfpq	12110.9559	-1.2304388	-0.2991729	0.06878456	4.79E-06	5.31E-05
Sfrp4	104.104051	1.15361756	0.20616503	0.21142991	0.11214859	0.25330611

Sfswap	2371.80391	1.07564517	0.10520224	0.06523341	0.08987101	0.21591291
Sft2d2	136.056264	1.1509321	0.20280273	0.19780899	0.11386828	0.2558591
Sfxn1	4660.37057	-1.1778772	-0.2361892	0.06188138	6.55E-05	0.00055944
Sfxn2	413.71792	1.24403673	0.31502909	0.12655036	0.00364219	0.01714048
Sfxn3	2611.74668	1.25171798	0.32390955	0.06455027	1.65E-07	2.45E-06
Sfxn5	30.4755313	-2.5520767	-1.3516717	0.46380383	0.00015481	0.00119393
Sgca	3.26736377	1.00782715	0.01124823	0.21203154	0.77841092	0.87661719
Sgce	794.889967	1.08734982	0.12081616	0.07933573	0.09949662	0.23207424
Sgip1	58.5661403	-1.0354818	-0.0503022	0.1845217	0.61889468	0.7761173
Sgk1	1208.71939	-2.9171269	-1.5445482	0.08015065	6.74E-84	6.17E-81
Sgk3	821.108985	-1.1454589	-0.1959257	0.09626232	0.02315483	0.07712256
Sgms1	1149.14198	-1.2278427	-0.2961258	0.07750038	4.60E-05	0.00040577
Sgms2	1166.39873	-1.6231674	-0.6988118	0.08597854	4.50E-17	2.17E-15
Sgo1	1288.07536	1.17613106	0.23404883	0.07797522	0.0012945	0.00718483
Sgpl1	1586.19466	-1.0362954	-0.0514352	0.0645469	0.40241782	0.60221639
Sgpp1	2007.16942	-1.1393353	-0.1881924	0.06633602	0.0027855	0.01377945
Sgsh	1741.00103	1.41934285	0.50522312	0.06553915	1.97E-15	8.27E-14
Sgsm2	290.2582	1.0230496	0.03287609	0.10295069	0.7162714	0.84070338
Sgsm3	862.424624	1.01841291	0.02632261	0.0861212	0.7337939	0.8512089
Sgta	1576.01859	1.00894156	0.01284261	0.08193106	0.86618738	0.92774752
Sgtb	1009.97323	-1.0051849	-0.0074609	0.08380463	0.92225007	0.96017745
Sh2b1	1244.41748	1.11665116	0.15917856	0.08091122	0.03313822	0.10157947
Sh2b2	40.9072684	-1.0360052	-0.0510312	0.18255707	0.61673393	0.77454526
Sh2b3	1018.04485	-1.5411375	-0.6239956	0.08817605	1.60E-13	5.54E-12
Sh2d3c	73.5007567	1.50844851	0.59306545	0.3091515	0.0039754	0.01840437
Sh2d4a	2.65043295	-1.0040392	-0.0058156	0.21340606	0.84523599	0.91649509
Sh3bgr	30.8096503	-1.0929891	-0.128279	0.24081807	0.16630299	0.33535617
Sh3bgrl	7355.1111	-1.0554554	-0.0778656	0.05880553	0.16777101	0.33775682
Sh3bgrl2	177.364861	-1.8397803	-0.8795335	0.1864503	1.73E-07	2.58E-06
Sh3bgrl3	3212.2017	-1.0194741	-0.0278252	0.07900841	0.7042202	0.83189421
Sh3bp2	89.1443498	-1.4346635	-0.5207123	0.25986409	0.00408722	0.01882677
Sh3bp4	1066.558	-1.2032075	-0.2668855	0.0713839	7.54E-05	0.00063469
Sh3bp5	928.389468	1.36164329	0.44534881	0.08537853	3.49E-08	5.79E-07
Sh3d19	3739.05996	1.13482508	0.18246994	0.06286027	0.00233934	0.01190584
Sh3d21	10.7018616	-1.0258777	-0.0368587	0.2129128	0.48551091	0.6744816
Sh3gl1	957.77354	-1.1225634	-0.1667969	0.07558399	0.01793795	0.06307579
Sh3gl2	2.224797	-1.0342299	-0.0485569	0.22272908	0.03078792	0.095773
Sh3glb1	4035.05582	1.07360352	0.10246131	0.06735925	0.10891414	0.24803379
Sh3glb2	1381.50219	1.09257658	0.1277344	0.0793036	0.08206149	0.20165119
Sh3kbp1	1329.85235	1.26492118	0.33904749	0.07651083	2.73E-06	3.21E-05
Sh3pxd2a	4682.23505	-1.1156758	-0.1579179	0.06123512	0.00676907	0.02823567
Sh3pxd2b	5661.52668	-1.3926104	-0.4777917	0.05477788	4.77E-19	2.70E-17
Sh3rf1	2553.58031	1.120229	0.16379369	0.06988371	0.01291568	0.04827288
Sh3rf2	312.394416	2.41854447	1.27413906	0.17188842	8.41E-15	3.28E-13
Sh3rf3	40.4327322	-7.7091733	-2.9465762	0.37795472	4.13E-16	1.83E-14
Sh3tc1	3.99216978	-1.0133836	-0.0191803	0.21181566	0.66603313	0.80656876
Sh3yl1	370.845595	1.11915265	0.16240683	0.10158161	0.07042668	0.17994121
Shank3	163.363307	1.16845647	0.22460399	0.19772164	0.08535294	0.20771252
Sharpin	155.996197	-1.0383355	-0.0542726	0.14919081	0.61726814	0.77476042
Shb	334.099072	-1.0401701	-0.0568195	0.11111054	0.55038865	0.72692841

Shc1	1334.63992	-1.0385389	-0.0545552	0.07148972	0.41753925	0.61649121
Shc4	8.56091527	1.00369138	0.00531572	0.21073823	0.90624937	0.95137762
Shcbp1	3289.18713	1.066214	0.09249703	0.06129856	0.11510376	0.25804675
Shd	4.76300315	-1.0030333	-0.0043695	0.21203284	0.90468089	0.95022649
Shf	910.584068	-1.1482081	-0.1993841	0.08148635	0.00804298	0.03260461
Shisa2	1.66287669	-1.0009168	-0.0013221	0.21366361	0.96055855	0.98137498
Shisa4	14.8358336	1.18149328	0.24061143	0.42086591	0.01850952	0.06448382
Shisa5	394.607921	1.21866218	0.28529826	0.11452006	0.00434877	0.01974751
Shisa7	14.9310609	1.00179832	0.0025921	0.20114786	0.97330053	0.98781495
Shkbp1	249.244346	1.07347238	0.10228508	0.13542239	0.34412525	0.54342565
Shld1	80.0129915	1.07448804	0.10364942	0.18084268	0.36076023	0.5610541
Shld2	646.186625	-1.3048953	-0.383934	0.0985738	2.17E-05	0.00020524
Shmt1	1140.47639	-1.0694962	-0.0969314	0.08275623	0.2025097	0.38540324
Shmt2	7079.68196	1.02341238	0.03338759	0.0552457	0.53209496	0.71201675
Shoc2	3030.37818	1.11024297	0.15087544	0.06012724	0.00878989	0.03517602
Shox2	2504.84712	1.01593211	0.022804	0.07516573	0.74678229	0.85950415
Shpk	59.0751701	1.11782234	0.16069091	0.21078865	0.18547932	0.3626912
Shprh	3760.46834	-1.0228833	-0.0326415	0.04941958	0.49851813	0.68452928
Shq1	996.693636	-1.1970576	-0.2594926	0.0879429	0.00129938	0.00720878
Shroom3	1549.35271	-1.8475911	-0.8856455	0.0644021	4.13E-44	8.26E-42
Shroom4	3858.11736	1.147001	0.19786665	0.06176382	0.00079473	0.00481576
Shtn1	281.347726	-1.1667252	-0.2224648	0.15438134	0.06073919	0.16159274
Siae	1459.53855	1.30151237	0.38018903	0.06875047	7.99E-09	1.48E-07
Siah2	488.816972	-1.3123629	-0.3921667	0.12515098	0.00034257	0.00234045
Sidt2	1984.16404	1.05991057	0.08394254	0.0929332	0.31695871	0.51591373
Sigmar1	1930.81898	-1.0452321	-0.0638233	0.07899233	0.38360593	0.58485979
Sik1	1290.54498	1.0220233	0.03142809	0.08758276	0.69566183	0.82612041
Sik2	1127.26238	-1.0819301	-0.1136073	0.07288055	0.09527931	0.22518119
Sik3	3553.95361	1.22027994	0.28721215	0.05508785	7.14E-08	1.14E-06
Sike1	578.343569	-1.0601585	-0.08428	0.1068424	0.36457385	0.56560386
Sil1	395.632193	1.27361652	0.34893096	0.1175545	0.00075266	0.00460216
Sin3a	8236.81572	-1.0026078	-0.0037574	0.04510638	0.94455578	0.97295716
Sin3b	1860.88756	1.12912385	0.17520374	0.09539202	0.0404359	0.11866756
Sinhcaf	1199.4286	-1.1111887	-0.1521038	0.0690141	0.01954228	0.06731287
Sipa1	614.042385	-1.1025552	-0.1408509	0.0962739	0.10129709	0.23528845
Sipa1l1	6220.72634	-1.0216331	-0.0308771	0.04716032	0.50294764	0.68781798
Sipa1l2	733.742385	-1.8880662	-0.9169093	0.10030125	4.29E-21	2.73E-19
Sipa1l3	1893.37583	-1.3111583	-0.3908419	0.07197399	1.29E-08	2.33E-07
Sirt2	777.579085	1.26703558	0.34145704	0.10479544	0.00029662	0.00206617
Sirt3	99.042182	1.12203468	0.16611727	0.195466	0.17553686	0.3491115
Sirt5	142.690052	1.06687194	0.09338702	0.14790393	0.39756324	0.59774473
Sirt6	178.04148	-1.0601065	-0.0842092	0.15447371	0.45354267	0.64835192
Sirt7	682.35016	-1.031322	-0.0444948	0.09365616	0.59701677	0.75976405
Sis	4.88560897	1.01801126	0.02575352	0.21350211	0.538329	0.71691387
Siva1	542.727488	1.11720714	0.1598967	0.14553249	0.155667	0.31987397
Six4	975.59607	-1.2064958	-0.2708229	0.08435551	0.00051214	0.00332517
Six5	35.7436063	1.1835989	0.24318026	0.33349375	0.05692277	0.15351172
Ska1	879.661252	-1.0515295	-0.0724893	0.08868714	0.37003113	0.57146175
Ska3	919.646784	-1.094335	-0.1300544	0.08682931	0.10056589	0.23405687
Skap2	736.494877	-2769.9861	-11.435663	0.97250331	2.02E-32	2.40E-30

Ski	4611.50399	1.02469776	0.03519845	0.05536462	0.49283298	0.67964156
Skida1	56.3714129	1.21869567	0.2853379	0.30361637	0.05284556	0.14495753
Skil	3294.16825	1.14022671	0.1893207	0.06151543	0.00127656	0.00710062
Skiv2l	718.16165	1.22483676	0.29258949	0.11677715	0.0039174	0.01816867
Skp1a	3986.29351	-1.0208164	-0.0297234	0.06455501	0.62941803	0.78348508
Skp2	738.611291	-1.0994235	-0.1367472	0.09623226	0.11143283	0.25237098
Slain2	6853.10327	-1.0091598	-0.0131547	0.05526221	0.80486567	0.89243739
Slc10a7	401.668926	-1.3774843	-0.4620359	0.12426007	3.21E-05	0.0002934
Slc11a1	12.2895468	-2.4173136	-1.2734047	0.93041355	0.00401469	0.01856611
Slc11a2	636.8566	-1.3320084	-0.4136032	0.10839734	2.63E-05	0.00024411
Slc12a2	1330.93984	-1.3793628	-0.464002	0.08296668	3.75E-09	7.33E-08
Slc12a4	1359.28513	1.01481096	0.02121101	0.09049691	0.79692361	0.8872407
Slc12a5	167.487707	1.33888775	0.42103501	0.18049576	0.0030786	0.01490907
Slc12a7	2154.42201	1.23754836	0.3074849	0.06572106	9.86E-07	1.27E-05
Slc12a8	1.91040432	1.0209159	0.02986402	0.2178261	0.14354746	0.30219276
Slc12a9	273.09166	-1.0963279	-0.1326794	0.13258728	0.21186792	0.39771806
Slc13a3	3.04666761	1.0257338	0.03665636	0.21749529	0.27274449	0.46853385
Slc14a1	27.1488126	-32.673878	-5.0300658	0.71623669	5.88E-13	1.94E-11
Slc15a3	113.308168	-1.1076374	-0.1474857	0.18942539	0.21506899	0.40119903
Slc15a4	123.185895	1.19581182	0.25799037	0.20574339	0.05822744	0.15627352
Slc16a1	9822.2975	-1.4142367	-0.5000236	0.04949817	8.71E-25	6.88E-23
Slc16a6	214.951668	1.14168336	0.19116258	0.16701854	0.11362201	0.25558259
Slc16a7	34.2682091	1.05480025	0.07696982	0.20417017	0.41375642	0.61245895
Slc17a5	804.572543	-1.0641557	-0.0897093	0.0978004	0.30034777	0.4981169
Slc17a6	92.8872967	-6.5619208	-2.7141182	0.26291057	4.99E-26	4.26E-24
Slc18b1	1646.26537	1.04042566	0.05717388	0.05961353	0.31884968	0.51733343
Slc19a2	652.834544	-1.203299	-0.2669952	0.10471998	0.00406289	0.01875254
Slc1a2	21.7273033	-3.4976907	-1.8064027	0.57949581	6.89E-05	0.00058529
Slc1a3	4936.38375	1.52489318	0.60870819	0.06250547	2.38E-23	1.73E-21
Slc1a4	671.745356	1.51782539	0.60200583	0.09882406	1.30E-10	3.19E-09
Slc1a5	3225.76044	1.00784213	0.01126966	0.05974633	0.84489532	0.916436
Slc20a1	5882.93779	-1.2350143	-0.3045277	0.05813791	5.50E-08	8.95E-07
Slc20a2	682.930585	1.16313607	0.21801988	0.09405648	0.01018621	0.03986718
Slc22a15	400.122897	1.00592834	0.00852754	0.11439668	0.93095271	0.96554969
Slc22a17	103.591575	2.02139125	1.01534859	0.27926456	1.44E-05	0.00014423
Slc22a18	19.3975271	1.09005948	0.12440685	0.24517685	0.14246655	0.30055937
Slc22a23	1461.70654	1.11790304	0.16079506	0.0876624	0.04413963	0.12677772
Slc22a4	103.962983	-1.137419	-0.1857638	0.18476225	0.13472304	0.28854743
Slc22a5	115.178633	1.6777888	0.74656112	0.25921262	0.00026927	0.00191309
Slc23a2	1684.30143	1.07657072	0.10644309	0.06775858	0.09704196	0.22809106
Slc24a1	4.46045331	1.03195882	0.0453854	0.21772644	0.30898962	0.50732594
Slc25a1	1027.62692	1.29459715	0.37250324	0.08532179	3.06E-06	3.57E-05
Slc25a10	1129.14834	1.15908899	0.21299134	0.08954379	0.00896876	0.03572446
Slc25a11	484.071895	1.07847993	0.10899933	0.10199938	0.22463216	0.41308326
Slc25a12	1457.68605	1.18246539	0.24179795	0.07787068	0.00087024	0.00520668
Slc25a13	2038.80908	1.07678273	0.10672718	0.06301825	0.07581068	0.19023208
Slc25a14	325.052624	1.05984663	0.08385551	0.12359775	0.40999684	0.60914632
Slc25a15	116.373176	-1.0723784	-0.100814	0.18008426	0.36807539	0.56972519
Slc25a16	1010.78911	1.12927059	0.17539122	0.08079669	0.01885188	0.06530358
Slc25a17	1687.03216	1.0349983	0.0496284	0.06080178	0.39488793	0.59576837

Slc25a19	533.157028	-1.1057416	-0.1450143	0.10409142	0.11172005	0.25276119
Slc25a20	1976.8176	1.53976556	0.62271071	0.06426096	4.04E-23	2.87E-21
Slc25a22	926.926201	-1.3002763	-0.3788183	0.09006822	6.04E-06	6.53E-05
Slc25a23	77.5751289	1.4962117	0.58131432	0.25541893	0.00190552	0.01002038
Slc25a24	1219.86982	-1.1219901	-0.16606	0.06919901	0.01087273	0.04211922
Slc25a25	422.633832	-1.1988154	-0.2616095	0.13802263	0.02024924	0.06931928
Slc25a26	82.1837499	1.02244147	0.03201826	0.16385626	0.76663362	0.87106819
Slc25a27	82.2934811	1.03434998	0.04872442	0.18234041	0.63627591	0.78819209
Slc25a28	786.614888	1.09943457	0.13676175	0.07574522	0.05295836	0.14523584
Slc25a3	14168.2616	1.00530387	0.00763165	0.04714289	0.87612769	0.93339951
Slc25a30	1303.18721	1.23917569	0.30938075	0.07308423	7.82E-06	8.29E-05
Slc25a33	567.690312	-1.1579946	-0.2116285	0.10673405	0.02343498	0.0778129
Slc25a35	289.412824	1.20432369	0.2682232	0.15450545	0.02662134	0.08551137
Slc25a36	463.530994	-1.01396	-0.0200007	0.10905386	0.83112739	0.90933349
Slc25a37	1683.5117	-1.2996518	-0.3781252	0.06663424	3.40E-09	6.69E-08
Slc25a38	167.985867	1.12195657	0.16601683	0.17257928	0.16749697	0.33731114
Slc25a39	3161.4138	-1.0542784	-0.0762559	0.07055354	0.23723985	0.42677187
Slc25a40	1375.72734	1.07793359	0.1082683	0.08322813	0.15741045	0.32273172
Slc25a42	86.2576792	1.12003765	0.16354723	0.20105579	0.17987733	0.35531666
Slc25a43	112.409032	1.02770863	0.0394313	0.17328975	0.70889006	0.83556144
Slc25a44	467.168478	-1.1180336	-0.1609635	0.11989606	0.10959065	0.24921998
Slc25a45	43.6538365	1.06735291	0.09403727	0.19806552	0.37254587	0.57414546
Slc25a46	229.834294	1.28647489	0.3634233	0.13618949	0.0016922	0.00903967
Slc25a47	15.3717585	1.84135669	0.88076912	0.69063934	0.00573524	0.02468696
Slc25a51	313.337363	-1.0346409	-0.0491301	0.11441837	0.61158547	0.77141206
Slc26a11	85.6992279	-1.025947	-0.0369562	0.16650705	0.72926391	0.84825849
Slc26a2	287.516504	-1.0169953	-0.024313	0.11355566	0.80329255	0.89146475
Slc26a6	413.065204	-1.0690162	-0.0962837	0.12227938	0.34055962	0.5400284
Slc26a8	16.5180706	1.03546646	0.05028082	0.20451935	0.53252095	0.71214157
Slc27a1	128.038135	-1.0161459	-0.0231075	0.15175797	0.82898096	0.90788335
Slc27a3	236.323598	2.34302075	1.22836973	0.20668997	1.85E-10	4.44E-09
Slc27a4	1156.19661	1.11950201	0.16285712	0.07384172	0.01856001	0.06460699
Slc28a3	17.2399871	1.02498521	0.0356031	0.20336816	0.64039738	0.79068702
Slc29a1	2023.87046	1.00147804	0.00213079	0.0833989	0.97903208	0.99034912
Slc29a2	321.474274	-1.0331067	-0.0469893	0.12389724	0.64271918	0.7919616
Slc29a3	163.347528	1.12715366	0.1726842	0.1566749	0.1396095	0.29594535
Slc2a1	1161.26844	-2.2626641	-1.1780224	0.07222582	4.86E-61	2.01E-58
Slc2a13	1603.13452	-1.018055	-0.0258155	0.05650821	0.63260935	0.78561455
Slc2a4	8.565802	1.0644772	0.09014505	0.235134	0.11656391	0.26040874
Slc2a8	141.668694	-1.1320785	-0.178974	0.16656053	0.13505649	0.28911658
Slc2a9	12.7997197	1.99345549	0.99527139	0.82201781	0.00557275	0.024158
Slc30a1	929.767426	-1.0541793	-0.0761203	0.11786122	0.44080324	0.63833253
Slc30a4	5710.9857	-1.1319504	-0.1788108	0.05804888	0.00133246	0.00736043
Slc30a5	2800.20031	-1.0410215	-0.0579999	0.06089	0.3203851	0.51911626
Slc30a6	239.50431	-1.1901234	-0.2511112	0.13197881	0.02180509	0.07354366
Slc30a7	853.121984	1.03136587	0.0445562	0.08722634	0.57644101	0.74377612
Slc30a9	1797.1199	1.00738426	0.01061409	0.08099176	0.88859926	0.94042932
Slc31a1	596.734022	1.01658094	0.02372508	0.09067849	0.7735684	0.87386763
Slc31a2	160.022484	1.30366375	0.38257181	0.18431641	0.00660331	0.02766135
Slc33a1	1012.78945	-1.0075721	-0.010883	0.07081664	0.87001186	0.92966733

Slc35a2	226.920007	1.32839472	0.40968389	0.16815059	0.00246521	0.01246719
Slc35a3	2178.90414	1.07502409	0.10436899	0.06209905	0.07863164	0.19537963
Slc35a4	314.8152	-1.1445193	-0.1947419	0.1822273	0.11766061	0.2620818
Slc35a5	281.603867	-1.0713813	-0.0994721	0.11391775	0.30439603	0.50256561
Slc35b1	1665.90653	1.08445856	0.11697493	0.05998342	0.04190624	0.12186581
Slc35b3	322.277977	-1.0856849	-0.1186054	0.13167031	0.2616108	0.45421989
Slc35b4	466.61636	-1.0202677	-0.0289477	0.10256155	0.75125118	0.862246
Slc35c1	2195.44678	-1.261087	-0.3346678	0.06239983	2.40E-08	4.14E-07
Slc35c2	1676.07089	1.50900455	0.59359716	0.08967295	4.21E-12	1.26E-10
Slc35d2	64.3300114	1.09962172	0.13700731	0.21329062	0.22795024	0.41667274
Slc35e2	1668.50289	-1.1003832	-0.138006	0.06304394	0.02162996	0.07310221
Slc35e3	1476.37971	1.14609548	0.19672724	0.0676511	0.00214861	0.01107148
Slc35e4	103.257902	-1.288979	-0.3662288	0.25070792	0.0208819	0.0710295
Slc35f2	379.494359	1.26383326	0.33780613	0.12942354	0.00228752	0.01168385
Slc35f5	485.222784	1.17774177	0.23602325	0.12044193	0.02124673	0.07202188
Slc35f6	304.737248	-1.1649565	-0.220276	0.12345426	0.03387953	0.10340643
Slc36a1	726.216708	1.10452968	0.14343218	0.0899477	0.07863992	0.19537963
Slc36a4	1305.27985	1.00095447	0.00137635	0.07658129	0.98538727	0.99212536
Slc37a2	2.02216912	-1.0121643	-0.0174434	0.21502693	0.48449615	0.67336397
Slc37a3	402.168679	1.10692485	0.14655729	0.1404269	0.18278977	0.35881728
Slc37a4	313.830951	1.06404665	0.0895614	0.13220227	0.39482289	0.59576837
Slc38a10	7097.73973	1.31931296	0.39978683	0.05881876	2.32E-12	7.15E-11
Slc38a2	7883.89314	-1.2365218	-0.3062876	0.05323757	3.02E-09	6.01E-08
Slc38a6	319.130105	-1.0592338	-0.083021	0.11864501	0.40973481	0.60896889
Slc38a7	1824.6401	-1.0522159	-0.0734308	0.06261988	0.21909331	0.40604533
Slc38a9	266.905993	-1.031985	-0.045422	0.12957104	0.66073453	0.80364692
Slc39a1	362.520505	-1.0296613	-0.0421699	0.14608606	0.69461313	0.82527263
Slc39a10	7857.18792	1.03445116	0.04886554	0.07743894	0.50372378	0.68843504
Slc39a11	1135.00311	1.40880141	0.49446826	0.08171117	2.19E-10	5.15E-09
Slc39a13	1396.01554	-1.0114933	-0.0164868	0.08954425	0.83863676	0.91371188
Slc39a14	1388.03504	-1.1876738	-0.2481387	0.0687049	0.00013661	0.00106894
Slc39a2	1.7437751	1.00990779	0.01422358	0.21543472	0.42619349	0.62458971
Slc39a3	230.912133	1.13556175	0.18340616	0.17254019	0.13191699	0.2839618
Slc39a4	2.45807438	-1.0289007	-0.0411038	0.21960736	0.1457541	0.30538274
Slc39a5	43.8532864	-1.0125213	-0.0179522	0.18583231	0.84866053	0.91853734
Slc39a6	1906.18861	-1.1640398	-0.2191404	0.0722417	0.00126263	0.00704454
Slc39a7	2675.30015	1.09152461	0.12634466	0.06505669	0.04006443	0.11777063
Slc39a8	1867.9183	-1.0856663	-0.1185807	0.09225667	0.15376061	0.31676317
Slc39a9	2118.74361	-1.0962168	-0.1325331	0.06260851	0.02640264	0.0850007
Slc3a1	10.6037836	-1.0018673	-0.0026914	0.20594235	0.96216481	0.98209252
Slc3a2	2855.52205	1.00063511	0.00091598	0.07261758	0.9888516	0.99475334
Slc40a1	199.738357	-1.0862216	-0.1193184	0.14020431	0.27348258	0.46917449
Slc41a1	3285.75522	-1.1090259	-0.149293	0.04860071	0.0015627	0.0084358
Slc41a2	1524.27776	1.67550371	0.74459488	0.06618294	2.24E-30	2.48E-28
Slc41a3	133.286054	1.26958653	0.34435873	0.20561766	0.01769575	0.06234395
Slc43a2	472.424387	-1.1092062	-0.1495276	0.12375447	0.14419693	0.30331079
Slc43a3	2.29637409	-1.0374864	-0.0530925	0.2240605	0.02517979	0.08207459
Slc44a1	1685.76331	-1.0528666	-0.0743227	0.08944718	0.35970047	0.559874
Slc44a2	1234.33361	1.17828813	0.23669237	0.07921671	0.0013288	0.00734973
Slc44a3	7.49046477	1.06713967	0.09374901	0.2403764	0.06238677	0.16481222

Slc45a1	2644.70239	1.38543771	0.47034185	0.07824989	3.15E-10	7.22E-09
Slc45a2	4.38871271	1.0298266	0.04240144	0.21806151	0.27789598	0.47363847
Slc45a4	405.800271	1.06284216	0.08792736	0.12065234	0.38086279	0.58280281
Slc46a1	385.134282	1.26032143	0.33379172	0.1547766	0.00730871	0.03013344
Slc46a3	259.012354	-1.2739261	-0.3492816	0.1406328	0.00300151	0.01459178
Slc48a1	478.495846	1.22623793	0.29423894	0.1154415	0.00346567	0.01644269
Slc49a4	238.583388	1.01183444	0.01697326	0.11615611	0.86323447	0.92665838
Slc4a10	42.070061	-1.0875659	-0.1211028	0.22708803	0.21936405	0.40642948
Slc4a1ap	1801.79856	-1.0168148	-0.0240569	0.06218854	0.68565441	0.81956079
Slc4a2	1561.87044	-1.8582556	-0.893949	0.06999275	1.68E-38	2.53E-36
Slc4a3	171.35058	1.49667623	0.58176216	0.18612835	0.00018407	0.00139603
Slc4a7	4525.74644	-1.3693038	-0.4534426	0.06326979	1.39E-13	4.87E-12
Slc50a1	434.959704	1.13154914	0.17829924	0.10308664	0.04962361	0.13833389
Slc51a	4.63532596	1.03807403	0.05390934	0.22008468	0.24583431	0.43597712
Slc52a2	170.765288	1.25907046	0.33235903	0.22348229	0.02540616	0.08264271
Slc52a3	87.0990883	-1.0052772	-0.0075934	0.17347214	0.93928384	0.97057563
Slc5a5	10.3791724	-1.0566849	-0.0795452	0.22625976	0.2097586	0.39514818
Slc5a6	222.994552	-1.0311963	-0.0443191	0.1347791	0.67318825	0.81109306
Slc66a2	176.33067	1.16224992	0.21692033	0.16796945	0.07819741	0.19483475
Slc6a15	1715.94096	-1.0215317	-0.0307339	0.07835757	0.67268993	0.81086907
Slc6a20b	2.40449808	-1.0140454	-0.0201222	0.21615196	0.26706404	0.46131208
Slc6a4	37.6974259	-1.0168029	-0.0240401	0.19131916	0.78752224	0.8808535
Slc6a6	7913.25709	-1.5228221	-0.6067474	0.04229774	1.32E-47	3.20E-45
Slc6a8	649.23109	1.23922094	0.30943343	0.11041957	0.00153637	0.0083159
Slc6a9	106.32807	1.36931735	0.45345684	0.31414451	0.01332502	0.04946203
Slc7a1	6946.51668	-1.0255819	-0.0364428	0.05215379	0.47102019	0.66276018
Slc7a11	256.192913	1.02401769	0.03424064	0.12569045	0.73869538	0.8542645
Slc7a5	2300.57578	1.03170205	0.04502639	0.08809758	0.57552185	0.74304698
Slc7a6	639.686487	1.01711394	0.0244813	0.08454243	0.75334286	0.86298066
Slc7a6os	351.078558	-1.1451941	-0.1955922	0.1338557	0.07169037	0.18232155
Slc7a7	156.733235	-1.0110918	-0.015914	0.15756592	0.88083277	0.93623198
Slc8b1	306.213792	1.03389571	0.04809066	0.11581202	0.62322834	0.77883907
Slc9a1	1162.751	-1.0633264	-0.0885845	0.10126647	0.32026264	0.51898349
Slc9a3r1	1540.91252	-1.0828376	-0.1148168	0.07768014	0.11122217	0.25203538
Slc9a5	213.837712	1.01244835	0.01784831	0.14919982	0.87066009	0.92983954
Slc9a6	202.827107	-1.0364873	-0.0517025	0.13171375	0.62010171	0.77694668
Slc9a8	421.693782	1.28267775	0.35915877	0.13606081	0.00187593	0.00988509
Slc9a9	384.485002	1.05701664	0.07999809	0.11053811	0.40046544	0.60048721
Slc9b1	2.48191941	-1.0474786	-0.0669208	0.22861939	0.05425654	0.14790941
Slc9b2	26.6042915	1.01328099	0.0190343	0.19556607	0.82186077	0.90384445
Slf1	2403.66893	-1.0535061	-0.0751987	0.06377526	0.216102	0.40218896
Slf2	3330.97088	1.13968991	0.18864134	0.05614971	0.00048828	0.00318475
Slfn2	2923.68247	-1.41202	-0.4977605	0.05708314	4.48E-19	2.56E-17
Slirp	1302.79335	-1.0777086	-0.1079671	0.07757146	0.13451649	0.28834609
Slit2	2.52715168	-1.0219859	-0.0313753	0.21698871	0.26104599	0.45357179
Slitrk3	68.2635114	1.0490197	0.06904178	0.18089406	0.51714831	0.70028223
Slk	10650.7112	-1.2733497	-0.3486286	0.07152374	3.69E-07	5.14E-06
Slmap	5583.97641	1.00562357	0.00809037	0.0408182	0.8402158	0.91430211
Sltm	4406.67746	1.0407751	0.05765835	0.05446598	0.27392737	0.46943272
Slx4	1570.18654	1.07019118	0.09786855	0.06730255	0.12538753	0.27419057

Slx4ip	253.728129	-1.0830719	-0.115129	0.14747385	0.30485058	0.50285037
Smad2	650.870397	1.041517	0.05868638	0.10857587	0.53226322	0.71201878
Smad3	468.783901	-1.0774564	-0.1076295	0.10312524	0.2332291	0.42258342
Smad4	818.403379	-1.0004931	-0.0007112	0.08247911	0.99181836	0.99615366
Smad5	1677.18159	1.06073182	0.08505996	0.06887404	0.19388349	0.373537
Smad6	240.909539	2.47089191	1.3050319	0.15911994	1.40E-17	7.00E-16
Smad7	421.944252	1.61265241	0.68943552	0.11356983	1.22E-10	3.02E-09
Smad9	465.015666	1.77163289	0.82507969	0.12935534	1.42E-11	3.93E-10
Smagp	390.640102	-1.2218282	-0.2890415	0.11906645	0.00489781	0.02173976
Smap1	1152.45824	1.10405529	0.14281242	0.07674052	0.04574836	0.13029885
Smap2	2575.30122	1.03769451	0.05338178	0.05305282	0.29928299	0.49712333
Smarca1	1251.05741	-1.1585896	-0.2123697	0.07332551	0.00202148	0.01050942
Smarca2	1331.17739	1.19843115	0.26114702	0.09788717	0.00306728	0.01486074
Smarca4	11066.159	-1.0358339	-0.0507927	0.04404401	0.24011066	0.42982359
Smarca5	14508.4097	-1.0558338	-0.0783828	0.04988351	0.10509738	0.24179191
Smarcad1	1023.56771	-6.6668482	-2.7370049	0.09648275	3.88E-178	1.24E-174
Smarcal1	1209.17508	1.03811156	0.0539615	0.08814688	0.50377851	0.68843639
Smarcb1	1806.28402	1.03700125	0.05241763	0.06551955	0.40069508	0.60076124
Smarcc1	13352.851	1.00636216	0.00914959	0.04191555	0.82197705	0.90384445
Smarcc2	2834.13515	1.53023401	0.61375229	0.05687138	4.50E-28	4.22E-26
Smarcd1	1699.61556	-1.050214	-0.0706833	0.06975418	0.28297192	0.47909996
Smarcd2	602.266892	-1.0576437	-0.0808537	0.0896111	0.31955509	0.5180335
Smarcd3	100.335056	-1.1022166	-0.1404078	0.20322872	0.22884943	0.41748237
Smarce1	13003.9004	-1.0799682	-0.1109888	0.05328281	0.03109246	0.09650942
Smc1a	7609.57456	-1.1227791	-0.1670741	0.05359367	0.00124702	0.00696352
Smc2	20042.9964	-1.029982	-0.0426192	0.05465061	0.41340812	0.61229715
Smc3	15076.4654	1.02095647	0.02992136	0.06357999	0.62164795	0.77793703
Smc4	16735.6997	1.01430867	0.02049675	0.04959061	0.61950881	0.77658361
Smc5	3634.67699	1.02370048	0.03379366	0.06404357	0.58039469	0.74680766
Smc6	11326.4913	1.28883611	0.36606882	0.05732367	4.10E-11	1.08E-09
Smchd1	12911.0134	1.02912903	0.04142388	0.05323642	0.42434401	0.62273419
Smco2	2.51349456	1.0129941	0.01862577	0.21429465	0.54882643	0.72563893
Smco4	10.7248491	1.03852407	0.05453465	0.2125727	0.42434275	0.62273419
Smcr8	1156.02399	1.02391257	0.03409253	0.08135158	0.65122746	0.79776454
Smdt1	170.047372	1.14736763	0.19832772	0.20067003	0.1218402	0.2684508
Smg1	6082.59944	-1.1664235	-0.2220916	0.04759295	2.02E-06	2.44E-05
Smg5	5036.61692	-1.0387208	-0.0548079	0.05292856	0.28526565	0.48189364
Smg6	452.669444	-1.0397164	-0.05619	0.1158758	0.56503185	0.73647314
Smg7	5623.48498	1.08177447	0.11339976	0.04788827	0.0148439	0.05414303
Smg8	1259.77648	-1.1462279	-0.1968939	0.07460042	0.00476838	0.02128326
Smg9	1069.69986	-1.0293576	-0.0417443	0.06689597	0.5090686	0.69337255
Smim1	8.69005347	1.01775918	0.02539623	0.21294575	0.56572673	0.73677912
Smim10l1	6.66544735	-1.0092383	-0.0132669	0.21025433	0.78126617	0.87751158
Smim11	88.3515647	-1.134857	-0.1825105	0.22152959	0.14320942	0.30167647
Smim12	148.767333	1.06405852	0.08957749	0.15249077	0.42109476	0.6196696
Smim13	41.0872639	-1.0246073	-0.035071	0.19443898	0.69168849	0.82348698
Smim15	1623.56357	-1.0709344	-0.0988701	0.08572837	0.2067119	0.39119212
Smim19	454.926641	1.09639273	0.13276467	0.09908944	0.13170789	0.2836547
Smim20	62.336513	1.08573537	0.11867252	0.19145849	0.29996524	0.49767745
Smim26	201.762261	1.09695083	0.13349886	0.16734314	0.25401612	0.445441

Smim3	196.037627	-2.0099437	-1.0071551	0.1696481	1.95E-10	4.64E-09
Smim4	138.899371	-1.4764221	-0.5621053	0.24308787	0.00187118	0.00986411
Smim7	67.6412763	-1.0808219	-0.1121288	0.20055184	0.30541267	0.50351818
Smim8	235.117831	-1.0139324	-0.0199615	0.12262794	0.84219702	0.9153694
Smndc1	2186.31978	-1.0522294	-0.0734493	0.05849725	0.19119629	0.36980643
Smo	1525.95977	-1.1055419	-0.1447537	0.09896724	0.09931351	0.23181597
Smox	169.057388	-4.6874558	-2.2288051	0.19156936	2.27E-32	2.67E-30
Smpd1	2682.448	1.31947971	0.39996917	0.07947563	1.06E-07	1.64E-06
Smpd2	168.135982	1.0765513	0.10641707	0.13374553	0.31777533	0.51645699
Smpd4	3584.55798	-1.0221863	-0.0316581	0.04979383	0.5129085	0.69679862
Smpdl3b	1.61291912	1.01927794	0.0275475	0.21777851	0.07842424	0.19526036
Smtn	1490.24616	-1.130217	-0.1765998	0.07950087	0.01655033	0.05882622
Smu1	1646.54389	-1.1223929	-0.1665778	0.07551351	0.018152	0.06349731
Smug1	1155.13041	-1.0569898	-0.0799614	0.07942715	0.27716794	0.47277248
Smurf1	5938.22809	1.1643739	0.21955441	0.05193239	1.27E-05	0.00012887
Smurf2	5085.81119	1.19791291	0.26052302	0.05919041	4.62E-06	5.15E-05
Smyd1	33.0251409	-2.488011	-1.3149928	0.52591032	0.0004484	0.00295474
Smyd2	737.005723	1.34512489	0.42774013	0.10905635	1.60E-05	0.0001576
Smyd3	142.56858	1.0561925	0.07887281	0.14620903	0.47230275	0.66385249
Smyd4	962.002493	1.23336175	0.30259601	0.08455112	0.00011476	0.00092111
Smyd5	308.717781	-1.7107514	-0.7746302	0.12050992	1.12E-11	3.17E-10
Snai1	46.5566293	2.03499413	1.02502463	0.37646601	0.00030631	0.00212326
Snai2	573.752269	-1.0356081	-0.0504781	0.08898782	0.53266437	0.71225789
Snap29	2021.92886	-1.0399636	-0.056533	0.0639603	0.35360206	0.55322613
Snap47	154.571242	1.05474894	0.07689964	0.14744947	0.48268326	0.67215703
Snap91	4.71749896	-1.0326905	-0.046408	0.21844263	0.28120188	0.47698571
Snapc2	365.106865	1.13390483	0.18129955	0.13892791	0.1012134	0.23517933
Snapc3	723.814025	-1.0005448	-0.0007858	0.08176376	0.99323789	0.9968955
Snapc4	787.253552	1.05146253	0.07239744	0.09985478	0.41375169	0.61245895
Snapc5	178.48182	1.24057917	0.3110138	0.20656092	0.02955571	0.09256266
Snapin	113.783383	1.03316604	0.04707213	0.15899416	0.66618831	0.80661107
Snd1	7158.60799	-1.0224164	-0.0319829	0.04271982	0.4501059	0.64570677
Snf8	1863.98424	1.14401824	0.19411006	0.07126216	0.00379983	0.01772606
Snip1	1214.12747	-1.1125332	-0.1538484	0.07874455	0.03525125	0.10654053
Snn	17.2480377	1.00603214	0.00867639	0.20322241	0.90067824	0.94858902
Snph	14.2229687	-1.0493761	-0.0695319	0.2145777	0.35714028	0.55717537
Snrk	301.419834	1.26386343	0.33784058	0.13692096	0.00338011	0.01611431
Snrnp200	11732.7793	-1.0984875	-0.1355185	0.04267027	0.00119021	0.00671908
Snrnp25	536.219609	1.04903663	0.06906505	0.1168059	0.48387251	0.67307243
Snrnp27	995.787434	1.02263851	0.03229625	0.10727063	0.70993405	0.83633025
Snrnp35	289.580657	-1.0854338	-0.1182718	0.11860496	0.23632406	0.42594252
Snrnp40	2594.66797	-1.0932308	-0.128598	0.06458687	0.03606883	0.10843504
Snrnp70	4144.35933	-1.1954662	-0.2575733	0.07618413	0.00030812	0.00213005
Snrpa	1806.13595	-1.076818	-0.1067744	0.06762806	0.09534284	0.22521516
Snrpa1	2606.40156	-1.0258076	-0.0367602	0.06356359	0.54432771	0.72190205
Snrpb	4226.71789	1.03493	0.0495332	0.06964183	0.4519929	0.6470374
Snrpd2	513.21346	-1.1006484	-0.1383537	0.13077244	0.19027326	0.36894577
Snrpd3	4690.07487	-1.135987	-0.1839463	0.07335971	0.00748642	0.03073753
Snrpe	367.850678	-1.1451343	-0.1955168	0.13154408	0.06900012	0.17762124
Snrpf	1608.29199	-1.0430777	-0.0608467	0.09654989	0.48172643	0.67176179

Sntb2	306.497347	1.12329138	0.16773221	0.12919547	0.11236882	0.25364661
Snupn	1315.05417	1.01898219	0.02712883	0.07010658	0.68267437	0.81728411
Snw1	3243.1047	-1.1594272	-0.2134122	0.07091856	0.00140621	0.0077212
Snx1	1235.66891	1.14643164	0.19715033	0.06757362	0.00208036	0.01076309
Snx11	212.649763	-1.1095509	-0.1499758	0.15026907	0.18608509	0.36332113
Snx12	609.742324	1.13788435	0.18635393	0.11184886	0.0534773	0.14631445
Snx13	2820.49074	-1.0369908	-0.0524031	0.06522294	0.39820794	0.59829272
Snx14	1091.39455	1.02126474	0.03035691	0.07612598	0.67082907	0.81004995
Snx15	105.820665	-1.0219953	-0.0313885	0.15126597	0.7698073	0.87206102
Snx16	611.710018	-1.1178047	-0.1606681	0.0882118	0.04528276	0.12940133
Snx17	2226.95668	1.09086115	0.12546748	0.07811167	0.08375542	0.20479231
Snx18	691.043989	-1.0900818	-0.1244364	0.10935414	0.18671241	0.36393578
Snx21	1163.69007	1.54203301	0.62483364	0.08178334	2.39E-15	9.96E-14
Snx24	8.67225152	-1.0465129	-0.0655901	0.22223965	0.23784337	0.42755734
Snx25	1166.67677	1.31641483	0.39661418	0.07389875	1.83E-08	3.21E-07
Snx27	2575.79919	1.11107619	0.15195775	0.06374163	0.01233698	0.04656357
Snx29	315.872601	-1.062058	-0.0868626	0.11030164	0.3594525	0.55976009
Snx30	377.949346	-1.0364532	-0.0516549	0.09919352	0.55666595	0.7312227
Snx33	1110.11783	1.0520629	0.07322096	0.0832502	0.33976634	0.53919807
Snx4	3968.51314	-1.031583	-0.0448599	0.05310249	0.38210823	0.58397551
Snx5	3148.58054	-1.3094192	-0.388927	0.05514171	4.22E-13	1.41E-11
Snx6	2490.90555	1.01266379	0.01815526	0.07374222	0.79378002	0.88527965
Snx7	1729.41499	-1.0019222	-0.0027705	0.07147227	0.96643282	0.98363296
Snx8	1849.2229	1.20614786	0.27040678	0.07511105	0.0001271	0.00100439
Snx9	6228.70087	-1.1756193	-0.2334209	0.04868416	8.41E-07	1.10E-05
Soat1	2083.55617	1.00847282	0.01217221	0.08464971	0.87656647	0.93371167
Sobp	227.806982	-2.287995	-1.1940839	0.14853163	5.23E-17	2.52E-15
Socs1	113.691686	-1.179776	-0.238513	0.22765307	0.07988998	0.19775664
Socs2	214.812395	-1.1188276	-0.1619878	0.13792827	0.13824677	0.29388337
Socs3	39.9883388	-1.0303969	-0.0432002	0.18427859	0.65888787	0.80223873
Socs4	639.082645	-1.0646618	-0.0903952	0.11852276	0.36277958	0.56370772
Socs5	536.819881	-1.0363505	-0.051512	0.09958344	0.55918833	0.73280844
Socs6	483.74651	-1.0313749	-0.0445688	0.10489728	0.6260454	0.781413
Socs7	268.002324	1.00903157	0.01297132	0.13800138	0.90373161	0.94993042
Sod1	4030.95476	-1.0289118	-0.0411193	0.05143397	0.41021764	0.60933639
Sod2	959.020146	-1.1046352	-0.14357	0.09127675	0.08055834	0.19906488
Soga1	1626.45034	1.02678581	0.03813526	0.07950431	0.60612931	0.76704121
Son	17496.8849	-1.0300577	-0.0427252	0.04530547	0.33409673	0.53403782
Sorbs1	900.134956	-1.0571317	-0.0801551	0.08603595	0.30841933	0.50664935
Sorbs2	254.857303	1.1159114	0.15822249	0.14684856	0.16529601	0.33404624
Sorbs3	1396.26729	1.05991044	0.08394236	0.07583222	0.23657594	0.42605621
Sord	3607.03953	-1.1442096	-0.1943513	0.05640445	0.00035261	0.00240005
Sorl1	2.86715411	-1.0222656	-0.0317701	0.21798592	0.1515623	0.31376889
Sort1	1540.56765	-1.0616971	-0.0863722	0.06830428	0.18086704	0.356734
Sos1	3503.24178	-1.1202111	-0.1637706	0.05342331	0.00152145	0.00825488
Sos2	2312.15388	1.09966695	0.13706665	0.07609874	0.05353264	0.14642969
Sowahc	244.588869	-1.0797489	-0.1106958	0.12441421	0.27910817	0.47481748
Sox12	214.045274	-1.0259856	-0.0370105	0.15505293	0.72086804	0.84286004
Sox4	189.813127	-1.7272119	-0.7884451	0.18796033	2.04E-06	2.47E-05
Sox5	407.605343	-1.2021735	-0.2656452	0.14116388	0.02053735	0.07007318

Sox7	2.32513676	-1.0288729	-0.0410648	0.22062449	0.04404423	0.12661729
Sox8	35.3190457	2.2409303	1.16409778	0.38705361	0.0001312	0.00103165
Sp1	4652.95345	-1.064325	-0.0899387	0.05472584	0.08832581	0.21340128
Sp2	1476.28977	-1.0660563	-0.0922837	0.0652468	0.13687952	0.29184863
Sp4	533.186367	1.0873815	0.12085819	0.09227507	0.14626171	0.30604581
Spa17	17.1658278	1.01279378	0.01834045	0.20024355	0.80876175	0.89543686
Spaca6	10.4336994	1.04431177	0.06255248	0.22097808	0.25494044	0.44657282
Spag1	4.63705988	-1.007534	-0.0108285	0.21237404	0.77104576	0.8727663
Spag5	8812.87618	1.10229334	0.1405082	0.05276786	0.0059275	0.02533747
Spag7	510.255187	1.0277232	0.03945175	0.09080038	0.63237455	0.78548608
Spag9	10298.8713	1.17603083	0.23392588	0.04854011	7.38E-07	9.76E-06
Sparc	6879.70378	1.48422635	0.56971113	0.08315854	9.56E-13	3.07E-11
Spast	1863.73799	1.06295517	0.08808075	0.05882665	0.11820166	0.26287557
Spata1	28.912579	1.00407417	0.00586584	0.19347111	0.94686046	0.97373406
Spata13	1900.1726	-1.0669085	-0.0934364	0.07642048	0.18850514	0.36659342
Spata17	18.6577894	1.03950661	0.05589893	0.20222681	0.51859726	0.70157682
Spata2	882.532851	1.40008024	0.48550951	0.10098062	2.32E-07	3.34E-06
Spata20	52.6451593	1.12566369	0.17077586	0.25656473	0.12568827	0.27465615
Spata21	7.12506888	1.03581282	0.05076331	0.21975346	0.24855884	0.43954151
Spata24	21.7988014	1.00969062	0.0139133	0.19828328	0.86191204	0.92618801
Spata32	2.49384556	-1.043361	-0.0612385	0.2254393	0.09577888	0.2258703
Spata33	12.3110662	1.02874754	0.04088898	0.2123623	0.48252843	0.67201447
Spata5	2731.71045	-1.0447004	-0.0630892	0.05349696	0.22302492	0.41130856
Spata7	313.71886	1.0703838	0.09812819	0.11478554	0.31452442	0.51364837
Spats1	19.7888169	-1.0885717	-0.1224365	0.2367033	0.18135768	0.35724924
Spats2	1779.93787	1.03233462	0.04591067	0.0835018	0.55119235	0.72763936
Spc24	600.430683	1.22059445	0.28758394	0.11192255	0.00344614	0.01637426
Spc25	1338.90148	1.14895777	0.20032578	0.08826723	0.01291799	0.04827288
Spcs1	1978.50663	1.0212053	0.03027292	0.07044478	0.65146641	0.79782837
Spcs3	7209.47	-1.1608946	-0.215237	0.04631712	1.86E-06	2.28E-05
Spdef	2.09283165	-1.0005225	-0.0007536	0.21356015	0.97685575	0.98936766
Spdl1	703.903223	-1.0059229	-0.0085197	0.09806973	0.92108824	0.9592797
Spdya	7.55877438	2.86119607	1.51661836	1.76439807	0.00505122	0.02233554
Specc1	28.3010869	-3.099557	-1.632062	0.57773739	0.00018741	0.00141803
Specc1l	4910.39036	1.13848417	0.18711423	0.05222209	0.00021628	0.00160156
Spef1	2.7886894	1.0071996	0.01034961	0.21270861	0.77621179	0.875268
Spef1l	2.85146461	-1.0201328	-0.028757	0.21637078	0.31451095	0.51364837
Speg	179.89861	1.07829026	0.10874558	0.13988936	0.31605302	0.51502851
Spen	5144.61274	-1.1112603	-0.1521968	0.06636675	0.01502521	0.05458675
Spg11	829.268635	1.25589056	0.32871075	0.09765253	0.00022164	0.0016308
Spg20	1155.17764	1.13762485	0.18602489	0.0692311	0.00444702	0.02013654
Spg21	1388.64034	1.27354482	0.34884974	0.09073874	3.23E-05	0.00029537
Spg7	1471.67919	1.35629915	0.43967542	0.06367621	1.01E-12	3.23E-11
Sphk1	669.376828	1.10536854	0.14452745	0.11062699	0.13016476	0.28151453
Sphk2	222.990954	1.0879471	0.1216084	0.13661843	0.25960787	0.45194185
Spice1	561.128692	1.30885875	0.38830941	0.09010514	3.73E-06	4.24E-05
Spidr	730.420438	1.13313049	0.18031401	0.08627374	0.02242864	0.07521228
Spin1	5510.6102	1.19457287	0.25649486	0.04966695	1.09E-07	1.68E-06
Spindoc	733.59136	1.00610671	0.00878332	0.08884332	0.9160461	0.95628315
Spink8	2.88123087	1.00825266	0.01185721	0.21356065	0.70697001	0.8342194

Spire1	1937.33759	1.07834909	0.10882429	0.06191891	0.06595951	0.17201573
Spn	7.11205538	-1.0057194	-0.0082278	0.2088772	0.87216008	0.93093268
Spns1	1354.02176	1.05905667	0.08277979	0.10672068	0.37219426	0.57388162
Spop	2927.83914	-1.2085463	-0.2732728	0.05566367	3.71E-07	5.16E-06
Spopl	1936.1376	1.04746186	0.06689771	0.06074086	0.25038032	0.44166509
Spout1	880.775471	-1.0955417	-0.1316444	0.07944389	0.07311492	0.18501531
Spp1	28.8468271	-3.2662588	-1.7076391	0.55344379	8.10E-05	0.00067468
Sppl2a	4312.45751	1.15830805	0.21201899	0.065236	0.00063186	0.003958
Sppl2b	1058.44844	1.00894757	0.01285121	0.08506877	0.87016422	0.92968488
Sppl3	1662.05357	1.04067782	0.0575235	0.06364429	0.34391756	0.54329558
Spred1	3381.7671	-1.0690852	-0.0963769	0.05566895	0.07250207	0.18387324
Spred2	2574.98189	-1.258964	-0.332237	0.05762237	2.48E-09	4.98E-08
Spred3	43.1538595	-1.0941002	-0.1297449	0.22132157	0.22586793	0.41443281
Sprr1a	101.613955	-13.002433	-3.7007097	0.28880674	7.03E-39	1.08E-36
Sprr3	2.11114923	1.00915827	0.01315245	0.21366935	0.67866761	0.81501191
Sprtn	315.937074	-1.0248072	-0.0353525	0.12421792	0.7273599	0.84650499
Spry1	20.6477399	1.03377158	0.04791745	0.2097197	0.49013962	0.67753194
Spry2	410.052712	-1.0367249	-0.0520331	0.11914604	0.59925265	0.76207947
Spry4	13.8913469	-1.0383463	-0.0542877	0.21530505	0.37250763	0.57414546
Spryd3	759.62254	1.29907043	0.37747965	0.0821158	1.04E-06	1.33E-05
Spryd4	829.865549	-1.0656214	-0.0916949	0.08778064	0.25042319	0.44167989
Spryd7	545.320732	-1.0189613	-0.0270993	0.09753545	0.75214975	0.86265899
Spsb1	548.003018	-1.3878062	-0.4728061	0.13227128	5.31E-05	0.00046217
Spsb2	85.0425437	-1.0266425	-0.0379339	0.17820804	0.70951409	0.83605162
Spsb3	558.152603	1.04018567	0.05684107	0.09450548	0.50319483	0.68793231
Spsb4	262.994755	-1.0646104	-0.0903256	0.14125537	0.40487788	0.60427423
Spta1	137.959198	-2.4020519	-1.2642673	0.21215304	1.52E-10	3.71E-09
Sptan1	11313.1278	1.02474613	0.03526654	0.05246358	0.49026026	0.67755248
Sptbn1	29100.7572	1.27320941	0.34846972	0.04802434	9.95E-14	3.55E-12
Sptbn4	14.2987305	1.0752618	0.10468796	0.23919854	0.13732395	0.29250411
Sptssa	687.58836	-1.2173519	-0.2837462	0.10894423	0.0031401	0.01515627
Sptssb	3.27456093	-1.0240088	-0.0342281	0.2170778	0.28441351	0.4809051
Spty2d1	1219.92046	-1.0003823	-0.0005515	0.07734007	0.99316591	0.9968955
Sqle	14769.2388	1.02027116	0.02895263	0.05841445	0.61614905	0.77428768
Sqor	225.573472	1.03762647	0.05328719	0.13672803	0.6165149	0.77442204
Sqstm1	816.51867	-1.4986554	-0.5836686	0.09940653	5.19E-10	1.14E-08
Sra1	716.977616	-1.0387749	-0.0548831	0.08175405	0.4659322	0.65900315
Srbd1	1664.26655	1.16689361	0.22267303	0.07789047	0.00217371	0.01117834
Src	3855.36922	-1.1059212	-0.1452486	0.05097098	0.00327712	0.01571692
Srcin1	4.16881541	-1.0555829	-0.0780399	0.23176277	0.08922652	0.21492886
Srd5a1	899.701882	-1.0175612	-0.0251156	0.08433827	0.74531753	0.85822982
Srd5a3	237.068488	-1.0583971	-0.081881	0.12878489	0.42972222	0.62760707
Srebf2	5778.57373	1.10225342	0.14045595	0.05904858	0.01292421	0.04828204
Srek1ip1	353.903961	-1.0278161	-0.0395821	0.11391046	0.68188301	0.81683798
Srf	1613.41063	-1.0995123	-0.1368638	0.06087319	0.01867534	0.06479715
Srfbp1	587.056828	-1.2239848	-0.2915856	0.11942197	0.00471922	0.0211301
Srgap1	863.78848	1.03477098	0.0493115	0.08532806	0.52607204	0.70743049
Srgap2	1965.08794	1.13481275	0.18245426	0.057622	0.00099026	0.00578181
Srgap3	2232.27645	1.38473488	0.46960979	0.0782237	3.33E-10	7.61E-09
Srgn	60.8720978	1.19180731	0.25315101	0.26280256	0.07080376	0.18056861

Sri	661.446416	1.09757256	0.13431632	0.10004764	0.12930987	0.27999653
Srl	3.00712632	1.01082405	0.0155319	0.21348243	0.6526814	0.7986117
Srm	5449.21326	1.05961133	0.08353517	0.06889923	0.19820834	0.37907567
Srp14	634.463757	1.03259088	0.04626876	0.0868327	0.55989632	0.73310886
Srp19	892.889754	-1.0857451	-0.1186855	0.08832704	0.13978183	0.29625845
Srp68	5600.38304	1.04628703	0.06527868	0.04424711	0.13067006	0.28227378
Srp72	5009.46459	-1.0076993	-0.0110652	0.05378414	0.83125393	0.90933927
Srp9	713.179861	-1.0335737	-0.0476412	0.09266424	0.56837956	0.73857312
Srpk1	7743.31777	-1.2307533	-0.2995416	0.04590539	2.45E-11	6.66E-10
Srpk2	1645.52367	1.10907357	0.14935507	0.06188019	0.01150386	0.04405516
Srpr	4806.8562	-1.0335887	-0.0476622	0.04610581	0.28924787	0.48574196
Srprb	1414.55823	-1.150503	-0.2022647	0.08173028	0.00729161	0.03007404
Srpx2	390.783417	1.61758593	0.69384236	0.1348126	2.51E-08	4.32E-07
Srr	75.5009274	1.04247551	0.06001349	0.16221449	0.58447535	0.74916242
Srrd	227.145352	1.04174095	0.05899657	0.12428984	0.56361867	0.73559175
Srrm1	11126.0105	-1.0168525	-0.0241105	0.05019679	0.62055541	0.777138
Srrm2	35128.4742	1.00159644	0.00230134	0.05707415	0.96906911	0.98529846
Srrm4	3.59994227	1.01644939	0.02353838	0.21424454	0.51192154	0.69592646
Srrt	5723.96438	-1.2218527	-0.2890704	0.05747956	1.97E-07	2.89E-06
Srsf1	3361.41586	-1.1267208	-0.1721301	0.06793894	0.00721926	0.02982223
Srsf10	2141.70881	-1.1003085	-0.137908	0.07664943	0.05336378	0.14608814
Srsf11	3101.62109	-1.1045895	-0.1435104	0.07996887	0.05225348	0.14379529
Srsf2	398.987845	-1.1149806	-0.1570186	0.14877277	0.16623222	0.33534405
Srsf4	2085.00005	-1.0455636	-0.0642809	0.06189193	0.27725009	0.47278669
Srsf5	3838.97856	-1.1473775	-0.1983401	0.05999812	0.00055854	0.00358283
Srsf6	9093.93335	1.07225345	0.10064596	0.06217051	0.09409332	0.2232516
Srsf7	5298.55058	-1.2376475	-0.3076005	0.05772044	3.37E-08	5.65E-07
Srsf9	498.863515	1.0402135	0.05687967	0.10395751	0.53414564	0.71327067
Srxn1	4278.96365	-1.0898712	-0.1241576	0.05548479	0.02021491	0.06922027
Ss18	924.095105	-1.0824595	-0.114313	0.07882487	0.11730568	0.26158572
Ss18l1	131.070944	1.94589889	0.96043675	0.18788786	2.15E-08	3.74E-07
Ss18l2	387.979004	1.10657075	0.14609569	0.13195217	0.17068123	0.34216377
Ssb	11378.6626	-1.1924417	-0.2539187	0.0575815	4.66E-06	5.19E-05
Ssbp2	178.749381	-1.0245913	-0.0350486	0.13807584	0.74102287	0.85589602
Ssbp3	2002.0992	-1.1623702	-0.2170696	0.05688613	7.35E-05	0.00061987
Ssbp4	144.484412	1.22550503	0.2933764	0.24162486	0.04516475	0.12911413
Ssc5d	240.780623	1.61724695	0.69353999	0.17890425	9.61E-06	0.00010031
Ssh1	856.849535	1.06387848	0.08933336	0.07728366	0.21510106	0.40120043
Ssh2	3564.53215	-1.0922775	-0.1273394	0.05504167	0.01638565	0.05833801
Ssh3	198.108213	2.18475236	1.12746976	0.15417329	1.83E-14	6.90E-13
Ssna1	223.271518	-1.0166941	-0.0238857	0.14631479	0.82311026	0.90419748
Sspn	202.686168	-1.4075191	-0.4931545	0.22097346	0.00286064	0.01404016
Ssr1	16742.9873	1.01737734	0.02485486	0.04277943	0.47300097	0.66421861
Ssr2	4683.88824	1.06513571	0.09103726	0.05893876	0.10778354	0.2458962
Ssr3	1081.32382	-1.0442235	-0.0624305	0.08687059	0.43151312	0.62957666
Ssr4	1923.88456	-1.1126516	-0.1540019	0.06379777	0.01108422	0.04269057
Ssrp1	13926.7101	1.00435091	0.00626342	0.04436448	0.88662972	0.93909612
Ssu72	507.736047	-1.2800669	-0.3562192	0.10008678	9.48E-05	0.00077225
Ssx2ip	2150.13941	-1.0533128	-0.074934	0.06930542	0.25180231	0.44319698
Ssxa1	15.260538	-1.0269012	-0.0382973	0.21330332	0.4675566	0.660123

St13	7163.74376	-1.0497993	-0.0701135	0.05548408	0.19208148	0.37084609
St14	167.597898	-3.5248006	-1.8175416	0.18460671	4.49E-24	3.36E-22
St3gal1	7762.08694	-1.2298867	-0.2985255	0.058809	1.37E-07	2.08E-06
St3gal2	283.849909	1.05281278	0.07424891	0.12294293	0.46468778	0.65789683
St3gal3	346.925749	-1.0170275	-0.0243587	0.13265782	0.81478458	0.89951701
St3gal4	801.857424	1.02299543	0.0327997	0.07676108	0.64802263	0.79553628
St3gal5	651.844063	-2.4055687	-1.266378	0.10609453	4.87E-34	5.94E-32
St3gal6	475.322209	-1.1622609	-0.216934	0.1153653	0.02844553	0.08966222
St6galnac4	424.738005	1.22746635	0.29568348	0.12993167	0.00707566	0.02934827
St6galnac6	483.917529	1.03728896	0.05281785	0.10834409	0.57263266	0.74125145
St7	794.339643	-1.0132636	-0.0190096	0.0802605	0.79789617	0.88793762
St7l	152.458282	-1.0321931	-0.0457128	0.14505621	0.67050796	0.80997438
St8sia4	452.013337	-1.0496147	-0.0698598	0.10542369	0.44684634	0.64302826
St8sia6	5.64331198	1.00026127	0.00037688	0.21239503	0.99406866	0.99742843
Stac3	142.552233	1.02635905	0.03753552	0.14907048	0.72962769	0.84829649
Stag1	5736.19337	-1.1533035	-0.2057722	0.04776729	8.04E-06	8.49E-05
Stag2	8444.32098	-1.0071883	-0.0103334	0.05570141	0.84708249	0.91772046
Stam	2738.31033	-1.1030284	-0.1414699	0.06379927	0.01959696	0.06746496
Stam2	1097.09938	1.0273719	0.03895851	0.07598677	0.58398887	0.74876363
Stambp	696.665419	-2.070566	-1.0500252	0.09742271	3.29E-28	3.17E-26
Stap2	100.357214	-1.1086608	-0.148818	0.16680168	0.2059681	0.39030346
Star	54.7574543	-1.6120836	-0.6889265	0.37144047	0.00357119	0.01686836
Stard10	4.6358267	1.00563333	0.00810437	0.21180011	0.83990761	0.9142043
Stard13	1988.3174	1.01742414	0.02492123	0.05951967	0.65788374	0.8014732
Stard3	846.428495	-1.0722071	-0.1005836	0.09506244	0.23670192	0.42617157
Stard3nl	631.147507	1.08109649	0.1124953	0.08735014	0.15782176	0.32331629
Stard4	183.584177	-1.0965132	-0.1329231	0.14750856	0.23527248	0.42514372
Stard7	3279.65743	-1.0569775	-0.0799446	0.0578931	0.15075177	0.31253119
Stat1	1511.62515	1.22200032	0.28924466	0.0842881	0.00021634	0.00160156
Stat2	345.398418	-1.0254448	-0.0362499	0.12266395	0.71858893	0.84156113
Stat3	1618.99814	1.09889235	0.13605006	0.07641142	0.05613983	0.151875
Stat5a	1279.56791	1.26770193	0.34221558	0.07333168	8.64E-07	1.13E-05
Stat6	2814.73283	-1.1354066	-0.1832091	0.06417315	0.00268453	0.01337565
Stau1	3108.30413	1.32810638	0.40937071	0.05637695	8.84E-14	3.17E-12
Stau2	3.60230535	-1.0689897	-0.0962479	0.24608008	0.01255672	0.04715672
Stbd1	240.889112	-1.1390803	-0.1878695	0.14712662	0.10033653	0.23362719
Stc2	1435.48284	1.28983759	0.36718942	0.08143708	1.64E-06	2.03E-05
Steap1	755.582054	1.13309354	0.18026696	0.09603223	0.03617423	0.10867538
Steap2	1555.35239	1.13062092	0.1771153	0.07670782	0.01318495	0.04901312
Stil	1632.55802	1.0256515	0.03654061	0.06047562	0.52913122	0.70983045
Stim1	1129.06238	1.02667591	0.03798083	0.07050667	0.56896952	0.73897389
Stim2	3548.54746	1.12738201	0.17297645	0.05628751	0.00141059	0.00773863
Stimate	362.746122	1.11381825	0.15551383	0.10606082	0.09325684	0.22167752
Stip1	10948.2921	1.05308658	0.07462405	0.05807172	0.18124441	0.3571909
Stk10	2450.52936	1.06587914	0.09204387	0.06045087	0.1120688	0.25323714
Stk11	2698.26058	-1.0490791	-0.0691235	0.05865729	0.22852317	0.41742118
Stk11ip	590.300771	1.25767081	0.33075435	0.10211154	0.00033428	0.00228744
Stk16	730.815088	1.01960089	0.02800453	0.08041653	0.70446362	0.83202831
Stk17b	2314.90597	-1.057567	-0.0807491	0.05480269	0.12682101	0.27618242
Stk19	159.448009	-1.000642	-0.000926	0.15867975	0.99426061	0.99742843

Stk24	3597.88304	1.09532774	0.13136261	0.04770767	0.00474237	0.02121149
Stk25	3420.30244	1.05387974	0.07571024	0.06191949	0.20123071	0.38365314
Stk3	1565.61559	1.01124981	0.01613944	0.06648401	0.8021212	0.89060202
Stk35	1337.76743	-1.0845399	-0.1170831	0.06747434	0.06671153	0.17342851
Stk36	247.84222	1.57805955	0.65815165	0.20675783	0.00012365	0.00097898
Stk38	1211.21703	1.05144191	0.07236915	0.077932	0.31865873	0.51720318
Stk38l	557.482758	-1.0531604	-0.0747251	0.08730746	0.34378469	0.54321967
Stk39	3567.09517	-1.1421696	-0.1917769	0.06051775	0.00092541	0.00547297
Stk4	7109.96408	1.3881749	0.47318935	0.04413031	1.44E-27	1.30E-25
Stmn1	3303.21766	1.03005	0.04271436	0.08341308	0.57725724	0.74410617
Stn1	678.304103	1.03520958	0.04992288	0.08584657	0.5254179	0.70689766
Stoml1	122.857477	1.22825057	0.29660491	0.2365335	0.0427349	0.1238379
Stoml2	564.41537	-1.108557	-0.148683	0.10456793	0.10412124	0.24040971
Ston1	1740.40477	-1.0043845	-0.0063117	0.05897625	0.90956921	0.95292637
Ston2	15.1638914	-1.048388	-0.0681727	0.22022625	0.28514164	0.48187146
Stpg2	22.1961864	-1.0852306	-0.1180016	0.23132039	0.20592167	0.39027321
Strada	299.415205	1.15270666	0.20502542	0.13374731	0.06018373	0.16041481
Stradb	330.943477	1.22362769	0.29116466	0.15261099	0.01623888	0.0580413
Strap	6489.20233	-1.0716866	-0.0998831	0.04681381	0.02381999	0.0788053
Strbp	2842.79771	1.19919356	0.26206454	0.06699949	3.95E-05	0.00035339
Strip1	2323.45048	1.08434269	0.11682077	0.05976449	0.04146551	0.12088759
Strip2	3314.28065	-1.0991213	-0.1363506	0.06371525	0.02462056	0.08080692
Strn	2070.04256	1.14051987	0.18969159	0.06567316	0.00235055	0.01195229
Strn3	1842.58515	1.00581721	0.00836814	0.05784827	0.88151834	0.9366498
Strn4	1916.77449	1.32063051	0.40122689	0.07866596	7.47E-08	1.18E-06
Stt3a	4588.4889	1.01878279	0.02684649	0.0511136	0.59260465	0.75565056
Stt3b	4206.89822	1.01014724	0.01456559	0.06085668	0.80195612	0.89054671
Stub1	1281.07028	1.05096594	0.07171591	0.06321458	0.23474744	0.42455382
Stx11	100.360114	-1.7901877	-0.8401109	0.33544903	0.00066431	0.004135
Stx12	2486.44505	1.00283742	0.00408774	0.05980039	0.94349847	0.97249883
Stx16	3023.79684	1.37673923	0.46125533	0.05381676	1.89E-18	1.02E-16
Stx17	346.041498	1.10433093	0.14317257	0.11200491	0.13620078	0.29078866
Stx18	564.924872	1.03986704	0.05639907	0.10369716	0.53290804	0.71228631
Stx1a	194.032461	1.14206856	0.19164925	0.14407948	0.09090249	0.21781909
Stx2	339.592911	1.14660418	0.19736744	0.11573957	0.04591196	0.13055099
Stx3	108.796363	1.23781317	0.30779357	0.24874377	0.04091464	0.11974331
Stx4a	1353.26687	1.03746912	0.0530684	0.06631789	0.40009083	0.60013033
Stx5a	734.868865	-1.0059217	-0.008518	0.096039	0.92003852	0.95873208
Stx6	636.032107	-1.0605157	-0.084766	0.0906054	0.30152166	0.49928806
Stx7	1178.82547	1.30410223	0.38305697	0.07667447	1.38E-07	2.09E-06
Stx8	183.527998	1.12575896	0.17089796	0.16656227	0.1533678	0.31626554
Stxbp1	456.846192	1.27023766	0.34509845	0.11520753	0.00069908	0.00430955
Stxbp3	1127.99439	-1.0621803	-0.0870286	0.07059374	0.19102808	0.369648
Stxbp4	371.305622	1.03057669	0.04345187	0.10279879	0.63292708	0.78571636
Stxbp5	1325.97731	1.14684053	0.1976648	0.0731052	0.00396086	0.0183437
Stxbp6	1813.68376	1.12867605	0.17463146	0.06954439	0.00779233	0.03175149
Styk1	2.15458759	-1.0257158	-0.0366311	0.21902578	0.11411914	0.25633285
Styxl1	2.49634855	-1.0030512	-0.0043952	0.21341283	0.88017865	0.93575978
Suclg1	3380.15686	-1.8981409	-0.9245871	0.06455084	1.15E-47	2.84E-45
Suclg2	4631.20886	1.0597708	0.08375228	0.05089546	0.09143856	0.21857212

Sucnr1	34.7645002	1.10381129	0.14249354	0.23235625	0.18648589	0.36367887
Suco	1930.09304	1.01760297	0.02517479	0.05946714	0.65430556	0.79983482
Suds3	4616.0898	1.152922	0.20529491	0.0541614	8.80E-05	0.00072673
Sufu	443.217298	1.06070969	0.08502985	0.10769082	0.36335651	0.56398848
Sugct	10.6480619	-1.0318373	-0.0452156	0.20879725	0.51703205	0.70019881
Sugp1	1079.43192	-1.0817551	-0.1133739	0.08874867	0.15987697	0.32585903
Sugp2	442.717168	1.4851716	0.57062963	0.11042984	3.06E-08	5.18E-07
Sulf2	1908.02063	-1.1388832	-0.1876198	0.07293815	0.00610345	0.02592347
Sult2b1	6.7115306	1.00797172	0.01145516	0.2112473	0.79544651	0.88621237
Sumf1	944.795823	1.26476717	0.33887183	0.08426464	1.64E-05	0.0001613
Sumo3	235.469131	-1.0370513	-0.0524872	0.13519727	0.61880005	0.77607487
Sun1	2614.97187	1.13008985	0.17643748	0.0649578	0.00428368	0.01953506
Sun2	1833.18257	1.31332334	0.39322215	0.07723837	8.00E-08	1.26E-06
Suox	588.300531	1.1253615	0.17038851	0.09562393	0.04672371	0.13221354
Supt16	16938.7205	-1.110629	-0.151377	0.06734101	0.01745458	0.06164685
Supt20	1499.63302	1.03793228	0.05371232	0.0797112	0.46818663	0.66059458
Supt3	628.261382	1.05944569	0.08330963	0.08121291	0.26712419	0.46135377
Supt4a	869.74288	-1.0827105	-0.1146475	0.10685756	0.21688117	0.40311198
Supt5	5839.77601	1.08249923	0.11436599	0.05014743	0.018546	0.06457576
Supt7l	649.511188	-1.0220629	-0.031484	0.08944412	0.69749203	0.82741461
Supv3l1	1543.28808	1.01306707	0.0187297	0.07676419	0.79461631	0.88582673
Surf1	89.0502856	1.12925355	0.17536944	0.19225749	0.15772781	0.32317551
Surf2	1441.77003	-1.0363452	-0.0515046	0.07661515	0.47095691	0.66274394
Surf4	961.870955	1.04422678	0.06243506	0.09600472	0.46699896	0.6597848
Susd1	10.8276453	-1.0222273	-0.031716	0.21124708	0.55679335	0.73124081
Susd4	6.7433976	-1.0935172	-0.1289759	0.27115513	0.01191804	0.04534584
Susd6	1462.9647	1.01821094	0.02603648	0.08415777	0.7373142	0.85336058
Suv39h1	1328.38577	-1.0752899	-0.1047257	0.08452762	0.17637616	0.35014864
Suv39h2	394.122984	-1.0267036	-0.0380198	0.10690298	0.68112324	0.81646968
Suz12	4538.23262	-1.0883785	-0.1221804	0.04928228	0.01067052	0.0414587
Sv2b	16.473176	-1.1426016	-0.1923225	0.31329872	0.05334882	0.14608814
Svbp	282.862011	-1.0527388	-0.0741476	0.1294746	0.47539617	0.66628281
Svil	929.339069	-1.0940956	-0.1297388	0.09643897	0.13150432	0.28351855
Swap70	2308.14513	-1.0164006	-0.0234691	0.0606966	0.6856316	0.81956079
Swsap1	304.836369	1.06640422	0.09275439	0.1113	0.33187581	0.53172095
Swt1	540.58627	1.039623	0.05606046	0.09882754	0.52370101	0.7055011
Syap1	1382.04079	-1.0069598	-0.0100061	0.07259423	0.88298622	0.9371989
Syde1	981.219302	1.07441114	0.10354616	0.07628128	0.14560411	0.30521725
Syde2	367.808389	1.06825126	0.09525102	0.12236359	0.34768552	0.54735793
Syf2	1744.6299	-1.0419317	-0.0592607	0.06724065	0.35291238	0.55265464
Sympk	1692.71419	1.11968051	0.16308713	0.07814479	0.02461768	0.08080692
Syn2	238.42923	-1.0045698	-0.0065778	0.13561582	0.94898973	0.97462988
Syn3	5.76587299	1.04190139	0.05921874	0.22315325	0.17714105	0.35130443
Synb	5.74654237	-1.0351951	-0.0499027	0.21958765	0.24107089	0.43075486
Syncrip	12335.3379	-1.0582037	-0.0816173	0.06074952	0.16020607	0.32637401
Syne1	5.41944899	-1.031689	-0.0450081	0.21791586	0.28725361	0.4836759
Syne2	12351.1413	1.19384252	0.25561254	0.068927	9.06E-05	0.0007428
Syne3	1319.28336	-1.2229476	-0.2903626	0.08764617	0.0003231	0.00222167
Syne4	15.8492075	-1.119489	-0.1628404	0.27847137	0.08124798	0.20022828
Syngap1	967.970415	1.11395649	0.15569288	0.09574093	0.06939465	0.17839563

Syngr1	225.670796	-1.1700883	-0.2266174	0.15305399	0.05532489	0.15011934
Syngr2	595.7654	-1.1048482	-0.1438482	0.10173436	0.10761149	0.24559115
Syngr3	17.0532466	-1.0072711	-0.0104521	0.2014736	0.88313045	0.93727433
Syngr4	7.48119646	1.00784953	0.01128026	0.20836588	0.8360434	0.91195011
Synj1	2422.80696	1.14435772	0.1945381	0.06989653	0.00318635	0.01535705
Synj2	992.97583	1.10334767	0.14188746	0.1023046	0.11500118	0.25790706
Synj2bp	136.26616	1.04152349	0.05869537	0.14360057	0.58862345	0.75207125
Synm	107.791106	-1.5411838	-0.624039	0.25026791	0.00099172	0.00578506
Synpo	339.172196	1.00209937	0.00302558	0.10996784	0.97540899	0.98867578
Synpo2	57.4206765	-1.1135761	-0.1552001	0.24396772	0.15188734	0.31402144
Synpo2l	1.77425721	1.0295102	0.04195812	0.22102888	0.02954754	0.09256266
Synrg	892.29907	1.13511752	0.18284167	0.08633003	0.02057839	0.0701835
Sypl	1095.27826	-1.0736786	-0.1025621	0.08149026	0.17209609	0.34413845
Sys1	189.929619	1.40585157	0.49144428	0.19451808	0.00139824	0.0076873
Syt11	15.9118379	1.03547293	0.05028984	0.20822868	0.49730674	0.68369178
Sytl2	6.26806087	-1.0817103	-0.1133142	0.25760867	0.01515612	0.05499998
Sytl4	532.928987	1.15286002	0.20521735	0.11046437	0.03241129	0.09978096
Syvn1	425.113845	1.01459439	0.02090309	0.13205152	0.84229878	0.91540234
Szrd1	671.788414	-1.048472	-0.0682884	0.08815048	0.39357354	0.59460751
Szt2	1510.15286	1.0160595	0.02298488	0.08886722	0.77668855	0.87544229
TARBP2	538.816035	1.05108472	0.07187896	0.12898243	0.49022106	0.67755248
TAZ	77.5262058	1.098041	0.13493192	0.19521815	0.24859829	0.4395506
TCP1	18130.3814	-1.1049328	-0.1439586	0.04946763	0.00270047	0.01344461
TK1	569.404647	-1.0456874	-0.0644516	0.08734978	0.41848742	0.61732196
TLR4	1810.06846	-1.1308147	-0.1773626	0.08820553	0.02720033	0.08680525
TRMT5	900.441222	1.00406872	0.00585802	0.08692082	0.94037923	0.97118925
TSFM	834.929686	-1.1428734	-0.1926656	0.07819912	0.00800075	0.03248481
TSN	3761.07697	-1.1203984	-0.1640119	0.0566729	0.00259571	0.01300899
TUBA1C	22359.9054	-1.1050889	-0.1441625	0.06734465	0.0234874	0.07792632
TUBB5	51459.173	1.08124059	0.11268757	0.05411058	0.03110776	0.09653352
Tab2	4552.98699	1.05421252	0.07616574	0.06377117	0.21049362	0.39583431
Tab3	1639.31075	-1.0172327	-0.0246497	0.0626379	0.67996215	0.81587815
Tac4	2.37409613	-1.0103908	-0.0149133	0.21429038	0.59580436	0.75879524
Tacc1	1726.17697	-1.1464874	-0.1972205	0.06412196	0.00123534	0.00691638
Tacc3	2822.78426	-1.0783965	-0.1088877	0.06346604	0.07173656	0.18236659
Taco1	327.359491	-1.0051059	-0.0073475	0.12002858	0.94030248	0.97118925
Tada1	613.762535	-1.007385	-0.0106152	0.086931	0.89257937	0.94347237
Tada2a	441.394683	-1.4203513	-0.5062479	0.11852094	2.81E-06	3.30E-05
Tada2b	326.543582	-1.0662765	-0.0925817	0.1104974	0.32906401	0.52889711
Taf1	3102.17144	1.01400996	0.02007183	0.05506353	0.70671003	0.83415689
Taf10	633.832289	-1.0369664	-0.0523691	0.09981801	0.55289881	0.72867565
Taf11	587.533049	1.32029207	0.40085712	0.11065978	6.05E-05	0.00052074
Taf12	688.811217	-1.232451	-0.3015303	0.09797013	0.00066914	0.00415898
Taf13	785.824439	-1.1334189	-0.1806811	0.09736549	0.03781155	0.11264325
Taf15	3245.89073	-1.2136018	-0.2792952	0.0638086	4.74E-06	5.28E-05
Taf1a	481.555157	1.01918395	0.02741447	0.09736184	0.75366682	0.86298066
Taf1c	335.065739	-1.0586079	-0.0821684	0.11715566	0.4042796	0.60366653
Taf1d	1576.48895	-1.0639935	-0.0894894	0.1457686	0.41253319	0.6114962
Taf2	1248.32441	-1.2534302	-0.3258816	0.09405634	0.00015298	0.00118266
Taf3	2736.08946	-1.0390509	-0.0552663	0.0599089	0.33722717	0.53697237

Taf4	1389.29772	1.30827658	0.38766757	0.07203145	1.73E-08	3.04E-07
Taf4b	1757.50758	-1.3254677	-0.4065015	0.06633119	1.91E-10	4.54E-09
Taf5	696.035767	-1.0604848	-0.0847239	0.08772344	0.288395	0.48491905
Taf6	1775.40006	1.04219181	0.05962082	0.06451561	0.33275795	0.53262894
Taf6l	589.499406	-1.0132486	-0.0189882	0.09161655	0.81835223	0.90192653
Taf7	418.273079	1.11952782	0.16289038	0.11398642	0.09502098	0.22476316
Taf7l2	2.10113391	-1.0083887	-0.0120518	0.21408916	0.66350949	0.80543405
Taf8	1020.84104	-1.0518119	-0.0728767	0.08402185	0.344722	0.5440967
Taf9b	177.32079	1.10489956	0.14391523	0.16903836	0.22444166	0.41301653
Tagln	172.899932	1.64428065	0.71745656	0.21561432	7.14E-05	0.00060338
Tal2	10.8483254	1.03577808	0.05071493	0.21938425	0.26177313	0.45444015
Tamm41	451.58765	-1.1162174	-0.1586181	0.1319912	0.13699746	0.2920029
Tanc1	1964.4476	-1.1480384	-0.1991709	0.07453131	0.00428576	0.01953757
Tanc2	1554.01977	1.24027055	0.31065486	0.07044861	3.42E-06	3.92E-05
Tango2	274.474818	1.07126584	0.09931653	0.14612437	0.36859518	0.57004761
Tango6	1741.90339	1.01831646	0.02618597	0.06382821	0.66789276	0.80811765
Taok1	2732.37158	-1.1292129	-0.1753176	0.05993955	0.00225341	0.01152804
Taok2	2304.67037	1.11511733	0.15719552	0.06495809	0.01088178	0.04213894
Taok3	2409.0145	1.05929268	0.08310126	0.07484903	0.23584074	0.42550985
Tap1	729.64383	-1.3547491	-0.4380257	0.10162303	2.90E-06	3.39E-05
Tapbp	1311.71994	1.17123152	0.22802629	0.10172716	0.0116137	0.0444492
Tapt1	854.83495	-1.1755432	-0.2333276	0.07720443	0.00120006	0.00675409
Tarbp1	626.612199	-1.0666944	-0.0931469	0.11758459	0.34530862	0.54468703
Tardbp	6407.69688	-1.2002606	-0.2633477	0.07272592	0.00012218	0.00097036
Tars	6928.30496	1.11023496	0.15086503	0.06360683	0.01285195	0.04811031
Tars2	837.755144	1.21941397	0.28618798	0.11801291	0.00510708	0.02251262
Tarsl2	1351.66553	1.06331769	0.0885727	0.07188375	0.19087913	0.36962885
Tas1r1	61.2647994	1.32303217	0.40384814	0.31034677	0.02042046	0.06981213
Tasor	913.204611	-1.1952344	-0.2572935	0.08657419	0.00123375	0.00691048
Tasor2	6951.01356	-1.0890234	-0.123035	0.04710928	0.00876511	0.03510871
Tasp1	567.27437	-1.0788627	-0.1095112	0.09278351	0.18999663	0.36865448
Tatdn1	874.327039	-1.0456573	-0.0644101	0.08876975	0.42818192	0.62608502
Tatdn3	105.618147	1.42277148	0.50870396	0.24522212	0.00383135	0.01785361
Tax1bp1	4511.30727	-1.7070187	-0.7714789	0.05975492	3.64E-39	5.69E-37
Tax1bp3	162.89435	1.00228688	0.0032955	0.13690408	0.97626705	0.98895979
Tbc1d1	3244.08546	1.09778343	0.13459347	0.04961637	0.00521055	0.02285078
Tbc1d10a	304.567793	1.01696432	0.02426907	0.1193216	0.81186368	0.89735937
Tbc1d10b	1545.35475	1.18945019	0.25029486	0.0674302	9.36E-05	0.00076451
Tbc1d10c	30.6271942	-1.0655539	-0.0916035	0.21515736	0.31331641	0.51209664
Tbc1d12	867.289762	1.5943594	0.67297688	0.08981034	6.70E-15	2.66E-13
Tbc1d13	2712.24663	1.1579823	0.2116132	0.06600448	0.00074348	0.00454822
Tbc1d14	1728.18591	1.05629388	0.07901127	0.06452485	0.19891015	0.38002074
Tbc1d15	3211.49133	-1.0535921	-0.0753164	0.05353699	0.14590558	0.30550023
Tbc1d16	280.358713	1.37104324	0.45527407	0.15212626	0.0004249	0.0028214
Tbc1d17	512.8142	1.17650895	0.23451229	0.10770766	0.01300917	0.04852868
Tbc1d19	822.504293	-1.0231006	-0.0329481	0.08446479	0.67287574	0.81086907
Tbc1d2	573.992589	1.04465962	0.06303295	0.08485158	0.41860151	0.61733754
Tbc1d20	4481.42839	1.46134114	0.547293	0.05941827	4.51E-21	2.84E-19
Tbc1d22a	1078.81116	1.14866753	0.19996128	0.07006764	0.00247775	0.01251084
Tbc1d22b	811.07846	1.06570891	0.09181343	0.09326994	0.27457624	0.46993205

Tbc1d23	1772.61555	1.09433988	0.13006089	0.07878275	0.07532869	0.18928143
Tbc1d24	296.57039	1.16712412	0.22295799	0.13741023	0.04482629	0.12834707
Tbc1d25	374.002872	-1.0190962	-0.0272902	0.11618498	0.77912059	0.87673482
Tbc1d2b	5257.19065	1.21965107	0.28646847	0.04429457	3.88E-11	1.03E-09
Tbc1d32	725.76984	1.22268145	0.29004858	0.09130782	0.00052581	0.00340014
Tbc1d4	627.101926	1.00602598	0.00866757	0.0995399	0.90345744	0.94988097
Tbc1d5	2119.98609	1.16524694	0.22063572	0.06187385	0.00019199	0.00144417
Tbc1d7	390.052518	1.0108026	0.01550128	0.10099132	0.8626447	0.92665838
Tbc1d8	70.9503467	-1.2218604	-0.2890794	0.22947141	0.04462832	0.12783738
Tbc1d8b	2586.34924	1.16954087	0.22594228	0.07321989	0.00103316	0.00599694
Tbc1d9b	2341.8473	1.11719979	0.15988721	0.05992782	0.00533969	0.02331335
Tbca	3012.43565	-1.1241606	-0.1688482	0.06062512	0.00357592	0.01688443
Tbcb	409.387333	1.08691987	0.12024559	0.11573391	0.22033509	0.40758014
Tbcc	137.797694	1.19152751	0.25281226	0.18709627	0.05458083	0.14857213
Tbccd1	1123.55731	-1.041765	-0.0590299	0.08394479	0.44350245	0.64035915
Tbcd	2484.30338	1.07392556	0.10289399	0.06347021	0.08885412	0.2143543
Tbce	616.960715	1.00573641	0.00825224	0.09960031	0.92175652	0.95974161
Tbcel	1875.65222	1.18376872	0.24338724	0.07243495	0.00036032	0.0024409
Tbck	2901.35354	1.25601497	0.32885366	0.05872718	6.80E-09	1.27E-07
Tbk1	1145.54667	1.1475958	0.19861459	0.07418862	0.00428298	0.01953506
Tbl1x	7852.55375	-1.0890134	-0.1230217	0.04246518	0.00305872	0.01482489
Tbl1xr1	2734.69482	-1.0214778	-0.0306579	0.06278938	0.60900393	0.76921124
Tbl2	3266.30444	-1.1699626	-0.2264624	0.06097812	0.00010322	0.00083683
Tbl3	1408.55006	-1.0734794	-0.1022946	0.08849338	0.20277158	0.38578701
Tbp	1082.28054	-1.0694025	-0.0968049	0.06670278	0.12590505	0.27486809
Tbpl1	408.5925	-1.1889947	-0.2497423	0.13566026	0.02460138	0.08078571
Tbr1	9.93051501	1.0048159	0.0069312	0.20406923	0.91808062	0.95747072
Tbrg1	876.6254	-1.1141829	-0.1559861	0.07433423	0.02485502	0.0813264
Tbrg4	1708.87939	1.09898898	0.13617691	0.07978632	0.06522737	0.17066229
Tbx15	605.377573	-1.0379545	-0.0537432	0.08969969	0.50892401	0.69327275
Tbx18	170.55946	-2.0260672	-1.0186821	0.21136416	8.52E-08	1.33E-06
Tbx2	495.978289	-1.3196377	-0.4001419	0.09830452	9.74E-06	0.00010166
Tbx6	2.80713941	-1.0198891	-0.0284122	0.21587815	0.3710549	0.57246938
Tbxas1	5.52478062	1.00028724	0.00041434	0.21138666	0.99422718	0.99742843
Tcaf1	792.422975	1.04069393	0.05754584	0.09060208	0.48320162	0.67255597
Tcaf2	2.66289828	-1.036798	-0.0521349	0.22289501	0.08520356	0.20758036
Tcaim	1092.13262	1.01483566	0.02124612	0.07488529	0.76274504	0.86877882
Tcam1	13.905771	1.13115006	0.17779033	0.33243121	0.0060901	0.02588394
Tcea2	59.9432594	2.49998716	1.32192069	0.3724018	1.99E-05	0.00019011
Tceal1	11.4396503	-1.0009028	-0.0013019	0.20488875	0.98106448	0.99096641
Tceal5	4.42361499	-1.0263292	-0.0374935	0.21730331	0.28870471	0.48527848
Tceal8	741.398817	-1.0725193	-0.1010036	0.11935024	0.33062982	0.53068137
Tceal9	526.015613	1.11441384	0.15628508	0.14343805	0.16191826	0.32881625
Tceanc	21.5506562	1.0519661	0.07308821	0.2104527	0.39175391	0.59313526
Tceanc2	555.40704	-1.1398345	-0.1888244	0.08861959	0.01926165	0.06643559
Tcerg1	3789.67391	-1.0448961	-0.0633594	0.06942726	0.33416471	0.53407985
Tcf12	4408.15867	1.01960624	0.0280121	0.06044951	0.62821613	0.7826155
Tcf19	1520.73436	1.14599872	0.19660544	0.08889679	0.01529061	0.05538107
Tcf25	2712.21894	1.03850225	0.05450434	0.06305004	0.36497274	0.56594853
Tcf3	2149.25902	1.07198899	0.10029009	0.07187085	0.1378525	0.2933373

Tcf4	455.395701	-1.4569727	-0.5429738	0.10269496	1.61E-08	2.85E-07
Tcf7	662.311198	1.17083471	0.22753742	0.09264587	0.00680273	0.02835761
Tcf7l1	708.092268	1.41037583	0.49607966	0.08845063	3.13E-09	6.22E-08
Tcf7l2	375.116572	-1.1007969	-0.1385483	0.12445792	0.17727363	0.3513655
Tchp	1206.82701	-1.002243	-0.0032323	0.09003836	0.96143767	0.98159055
Tcirg1	359.594416	-1.054983	-0.0772198	0.14048008	0.47398087	0.66475866
Tcn2	3.49835267	-1.0720741	-0.1004046	0.24961025	0.00679019	0.02831457
Tcof1	3841.20498	-1.0774112	-0.1075689	0.05496525	0.04250263	0.12329228
Tcp11l1	700.15068	1.16873099	0.2249429	0.10352342	0.01398512	0.05162806
Tcp11l2	238.279001	-1.050341	-0.0708578	0.14407651	0.5115336	0.69562053
Tcp11x2	1.9838562	-1.0288005	-0.0409632	0.22086015	0.02379301	0.07873635
Tcta	33.7087654	1.06914395	0.09645611	0.21503157	0.30798529	0.50619599
Tctex1d2	246.972824	1.49342034	0.57862029	0.21803264	0.00077553	0.00472175
Tctn1	354.488663	1.60154802	0.67946705	0.16209301	2.59E-06	3.08E-05
Tctn2	855.432463	1.20519506	0.26926667	0.08031561	0.0003167	0.00218467
Tctn3	398.950841	1.19617967	0.2584341	0.10940069	0.00715785	0.02960674
Tdgf1	54.4605614	1.27533923	0.35088104	0.32204542	0.03188564	0.0984229
Tdp1	1700.78941	-1.010881	-0.0156132	0.06381977	0.79712158	0.88738397
Tdp2	1149.47907	1.04903072	0.06905692	0.06941199	0.29220771	0.48930467
Tdrd3	3704.00874	1.07641763	0.10623793	0.06420718	0.08251498	0.20245488
Tdrd5	13.6554918	-1.0092677	-0.0133088	0.20979815	0.78801613	0.88115282
Tdrd7	1243.08396	1.26597719	0.34025141	0.07445991	1.42E-06	1.77E-05
Tead1	1769.76399	1.05506899	0.07733733	0.05566869	0.15029612	0.31183889
Tead3	534.357468	1.23393734	0.30326913	0.11688768	0.00290905	0.01423411
Tead4	578.400239	-1.9938501	-0.9955569	0.10697842	8.98E-22	5.97E-20
Tec	1.76876369	-1.0289176	-0.0411275	0.21997654	0.11291393	0.25453854
Tecpr1	829.03856	1.00322649	0.00464735	0.09169723	0.95612782	0.97889239
Tecpr2	518.664662	1.02569013	0.03659495	0.09323878	0.66372047	0.80543405
Tecr	3276.69676	1.0838382	0.11614941	0.07065709	0.08079892	0.19943512
Tedc1	1127.62851	1.10418479	0.14298164	0.10019375	0.10693951	0.24484274
Tedc2	370.428022	1.11455993	0.15647419	0.10344603	0.08555071	0.20806998
Tef	530.735953	1.3819159	0.46666982	0.12303981	2.31E-05	0.0002172
Tefm	264.274543	-1.1095373	-0.1499582	0.13856379	0.16979364	0.34086453
Tek	2.50237846	-1.0025974	-0.0037425	0.21393737	0.88178785	0.93670865
Telo2	661.780743	-1.0483276	-0.0680896	0.09599352	0.42662675	0.62469495
Telomerase-vert	450.054681	-1.1864377	-0.2466363	0.12638319	0.01993266	0.06841321
Ten1	103.923266	1.30238797	0.38115928	0.33602618	0.02591247	0.08382292
Tenm1	5.74893753	1.04471993	0.06311623	0.22219886	0.22171906	0.40954884
Tenm3	14.7231517	-344.67337	-8.429086	2.82530797	1.04E-09	2.19E-08
Tenm4	3.73404541	-1.0130279	-0.0186739	0.21465043	0.51383977	0.69757179
Tent2	1525.34998	-1.0480722	-0.0677381	0.07836879	0.35209577	0.55192034
Tent4a	2567.10486	-1.0111106	-0.0159408	0.0548764	0.76322059	0.86905384
Tent4b	3173.0961	-1.0223312	-0.0318627	0.05407719	0.54253673	0.72027106
Tent5a	1273.21811	-1.0625034	-0.0874674	0.07569977	0.21584809	0.4021391
Tent5d	221.006526	1.09704159	0.13361822	0.1440194	0.22909847	0.41766719
Tep1	1914.69452	-1.0575716	-0.0807553	0.07133237	0.22882446	0.41748237
Tepsin	651.75324	1.03892189	0.05508719	0.09778418	0.52754873	0.70881471
Terb1	2.59761706	-1.0101318	-0.0145435	0.21336978	0.67007594	0.80967875
Terf1	1130.71226	-1.0824601	-0.1143138	0.08239175	0.13155342	0.28351855
Terf2	1072.30299	-1.103777	-0.1424488	0.09602007	0.0969385	0.22798111

Terf2ip	226.357675	1.07308357	0.10176244	0.12129135	0.31596284	0.51496817
Tert	379.874268	1.05110277	0.07190373	0.10426672	0.43182207	0.62974052
Tesk1	411.789441	1.23942502	0.309671	0.10656699	0.00113409	0.0064625
Tesk2	210.94176	1.02831293	0.04027937	0.13129772	0.70081194	0.82981668
Tet3	1779.23779	-2.2009045	-1.1380965	0.07820298	3.26E-49	8.70E-47
Tex10	2941.57439	1.15165998	0.20371483	0.07297311	0.00295848	0.01441541
Tex14	32.2912759	-1.0573471	-0.0804491	0.20446712	0.39855007	0.59859613
Tex15	2.85430684	-1.043177	-0.0609839	0.22620944	0.06515838	0.17051663
Tex16	5.86947207	1.01761059	0.02518559	0.2147827	0.46975258	0.66177615
Tex2	2879.73656	1.19366804	0.25540167	0.05083715	2.32E-07	3.34E-06
Tex21	2.2468177	1.00728315	0.01046929	0.21391396	0.71127924	0.83695423
Tex22	6.26117203	-1.0325602	-0.0462259	0.2178338	0.3033231	0.50104048
Tex26	71.7019642	1.59740909	0.67573383	0.29565161	0.00151786	0.00824835
Tex261	4148.11923	1.13682718	0.18501295	0.0561414	0.0006216	0.00391093
Tex264	752.338362	1.29708187	0.37526954	0.08887655	5.67E-06	6.15E-05
Tex30	472.739483	-1.2347196	-0.3041835	0.09882469	0.00066947	0.00415903
Tex33	662.090722	1.07071874	0.09857956	0.09351529	0.24163161	0.43150638
Tex38	7.16376291	1.02673592	0.03806516	0.21137133	0.51753659	0.70073393
Tex52	5.29671406	-1.0222921	-0.0318075	0.21553271	0.39490002	0.59576837
Tex9	240.134274	1.04601589	0.06490477	0.12780968	0.53073444	0.71101319
Tfam	2084.08692	-1.0385913	-0.0546281	0.06729078	0.39207779	0.5933975
Tfap2a	2104.82651	-1.0311569	-0.0442639	0.06192853	0.45484611	0.64934566
Tfap2e	14.764144	-1.1399918	-0.1890234	0.3509158	0.00562863	0.02435902
Tfap4	1607.51512	-1.0973276	-0.1339943	0.06461646	0.0290598	0.09132876
Tfb1m	1542.71697	1.04241294	0.0599269	0.06466328	0.33118308	0.53110356
Tfb2m	1719.62594	-1.1768301	-0.234906	0.08211541	0.00198469	0.0103771
Tfcp2	436.108316	-1.0566402	-0.0794842	0.09464257	0.34870432	0.54835531
Tfdp1	4251.82622	-1.0179191	-0.0256229	0.06090987	0.66199733	0.80434277
Tfdp2	1144.50385	1.09799913	0.13487691	0.06955898	0.03997344	0.11768784
Tfe3	1614.58622	-1.1794804	-0.2381514	0.08054319	0.00143411	0.00784415
Tfeb	194.318694	1.13095845	0.17754593	0.1441248	0.11494234	0.25790472
Tfg	3733.94153	1.02811146	0.03999669	0.05250881	0.43243612	0.63037983
Tfip11	1154.32287	-1.0321376	-0.0456353	0.07612055	0.52041801	0.70307474
Tfpi	3377.05111	-1.3098294	-0.3893789	0.06286361	1.34E-10	3.30E-09
Tfpt	315.491255	-1.0221682	-0.0316326	0.12226195	0.75237975	0.86284553
Tfr2	27.0932842	-1.0008045	-0.0011602	0.19259612	0.98748497	0.99384683
Tfrc	9043.79588	-1.3252612	-0.4062767	0.07072054	1.97E-09	4.01E-08
Tgds	498.2787	-1.0527229	-0.0741257	0.08936841	0.36113908	0.56143102
Tgfb1	2302.42243	1.50145217	0.58635852	0.07796592	7.65E-15	3.00E-13
Tgfb1i1	1724.96442	1.69555142	0.76175454	0.07060719	3.43E-28	3.27E-26
Tgfb3	405.534844	1.46422557	0.55013783	0.10857132	5.11E-08	8.36E-07
Tgfbr1	1867.46328	-1.4469631	-0.5330282	0.07245401	2.77E-14	1.02E-12
Tgfbr2	4234.29654	-1.0157503	-0.0225458	0.06571101	0.71711225	0.84130487
Tgfbr3	1236.1031	-1.0059277	-0.0085266	0.08902846	0.91498906	0.95558004
Tgfbrap1	965.956422	1.10973624	0.15021682	0.09210913	0.07069565	0.1804046
Tgif1	715.419229	-1.2540053	-0.3265434	0.10270139	0.0004157	0.00276483
Tgm1	17.1689409	-1.0319925	-0.0454325	0.21000898	0.49589836	0.68225303
Tgm2	2507.89772	1.28992704	0.36728947	0.06641923	8.78E-09	1.62E-07
Tgs1	970.114769	1.0390269	0.05523301	0.08006404	0.45756848	0.6518519
Tha1	49.421102	1.46351734	0.54943984	0.38749524	0.0094677	0.03737479

Thada	1953.61956	-1.0378318	-0.0535727	0.05559565	0.31777627	0.51645699
Thap1	405.469523	-1.1467381	-0.197536	0.13410957	0.06943585	0.17846573
Thap11	101.945445	-1.0261209	-0.0372007	0.16570847	0.72324047	0.84393428
Thap12	2703.57879	-1.1299436	-0.1762508	0.05490345	0.00086866	0.00519981
Thap2	128.604972	-1.2247368	-0.2924717	0.22677457	0.0421801	0.12260656
Thap3	224.172421	1.03105435	0.04412038	0.132137	0.67547378	0.81273255
Thap4	3213.92467	1.07680217	0.10675322	0.06162943	0.07022576	0.17955928
Thap7	413.868177	-1.0351091	-0.0497828	0.12487391	0.61388868	0.7729422
Thbd	657.483254	-1.7575355	-0.8135538	0.10480939	6.78E-16	2.92E-14
Thbs1	4504.89398	-1.1659971	-0.2215642	0.08005405	0.00285248	0.01401621
Thbs2	38.7558859	-1.1168032	-0.1593749	0.24780888	0.1420745	0.3000782
Thbs3	1448.77076	1.09519159	0.13118328	0.07867277	0.07251617	0.18387324
Them6	139.556516	-1.0767611	-0.1066982	0.17527558	0.34816139	0.54770323
Themis	21.5937612	-561.00312	-9.131865	2.85237523	1.43E-12	4.53E-11
Themis2	9.02511787	1.00943616	0.01354967	0.20899628	0.80046125	0.8895557
Thg1l	565.531815	-1.0957303	-0.1318928	0.10016097	0.13594719	0.29040815
Thnsl1	412.259197	-1.074296	-0.1033915	0.1101253	0.27448643	0.46993205
Thoc1	1957.17159	-1.0737277	-0.1026281	0.07829101	0.15764428	0.32310771
Thoc2	4886.62178	-1.0640932	-0.0896245	0.04947895	0.06205219	0.16426711
Thoc2l	1408.74738	-1.0114148	-0.0163748	0.08239939	0.82900408	0.90788335
Thoc3	1517.40627	-1.0439641	-0.062072	0.06681104	0.32758286	0.52745833
Thoc5	4401.23038	-1.0633776	-0.088654	0.04797684	0.05539552	0.15024702
Thoc6	496.082067	1.15598436	0.20912188	0.10285439	0.02144289	0.07257157
Thoc7	893.566292	-1.0548199	-0.0769967	0.09939075	0.38219587	0.58397551
Thop1	2658.10116	1.02420308	0.03450181	0.06748817	0.5908951	0.75407115
Thpo	15.0220184	-1.1050746	-0.1441438	0.26473209	0.09331076	0.22176453
Thra	1693.26879	1.26760196	0.3421018	0.07359899	9.53E-07	1.24E-05
Thrap3	11801.7482	1.04442794	0.06271295	0.05766753	0.2579299	0.45026721
Thsd4	5.08625629	1.04524439	0.06384029	0.22631556	0.09735317	0.22853109
Thsd7a	555.437486	1.27600709	0.35163635	0.09003861	2.47E-05	0.00023094
Thtpa	853.898512	1.02390924	0.03408784	0.0909542	0.68104332	0.81646968
Thumpd1	2658.81682	-1.2691658	-0.3438805	0.06019883	3.27E-09	6.44E-08
Thumpd2	197.03321	-1.0268023	-0.0381585	0.13967311	0.71912077	0.84165712
Thumpd3	1438.62824	1.18860758	0.24927249	0.06697162	8.98E-05	0.00073965
Thyn1	252.742736	-1.1432785	-0.1931769	0.12353322	0.06133136	0.16289752
Tia1	284.834221	1.04236882	0.05986584	0.12539303	0.55896776	0.73259433
Tial1	3734.26036	-1.0858255	-0.1187923	0.05361748	0.02222792	0.07477408
Tiam2	1036.94031	1.13089914	0.17747026	0.07158888	0.00836035	0.0337523
Ticam1	130.007661	-1.1084276	-0.1485146	0.16884294	0.20771026	0.39250162
Ticrr	3051.90321	-1.078749	-0.1093592	0.05246235	0.03115934	0.09664678
Tifa	74.4192483	1.10210227	0.1402581	0.20468966	0.22861184	0.41744354
Tigar	843.574212	-1.1792213	-0.2378345	0.11177987	0.01437854	0.05276114
Tigd2	547.953386	-1.8936935	-0.9212029	0.10116855	6.67E-21	4.19E-19
Tigd3	24.2965422	1.08409454	0.11649058	0.22253898	0.24301804	0.43297095
Tigd5	34.4616855	1.12171471	0.1657058	0.282091	0.07895237	0.19600385
Tigit	28.8929777	-2.1900426	-1.1309589	0.51751224	0.00104028	0.00602752
Timeless	2558.02403	1.14393376	0.19400351	0.06472084	0.0016251	0.00874318
Timm10	946.036471	-1.0817717	-0.113396	0.09828304	0.19276695	0.37183325
Timm17a	2548.65137	-1.0505171	-0.0710997	0.0628403	0.23603486	0.42571906
Timm17b	894.984463	1.15302314	0.20542147	0.11739818	0.04008166	0.11777063

Timm21	1425.04967	-1.0329863	-0.0468212	0.08129232	0.53222209	0.71201878
Timm22	367.944414	-1.0588185	-0.0824552	0.09986359	0.35050529	0.55010693
Timm29	84.5819635	-1.1331265	-0.1803089	0.197994	0.14818983	0.30867285
Timm44	938.542084	-1.0314318	-0.0446484	0.08560305	0.56909385	0.73906044
Timm50	1025.72103	1.09918608	0.13643563	0.09121563	0.09810843	0.22975668
Timm8a1	1118.75017	-1.1890869	-0.2498541	0.09326368	0.00312553	0.01509729
Timm9	511.357046	-1.0254365	-0.0362382	0.10516644	0.69228057	0.8238679
Timmdc1	288.012908	-1.0227545	-0.0324599	0.11513061	0.73766766	0.85348047
Timp1	2026.30247	-1.6385126	-0.7123867	0.08823108	6.10E-17	2.89E-15
Timp2	9249.01518	1.34346107	0.42595452	0.06399102	5.69E-12	1.67E-10
Tinag	22.3881143	1.06148798	0.08608803	0.21892629	0.29769896	0.49564881
Tinagl1	5844.62804	1.41873264	0.50460274	0.0489796	8.25E-26	6.96E-24
Tinf2	263.226035	-1.095248	-0.1312576	0.14637396	0.23851916	0.42835139
Tiparp	4277.89453	-1.1083462	-0.1484086	0.09457744	0.07984874	0.19775664
Tipin	2228.63016	-1.0139495	-0.0199859	0.06824077	0.75675135	0.86499374
Tirap	83.6568501	1.07638238	0.10619068	0.19181322	0.3394669	0.53912361
Tjap1	806.618259	-1.0693886	-0.0967862	0.09529194	0.25381818	0.44520653
Tjp1	13740.4459	1.00301815	0.00434771	0.03893513	0.88566265	0.93871741
Tjp2	4559.91369	-1.1273045	-0.1728773	0.04841714	0.00023916	0.00173673
Tjp3	76.8902917	-1.0083694	-0.0120243	0.17381456	0.90427389	0.95011061
Tk2	188.694243	1.03352245	0.04756973	0.13597367	0.65447842	0.79983482
Tkfc	368.458685	1.13783675	0.18629359	0.11100189	0.05245769	0.14420237
Tkt	17080.3476	-1.0087188	-0.0125241	0.04354374	0.76466847	0.86962346
Tlcd1	167.958788	1.5108592	0.59536922	0.24337133	0.00120108	0.00675409
Tlcd4	6.43404173	-1.0590164	-0.082725	0.23493072	0.06210623	0.1643083
Tle1	309.772924	1.16824318	0.22434062	0.13094581	0.03810384	0.11327691
Tle2	93.7312246	1.99259389	0.9946477	0.23920892	1.90E-06	2.32E-05
Tle3	901.517769	-1.4009536	-0.4864092	0.08153837	3.88E-10	8.69E-09
Tle4	482.704373	1.09710541	0.13370214	0.09560557	0.11799186	0.26250012
Tle5	4817.84654	1.02350994	0.03352512	0.05214282	0.50782865	0.69219621
Tlk1	3113.66404	-1.0621129	-0.0869371	0.04869858	0.06642584	0.1729502
Tlk2	2029.56896	-1.0261308	-0.0372146	0.06496996	0.54722485	0.72401884
Tln1	4897.74759	-1.0229382	-0.032719	0.04958141	0.50014732	0.68576981
Tln2	2590.14597	1.15787946	0.21148507	0.06589859	0.00073359	0.00449203
Tlnrd1	88.1072488	-1.3347984	-0.4166219	0.26448808	0.01348601	0.04995831
Tlr1	80.9792511	-1.0781588	-0.1085697	0.19222689	0.32868667	0.52855589
Tlr2	272.13554	-1.4552693	-0.5412862	0.15938086	8.01E-05	0.00066862
Tlr3	147.675702	-1.3595184	-0.4430957	0.20415485	0.00406525	0.01875254
Tlr6	60.6560248	-1.081249	-0.1126988	0.20082451	0.30388286	0.50190039
Tlr7	4.49135568	-1.0077074	-0.0110768	0.21248089	0.76442934	0.86962346
Tlx1	3.57459271	1.01928042	0.02755101	0.21483631	0.45520675	0.64971571
Tlx2	3.81733674	-1.0382793	-0.0541946	0.22315675	0.1063172	0.24394112
Tm2d1	205.035837	-1.0302556	-0.0430023	0.12602723	0.67247245	0.81086907
Tm2d2	1413.87023	1.1469426	0.19779319	0.06797292	0.00213536	0.01100764
Tm2d3	425.945973	1.10232406	0.14054841	0.11894749	0.16053052	0.32677513
Tm4sf1	316.809563	-1.1960457	-0.2582725	0.12676632	0.01534648	0.05550209
Tm6sf2	2.43436981	1.0029548	0.00425659	0.21369049	0.87925582	0.93525426
Tm7sf2	12.4404761	1.0261735	0.03727467	0.20966973	0.55029196	0.72692841
Tm7sf3	223.723824	1.2069073	0.27131487	0.16045698	0.02837347	0.08955968
Tm9sf2	6353.04546	1.01443982	0.02068328	0.05185477	0.68236767	0.81710659

Tm9sf4	2014.53614	1.06049618	0.08473942	0.05951829	0.13794075	0.29342761
Tma16	2173.56915	-1.0424737	-0.060011	0.05991544	0.29562353	0.49315502
Tmbim1	380.538678	1.02966477	0.04217471	0.10859275	0.65380192	0.79954201
Tmbim4	215.850788	-1.0141802	-0.020314	0.13271865	0.84451107	0.91625195
Tmbim6	2943.90413	1.00053206	0.00076739	0.05931051	0.98836245	0.99449556
Tmc2	5.17354557	1.01225361	0.01757078	0.21331417	0.62775018	0.78249609
Tmc4	338.331497	1.09630262	0.13264609	0.10849118	0.15871745	0.32478763
Tmc6	1247.4904	1.17953242	0.23821507	0.09549168	0.00563638	0.0243761
Tmc8	250.700064	1.09561912	0.13174635	0.14096371	0.23133277	0.4203962
Tmcc1	676.173634	1.01671544	0.02391595	0.09439383	0.77860565	0.87673482
Tmcc2	413.679685	1.23339372	0.3026334	0.14665012	0.01093241	0.04219664
Tmcc3	12.8888383	-283.34625	-8.1464223	2.80446697	5.14E-09	9.85E-08
Tmco1	2848.88159	-1.0270436	-0.0384974	0.05803381	0.47706837	0.66738515
Tmco3	793.754216	1.03507292	0.04973241	0.08006552	0.49877607	0.68466797
Tmco4	127.483733	1.06077023	0.0851122	0.16193207	0.44873883	0.64443323
Tmco6	48.3510183	1.04987979	0.07022415	0.18081745	0.51148798	0.69562053
Tmed1	1779.27762	1.30309099	0.38193783	0.06631259	2.14E-09	4.34E-08
Tmed10	11927.1332	1.08252671	0.11440262	0.04035151	0.00385811	0.01797176
Tmed2	7971.52761	1.00371923	0.00535577	0.05186726	0.91865084	0.9579874
Tmed3	1217.73469	1.39824423	0.48361637	0.08809074	6.51E-09	1.23E-07
Tmed4	3024.80003	1.05598525	0.07858969	0.06287462	0.19022595	0.36893178
Tmed5	7036.23731	-1.0721132	-0.1004573	0.05539114	0.0603388	0.16069438
Tmed6	17.6619526	-1.0526581	-0.074037	0.21399114	0.35423751	0.55392853
Tmed7	3686.36225	-1.0695203	-0.0969639	0.07004571	0.14810901	0.30867285
Tmed8	182.437117	-1.1316025	-0.1783673	0.14948825	0.118839	0.26378923
Tmed9	4658.75711	1.06621622	0.09250004	0.0598801	0.1065578	0.24425013
Tmeff1	527.257982	-1.0375104	-0.0531258	0.11058719	0.57441657	0.74221064
Tmeff2	349.204093	1.22058801	0.28757632	0.15209158	0.01737402	0.06141314
Tmem101	1226.55588	1.04709219	0.06638846	0.06135116	0.25868763	0.45091693
Tmem104	552.903709	1.24937541	0.32120704	0.09853355	0.00032715	0.00224349
Tmem106a	192.848612	1.54127736	0.62412651	0.14928884	3.01E-06	3.51E-05
Tmem106b	1254.75496	1.19815455	0.26081401	0.09335709	0.00211626	0.01091356
Tmem106c	2571.74888	-1.0909465	-0.1255804	0.07186715	0.06273822	0.16560407
Tmem107	77.6454545	1.05741438	0.08054085	0.18006394	0.46020295	0.65424127
Tmem108	65.1569084	-10.535129	-3.3971361	0.86493255	3.22E-06	3.73E-05
Tmem109	984.176143	-1.0679744	-0.0948771	0.08653459	0.22948802	0.41806073
Tmem115	550.043402	-1.1428165	-0.1925937	0.1160014	0.05131391	0.14172729
Tmem116	81.5043987	1.06085591	0.08522871	0.1845061	0.43347835	0.63143708
Tmem120a	266.906693	1.4235434	0.50948648	0.15849962	0.00017338	0.00132199
Tmem120b	327.216031	-1.0088487	-0.0127098	0.13507872	0.90262422	0.94945197
Tmem121	42.8582164	-1.0313485	-0.0445319	0.1937115	0.62457694	0.78003613
Tmem123	1240.39282	-1.0394855	-0.0558697	0.08465325	0.47184636	0.66348539
Tmem126b	1237.76689	-1.1185787	-0.1616668	0.08906571	0.04537912	0.12955349
Tmem127	239.24496	-1.0883294	-0.1221153	0.12383574	0.23147526	0.42059547
Tmem128	377.634799	1.06282338	0.08790188	0.12788956	0.39773146	0.59792748
Tmem129	637.824108	-1.0506842	-0.0713291	0.09036838	0.38299119	0.58441721
Tmem131	4919.44486	-1.3233599	-0.4042055	0.05242878	2.81E-15	1.16E-13
Tmem131l	1330.98881	-1.1328706	-0.1799831	0.08354954	0.01908649	0.06597355
Tmem132a	2161.05055	-1.0051807	-0.0074549	0.08328442	0.91998795	0.95873208
Tmem134	116.973316	1.21017494	0.27521562	0.19566378	0.04243788	0.12316023

Tmem135	856.258153	1.02603454	0.0370793	0.07725773	0.60755346	0.76801885
Tmem138	126.774446	1.0255587	0.03641007	0.16782659	0.73280651	0.85067979
Tmem140	14.7024236	1.00744365	0.01069914	0.20544524	0.86613886	0.92774752
Tmem141	109.587005	1.00820087	0.0117831	0.16552031	0.91340266	0.95523619
Tmem143	55.2575628	-1.0043834	-0.0063101	0.17834434	0.94816521	0.97437186
Tmem147	1176.98107	1.01330747	0.019072	0.08262522	0.80322592	0.89146475
Tmem14a	797.013195	1.03226568	0.04581434	0.10223762	0.61130376	0.77127395
Tmem150a	83.2490261	-2.1913266	-1.1318045	0.27258275	1.72E-06	2.12E-05
Tmem151b	1300.37485	1.48803621	0.57340963	0.08874609	1.30E-11	3.64E-10
Tmem156	215.221786	-1.2088623	-0.2736499	0.23118207	0.0540096	0.14736165
Tmem158	7.28815133	1.04166132	0.05888629	0.22198664	0.21018221	0.39545402
Tmem159	190.368563	1.04285276	0.06053548	0.14542081	0.57748586	0.74410617
Tmem160	76.0145583	1.09303395	0.12833822	0.19135566	0.26964218	0.46476268
Tmem161a	132.467256	1.08551561	0.11838048	0.16094172	0.30118701	0.49905648
Tmem161b	724.546739	-1.1502887	-0.201996	0.08575004	0.01016376	0.03980365
Tmem164	1406.67262	1.22249936	0.28983371	0.0696133	1.17E-05	0.00011958
Tmem165	1957.14042	-1.0528355	-0.07428	0.06500099	0.2297244	0.41842309
Tmem167	170.632723	-1.0586931	-0.0822844	0.13758401	0.44171424	0.63907133
Tmem168	1989.86899	-1.1451355	-0.1955183	0.06753378	0.00221351	0.01134658
Tmem170	21.6439032	-1.0246493	-0.0351303	0.20479484	0.62739403	0.78236001
Tmem170b	4.7162762	1.00020049	0.00028921	0.21249318	0.99590576	0.99827695
Tmem175	862.685588	1.19602294	0.25824506	0.09841475	0.00352929	0.01670741
Tmem176a	29.3191854	-1.3325288	-0.4141667	0.42846618	0.02248708	0.07538851
Tmem176b	35.9116105	1.00679524	0.0097703	0.18785536	0.91714003	0.95692723
Tmem179b	11.3548507	1.01743182	0.02493212	0.20839198	0.67272406	0.81086907
Tmem18	894.91094	-1.0067846	-0.0097551	0.0857708	0.90047773	0.9485077
Tmem181a	464.881075	1.18697564	0.24729033	0.11359002	0.01200713	0.04558254
Tmem183a	2350.91861	1.03291925	0.04672747	0.0624156	0.43361305	0.63155823
Tmem184b	850.069573	-1.0489487	-0.0689442	0.09808439	0.42846417	0.62612663
Tmem185a	1088.76577	-1.1667754	-0.2225269	0.08671789	0.00505539	0.02234631
Tmem185b	220.055533	-1.015979	-0.0228706	0.1339476	0.82631071	0.90665159
Tmem186	1710.39228	1.03297935	0.04681141	0.06800351	0.46874549	0.66108441
Tmem19	357.239579	1.28493974	0.3617007	0.13260545	0.00141565	0.00775971
Tmem192	1753.96849	1.18617057	0.24631148	0.0633783	4.75E-05	0.0004174
Tmem198	6.74399497	-1.0136352	-0.0195386	0.20958192	0.71084432	0.83678696
Tmem198b	100.007132	1.11583846	0.15812818	0.18445326	0.19165951	0.37049331
Tmem199	664.278733	1.01208219	0.01732646	0.08820557	0.83028556	0.90866792
Tmem201	1131.91951	-1.0202495	-0.0289219	0.08540124	0.71124386	0.83695423
Tmem203	29.8246347	1.06870369	0.09586191	0.21199579	0.31918471	0.51762959
Tmem205	63.7236282	1.06650593	0.09289199	0.18428853	0.40088303	0.60080425
Tmem208	512.277031	-1.0417071	-0.0589497	0.10735263	0.52580558	0.70722065
Tmem209	1078.58827	-1.0192092	-0.0274501	0.07614681	0.69920698	0.82849333
Tmem214	818.825638	1.24984748	0.32175205	0.09417834	0.00019007	0.00143392
Tmem216	93.4304683	-1.032937	-0.0467522	0.17216141	0.6602157	0.80332096
Tmem217	4.32899513	-1.0048273	-0.0069476	0.21248108	0.84321075	0.91561666
Tmem218	176.851339	1.3658361	0.44978437	0.17144949	0.00127793	0.00710288
Tmem219	496.40614	1.23059215	0.2993527	0.09798717	0.00075924	0.00463354
Tmem220	5.97356878	1.01793542	0.02564604	0.21299189	0.55993264	0.73310886
Tmem222	102.966485	1.00706864	0.01016202	0.1635125	0.92794676	0.96368042
Tmem223	214.76503	1.08069811	0.11196357	0.14800607	0.31436749	0.51358852

Tmem229b	54.5825077	-1.2400156	-0.3103583	0.31877852	0.04247182	0.1232308
Tmem231	896.848894	1.12754639	0.17318679	0.09719689	0.04625609	0.13128616
Tmem232	9.60091906	-1.0724991	-0.1009764	0.24325057	0.07676627	0.19215162
Tmem234	38.4239492	1.04502079	0.06353165	0.19056802	0.52819144	0.70916386
Tmem235	5.45906803	1.02545351	0.03626209	0.2163071	0.34535706	0.54469637
Tmem237	394.848545	1.02373622	0.03384404	0.10379441	0.71131309	0.83695423
Tmem240	12.5755981	1.04010278	0.05672609	0.2127133	0.41834286	0.61725086
Tmem241	178.942564	-1.0027868	-0.0040149	0.13782923	0.96875451	0.98513498
Tmem242	781.694134	1.06535648	0.09133625	0.08032074	0.21782229	0.40439182
Tmem243	260.082034	1.15104286	0.20294156	0.14637719	0.07664724	0.19203035
Tmem245	1328.98688	1.07177555	0.10000281	0.07192579	0.13940068	0.29562851
Tmem246	11.8666359	1.04785099	0.06743357	0.21994361	0.28845302	0.48491905
Tmem25	22.4354697	1.11798328	0.16089861	0.27438131	0.08783528	0.21261714
Tmem250-ps	44.0533953	1.02169835	0.03096932	0.17994978	0.75861552	0.86622743
Tmem253	2.20802334	1.00303247	0.00436831	0.21395653	0.86421326	0.92713638
Tmem256	167.390869	1.77815978	0.83038496	0.19267758	1.25E-06	1.58E-05
Tmem259	1840.64761	1.06052175	0.08477421	0.07643951	0.234571	0.42447443
Tmem260	325.510196	-1.2006997	-0.2638754	0.11888816	0.00980491	0.03862264
Tmem263	554.644904	1.18017799	0.23900446	0.14064363	0.0353549	0.10673962
Tmem265	22.9419222	-1.0613541	-0.0859061	0.21087999	0.34836693	0.547892
Tmem267	239.712085	1.03954463	0.0559517	0.13263249	0.59588577	0.75879524
Tmem268	1523.48698	1.11549555	0.15768476	0.06379304	0.00941866	0.03720414
Tmem30a	1829.8517	1.07431661	0.10341924	0.06945306	0.10947444	0.24901339
Tmem30c	2.55668253	1.02545912	0.03626998	0.21819149	0.19635074	0.37705918
Tmem33	1041.98425	-1.0134614	-0.0192911	0.07950104	0.79307926	0.88496039
Tmem35a	2.21383312	-1.013161	-0.0188635	0.21574796	0.34484436	0.54415574
Tmem35b	116.391887	1.0029947	0.00431398	0.14926046	0.96920904	0.9853483
Tmem38a	27.2372119	-1.0012251	-0.0017664	0.19722173	0.98007842	0.99079336
Tmem38b	367.963648	-1.1234392	-0.1679221	0.1323227	0.11737439	0.26162624
Tmem39a	1387.18278	-1.0444246	-0.0627084	0.07779184	0.38586809	0.58698138
Tmem39b	715.063072	-1.1128579	-0.1542693	0.10614243	0.09502867	0.22476316
Tmem40	17.9845678	-1.0872241	-0.1206493	0.24908679	0.11217842	0.25330611
Tmem41b	1771.73541	1.10633394	0.14578692	0.08079261	0.05089745	0.14099474
Tmem42	253.972773	1.22565082	0.29354802	0.14916	0.01414868	0.05208387
Tmem43	3660.42934	-1.2703196	-0.3451915	0.05967165	2.02E-09	4.12E-08
Tmem44	250.559716	-1.226277	-0.2942849	0.15367939	0.01573258	0.05663324
Tmem50a	332.026485	1.00478392	0.00688528	0.11647792	0.9446506	0.97295716
Tmem50b	227.609918	1.08546306	0.11831062	0.13414963	0.26926807	0.46424279
Tmem51	608.22477	1.03199924	0.04544191	0.09643302	0.59886618	0.76173923
Tmem53	36.8567844	1.03795371	0.05374211	0.20213315	0.53164723	0.71171502
Tmem59	1299.62329	1.09702865	0.1336012	0.09180921	0.10678804	0.2446271
Tmem60	245.714362	-1.0064995	-0.0093465	0.14406068	0.93076494	0.96546527
Tmem62	426.300852	1.07659757	0.10647907	0.10302508	0.23939638	0.42914465
Tmem63a	259.386211	1.04749357	0.06694139	0.13297745	0.52617117	0.70748953
Tmem63b	777.374316	-1.0953209	-0.1313536	0.0813109	0.07988429	0.19775664
Tmem64	61.9725027	-1.3172239	-0.3975006	0.326737	0.02279421	0.07613919
Tmem65	309.283754	-1.7049193	-0.7697035	0.16038515	1.22E-07	1.86E-06
Tmem67	269.042675	1.00753291	0.01082696	0.12025889	0.91462356	0.9555461
Tmem68	128.338162	-1.1731273	-0.2303595	0.19388192	0.07750024	0.19356172
Tmem69	1687.31799	-1.0917929	-0.1266992	0.07452064	0.06863582	0.17694201

Tmem70	351.564744	-1.1525857	-0.204874	0.15167357	0.07859519	0.19534763
Tmem79	9.60133456	1.10340075	0.14195686	0.27124095	0.05808982	0.15600223
Tmem8	76.3400375	1.07145532	0.09957169	0.18243868	0.37318688	0.57484647
Tmem80	12.6824534	1.04820286	0.06791795	0.22003741	0.29085384	0.48747935
Tmem81	43.1020762	-1.2108961	-0.2760751	0.33391049	0.05101482	0.14115699
Tmem86a	5.8446836	1.0171066	0.02447089	0.21300151	0.56637269	0.73729312
Tmem86b	4.48280331	-1.0281667	-0.0400742	0.21629004	0.35637946	0.55646281
Tmem87a	1411.99513	-1.0071563	-0.0102876	0.06858013	0.87432829	0.93221108
Tmem87b	1729.08693	1.27831759	0.35424631	0.07767845	1.37E-06	1.72E-05
Tmem9	1061.80468	1.06155995	0.08618584	0.09673814	0.31826312	0.51688671
Tmem94	679.779386	1.1592696	0.21321612	0.1146349	0.03032779	0.09461738
Tmem97	873.858404	-1.0280606	-0.0399253	0.08655245	0.61349108	0.77269695
Tmem9b	194.774822	1.18239554	0.24171273	0.15042031	0.0405504	0.11889461
Tmf1	2713.44385	1.05876035	0.08237607	0.05714457	0.13460335	0.28845681
Tmlhe	721.590342	-1.0107549	-0.0154333	0.08830988	0.84771613	0.91809635
Tmod2	196.623704	1.18171167	0.24087807	0.16480305	0.05158959	0.14227397
Tmod3	6381.67488	-1.1973548	-0.2598507	0.05406255	6.61E-07	8.80E-06
Tmod4	58.8425942	3.01584358	1.59256161	0.32861889	7.50E-08	1.19E-06
Tmpo	9109.1808	-1.1418006	-0.1913107	0.0482474	4.65E-05	0.00040928
Tmprss11b	45.9775435	1.0303092	0.04307736	0.19048407	0.65168213	0.79791215
Tmprss11f	88.2180459	-2.8486833	-1.5102952	0.25518787	1.68E-10	4.07E-09
Tmprss6	1.82099282	1.01826853	0.02611807	0.21654678	0.28538597	0.48190417
Tmprss7	3.34891817	-1.0380469	-0.0538717	0.2228773	0.11831259	0.26303095
Tmtc1	167.860355	1.21407092	0.27985269	0.19445604	0.03911938	0.11563024
Tmtc3	6948.50118	-1.0115422	-0.0165564	0.05476525	0.75362468	0.86298066
Tmtc4	1196.90429	1.01269879	0.01820513	0.06997617	0.78355751	0.87900619
Tmub1	137.789203	-1.4615816	-0.5475304	0.22518023	0.00140922	0.00773439
Tmub2	387.94076	1.17370036	0.23106415	0.117459	0.02156601	0.0729112
Tmx1	5087.36721	-1.0674518	-0.0941709	0.05212522	0.06199758	0.16419041
Tmx2	2066.47818	-1.2778082	-0.3536713	0.06786362	4.99E-08	8.18E-07
Tmx3	10682.4307	1.00968675	0.01390777	0.05495497	0.79426898	0.88567075
Tmx4	529.614961	-1.0598332	-0.0838373	0.09494181	0.32408201	0.52351709
Tnc	2.20882857	-1.0125717	-0.018024	0.21500296	0.48646732	0.67514677
Tnfaip1	628.836535	-1.0821392	-0.113886	0.0949678	0.18069116	0.3565394
Tnfaip2	1180.1754	1.11833217	0.16134877	0.09348506	0.0550255	0.14943326
Tnfaip3	2232.69004	-1.044736	-0.0631384	0.06851422	0.32980047	0.52974846
Tnfaip6	14.7371095	-8.6703063	-3.116083	0.79763316	4.57E-06	5.10E-05
Tnfaip8	504.258603	-1.3776228	-0.4621809	0.13059541	6.14E-05	0.00052711
Tnfaip8l1	13.0038181	1.03305609	0.04691859	0.21118423	0.47110871	0.66281193
Tnfrsf12a	5809.35451	1.00098662	0.00142269	0.06236414	0.98171788	0.99108517
Tnfrsf19	120.800421	-1.2226512	-0.2900129	0.25425925	0.04794814	0.13487074
Tnfrsf1a	821.419836	-1.1923075	-0.2537564	0.10722214	0.00712938	0.0294985
Tnfrsf1b	292.600667	1.02895037	0.04117339	0.11396735	0.67009559	0.80967875
Tnfrsf21	3778.44717	1.092462	0.1275831	0.04700515	0.00532682	0.023281
Tnfrsf25	3.08926963	-1.0163345	-0.0233753	0.21460887	0.48736667	0.67570424
Tnfrsf4	6981.66744	1.05841681	0.08190788	0.05735298	0.13890946	0.29485173
Tnfrsf9	150.6487	-1.5655098	-0.6466325	0.21667682	0.00023424	0.00170687
Tnfsf11	3.58685743	1.03290525	0.04670792	0.21989676	0.20188578	0.3844443
Tnfsf12	308.769211	1.89462327	0.92191101	0.16165406	8.82E-10	1.89E-08
Tnfsf13	13.2810278	1.06544334	0.09145388	0.23469365	0.13699354	0.2920029

Tnfsf15	1.603964	-1.0274044	-0.0390041	0.22023816	0.03798965	0.11306864
Tnfsf4	103.719013	1.17259284	0.22970215	0.19478561	0.07871257	0.19552219
Tnik	955.57443	1.17051049	0.22713786	0.07765062	0.00170907	0.00912594
Tnip1	2454.62965	-1.0868277	-0.1201233	0.08229031	0.11289922	0.25453854
Tnip2	264.773486	-1.0631335	-0.0883228	0.14441449	0.41864256	0.61733754
Tnk2	1074.77377	1.54895578	0.63129596	0.08268058	2.60E-15	1.07E-13
Tnks	1650.69499	1.1005963	0.13828538	0.06583365	0.02691627	0.08620179
Tnks1bp1	6399.3538	-1.1299979	-0.1763201	0.04188278	1.70E-05	0.00016668
Tnks2	2141.44912	1.0865819	0.11979692	0.06077908	0.03943574	0.11639903
Tnn	4.73303353	1.05178843	0.07284453	0.22951514	0.10112997	0.23511343
Tnnc1	4.88863849	1.05479937	0.07696862	0.2329574	0.0512675	0.14166019
Tnni1	12.8612151	1.05381697	0.07562432	0.21804553	0.31641328	0.51535335
Tnnt2	15.8889702	1.05014506	0.07058863	0.21211455	0.38240395	0.58400032
Tnpo1	5236.40989	-1.0916257	-0.1264782	0.05527171	0.0176083	0.06210418
Tnpo2	1209.22371	-1.0068445	-0.0098409	0.07045718	0.88186622	0.93670865
Tnpo3	7642.79493	-1.070129	-0.0977847	0.05207578	0.0525212	0.14428053
Tnrc18	2082.5117	-1.0736445	-0.1025164	0.09216108	0.21578273	0.40212057
Tnrc6a	2427.95412	-1.1455601	-0.1960532	0.06801474	0.00231419	0.01179187
Tnrc6b	5363.08347	-1.193523	-0.2552264	0.06086508	1.22E-05	0.00012431
Tnrc6c	1618.58545	1.10958724	0.1500231	0.05649487	0.00579172	0.02487993
Tns1	9444.54518	-1.0000246	-3.54E-05	0.05342341	0.99893614	0.99959215
Tns2	210.082339	1.03157055	0.0448425	0.1262381	0.66177299	0.80429905
Tns3	1168.60964	1.04521858	0.06380467	0.06931122	0.3310154	0.53103409
Tns4	3.68587685	1.01493591	0.02138862	0.21394768	0.55121095	0.72763936
Tnxb	474.792598	-1.3067309	-0.385962	0.15928242	0.00284931	0.01401466
Tob1	509.451653	1.0743738	0.10349603	0.0899218	0.20425272	0.38802867
Tob2	364.289643	-1.1307396	-0.1772667	0.11626813	0.07263155	0.18405739
Toe1	633.723064	1.04231147	0.05978646	0.10619766	0.51764635	0.70080037
Togaram1	2078.755	-1.0904577	-0.1249338	0.06719572	0.04909749	0.1373488
Togaram2	1.92347538	-1.0230508	-0.0328777	0.21861055	0.08210969	0.20170882
Tollip	2619.32913	1.19172564	0.25305214	0.07011408	0.00013554	0.00106196
Tom1	728.03998	1.1735697	0.23090353	0.10317549	0.0117412	0.04485678
Tom1l2	613.436644	-1.0623098	-0.0872046	0.09235682	0.29407136	0.49139762
Tomm22	778.009578	1.01957563	0.0279688	0.0802342	0.70776642	0.8347011
Tomm34	4041.59072	1.17118924	0.2279742	0.05177621	5.50E-06	6.00E-05
Tomm40	978.78483	-1.0749149	-0.1042224	0.08633413	0.18600749	0.3632307
Tomm40l	953.620808	-1.0289231	-0.0411352	0.07882448	0.57387407	0.74199183
Tomm5	125.646034	-1.1435378	-0.1935041	0.16453554	0.10784413	0.24599061
Tomm6	182.659279	-1.102551	-0.1408454	0.19629166	0.2304602	0.41934591
Tomm7	83.3703037	-1.1654156	-0.2208445	0.24344666	0.09589623	0.2260638
Tomm70a	6947.92156	-1.1271508	-0.1726805	0.05931466	0.00237854	0.01206695
Tonsl	1242.48798	1.12358653	0.16811124	0.06753346	0.00861562	0.03460837
Top1	18154.7953	1.13824142	0.18680658	0.05341858	0.0002978	0.0020733
Top1mt	606.716157	-1.1119622	-0.1531077	0.10677253	0.09955545	0.23216919
Top2a	52631.2413	-1.0125037	-0.0179272	0.04910422	0.70971397	0.83614789
Top2b	9014.90319	-1.0447368	-0.0631395	0.04613726	0.16071636	0.32699756
Top3a	851.951258	1.02805216	0.03991346	0.08674044	0.6166807	0.77454526
Top3b	1013.38209	1.12703677	0.17253458	0.07531141	0.01417951	0.05216529
Topbp1	8382.49501	1.12520189	0.17018388	0.04450071	8.22E-05	0.00068385
Topors	2406.64999	-1.0713766	-0.0994657	0.08101152	0.18366481	0.36013259

Tor1a	626.580718	-1.0632542	-0.0884865	0.10323945	0.32714859	0.52694247
Tor1aip1	5218.13888	1.03834937	0.05429195	0.04948303	0.25708072	0.44927749
Tor1aip2	1313.94195	-1.4888835	-0.5742308	0.07567088	4.15E-15	1.69E-13
Tor1b	1035.52963	1.00107629	0.00155192	0.0847178	0.98496506	0.99201214
Tor2a	411.954985	-1.1758978	-0.2337627	0.1057453	0.01200945	0.04558254
Tor3a	372.427907	1.13395366	0.18136168	0.11194377	0.05993498	0.15981831
Tor4a	41.8058489	1.14461248	0.19485924	0.24637493	0.1204197	0.26628282
Tox4	2216.79724	1.00133892	0.00193036	0.05862881	0.97325185	0.98781495
Tpbg	1042.13748	-1.1094901	-0.1498967	0.09047317	0.06695521	0.17383385
Tpcn1	2599.75438	1.16002154	0.21415159	0.06055786	0.00022115	0.00162906
Tpcn2	150.579873	-1.0374136	-0.0529912	0.14785784	0.62430353	0.77992278
Tpd52l2	5252.31009	1.15173014	0.20380272	0.05314547	7.33E-05	0.00061881
Tpgs1	174.692514	-1.0370353	-0.052465	0.1369273	0.62022991	0.77697362
Tpgs2	446.421875	-1.026745	-0.038078	0.09192556	0.64513253	0.79348514
Tph1	2.71042669	1.00829661	0.0119201	0.21429045	0.64751079	0.79526493
Tpk1	214.222677	1.44820474	0.53426558	0.17044989	0.00019908	0.00148875
Tpm1	1861.70366	-1.0554553	-0.0778655	0.0712378	0.24581517	0.43597712
Tpm2	1090.03366	1.57520609	0.65554059	0.0877834	9.42E-15	3.64E-13
Tpm3	2588.44934	-1.2611862	-0.3347813	0.10625701	0.00044159	0.0029234
Tpm4	34394.8359	1.30375308	0.38267066	0.05692893	4.45E-12	1.33E-10
Tpmt	587.612368	-1.0106867	-0.0153359	0.08852721	0.84858474	0.91853734
Tpp2	3287.86004	-1.1251808	-0.1701568	0.05908139	0.00264272	0.01321131
Tppp3	2.51087782	1.00689277	0.00991005	0.21424127	0.69815768	0.82770938
Tpr	31633.8633	-1.0742768	-0.1033658	0.05741743	0.06080618	0.16173737
Tpra1	242.50615	1.00120652	0.00173959	0.13856397	0.98813008	0.99433986
Tprg	2.05701728	1.00997035	0.01431295	0.21369782	0.6578038	0.80145203
Tprkb	497.450478	-1.8605202	-0.8957061	0.10370044	4.64E-19	2.64E-17
Tprn	421.041019	-1.0575199	-0.0806848	0.10934724	0.39095667	0.59245839
Tpst1	1126.8373	1.31503196	0.39509787	0.08463771	6.81E-07	9.04E-06
Tpx2	8192.4856	-1.0227776	-0.0324925	0.0494635	0.50955776	0.69381749
Tra2a	1360.20442	-1.8054518	-0.8523599	0.06908924	4.51E-36	5.83E-34
Tra2b	4244.1502	-1.0343806	-0.0487671	0.05872141	0.38671733	0.58799394
Trabd	499.807623	1.09073701	0.1253033	0.09881885	0.15328152	0.31618941
Trabd2b	604.370996	1.99348978	0.99529621	0.11756776	1.97E-18	1.05E-16
Tradd	189.859385	1.01920556	0.02744506	0.14871404	0.79971055	0.88902995
Traf1	43.9855442	-3.7030181	-1.8887016	0.45359225	1.49E-06	1.86E-05
Traf2	1561.22306	-1.2358439	-0.3054966	0.0705282	5.02E-06	5.53E-05
Traf3	858.820832	-1.0141722	-0.0203027	0.07471917	0.77127345	0.87286097
Traf3ip1	1860.69052	-1.0738929	-0.1028501	0.06492826	0.09560129	0.22564627
Traf3ip2	340.656301	1.02684987	0.03822527	0.10684489	0.68128372	0.81646968
Traf4	720.268756	-1.1274763	-0.1730971	0.09261846	0.0378537	0.11274232
Traf5	319.384929	1.47742162	0.56308159	0.15339381	2.75E-05	0.00025415
Traf6	944.628352	1.04121662	0.05827024	0.09007887	0.47641293	0.66709819
Traf7	1748.53372	-1.0206423	-0.0294773	0.0727178	0.66576812	0.80655133
Trafd1	1100.41558	1.21598199	0.28212186	0.07629319	8.21E-05	0.00068366
Traip	483.665198	-1.1076778	-0.1475383	0.1034547	0.10388908	0.24022006
Trak1	888.191091	-1.0243598	-0.0347226	0.09748299	0.6890885	0.82183486
Trak2	4353.72664	1.03330506	0.04726624	0.05617918	0.38408952	0.58537304
Tram1	2044.04591	-1.0615752	-0.0862066	0.07327301	0.210026	0.39530312
Tram2	214.138961	1.00069638	0.00100432	0.11761328	0.99281877	0.99685733

Trap1	7864.36718	1.00266053	0.00383324	0.04695324	0.94813334	0.97437186
Trappc1	270.853564	-1.0038167	-0.0054959	0.12559364	0.95595815	0.97880456
Trappc10	2371.14084	1.17802368	0.23636854	0.06445632	0.00011958	0.00095453
Trappc11	1714.22204	1.09674502	0.13322815	0.06147606	0.02327004	0.07738557
Trappc12	1691.11533	1.03801196	0.05382306	0.07116653	0.42299449	0.62146569
Trappc2l	1721.15674	1.13286869	0.17998065	0.05581569	0.00081469	0.00491352
Trappc3	2391.44645	1.10158168	0.13957647	0.05773357	0.01192164	0.04534584
Trappc4	473.512152	1.00155935	0.00224791	0.13120962	0.9851126	0.99208274
Trappc5	205.299491	1.02901349	0.04126189	0.11840623	0.67804424	0.81472158
Trappc6a	139.691905	1.18871816	0.2494067	0.17951203	0.05354877	0.14642969
Trappc6b	527.315864	1.05461045	0.0767102	0.09962544	0.38595795	0.58704836
Trappc8	1523.16431	-1.0271513	-0.0386488	0.06764086	0.54648385	0.72332988
Trappc9	682.455862	1.30508881	0.38414798	0.09471899	1.13E-05	0.0001166
Trdmt1	766.36523	-1.0016047	-0.0023133	0.08538992	0.97900626	0.99034912
Treml2	4.13497655	1.00678527	0.00975602	0.21220768	0.79899414	0.88860215
Treml4	3.75496442	-1.0419565	-0.059295	0.22436169	0.11647654	0.26030434
Trex1	37.4123268	-1.0298215	-0.0423942	0.18815063	0.65920371	0.80239448
Trex2	8.73106678	-1.0778018	-0.1080919	0.24304834	0.11225054	0.25342428
Trhr2	2.7353693	1.0053288	0.00766743	0.2132939	0.8060489	0.8931309
Triap1	94.8987568	-1.046203	-0.0651628	0.16731567	0.54901213	0.72578384
Trib1	3133.52723	-1.5475313	-0.6299685	0.07313644	7.80E-19	4.30E-17
Trib3	316.875436	1.52664027	0.61036015	0.18465813	8.96E-05	0.00073835
Trim11	277.503469	-1.1599942	-0.2141176	0.13074694	0.04666373	0.13212527
Trim13	282.485451	-1.5183158	-0.6024719	0.13292733	6.31E-07	8.43E-06
Trim2	830.305444	-1.1702771	-0.2268502	0.09038525	0.005837	0.02502408
Trim21	260.405768	-1.2199194	-0.2867858	0.1340513	0.01033536	0.04030318
Trim23	760.009669	-1.0283218	-0.0402918	0.09600892	0.63800886	0.78957526
Trim24	1243.17999	1.07021715	0.09790355	0.07200163	0.14819188	0.30867285
Trim25	2443.95712	1.04374724	0.06177239	0.0687247	0.34246625	0.54197121
Trim26	923.34188	1.22873623	0.29717525	0.09523066	0.00058167	0.00370339
Trim27	2805.92815	-1.0693264	-0.0967022	0.05350018	0.06152366	0.16327284
Trim28	6417.91089	1.00413334	0.00595086	0.05505886	0.9019378	0.94921321
Trim3	486.83545	1.46654464	0.55242099	0.11050402	7.47E-08	1.18E-06
Trim32	543.520152	-1.0203546	-0.0290706	0.09973807	0.74125587	0.85589602
Trim33	3514.1327	1.32451365	0.40546271	0.05667895	1.91E-13	6.56E-12
Trim35	2396.15133	-1.3166031	-0.3968205	0.06839357	1.47E-09	3.01E-08
Trim36	3.43372714	-1.0139123	-0.0199328	0.21274542	0.62531798	0.78077618
Trim37	4128.7176	1.14380204	0.19383739	0.05449539	0.00022913	0.00167496
Trim41	749.142145	1.01782661	0.02549182	0.08613028	0.74686881	0.8595265
Trim44	2597.78963	-1.0249958	-0.035618	0.05598996	0.50938318	0.69365351
Trim45	631.206713	-1.0261706	-0.0372706	0.08900341	0.64580317	0.79400505
Trim46	32.9411546	1.03274534	0.04648455	0.19352253	0.61970805	0.77673241
Trim47	1671.23156	1.01886816	0.02696738	0.08372098	0.7270597	0.84644139
Trim59	509.83278	1.24150208	0.31208668	0.13462608	0.00582219	0.02498569
Trim6	525.713053	1.07322787	0.10195642	0.11907248	0.30719872	0.50567428
Trim62	40.268224	1.06770685	0.09451559	0.19626031	0.37561529	0.57741892
Trim65	10.8451139	-1.0676953	-0.0945	0.23571338	0.13162706	0.28352828
Trim66	3.30744445	1.05238745	0.07366595	0.23232892	0.0337911	0.1032127
Trim8	2578.05068	1.0795034	0.11036779	0.08114633	0.14426939	0.30341337
Trio	4569.87769	-1.1340205	-0.1814467	0.05064043	0.00021725	0.00160681

Triobp	3131.85878	-1.1462902	-0.1969723	0.07543739	0.0051634	0.02268284
Trip10	4380.79232	1.09626068	0.13259089	0.05132048	0.00767943	0.03136908
Trip11	5393.66502	-1.0430749	-0.0608427	0.05989294	0.28939755	0.48574196
Trip12	13935.173	1.05689394	0.07983061	0.04688027	0.0799479	0.19782357
Trip13	4389.34251	-1.1202846	-0.1638653	0.06037485	0.00457071	0.02061646
Trip4	1603.12083	-1.0230214	-0.0328363	0.06126496	0.575363	0.74298387
Trip6	2.5371645	-1.0410594	-0.0580524	0.22662558	0.0061417	0.02605999
Trir	2442.81061	-1.0118527	-0.0169993	0.07118529	0.79904826	0.88860215
Trit1	439.730082	-1.0196641	-0.028094	0.09402648	0.73926005	0.85471157
Trmo	293.699627	1.07506747	0.1044272	0.11587918	0.28750462	0.4837691
Trmt1	746.199685	-1.1177421	-0.1605873	0.07780855	0.02631925	0.08479618
Trmt10a	1218.50156	-1.045893	-0.0647353	0.07911017	0.37814387	0.58024766
Trmt10c	2253.85878	-1.1725632	-0.2296657	0.06483026	0.00019878	0.00148734
Trmt11	1082.32867	-1.1387729	-0.1874801	0.08141006	0.01258845	0.04724817
Trmt112	496.621902	1.01220233	0.0174977	0.11786229	0.86122407	0.92599298
Trmt13	982.461612	1.07369523	0.10258454	0.08505259	0.18903967	0.36729838
Trmt1l	1274.62892	-1.0430302	-0.0607809	0.07476403	0.38478897	0.58596609
Trmt2a	1042.26902	-1.0650067	-0.0908625	0.08366318	0.23674669	0.42617157
Trmt2b	591.735029	1.04122131	0.05827674	0.09667136	0.49964083	0.68548774
Trmt44	422.990445	-1.0127738	-0.0183119	0.10479509	0.84060695	0.91457236
Trmt6	4514.97525	1.02000139	0.02857112	0.06130825	0.62715546	0.78218883
Trmt61a	911.471249	-1.2258953	-0.2938358	0.11052157	0.00252549	0.01271181
Trmu	339.27193	-1.0138038	-0.0197784	0.14308822	0.85217028	0.92050774
Trnau1ap	565.969489	1.01294021	0.01854903	0.09904531	0.83446685	0.91129756
Trnp1	2.37370996	1.03374844	0.04788515	0.22147441	0.10800605	0.2463161
Trnt1	2973.65003	-1.1778181	-0.2361167	0.06663489	0.00019143	0.00144167
Troap	1158.56295	1.1322237	0.17915903	0.07742024	0.01294232	0.04832151
Trp53	1935.17047	-1.1702355	-0.2267988	0.09006476	0.00564468	0.02440375
Trp53bp1	3948.51854	1.07815127	0.1085596	0.04494594	0.01341013	0.04974914
Trp53bp2	1128.25058	1.08907406	0.12310206	0.08414434	0.11134143	0.25226101
Trp53i13	147.568368	1.04824186	0.06797163	0.17161913	0.53337391	0.71276025
Trp53inp1	5.51992136	1.01919666	0.02743245	0.2126117	0.55941949	0.73303638
Trp53inp2	156.594758	-1.0617601	-0.0864578	0.14921225	0.43171747	0.62965965
Trpc1	163.838951	1.21058274	0.27570169	0.1796858	0.03570889	0.10765612
Trpc4ap	2935.0368	1.10299829	0.14143056	0.05476181	0.00746816	0.0307019
Trpm3	1.66425728	-1.0226738	-0.0323461	0.2175596	0.22123696	0.4090122
Trpm4	344.185471	1.25542896	0.3281804	0.1402231	0.00487455	0.02165154
Trpm5	3.78503565	1.00288592	0.0041575	0.21223801	0.91174803	0.95428485
Trpm6	3.23407558	-1.0415515	-0.0587342	0.22607037	0.03904546	0.11551324
Trpm7	6568.94404	1.02384406	0.033996	0.05163772	0.49855008	0.68452928
Trps1	1017.6072	-1.2035424	-0.2672869	0.0830087	0.00051492	0.00333982
Trpt1	85.4210968	1.0997792	0.13721391	0.18374077	0.24571634	0.43595933
Trpv2	861.244499	-1.1080255	-0.1479911	0.09176547	0.07386962	0.1865674
Trrap	5738.97229	-1.1379	-0.1863738	0.05708074	0.00069352	0.00428351
Trub2	989.040844	1.14414929	0.1942753	0.07051512	0.00345949	0.01642114
Tsacc	167.169653	-1.1336751	-0.1810072	0.19885234	0.14563638	0.30521725
Tsc1	1260.67623	1.00771512	0.01108785	0.07785244	0.87953497	0.93547351
Tsc2	2207.87152	1.43942684	0.52549447	0.07585169	6.13E-13	2.02E-11
Tsc22d1	4962.12789	-1.888047	-0.9168947	0.08254532	8.28E-30	8.83E-28
Tsc22d2	1645.59365	1.21292991	0.27849619	0.07562444	8.83E-05	0.00072858

Tsc22d3	473.245622	1.35115521	0.43419341	0.11178338	1.89E-05	0.00018188
Tsc22d4	1341.21176	-1.0525287	-0.0738596	0.09274649	0.37546106	0.57736718
Tsen2	467.520092	1.33495875	0.41679516	0.13064414	0.00025899	0.00185158
Tsen34	324.892442	1.02771777	0.03944413	0.12393189	0.69872138	0.82824051
Tsen54	350.595219	1.07794197	0.10827952	0.13079878	0.30443827	0.50256561
Tsg101	2329.04915	1.06183747	0.08656296	0.0739665	0.21213888	0.39787687
Tsga10	167.657332	1.07625752	0.10602332	0.13865667	0.32829672	0.52808662
Tshz1	2330.2104	-1.0138399	-0.0198299	0.05292774	0.69807125	0.82770938
Tsku	197.793456	-1.1097158	-0.1501902	0.14651382	0.18043488	0.35619832
Tsnaxip1	14.6612056	-1.0625942	-0.0875907	0.22335536	0.25633681	0.44840565
Tspan10	2.53565703	-1.0182205	-0.02605	0.21567912	0.38624705	0.58741835
Tspan12	248.716897	-1.4609697	-0.5469263	0.16696913	0.00011993	0.00095662
Tspan14	309.428247	-1.0744932	-0.1036563	0.11023825	0.27458559	0.46993205
Tspan17	308.39848	1.29654683	0.37467432	0.14474155	0.00200875	0.01046446
Tspan2	67.5779843	-1.0193898	-0.0277058	0.17460165	0.78653886	0.88053373
Tspan3	2891.93017	1.00392093	0.00564564	0.0591101	0.92146315	0.95951415
Tspan31	2176.39955	1.04383429	0.0618927	0.09335131	0.46284366	0.65644677
Tspan4	443.59896	1.28080484	0.35705066	0.13865144	0.00231073	0.01178242
Tspan5	876.185259	-1.1380788	-0.1866004	0.09926367	0.03455994	0.10500779
Tspan6	3355.06067	1.20880821	0.27358537	0.0550525	2.77E-07	3.94E-06
Tspan7	3364.76881	1.53180337	0.61523112	0.05899678	2.26E-26	1.94E-24
Tspan8	4.09221095	-1.0473899	-0.0667986	0.22869604	0.04785173	0.13472103
Tspan9	1600.58698	-1.2450263	-0.3161762	0.07351167	5.35E-06	5.86E-05
Tspo	530.447831	-1.2056656	-0.2698298	0.15556028	0.02654502	0.08533041
Tspoap1	909.794971	1.12304884	0.16742067	0.10182828	0.06247233	0.16499459
Tspyl1	1103.46599	-1.0678223	-0.0946716	0.06909299	0.14604898	0.30575052
Tspyl2	604.713436	1.05661445	0.07944905	0.09931299	0.36734959	0.56880796
Tsr1	2936.90554	-1.1162139	-0.1586135	0.0565808	0.00356638	0.01685185
Tsr2	3134.89932	-1.0562284	-0.0789218	0.06686441	0.21352545	0.39933082
Tsr3	739.060329	1.07978815	0.11074829	0.08622984	0.1604485	0.3266601
Tssk1	6.1388743	1.03800708	0.05381629	0.22044107	0.23980086	0.42944771
Tssk4	28.8407484	1.04548407	0.06417109	0.20317107	0.47236655	0.66385249
Tssk6	4.38512205	1.00158474	0.00228449	0.20983616	0.96330754	0.98254201
Tsta3	578.995271	1.06468558	0.09042744	0.10964139	0.3390153	0.53860673
Tstd2	1488.82866	-1.00763	-0.010966	0.08282001	0.88501843	0.93818984
Ttbk1	1.84696819	-1.0092269	-0.0132506	0.21494639	0.54455683	0.72212972
Ttbk2	505.063741	1.13194098	0.17879874	0.10825453	0.05785348	0.15559587
Ttc13	2266.29492	1.07877392	0.10939255	0.05822006	0.05057862	0.14021036
Ttc14	3221.59637	1.04142795	0.05856303	0.06478551	0.34316762	0.54257927
Ttc17	1272.65793	-1.0418011	-0.0590798	0.06187936	0.3179882	0.51667032
Ttc19	72.1385399	-1.0469991	-0.0662602	0.18544197	0.52358778	0.70549906
Ttc21a	21.3112143	1.10651709	0.14602573	0.26481172	0.09749866	0.22883069
Ttc21b	2487.80341	-1.0027635	-0.0039813	0.0587271	0.9430682	0.97213355
Ttc23	824.10452	1.07157139	0.09972797	0.08836672	0.21590481	0.40214004
Ttc25	20.2760486	1.25467244	0.32731076	0.60301834	0.01858085	0.06464441
Ttc26	956.551943	1.33142693	0.41297325	0.08371381	1.65E-07	2.46E-06
Ttc27	3887.7134	1.01467105	0.02101209	0.05306771	0.68351443	0.81791871
Ttc28	2097.44602	1.18437822	0.24412987	0.06188736	3.74E-05	0.00033673
Ttc3	12227.0269	1.2282841	0.29664429	0.04650076	6.94E-11	1.76E-09
Ttc30b	335.879407	1.12053756	0.16419101	0.11679679	0.09778417	0.22929072

Ttc32	120.088256	-1.2043907	-0.2683035	0.18769005	0.04327041	0.12508042
Ttc33	1303.36307	1.00249183	0.00359048	0.07774758	0.96125486	0.98152343
Ttc36	47.0686396	1.0513802	0.07228447	0.19443948	0.47333316	0.66426478
Ttc37	3369.67548	-1.0504848	-0.0710553	0.05121713	0.15247028	0.31487093
Ttc38	117.502741	1.06072653	0.08505276	0.154111	0.44669322	0.64300935
Ttc39b	360.154362	-1.6217636	-0.6975635	0.12198361	9.89E-10	2.09E-08
Ttc39c	332.32155	-1.0464036	-0.0654395	0.1268564	0.525862	0.70722229
Ttc39d	5.70185121	1.04545361	0.06412905	0.22212274	0.23132531	0.4203962
Ttc4	1532.73781	-1.0088133	-0.0126592	0.07829913	0.86145889	0.92611368
Ttc41	10.0461476	-1.0199733	-0.0285314	0.21107691	0.58607415	0.74971139
Ttc5	1005.07235	1.16016694	0.21433241	0.0730589	0.00179967	0.00956194
Ttc7	2037.48668	-1.0777251	-0.1079893	0.07133392	0.10724138	0.24514515
Ttc7b	956.802673	1.20375715	0.26754437	0.07230677	8.86E-05	0.00073015
Ttc8	577.056165	1.39963474	0.48505038	0.12039828	8.33E-06	8.79E-05
Ttc9c	183.33662	1.05497303	0.07720612	0.12981628	0.45892659	0.65306039
Ttf1	1358.49632	1.03407476	0.04834049	0.07684595	0.5012345	0.68649781
Tti1	2444.7654	1.30090156	0.37951179	0.0555197	2.05E-12	6.38E-11
Tti2	862.003094	-1.1019233	-0.1400238	0.09251299	0.092702	0.22072724
Ttk	3495.13922	-1.0450409	-0.0635594	0.05865582	0.25369586	0.44518411
Ttl	577.07604	1.12986712	0.17615312	0.11088566	0.06611266	0.17227485
Ttll11	11.113462	-1.0185969	-0.0265832	0.207441	0.66760695	0.80795231
Ttll12	2853.02078	-1.1566265	-0.2099231	0.0598792	0.00025236	0.0018131
Ttll13	44.8382514	-1.4424833	-0.5285546	0.3956454	0.01117761	0.04299855
Ttll3	118.353425	1.15610433	0.2092716	0.17828171	0.09461888	0.22408354
Ttll4	2634.55722	-1.0600824	-0.0841765	0.0565668	0.12420599	0.27249165
Ttll5	1483.75568	1.27350283	0.34880217	0.08152974	5.22E-06	5.73E-05
Ttll6	9.54552629	1.0131938	0.01891016	0.20820137	0.74404548	0.85736145
Ttll7	186.777798	1.30509507	0.3841549	0.18416296	0.00650799	0.02730671
Ttll9	2.53548717	1.00534558	0.0076915	0.21373781	0.78238338	0.87827816
Ttpal	4232.84099	1.47483175	0.56055038	0.05149972	1.85E-28	1.79E-26
Ttyh2	73.4953295	-1.0919417	-0.1268958	0.2184438	0.23619952	0.42585726
Ttyh3	359.793422	-1.0711589	-0.0991725	0.13292013	0.34742493	0.54708216
Tuba1a	17933.1146	1.11225053	0.15348179	0.07327277	0.02491417	0.0813952
Tuba8	27.3863201	1.30356733	0.3824651	0.66055292	0.01881143	0.06519869
Tubal3	4.56706652	-1.0479638	-0.0675889	0.22753123	0.09701089	0.22806194
Tubb4a	1.77021118	-1.0232928	-0.033219	0.21829097	0.14265452	0.30085683
Tubb4b	10775.5598	-1.0041316	-0.0059484	0.06344071	0.92036401	0.95899324
Tubb6	4218.85863	-1.2020042	-0.265442	0.04991393	4.47E-08	7.38E-07
Tubd1	1338.17466	1.14851351	0.19976782	0.07621998	0.00497064	0.02203248
Tube1	830.103865	-1.0886755	-0.122574	0.08209886	0.10468377	0.24118689
Tubg1	3574.44795	1.15567804	0.20873954	0.05891118	0.00022031	0.00162477
Tubg2	7.87676344	1.01356673	0.01944108	0.21180803	0.66356805	0.80543405
Tubgcp2	4594.25541	1.19032539	0.251356	0.05941404	1.05E-05	0.00010874
Tubgcp3	2132.61001	1.2709689	0.34592872	0.07158088	3.85E-07	5.33E-06
Tubgcp5	997.708549	1.11139583	0.15237273	0.07070897	0.02211489	0.07445249
Tubgcp6	2700.60622	-1.0474324	-0.0668571	0.06542	0.28189404	0.47790913
Tufm	3505.41146	-1.0528217	-0.0742612	0.05338609	0.13887311	0.294837
Tuft1	860.464179	-1.2054596	-0.2695833	0.08095635	0.00034394	0.00234853
Tulp2	1.60487344	-1.0077434	-0.0111283	0.21514712	0.52502702	0.70669288
Tulp3	1191.19714	-1.1301428	-0.176505	0.07848904	0.01533087	0.05546128

Tulp4	2559.76254	1.19600668	0.25822545	0.05876722	4.93E-06	5.45E-05
Tusc2	16.619518	-1.0061739	-0.0088797	0.19616771	0.91164806	0.95425818
Tusc3	1084.22782	1.27557689	0.35114987	0.08626604	1.25E-05	0.00012693
Tut1	852.907709	-1.1586185	-0.2124056	0.09666385	0.01422224	0.05229955
Tut7	4491.72202	1.04126713	0.05834023	0.04823853	0.21666279	0.4029572
Tvp23b	135.139112	1.08671648	0.11997559	0.15640403	0.29246509	0.48954368
Twf1	4056.4718	-1.0044326	-0.0063808	0.0630355	0.91496861	0.95558004
Twf2	878.477914	1.00513578	0.00739041	0.08820527	0.92750596	0.96330074
Twist1	69.3079805	-1.0554249	-0.0778239	0.17943241	0.47373769	0.66461283
Twist2	88.4477484	1.47772987	0.56338256	0.30493829	0.0048579	0.02160753
Twistnb	1645.30262	-1.1111177	-0.1520116	0.07858936	0.03713932	0.11105385
Twnk	1825.27755	-1.2319003	-0.3008854	0.06181057	3.94E-07	5.44E-06
Twsg1	18192.1469	-1.0280281	-0.0398797	0.04764309	0.39111604	0.59250195
Txlna	3004.73684	1.00533243	0.00767263	0.05378212	0.88342901	0.93735821
Txlng	1056.52669	-1.107032	-0.146697	0.08071594	0.04922782	0.13753726
Txn1	13839.3191	-1.3752836	-0.4597291	0.05508336	1.28E-17	6.46E-16
Txndc11	1124.62996	1.08976125	0.12401209	0.08243511	0.10243001	0.23744634
Txndc12	632.493908	1.04422515	0.06243281	0.08972732	0.44445203	0.64099039
Txndc15	652.085623	1.08529049	0.11808125	0.11423868	0.22455453	0.41301653
Txndc16	717.052036	1.07410662	0.10313721	0.08745889	0.19599314	0.37652552
Txndc17	303.753853	-1.2357898	-0.3054334	0.16592651	0.01680457	0.05961411
Txndc5	10441.2254	1.10150823	0.13948027	0.05756486	0.01143021	0.04382548
Txnip	843.106653	-3.2214837	-1.6877253	0.11398562	6.23E-51	1.86E-48
Txnl1	3549.32354	-1.0910723	-0.1257467	0.05636372	0.02046357	0.06992219
Txnl4b	533.204106	1.20879655	0.27357145	0.10300233	0.0029479	0.01438575
Txnrd1	22666.4885	-1.340475	-0.4227443	0.04622092	1.21E-20	7.47E-19
Txnrd2	339.170513	1.28380935	0.36043097	0.12652868	0.0010326	0.00599694
Tyk2	398.209863	1.08982829	0.12410084	0.11405671	0.20133947	0.38368917
Tymp	3.58430303	1.00270405	0.00389585	0.21190414	0.92068074	0.95920569
Tyro3	772.246061	1.24014862	0.31051303	0.08870903	0.00014701	0.00114137
Tysnd1	17.378594	-1.0152452	-0.0218282	0.19903504	0.78059392	0.87721801
Tyw1	318.124469	1.00771944	0.01109404	0.10191941	0.90242491	0.94945197
Tyw3	489.796629	1.07643485	0.106261	0.10418118	0.24460432	0.43495021
Tyw5	366.830658	-1.001978	-0.0028508	0.09913155	0.96866557	0.98512274
U1	16803.063	-1.0384149	-0.054383	0.21067309	0.4644395	0.657618
U12	8.45863597	-1.0521898	-0.073395	0.22735781	0.15125723	0.31327489
U2	15186.2684	-1.01068	-0.0153263	0.18963347	0.87157649	0.93041135
U2surp	5116.97478	-1.0798968	-0.1108935	0.06730207	0.08191967	0.20145728
U3	3244.4599	-1.0758303	-0.1054505	0.24518285	0.08058526	0.19909299
U4	1630.6086	-1.0428123	-0.0604795	0.22142924	0.23266013	0.42197031
U5	55.8128761	-1.0759204	-0.1055713	0.24848428	0.04570644	0.13025574
U6	99.6603771	-1.2889143	-0.3661564	0.23473349	0.01821663	0.06365853
U8	6.35809325	-1.0830919	-0.1151557	0.25752566	0.02294759	0.07649198
UGP2	2300.90236	1.17997182	0.23875241	0.05996678	3.33E-05	0.00030305
USB1	298.684215	-1.0535215	-0.0752197	0.12210503	0.45442434	0.64917763
USP16	2440.7729	1.08213618	0.11388206	0.06096118	0.05101927	0.14115699
Uaca	2939.56129	-1.1000146	-0.1375227	0.05555085	0.0102139	0.03995116
Uap1	2604.45014	-1.0871297	-0.1205241	0.05350503	0.0198157	0.06807163
Uap1l1	780.152222	1.44816821	0.53422918	0.11357564	3.32E-07	4.66E-06
Uba1	12067.0834	-1.0627821	-0.0878459	0.05390349	0.09215208	0.21974462

Uba2	3783.10273	1.0180233	0.02577059	0.06481996	0.67582824	0.81289763
Uba3	2846.22302	-1.0042749	-0.0061543	0.05795379	0.91127956	0.95410634
Uba5	1105.63039	-1.1129562	-0.1543969	0.07057502	0.01974105	0.06790624
Uba6	1944.47094	-1.0872186	-0.1206421	0.0691328	0.06454331	0.16949566
Uba7	41.0606944	1.11373026	0.15539986	0.23013483	0.17441005	0.34762607
Ubac1	969.076208	1.06317985	0.08838567	0.07788151	0.2227593	0.41111463
Ubac2	357.846033	1.19001062	0.25097445	0.14041647	0.02733701	0.08715456
Ubald1	467.974721	-1.2776955	-0.353544	0.14889472	0.00389208	0.01809713
Ubap1	1153.78519	1.00521249	0.0075005	0.0756537	0.91636417	0.95653724
Ubap2	5295.27008	1.00267414	0.00385282	0.05158575	0.94071424	0.9712702
Ubash3b	6122.06824	1.15020063	0.20188554	0.06958262	0.00211548	0.01091356
Ube2a	626.92897	-1.0433143	-0.0611739	0.09060585	0.45611016	0.6503867
Ube2c	5307.40812	1.24965148	0.32152579	0.07820052	1.22E-05	0.00012439
Ube2cbp	243.057901	-1.0476864	-0.067207	0.14374093	0.53442295	0.71345954
Ube2d1	2026.5335	1.06893143	0.0961693	0.07019865	0.14678123	0.30688224
Ube2d2a	429.771331	1.00008255	0.00011909	0.12197317	0.99199236	0.99619177
Ube2e1	1133.59944	-1.0427193	-0.0603508	0.07574306	0.39349577	0.59456014
Ube2e2	1069.06104	1.06154183	0.08616122	0.09135508	0.29730411	0.49518472
Ube2e3	1157.95558	-1.0368574	-0.0522175	0.08199341	0.48982532	0.67731675
Ube2g1	1201.10044	-1.2906042	-0.3680466	0.08242433	1.97E-06	2.40E-05
Ube2g2	1004.29647	-1.0623945	-0.0873196	0.0801388	0.2380646	0.42777494
Ube2h	4698.29242	1.00489188	0.00704028	0.04993197	0.88640077	0.93903357
Ube2j1	897.609937	-1.0442585	-0.0624789	0.08282751	0.41249601	0.6114962
Ube2k	5119.54588	-1.1459747	-0.1965751	0.06600631	0.0016825	0.00901038
Ube2l6	25.2932028	-1.5141627	-0.5985202	0.50947952	0.01032183	0.04027493
Ube2m	827.27432	-1.0839679	-0.116322	0.08470022	0.13428833	0.28790518
Ube2n	1757.83188	-1.1063542	-0.1458134	0.07355432	0.03432645	0.10447181
Ube2o	2229.663	-1.1309266	-0.1775053	0.06697048	0.00511949	0.02252856
Ube2q1	1825.1137	1.03097738	0.04401268	0.06199431	0.45880814	0.6530184
Ube2q2	3580.02473	1.06815792	0.09512496	0.07514147	0.17612631	0.34986813
Ube2r2	1914.74623	1.0217591	0.0310551	0.06923003	0.63757658	0.7892098
Ube2t	1241.00187	1.01274783	0.018275	0.07227617	0.7891664	0.88205406
Ube2w	184.627495	1.01473431	0.02110203	0.1395058	0.84396071	0.91592523
Ube2z	2013.43768	1.06158872	0.08622495	0.06636753	0.17147259	0.34337407
Ube3a	2453.75341	-1.1483271	-0.1995336	0.06441735	0.00111612	0.00638567
Ube3b	4330.01919	1.24090918	0.31139753	0.0618703	1.61E-07	2.41E-06
Ube3c	3454.66636	-1.8479901	-0.885957	0.06436213	3.01E-44	6.33E-42
Ube4a	2835.36579	-1.1367409	-0.1849035	0.05661559	0.00068594	0.00424485
Ube4b	6543.55492	1.09240987	0.12751426	0.04186664	0.00208232	0.01076886
Ubfd1	2454.88343	1.00293645	0.00423019	0.05663169	0.95829926	0.98010487
Ubiad1	2107.03603	-1.0064099	-0.0092181	0.08179577	0.90219783	0.94933088
Ubl3	2638.24682	-1.0983788	-0.1353757	0.05141622	0.0065986	0.02765067
Ubl4a	303.984137	1.1495425	0.2010598	0.11907094	0.0467078	0.13219772
Ubl7	992.498417	-1.038471	-0.0544609	0.0743927	0.43401764	0.63179158
Ublcp1	373.229616	-1.2807615	-0.3570018	0.13948742	0.0023577	0.01197075
Ubn1	2492.05533	-1.0817249	-0.1133336	0.05944715	0.0466679	0.13212527
Ubn2	1517.94802	-1.0498273	-0.070152	0.0710385	0.2944002	0.49169056
Ubox5	330.235527	1.1269668	0.17244502	0.11404541	0.07680065	0.19215162
Ubp1	1083.55673	1.11431066	0.1561515	0.10527621	0.09003106	0.21613528
Ubqln1	8134.14262	1.0492892	0.06941236	0.04957219	0.14990167	0.31130567

Ubqln2	3465.36487	1.22347071	0.29097956	0.05594299	7.43E-08	1.18E-06
Ubqln4	2473.87489	1.04101069	0.05798489	0.06382944	0.34075919	0.54021887
Ubr1	1461.17683	1.09134543	0.12610781	0.07234562	0.06345946	0.16708096
Ubr2	7083.93827	-1.1310182	-0.1776222	0.04508553	5.61E-05	0.00048536
Ubr3	3824.1195	1.23009504	0.29876978	0.04653336	5.01E-11	1.31E-09
Ubr4	7903.23962	-1.3231691	-0.4039974	0.04668554	1.13E-18	6.15E-17
Ubr5	11601.719	1.09383487	0.12939496	0.03631005	0.00029515	0.00205927
Ubr7	2457.81078	1.11470018	0.15665572	0.0587663	0.00544067	0.02368162
Ubtd1	209.204127	-1.2834568	-0.3600347	0.17176576	0.00710051	0.02939803
Ubtd2	1136.89657	1.02605184	0.03710362	0.07290255	0.58906528	0.75240733
Ubtf	10525.2536	1.06659766	0.09301607	0.04971476	0.05430295	0.14798835
Ubxn1	2007.57755	-1.0495521	-0.0697737	0.07442435	0.31592288	0.51496817
Ubxn11	43.1365061	1.10293726	0.14135072	0.2216584	0.20740364	0.39226939
Ubxn2a	2110.75456	1.00310138	0.00446742	0.06001413	0.93808613	0.97004225
Ubxn2b	856.354439	-1.0280778	-0.0399495	0.08806362	0.61989225	0.77676022
Ubxn4	2201.70576	1.0023369	0.0033675	0.07246588	0.96005298	0.98111152
Ubxn6	1195.61414	1.18967268	0.2505647	0.07096691	0.00018399	0.00139603
Ubxn7	8024.47146	-1.037274	-0.052797	0.04681805	0.22687524	0.41553786
Ubxn8	1021.12809	-1.0982579	-0.1352169	0.08016323	0.06811634	0.17594372
Uchl5	1715.25446	-1.4432642	-0.5293354	0.08384056	3.74E-11	9.93E-10
Uck2	6011.87177	-1.2745307	-0.3499661	0.0501615	8.44E-13	2.74E-11
Uckl1	484.335275	1.27850448	0.35445721	0.10277172	0.00014225	0.00110705
Uevld	1385.0665	-1.0008411	-0.001213	0.0671211	0.98291299	0.99129521
Ufc1	474.684659	-1.0085049	-0.0122181	0.09143702	0.88183122	0.93670865
Ufl1	1769.69001	1.00281489	0.00405532	0.06503318	0.94850461	0.9744714
Ufm1	1800.31	-1.0474709	-0.0669101	0.06756631	0.29601374	0.49369457
Ufsp1	24.8183246	1.06153456	0.08615134	0.2078799	0.36492737	0.56594669
Ufsp2	1392.16332	-1.0906951	-0.1252479	0.07467524	0.07249481	0.18387324
Ugcg	2809.25113	1.02910305	0.04138745	0.05492057	0.43490858	0.63258532
Uggt1	9348.73565	1.06408666	0.08961565	0.04858626	0.05669331	0.15308627
Uggt2	745.309071	1.01880008	0.02687097	0.09290371	0.74926214	0.86088869
Uhmk1	490.234828	-1.2041713	-0.2680406	0.10974516	0.00539629	0.0235284
Uhrf1	3869.3885	-1.0117159	-0.0168043	0.04892276	0.72577034	0.84550835
Uhrf1bp1	1257.41165	1.01359801	0.0194856	0.0708388	0.77139155	0.87286097
Uhrf1bp1l	8365.84202	-1.0226016	-0.0322442	0.05286057	0.52834656	0.70920152
Uhrf2	2145.258	-1.1242144	-0.1689172	0.07052523	0.01093292	0.04219664
Uimc1	1413.78537	-1.0307844	-0.0437426	0.07076142	0.51001079	0.69413921
Ulk1	536.637891	1.06594137	0.09212808	0.11863277	0.35388772	0.5534799
Ulk2	188.378733	1.00338947	0.00488171	0.13402549	0.9639666	0.9827954
Ulk4	442.665627	1.65605456	0.7277502	0.11227606	8.07E-12	2.32E-10
Umps	5153.34663	-1.011007	-0.015793	0.05173159	0.75156534	0.86237477
Unc119	410.866988	1.29275903	0.37045338	0.11579893	0.00031623	0.0021838
Unc119b	260.181526	1.00989532	0.01420575	0.12438792	0.88959389	0.94124868
Unc13d	4.29353262	1.01089038	0.01562656	0.21161621	0.72226665	0.84343968
Unc45a	1587.21777	1.07292952	0.10155532	0.07604271	0.15268988	0.31522277
Unc45b	1.99368886	-1.0134024	-0.0192072	0.21536861	0.42307432	0.62148931
Unc50	661.061658	1.08139986	0.11290008	0.10873047	0.23388185	0.42334698
Unc5b	170.59961	1.02351247	0.03352868	0.14845402	0.75716849	0.86530011
Unc5c	2.66284389	-1.0274406	-0.039055	0.21938158	0.13408466	0.28766111
Unc80	17.6621288	-1.0360275	-0.0510623	0.21306272	0.42406632	0.62254064

Unc93b1	216.829134	-1.2051973	-0.2692694	0.16978774	0.03376752	0.10318749
Ung	790.889853	-1.145482	-0.1959548	0.08465961	0.01167719	0.04465218
Unk	489.88891	-1.0035345	-0.0050901	0.10427518	0.95441666	0.97802764
Unkl	221.434663	1.30638631	0.38558158	0.18355272	0.00621067	0.02632652
Upb1	294.918553	-2.4004332	-1.2632948	0.14066949	1.48E-20	9.05E-19
Upf1	3564.13491	-1.0722369	-0.1006237	0.06537376	0.10481877	0.24145448
Upf2	2989.76555	-1.0164713	-0.0235695	0.06527079	0.70443595	0.83202831
Upf3a	1075.64072	1.19609918	0.25833702	0.09097024	0.00188185	0.00991222
Upk1a	38.2072341	-1.0168095	-0.0240493	0.19387492	0.77974801	0.87680583
Uprt	1647.7346	-1.3677787	-0.4518348	0.07232921	7.66E-11	1.94E-09
Uqcc1	2706.05866	1.0054218	0.00780087	0.05497921	0.88401542	0.9377474
Uqcc2	1375.60324	1.08807056	0.12177212	0.10561255	0.18862637	0.3667735
Uqcc3	301.391499	1.0723466	0.10077129	0.1288182	0.33565104	0.53545328
Uqcr10	567.469235	-1.0292782	-0.041633	0.11885859	0.67386507	0.81160319
Uqcr11	44.7677744	1.09574524	0.13191242	0.21810037	0.22899323	0.41766719
Uqcrc1	3114.88446	1.10396344	0.1426924	0.06635144	0.02345369	0.07784316
Uqcrc2	2705.7978	-1.0382026	-0.054088	0.06109674	0.35494237	0.55469218
Uqcrfs1	4367.10575	-1.1047702	-0.1437463	0.05557519	0.00724933	0.02993677
Uqcrq	1133.61687	1.02469688	0.0351972	0.07672023	0.62369095	0.77930951
Urb1	2025.11814	-1.0802663	-0.1113869	0.07002798	0.0913614	0.21851
Urb2	2519.45951	1.01719312	0.02459361	0.05948921	0.66918126	0.80915726
Urgcp	1777.80246	1.51522311	0.59953024	0.08174582	2.64E-14	9.77E-13
Uri1	2825.39135	1.00193742	0.0027924	0.07270277	0.96738643	0.98424124
Urm1	1661.58076	1.04467884	0.06305949	0.06571346	0.31341416	0.51216169
Urod	284.27545	1.03466627	0.0491655	0.11916839	0.62138524	0.77778489
Uros	214.677453	1.03006615	0.04273699	0.1365895	0.68785519	0.82096571
Use1	195.418403	1.05388201	0.07571335	0.13297425	0.4734861	0.66433264
Usf1	932.475752	-1.2406936	-0.3111469	0.0798304	3.41E-05	0.00030934
Usf2	638.802301	1.01645728	0.02354958	0.08242095	0.75785171	0.86561668
Usf3	1182.37361	-1.4071551	-0.4927813	0.0768297	2.29E-11	6.25E-10
Ush2a	59.5754014	-5.032027	-2.3311397	0.46201198	2.11E-08	3.68E-07
Uso1	3783.73671	1.31432959	0.3943271	0.04743732	2.24E-17	1.10E-15
Usp1	7199.4968	1.00649641	0.00934203	0.05563489	0.86251173	0.92665838
Usp10	6345.61215	-1.2949508	-0.3728972	0.04280886	7.82E-19	4.30E-17
Usp11	1273.73787	-1.1659962	-0.2215631	0.07833381	0.00236205	0.01198808
Usp13	854.082193	-1.1278077	-0.1735211	0.10448002	0.05825377	0.15631144
Usp14	1828.15041	-1.1496914	-0.2012467	0.07264723	0.00317653	0.01532054
Usp15	4584.48274	-1.0692729	-0.09663	0.05483109	0.06786332	0.17556295
Usp18	4.816228	1.01655439	0.02368741	0.21290617	0.58370616	0.74847607
Usp19	3411.24518	-1.01852	-0.0264743	0.05545779	0.62062899	0.777138
Usp20	272.625392	1.13169697	0.17848771	0.1291309	0.09261112	0.22059286
Usp24	6884.00379	-1.1140715	-0.1558419	0.05146201	0.00177622	0.00946081
Usp25	4501.37132	-1.1282117	-0.1740378	0.06063067	0.00268114	0.01336396
Usp28	6433.33341	1.11761963	0.16042927	0.04712041	0.00047057	0.00308495
Usp3	646.300227	-1.0181576	-0.0259609	0.09065986	0.75136841	0.86230329
Usp30	1699.11241	1.10830366	0.14835321	0.05837926	0.00805658	0.03264941
Usp31	1693.613	-1.0380041	-0.0538122	0.07134651	0.42295209	0.62146569
Usp32	3628.15892	1.20414931	0.2680143	0.04997817	3.47E-08	5.78E-07
Usp33	1319.78436	1.25130625	0.32343493	0.08041087	1.77E-05	0.00017227
Usp34	9987.62983	1.10466144	0.14360428	0.04750243	0.00193591	0.01014688

Usp35	158.301631	1.16981325	0.22627824	0.19603278	0.08333723	0.20396444
Usp36	3685.53286	-1.0735606	-0.1024036	0.05884832	0.06979542	0.17885163
Usp37	2413.4232	1.08059388	0.11182441	0.05692285	0.04135082	0.12069693
Usp38	2397.21197	-1.1092926	-0.14964	0.06163476	0.01093046	0.04219664
Usp39	1692.77443	-1.9986236	-0.9990068	0.07932393	1.59E-37	2.24E-35
Usp4	2786.9742	1.12747223	0.1730919	0.05406949	0.0008973	0.00533879
Usp40	2910.57267	1.01857718	0.02655531	0.05835237	0.63671173	0.78844056
Usp42	1914.93317	-1.0260505	-0.0371018	0.06460608	0.54642664	0.72332988
Usp45	2412.15238	-1.2612977	-0.3349089	0.07208032	9.83E-07	1.27E-05
Usp46	2284.73423	-1.1295034	-0.1756886	0.07039857	0.00799545	0.0324736
Usp47	8236.65155	1.08129412	0.112759	0.04280045	0.00711413	0.0294449
Usp48	3565.18336	-1.0459404	-0.0648006	0.05293241	0.24128785	0.43102738
Usp49	217.103482	1.17226975	0.22930458	0.18083375	0.07228792	0.18351333
Usp50	1.86465439	1.02853384	0.04058925	0.22067483	0.03004329	0.09386695
Usp51	139.804586	-1.4312991	-0.5173251	0.18464421	0.00058569	0.0037213
Usp53	613.632621	-1.0091908	-0.013199	0.10415662	0.88790673	0.93988646
Usp54	1553.23251	-1.0098596	-0.0141547	0.06563596	0.82020975	0.90327432
Usp6nl	2476.73565	1.02229096	0.03180587	0.0672009	0.62267705	0.77857423
Usp7	10270.2788	-1.0415853	-0.058781	0.0532074	0.25384766	0.44520653
Usp8	4057.61374	-1.02048	-0.0292479	0.04708847	0.5349014	0.71398218
Usp9x	12925.7108	1.00507594	0.00730451	0.0394399	0.85650018	0.9234717
Ust	1664.19578	1.02608925	0.03715622	0.06220765	0.5330306	0.71237579
Utp11	2297.99958	-1.1689562	-0.2252209	0.07461138	0.00127529	0.00709664
Utp14a	2552.81963	-1.4399959	-0.5260647	0.0795788	5.60E-12	1.64E-10
Utp15	4948.34889	-1.2050664	-0.2691126	0.04985929	2.84E-08	4.84E-07
Utp18	2553.48618	-1.0295268	-0.0419814	0.06923807	0.52112295	0.70343361
Utp20	11705.9566	-1.0548865	-0.0770878	0.04381562	0.07237648	0.18370175
Utp23	280.826041	-1.174485	-0.2320282	0.13451231	0.03506939	0.10612778
Utp25	2047.13869	-1.0748814	-0.1041775	0.06120446	0.07511995	0.18898008
Utp3	1981.1246	-1.1991257	-0.2619829	0.07183396	0.00011157	0.00089663
Utp4	3584.99543	-1.1011828	-0.139054	0.05175539	0.00551954	0.0239563
Utp6	2842.15011	-1.0670458	-0.0936221	0.07018481	0.15705768	0.32219332
Utrn	6347.1974	-1.0962548	-0.1325832	0.05657861	0.01485901	0.05416731
Uvrag	827.679707	-1.0786149	-0.1091798	0.08583686	0.16402392	0.33209205
Uvssa	693.438315	1.17303669	0.23024814	0.09711677	0.00835707	0.03374971
Uxs1	1216.04131	-1.0262938	-0.0374438	0.09040078	0.64724511	0.79509108
Uxt	319.712244	-1.0885441	-0.1223998	0.12294978	0.22903095	0.41766719
VMA21	2433.29182	-1.0160549	-0.0229784	0.06267563	0.70087583	0.82981668
Vac14	1099.85325	1.14853943	0.19980039	0.09094925	0.01553209	0.05606257
Vamp1	10.9085999	1.02425598	0.03457631	0.2107951	0.5529927	0.7287148
Vamp2	545.776938	1.46319974	0.54912673	0.13079285	3.31E-06	3.81E-05
Vamp3	555.383625	1.03174268	0.0450832	0.13936132	0.67223088	0.81086907
Vamp5	19.5824181	-113.5907	-6.8277009	1.33195787	1.70E-10	4.11E-09
Vamp7	1139.46216	1.02650803	0.03774491	0.07305465	0.58320653	0.74830245
Vamp8	1022.97664	-1.7232847	-0.7851611	0.08940067	1.41E-19	8.23E-18
Vangl1	1873.90224	-1.1967057	-0.2590684	0.06855846	6.61E-05	0.00056383
Vangl2	885.202766	1.07147322	0.0995958	0.07095689	0.13624513	0.29079652
Vars	3169.5681	1.06417784	0.08973926	0.0743883	0.19774611	0.37850808
Vars2	1052.18326	-1.0177291	-0.0253536	0.06779875	0.69288891	0.82436212
Vash1	261.46015	1.03490585	0.04949953	0.11890878	0.60614818	0.76704121

Vash2	525.77496	1.06295711	0.08808338	0.0954922	0.3029254	0.50070638
Vasn	1458.88189	-1.0303224	-0.0430959	0.08098888	0.56420528	0.73589476
Vasp	833.600913	1.10357621	0.14218626	0.07778813	0.0492409	0.13754381
Vat1	9849.40734	1.17052888	0.22716052	0.06104996	0.00010275	0.00083359
Vav2	723.168533	1.06530071	0.09126073	0.08781202	0.25468809	0.44629303
Vav3	3.60083135	-1.0039984	-0.0057569	0.21341018	0.84333594	0.91567498
Vax1	106.621769	1.15282679	0.20517577	0.2025936	0.11372651	0.25567507
Vcam1	35.4107984	-1.1231723	-0.1675793	0.26933234	0.10422169	0.2405116
Vcl	6802.82543	-1.3023048	-0.3810672	0.05705322	5.92E-12	1.73E-10
Vcp	8990.99307	-1.0284788	-0.0405121	0.04888619	0.39404764	0.59497293
Vcpip1	1824.48081	-1.1411671	-0.19051	0.06977118	0.00377648	0.01764283
Vcpkmt	235.339399	-1.080336	-0.1114801	0.13898882	0.30300365	0.50077109
Vdac2	5163.28464	-1.0929492	-0.1282263	0.05665137	0.01869052	0.06483227
Vdac3	1749.5716	1.04454833	0.06287925	0.07839647	0.38902859	0.5908069
Vdr	2904.19761	-1.0702991	-0.098014	0.05715968	0.07458664	0.18796129
Vegfa	655.19645	-1.2204006	-0.2873548	0.09698781	0.00089415	0.00532383
Vegfb	317.525857	1.08849912	0.12234024	0.13580772	0.25628987	0.44838477
Vegfc	1452.38017	-1.11618	-0.1585697	0.07101145	0.01755579	0.06193828
Vegfd	17.1078043	-1.0577855	-0.0810471	0.21819611	0.30629742	0.50452231
Vezf1	3501.03951	1.048122	0.06780665	0.05389083	0.19368233	0.37326171
Vezt	2065.88748	-1.2357428	-0.3053784	0.08917565	0.00020144	0.00150374
Vgf	39.4388118	-1.3912552	-0.4763871	0.46561169	0.01683779	0.05968236
Vgll3	575.473135	1.30489033	0.38392856	0.09874814	2.31E-05	0.00021698
Vgll4	540.390524	-1.2271454	-0.2953062	0.11071546	0.00247077	0.01249038
Vhl	242.865432	1.43422964	0.52027603	0.15045187	7.07E-05	0.00059883
Vil1	2.74750185	-1.0004385	-0.0006325	0.21283079	0.98282411	0.99129521
Vill	133.449521	1.34679981	0.42953542	0.23350987	0.00849396	0.03421622
Vim	57418.0897	-1.2316952	-0.3006452	0.05495779	1.57E-08	2.79E-07
Vinac1	6.82541718	1.0305707	0.04344348	0.21304786	0.45388896	0.6486298
Vipas39	854.359317	-1.0118647	-0.0170164	0.08263932	0.82269803	0.90419748
Virma	2013.53753	-1.0973633	-0.1340413	0.06380543	0.02717956	0.08676056
Vkorc1	79.1913817	1.01435507	0.02056276	0.16030111	0.84912093	0.91884095
Vkorc1l1	196.567241	-1.1386775	-0.1873592	0.15086218	0.10415429	0.24044268
Vldlr	1592.22437	1.29474636	0.37266951	0.08186746	1.31E-06	1.65E-05
Vmac	13.9256249	1.02331296	0.03324744	0.20662832	0.62656951	0.7819148
Vmp1	2476.4712	-1.0528791	-0.0743398	0.06829954	0.24981403	0.44124022
Vnn1	385.785192	1.17062181	0.22727506	0.13007546	0.03463307	0.10515516
Vopp1	87.9879401	-1.1686499	-0.2248428	0.21917521	0.09179145	0.21917026
Vps11	2245.88873	1.116126	0.1584999	0.08378347	0.04050672	0.11882096
Vps13a	8285.21008	1.03804825	0.0538735	0.05778292	0.33323348	0.53306209
Vps13b	3392.33227	1.20809105	0.27272919	0.06905089	3.13E-05	0.00028754
Vps13c	6803.13421	-1.0204806	-0.0292487	0.05561665	0.58474979	0.74932628
Vps13d	3758.71034	1.04224851	0.05969931	0.04861049	0.20752208	0.39237755
Vps16	1000.57889	1.11474044	0.15670782	0.07805493	0.03075231	0.09568548
Vps18	963.809449	-1.0443989	-0.0626728	0.08978389	0.44090402	0.63840629
Vps25	2037.31039	1.11339964	0.15497153	0.07976514	0.03593566	0.10814827
Vps26a	3289.94764	1.13410574	0.18155516	0.0728314	0.00789858	0.03210076
Vps26b	1553.8222	1.02050245	0.02927965	0.06838407	0.65220121	0.79842293
Vps26c	538.859089	1.01671872	0.02392061	0.10602478	0.79625696	0.88665261
Vps28	267.578484	1.15622052	0.20941658	0.14275456	0.06429047	0.16893558

Vps29	2630.25633	1.10323931	0.14174576	0.06454674	0.02100301	0.07136568
Vps33a	1407.72908	1.02230384	0.03182404	0.06407954	0.60338764	0.76513519
Vps33b	858.144006	1.11966368	0.16306545	0.09804694	0.06231391	0.16468767
Vps35	5046.1599	1.06322358	0.088445	0.06032434	0.12529109	0.27411373
Vps35l	3325.27227	1.21350957	0.27918549	0.05271326	4.81E-08	7.89E-07
Vps36	2869.14045	1.0212509	0.03033735	0.05920477	0.5934264	0.75662309
Vps37a	978.559195	1.1560079	0.20915126	0.0952387	0.01475947	0.05391185
Vps37b	2003.73374	-1.1999521	-0.2629768	0.08119389	0.00048728	0.00317985
Vps37c	991.891236	1.00162867	0.00234777	0.07168313	0.9732948	0.98781495
Vps37d	78.2552223	1.03672954	0.05203958	0.17903276	0.61907739	0.77619471
Vps39	2921.6702	1.07336579	0.10214181	0.0506751	0.0377592	0.11253965
Vps41	2427.93036	1.08765297	0.12121832	0.07124104	0.07065021	0.18035657
Vps45	806.048437	1.0873458	0.12081082	0.11158784	0.207808	0.39262838
Vps4a	2007.63628	1.0139363	0.01996702	0.07565678	0.77867179	0.87673482
Vps50	2502.72263	1.02700308	0.03844051	0.06187824	0.51653962	0.69986443
Vps51	584.806645	1.10740222	0.14717932	0.09254603	0.0775965	0.19372659
Vps52	1546.218	1.02644682	0.03765889	0.09319879	0.65454153	0.79983563
Vps54	1728.29018	-1.0436229	-0.0616005	0.08325967	0.420424	0.61905069
Vps72	1750.10614	1.08770126	0.12128238	0.0635023	0.04500825	0.12883927
Vps8	1707.93694	1.21295354	0.27852429	0.0650782	7.44E-06	7.92E-05
Vps9d1	500.735712	1.26822079	0.34280593	0.1122878	0.00058775	0.00372914
Vrk1	906.281031	-1.0932253	-0.1285907	0.09374762	0.12594049	0.27488458
Vrk2	322.218188	-1.0642947	-0.0898977	0.13307083	0.39459973	0.59565027
Vrk3	316.818429	-1.0274382	-0.0390516	0.13883079	0.71226691	0.83736812
Vsig1	3.09150285	1.02687136	0.03825546	0.2173819	0.29840435	0.49630742
Vsig10	824.587013	1.20958323	0.27451004	0.09973716	0.00217732	0.01119242
Vstm2l	3.98107503	7.86330762	2.97513629	1.6332892	0.00262381	0.01312931
Vstm4	416.308873	-1.0462744	-0.0652613	0.12121391	0.51527767	0.69893127
Vstm5	131.588552	-1.0792028	-0.1099659	0.14663909	0.32044757	0.51915181
Vta1	1734.00939	-1.0383882	-0.0543459	0.06466856	0.37751213	0.57950034
Vti1a	935.289383	1.01424925	0.02041223	0.07033332	0.75935065	0.86662603
Vti1b	1835.41361	1.11821704	0.16120023	0.07127839	0.01627276	0.05806761
Vwa2	1.79450239	-1.0084191	-0.0120953	0.21460995	0.6079291	0.76830819
Vwa3a	27.6106482	-1.0464361	-0.0654843	0.20864921	0.42734208	0.62541437
Vwa8	3452.28606	1.11363974	0.1552826	0.06191896	0.00863371	0.03467017
Vwf	7.57925304	-1.0466894	-0.0658334	0.22433514	0.1805619	0.35633924
WDR12	3093.46212	-1.0981749	-0.1351078	0.06350001	0.02547679	0.0827619
Wac	6148.87026	-1.0188026	-0.0268746	0.06013618	0.64098173	0.79087655
Wapl	7085.65292	-1.0300327	-0.0426902	0.05211139	0.39811642	0.59826851
Wars	2468.93123	1.10122922	0.13911479	0.05538157	0.00912163	0.03625443
Wars2	248.688204	1.00507957	0.00730972	0.12756903	0.94513031	0.97295716
Was	1.94630085	1.02204805	0.03146303	0.21782768	0.14363531	0.30232801
Wasf1	578.835324	1.04527684	0.06388509	0.0991528	0.46864134	0.6610103
Wasf2	7344.6767	-1.0120482	-0.017278	0.0458237	0.67226175	0.81086907
Washc1	919.053047	1.05396275	0.07582388	0.07266972	0.26679423	0.46101539
Washc2	3326.06882	1.14276766	0.19253212	0.05832848	0.00058646	0.00372275
Washc3	341.059754	1.01846396	0.02639493	0.12379109	0.79530604	0.88617607
Washc4	1482.19932	1.14798065	0.19909832	0.10179541	0.02716413	0.08674152
Washc5	6360.35456	1.04787649	0.06746868	0.05254146	0.18525063	0.36229932
Wasl	1312.59244	-1.0510151	-0.0717834	0.07376192	0.2994001	0.49718892

Wbp1	61.0554234	-1.077301	-0.1074214	0.20861323	0.30099559	0.4989329
Wbp2	2471.27147	1.290554	0.36799051	0.05805535	6.09E-11	1.57E-09
Wbp4	2112.62699	1.01805274	0.0258123	0.06653599	0.68686662	0.8200478
Wdcp	822.713712	-1.0150175	-0.0215046	0.08117569	0.77419372	0.87402317
Wdfy1	1481.89896	1.17178561	0.22870863	0.06639564	0.0002893	0.00202618
Wdfy2	356.87311	-1.0519783	-0.073105	0.09746724	0.39909755	0.59920764
Wdfy3	3294.9488	1.06452233	0.09020621	0.0679632	0.1632101	0.330758
Wdfy4	81.2586183	-1.4964776	-0.5815707	0.23639139	0.00122364	0.00686288
Wdhd1	2150.93849	-1.0060851	-0.0087524	0.06417605	0.88583419	0.93872913
Wdpcp	175.790023	1.28738651	0.36444526	0.16377591	0.0053172	0.02326274
Wdr1	4298.00267	1.01753682	0.02508101	0.05201931	0.61977742	0.77673241
Wdr11	2331.43115	1.27660169	0.35230846	0.05928207	7.91E-10	1.70E-08
Wdr13	933.726238	1.14189896	0.191435	0.07163712	0.00450509	0.02036354
Wdr17	6.66835286	-1.0056974	-0.0081963	0.20805283	0.87883319	0.93511531
Wdr18	742.396328	1.00625045	0.00898943	0.07907501	0.90337196	0.94988097
Wdr19	1080.28928	1.2043813	0.26829221	0.07559157	0.00015813	0.00121588
Wdr24	554.511365	1.25797572	0.33110408	0.12347214	0.00193214	0.01013128
Wdr25	185.634279	1.00321321	0.00462825	0.15315342	0.96692831	0.98398091
Wdr26	3857.9225	-1.1863645	-0.2465474	0.06497521	6.81E-05	0.00057934
Wdr27	13.4306727	-1.0536799	-0.0754366	0.21925804	0.29984852	0.49767745
Wdr3	5587.6285	-1.0577288	-0.0809697	0.04597466	0.07107325	0.18108552
Wdr31	77.5106391	-1.0425844	-0.0601642	0.18102786	0.56510994	0.73649999
Wdr33	1462.31813	-1.0212955	-0.0304004	0.07290039	0.65693672	0.80127899
Wdr34	378.952083	1.06780597	0.09464953	0.10284128	0.29536925	0.49287733
Wdr35	2356.5616	1.11043636	0.15112671	0.05858189	0.00719878	0.02975682
Wdr36	1832.34855	-1.0910119	-0.1256668	0.05816241	0.02441659	0.08031085
Wdr37	844.326905	1.00122631	0.00176811	0.09139056	0.98382803	0.99141261
Wdr4	231.464293	1.05593079	0.07851528	0.12261357	0.43832409	0.63600036
Wdr41	293.647673	1.0000316	4.56E-05	0.11555484	0.99946183	0.99979674
Wdr43	5866.68937	-1.1454205	-0.1958773	0.05013001	5.61E-05	0.00048554
Wdr44	750.802326	1.05171989	0.07275051	0.08121627	0.33116731	0.53110356
Wdr45	85.0913369	-1.057531	-0.0807	0.17479685	0.46319019	0.65677783
Wdr45b	3035.0485	1.14821135	0.19938822	0.06430789	0.00112835	0.0064375
Wdr46	2194.68024	1.11835376	0.16137661	0.05826994	0.00389735	0.01810845
Wdr47	1076.80402	1.17319395	0.23044154	0.07368484	0.00086721	0.00519351
Wdr48	3201.29278	-1.0269073	-0.0383059	0.04904717	0.42230821	0.62078474
Wdr5	2707.5841	-1.1517401	-0.2038152	0.05956505	0.00035578	0.00241648
Wdr53	400.603307	-1.0306089	-0.0434969	0.10906858	0.64257272	0.7918573
Wdr54	9.93383105	-1.1209109	-0.1646716	0.31163859	0.00789864	0.03210076
Wdr55	678.342442	-1.2663256	-0.3406484	0.08809769	3.03E-05	0.00027791
Wdr59	1027.15749	-1.0618439	-0.0865717	0.09457645	0.30685187	0.50524068
Wdr5b	277.114926	1.01044303	0.01498798	0.1214175	0.88257339	0.93698481
Wdr6	1846.01047	1.05305215	0.07457688	0.06174438	0.2069463	0.39146221
Wdr60	454.194004	1.16083263	0.21515998	0.10242226	0.01773972	0.06248166
Wdr61	3488.87476	-1.0398563	-0.0563842	0.0531525	0.27224936	0.46805989
Wdr62	474.524327	-1.0931673	-0.1285142	0.1202751	0.20055022	0.38275451
Wdr64	1.70510854	1.02002217	0.02860051	0.21666764	0.29138702	0.48818569
Wdr7	969.8214	1.30642544	0.38562479	0.08461029	1.20E-06	1.52E-05
Wdr70	1035.58254	-1.0065813	-0.0094636	0.09121276	0.9086329	0.95247976
Wdr73	737.323999	1.225873	0.29380952	0.11374468	0.00319284	0.01537603

Wdr74	749.281263	-1.1607039	-0.215	0.11823713	0.03256692	0.10018785
Wdr75	4560.56346	-1.1641476	-0.219274	0.05900587	0.00010704	0.000864
Wdr76	1683.84816	-1.1369198	-0.1851305	0.06281969	0.00199359	0.01039393
Wdr77	4557.02124	-1.192363	-0.2538235	0.07734782	0.00044552	0.00294142
Wdr78	123.461848	-1.0069987	-0.0100618	0.15086305	0.92449227	0.96122057
Wdr81	4164.73654	-1.0399723	-0.0565451	0.07115144	0.39860668	0.59861096
Wdr82	1575.07312	-1.0548561	-0.0770463	0.06509827	0.21320602	0.39915332
Wdr83	365.888272	-1.0147205	-0.0210824	0.11649161	0.82872557	0.90788335
Wdr83os	969.417193	1.04287624	0.06056797	0.09033895	0.45949315	0.65351044
Wdr89	174.71991	1.02066288	0.02950643	0.14181937	0.78410795	0.87922895
Wdr90	630.103651	1.1442387	0.19438804	0.11587644	0.04947359	0.13807336
Wdr91	571.306801	1.06330862	0.08856039	0.09691023	0.30596843	0.50411004
Wdr93	12.9763688	-1.0367264	-0.0520351	0.2104872	0.45976384	0.65381603
Wdr95	2.70421085	-1.0086457	-0.0124195	0.21591542	0.14807559	0.30867285
Wdtc1	1551.26925	1.1083846	0.14845857	0.08487718	0.05629315	0.15219822
Wdyhv1	222.949407	-1.1718339	-0.2287681	0.18075319	0.07276351	0.18428243
Wee1	1583.12393	1.00496245	0.0071416	0.07209333	0.9169631	0.95692723
Wfikkn1	46.7324992	-1.033136	-0.0470301	0.18628263	0.6338985	0.7864639
Wfs1	983.41311	1.25289376	0.32526409	0.08480284	3.72E-05	0.00033494
Whamm	241.294069	-1.0868952	-0.1202129	0.12768998	0.24739714	0.43821312
Whrn	923.82566	1.21517002	0.28115818	0.07304783	4.50E-05	0.00039918
Wipf2	623.032142	1.04444909	0.06274217	0.07912498	0.39383099	0.59478601
Wipi1	865.403119	1.27059292	0.34550188	0.09447199	6.82E-05	0.00058034
Wipi2	5101.04453	-1.2881852	-0.36534	0.06190072	9.15E-10	1.95E-08
Wiz	2723.33679	1.19295521	0.25453988	0.07456415	0.0002793	0.00197015
Wls	3165.27003	1.04549777	0.06418998	0.06731186	0.31449923	0.51364837
Wnk1	24124.8993	1.01143363	0.01640165	0.0453727	0.68683604	0.8200478
Wnk2	6.1497604	-1.1407898	-0.190033	0.36939114	0.00036072	0.00244228
Wnk4	355.442029	1.45385972	0.53988807	0.11435973	3.07E-07	4.33E-06
Wnt11	3.91717742	-1.0315724	-0.0448451	0.22007093	0.16593484	0.33495514
Wnt4	67.0092705	2.33288474	1.22211503	0.30707025	3.72E-06	4.23E-05
Wnt5b	25.5751849	-1.362023	-0.4457511	0.59060927	0.01832602	0.06392704
Wnt9a	3.26080649	1.02977445	0.04232838	0.21955323	0.16852803	0.33896122
Wrap53	238.715731	-1.1146807	-0.1566305	0.14993076	0.16811193	0.33828367
Wrap73	365.107766	1.02343358	0.03341747	0.10723957	0.72025704	0.8424482
Wrn	1585.81038	1.07500068	0.10433757	0.05749373	0.05989737	0.15977832
Wrnip1	1289.41628	-1.1044944	-0.1433861	0.07629366	0.04357416	0.12578613
Wsb2	1950.95613	-1.0412787	-0.0583562	0.07477518	0.40344215	0.60325598
Wt1	9.79902179	1.04119259	0.05823694	0.21543846	0.36896273	0.57027179
Wtap	1456.54353	-1.2105185	-0.2756251	0.09230576	0.00105714	0.00610821
Wtip	272.069808	1.1095513	0.14997638	0.14668722	0.18247271	0.35850849
Wwc2	12716.5344	1.40416915	0.48971674	0.03900385	6.40E-37	8.54E-35
Wwox	299.7348	1.15300414	0.20539769	0.12153758	0.04515847	0.12911413
Wwp1	933.400977	-1.0734983	-0.10232	0.08178104	0.17469763	0.34798268
Wwp2	1044.58814	1.25107821	0.32317198	0.07714255	8.65E-06	9.09E-05
Wwtr1	2037.21283	-1.2228579	-0.2902567	0.05858423	2.50E-07	3.59E-06
Xab2	1537.62464	1.04905624	0.06909202	0.07598476	0.33051274	0.53062642
Xbp1	1887.42245	1.13582706	0.18374319	0.10268575	0.04272682	0.1238379
Xdh	283.567416	-1.6001228	-0.6781826	0.1394078	1.03E-07	1.59E-06
Xiap	2728.68967	1.05164136	0.07264279	0.06121007	0.21548263	0.40173665

Xkr6	63.2485045	-1.0316158	-0.0449057	0.17452522	0.66806916	0.80811765
Xkr8	252.477158	1.03246874	0.0460981	0.1151828	0.63678423	0.78844056
Xkr9	11.4414548	1.02823445	0.04016925	0.21311533	0.46667326	0.65954153
Xkrx	3.81412591	1.06552877	0.09156955	0.24380014	0.00605037	0.02574925
Xpa	238.121846	-1.3050327	-0.3840859	0.13950162	0.00119197	0.00672356
Xpc	1223.97379	1.10306414	0.14151668	0.06761628	0.0270599	0.08649645
Xpnpep1	2817.80284	1.02888373	0.04107996	0.05339707	0.42726689	0.62541437
Xpnpep2	21.9482145	-1.3971013	-0.4824366	0.75150426	0.01474566	0.05389462
Xpnpep3	809.99885	-1.0111699	-0.0160254	0.09357703	0.84838501	0.91851015
Xpo1	18246.1333	-1.0276773	-0.0393873	0.04816456	0.40089986	0.60080425
Xpo4	3389.12685	-1.0149	-0.0213376	0.05474063	0.68634302	0.8196191
Xpo5	3990.21799	-1.1017656	-0.1398173	0.04553555	0.00163374	0.00878231
Xpo6	5473.1951	1.06762226	0.0944013	0.05756542	0.08814163	0.21311709
Xpo7	3627.51757	1.13465915	0.18225897	0.05145383	0.00025463	0.00182527
Xpot	10855.018	1.22173458	0.28893089	0.05407581	3.31E-08	5.56E-07
Xpr1	1115.6291	-1.0606681	-0.0849733	0.06939817	0.19486303	0.37480411
Xrcc1	938.441614	1.0157795	0.02258726	0.08638455	0.77599082	0.87520555
Xrcc2	508.320277	-1.7251066	-0.7866855	0.1211381	7.15E-12	2.06E-10
Xrcc3	26.596929	-1.0946368	-0.1304523	0.23593418	0.18419269	0.36075211
Xrcc4	499.894611	1.05041821	0.07096383	0.09807002	0.41674333	0.61567087
Xrcc5	1902.84473	1.15219375	0.20438333	0.06489168	0.00093536	0.00551654
Xrcc6	2512.32482	1.06303193	0.08818494	0.05569694	0.10616814	0.24373009
Xrn1	2794.64047	1.06044686	0.08467232	0.05846347	0.13204046	0.28413208
Xrn2	6270.45232	-1.0380884	-0.0539292	0.0572661	0.32500845	0.52461672
Xrra1	21.4775232	1.12762377	0.17328579	0.29569412	0.05790663	0.15564596
Xxylt1	328.731638	-1.2963712	-0.3744789	0.14013844	0.00154298	0.00834694
Xylb	1136.41841	1.02951832	0.0419695	0.07834396	0.56579019	0.73678686
Xylt2	806.516437	1.08846187	0.12229086	0.08473781	0.11531782	0.2583019
YPEL	65.4475929	1.06405725	0.08957578	0.19004169	0.40736735	0.60664679
Y_RNA	2834.99944	-1.0850832	-0.1178056	0.25626805	0.04816127	0.13529516
Yae1d1	513.842261	-1.2728679	-0.3480827	0.12592596	0.00136674	0.00753029
Yaf2	663.108789	1.01244271	0.01784027	0.11786713	0.85756687	0.92418115
Yap1	4641.95528	-1.1098531	-0.1503688	0.05036764	0.00206646	0.01069983
Yars	2683.09342	1.16679997	0.22255725	0.07260819	0.00112578	0.00643007
Yars2	564.147084	-1.1157099	-0.157962	0.09987045	0.07404547	0.1869378
Ybey	144.392612	-1.0746471	-0.103863	0.14517803	0.34500552	0.54434299
Ybx1	49455.4544	-1.0415386	-0.0587163	0.05205512	0.24293935	0.43289095
Ybx3	13698.5239	-1.1109912	-0.1518473	0.07228431	0.02480024	0.08123015
Yeats2	4381.12626	1.0849377	0.1176122	0.05522808	0.02740127	0.08730956
Yes1	1451.04702	1.00153148	0.00220777	0.06551177	0.97238873	0.98767978
Yif1a	848.104488	1.11819239	0.16116844	0.1260647	0.12127418	0.26766406
Yif1b	290.822426	1.04553757	0.0642449	0.12932932	0.53667166	0.71552497
Yipf1	353.126591	1.26512513	0.33928008	0.1494709	0.00546069	0.02374456
Yipf2	991.392076	1.13824359	0.18680934	0.08185229	0.01342112	0.04977548
Yipf3	520.536419	1.0225962	0.03223657	0.10068518	0.720173	0.84242682
Yipf4	621.549851	1.02926624	0.04161622	0.10514002	0.6485134	0.79587333
Yipf5	1068.32683	1.01662555	0.0237884	0.07672629	0.7408381	0.8558925
Yipf6	1129.83802	-1.0291958	-0.0415174	0.07523799	0.55521651	0.73032326
Yju2	380.004841	-1.0540969	-0.0760075	0.10311485	0.39972426	0.59986735
Ykt6	1002.21218	1.1165258	0.15901659	0.09306731	0.05790411	0.15564596

Ylpm1	5672.79516	-1.0053465	-0.0076929	0.05692923	0.88693741	0.93927995
Yme1l1	5643.91044	1.11396074	0.15569839	0.05432098	0.0028335	0.01395507
Yod1	702.943307	-1.3188178	-0.3992453	0.10078148	1.52E-05	0.00015102
Ypel5	30.8040807	1.04671862	0.06587367	0.2026759	0.4662313	0.65925935
Ythdc1	3859.59784	-1.1066586	-0.1462103	0.05530931	0.00608363	0.02587361
Ythdc2	913.281301	1.08258676	0.11448265	0.0947735	0.17817197	0.35270946
Ythdf1	3663.61957	1.14381137	0.19384916	0.05725993	0.00042818	0.002839
Ythdf2	2597.36653	-1.1213628	-0.1652532	0.06384126	0.00641369	0.02700292
Ythdf3	2159.14614	-1.0691717	-0.0964936	0.05485304	0.06829317	0.17630703
Ywhab	6727.96967	1.31563072	0.3957546	0.06428019	1.69E-10	4.07E-09
Ywhag	4142.42753	-1.5530107	-0.6350678	0.06079418	1.62E-26	1.40E-24
Ywhah	8054.59231	-1.0470655	-0.0663516	0.05037185	0.17370702	0.34660387
Ywhaz	4784.27089	-1.0821432	-0.1138915	0.07816955	0.11654693	0.26040874
Yy1	1402.14614	-1.0720268	-0.100341	0.06904187	0.12381472	0.27208408
Zadh2	397.683031	-1.0318295	-0.0452047	0.11870689	0.64658088	0.79457992
Zan	9.52274659	1.02559117	0.03645574	0.21104283	0.53762539	0.7163492
Zbed3	74.9030088	-1.0485802	-0.0684372	0.1619267	0.533774	0.71299738
Zbed4	519.65151	-1.1685555	-0.2247262	0.11637836	0.02415916	0.07974203
Zbed5	210.799379	1.28913877	0.36640758	0.18016784	0.00803223	0.03258073
Zbtb1	2215.09094	1.18584715	0.24591807	0.06802359	0.00013949	0.00108891
Zbtb10	436.870298	1.01285701	0.01843052	0.10622994	0.85014554	0.91935375
Zbtb11	1425.5196	-1.0626553	-0.0876737	0.08383966	0.25453901	0.44617471
Zbtb12	206.210003	1.15889718	0.21275257	0.14743742	0.06492351	0.17023359
Zbtb14	1112.01345	1.03757828	0.05322018	0.08231471	0.4844282	0.67336397
Zbtb17	765.177003	-1.1672417	-0.2231033	0.08645868	0.00484675	0.02156954
Zbtb18	692.724678	-1.1134551	-0.1550434	0.09068868	0.05838171	0.15655637
Zbtb2	982.583626	-1.0639257	-0.0893975	0.08684726	0.25962842	0.45194185
Zbtb20	409.236928	1.01339255	0.01919312	0.09632001	0.82449851	0.9052735
Zbtb21	1614.66947	-1.0651465	-0.0910518	0.07013593	0.16600653	0.33499428
Zbtb22	74.5613514	1.02686687	0.03824916	0.18691114	0.69487956	0.82550377
Zbtb24	341.664541	1.15252614	0.20479947	0.15538313	0.08356996	0.20444898
Zbtb25	1797.96647	1.13844967	0.18707051	0.0853263	0.01683211	0.05967875
Zbtb26	363.660741	-1.0137181	-0.0196565	0.1210035	0.8426202	0.91543734
Zbtb3	101.108278	1.04347369	0.06139422	0.16509405	0.57745603	0.74410617
Zbtb32	244.058664	-1.3645779	-0.4484548	0.2207782	0.00511751	0.02252758
Zbtb33	578.486504	-1.0562891	-0.0790048	0.08883249	0.32780092	0.52772776
Zbtb34	658.496335	1.00274927	0.00396091	0.09707782	0.96415221	0.9827954
Zbtb37	403.507644	1.19870085	0.26147166	0.12514324	0.01362239	0.0504052
Zbtb38	1389.07062	1.03435901	0.04873701	0.07486318	0.48768522	0.67603589
Zbtb39	255.172602	1.18742575	0.2478373	0.13919233	0.02840802	0.08956603
Zbtb4	1426.34233	1.80735719	0.85388165	0.07159049	7.56E-34	9.14E-32
Zbtb40	1603.20097	-1.0804313	-0.1116073	0.07909369	0.12762434	0.27766379
Zbtb41	1640.57871	1.1513676	0.20334852	0.08296554	0.00789445	0.03210076
Zbtb43	3151.30955	1.00105833	0.00152604	0.06452871	0.98034776	0.99079336
Zbtb44	1189.1636	-1.1129263	-0.154358	0.07791481	0.03295121	0.10116872
Zbtb45	129.683567	1.11021583	0.15084017	0.17662115	0.2055893	0.38975861
Zbtb48	425.838242	1.02939863	0.04180176	0.1132748	0.66542315	0.80643997
Zbtb49	247.049532	-1.0512419	-0.0720946	0.14664935	0.50717152	0.69152391
Zbtb5	124.51977	1.08007761	0.11113499	0.16582236	0.33195305	0.53173923
Zbtb7a	2028.24229	-1.0378326	-0.0535738	0.05963296	0.34918281	0.54883826

Zbtb7b	912.819826	1.21814231	0.28468269	0.09294738	0.00077937	0.00474058
Zbtb8a	163.960199	1.15942988	0.21341557	0.1637529	0.07953445	0.19729595
Zc2hc1a	452.878642	1.16484136	0.22013349	0.09927053	0.01300854	0.04852868
Zc2hc1c	155.679378	-1.0978059	-0.134623	0.16142993	0.24406424	0.43430597
Zc3h10	235.036517	1.13235771	0.17932977	0.13974505	0.10511884	0.24179788
Zc3h12a	248.194936	-1.2370071	-0.3068538	0.15115956	0.01128848	0.04337294
Zc3h12b	2.91915831	-1.0147812	-0.0211687	0.21463285	0.50048534	0.68583839
Zc3h12c	1132.47118	-1.0693582	-0.0967452	0.08199895	0.19999932	0.38177605
Zc3h12d	3.36583327	1.02667322	0.03797706	0.21818765	0.24030577	0.42993254
Zc3h13	3847.93916	-1.0677383	-0.0945582	0.07152923	0.15981412	0.32585903
Zc3h14	4316.63091	1.20674312	0.27111861	0.05393603	2.06E-07	3.01E-06
Zc3h18	4152.12906	1.00293192	0.00422368	0.05612975	0.93865327	0.9703154
Zc3h3	1017.95936	-1.2041952	-0.2680692	0.09090368	0.00123986	0.00693727
Zc3h4	2424.66355	-1.0562891	-0.0790048	0.07113709	0.23880092	0.4286771
Zc3h6	124.424736	-1.1284735	-0.1743725	0.19619697	0.15942048	0.32567047
Zc3h7a	3133.65015	-1.1184199	-0.161462	0.05356719	0.00180268	0.00957393
Zc3h7b	4073.6719	-1.0097694	-0.0140259	0.05195786	0.78343938	0.87895064
Zc3h8	462.616797	-1.2515627	-0.3237306	0.12940637	0.00327509	0.01571396
Zc3hav1	7423.41776	1.08540749	0.11823677	0.05562609	0.02751104	0.08751338
Zc3hc1	662.11832	-1.0565496	-0.0793605	0.08971426	0.32901673	0.52888748
Zc4h2	756.108334	1.74863486	0.80622907	0.09664621	6.44E-18	3.31E-16
Zcchc10	258.215109	-1.2285387	-0.2969434	0.14845759	0.01316438	0.04896506
Zcchc14	968.805383	1.04123794	0.05829978	0.07545908	0.40912211	0.60826999
Zcchc17	1544.6661	-1.1871114	-0.2474553	0.08338701	0.00133048	0.00735581
Zcchc2	898.557867	-1.0875993	-0.1211472	0.09478881	0.15353927	0.31641539
Zcchc24	1648.59447	1.12305988	0.16743485	0.07604962	0.01833473	0.06392704
Zcchc4	359.551173	-1.0020819	-0.0030005	0.10475949	0.97296553	0.98777052
Zcchc7	277.344539	-1.0730236	-0.1016818	0.12324062	0.31728638	0.51625029
Zcchc8	1131.06309	-1.0290682	-0.0413385	0.07287085	0.54574845	0.72318588
Zcchc9	1285.34192	1.00764294	0.01098451	0.09251453	0.89632772	0.9455619
Zcrb1	1017.28324	-1.045578	-0.0643007	0.08463791	0.4076573	0.60672593
Zcwpw1	54.5608044	-1.0223488	-0.0318875	0.1913049	0.71844784	0.84156113
Zdbf2	1.75630036	-1.0287622	-0.0409095	0.22043992	0.06180916	0.16384437
Zdhhc1	77.1638139	1.18023729	0.23907694	0.21954417	0.07819595	0.19483475
Zdhhc13	653.095068	1.1016285	0.13963779	0.11617066	0.15599574	0.32049806
Zdhhc14	15.6747662	1.00921523	0.01323389	0.19920129	0.86482196	0.92729299
Zdhhc15	983.3353	1.13757585	0.18596274	0.09701072	0.03213514	0.09909753
Zdhhc16	359.247976	-1.0849733	-0.1176596	0.10215212	0.18938394	0.36768843
Zdhhc17	1059.8419	-1.003734	-0.005377	0.08356913	0.94361474	0.97250604
Zdhhc18	358.710028	-1.1131415	-0.1546369	0.11514552	0.11389374	0.25587142
Zdhhc19	49.0385679	1.25587473	0.32869257	0.3050596	0.03773113	0.11248876
Zdhhc20	2658.09287	-1.0228212	-0.032554	0.05955002	0.56962021	0.73906967
Zdhhc21	375.186172	-1.0909993	-0.1256502	0.1066038	0.17519514	0.34881096
Zdhhc24	43.7668806	-1.0247121	-0.0352186	0.18517635	0.716562	0.84081334
Zdhhc3	3479.39234	-1.0212522	-0.0303391	0.05750862	0.58161096	0.74773549
Zdhhc4	255.953352	1.04097287	0.05793247	0.14848358	0.59531457	0.75850205
Zdhhc5	2153.46387	-1.0431419	-0.0609355	0.07143968	0.36516248	0.56617422
Zdhhc6	2719.46043	1.05477588	0.07693648	0.06042712	0.18409667	0.36070333
Zdhhc7	876.067576	-1.0576651	-0.0808829	0.09396269	0.33767482	0.53733566
Zdhhc8	1193.96002	-1.1047265	-0.1436892	0.09205009	0.08333206	0.20396444

Zdhhc9	2.4924778	-1.0363511	-0.0515129	0.22382268	0.02470456	0.08102034
Zeb1	3258.44264	-1.0027838	-0.0040106	0.05410224	0.93845441	0.97018812
Zeb2	3524.5445	-1.0905812	-0.1250972	0.05838854	0.02651295	0.08529155
Zer1	2345.84635	1.1818	0.24098591	0.07516863	0.00062367	0.00391863
Zfand1	443.143708	-1.2190059	-0.2857051	0.11778101	0.00510417	0.02250756
Zfand2a	420.029565	-1.2716669	-0.3467208	0.12677822	0.00150543	0.00818527
Zfand2b	254.506224	-1.0188908	-0.0269995	0.1328236	0.79532344	0.88617607
Zfand3	646.020415	1.13491137	0.18257964	0.09114673	0.02706306	0.08649645
Zfand4	189.020227	1.12024175	0.1638101	0.14105108	0.13998155	0.29653772
Zfand5	832.048293	-1.1234621	-0.1679515	0.11431318	0.08513939	0.20750296
Zfand6	1152.84247	-1.0737165	-0.1026131	0.07212822	0.12994589	0.28118359
Zfat	489.982804	-1.0063867	-0.0091847	0.09203609	0.91123793	0.95410634
Zfc3h1	3385.3447	1.03216863	0.04567869	0.06386446	0.45371778	0.64845752
Zfhx2	1.95530156	-1.0101368	-0.0145506	0.21436758	0.59963405	0.76241312
Zfhx3	2483.88776	-1.173144	-0.2303801	0.06114996	8.33E-05	0.00069266
Zfhx4	707.214665	-1.9460986	-0.9605848	0.10068142	1.15E-22	8.00E-21
Zfp1	373.436261	1.10848822	0.14859344	0.1292167	0.15950251	0.32567047
Zfp101	433.805629	-1.0136475	-0.0195561	0.09771971	0.82171386	0.90376956
Zfp105	741.115718	-1.0121515	-0.0174252	0.07583455	0.80535332	0.89269198
Zfp106	22097.5316	1.33841962	0.4205305	0.04154928	7.56E-25	6.09E-23
Zfp11	422.352699	1.10204734	0.1401862	0.10146859	0.11731537	0.26158572
Zfp112	556.804078	1.08616957	0.11924935	0.10369092	0.18997554	0.36865448
Zfp113	278.625467	1.03687442	0.05224117	0.12354651	0.60758025	0.76801885
Zfp12	561.614522	1.08682455	0.12011905	0.10642168	0.1943884	0.37422835
Zfp128	136.614614	-1.1310005	-0.1775995	0.16193116	0.13397225	0.28746809
Zfp13	416.009098	-1.0548153	-0.0769905	0.10739791	0.40670353	0.60628124
Zfp131	3659.83668	-1.0515951	-0.0725794	0.06363887	0.23188741	0.42116513
Zfp14	108.832761	1.18887494	0.24959696	0.19148125	0.059644	0.15930419
Zfp143	941.25056	-1.1414321	-0.1908451	0.08642547	0.01577096	0.05673294
Zfp146	477.767681	1.01514731	0.02168909	0.12712365	0.82902653	0.90788335
Zfp148	1484.20062	1.03965951	0.05611112	0.06874914	0.39015066	0.59162937
Zfp157	275.406797	1.15279499	0.20513597	0.16415598	0.08985788	0.21591291
Zfp169	290.356171	-1.0403347	-0.0570478	0.11753964	0.56181029	0.73459119
Zfp180	746.301218	1.03790239	0.05367077	0.08305427	0.48153696	0.67165597
Zfp182	137.276685	1.13910428	0.18789982	0.18105065	0.13009744	0.28141645
Zfp184	488.871667	-1.013146	-0.0188421	0.10455779	0.83570618	0.91187361
Zfp185	3.24796979	-1.0012191	-0.0017577	0.2136357	0.94611366	0.97323665
Zfp189	344.260766	-1.1023746	-0.1406146	0.1050346	0.12540848	0.27419057
Zfp202	129.727774	-1.1136519	-0.1552983	0.15766267	0.18053806	0.35633924
Zfp207	3622.6416	-1.0055029	-0.0079173	0.05006115	0.86557096	0.92774752
Zfp212	561.218801	-2.269705	-1.1825048	0.10674804	8.89E-30	9.41E-28
Zfp213	146.262743	-1.0605891	-0.0848659	0.16757006	0.44617323	0.64259525
Zfp217	2070.84548	-1.0362593	-0.051385	0.06775842	0.42322188	0.6215859
Zfp219	375.030172	-1.0582886	-0.0817331	0.11514065	0.40045126	0.60048721
Zfp236	1069.15537	1.10611693	0.1455039	0.06839573	0.02403971	0.07938869
Zfp239	104.104167	-1.2050659	-0.269112	0.18721381	0.04237654	0.12309375
Zfp24	635.072724	-1.2479324	-0.3195398	0.10691998	0.00081243	0.0049114
Zfp248	202.461752	1.04032558	0.05703511	0.12275845	0.57314885	0.74163302
Zfp251	372.076747	-1.0033458	-0.004819	0.12402799	0.96129531	0.98152343
Zfp26	1497.04547	-1.0100316	-0.0144004	0.08161577	0.84780465	0.9181146

Zfp260	434.202403	1.08798389	0.1216572	0.11395907	0.20949963	0.39483452
Zfp263	797.834022	1.11485059	0.15685038	0.10555813	0.08889582	0.2144064
Zfp266	1433.27118	-1.0186331	-0.0266346	0.09089876	0.74877245	0.86055761
Zfp267	830.308036	1.08549306	0.1183505	0.09270801	0.1562543	0.32092634
Zfp276	306.548852	1.18092078	0.23991219	0.12367455	0.0216759	0.07316689
Zfp277	896.242629	1.05239835	0.0736809	0.08572072	0.3462419	0.54575596
Zfp28	941.251913	1.26752868	0.34201839	0.08619481	2.03E-05	0.00019348
Zfp280b	484.477217	-1.1171595	-0.1598352	0.0986615	0.06836442	0.1764373
Zfp280c	5210.95197	1.02259348	0.03223274	0.06364244	0.60611165	0.76704121
Zfp280d	1348.41696	1.04878504	0.06871901	0.06875791	0.29169312	0.48863461
Zfp281	2076.71924	-1.1139252	-0.1556524	0.08411959	0.04367907	0.12596329
Zfp282	344.49971	-1.726548	-0.7878904	0.13498431	4.15E-10	9.27E-09
Zfp286	122.85249	1.12155408	0.16549919	0.18275542	0.1737278	0.34660387
Zfp292	1992.95232	-1.0545797	-0.0766681	0.07041217	0.24816475	0.43923403
Zfp296	88.0767195	1.11712081	0.15978521	0.20683987	0.18765069	0.36526445
Zfp300	230.722419	1.05756669	0.08074864	0.13103353	0.44305216	0.6401419
Zfp316	302.113575	-1.027655	-0.0393561	0.11411439	0.68116827	0.81646968
Zfp317	556.581321	-1.0448108	-0.0632417	0.09155045	0.44462232	0.64103679
Zfp318	3193.0856	1.41874853	0.5046189	0.0712446	2.22E-13	7.57E-12
Zfp319	106.178803	-1.0606309	-0.0849227	0.16488212	0.44487847	0.64126176
Zfp322a	210.820209	-1.1315733	-0.17833	0.16605058	0.13640332	0.29102708
Zfp324	45.6854502	1.12071165	0.16441513	0.21508084	0.17433867	0.34756268
Zfp326	1905.83766	-1.056887	-0.0798212	0.07208643	0.23857805	0.42839709
Zfp329	61.4514109	1.4081779	0.4938296	0.3306008	0.01089957	0.04217765
Zfp330	1822.74037	-1.0171801	-0.0245751	0.07417381	0.72332215	0.8439527
Zfp334	1535.55998	1.2655364	0.33974901	0.07105745	5.17E-07	7.01E-06
Zfp335	1472.24041	1.12768626	0.17336575	0.08054196	0.0199365	0.06841321
Zfp341	256.404357	1.23324216	0.30245612	0.1655336	0.01796981	0.06311852
Zfp346	265.392112	1.05794711	0.0812675	0.13310595	0.44008896	0.63744228
Zfp354a	229.738156	-1.0536865	-0.0754457	0.13600092	0.47789017	0.66831583
Zfp354c	226.966341	1.12163965	0.16560926	0.15061812	0.14896045	0.3098855
Zfp358	438.37877	1.57313775	0.653645	0.13243439	7.75E-08	1.22E-06
Zfp36	240.103235	-1.2228885	-0.2902929	0.14405554	0.01320794	0.04908436
Zfp362	659.375872	1.29222274	0.36985477	0.09414871	2.09E-05	0.00019848
Zfp365	400.128785	-1.2502033	-0.3221628	0.11231176	0.00116323	0.00659919
Zfp366	5.07326469	1.03612174	0.05119352	0.22057195	0.20667616	0.39119212
Zfp367	477.895389	1.03819357	0.05407546	0.09626987	0.5305003	0.71086309
Zfp36l1	2142.48155	-1.0504878	-0.0710594	0.06241858	0.23306252	0.42240108
Zfp36l2	714.201882	-1.0730838	-0.1017627	0.10434611	0.26488711	0.45869182
Zfp37	657.260162	-1.0462279	-0.0651971	0.08668677	0.40999142	0.60914632
Zfp382	160.82318	-1.0548181	-0.0769942	0.15827659	0.48960248	0.67718402
Zfp383	216.357409	1.21880704	0.28546974	0.15756536	0.02076095	0.0706932
Zfp384	869.98112	1.08549893	0.11835831	0.09226225	0.15532262	0.3194225
Zfp385a	181.530307	1.15397005	0.20660578	0.15874119	0.08429613	0.20583939
Zfp385b	10.614212	-7.573446	-2.9209499	0.9404515	9.37E-05	0.00076482
Zfp386	915.609411	-1.0881285	-0.1218489	0.08366916	0.11273707	0.25429861
Zfp39	210.743202	1.04347905	0.06140163	0.131538	0.55804679	0.73205144
Zfp395	539.09371	1.04857273	0.06842693	0.10723683	0.46132488	0.65509053
Zfp397	188.217276	1.03003581	0.0426945	0.13585886	0.68732725	0.8204885
Zfp398	190.409117	-2.0910325	-1.0642155	0.18430806	5.17E-10	1.14E-08

Zfp407	1983.53573	-1.0407008	-0.0575554	0.06408368	0.34571552	0.54512749
Zfp408	481.229756	-1.0083018	-0.0119275	0.10096153	0.89319487	0.94396719
Zfp41	96.0624452	1.0367329	0.05204424	0.17534327	0.62448802	0.78000113
Zfp410	989.752169	1.09095321	0.12558922	0.07198595	0.06341871	0.16705607
Zfp414	197.344618	1.60281529	0.68060818	0.18638582	2.28E-05	0.00021447
Zfp420	96.8617031	1.08790104	0.12154733	0.19485276	0.28640496	0.48293374
Zfp423	873.724266	-1.028475	-0.0405068	0.08273967	0.59489499	0.75826914
Zfp426	161.579215	-1.2319657	-0.300962	0.19166977	0.02807704	0.08885051
Zfp428	132.50002	1.16981169	0.22627632	0.16251888	0.06395089	0.16821578
Zfp438	415.106833	1.07017128	0.09784172	0.1039118	0.28218243	0.47814202
Zfp444	82.1332274	-1.0280163	-0.0398632	0.15878177	0.71268118	0.8376407
Zfp445	3808.02196	-1.156949	-0.2103252	0.06022213	0.00026548	0.00189038
Zfp446	9.63767226	1.04430779	0.06254698	0.21819008	0.32318821	0.52260047
Zfp449	1027.36195	1.17871078	0.23720976	0.07371129	0.00061792	0.00389354
Zfp451	1791.31422	1.09931056	0.13659901	0.06849237	0.03494191	0.10584613
Zfp46	402.907928	1.03884934	0.05498644	0.11254214	0.56721389	0.7378158
Zfp462	3330.60269	-1.2375853	-0.307528	0.06903313	2.79E-06	3.28E-05
Zfp469	1922.00891	-1.1674623	-0.223376	0.09224134	0.00750565	0.03078687
Zfp472	415.145732	1.09493963	0.13085133	0.11757695	0.18649543	0.36367887
Zfp473	712.474038	1.08028379	0.11141036	0.10220339	0.21703783	0.40331334
Zfp507	1802.29471	1.06812203	0.09507648	0.07954466	0.19736616	0.37806555
Zfp511	254.238625	-1.0553706	-0.0777497	0.11929752	0.43397611	0.63179158
Zfp512b	1159.39408	1.47540519	0.56111121	0.08951818	5.27E-11	1.37E-09
Zfp513	300.615204	1.02019413	0.0288437	0.12228937	0.77570608	0.87508221
Zfp516	1072.00455	1.15952466	0.21353351	0.09432723	0.01201759	0.04559994
Zfp518a	1177.70738	1.06849433	0.09557925	0.07990247	0.19672747	0.3773703
Zfp523	477.398071	-1.1449641	-0.1953024	0.12191199	0.05601061	0.15165841
Zfp526	112.198287	1.00193791	0.0027931	0.15518169	0.98058611	0.99086465
Zfp532	527.58545	1.11193035	0.15306642	0.09160042	0.06433231	0.16901086
Zfp551	133.752128	1.26288643	0.3367249	0.21565219	0.02234452	0.07504807
Zfp553	526.463745	1.4025884	0.4880917	0.10106271	2.06E-07	3.01E-06
Zfp558	134.733888	1.32327836	0.40411658	0.19792136	0.00640167	0.02697548
Zfp560	445.574512	1.01147235	0.01645688	0.10840649	0.86176586	0.92618801
Zfp563	98.7560319	-1.0179003	-0.0255963	0.15817231	0.8112778	0.8971399
Zfp566	522.144867	-1.138742	-0.1874409	0.10263287	0.03838524	0.1138668
Zfp568	712.541904	-1.229758	-0.2983745	0.09103505	0.00035464	0.00241131
Zfp574	267.028965	-1.0155243	-0.0222247	0.12527602	0.82631985	0.90665159
Zfp58	83.5738989	1.19185411	0.25320766	0.2426583	0.06997328	0.17916405
Zfp580	21.9811808	1.098969	0.13615069	0.25752507	0.10322421	0.23894148
Zfp583	20.0691891	1.06643094	0.09279055	0.22811277	0.2133671	0.39930352
Zfp592	1515.37247	1.02673975	0.03807055	0.06401842	0.5337147	0.71299738
Zfp593	282.341079	-1.1131378	-0.1546322	0.1777629	0.19729081	0.37799809
Zfp597	284.856896	-1.1024845	-0.1407583	0.14425665	0.20486033	0.3887218
Zfp598	2112.56414	1.20980784	0.27477792	0.06092261	2.59E-06	3.08E-05
Zfp605	602.327661	-1.2618652	-0.3355578	0.11734198	0.00109954	0.0063077
Zfp606	36.4069773	1.09912743	0.13635866	0.22773951	0.20443576	0.38823505
Zfp608	1143.73421	-1.1996626	-0.2626287	0.08119178	0.00050265	0.0032718
Zfp609	4271.45997	1.0503481	0.07086753	0.05937723	0.21315272	0.39913555
Zfp61	226.684214	1.07007091	0.0977064	0.12859569	0.34831626	0.54787957
Zfp622	1313.8552	-1.1308702	-0.1774333	0.06812232	0.00586921	0.02515377

Zfp623	373.600233	1.20501481	0.26905087	0.10997888	0.00532022	0.02326801
Zfp628	110.626041	1.0390841	0.05531243	0.15075336	0.613627	0.77269695
Zfp629	881.229154	1.28198586	0.35838035	0.07881439	1.40E-06	1.75E-05
Zfp637	258.822058	1.01403591	0.02010874	0.12615107	0.84480876	0.9164197
Zfp638	5705.94726	-1.1724463	-0.2295218	0.05527456	1.68E-05	0.00016468
Zfp64	1629.71472	1.128214	0.17404075	0.09196918	0.03602825	0.10833846
Zfp644	3549.1526	-1.0659753	-0.092174	0.04963315	0.05586396	0.15129331
Zfp646	2197.76211	1.04410701	0.06226959	0.06757711	0.33190014	0.53172095
Zfp647	49.1930254	-1.1418531	-0.191377	0.22949183	0.12942207	0.28014476
Zfp651	246.160396	1.30938533	0.38888972	0.14810594	0.0016794	0.00899754
Zfp652	979.14889	1.18173319	0.24090434	0.10745685	0.01071473	0.04159938
Zfp653	95.1134265	1.03320028	0.04711994	0.15762538	0.66620273	0.80661107
Zfp654	992.46153	1.02939434	0.04179576	0.06952444	0.5257599	0.70722065
Zfp655	3357.7172	-1.0344564	-0.0488728	0.0593249	0.39074437	0.59235922
Zfp664	1500.39751	-1.390717	-0.4758289	0.07463414	2.97E-11	8.00E-10
Zfp667	547.579352	-1.1157087	-0.1579605	0.14429351	0.15797289	0.32352242
Zfp668	226.264719	-1.0125258	-0.0179586	0.12585974	0.85853694	0.92418976
Zfp672	97.9569197	1.01449274	0.02075853	0.16586958	0.84691244	0.91769146
Zfp68	1106.49104	-1.0022347	-0.0032205	0.08124633	0.96507086	0.98295812
Zfp687	2685.78799	1.1412594	0.19062675	0.06279536	0.00145942	0.00795539
Zfp688	102.699625	-1.0455768	-0.0642991	0.1664388	0.55716134	0.73133339
Zfp689	250.014189	-1.0351406	-0.0498268	0.11848708	0.61508147	0.77376891
Zfp69	256.032367	1.30395329	0.38289219	0.13121954	0.00073071	0.00447649
Zfp692	200.242548	1.11453334	0.15643978	0.16638589	0.18614128	0.36337547
Zfp697	1017.87138	-1.2302003	-0.2988933	0.09364273	0.00046492	0.00305105
Zfp7	492.206645	1.2488922	0.32064895	0.10726978	0.00081612	0.00491974
Zfp703	748.176619	-1.1482064	-0.199382	0.08531156	0.01077441	0.04179898
Zfp706	1710.21837	-1.028877	-0.0410705	0.06860894	0.52720742	0.70851102
Zfp707	91.4610539	1.07370319	0.10259523	0.17819442	0.36492106	0.56594669
Zfp709	408.144511	1.13353009	0.18082269	0.12545564	0.08263785	0.20267871
Zfp712	285.130239	1.28120475	0.35750105	0.13263934	0.00161617	0.00870247
Zfp715	798.095477	1.06532309	0.09129104	0.08422041	0.23719794	0.42675641
Zfp72	106.352993	-1.0547895	-0.0769551	0.16607163	0.48620058	0.67499887
Zfp74	335.259933	1.29428661	0.37215713	0.12797436	0.00079982	0.00483972
Zfp740	1641.79511	1.05021161	0.07068005	0.0623649	0.23629394	0.42594252
Zfp746	296.504129	-1.79292	-0.8423111	0.13733945	6.60E-11	1.68E-09
Zfp748	511.59484	1.10505302	0.1441156	0.09454322	0.08925921	0.21496719
Zfp768	689.206337	1.47995068	0.5655491	0.1052642	1.01E-08	1.83E-07
Zfp770	886.730667	-1.0224017	-0.0319621	0.09282802	0.70180629	0.83028433
Zfp771	192.982278	-1.0438843	-0.0619618	0.15789749	0.56941383	0.73906967
Zfp772	53.0746771	1.08598684	0.11900662	0.20989759	0.27364403	0.46928911
Zfp775	62.1612834	-1.6840532	-0.7519377	0.29953981	0.00073889	0.00452229
Zfp777	787.699429	-1.430978	-0.5170015	0.09070615	1.76E-09	3.60E-08
Zfp784	35.4667472	1.03921301	0.05549139	0.20775264	0.4780278	0.66843533
Zfp786	45.4492837	-1.4318016	-0.5178316	0.38587697	0.01138029	0.04366023
Zfp787	156.992521	-1.0014348	-0.0020685	0.14704004	0.98323789	0.99140011
Zfp790	418.595506	1.05116406	0.07198785	0.09361503	0.39529275	0.59593975
Zfp799	107.937423	1.07730899	0.10743209	0.17350424	0.34662458	0.54629193
Zfp800	1394.08286	-1.1076378	-0.1474862	0.07381087	0.03273264	0.10061663
Zfp809	538.252147	-1.0662172	-0.0925013	0.09135633	0.26225881	0.45509826

Zfp81	132.298304	1.06563041	0.09170716	0.16704336	0.41598709	0.61489808
Zfp810	59.8065434	-1.0174169	-0.024911	0.17728129	0.80554698	0.89269198
Zfp82	107.556407	1.30143025	0.38009799	0.24021647	0.01656017	0.05882856
Zfp821	143.100722	1.0381589	0.05402729	0.13620437	0.61033401	0.77058729
Zfp827	485.307994	-1.0137107	-0.019646	0.10903299	0.8325502	0.91007181
Zfp830	1179.93995	-1.1759115	-0.2337795	0.08788371	0.0036637	0.01723538
Zfp84	291.490245	1.06553789	0.09158189	0.13204629	0.38700969	0.58829882
Zfp846	246.587505	1.00944579	0.01356343	0.12472373	0.89471817	0.94487549
Zfp853	1.97707667	-1.0075836	-0.0108996	0.2141196	0.6908319	0.82298489
Zfp865	78.8681	1.29314367	0.37088256	0.26506725	0.0225068	0.07543489
Zfp866	413.341385	1.10923674	0.14956731	0.11327735	0.12524612	0.27406949
Zfp868	238.271176	1.00595506	0.00856586	0.13579353	0.93645141	0.96922329
Zfp869	561.089712	1.1059185	0.14524508	0.10776594	0.12081408	0.26692452
Zfp87	251.257622	1.13973135	0.1886938	0.12642457	0.07175371	0.182374
Zfp870	81.4968063	-1.0109191	-0.0156676	0.17267829	0.87806949	0.93464187
Zfp871	665.567328	-1.0527468	-0.0741585	0.09404539	0.37940372	0.58156775
Zfp882	2527.08786	1.00574531	0.00826502	0.06543277	0.89490976	0.94499992
Zfp9	1308.17885	1.23407377	0.30342864	0.09129675	0.00029599	0.00206401
Zfp90	67.2404813	1.32024599	0.40080676	0.34653806	0.02353036	0.07804865
Zfp94	92.9901703	1.08708855	0.12046946	0.17570918	0.2999155	0.49767745
Zfp952	94.9978024	1.01433731	0.02053749	0.1667316	0.84869696	0.91853734
Zfp954	80.7031874	1.04942796	0.06960313	0.16928806	0.52698271	0.70828334
Zfp956	65.5436968	-1.1705924	-0.2272388	0.27246427	0.08463835	0.20651757
Zfp961	197.976805	1.25231838	0.32460139	0.16579979	0.01203386	0.0456211
Zfp963	76.9177452	1.00421823	0.00607282	0.15896338	0.95525776	0.97846346
Zfp964	88.8198218	1.05887773	0.082536	0.17548772	0.45471819	0.64930776
Zfpl1	302.777824	1.21780364	0.28428153	0.12286663	0.00683894	0.02847149
Zfpm1	395.288004	1.1131569	0.15465696	0.12871233	0.14179948	0.29954673
Zfr	10381.813	-1.1749206	-0.2325633	0.05488587	1.13E-05	0.00011662
Zfr2	26.7850823	1.09292788	0.1281982	0.23271139	0.19883299	0.37997035
Zfx	3076.13704	-1.0179393	-0.0256516	0.06506971	0.67859202	0.81499755
Zfyve1	208.009372	1.11670152	0.15924363	0.14767068	0.15965734	0.32567047
Zfyve16	2247.19576	1.19031907	0.25134835	0.06137018	1.90E-05	0.0001826
Zfyve19	555.013069	1.05258522	0.07393704	0.08844442	0.35861501	0.55879556
Zfyve21	658.445645	1.06721289	0.093848	0.09672864	0.2767148	0.4723077
Zfyve26	3206.33198	-1.0658715	-0.0920335	0.05142493	0.06478257	0.16998459
Zfyve27	746.983158	1.02084592	0.02976514	0.0937977	0.72467117	0.84468495
Zfyve9	2002.26454	1.08160059	0.11316784	0.06759287	0.07726489	0.19303545
Zglp1	28.8744406	-1.0820332	-0.1137448	0.22998485	0.21095462	0.3963521
Zgpat	518.568365	1.43285395	0.51889157	0.11783164	1.48E-06	1.85E-05
Zgrf1	661.389832	-1.0346236	-0.0491061	0.08650035	0.5340661	0.71326991
Zhx1	7061.43401	-1.0791149	-0.1098485	0.0597919	0.05493655	0.14925497
Zhx2	1257.97014	-1.4721891	-0.557963	0.1066684	2.12E-08	3.70E-07
Zhx3	1008.80147	1.5394747	0.62243816	0.08302469	7.26E-15	2.86E-13
Zkscan1	789.63062	1.00604208	0.00869064	0.07594795	0.90333677	0.94988097
Zkscan14	139.757752	-1.1476062	-0.1986277	0.1880861	0.11492881	0.25790472
Zkscan16	1140.02608	1.02101441	0.03000322	0.07760983	0.67912053	0.81532652
Zkscan17	153.95632	-1.0584048	-0.0818916	0.1486797	0.45470292	0.64930776
Zkscan3	444.411078	1.08261733	0.11452339	0.10021999	0.19663643	0.37725216
Zkscan5	1127.19236	-1.0291017	-0.0413856	0.08652661	0.6009023	0.76319239

Zkscan6	180.675279	-1.0427479	-0.0603904	0.14153913	0.57476713	0.74243894
Zkscan7	250.171656	1.28518512	0.36197619	0.13896089	0.00201432	0.0104807
Zkscan8	646.3717	1.1477949	0.19886487	0.11175158	0.04009548	0.11777645
Zmat1	102.492788	1.00132526	0.00191067	0.15295423	0.983803	0.99141261
Zmat2	718.24287	-1.017983	-0.0257135	0.0864236	0.7453401	0.85822982
Zmat3	263.332436	-1.0332308	-0.0471626	0.12382104	0.64149393	0.79121291
Zmat5	637.572122	1.06763771	0.09442217	0.10997543	0.3187199	0.51720318
Zmiz1	6492.43706	1.15712993	0.21055087	0.0601287	0.00026751	0.00190269
Zmiz2	717.265694	1.06278684	0.08785227	0.10151843	0.32673199	0.52661551
Zmpste24	2487.93915	-1.1201596	-0.1637043	0.05879958	0.00367141	0.01725943
Zmym2	2655.6027	1.15863788	0.21242974	0.06136395	0.00029649	0.00206617
Zmym3	1034.84697	1.27682499	0.35256079	0.08131125	3.85E-06	4.37E-05
Zmym4	2777.53132	1.07398535	0.10297431	0.08184438	0.17334237	0.3461443
Zmym5	1044.85248	1.16283797	0.21765009	0.0726606	0.00144077	0.00786904
Zmym6	488.232667	-1.0194167	-0.0277439	0.08596913	0.72395258	0.84436479
Zmynd10	12.4429234	1.0142944	0.02047645	0.2094594	0.7067868	0.83415689
Zmynd11	4029.93471	1.23784991	0.3078364	0.05476275	6.65E-09	1.25E-07
Zmynd12	141.680652	1.01321945	0.01894668	0.13163581	0.85796441	0.92418115
Zmynd15	68.0334209	-1.017666	-0.0252641	0.16274578	0.8123196	0.89751717
Zmynd19	1832.70823	1.06439298	0.09003091	0.08328447	0.240739	0.430347
Zmynd8	3151.37854	1.0462865	0.06527796	0.06030958	0.26009182	0.45219547
Znfx1	2029.97151	1.35551218	0.43883808	0.0612137	1.45E-13	5.06E-12
Znhit2	390.8642	-1.0893226	-0.1234313	0.12950755	0.24282732	0.4328507
Znhit6	1745.08932	-1.0295126	-0.0419614	0.07128932	0.53223513	0.71201878
Znrd1	267.486633	1.09043226	0.12490016	0.1436998	0.25940551	0.45173799
Znrd1as	120.103615	1.17365404	0.2310072	0.21317595	0.08456653	0.20638165
Znrf1	600.772749	-1.0103809	-0.0148993	0.09452448	0.85991059	0.9249689
Znrf2	75.7377892	-2.3339976	-1.2228031	0.30172952	2.70E-06	3.18E-05
Znrf3	469.295469	-1.1958499	-0.2580363	0.12241317	0.01313399	0.04886624
Zpbp2	854.181595	1.2198361	0.28668731	0.08538691	0.00028903	0.00202618
Zpr1	2352.92	1.07478849	0.10405278	0.05708881	0.0582882	0.15637107
Zranb1	1522.42652	1.05031943	0.07082815	0.07263851	0.29932857	0.49713458
Zranb3	466.804613	-1.0263582	-0.0375343	0.09280673	0.64992821	0.79708764
Zrsr1	97.0678983	-1.0913049	-0.1260542	0.17374702	0.27821304	0.47403611
Zscan18	2.1601309	1.00146173	0.0021073	0.21379135	0.93652082	0.96922329
Zscan2	54.3402892	1.37761488	0.46217263	0.38352092	0.01655953	0.05882856
Zscan20	561.243384	-1.0111421	-0.0159858	0.09808866	0.85379423	0.92164019
Zscan21	344.050964	1.14507713	0.19544477	0.1160695	0.048589	0.13631737
Zscan22	328.651591	1.08514505	0.1178879	0.12117188	0.2429236	0.43289095
Zscan25	605.248903	-1.0508401	-0.0715432	0.08605719	0.36318661	0.56391848
Zscan26	2727.45239	-1.0771834	-0.1072639	0.05478403	0.04230292	0.12290777
Zscan29	1799.31306	1.08814751	0.12187415	0.07718843	0.09001858	0.21613528
Zswim1	942.674017	1.20775903	0.27233264	0.09169161	0.00114703	0.00652751
Zswim3	584.506249	1.28862569	0.36583326	0.1119788	0.00025131	0.00180759
Zswim4	499.216087	-1.0702059	-0.0978884	0.09405879	0.24606605	0.43627766
Zswim6	378.092941	-1.1593356	-0.2132982	0.1287365	0.04535636	0.12951738
Zswim7	40.9028896	1.06186053	0.08659428	0.19404963	0.40973023	0.60896889
Zswim8	3683.11258	1.13902457	0.18779887	0.07532482	0.00764305	0.03124042
Zswim9	545.96337	1.12879416	0.17478242	0.12040515	0.08431351	0.2058426
Zup1	773.07041	1.00223213	0.00321669	0.08186621	0.9627236	0.98251189

Zw10	2285.70598	-1.1197121	-0.1631279	0.0715994	0.01522639	0.05519243
Zwilch	1748.1074	1.05282089	0.07426002	0.06581521	0.23569648	0.42542782
Zwint	1070.49669	-1.0441764	-0.0623655	0.08393381	0.41890875	0.61765897
Zxdb	318.630009	-1.1500342	-0.2016767	0.14021898	0.07071128	0.1804046
Zxdc	486.48735	1.02650746	0.03774412	0.09365282	0.65538797	0.80041185
Zyg11a	191.27274	2.01751712	1.01258092	0.2366558	1.13E-06	1.44E-05
Zyg11b	1042.23816	1.12188229	0.16592132	0.08707105	0.03696181	0.11062634
Zyx	2225.92506	1.06244092	0.08738262	0.07780798	0.22765509	0.41631144
Zzef1	1629.64957	1.1549806	0.20786862	0.06073713	0.00034753	0.00236676
Zzz3	3917.53421	1.03076054	0.04370922	0.05741397	0.42983059	0.62762223
ctsz	15114.8824	1.26258904	0.33638513	0.04695246	2.42E-13	8.21E-12
